# P-Stereogenic Ir-MaxPHOX: A Step toward Privileged Catalysts for Asymmetric Hydrogenation of Nonchelating Olefins

**DOI:** 10.1021/acscatal.2c05579

**Published:** 2023-02-14

**Authors:** Maria Biosca, Pol de la Cruz-Sánchez, Jorge Faiges, Jèssica Margalef, Ernest Salomó, Antoni Riera, Xavier Verdaguer, Joan Ferré, Feliu Maseras, Maria Besora, Oscar Pàmies, Montserrat Diéguez

**Affiliations:** † Departament de Química Física i Inorgànica, Universitat Rovira i Virgili, C/Marcel·lí Domingo, 1, 43007 Tarragona, Spain; ‡ Institute for Research in Biomedicine (IRB Barcelona), The Barcelona Institute of Science and Technology (BIST), C/Baldiri Reixac, 10, 08028 Barcelona, Spain; § Departament de Química Inorgànica i Orgànica, Secció Química Orgànica, Universitat de Barcelona, Martí i Franquès 1, 08028 Barcelona, Spain; ∥ Departament de Química Analítica i Química Orgànica, Universitat Rovira i Virgili, C/Marcel·lí Domingo, 1, 43007 Tarragona, Spain; ⊥ Institute of Chemical Research of Catalonia (ICIQ), The Barcelona Institute of Science and Technology, Avenida Països Catalans 16, 43007 Tarragona, Spain

**Keywords:** asymmetric hydrogenation, iridium, P−N ligands, DFT calculations, olefins

## Abstract

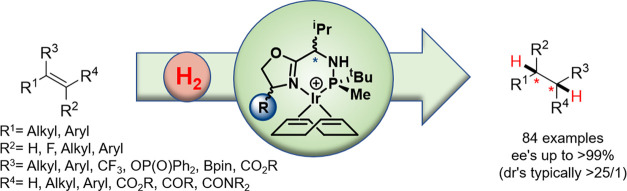

The Ir-MaxPHOX-type catalysts demonstrated high catalytic performance in the hydrogenation of a wide range of nonchelating olefins with different geometries, substitution patterns, and degrees of functionalization. These air-stable and readily available catalysts have been successfully applied in the asymmetric hydrogenation of di-, tri-, and tetrasubstituted olefins (ee′s up to 99%). The combination of theoretical calculations and deuterium labeling experiments led to the uncovering of the factors responsible for the enantioselectivity observed in the reaction, allowing the rationalization of the most suitable substrates for these Ir-catalysts.

## Introduction

Advances in the synthesis of chiral molecules, whether creating new compounds or improving existing synthetic procedures, are made possible by the continuous innovations in asymmetric catalysis.^1^ Among the asymmetric catalytic reactions that lead to enantiomerically pure products, the hydrogenation of olefins is one of the most powerful.^1,2^ This 100% atom economy process has a large record of successful examples in the production of single enantiomer intermediates, especially in the pharmaceutical industry, using substrates ranging from olefins with coordinating functional groups to nonfunctionalized counterparts, passing through olefins with intermediate coordinating properties.^3^ As the number of substrates continues to increase to reach more complex molecules, finding a catalyst that performs well with many of them regardless of geometry, substitution patterns, and functionalization remains a challenge. While Rh- and Ru-catalysts (mainly with diphosphine ligands) have been shown to be optimal for the reduction of olefins with strong coordinating functional groups,^4^ the Ir–P,X-catalysts (X = N, S, and O; mainly with phosphine/phosphinite/phosphite–oxazoline ligands) gave the best results for the hydrogenation of nonchelating alkenes.^5^ Particularly, the reduction of nonchelating olefins is the most difficult and less explored field since they do not have a coordinating group to help transfer the chiral information to the product. Currently, Ir-catalysts only perform well for specific types of olefins. The most common substitution patterns are *E*-trisubstituted alkenes and, to a lesser extent, *Z*-trisubstituted and 1,1-disubstituted alkenes. The hydrogenation of tetrasubstituted olefins is the least developed category.^5^ Even for the most studied trisubstituted olefins, there is still room for improvement. For example, the reduction of the so-called purely alkyl-trisubstituted olefins, those without functional groups or aryl substituents, has been achieved in very few cases^6^ and the effectiveness for exocyclic substrates needs to be improved.^7^ For tetrasubstituted olefins, only a few specific Ir-catalysts have provided high performance for certain substrates, with variable enantioselectivity and low functional group tolerance. Most of the substrates studied were restricted to cyclic olefins and only a few were acyclic, mainly trimethyl styrene derivatives,^7b,8^ until recently when Gosselin′s group in collaboration with Bigler, Pfaltz, and Denmark^9^ presented the reduction of a wide range of acyclic olefins with two or more aryl substituents. In addition, there are fewer reports of tetrasubstituted olefins with poorly coordinative groups that are useful for further synthesis, and, in most cases, the same catalyst was unsuccessful for tetrasubstituted olefins without a poorly coordinative group.^10^ The finding of a catalyst that could work on all of them is highly desirable to limit time-consuming catalyst design and avoid a variety of preparation methods.

The bottleneck in finding the best catalysts is the identification of the right ligands with a broad substrate scope.^11^ To overcome the substrate scope limitation in the asymmetric hydrogenation of nonchelating olefins, we recently reported on the first P,N-ligand library that could reduce different types of nonchelating olefins.^7b^ From a common backbone, the selection of the phosphite or phosphinite group led to ligands that were suitable for 56 examples of di-, tri-, and tetrasubstituted olefins. However, only 11 examples of tetrasubstituted olefins could be reduced, mainly indene derivatives and some acyclic olefins, to the detriment of tetrasubstituted acyclic alkenes with relevant poorly coordinative groups. Even for trisubstituted olefins, only one example of *Z*-olefin was successfully reduced and none of purely alkyl-substituted. Later on, we reported the successful application of a family of P-stereogenic aminophosphine-oxazoline (MaxPHOX) ligands in the Ir-catalyzed hydrogenation of the aforementioned unfunctionalized tetrasubstituted olefins and also in the reduction of several tetrasubstituted substrates with poorly coordinative groups, such as acyclic-tetrasubstituted vinyl fluorides with ester functionalities.^8c^

**Figure 1:**
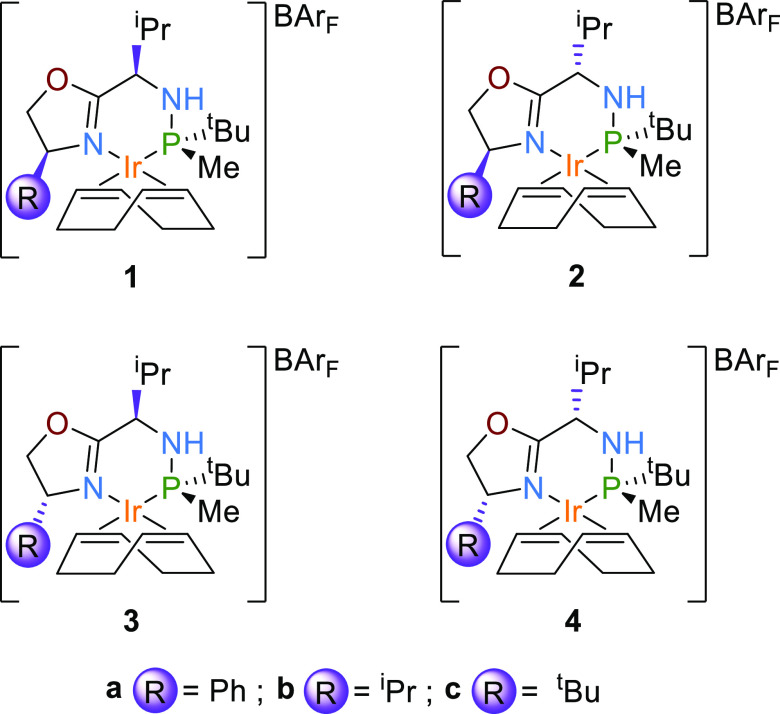
Family of aminophosphine-oxazoline iridium(I) catalysts (Ir-MaxPHOX) **1**-**4a**-**c**.

To advance the search for a ligand library capable of hydrogenating a larger range of substituted nonchelating olefins, here we report an extension of the scope of olefins that Ir-MaxPHOX-type catalysts can successfully reduce. With the Ir-MaxPHOX **1**-**4a**-**c** family of catalysts (Figure 1), we have been able to hydrogenate, with high catalytic performance, a wide range of di- and trisubstituted olefins and we have also increased the number of tetrasubstituted olefins containing neighboring poorly coordinative polar groups that could be used successfully. These catalysts have the advantage that they are prepared in four steps from available starting materials^12^ and allow us to easily study the effect of varying some ligand properties, such as the bulkiness of the oxazoline and its configuration and the configuration of the stereogenic center at the alkyl backbone chain. Together with mechanistic studies based on density functional theory (DFT) calculations and deuterogenation experiments, we were able to explain the origin of enantioselectivity, identify the preferred pathway, and predict enantioselectivities with good accuracy.

## Results and Discussion

### Initial Catalytic Screening

As mentioned in the introduction, the hydrogenation of nonchelating olefins depends largely on the substitution pattern of the substrate. The most successful examples have been reported for *E*-trisubstituted, while 1,1′-disubstituted olefins are usually hydrogenated less enantioselectively and tetrasubstituted olefins are still underdeveloped.^5^ To explore the scope of the Ir-MaxPHOX catalysts (**1**-**4a**-**c**), we initially applied them in the asymmetric hydrogenation of the nonfunctionalized disubstituted olefin **S1** and the widely used benchmark trisubstituted substrate **S2** (Table 1). The initial test conditions were the optimal conditions reported in previous studies with other P,N ligands.^5^ Therefore, the reactions were carried out at room temperature using 1 mol % of the catalyst in dichloromethane under 1 bar of H_2_ for the disubstituted substrate **S1** and 50 bar of H_2_ for the trisubstituted olefin **S2**. The previous results for the model acyclic-tetrasubstituted substrate **S3** were also included in Table 1 for comparison.^8c^

**Table 1:**
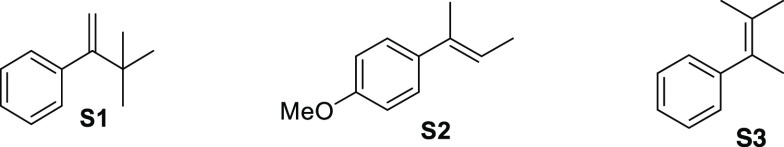
Asymmetric Hydrogenation of Substrates **S1**, **S2**, and **S3**(8c) with Ir-Catalysts **1**-**4a**-**c**^a^

entry	Ir complex	% conv^b^	% ee^c^	% conv^b^	% ee^c^	% conv^b^	% ee^c^
1	**1a**	100	74 (*S*)	100	67 (*R*)	100	75 (*R*)
2	**1b**	100	66 (*S*)	100	75 (*R*)	100	85 (*S*)^d^
3	**1c**	100	81 (*S*)	100	77 (*R*)	85	44 (*R*)
4	**2b**	100	15 (*S*)	100	15 (*S*)	85	33 (*S*)
5	**3b**	100	80 (*R*)	100	23 (*S*)	100	44 (*R*)
6	**4a**	100	83 (*R*)	100	82 (*S*)	100	28 (*R*)
7	**4b**	100	88 (*R*)	100	85 (*S*)	100	25 (*R*)
8	**4c**	100	91 (*R*)	100	88 (*S*)	100	31 (*R*)
9^e^	**4c**	100	91 (*R*)	100	89 (*S*)		
10^e^	**1b**					100	98 (*S*)^f^

a Reaction conditions: catalyst (1 mol %), CH_2_Cl_2_, 1 bar of H_2_ (**S1**), 50 bar of H_2_ (**S2**), or 75 bar of H_2_ (**S3**), rt, 4 h (**S1** and **S2**) or 24 h (**S3**).

b Conversions were measured by ^1^H NMR spectroscopy after 4 h (**S1** and **S2**) or 24 h (**S3**).

c Enantiomeric excess determined by GC.

d Using 2 bar of H_2_—98% (*S*) ee.

e Reactions carried out in PC instead of CH_2_Cl_2_ after 6 h (**S1** and **S2**) and 30 h (**S3**).

f Using 2 bar of H_2_.

For substrates **S1** and **S2**, the best enantioselectivities were obtained with Ir-catalyst **4c** (ee′s up to 91%, entry 8) regardless of the substitution pattern of the substrate. The results showed that both the oxazoline substituent and the diastereoisomeric backbone of the ligand had a noticeable effect on the stereochemical outcome. This effect also occurred in the hydrogenation of the tetrasubstituted olefin **S3**. However, while for the di- and trisubstituted substrates (**S1** and **S2**) the best results were obtained with the bulkier *^t^*Bu group in the oxazoline (e.g., see entry 8 vs. 6–7), the best results for the tetrasubstituted substrate **S3** were obtained with the less bulky *^i^*Pr group, in accordance with the higher steric hindrance of **S3** (entry 2). Similarly, the effect of the diastereoisomeric backbone differed between the di/trisubstituted alkenes **S1** and **S2** and the tetrasubstituted olefin **S3**. While backbone **4** (Figure 1) was best for **S1** and **S2** (ee′s up to 91%), the best backbone for **S3** was **1** (ee′s up to 98% at 2 bars of H_2_, entry 2). In summary, optimizing the ligand structure led us to identify **1b** and **4c** as the best catalysts of the family for the hydrogenation of olefins with different substitution patterns.^13^

To make the process more sustainable, the reaction was carried out in 1,2-propylene carbonate (PC),^14^ an eco-friendly alternative to standard organic solvents due to its high boiling point, low toxicity, and green synthesis (Table 1, entries 9 and 10). Advantageously, enantioselectivities remained as high as those obtained with dichloromethane (ee’s up to 98%). In addition, the catalyst could be recycled up to five times with a simple two-phase extraction with hexane with a minimal decrease in enantioselectivity (see the Supporting Information).

### Mechanistic Studies: The Origin of Enantioselectivity

**Scheme 1:**
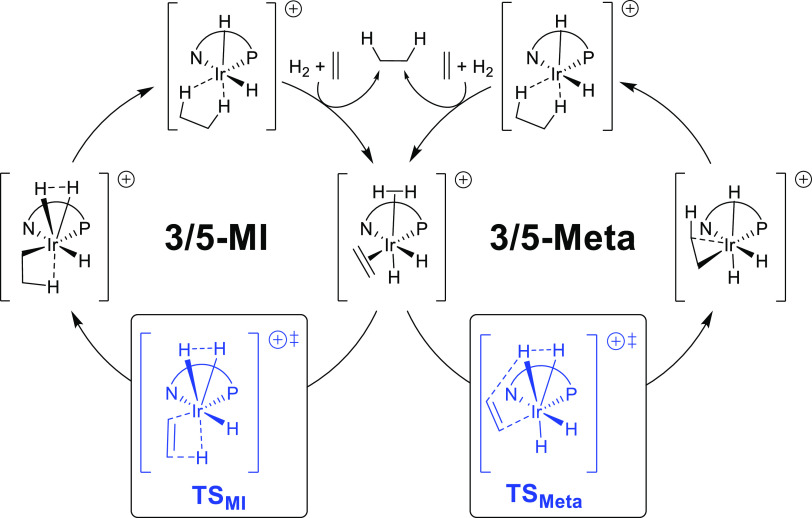
Proposed Catalytic Cycles 3/5-MI and 3/5-Meta for the Asymmetric Hydrogenation of Nonchelating Olefins

To understand why the best ligand for tetrasubstituted olefins is different from that of di- and trisubstituted analogues, we performed a density functional theory (DFT) study. The transition states (TSs) involved in the enantiodetermining step of the reaction for the tri- and tetrasubstituted olefins, **S2** and **S3**, with catalyst **4c** (for **S2**) and catalysts **1b** and **4c** (for **S3**) were searched using the B3LYP^15^ functional with the Grimme Dispersion correction, GD3.^16^ Mechanistically it is well known that Ir-catalyzed hydrogenation of nonfunctionalized alkenes proceeds through an Ir(III)/Ir(V) tetrahydride intermediate^17^ and enantioselectivity is determined in the first hydrogen transfer from the metal to the coordinated olefin. Our calculations also support this mechanism; the free energy reaction profile is presented in the Supporting Information. Consequently, enantioselectivity can be reliably estimated from the relative energies of the TSs of this step. Nevertheless, two different mechanisms can be considered for this process: (i) an Ir(III)/Ir(V) migratory-insertion step (mechanism 3/5-MI, Scheme 1) and (ii) an Ir(III)/Ir(V) σ-bond metathesis (mechanism 3/5-Meta, Scheme 1). While (i) is usually the most favorable mechanism, (ii) is also energetically feasible and cannot be immediately discarded. We, therefore, computed the TSs for both pathways (see the Supporting Information for the full set of calculated TSs). A data set collection of computational results is available in the ioChem-BD repository.^18^

The calculated relative energies for the most stable isomers of the TSs for both pathways (**TS**_**MI**_ and **TS**_**Meta**_) are shown in Table 2. These key isomers are the result of the relative arrangement of the hydride (up or down), the coordination of the olefin through the *Re-* or *Si*-face, and the attack of the hydride through the two olefinic carbons (C_1_ or C_2_). In addition, in these calculations, we also considered the rotamers of the isopropyl group. As in other reported studies, the results show that in all cases, the migratory insertion is the preferred reaction pathway.

**Table 2:**
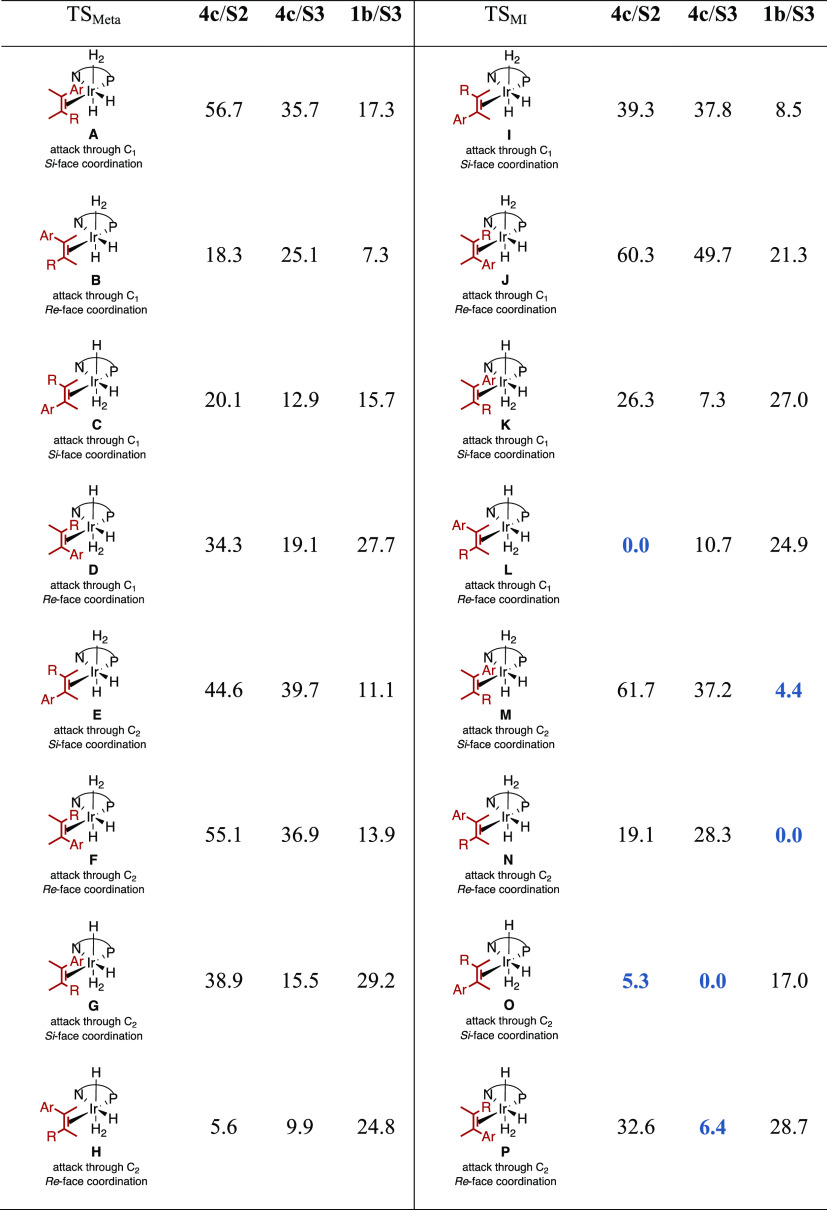
Calculated Relative Energies (kJ/mol) for the Transition States **TS**_**MI**_ and **TS**_**Meta**_ with Substrates **S2** and **S3** Using Ir-Catalyst **4c** (for **S2**) and Ir-Catalysts **1b** and **4c** (for **S3**)^a^,^b^

a Values in blue and bold indicate the lowest *Re* and *Si* energy TSs for each combination of substrate and catalyst.

b Relative Gibbs free energies (kJ/mol) in solution (B3LYP-D3/6-31G(d,p)&LANL2DZ) with respect to the corresponding lowest energy transition state; for **S2** Ar = 4-CH_3_O-C_6_H_4_ and R = H and for **S3** Ar = C_6_H_5_ and R = CH_3_; C_1_ is the least electronegative olefinic carbon atom and C_2_ is the most electronegative one. In all TSs, the most stable rotamer was selected.

**Figure 2:**
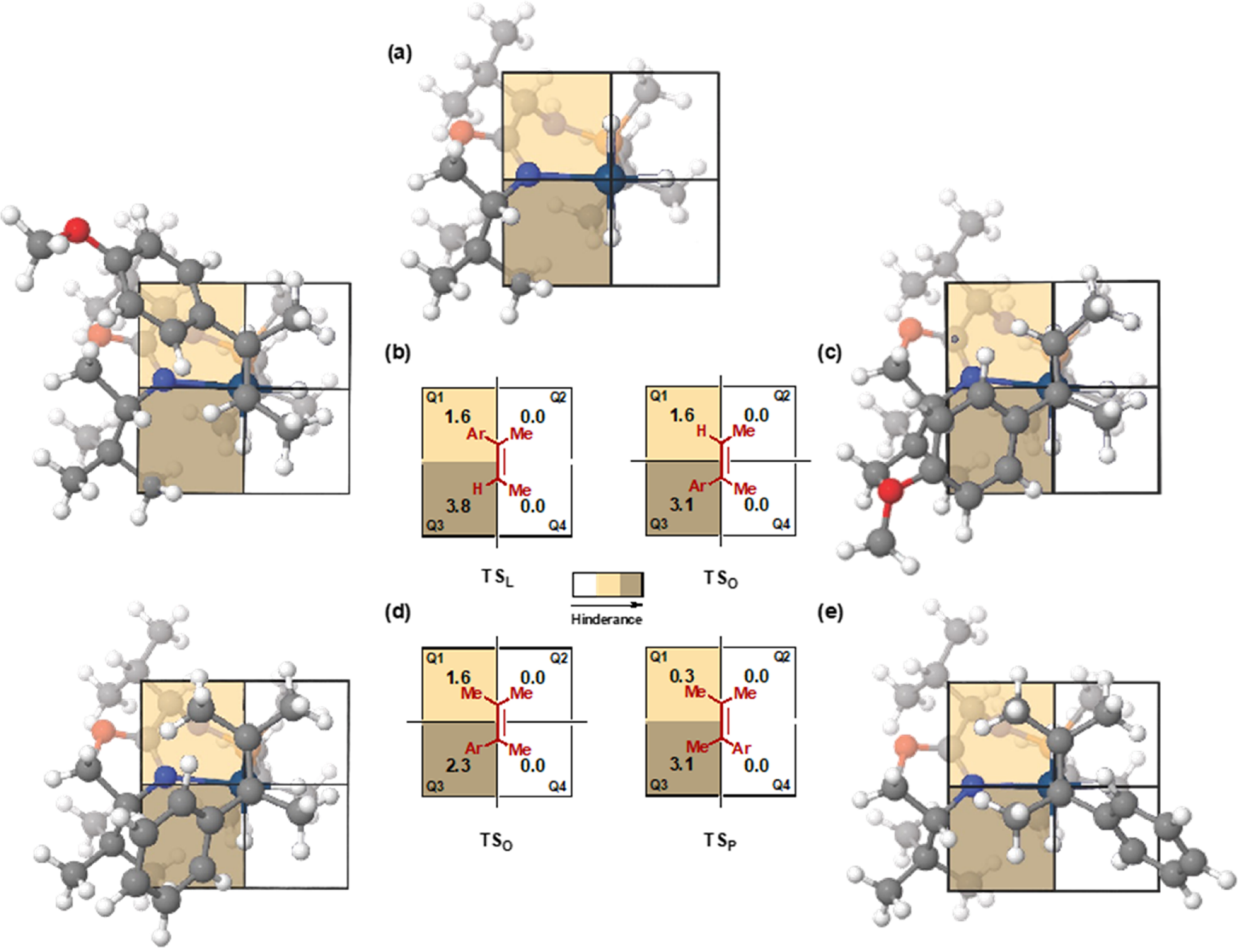
Models of the most favored TSs for the asymmetric hydrogenation of **S2** and **S3** with **4c**; (a) schematic quadrant model for **4c** (the olefin coordinates above the plane of the paper), (b) the most favorable coordination of **S2** giving the major (*S*)-product, (c) the most favorable coordination of **S2** giving the minor (*R*)-product, (d) the most favorable coordination of **S3** giving the major (*R*)-product, and (e) the most favorable coordination of **S3** giving the minor (*S*)-product.

Positively, the calculations for the trisubstituted substrate **S2** with the Ir-catalyst **4c** reproduce the experimental outcome. The favored pathway TS**_L_** (Table 2) proceeds through the *Re*-face, which leads to the formation of the (*S*)-product, and the energy difference between the two most stable TSs (TS_**L**_ and TS**_O_**, Table 2), which lead to opposite enantiomers, is 5.3 kJ/mol (ee_calc_ = 79% (*S*)) in agreement with the experimental enantioselectivity (88% (*S*)). Single-point calculations on the most stable TSs with larger basis sets, B97D3/cc-pVTZ & cc-pVTZ-PP, improve the agreement ee_calc_ = 85% (*S*) (see the Supporting Information for further details). Thus, the factors responsible for enantioselectivity can be deduced by analyzing the structures of both TSs via quantitative quadrant diagram representations using MolQuO^19^ software (Figure 2).

Figure 2a shows the quadrant diagram obtained by analyzing the two most stable TSs for the hydrogenation of **S2** (TS**_L_** and TS**_O_**, Table 2).^20^ In this diagram, the oxazoline substituent (*^t^*Bu) blocks the lower-left quadrant Q3 (quadrant occupancy = 3.8), while the methylenic carbon of the oxazoline partly occupies the upper-left quadrant Q1 (quadrant occupancy = 1.6) making it semihindered (Figure 2a). The other two quadrants, Q2 and Q4, free from bulky groups, are empty (quadrant occupancy = 0). According to this model, the coordination of the trisubstituted olefin **S2** through the *Re*-face is favored because the smallest substituent, the olefinic hydrogen, is located in the most hindered quadrant Q3 and the aryl substituent (4-OMe-C_6_H_5_) is located in the semihindered quadrant Q1 (Figure 2b). In contrast, when the olefin coordinates through the *Si*-face, which leads to the opposite enantiomer ((*R*)-enantiomer, TS**_O_**, Table 2), the aryl group is located at the most hindered quadrant resulting in a less favorable TS (Figure 2c). The occupancy value for this quadrant (3.1) is slightly lower than that obtained for the TS leading to the major product, indicating that the ligand adapts its chiral pocket to suit the olefin in this coordination manner. It is noteworthy that all TSs with the methyl group located in Q3 are less stable, at least 26.3 kJ/mol higher in energy than the most stable one. Note that despite the small size of a methyl group, the flat 4-MeO-C_6_H_5_ group fits better into the cavity in Q3. In summary, the model indicates that the stereochemical outcome with trisubstituted olefin **S2** depends on steric factors. Following this observation, it can be hypothesized that the catalyst may also work for other aryl-containing trisubstituted olefins, including the less studied triaryl trisubstituted and *Z*-olefins (see Table 3 below), where the TS with olefinic hydrogen located in the most hindered quadrant Q3 will continue to be more stable than a TS with the aryl substituent (for triaryl olefins) or the methyl substituent (for *Z*-olefins) in Q3. In addition, this model suggests that if the olefinic aryl group is replaced by a bulkier substituent (e.g., purely alkyl-substituted olefins), then a higher destabilization of the TS_**O**_ could be expected, resulting in a higher energy gap between the TSs and high enantioselectivity (see results for **S20** and **S21**, Table 3 below).

**Table 3:**
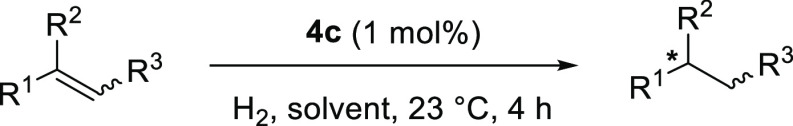

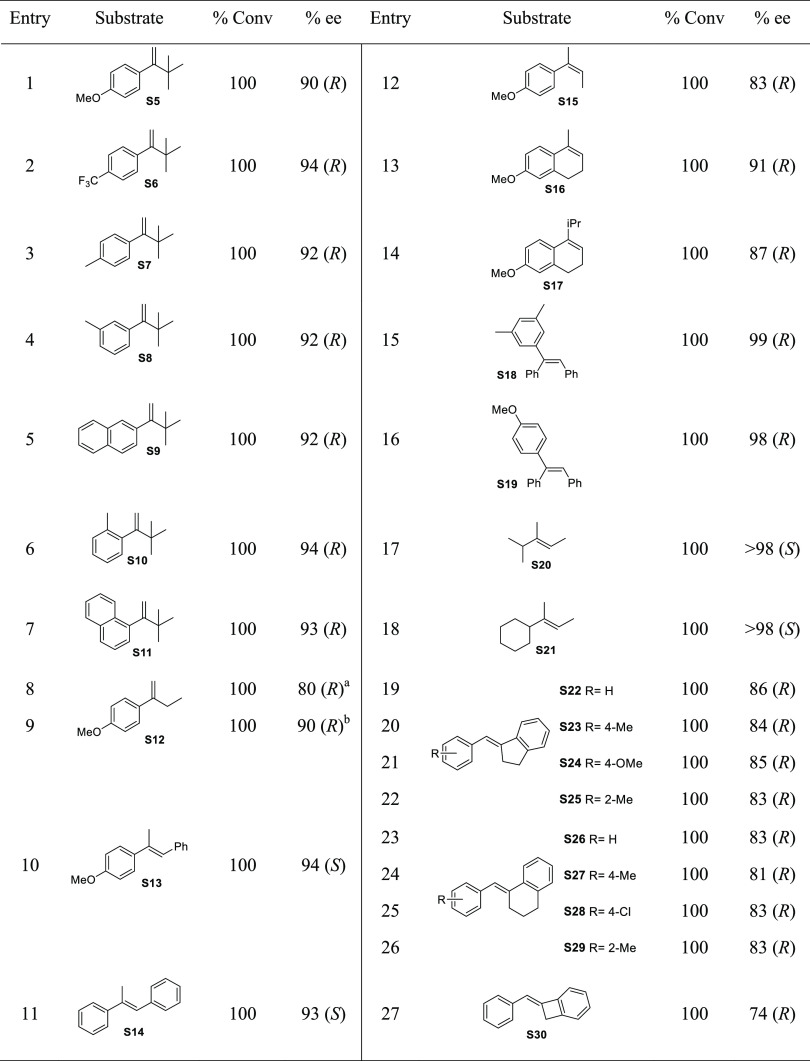
Asymmetric Hydrogenation of Nonfunctionalized Trisubstituted Olefins with Only Aryl and/or Alkyl Substituents **S5**–**S30**

a Reaction conditions: **4c** (1 mol %), CH_2_Cl_2_, 23 °C, 4 h, using 1 bar of H_2_ for **S5**–**S12** or 50 bar of H_2_ for **S13**–**S30**.

b Reaction carried out using propylene carbonate (PC) as a solvent for 6 h.

**Figure 3:**
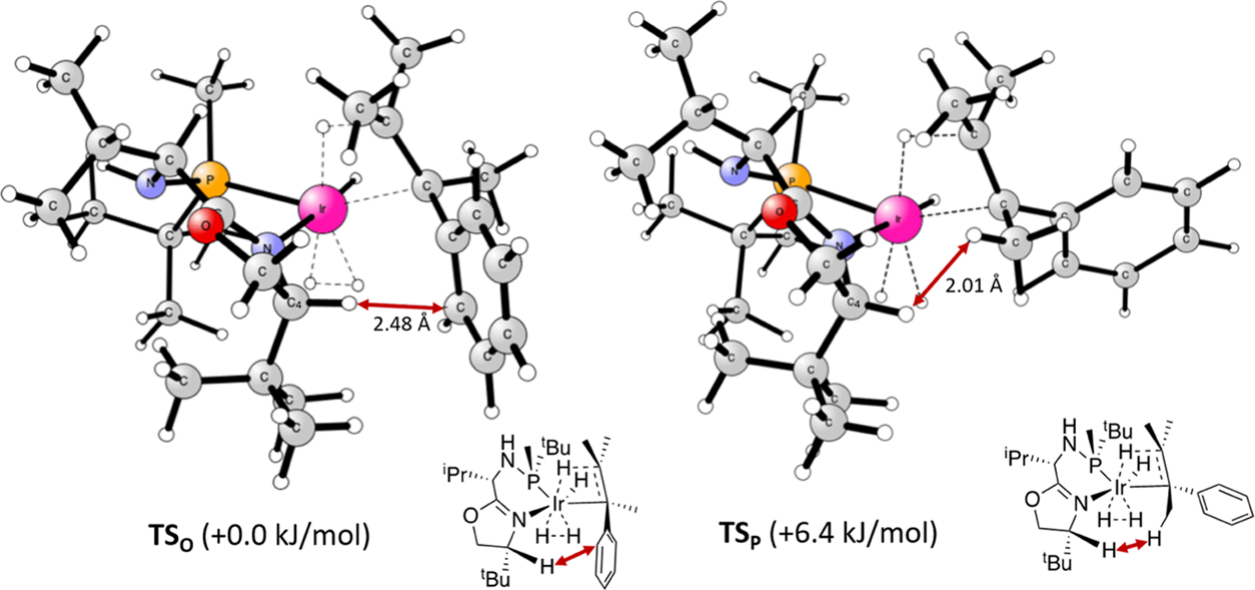
Representation of the two most stable TSs (TS_**O**_ and TS_**P**_) for **4c** and substrate **S3**. Relative Gibbs free energies in solution (kJ/mol) with respect to the corresponding lowest TS.

In contrast, the most favorable TS with the same Ir-catalyst **4c** system but with the tetrasubstituted olefin **S3** was TS**_O_** (Table 2), where the olefin coordinates through the *Si*-face and the (*R*)-enantiomer would be obtained as observed experimentally. The quadrant diagrams of the two most stable TSs (TS**_O_** and TS**_P_**, Table 2) with the tetrasubstituted olefin **S3** and **4c** were analyzed (Figure 2d,e). The diagrams show that the preferred coordination of **S3** is through the *Si*-face with the olefinic phenyl substituent occupying the most hindered quadrant (Q3, Figure 2d), which explains why the enantioselectivity is opposite to that of **S2**. Again, the planarity of the phenyl substituent makes the TS less crowded in Q3 than with a methyl group. This is reflected in the fact that the distance between the hydrogen of the C_4_ of the oxazoline and the olefinic phenyl substituent (TS**_O_**) is greater than the distance between the hydrogen of the C_4_ of the oxazoline and the methyl substituent in the TS**_P_** (Figure 3).

**Figure 4:**
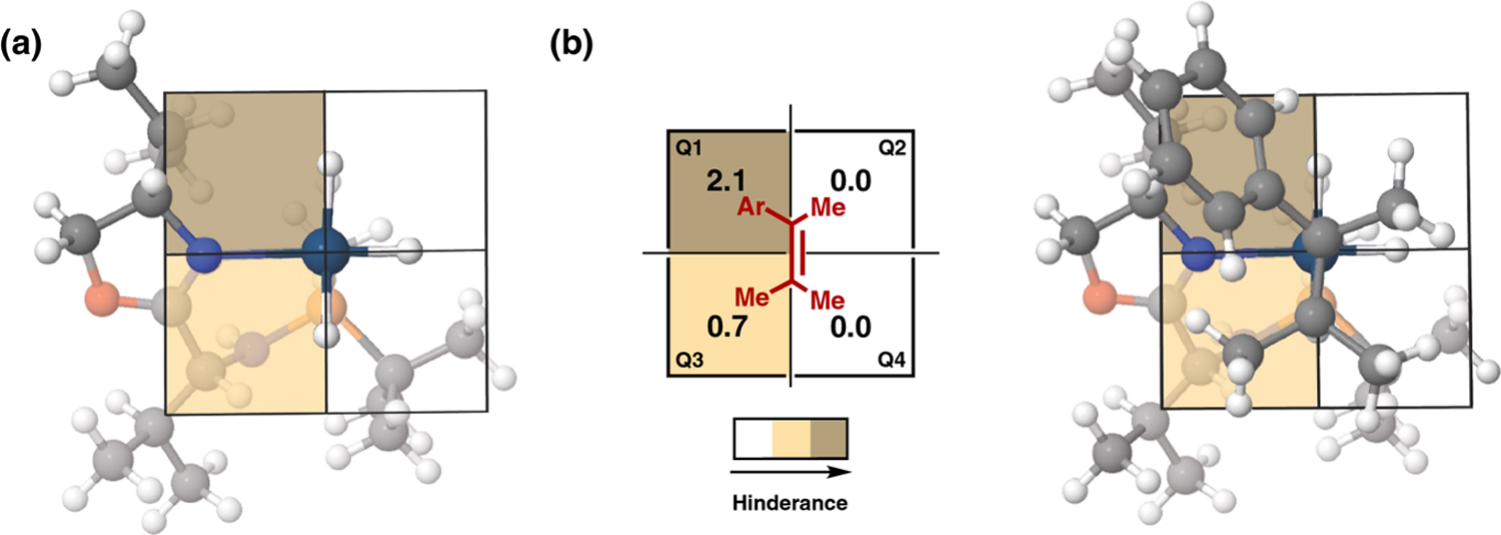
Model of the most favored TS for the asymmetric induction of **S3** with **1b**; (a) schematic quadrant model for **1b** (the olefin coordinates above the plane of the paper) and (b) the most favorable coordination of **S3** giving the major (*S*)-product.

When Ir-catalyst **1b** was used in the hydrogenation of tetrasubstituted olefin **S3**, reverse enantioselectivity was obtained compared to Ir-catalyst **4c**. This can be rationalized by analyzing the quadrant model of the most stable transition state, TS_**N**_ (Table 2), for the hydrogenation of **S3** with **1b** (Figure 4). Ir-catalyst **1b** has the opposite configuration in the oxazoline substituent compared to **4c**, making the upper-left quadrant Q1 the most hindered (Figure 4a). Therefore, the preferred coordination of **S3** is through the *Re*-face (the opposite of **4c**) with the olefinic phenyl located in the most hindered quadrant (Q1) (Figure 4b).

**Scheme 2:**
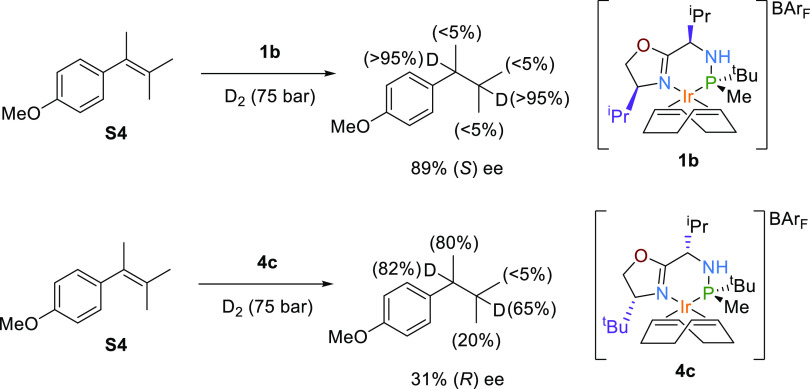
Deuterium Labeling Experiments of the Tetrasubstituted Substrate (**S4**) Percentage of deuterium incorporation is shown in brackets.

Although the sense of enantioselectivity for **S3** was well predicted for both Ir-catalysts **4c** and **1b**, the enantioselectivity value was greatly overestimated with **4c** (82% (*R*) B3LYP-D3/6-31G(d,p)&LANL2DZ and 85% (*R*) B97D3/cc-pVTZ&cc-pVTZ-PP//B3LYP-D3/6-31G(d,p)&LANL2DZ predicted ee vs. 31% (*R*) observed ee). To explain this disagreement, we conducted deuterium labeling experiments with **1b** and **4c** (Scheme 2) in which the related tetrasubstituted olefin **S4** was reduced with deuterium. Note that in these deuterogenation experiments, we used substrate **S4**, which differs from the tetrasubstituted olefin **S3** in a methoxy group in the aryl group, which was introduced to facilitate product analysis. Both substrates performed in the same way. As expected, no deuteration at the methyl groups was observed using **1b**. However, in the case of **4c**, a substantial deuteration was found at the allylic position, indicating the existence of a competing isomerization process. This isomerization would explain the lower enantioselectivity observed when using **4c** in the hydrogenation of tetrasubstituted alkenes such as **S3** or **S4** (Table 1, entry 2 vs. 7).

### Substrate Scope

We first evaluated the Ir-precatalysts **1**-**4a**-**c** in the reduction of a wide range of di- and trisubstituted substrates with *E*- and *Z*-geometries and different neighboring polar groups.

We first focused on the hydrogenation of nonfunctionalized olefins with aryl and/or alkyl substituents only (Table 3). According to the previous screening, Ir-catalyst **4c** was selected for the hydrogenation of a wide range of 1,1′-disubstituted olefins. As expected, this catalyst provided high enantioselectivities (up to 94% ee) for other α-*tert*-butylstyrenes (substrates **S5**–**S11**) with a range of electronic and steric properties at the aryl group. These are significant results because disubstituted substrates suffer more face-selectivity indetermination than the trisubstituted equivalents and therefore there are fewer catalysts^21^ that can provide those high ee′s. Nevertheless, the hydrogenation of α-alkylstyrene **S12**, which has a less bulky ethyl group, proceeded with lower enantioselectivity (ee′ up to 80%) than α-*tert*-butylstyrenes. Although this is still a remarkable result for this challenging substrate, the lower ee was due to the isomerization of **S12** (as observed in deuteration experiments; see the Supporting Information). Thus, like the most successful cases reported in the literature,^22^ the competition between direct hydrogenation and isomerization is responsible for the observed decrease in enantioselectivity. Börner et al. found that the use of 1,2-propylene carbonate (PC) as a solvent reduces the isomerization rate.^14a^ We, therefore, performed the reaction of **S12** in PC and we were glad to see that the enantioselectivity increased to 90% ee (entry 9).

As far as the hydrogenation of aryl trisubstituted olefins is concerned (**S13**–**S19**; Table 3, entries 10–16), the catalyst **4c** also worked well for those with an *E*-geometry, **S13** and **S14** (ee′s up to 94%), which differ from **S2** in the substituent of the aryl ring and the substituent *trans* to the aryl group as well as for the more challenging *Z*-geometry alkenes **S15**–**S17** (ee′s up to 91%). In addition, the substrate scope was extended to the triaryl trisubstituted substrates **S18** and **S19** (ee′s up to 99%), whose reduction has been less studied despite the fact that they are an easy entry point to obtain diaryl methine chiral centers present in natural products and medicines.^23^ These catalytic results are completely consistent with the calculated TSs (vide supra). The analysis of the TSs indicated that the stereochemical outcome for the *E*-olefins mainly depends on steric factors. This finding suggested that enantioselectivities could also be high for substrates such as **S2** that have a bulkier group in the position of the phenyl moiety. This hypothesis was confirmed by the high enantioselectivities (ee′s > 98%) found in the hydrogenation of substrates **S20** and **S21**, which contain a bulky isopropyl and cyclohexyl group, respectively (Table 3, entries 17 and 18).^24^ These are valuable results because the highly enantioselective hydrogenation of purely alkyl substrates is rare,^6^ and indicate that the chiral pocket of the catalyst **4c** is suitable for achieving the hydrogenation of these elusive substrates with excellent enantiocontrol.

The results up to this point led us to test the reduction of exocyclic trisubstituted olefins (**S22**–**S30**, Table 3). The hydrogenation of these substrates is of interest because the chiral benzofused ring motif is present in pharmaceuticals, natural products, and intermediates of relevant bioactive drugs.^25^ Despite the similarities with the acyclic olefins discussed above, the asymmetric hydrogenation of exocyclic olefins has hardly been explored and has yet to be resolved. The main challenge with exocyclic olefins is that the stereochemical outcome is highly influenced by ring size, and until recently, only a few examples had been able to provide high enantiocontrol, particularly for exocyclic olefins with a benzofused 5-membered ring^7a,7b,26^ although enantioselectivity decreased when an *ortho*-substituent was present and required an additive to work.^27^ Positively, the stereochemical outcome using Ir-catalyst **4c** was barely affected by the size of the ring of the substrate, being able to hydrogenate five- and six-membered ring benzofused olefins with high enantioselectivities (up to 86% ee, Table 3) at room temperature without additives. In addition, **4c** tolerates well the presence of several substituents that decorate the aryl group, even an *ortho* group. Note also that, surpassing the previously reported results, the more challenging benzofused olefin with a four-membered ring **S30** could also be hydrogenated with a significant enantioselectivity of 74% ee.

**Scheme 3:**
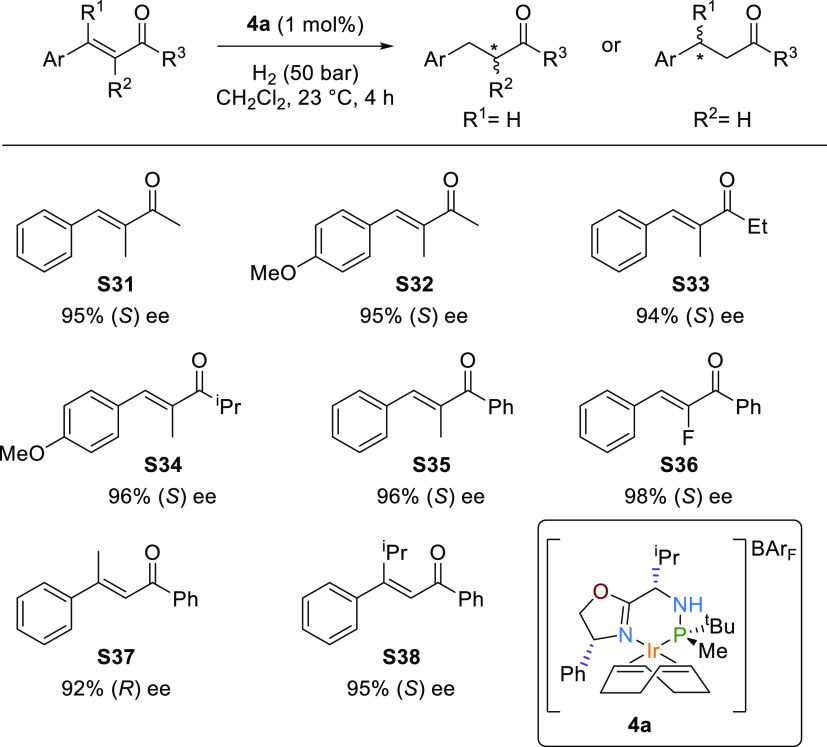
Asymmetric Hydrogenation of α,β- and β,β-Unsaturated Trisubstituted Enones Full conversions were achieved in all cases.

We then moved on to asymmetric hydrogenation of key acyclic olefins with neighboring polar groups. In this context, a set of α,β-unsaturated trisubstituted acyclic enones **S31**–**S36** (Scheme 3) could be hydrogenated with enantioselectivities comparable to the best ones reported but, in contrast to the asymmetric hydrogenation of di- and trisubstituted alkenes mentioned above, this was done with the catalytic system **4a**.^7d−7f,28^ The reduction of these olefins opens a direct, atom-efficient path to prepare optically pure ketones, the synthesis of which until now has been mainly based on noncatalytic methods with a limited substrate scope. The attained enantioselectivities, between 95 and 98% ee, were quite independent of the nature of the substituents, which also allowed the successful hydrogenation of the highly appealing α-fluoride substituted enone **S36**.^29^ It has been reported that the stereochemical outcome in the hydrogenation of acyclic enones is greatly influenced by the enone substitution pattern and, therefore, only a few catalysts have been able to hydrogenate both α,β- and β,β-unsaturated trisubstituted enones with high enantioselectivities.^28c,28d^ Gratifyingly, the catalytic system **4a** also proved to be very efficient in the hydrogenation of β,β-unsaturated enones **S37** and **S38** (Scheme 3).

**Scheme 4:**
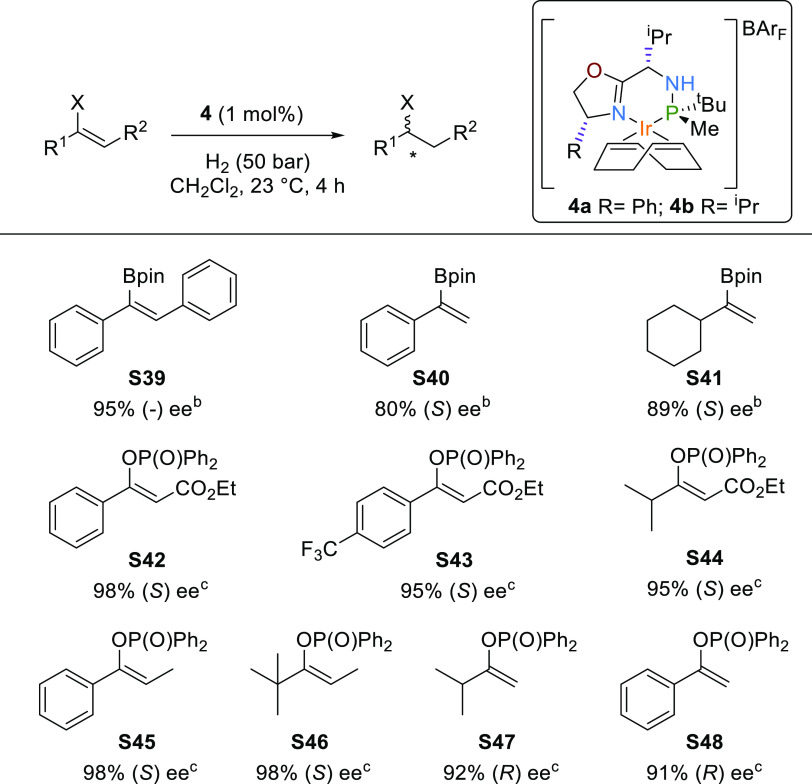
Asymmetric Hydrogenation of Vinyl Boronates **S39–S41** and Enol Phosphinates **S42**–**S48** Full conversions were achieved in all cases. Reactions carried out using **4b**. Reactions carried out with **4a**.

We then tested whether the high enantioselectivities were maintained for acyclic olefins containing other relevant neighboring polar groups (see Scheme 4, substrates **S39**–**S48**). High enantioselectivities up to 98% in alkenyl boronic esters and enol phosphinates were obtained. Among these results, one can highlight the effective hydrogenation of the pure alkyl-trisubstituted enol phosphinates **S44** and **S46**, a good alternative to the hydrogenation of dialkyl ketones to alcohols whose hydrogenation is still elusive. While for the reduction of vinyl boronate, the best enantioselectivity was achieved with **4b** (95% ee); for enol phosphinates, the highest enantioselectivities (up to 98% ee) were with **4a**. Both types of substrates are of interest because their reduction opens up straightforward routes for preparing enantiomerically pure organoboron and organophosphorus compounds, which can be easily transformed into high-value compounds.^30^ The excellent enantioselectivities obtained in the hydrogenation of the trisubstituted alkenyl boronic ester and enol phosphinates were also reached in the even more challenging disubstituted analogues (**S40**, **S41** and **S47**, **S48**; up to 92% ee), including the hydrogenation of nonaromatic disubstituted olefins **S41** and **S47**.

**Scheme 5:**
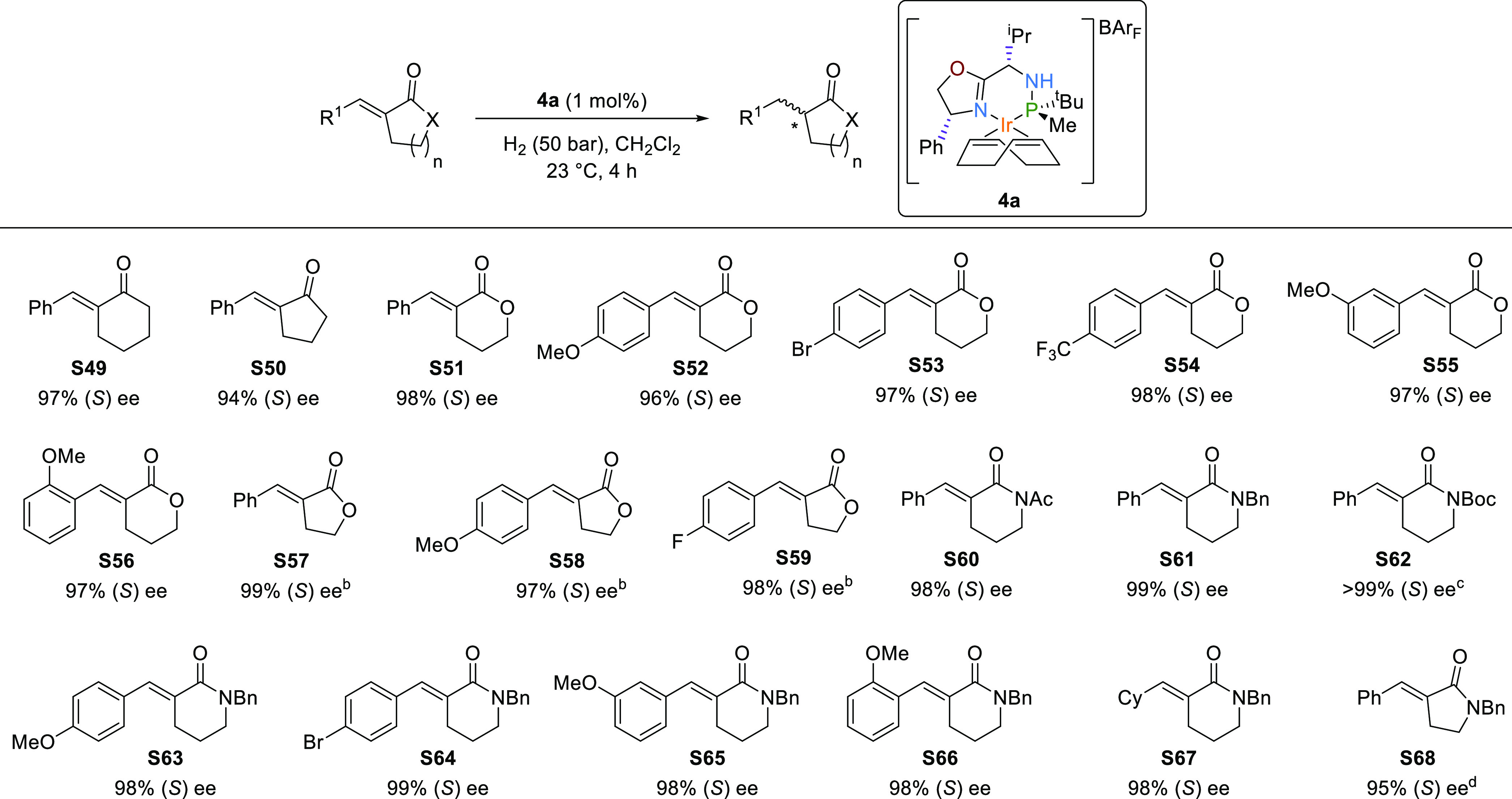
Asymmetric Hydrogenation of Exocyclic α,α-Unsaturated Enones, Lactones, and Lactams (**S49**–**S68**) Full conversions were attained in all cases otherwise noted. Reactions carried out using 2 mol% of catalysts. 28% of deprotected lactam was also obtained. 76% conversion was attained.

Subsequently, we focused on the asymmetric hydrogenation of exocyclic olefins containing a neighboring polar group (Scheme 5, **S49**–**S68**). In particular, we considered the hydrogenation of α,α-unsaturated exocyclic enones and α,α-unsaturated lactones and lactams, since the reduced products of these olefins are encountered in natural products and drugs.^31^ These substrates suffer from the same ring size limitation that was discussed for exocyclic olefins without a neighboring polar group.^7^ In our case, however, the hydrogenation of the exocyclic enones **S49** and **S50** using **4a** proceeded with high enantioselectivities (up to 97%), comparable to the best ones, regardless of the size of the ring. In addition, hydrogenation of α,α-unsaturated lactones (**S51**–**S59**) also proceeded with excellent levels of enantioselectivity (ee′s up to 99%) regardless of the size of the lactone ring. In addition, ee′s were found to be quite independent of the electronic and steric nature of the olefinic substituent. Chiral α-substituted-δ-valerolactones and γ-butyrolactones were therefore attained with ee′s up to 99%. The hydrogenation of α,α-unsaturated lactams (**S60**–**S68**) followed the same trend as related lactones, with ee′s up to >99%. Note that the Ir-catalyst **4a** also allows the presence of different protecting groups, such as Bn, Ac, and Boc, albeit in the latter case, the Boc group can also be partially cleaved under the reaction conditions.

**Scheme 6:**
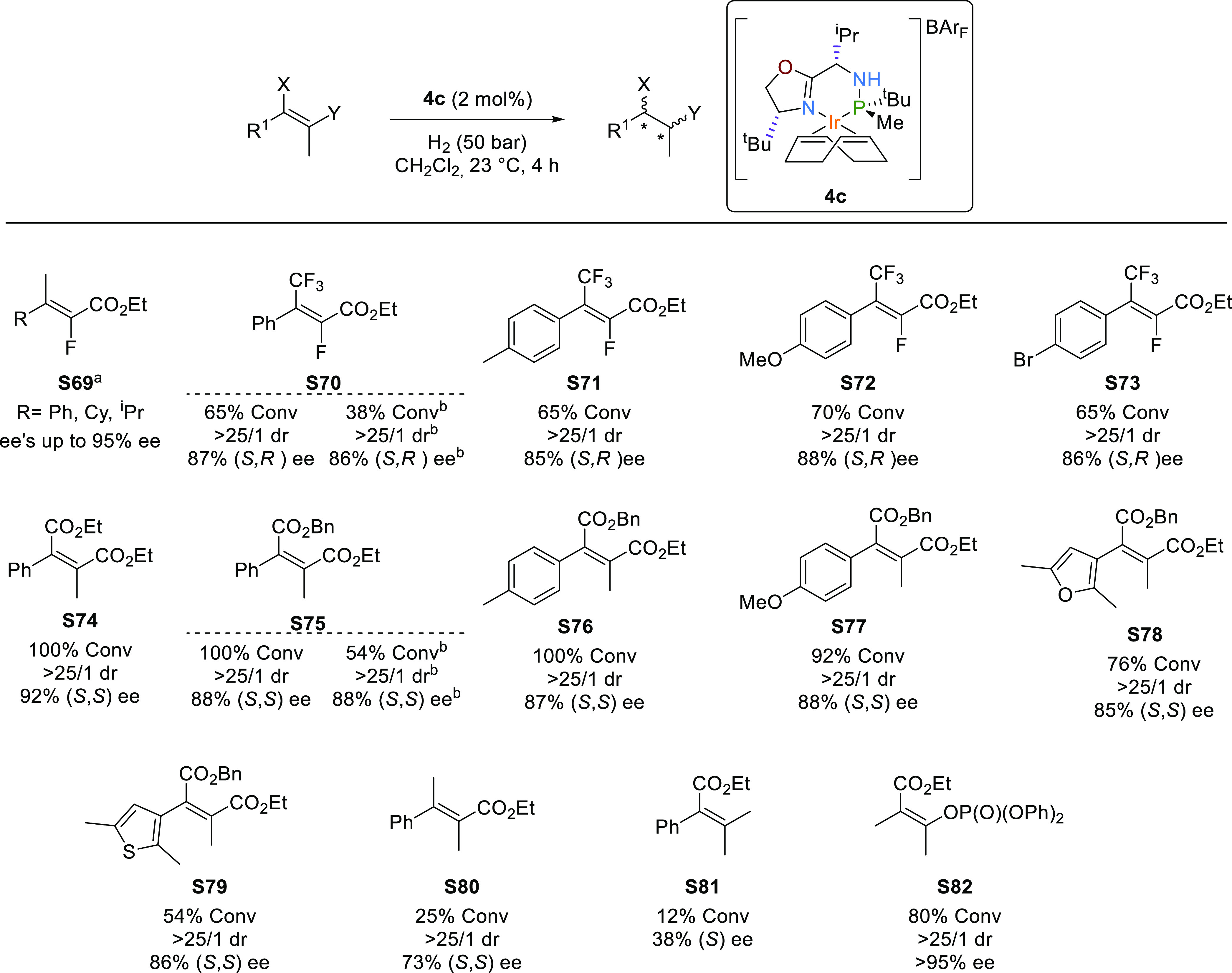
Asymmetric Hydrogenation of Tetrasubstituted Olefins **S69**–**S82** Data from ref (8). Reaction carried out at 1 mol % of catalysts.

Finally, we studied how using Ir-catalysts **1**-**4a**-**c**, we can extend the asymmetric hydrogenation domain to new types of tetrasubstituted olefins. Tetrasubstituted acyclic olefins are considered to be some of the most challenging substrates to be hydrogenated due to the difficulty in differentiating the prochiral faces and the slow activities that result from their steric hindrance. Compared to the progress made with functionalized tetrasubstituted olefins, the reduction of nonchelating tetrasubstituted acyclic olefins remains an open challenge. Furthermore, there are only a few reports on the hydrogenation of tetrasubstituted olefins with poorly coordinative groups that can create intermediates useful for subsequent synthesis.^10^ As mentioned in the Introduction section, the Ir-catalysts **1**-**4a**-**c** were successfully applied in reducing a range of nonchelating tetrasubstituted substrates, most of them without poorly coordinative groups. However, high enantioselectivities were attained in the reduction of several acyclic-tetrasubstituted vinyl fluorides containing an ester functionality such as substrates **S69** type (Scheme 6).^8c^ The challenge of these substrates is that the catalysts must not only control enantioselectivity but also diastereoselectivity (two vicinal stereogenic centers are created) and the defluorination side reaction. We first studied whether we could further expand the previous olefin scope to the reduction of the elusive vinyl fluoride **S70** with an ester functionality and also a CF_3_-functional group instead of the methyl group of **S69**.^32^ Improving on previous results reported in the literature (67% ee)^10c^ the reduction proceeded for the first time with high enantioselectivity (87% ee; Scheme 6), excellent diastereoselectivity without any defluorination with **4c**. The result is in line with the quadrant model developed for **4c** (vide supra, Figure 2a). The smallest substituent of the olefin (F) is placed in the most hindered quadrant (Q3) and the aryl substituent is in the semihindered quadrant Q1. According to this model, the predicted absolute configuration of the reduced product would be 2*S*, 3*R*, in agreement with the experimental result. Positively, the high enantioselectivity was extended for the first time to substrates with different aryl substituents **S71**–**S73** (Scheme 6).

Encouraged by these results, we then studied other functionalized tetrasubstituted olefins lacking a strong coordinative group. Due to the importance of succinic acid derivatives,^33^ we focused on the asymmetric hydrogenation of tetrasubstituted maleates, with two vicinal ester groups (substrates **S74**–**S79**; Scheme 6), as an atom-efficient method for their preparation. The reactions with **4c** proceeded smoothly, providing the hydrogenated products with excellent diastereoselectivity (>25/1 dr) and high enantioselectivities (up to 92%). Moreover, the enantioselectivity was almost unaffected by the electronic nature of the aromatic group (**S75**–**S77**) or the presence of heteroaromatic cyclic substituents (**S78** and **S79**).

Next, we studied whether these results could be reproduced by replacing one of the ester groups with other substituents (Scheme 6). While the exchange of any of the esters by a methyl group (**S80** and **S81**) led to a decrease in activity and enantioselectivity (ee′s up to 73%), positively the reduction of **S82**, with a phosphate instead of one of the ester groups, proceeded with high enantioselectivity (>95% ee) and diastereoselectivity (>25/1 dr), being the first time that this substrate class was hydrogenated.

**Scheme 7:**
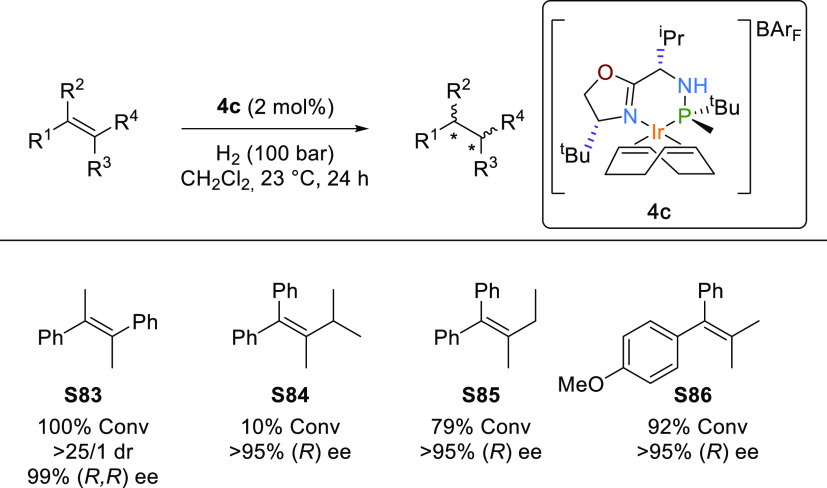
Asymmetric Hydrogenation of Tetrasubstituted Olefins **S83**–**S86**

Based on the recent findings by Gosslein and collaborators of an Ir–P,N catalyst applicable to a wide range of unfunctionalized tetrasubstituted acyclic olefins containing two or three aryl substituents,^9^ the scope of our iridium catalysts **1**–**4** was also studied in the reduction of some of these unfunctionalized olefins (Scheme 7 and the Supporting Information for pressure and catalyst loading effects). Initially, we studied the hydrogenation of substrate **S83**, having two phenyl groups in a *trans* disposition. In agreement with our quadrant model, high diastereo- and enantioselectivities were attained (>25/1 dr and 99% ee). Calculations performed for substrate **S83** with the catalyst **4c** reproduce the enantiomeric excess (computed 99% ee (*R*,*R*)) and support our quadrant model; see the Supporting Information for further details. We then proceed to study several *E*-1,2-dialkyl-1,2-diaryl olefins (**S84**–**S86**). Overcoming the limitations of Gosselin′s system,^9^ our catalyst was able to differentiate the *Re-* and *Si*-faces in substrates differentiated only in the length of an alkyl substituent **S84** and **S85** and in the electronic properties of the aromatic substituents **S86**. Thus, enantioselectivities >95% ee were achieved for these elusive substrate types.

**Scheme 8:**
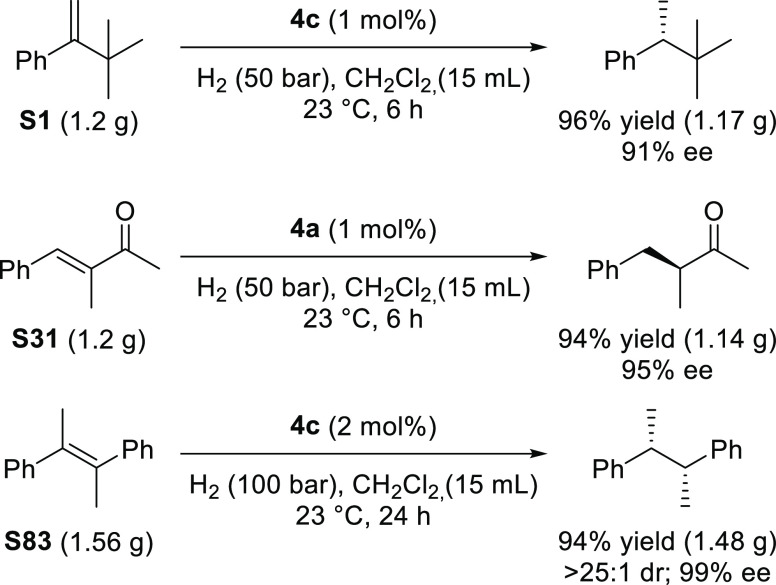
Practical Hydrogenation of **S1**, **S31**, and **S83**

Finally, to show the potential utility of our catalysts, we also carried out the reaction of some representative di-, tri-, and tetrasubstituted substrates (**S1**, **S31**, and **S83**) on a 7.5 mmol scale (Scheme 8).

## Conclusions

In summary, we have shown that Ir-MaxPHOX catalysts (**1**-**4a**-**c**) that had been previously found to be successful in the asymmetric hydrogenation of nonfunctionalized cyclic and few acyclic-tetrasubstituted olefins are also good performers in the hydrogenation of a new set of 84 olefins which included di- and trisubstituted olefins, some with key poorly coordinative groups (such as lactams, lactones, enol phosphinates, ···) and some new examples of challenging tetrasubstituted alkenes. This family of Ir-MaxPHOX-type catalysts allowed the hydrogenation of exocyclic olefins, *Z*-olefins, pure alkyl-substituted olefins, and a broad range of tetrasubstituted olefins, thus improving over a previous family^7b^ also based on P,N ligands, that was so far the only one able to hydrogenate di-, tri-, and tetrasubstituted olefins. DFT calculations and deuterium labeling experiments allowed the rationalization of the stereochemical outcomes of the reactions and helped in the selection of suitable substrates for these Ir-MaxPHOX-type catalysts. The analysis of the TSs indicated that the high catalytic performance of these catalysts is due to their ability to adapt to the demands of each substrate. This ability also explains its excellent performance in the hydrogenation of functionalized olefins such as allyl amines and phthalimides,^34^ cyclic α- and β-enamides,^12^ and imines.^35^ These results open a new perspective for the growth of ligand libraries for the asymmetric hydrogenation of nonchelating olefins, where the Ir/P-stereogenic aminophosphine-oxazoline catalysts could be a good choice for further development.

## Experimental Section

### General Considerations

All reactions were carried out using standard Schlenk techniques under an atmosphere of argon. Solvents were purified and dried by standard procedures. All reagents were used as received. Ir-catalyst precursors **1**-**4a**-**c** were prepared as previously reported.^12^^1^H and ^13^C{^1^H} were recorded using a 400 MHz spectrometer. Chemical shifts are relative to that of SiMe_4_ (^1^H and ^13^C). ^1^H and ^13^C assignments were made based on ^1^H–^1^H gCOSY and ^1^H–^13^C gHSQC.

### Typical Procedure for the Hydrogenation of Olefins

The alkene (0.5 mmol) and Ir complex (1 or 2 mol %) were dissolved in CH_2_Cl_2_ (2 mL) in a high-pressure autoclave, which was purged four times with hydrogen. The apparatus was pressurized to the desired pressure, and after the required reaction time, the autoclave was depressurized and the solvent evaporated. The residue was dissolved in Et_2_O (1.5 mL) and filtered through a short Celite plug.

### Computational Details

All species were optimized using the B3LYP^15^-D3^16^ functional as implemented in Gaussian 09.^36^ The LANL2DZ^37^ basis set together with the associated pseudopotential was used for iridium, and the 6-31G**^38^ basis set was used for all other atoms. Implicit solvation using the PCM^39^ model with the parameters for dichloromethane was included in geometry optimizations. The reported energies are Gibbs free energies in solution within the quasi-harmonic approximation to the Rigid Rotor Harmonic Oscillator Model proposed by Cramer and Truhlar;^40^ corrections were done using the GoodVibes program.^41^ Single-point calculations were done using Grimme’s standalone pure functional B97D3^42^ and the larger basis sets cc-pVTZ^43^ for all atoms except for Ir, for which the cc-pVTZ-PP^44,45^ basis sets were used instead (see the Supporting Information).

Quadrant analysis was done by means of MolQuO (Quantitative Quadrant Diagram Representation of Molecular Systems).^19^ Note that this analysis was done taking the geometry of the whole TS, as shown in the figure, but removing the atoms of the olefin in the MolQuO calculation.

## Supporting Information Available

Calculated energies and computed cartesian coordinates for all TSs; synthesis of substrates; characterization details and enantiomeric excess determination of hydrogenated products; copies of NMR spectra and GC or HPLC traces for ee determination of hydrogenated products, and hydrogenation experiments carried out in PC and deuterogenation experiments (PDF)
