# Supplementary material for: P-Stereogenic Ir-MaxPHOX: A Step toward Privileged Catalysts for Asymmetric Hydrogenation of Nonchelating Olefins
Source: ACS Catal. 2023 Feb 14;13(5):3020–35. doi: 10.1021/acscatal.2c05579 (PMC9990153; doi:10.1021/acscatal.2c05579)
Supplement: Supplementary file 1 — cs2c05579_si_001.pdf [file cs2c05579_si_001.pdf]

P-Stereogenic Ir-MaxPHOX: a step towards privileged catalysts for asymmetric hydrogenation of non-chelating olefins

Maria Biosca,[a] Pol de la Cruz-Sánchez,[a] Jorge Faiges,[a] Jèssica Margalef,[a] Ernest Salomó,[b] Antoni Riera,[b,c] Xavier Verdaguer,[,b,c] Joan Ferré,[d] Feliu Maseras,[e] Maria Besora[*,a] Oscar Pàmies[a] and Montserrat Diéguez[*,a]*

[a] Departament de Química Física i Inorgànica, Universitat Rovira i Virgili, C/ Marcel·lí Domingo, 1, 43007 Tarragona, Spain

[b] Institute for Research in Biomedicine (IRB Barcelona), The Barcelona Institute of Science and Technology (BIST), C/Baldiri Reixac, 10, 08028 Barcelona, Spain

[c] Departament de Química Inorgànica i Orgànica, Secció Química Orgànica, Universitat de Barcelona, Martí i Franquès 1, 08028 Barcelona, Spain.

[d] Departament de Química Analítica i Química Orgànica, Universitat Rovira i Virgili, C/ Marcel·lí Domingo, 1, 43007 Tarragona, Spain

[e] Institute of Chemical Research of Catalonia (ICIQ), The Barcelona Institute of Science and Technology, Av. Països Catalans 16, 43007 Tarragona, Spain

* E-mail for X.V.: xavier.verdaguer@irbbarcelona.org

* E-mail for M.B.: maria.besora@urv.cat

* E-mail for M.D.: montserrat.dieguez@urv.cat

Contents:

1. Hydrogenation experiments using 1,2-propylene carbonate (PC) as solvent. Catalyst recycling....2
2. Absolute energies and energy corrections....3
3. Free energy profile for the catalytic cycle....7
4. Calculated relative energies for the transition states and quadrant model of substrate **S83** and Ir-catalyst **4c**....8
5. Effect of the DFT functional and basis sets on the computed enantiomeric excess....10
6. Deuterogenation experiments....12
7. Pressure and catalyst loading effects on the hydrogenation of substrates **S83–S86**....14
8. Synthesis of substrates....15
9. Characterization details and methods for ee determination of hydrogenated products....22
10. Cartesian coordinates....187
11. References....212

Hydrogenation experiments using 1,2-propylene carbonate (PC) as solvent. Catalyst recycling

Procedure. The alkene (0.5 mmol) and Ir complex (1 mol%) were dissolved in PC (2 mL) in a high-pressure autoclave, which was purged four times with hydrogen. The apparatus was pressurized to the desired pressure and, after the required reaction time, the autoclave was depressurized. Under a positive stream of argon, the mixture was quickly extracted with hexane (2 mL). To the PC phase the corresponding alkene (0.5 mmol) was added and pressurized back to the desired pressure of hydrogen. The hexane phase was dried over $MgSO_4$, diluted with Et_2O (5 mL), filtered over a celite path and analyzed by GC/MS.

Table S1. Hydrogenation experiments with PC as solvent. Catalyst recycling.

Run	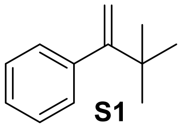 **S1**		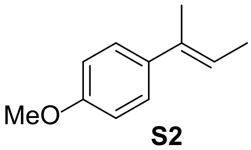 **S2**		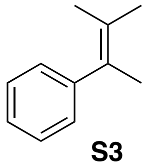 **S3**	
	% Conv[a]	% ee[a]	% Conv[a]	% ee[a]	% Conv[b]	% ee[b]
1	100	91 (*R*)	100	89 (*S*)	100	98 (*S*)
2	98	91 (*R*)	100	88 (*S*)	96	98 (*S*)
3	92	91 (*R*)	94	88 (*S*)	93	98 (*S*)
4	85	90 (*R*)	92	88 (*S*)	86	96 (*S*)
5	80	91 (*R*)	83	88 (*S*)	79	97 (*S*)

[a] Runs carried out using **4c**. [b] Runs carried out using **1b**.

2. Absolute energies and energy corrections

Ir/4c with S2

Transition State	Electronic energy 6-31G**/LANL2DZ (Hartree)	Corrected Gibbs free energy (Hartree)
**A**	-1765.625922	-1764.951191
**B**	-1765.641544	-1764.965819
**C**	-1765.640797	-1764.965113
**D**	-1765.633736	-1764.959712
**E**	-1765.630322	-1764.955802
**F**	-1765.626771	-1764.951789
**G**	-1765.632497	-1764.957969
**H**	-1765.645576	-1764.970631
**I**	-1765.633592	-1764.957800
**J**	-1765.625239	-1764.949785
**K**	-1765.638627	-1764.962754
**L**	-1765.648776	-1764.972771
**M**	-1765.625501	-1764.949284
**N**	-1765.642494	-1764.965487
**O**	-1765.645730	-1764.970756
**P**	-1765.636407	-1764.960337

Ir/4c with S3

Transition State	Electronic energy 6-31G**/LANL2DZ (Hartree)	Corrected Gibbs free energy (Hartree)
**A**	-1690.414403	-1689.742158
**B**	-1690.418792	-1689.746189
**C**	-1690.423283	-1689.750846
**D**	-1690.421018	-1689.748476
**E**	-1690.412647	-1689.740647
**F**	-1690.414153	-1689.741709
**G**	-1690.421918	-1689.749874
**H**	-1690.424761	-1689.751981
**I**	-1690.413910	-1689.741380
**J**	-1690.411086	-1689.736832
**K**	-1690.426316	-1689.752997
**L**	-1690.425008	-1689.751698
**M**	-1690.414083	-1689.741601
**N**	-1690.417874	-1689.744977
**O**	-1690.430074	-1689.755765
**P**	-1690.426653	-1689.753341

Ir/1b with S3

Transition State	Electronic energy 6-31G**/LANL2DZ (Hartree)	Corrected Gibbs free energy (Hartree)
**A**	-1651.107698	-1650.462049
**B**	-1651.111801	-1650.465863
**C**	-1651.107318	-1650.462657
**D**	-1651.103309	-1650.458092
**E**	-1651.110670	-1650.464409
**F**	-1651.107933	-1650.461446
**G**	-1651.103211	-1650.457530
**H**	-1651.105335	-1650.459179
**I**	-1651.111522	-1650.465408
**J**	-1651.107669	-1650.460535
**K**	-1651.104278	-1650.458346
**L**	-1651.104112	-1650.459167
**M**	-1651.114007	-1650.466956
**N**	-1651.116104	-1650.468633
**O**	-1651.109109	-1650.462175
**P**	-1651.103324	-1650.457694

Ir/4c with S83

Transition State	Electronic energy 6-31G**/LANL2DZ (Hartree)	Corrected Gibbs free energy (Hartree)
**A**	-1882.157260	-1881.433495
**B**	-1882.161663	-1881.437816
**C**	-1882.167103	-1881.442736
**D**	-1882.167998	-1881.444183
**E**	-1882.157704	-1881.435090
**F**	-1882.160595	-1881.435390
**G**	-1882.174032	-1881.450089
**H**	-1882.168495	-1881.444352

Free energy profile for the catalytic cycle

Stationary point	Electronic energy 6-31G**/LANL2DZ (Hartree)	Corrected Gibbs free energy (Hartree)
**Int1**	-1262.789051	-1.262.324774
**Int2*Si***	-1690.452916	-1.689.776507
**Int2*Re***	-1690.447125	-1.689.772964
**Int3*Si***	-1690.436842	-1.689.759406
**Int3*Re***	-1690.437432	-1.689.760679
**TS1*Si***	-1690.434960	-1.689.757938
**TS1*Re***	-1690.434985	-1.689.758954
**Int4*Si***	-1690.475910	-1.689.793555
**Int4*Re***	-1690.475505	-1.689.799355
**Int5**	-1261.601894	-1.261.155451
**S3**	-427.627081	-427.446144
**S3-H_2-(*R*)**	-428.858515	-428.651469
**S3-H_2-(*S*)**	-428.858515	-428.651469
**H_2**	-1.178654	-1.179982

3. Free energy profile for the catalytic cycle

From the geometries of the two most stable transition states of tetrasubstituted olefin **S3** with Ir-catalyst **4c** (**TS**$_{\mathbf{O}}$ and **TS**$_{\mathbf{P}}$), we calculated the free energy profiles for the migratory insertion catalytic cycle (Scheme S1). The calculations were carried out using the B3LYP[1] functional with the Grimme Dispersion correction (GD3)[2], the 6-31G(d,p)[3] basis sets were used for all atoms except for iridium for which the LANL2DZ[4] basis sets together with the associated pseudopotential were used. The obtained results agree with the literature[5], showing that the enantioselectivity is determined in the first hydrogen transfer from the metal to the coordinated olefin.

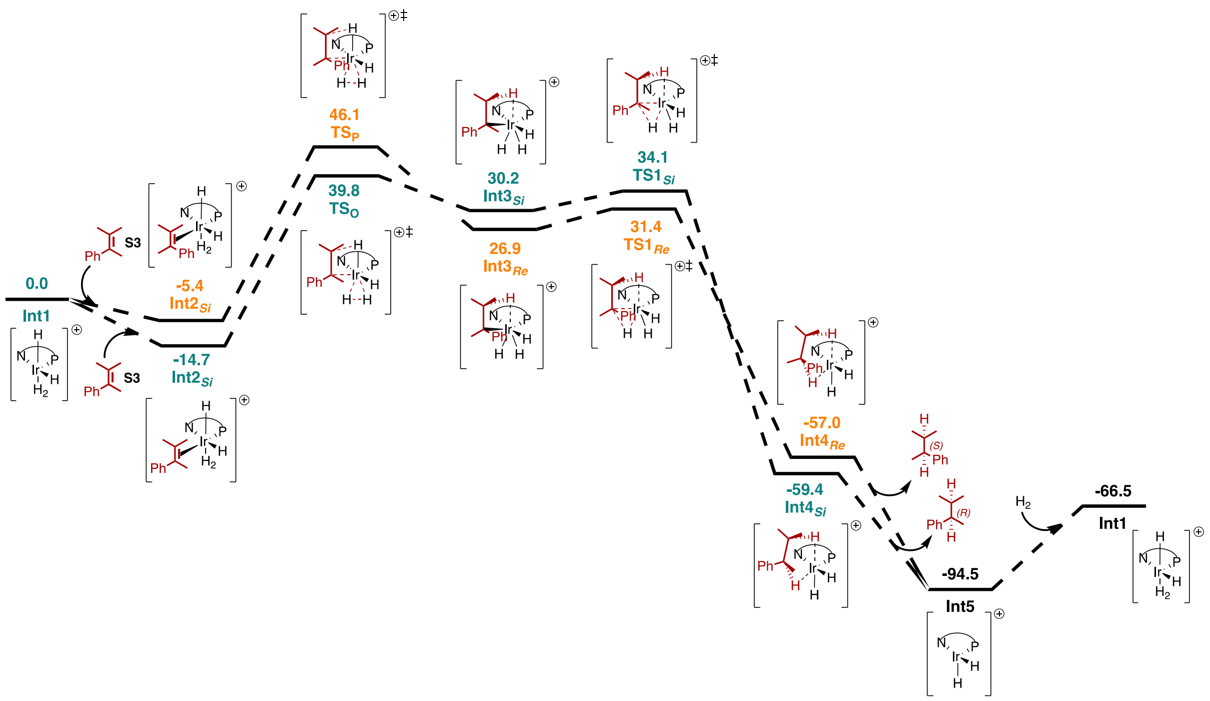

Scheme S1. Calculated free energy profile for the migratory insertion catalytic cycle of tetrasubstituted olefin **S3** with Ir-catalyst **4c**. In blue, calculated free energies in solution (kJ/mol) of the pathway that give the (*R*)-product. In orange, calculated free energies in solution (kJ/mol) of the pathway that give the (*S*)-product.

4. Calculated relative energies for the transition states and quadrant model of substrate S83 and Ir-catalyst 4c

The transition states (TSs) involved in the enantiodetermining step of the reaction for the tetrasubstituted olefin **S83** with catalyst **4c** were located using the B3LYP[1] functional with the Grimme Dispersion correction (GD3)[2], the 6-31G(d,p)[3] were used as basis sets for all atoms except for iridium for which the LANL2DZ[4] basis sets together with the associated pseudopotential were used. The calculated relative energies for the most stable isomers of the TSs for both pathways (**TS_{MI}** and **TS_{Meta}**) are shown in Table S2. These key isomers are the result of the relative arrangement of the hydride (up or down) and the coordination of the olefin through the *Re* or *Si* face. In addition, in these calculations we also considered the rotamers of the isopropyl group. As in the previous calculated substrates, the results show that the migratory insertion is the most favorable reaction pathway.

Table S2. Calculated relative energies (kJ/mol) for the transition states **TS_{MI}** and **TS_{Meta}** with substrate **S83** using Ir-catalyst **4c**. Values in blue and bold indicate lowest *Re* and *Si* energy TSs.

TS_{Meta}	**4c/S83**	TS_{MI}	**4c/S83**
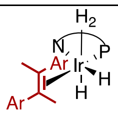 **A** *Si*-face coordination	43.6	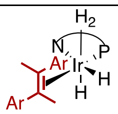 **E** *Si*-face coordination	39.4
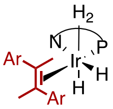 **B** *Re*-face coordination	32.2	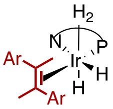 **F** *Re*-face coordination	38.6
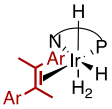 **C** *Si*-face coordination	19.3	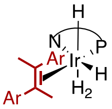 **G** *Si*-face coordination	**0.0**
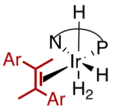 **D** *Re*-face coordination	15.5	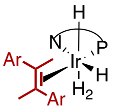 **H** *Re*-face coordination	**15.1**

Relative Gibbs free energies (kJ/mol) in solution (B3LYP-D3/6-31G(d,p)&LANL2DZ) with respect to the corresponding lowest energy transition state; Ar= C_6H_5.

Calculations for the tetrasubstituted substrate **S83** with the Ir-catalyst **4c** reproduce the experimental outcome. The favored pathway proceeds via **TS_G** through the *Si*-face (Table S2) and leads to the formation of the (*R,R*)-product. The energy difference between the two most stable TSs that lead

to opposite enantiomers (**TS**$_G$ and **TS**$_H$, Table S2), is 15.1 kJ/mol (ee$_{calc}$= >99% (*R*,*R*)) in agreement with the experimental enantioselectivity (99% (*R*,*R*)).

The observed enantioselectivity can be rationalized by analyzing the structures of the two most stable TSs for the hydrogenation of **S83** (**TS**$_G$ and **TS**$_H$, Table S2) via quantitative quadrant-diagram representation using the MolQuO software[6]. Figure S1 shows the obtained quadrant diagram supports our previous quadrant model. In this diagram, the oxazoline substituent (^{t}Bu) blocks the lower-left quadrant Q3, while the methylenic carbon of the oxazoline partly occupies the upper-left quadrant Q1 making it semi-hindered (Figure S1a). The other two quadrants Q2 and Q4, free from bulky groups, are empty. The diagrams show that the preferred coordination of **S83** is through the *Si*-face, leading to the (*R,R*)-product, with one of the olefinic phenyl substituents occupying the most hindered quadrant (Q3, Figure S1b). The lowest TS leading to the minor (*S,S*)-product, coordinates the olefin **S83** with a methyl group on the Q3 quadrant (Figure S1c). Again, the planarity of the phenyl substituent makes the TS less crowded in Q3 than with a methyl group. Therefore, the model indicates that again the stereochemical outcome depends on steric factors.

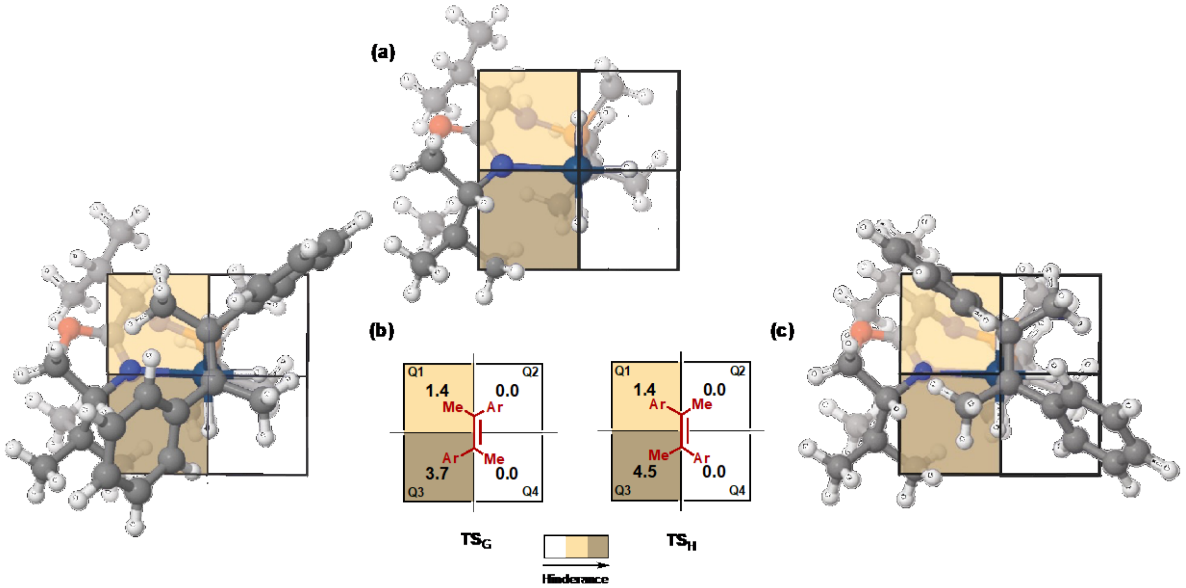

Figure S1. Models of the most favored TSs for the asymmetric hydrogenation of **S83** with **4c**; (a) Schematic quadrant model for **4c** (the olefin coordinates above the plane of the paper), (b) The most favorable coordination of **S83** giving the major (*R,R*)-product, (c) The most favorable coordination of **S83** giving the minor (*S,S*)-product.

5. Effect of the DFT functional and basis sets on the computed enantiomeric excess

We have carried single point calculations using larger basis sets, the cc-pVTZ[7,8] basis sets were chosen for all atoms except for Ir for which the cc-pVTZ-PP[9,10] basis were used instead. Also the effect of different DFT functionals (B3LYP-D3, PBE-D2, PBE-D3, BP86-D3, B97D3, M06-D3, MN15L and TPSS-D3) was investigated. We compared these computational results with the experimentally measured ee's and assuming the observed ee correspond mainly to the lowest pro-*S* and the lowest pro-*R* transition states, the experimentally deduced free energy difference between the pro-*S* and pro-*R* was obtained, see Table S3. We investigated catalyst **4c** with substrates **S2**, **S3** and **S83** and catalyst **1b** with substrate **S3**.

All functionals give values of ee agreeing with the experimental value of > 99 (*R,R*) for catalyst **4c** and substrate **S83**. Also, due to experimentally observed isomerization of olefin **S3** with catalyst **4c**, these results have not been included in Table S3. However, it is to note that all DFT functionals and basis sets predict the formation of the *R* product in agreement with experiments. The DFT predicted enantioselectivity is in all cases higher (57-91% ee (*R*)) than the experimentally observed (31% ee (*R*)), indicating that the observed isomerization very likely has a negative impact in the enantioselectivity and reduces it.

Some differences between functionals appear with catalyst **4c** and substrate **S2** and catalyst **1b** and substrate **S3**. As it can be seen in Table S3, all tested DFT functionals (B3LYP-D3, PBE-D2, PBE-D3, BP86-D3, B97D3 and TPSS-D3) reproduce well the energy differences with errors smaller than 1 kcal.mol^{-1}, except for the Minnesota functionals M06-D3 and MN15L that show larger errors. B3LYP-D3 and TPSS-D3 DFT functionals provide fair results but their estimated errors are of about 1 kcal.mol^{-1} for the free energy difference between the lowest transition states pro-*S* and pro-*R* for catalyst **1b** and olefin **S3**.

The literature recommended PBE-D2 and PBE-D3 functionals[11] for the study of similar iridium catalysed reactions give overall good results, achieving a very good agreement for catalyst **4c** but slightly over stabilizing the pro-*R* transition state for catalysts **1b.** Instead BP86-D3 and B97D3 functionals show also a good agreement with **4c** but a better agreement for catalyst **1b** with errors smaller than 0.4 kcal.mol^{-1}. B97D3 shows overall a better agreement than BP86-D3 and hence we decided to report also the ee's obtained from the single points with the B97D3[12,13] functional and these larger basis sets.

Please note these free energies are obtained as $G_{SP} = G_{OPT} - E_{OPT} + E_{SP}$, where G_{OPT} and E_{OPT} are respectively the free energies in solution and potential energies in solution obtained at the optimization level (B3LYP-D3/6-31G(d,p) & LANL2DZ) and the E_{SP} the potential energy in solution obtained from the single point calculation (B97D3/cc-pVTZ & cc-pVTZ-PP).

Table S3. Experimentally measured and DFT calculated energies and ee %. Energies correspond to free energies in solution and in $kcal.mol^{-1}$ where ΔG = ΔG(TS pro-*S*) – ΔG(TS pro-*R*). Hence, positive values of energy and ee correspond to enantioselective formation of the *S* hydrogenated product and negative values to the formation of the *R* hydrogenated product.

		**4c_S2**	**1b_S3**	**4c_S83**
**Experimental measures**	**ΔG[a]**	**1.6**	**1.5**	**> -3.1**
	**ee %**	**88**	**85**	**> -99**
B3LYPD3<br>6-31g(d,p) & LANL2DZ	ΔG	1.3	1.1	-3.6
	ee %	79	71	-99.5
B3LYPD3<br>cc-pVTZ & cc-pVTZ-PP	ΔG	0.9	0.5	-3.1
	ee %	89	38	-98.9
PBE-D2<br>cc-pVTZ & cc-pVTZ-PP	ΔG	1.1	0.7	-3.5
	ee %	71	53	-99.5
PBE-D3<br>cc-pVTZ & cc-pVTZ-PP	ΔG	1.5	0.6	-2.6
	ee %	85	44	-98
BP86-D3<br>cc-pVTZ & cc-pVTZ-PP	ΔG	1.3	1.5	-3.8
	ee %	79	85	-99.7
**B97D3<br>cc-pVTZ & cc-pVTZ-PP**	**ΔG**	**1.5**	**1.1**	**-3.2**
	**ee %**	**85**	**72**	**-99.1**
M06<br>cc-pVTZ & cc-pVTZ-PP	ΔG	-0.0	0.2	-4.0
	ee %	-3	20	-99.8
MN15L<br>cc-pVTZ & cc-pVTZ-PP	ΔG	0.4	0.7	-5.5
	ee %	33	53	-99.98
TPSSTPSS-D3<br>cc-pVTZ & cc-pVTZ-PP	ΔG	1.1	0.5	-3.0
	ee %	71	40	-98.7

[a] Difference of free energies between the transition states for the formation of the pro-*S* and the pro-*R* hydrogenation products and assuming participation of a TS pro-*R* and a TS pro-*S*.

6. Deuterogenation experiments

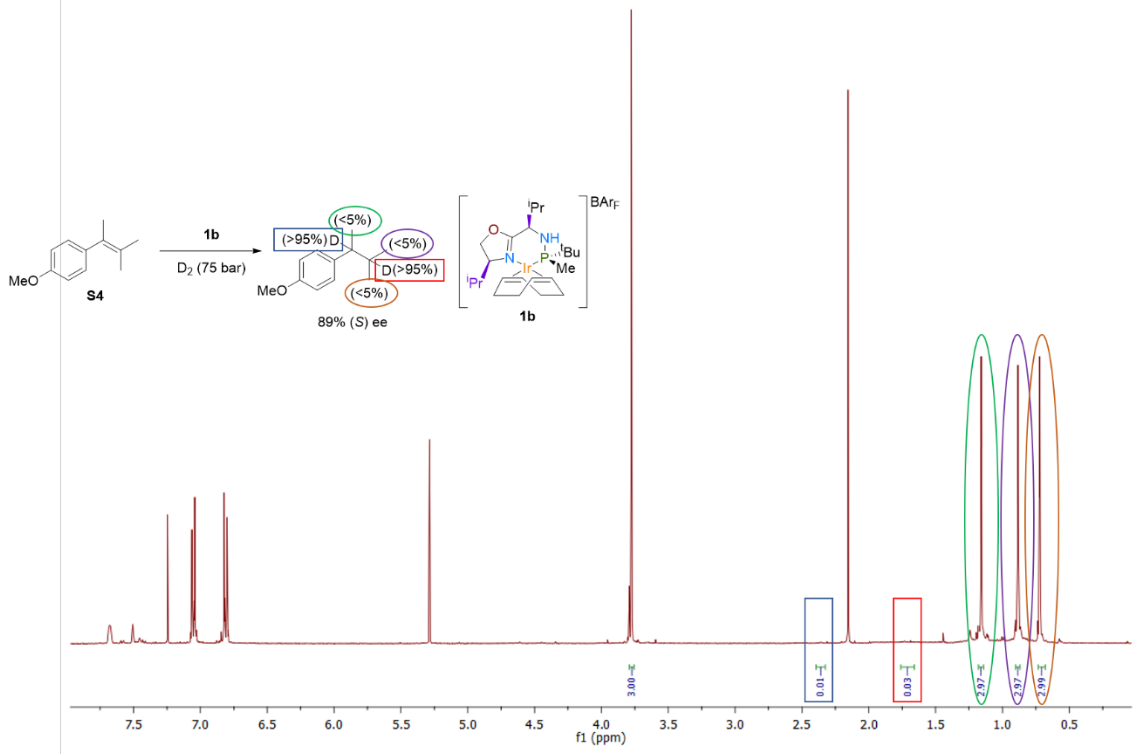

Figure S2. ¹H NMR spectrum of the deuteragenated product from **S4** with **1b**.

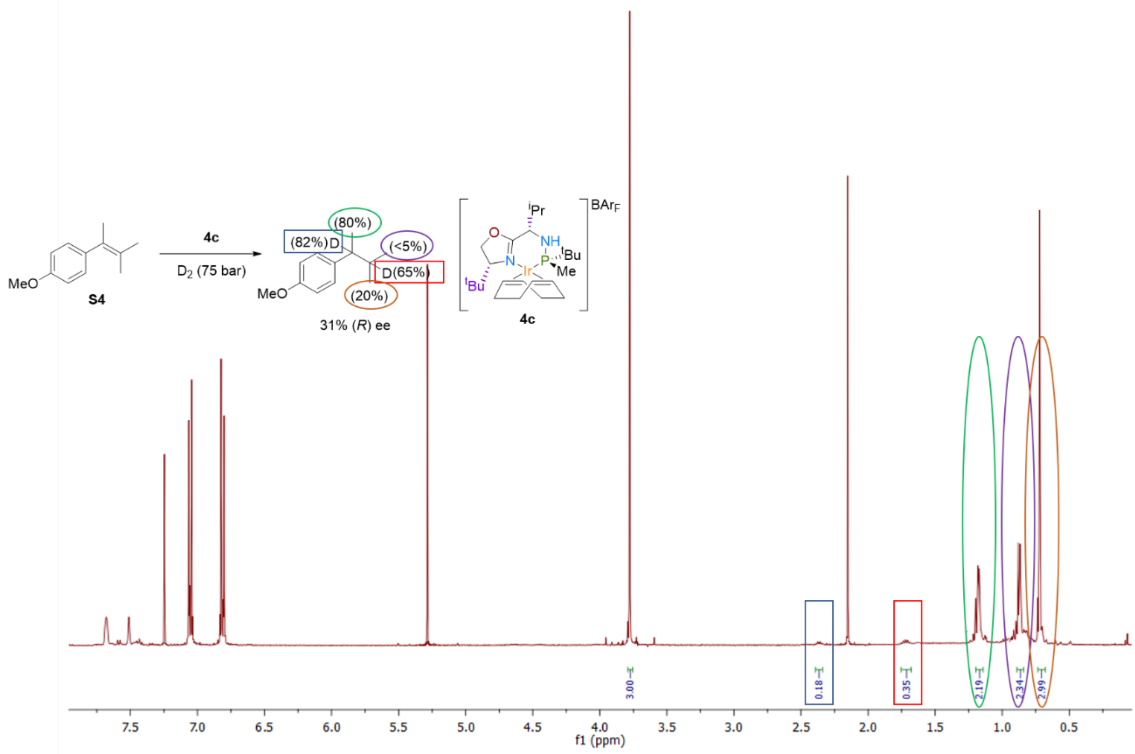

Figure S3. ¹H NMR spectrum of the deuteragenated product from **S4** with **4c**.

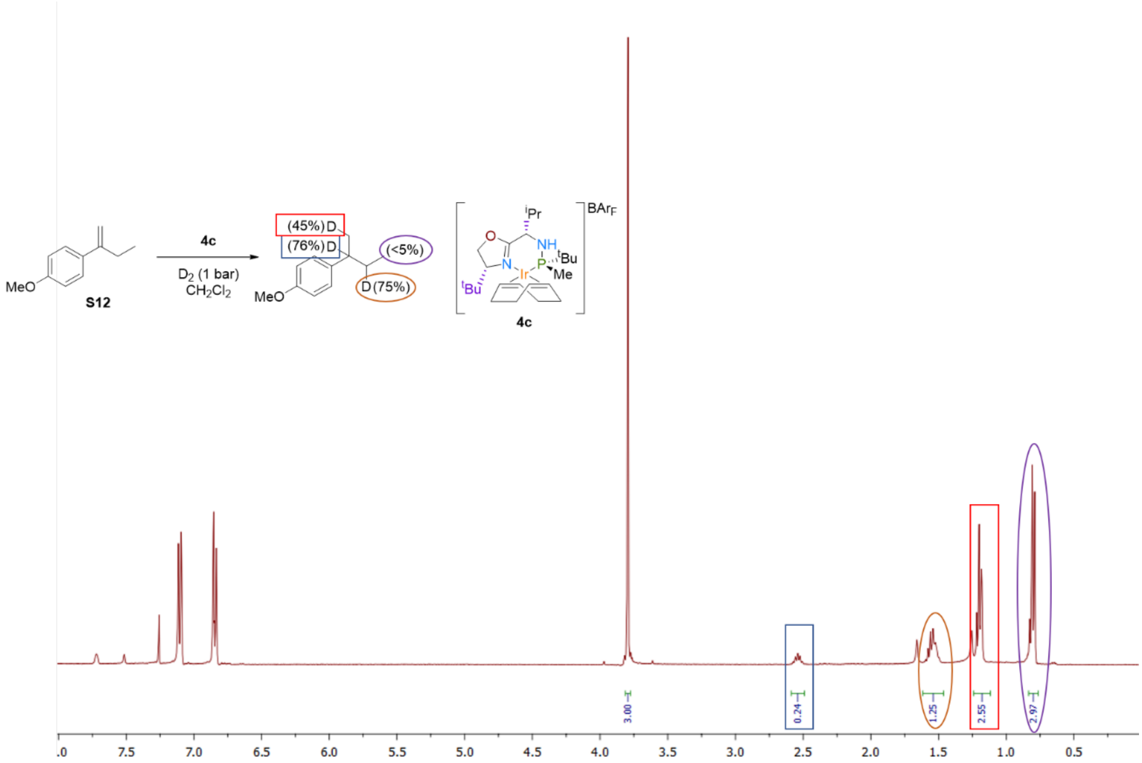

Figure S4. ^{1}H NMR spectrum of the deuteragenated product from **S12** with **4c**.

7. Pressure and catalyst loading effects on the hydrogenation of substrates S83–S86

Table S4. Pressure and catalyst loading effects on the hydrogenation of substrates **S83–S86**.[a]

Entry	P (bar)	Cat. loading (mol%)	Substrate	% Conv[b]	% ee[c]	dr[b]
1	100	2	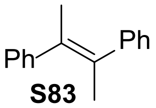 **S83**	100	99 (*R*,*R*)	>25/1
2	50	2	**S83**	74	99 (*R*,*R*)	>25/1
3	100	1	**S83**	89	99 (*R*,*R*)	>25/1
4	100	2	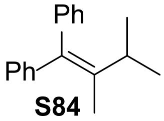 **S84**	10	>95 (*R*)	na
5	50	2	**S84**	<2	nd	na
6	100	1	**S84**	3	nd	na
7	100	2	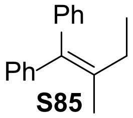 **S85**	79	>95 (*R*)	na
8	50	2	**S85**	<3	nd	na
9	100	1	**S85**	24	>95 (*R*)	na
10	100	2	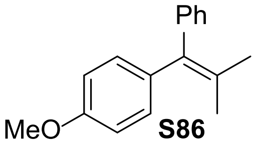 **S86**	92	>95 (*R*)	na
11	50	2	**S86**	58	>95 (*R*)	na
12	100	1	**S86**	41	>95 (*R*)	na

[a] Reactions carried out using **4c** in CH_2Cl_2 at rt for 24 h. [b] Determined by ^1H-NMR. [c] Determined by chiral HPLC. nd= not determined. na= not applicable.

8. Synthesis of substrates

Substrates **S1**[14], **S2**[15], **S3-S4**[16], **S5-S11**[14], **S12**[17], **S13**[18], **S15**[18], **S16-S17**[19], **S18**[20], **S19**[21], **S22-S23**[22], **S24-S25**[23], **S26**[22], **S27-S30**[23], **S31**[24], **S32-S35**[25], **S36**[26], **S37-S38**[27], **S41**[28], **S42-S48**[29], **S49**[30], **S50**[31], **S51**[32], **S52-S56**[33], **S57-S61**[32], **S62**[34], **S63-S66**[33], **S67-S68**[32], **S69-S70**[35], **S74-S81**[36], **S82**[37] and **S83-S86**[38] were prepared following the reported procedures and **S14**, **S20**, **S21**, **S39** and **S40** were commercially available.

.

Procedure for the preparation of olefins S71-S73

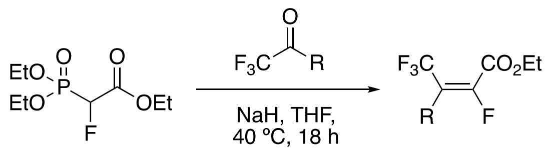

The tetrasubstituted vinyl-fluorides were synthetized according a literature procedure,[35] to a stirred suspension of NaH (1 mmol, 1 equiv., 60 % mineral dispersion) in anhydrous THF (2 mL) at 0 °C corresponding substituted diethoxyphosphoryl-2-fluoroacetate (1.02 mmol, 1.02 equiv.) was added dropwise. The reaction mixture was then heated to 40 °C and stirred for 1 h. After cooling again to 0 °C, the corresponding ketone (1 mmol, 1 equiv.) was added dropwise and the resulting mixture was stirred at 40 °C for another 18 h. After this time, the reaction was quenched with water. The reaction mixture was extracted with diethyl ether (3 x 10 mL). The combined organic layers were dried over Na_2SO_4, filtered, and concentrated under reduced pressure. The crude product was purified by flash chromatography using petroleum ether, Et_2O mixture (95:5) to afford the desired tetrasubstituted vinyl-fluorides.

Ethyl (E)-2,4,4,4-tetrafluoro-3-(p-tolyl)but-2-enoate (S71). Colorless oil. ^{1}H NMR ($CDCl_3$), δ: 1.32 (t, 3H, *J*= 7.2 Hz), 2.32 (s, 3H), 4.33 (q, 2H, *J*= 7.2 Hz), 7.16-7.19 (m, 4H). ^{19}F NMR ($CDCl_3$), δ: -58.51 (d, *J*= 10.1 Hz), -107.39 (q, *J*= 9.9 Hz). ^{13}C NMR ($CDCl_3$), δ: 13.7, 21.3, 63.0, 118.9, 120.9 (d, *J*= 13.8 Hz), 123.7 (d, *J*= 14.0 Hz), 124.4, 129.0 (d, *J*= 2.6 Hz), 129.3, 139.7, 149.8 (d, *J*= 4.6 Hz), 152.5 (d, *J*= 4.5 Hz), 159.6 (d, *J*= 36.0 Hz).

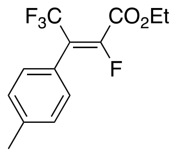

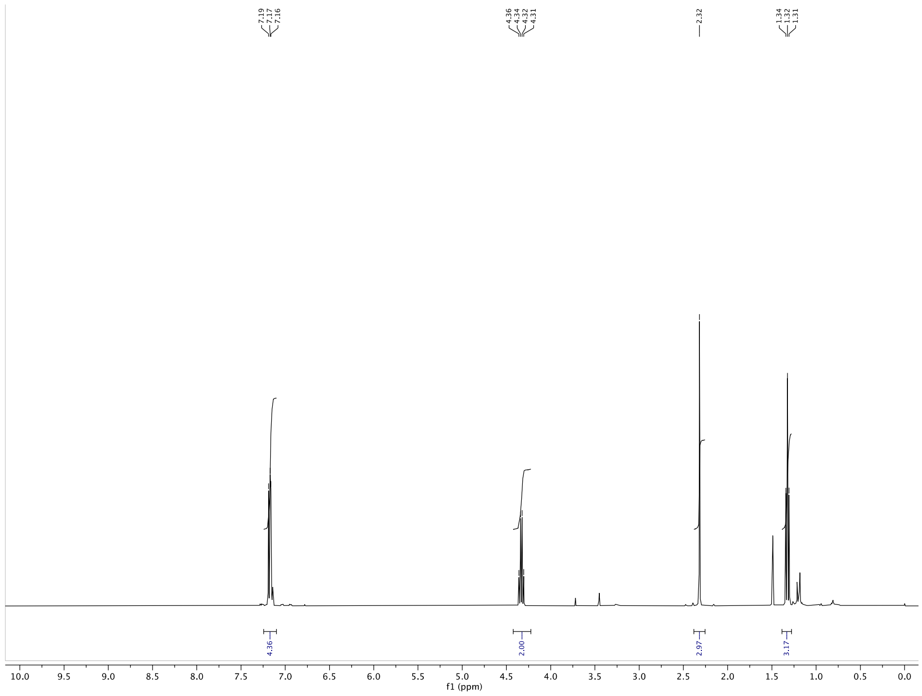

Figure S5. ^{1}H NMR spectrum of ethyl (*E*)-2,4,4,4-tetrafluoro-3-(*p*-tolyl)but-2-enoate (**S71**) in $CDCl_3$.

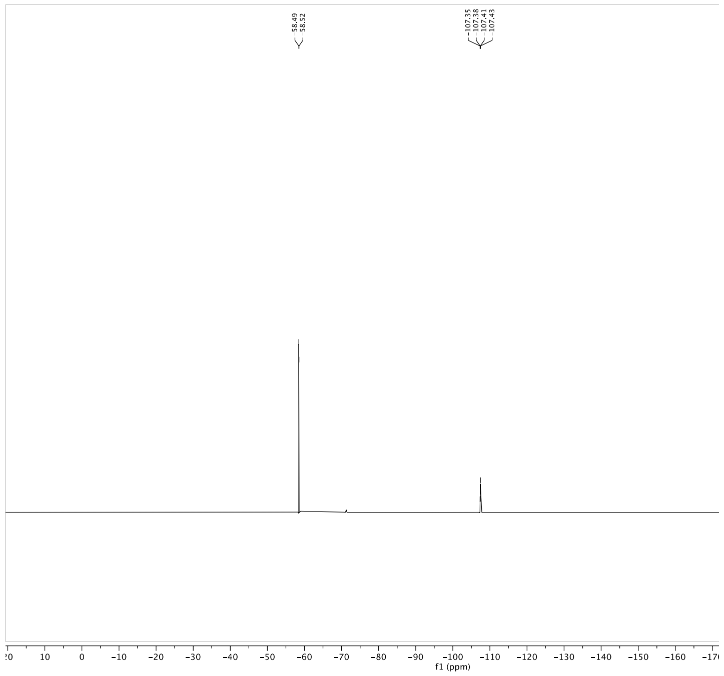

Figure S6. ^{19}F NMR spectrum of ethyl (*E*)-2,4,4,4-tetrafluoro-3-(*p*-tolyl)but-2-enoate (**S71**) in $CDCl_3$.

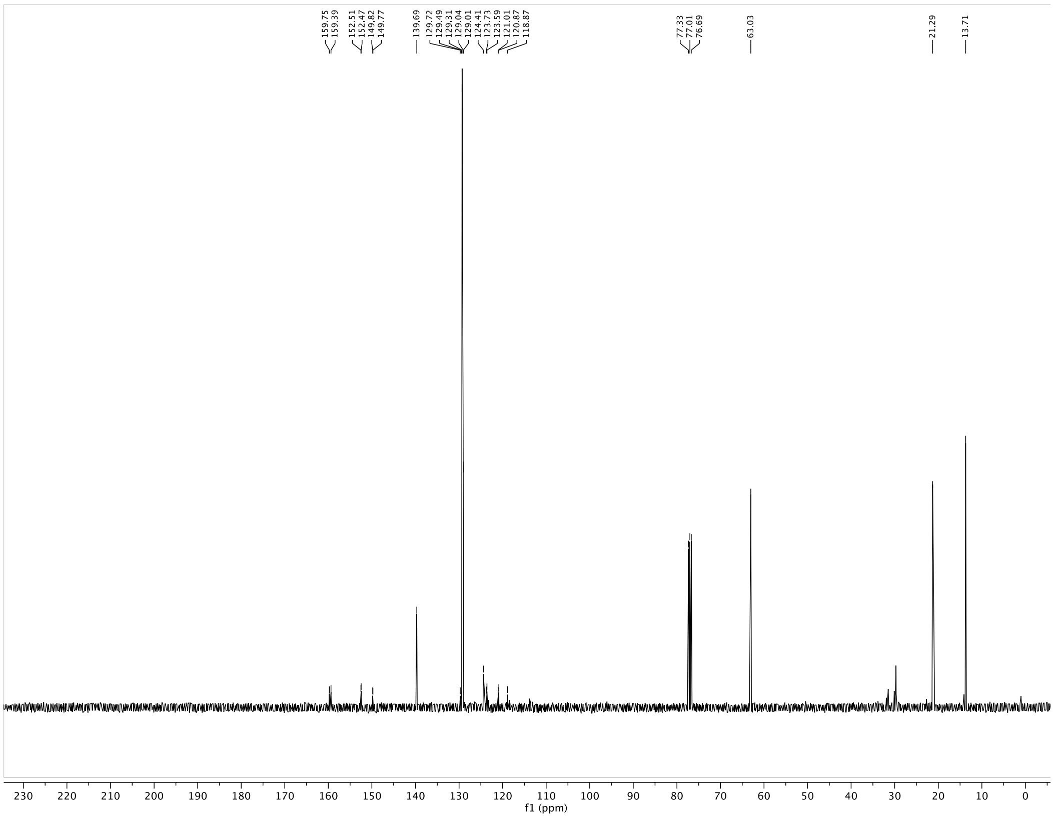

Figure S7. $^{13}C\{^{1}H\}$ NMR spectrum of ethyl (*E*)-2,4,4,4-tetrafluoro-3-(*p*-tolyl)but-2-enoate (**S71**) in $CDCl_3$.

Ethyl (E)-2,4,4,4-tetrafluoro-3-(4-methoxyphenyl)but-2-enoate (S72). Colorless oil. ^{1}H NMR

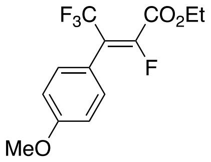

(CDCl3), δ: 1.32 (t, 3H, *J*= 7.2 Hz), 3.77 (s, 3H), 4.33 (q, 2H, *J*= 7.2 Hz), 6.89 (d, 2H, *J*= 8.9 Hz), 7.20 (d, 2H, *J*= 9.0 Hz). ^{19}F NMR (CDCl3), δ: -58.58 (d, *J*= 9.8 Hz), -107.87 (q, *J*= 10.0 Hz). ^{13}C NMR (CDCl3), δ: 13.7, 55.3, 63.0, 114.1, 118.9-119.4 (m), 121.0 (d, *J*= 13.7 Hz), 123.2, 123.7 (d, J= 13.7 Hz), 129.5, 129.7, 130.6 (d, *J*= 2.7 Hz), 149.7 (d, *J*= 4.3 Hz), 152.4 (d, *J*= 4.2 Hz), 159.6 (d, *J*= 36.3 Hz), 160.44.

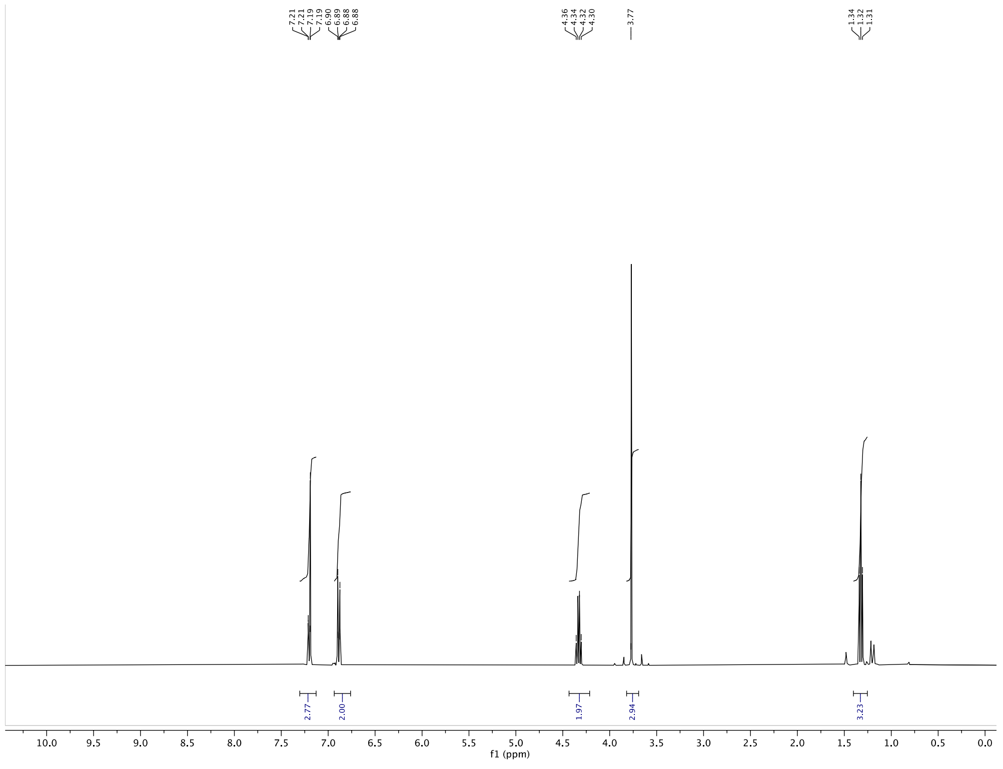

Figure S8. ^{1}H NMR spectrum of ethyl (*E*)-2,4,4,4-tetrafluoro-3-(4-methoxyphenyl)but-2-enoate (**S72**) in CDCl3.

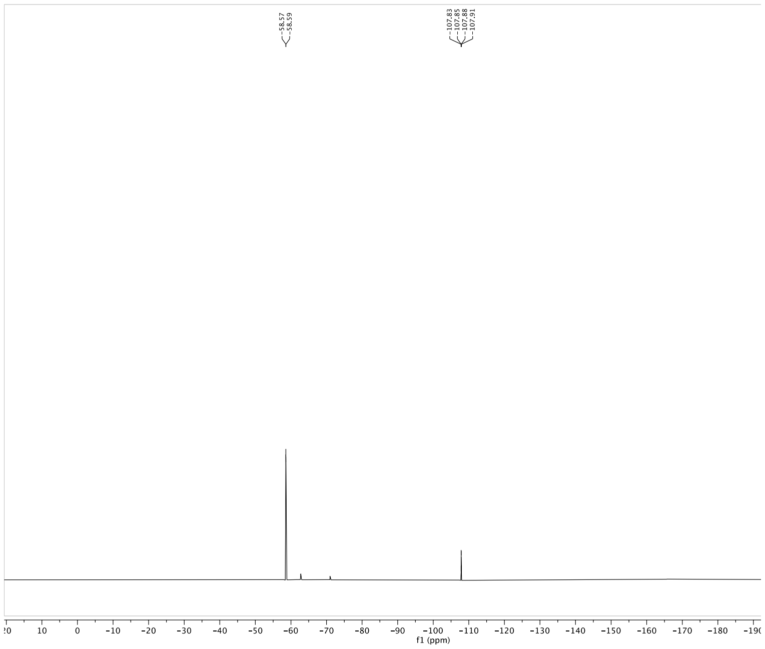

Figure S9. ^{19}F NMR spectrum of ethyl (*E*)-2,4,4,4-tetrafluoro-3-(4-methoxyphenyl)but-2-enoate (**S72**) in $CDCl_3$.

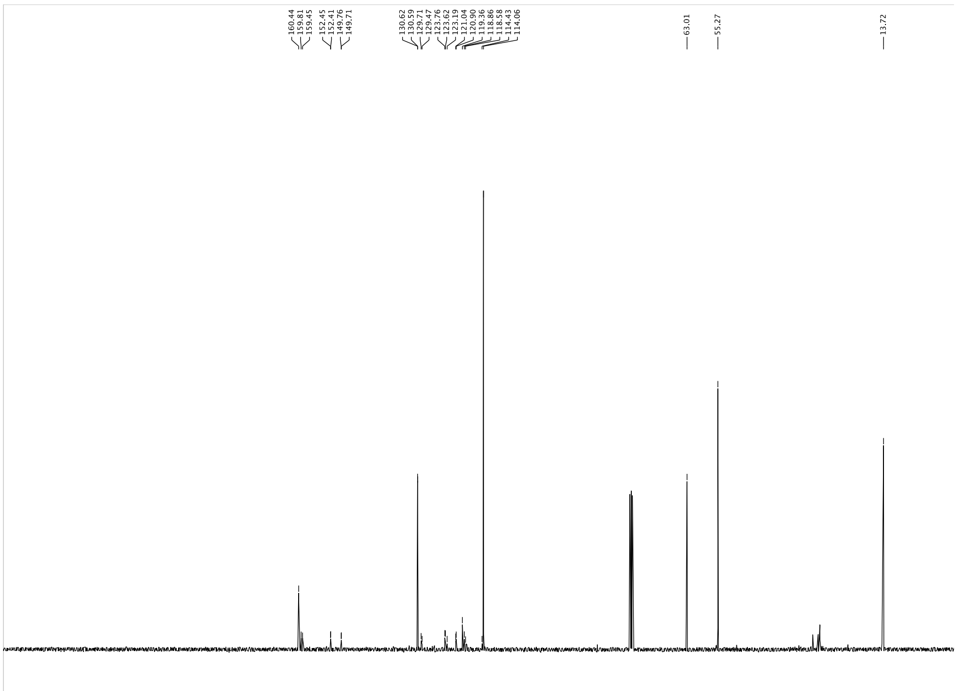

Figure S10. $^{13}C\{^1H\}$ NMR spectrum of ethyl (*E*)-2,4,4,4-tetrafluoro-3-(4-methoxyphenyl)but-2-enoate (**S72**) in $CDCl_3$.

Ethyl (E)-3-(4-bromophenyl)-2,4,4,4-tetrafluorobut-2-enoate (S73). Colorless oil. ^{1}H NMR ($CDCl_3$), δ: 1.40 (t, 3H, *J*= 7.1 Hz), 4.41 (q, 2H, *J*= 7.1 Hz), 7.21 (dt, 2H, *J*= 8.9 Hz, *J*= 0.6 Hz), 7.57-7.60 (m, 2H). ^{19}F NMR ($CDCl_3$), δ: -58.44 (d, *J*= 9.8 Hz), -105.68 (q, *J*= 10.2 Hz). ^{13}C NMR ($CDCl_3$), δ: 13.8, 63.4, 117.7-118.2 (m), 120.8 (d, *J*= 14.0 Hz), 123.5 (d, *J*= 14.0 Hz), 124.3, 126.4, 130.9 (d, *J*= 2.7 Hz), 132.1, 150.3 (d, *J*= 4.2 Hz), 153.0 (d, *J*= 4.3 Hz), 159.3 (d, *J*= 36.0 Hz).

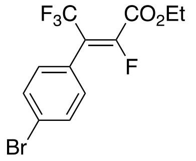

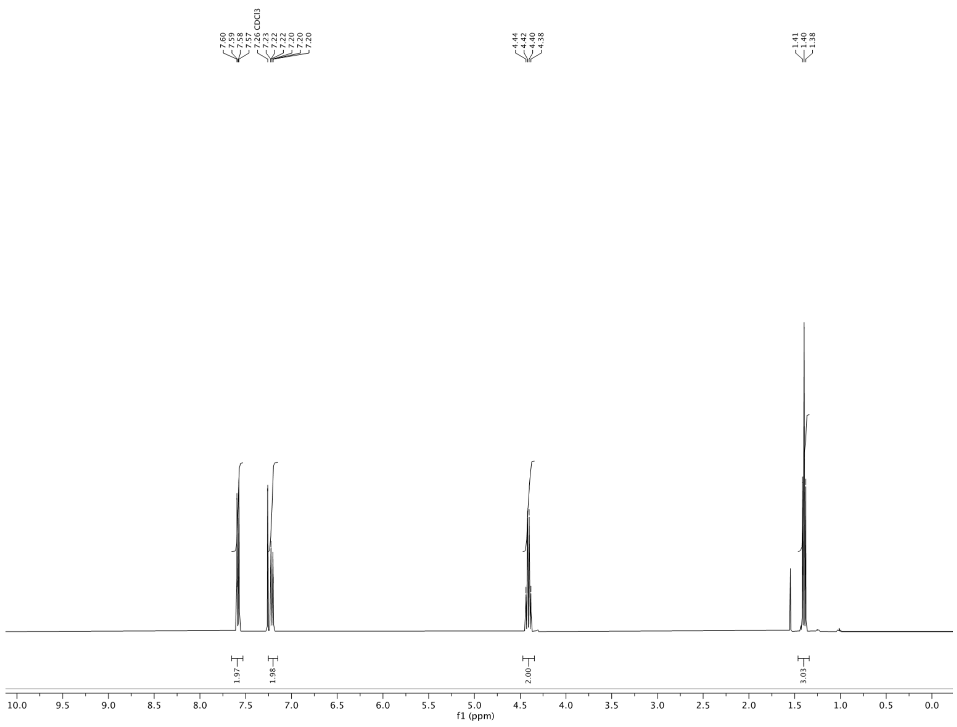

Figure S11. ^{1}H NMR spectrum of ethyl (*E*)-3-(4-bromophenyl)-2,4,4,4-tetrafluorobut-2-enoate (**S73**) in $CDCl_3$.

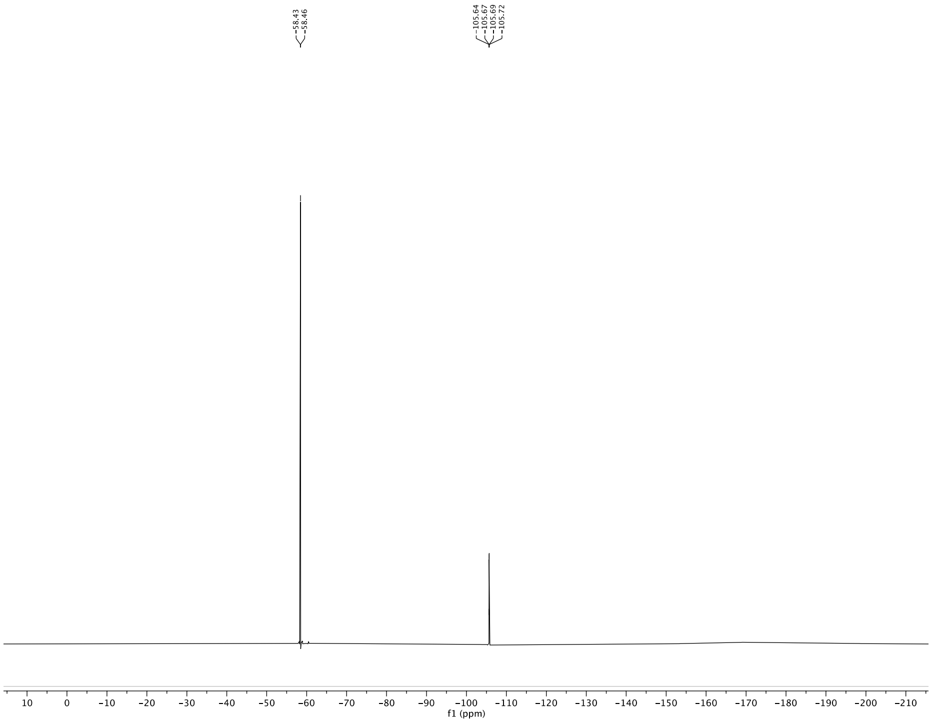

Figure S12. ^{19}F NMR spectrum of ethyl (*E*)-3-(4-bromophenyl)-2,4,4,4-tetrafluorobut-2-enoate (**S73**) in $CDCl_3$.

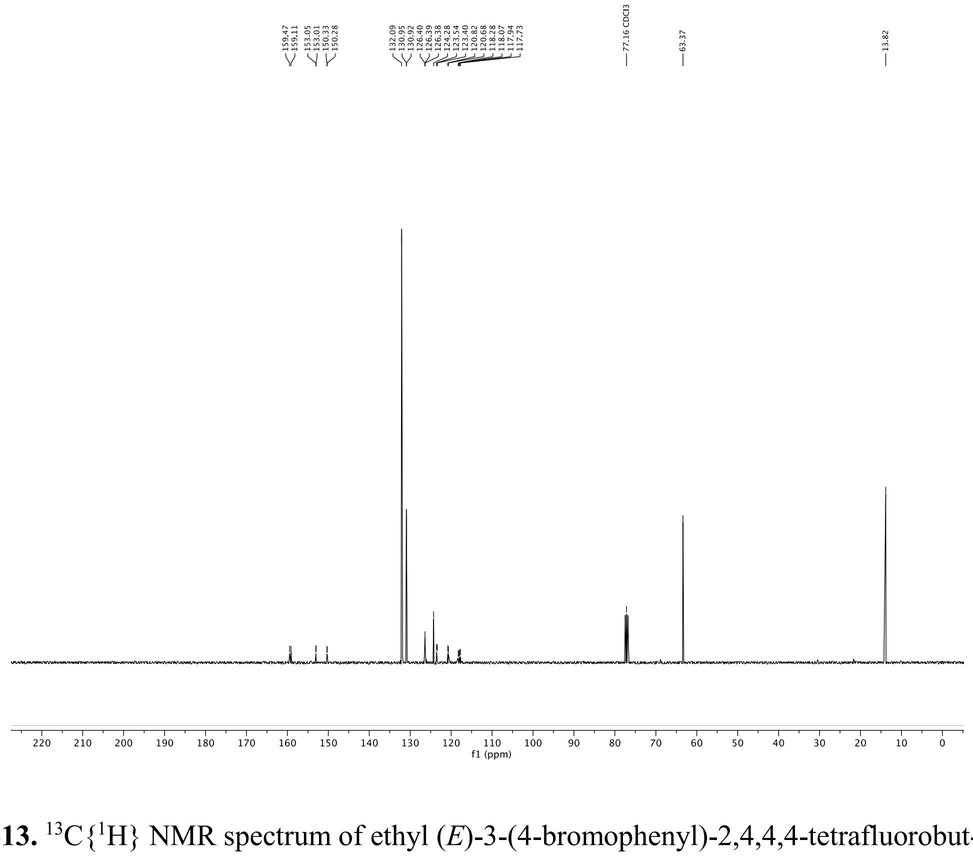

Figure S13. ^{13}C{^{1}H} NMR spectrum of ethyl (*E*)-3-(4-bromophenyl)-2,4,4,4-tetrafluorobut-2-enoate (**S73**) in $CDCl_3$.

9. Characterization details and methods for ee determination of hydrogenated products

(3,3-Dimethylbutan-2-yl)benzene (from S1).[40] Enantiomeric excess determined by GC using Chiradex

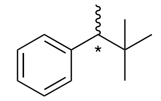

B-DM column (100 kPa H_2, 60 °C for 30 min, 3 °C/min until 175 °C). t_R 30.2 min (*S*); t_R 31.6 min (*R*). ^1H NMR ($CDCl_3$), δ: 0.87 (s, 9H), 1.26 (d, 3H, *J*= 7.2 Hz), 2.56 (q, 1H, *J*= 7.2 Hz), 7.15-7.20 (m, 3H), 7.24-7.28 (m, 2H).

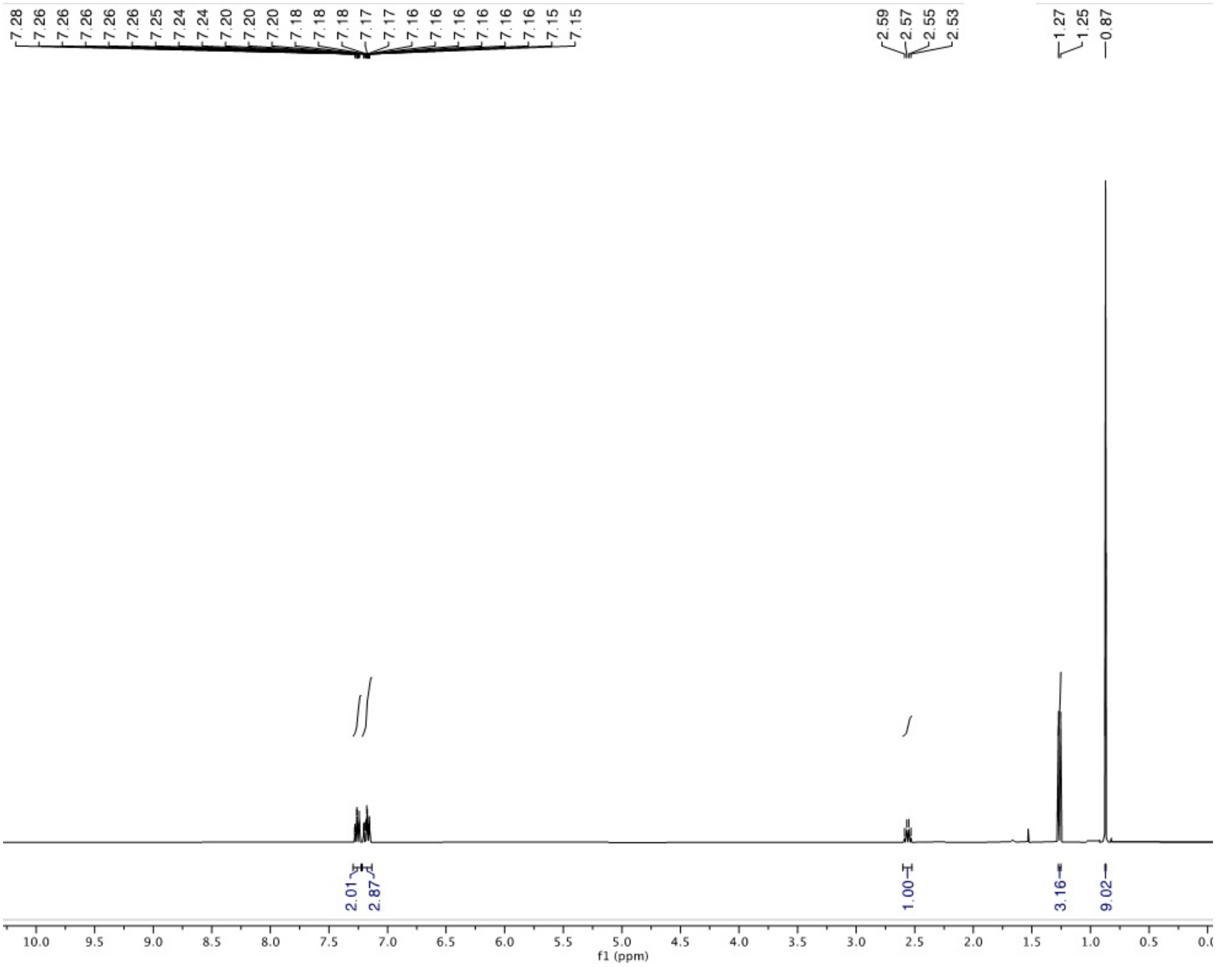

Figure S14. ^1H NMR spectrum of (3,3-dimethylbutan-2-yl)benzene (from **S1**) in $CDCl_3$.

(a)

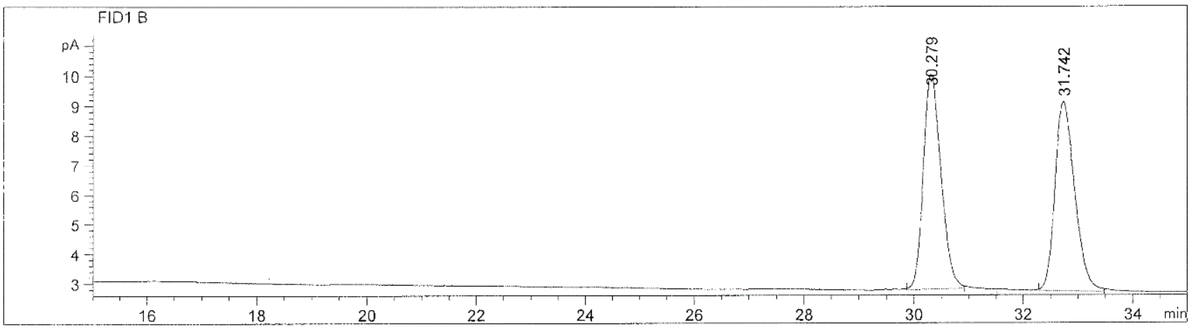

(b)

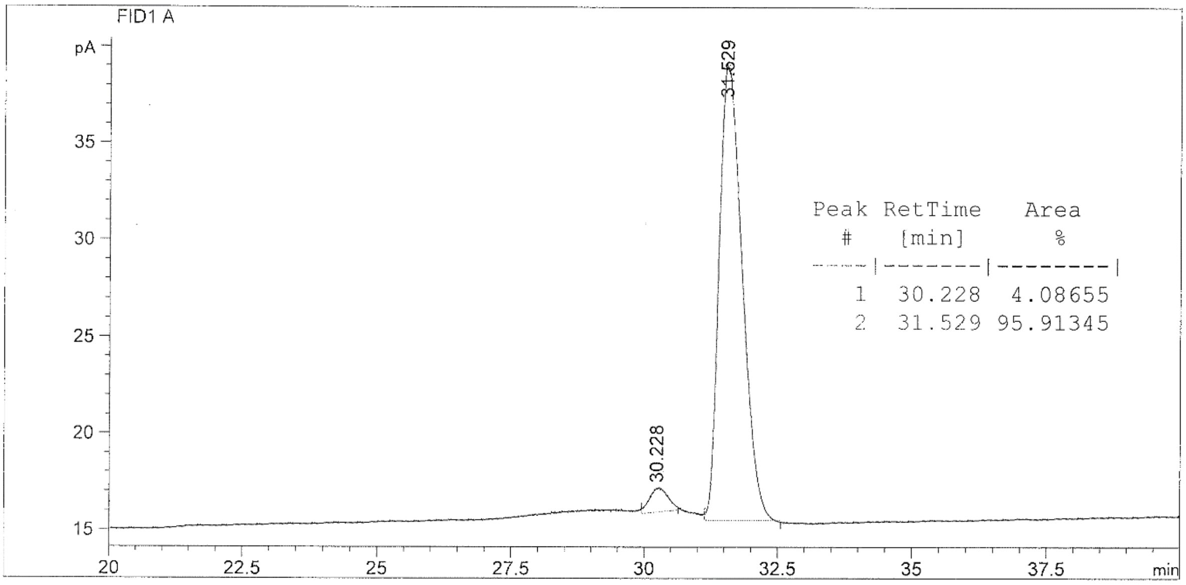

Figure S15. GC trace of: (a) racemic and (b) enantioenriched product (91% (*R*) ee using **4c** catalytic system; Table 1, entry 7).

1-(sec-Butyl)-4-methoxybenzene (from S2, S12 and S15).[40] Enantiomeric excess determined by GC using Chiradex B-DM column (100 kPa H_2, 60 °C for 30 min, 3 °C/min until 175 °C). t_R 47.1 min (*S*); t_R 47.4 min (*R*). ^{1}H NMR ($CDCl_3$), δ: 0.81 (t, 3H, *J*= 7.4 Hz), 1.21 (d, 3H, *J*= 7.0 Hz), 1.55 (m, 2H), 2.56 (m, 1H), 3.79 (s, 3H), 6.83-6.85 (m, 2H), 7.09-7.11 (m, 2H).

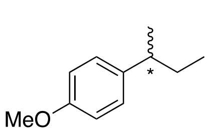

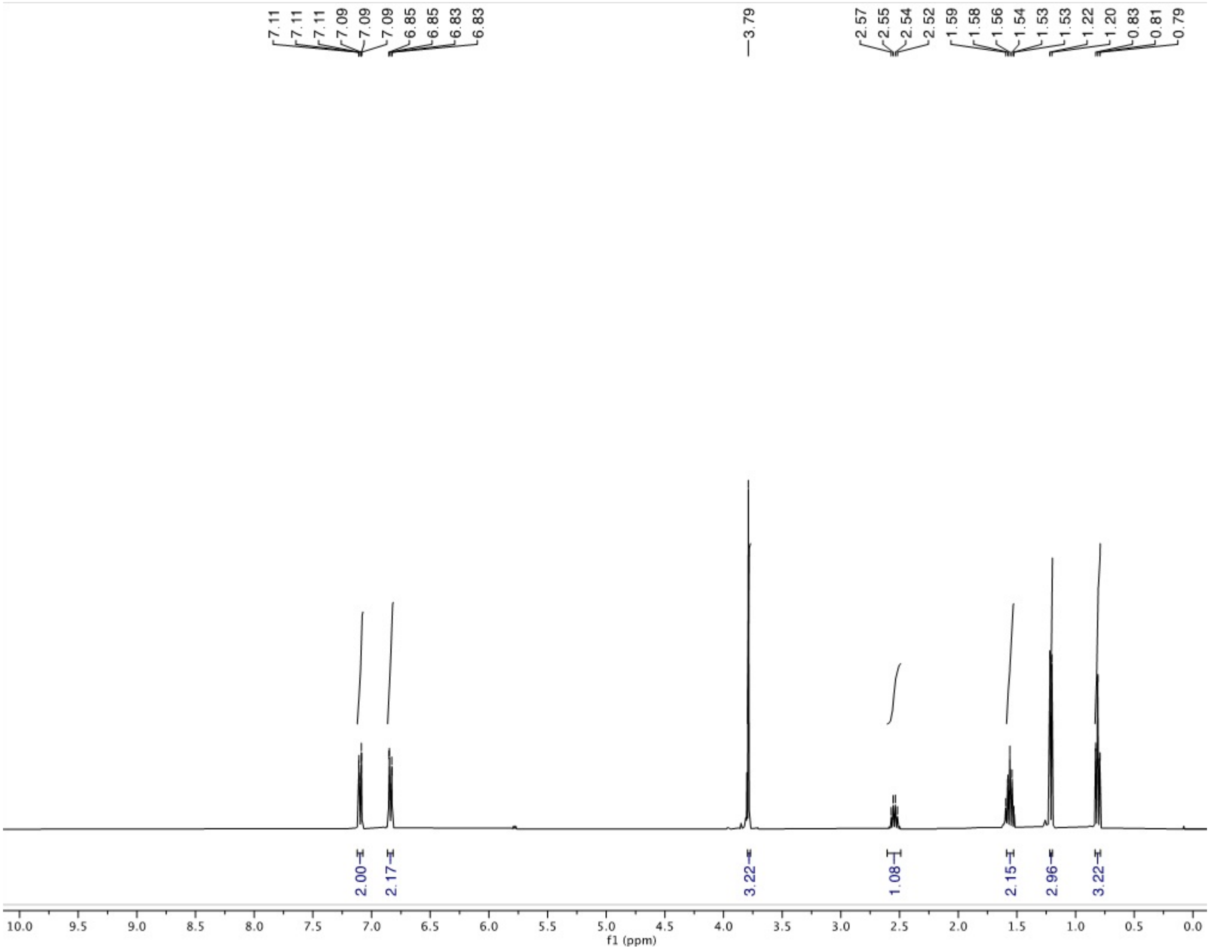

Figure S16. ^{1}H NMR 1-(*sec*-butyl)-4-methoxybenzene (from **S2**, **S12** and **S15**) in $CDCl_3$.

(a)

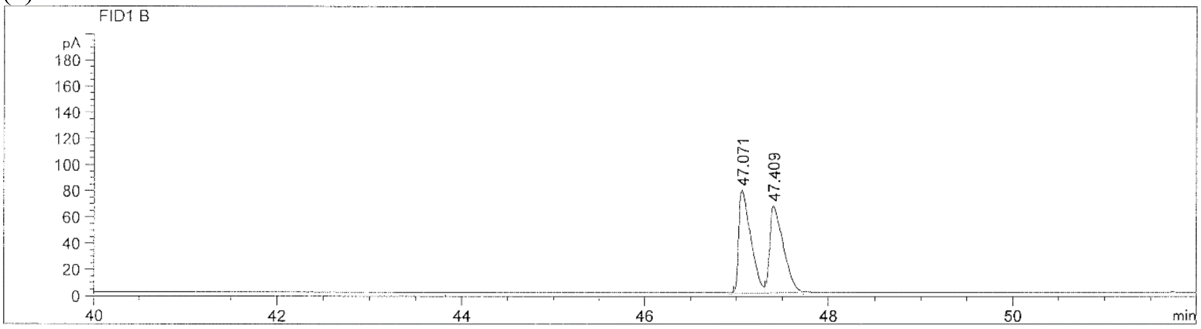

(b)

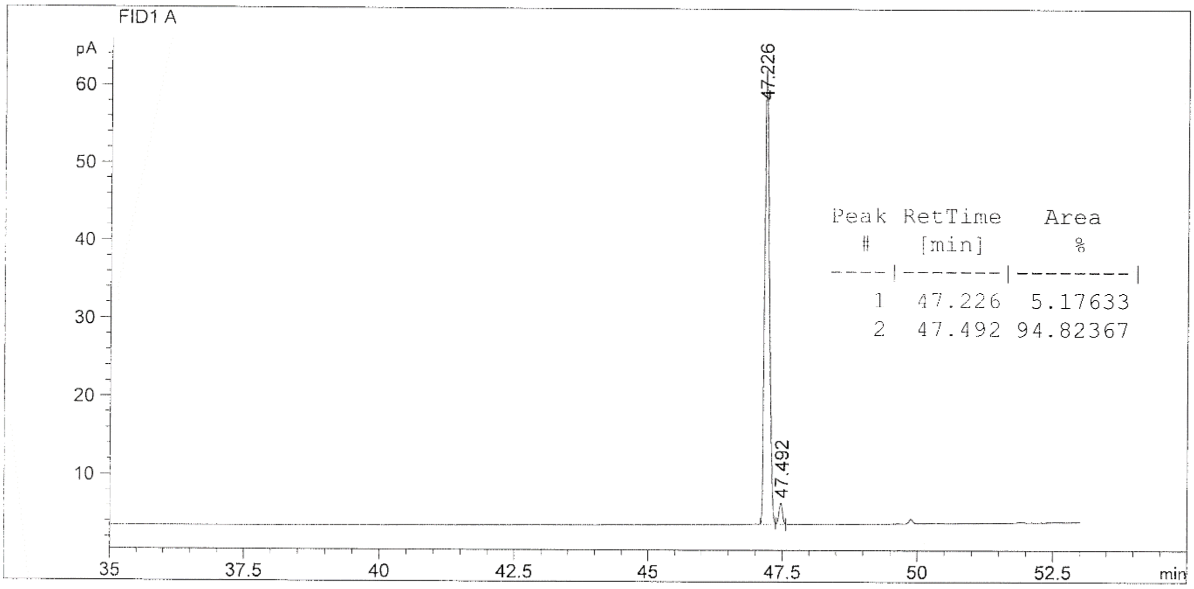

(c)

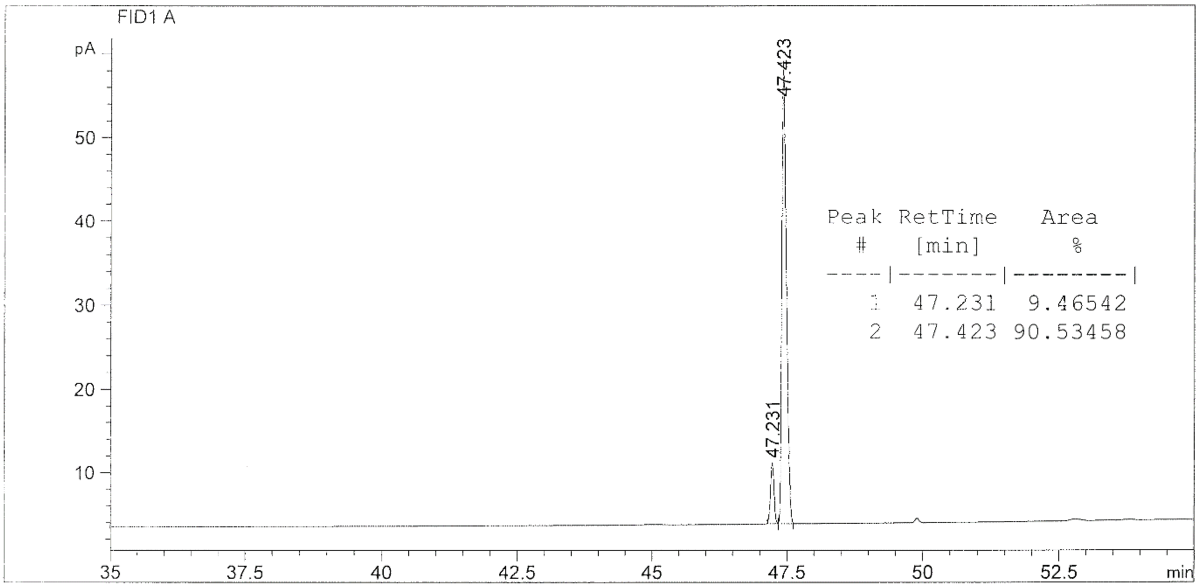

Figure S17. GC trace of (a) racemic; (b) enantioenriched product (89% (*S*) ee using **4c** catalytic system; Table 1, entry 9) and (c) enantioenriched product (80% (*R*) ee using **4c** catalytic system; Table 3, entry 8).

1-(3,3-Dimethylbutan-2-yl)-4-methoxybenzene (from S5).[14] Enantiomeric excess determined by GC

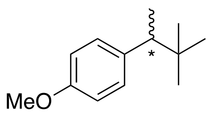

using Chiradex B-DM column (100 kPa H_2, 60 °C for 30 min, 3 °C/min until 175 °C). t_R 51.5 min (*S*); t_R 51.9 min (*R*). ^{1}H NMR ($CDCl_3$), δ: 0.85 (s, 9H), 1.23 (d, 3H, *J*= 7.2 Hz), 2.51 (q, 1H, *J*= 7.2 Hz), 3.79 (s, 3H), 6.81 (d, 2H, *J*= 8.7 Hz), 7.08 (d, 2H, *J*= 8.6 Hz).

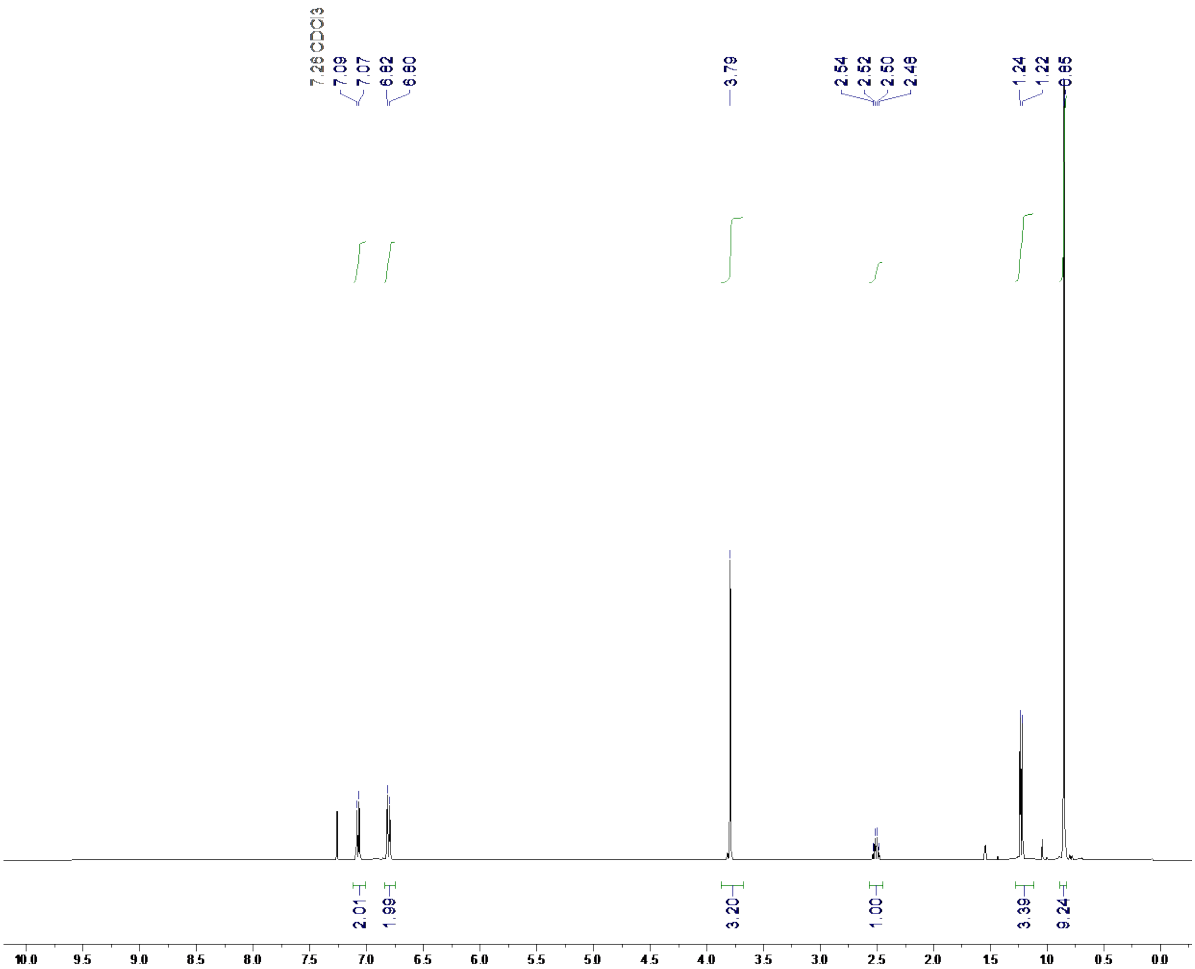

Figure S18. ^{1}H NMR spectrum of 1-(3,3-dimethylbutan-2-yl)-4-methoxybenzene (from **S5**) in $CDCl_3$.

(a)

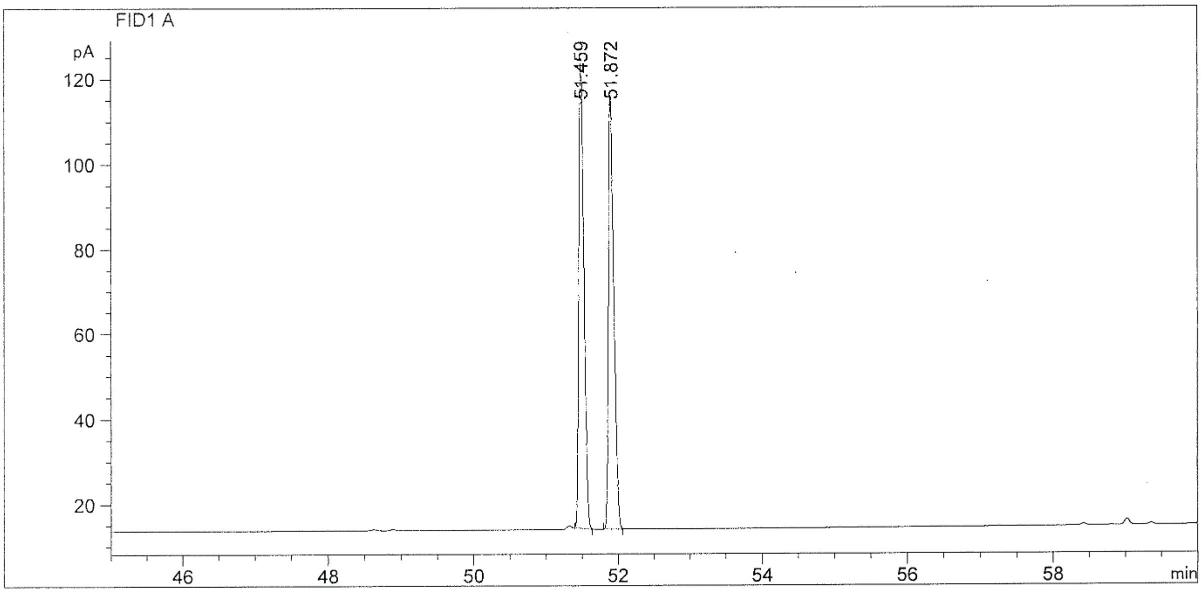

(b)

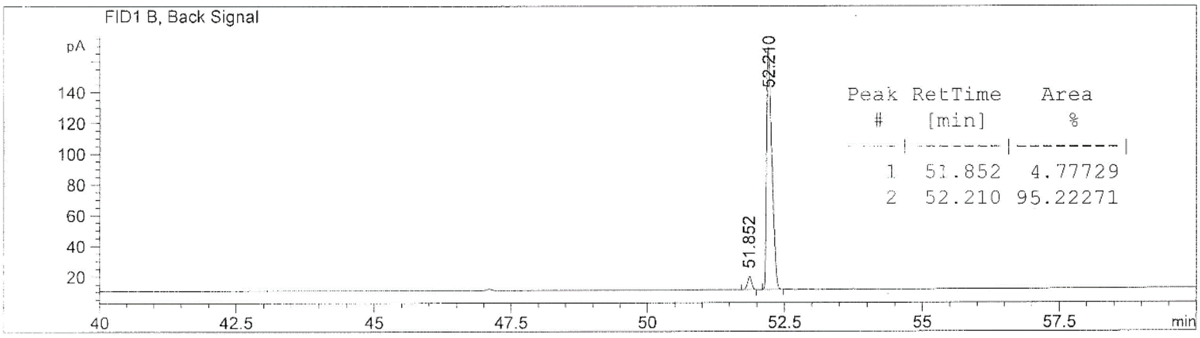

Figure S19. GC trace of (a) racemic and (b) enantioenriched product (90% (*R*) ee using **4c** catalytic system; Table 3, entry 1).

1-(3,3-Dimethylbutan-2-yl)-4-(trifluoromethyl)benzene (from S6).[14] Enantiomeric excess determined by GC using Chiradex B-DM column (100 kPa H_2, 60 °C for 30 min, 3 °C/min until 175 °C). t_R 39.5 min (*S*); t_R 40.0 min (*R*). ^{1}H NMR ($CDCl_3$), δ: 0.88 (s, 9H), 1.25 (d, 3H, *J*= 8.0 Hz), 2.63 (q, 1H, *J*= 7.2 Hz), 7.26 (d, 2H, *J*= 6.7 Hz), 7.51 (d, 2H, *J*= 8.0 Hz).

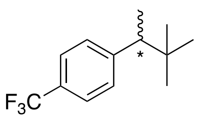

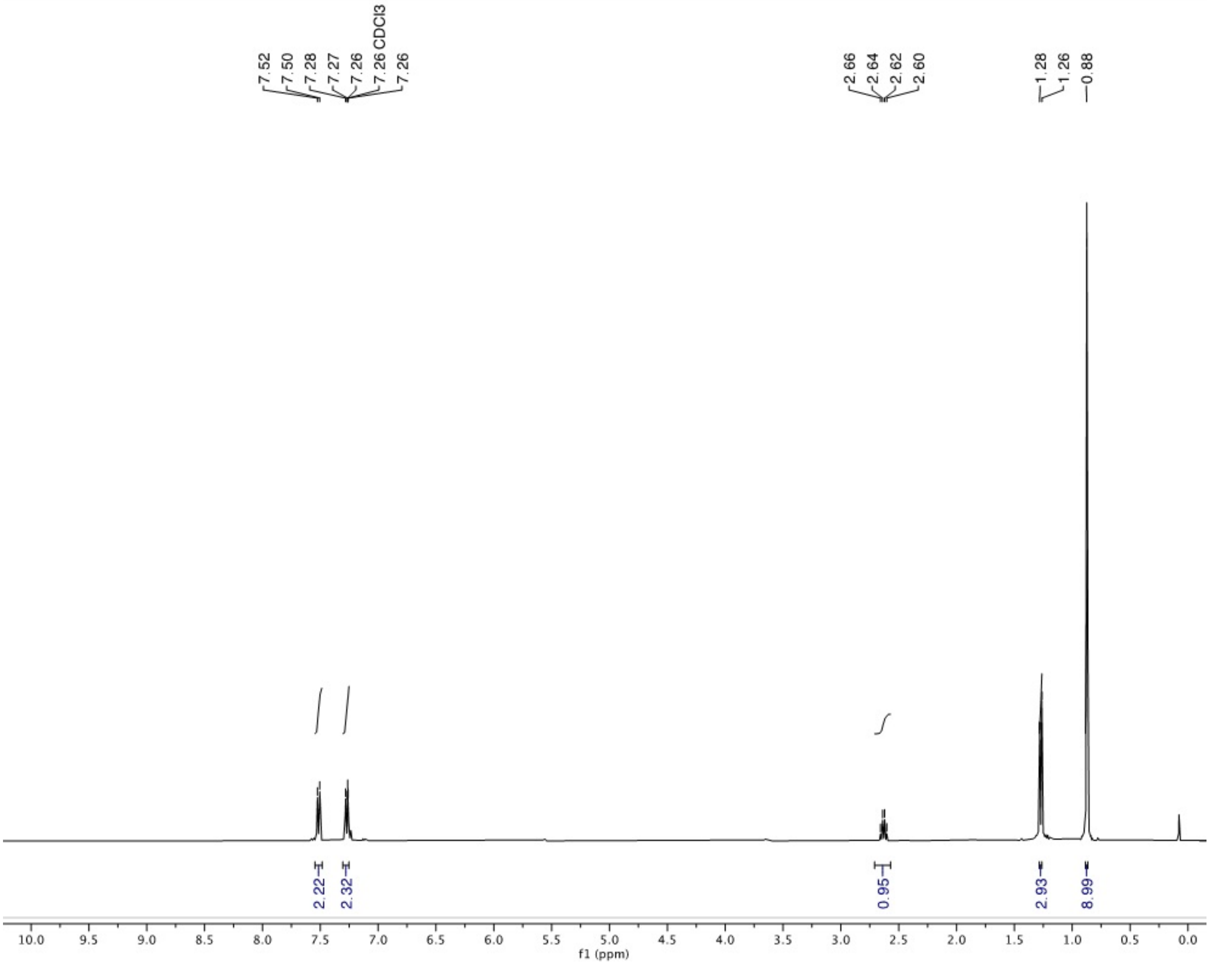

Figure S20. ^{1}H NMR spectrum of 1-(3,3-dimethylbutan-2-yl)-4-(trifluoromethyl)benzene (from **S6**) in $CDCl_3$.

(a)

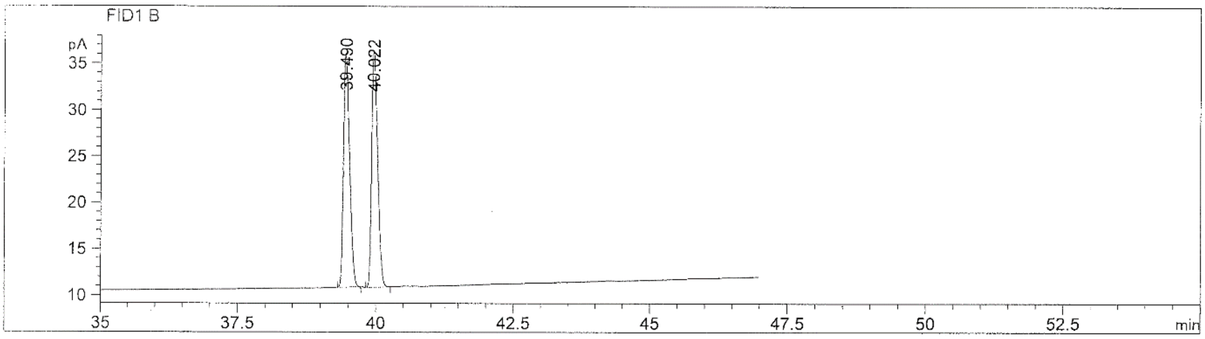

(b)

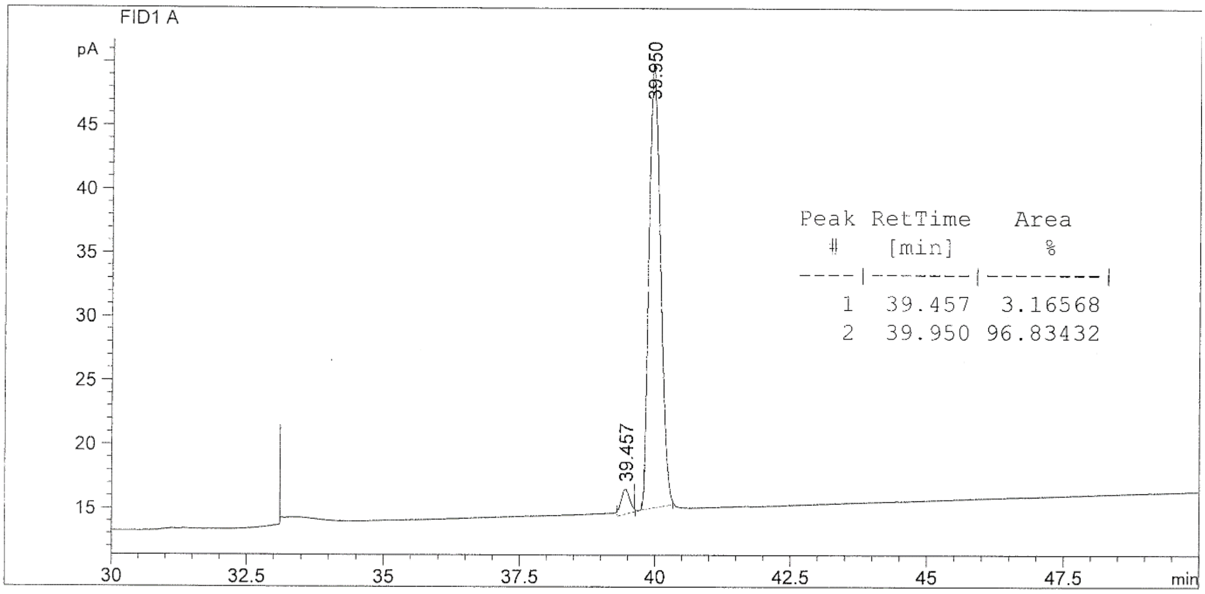

Figure S21. GC trace of (a) racemic and (b) enantioenriched product (94% (*R*) ee using **4c** catalytic system; Table 3, entry 2).

1-(3,3-Dimethylbutan-2-yl)-4-methylbenzene (from S7).[14] Enantiomeric excess determined by GC using Chiradex B-DM column (100 kPa H_2, 60 °C for 30 min, 3 °C/min until 175 °C). t_R 41.9 min (*S*); t_R 42.4 min (*R*). ^{1}H NMR ($CDCl_3$), δ: 0.86 (s, 9H), 1.24 (d, 3H, *J*= 7.2 Hz), 2.33 (s, 3H), 2.53 (q, 1H, *J*= 7.2 Hz), 7.06-7.07 (m, 4H).

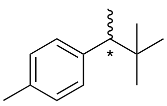

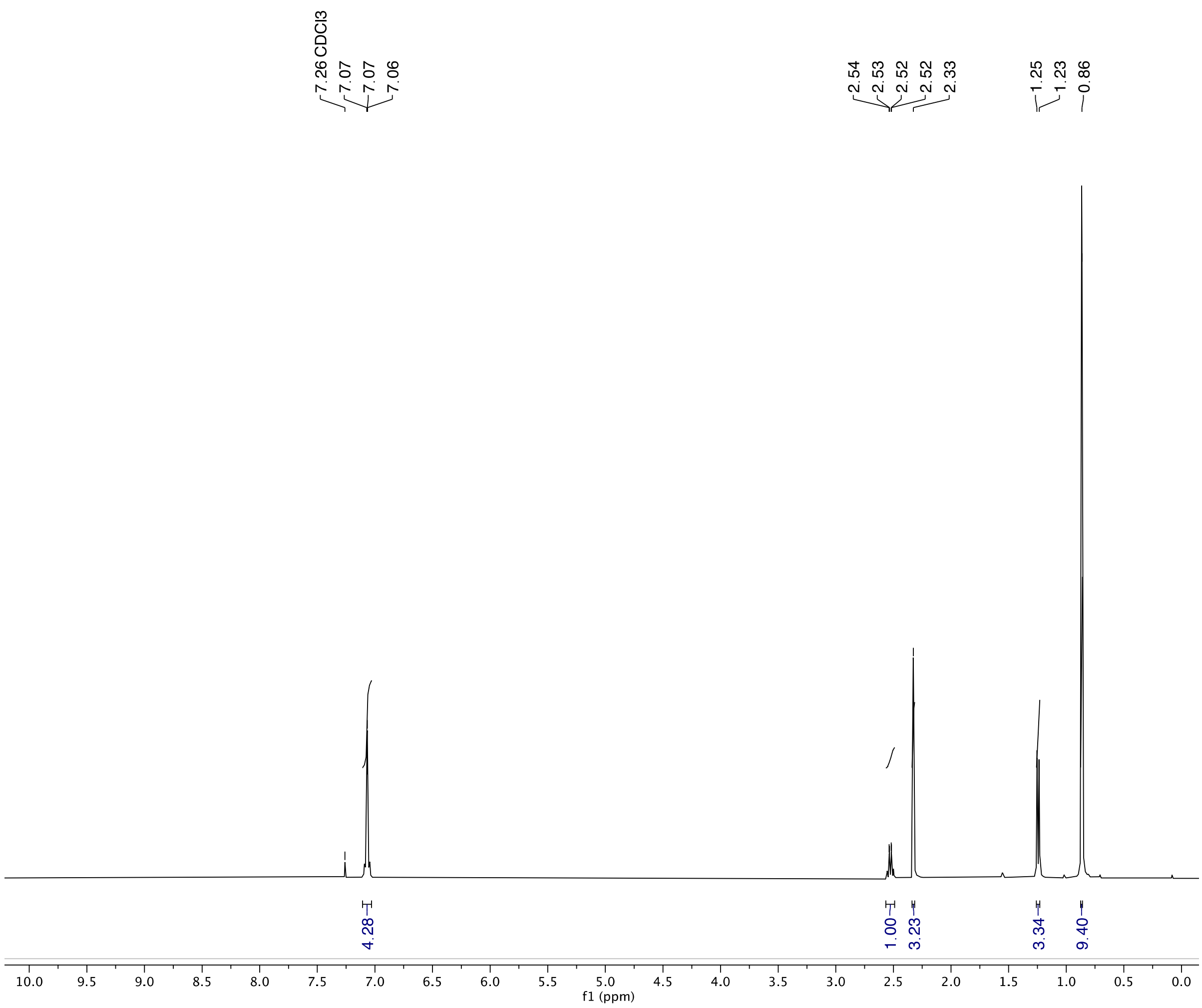

Figure S22. ^{1}H NMR spectrum of 1-(3,3-dimethylbutan-2-yl)-4-methylbenzene (from **S7**) in $CDCl_3$.

(a)

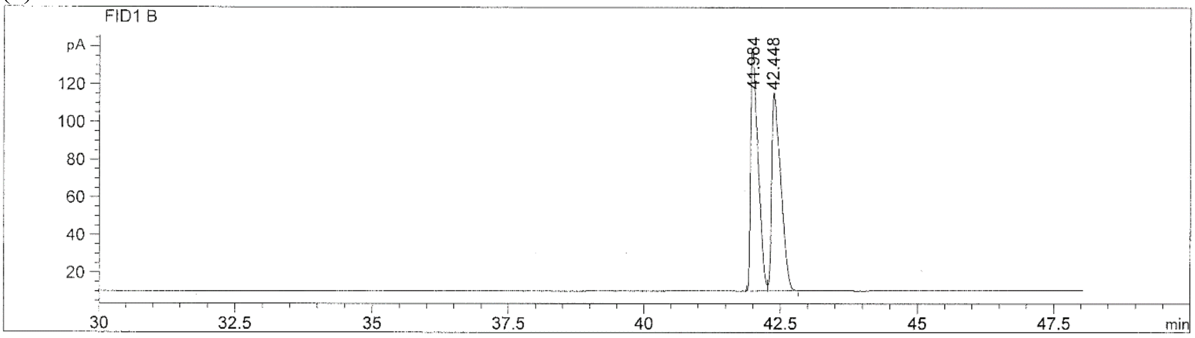

(b)

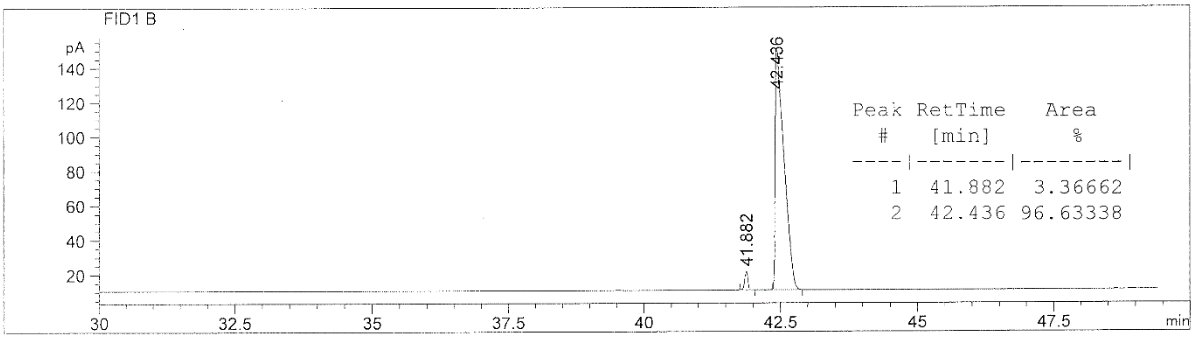

Figure S23. GC trace of (a) racemic and (b) enantioenriched product (92% (*R*) ee using **4c** catalytic system; Table 3, entry 3).

1-(3,3-Dimethylbutan-2-yl)-3-methylbenzene (from S8).[14] Enantiomeric excess determined by GC using Chiradex B-DM column (100 kPa H_2, 60 °C for 30 min, 3 °C/min until 175 °C). t_R 41.4 min (*S*); t_R 42.3 min (*R*). ^{1}H NMR ($CDCl_3$), δ: 0.86 (s, 9H), 1.24 (d, 3H, *J*= 7.2 Hz), 2.33 (s, 3H), 2.51 (q, 1H, *J*= 7.2 Hz), 6.95-7.01 (m, 3H), 7.12-7.16 (m, 1H).

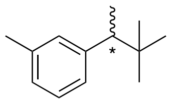

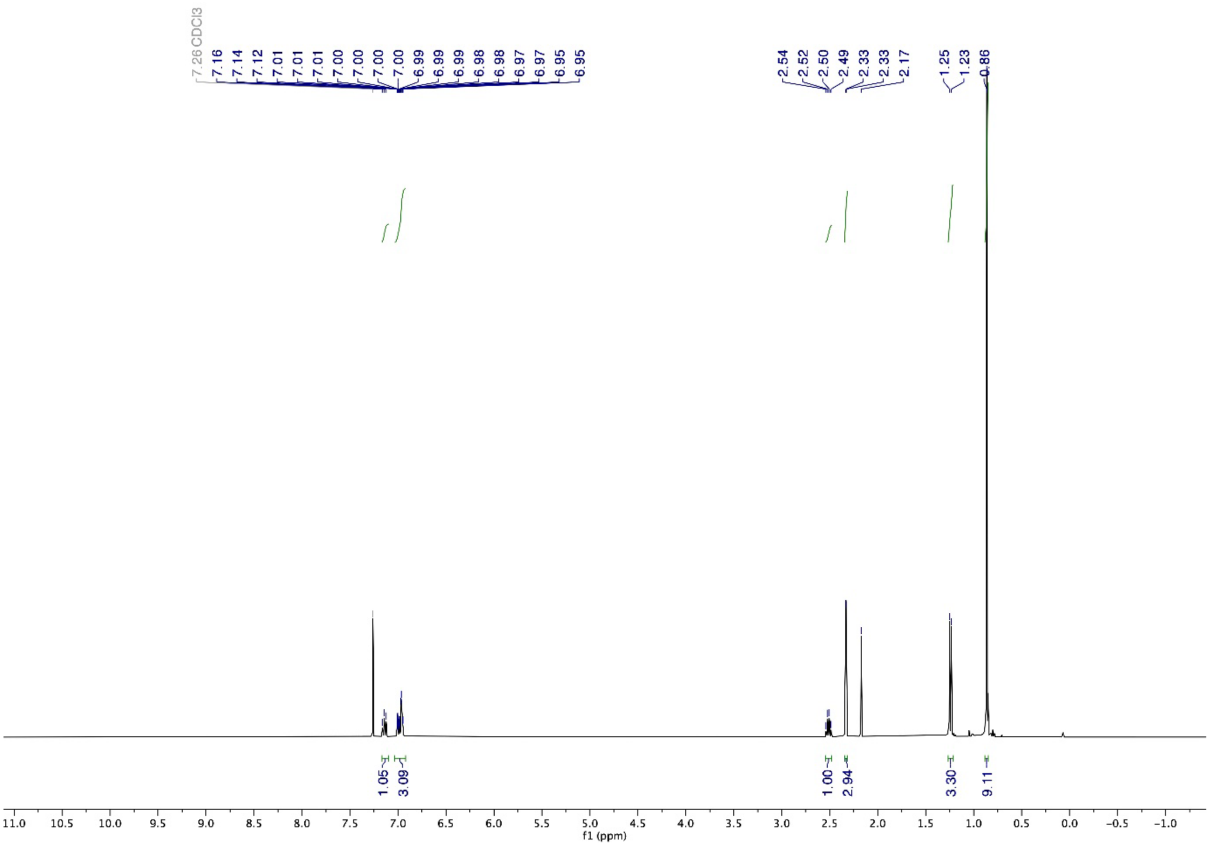

Figure S24. ^{1}H NMR spectrum of 1-(3,3-dimethylbutan-2-yl)-3-methylbenzene (from **S8**) in $CDCl_3$.

(a)

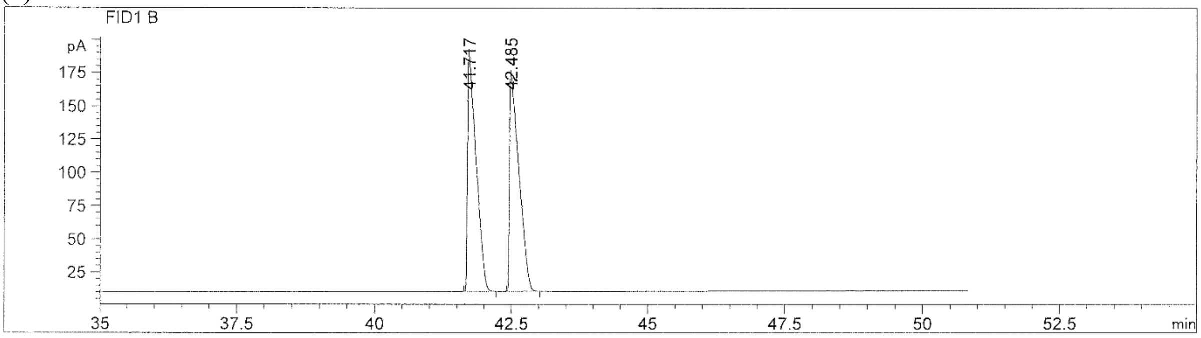

(b)

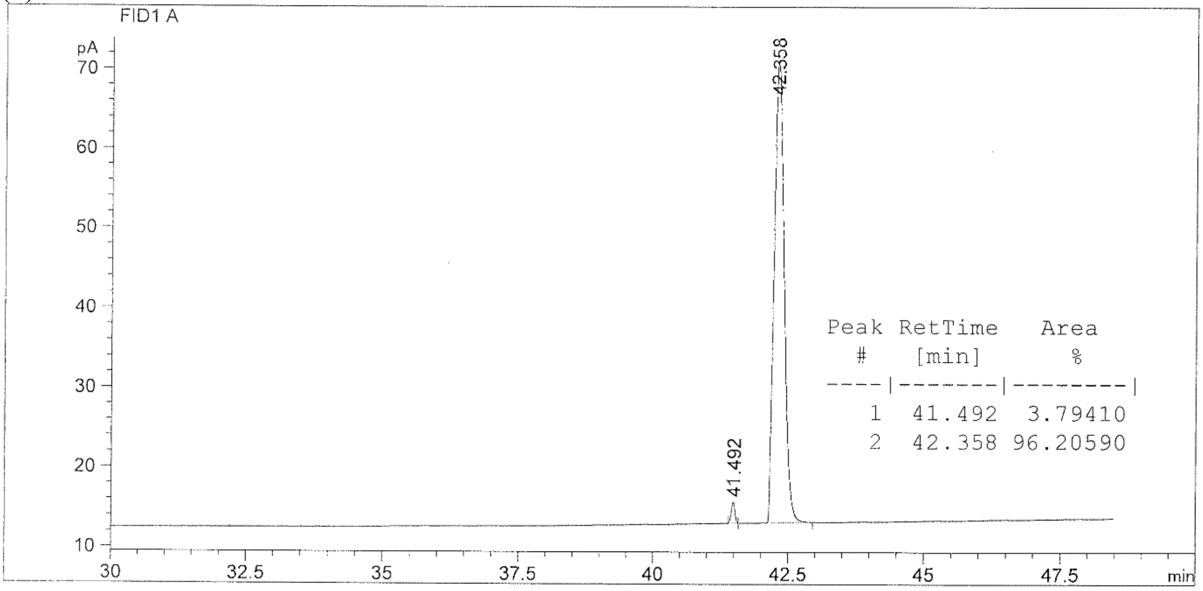

Figure S25. GC trace of (a) racemic and (b) enantioenriched product (92% (*R*) ee using **4c** catalytic system; Table 3, entry 4).

2-(3,3-Dimethylbutan-2-yl)naphthalene (from S9).[14] Enantiomeric excess determined by GC using

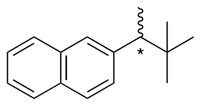

Chiradex B-DM column (100 kPa H_2, 60 °C for 30 min, 3 °C/min until 175 °C). t_R 63.5 min (*S*); t_R 63.7 min (*R*). ^{1}H NMR ($CDCl_3$), δ: 0.94 (s, 9H), 1.37 (d, 3H, *J*= 7.2 Hz), 2.74 (q, 1H, *J*= 7.2 Hz), 7.35-7.37 (m, 1H), 7.41-7.48 (m, 2H), 7.61-7.62 (m, 1H), 7.74-7.76 (m, 1H), 7.80-7.83 (m, 2H).

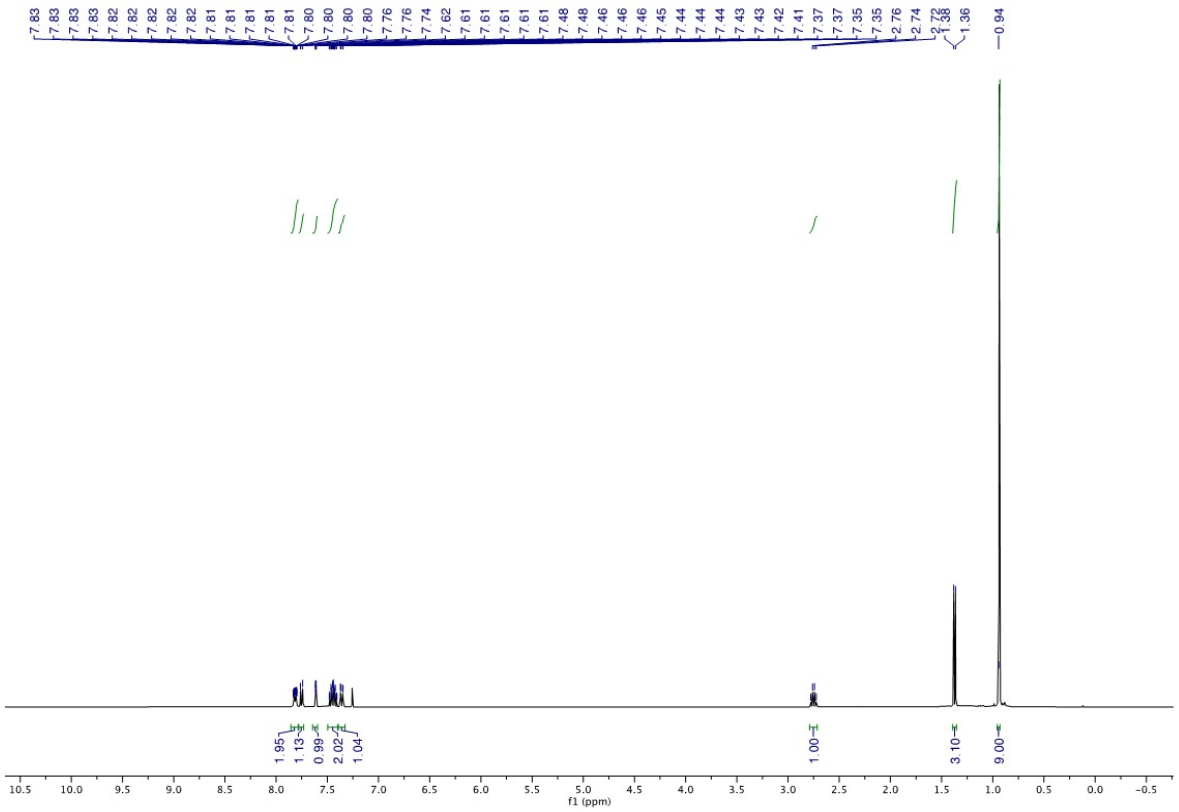

Figure S26. ^{1}H NMR spectrum of 2-(3,3-dimethylbutan-2-yl)naphthalene (from **S9**) in $CDCl_3$.

(a)

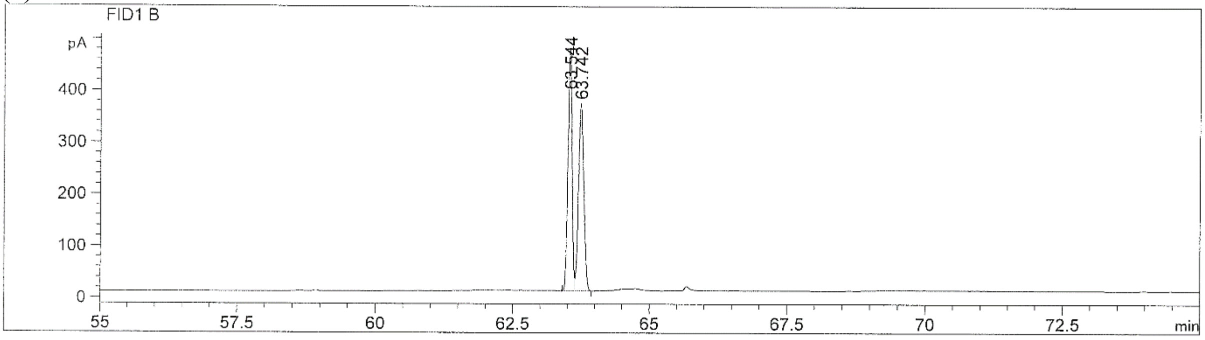

(b)

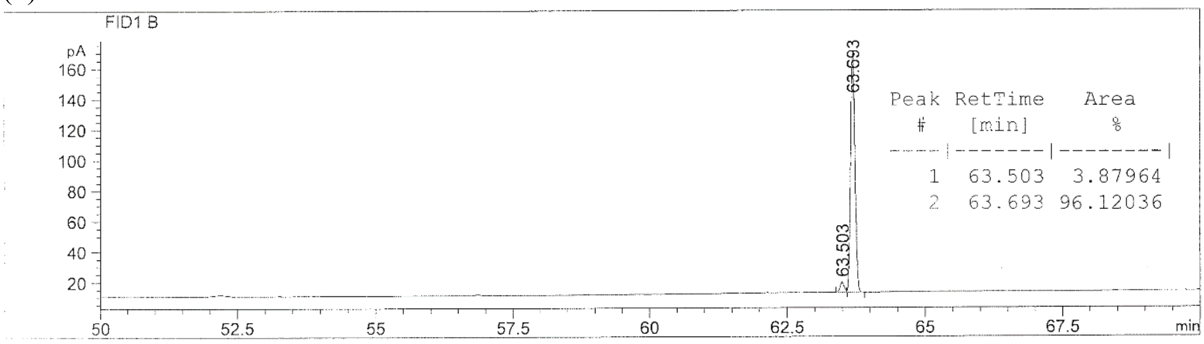

Figure S27. GC trace of: (a) racemic and (b) enantioenriched product (92% (*R*) ee using **4c** catalytic system; Table 3, entry 5).

1-(3,3-Dimethylbutan-2-yl)-2-methylbenzene (from S10).[14] Enantiomeric excess determined by GC

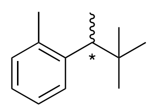

using Chiradex B-DM column (100 kPa H_2, 60 °C for 30 min, 3 °C/min until 175 °C). t_R 39.7 min (*S*); t_R 40.3 min (*R*). ^{1}H NMR ($CDCl_3$), δ: 0.91 (s, 9H), 1.21 (d, 3H, *J*= 7.2 Hz), 2.35 (s, 3H), 2.94 (q, 1H, *J*= 7.2 Hz), 7-07-7.22 (m, 4H).

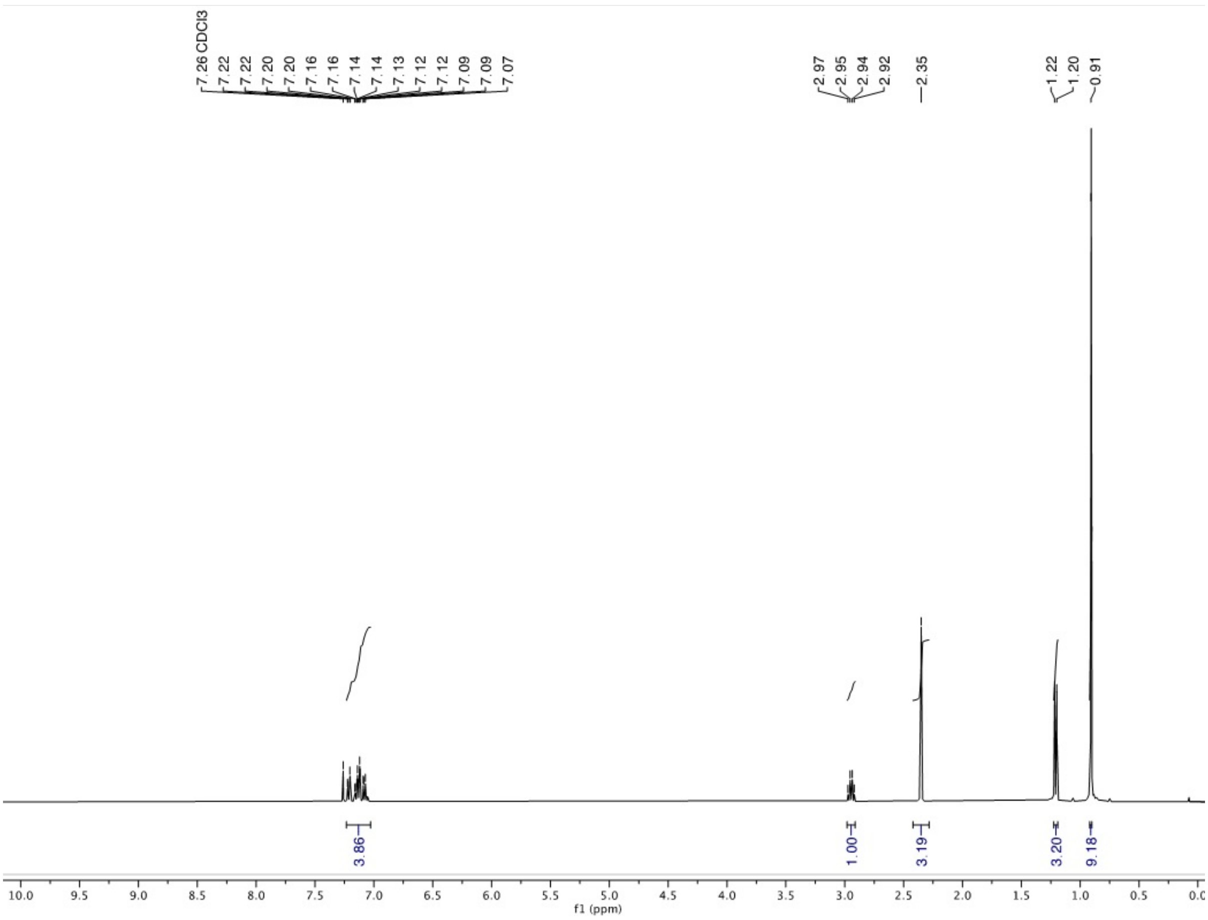

Figure S28. ^{1}H NMR spectrum of 1-(3,3-dimethylbutan-2-yl)-2-methylbenzene (from **S10**) in $CDCl_3$.

(a)

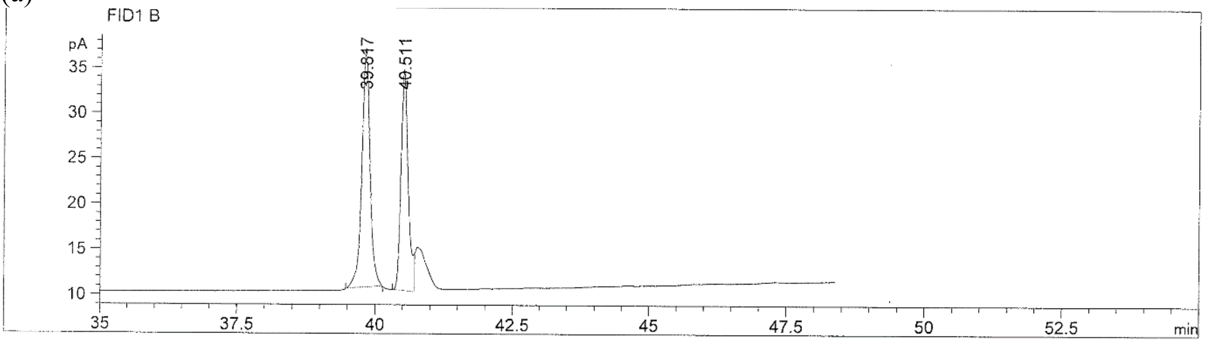

(b)

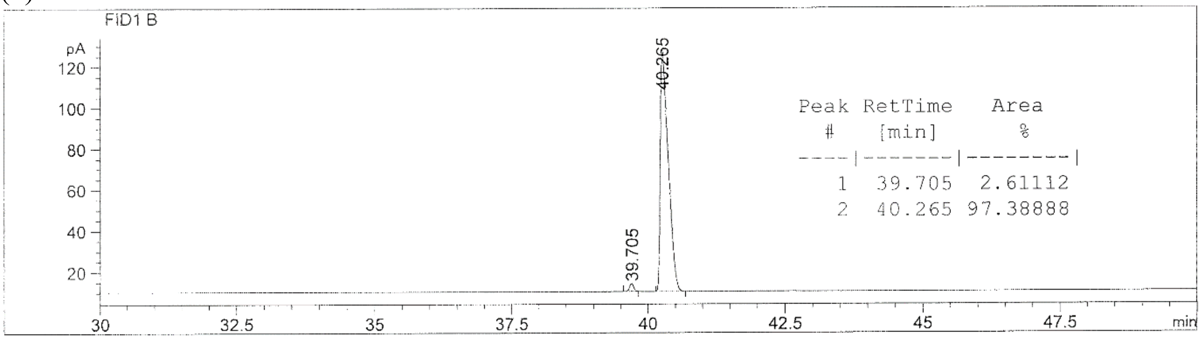

Figure S29. GC trace of (a) racemic and (b) enantioenriched product (94% (*R*) ee using **4c** catalytic system; Table 3, entry 6).

1-(3,3-Dimethylbutan-2-yl)naphthalene (from S11).[14] Enantiomeric excess determined by GC using

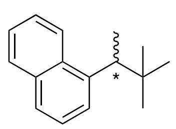

Chiradex B-DM column (100 kPa H_2, 60 °C for 30 min, 3 °C/min until 175 °C). t_R 62.3 min (*S*); t_R 62.7 min (*R*). ^1H NMR ($CDCl_3$), δ: 0.99 (s, 9H), 1.43 (d, 3H, *J*= 7.1 Hz), 3.70 (q, 1H, *J*= 7.1 Hz), 7.46-7.54 (m, 4H), 7.75-7.77 (m, 1H), 7.88-7.90 (m, 1H), 8.29-8.32 (m, 1H).

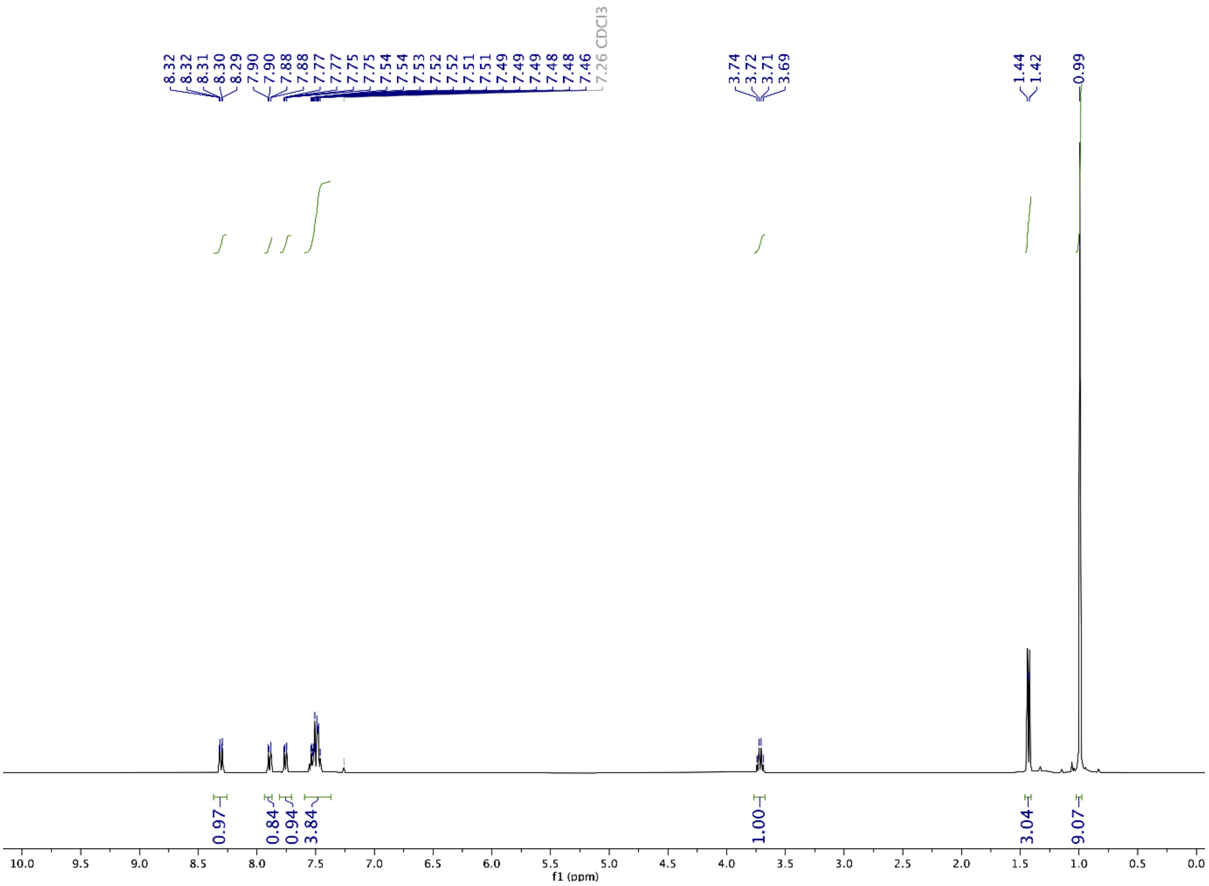

Figure S30. ^1H NMR spectrum of 1-(3,3-dimethylbutan-2-yl)naphthalene (from **S11**)in $CDCl_3$.

(a)

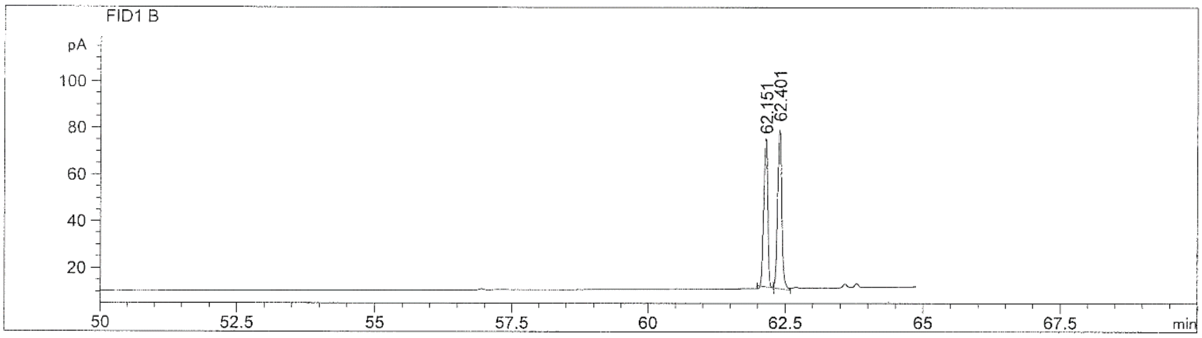

(b)

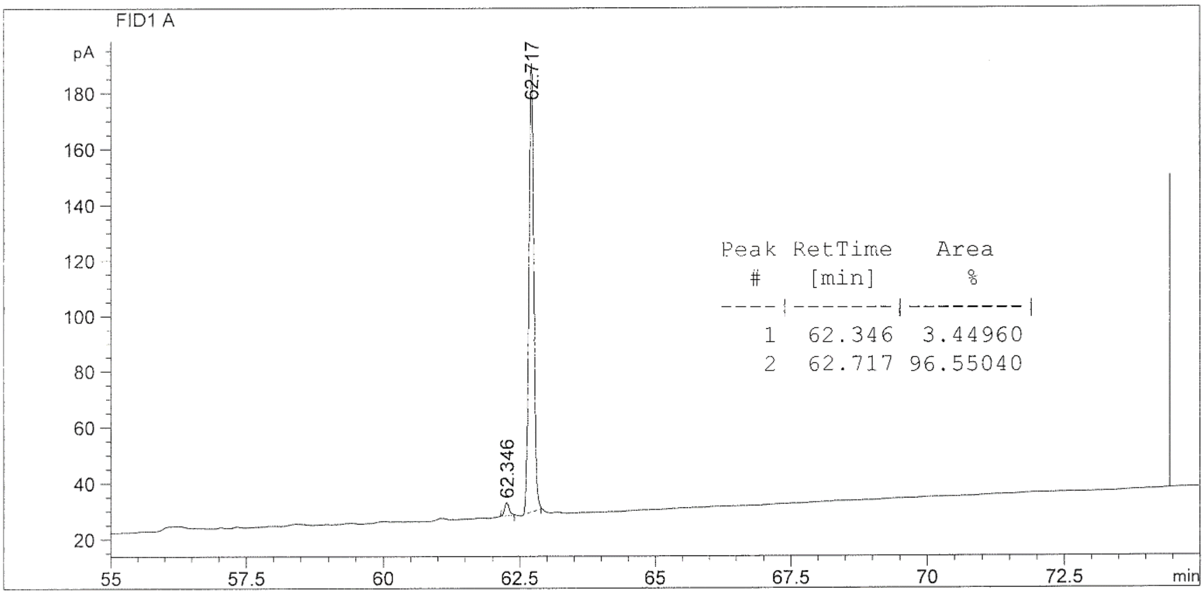

Peak #	RetTime [min]	Area %
1	62.346	3.44960
2	62.717	96.55040

Figure S31. GC trace of: (a) racemic and (b) enantioenriched product (93% (*R*) ee using **4c** catalytic system; Table 3, entry 7).

1-Methoxy-4-(1-phenylpropan-2-yl)benzene (from S13).[39] Enantiomeric excess determined by HPLC

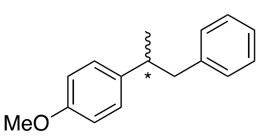

using Chiracel OJ-H column (hexane/2-propanol=95/5, 0.5 mL/min, 210 nm). t_R 16.2 min (*R*); t_R 19.4 min (*S*). ^{1}H NMR ($CDCl_3$), δ: 1.21 (d, 3H, *J*= 6.8 Hz), 2.71-2.76 (m, 1H), 2.87-2.97 (m, 2H), 3.79 (s, 3H), 6.81-6.83 (d, 2H, *J*= 8.7 Hz), 7.05-7.26 (m, 7 H).

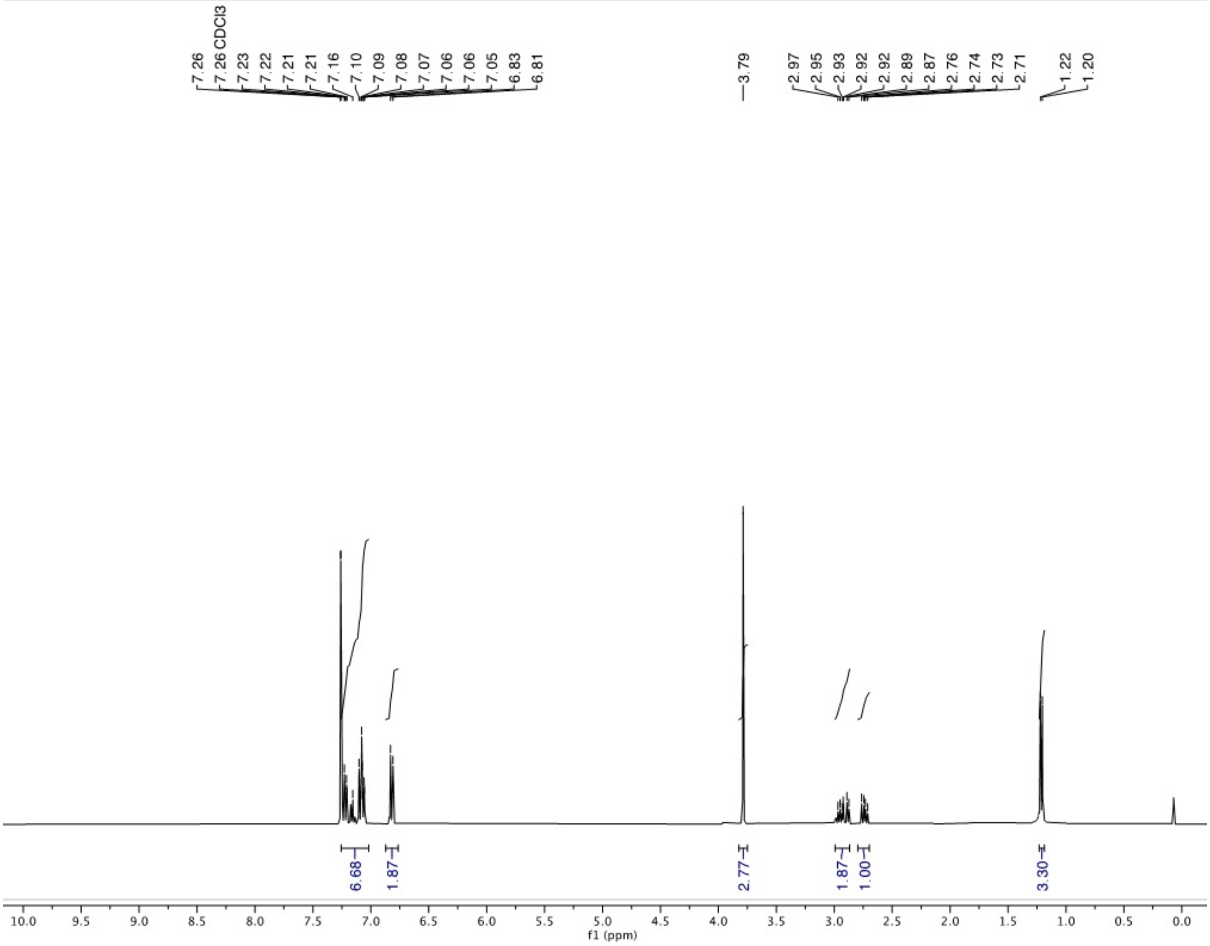

Figure S32. ^{1}H NMR spectrum of 1-methoxy-4-(1-phenylpropan-2-yl)benzene (from **S13**) in $CDCl_3$.

(a)

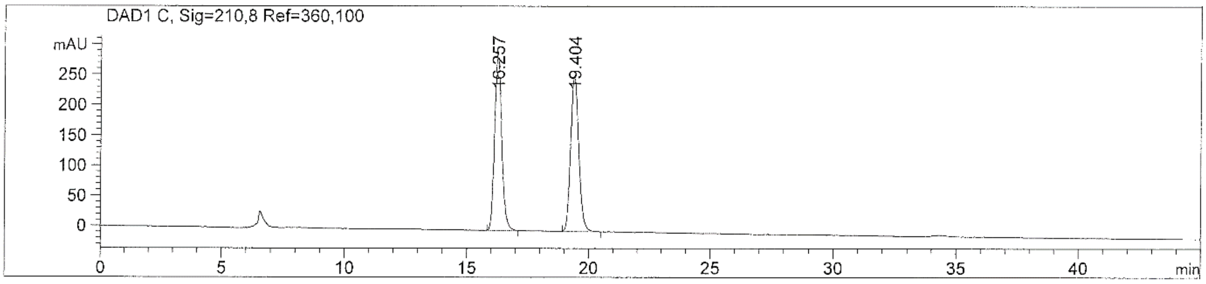

(b)

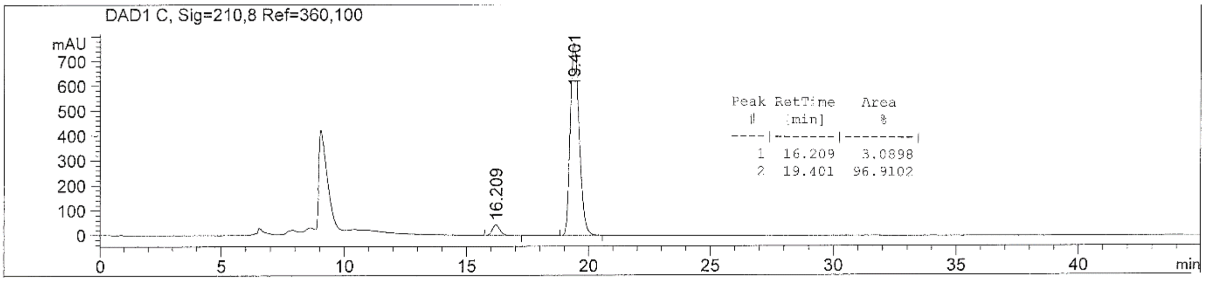

Figure S33. HPLC trace of enantioenriched product (94% (*S*) ee using**4c** catalytic system; Table 3, entry 10).

Propane-1,2-diyldibenzene (from S14).[40] Enantiomeric excess determined by HPLC using Chiracel OJ-H column (hexane/2-propanol=99/1, 0.5 mL/min, 220 nm). t_R 13.8 min (*R*); t_R 21.1 min (*S*). ^1H NMR ($CDCl_3$), δ: 1.27 (d, 3H, *J*= 6.8 Hz), 2.77-2.82 (m, 1H), 2.96-3.06 (m, 2H), 7.10-7.12 (m, 2H), 7.19-7.33 (m, 8H).

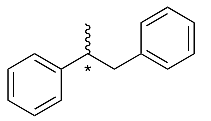

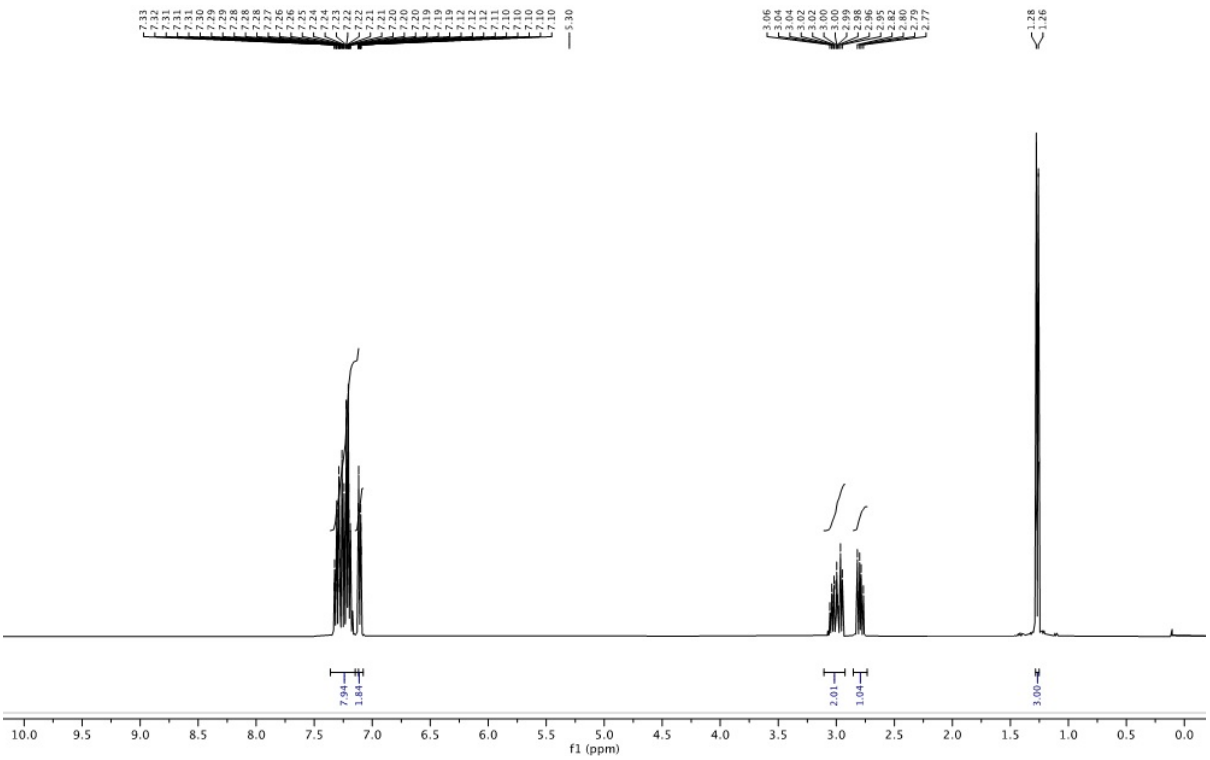

Figure S34. ^1H NMR spectrum of propane-1,2-diyldibenzene (from **S14**) in $CDCl_3$.

(a)

(b)

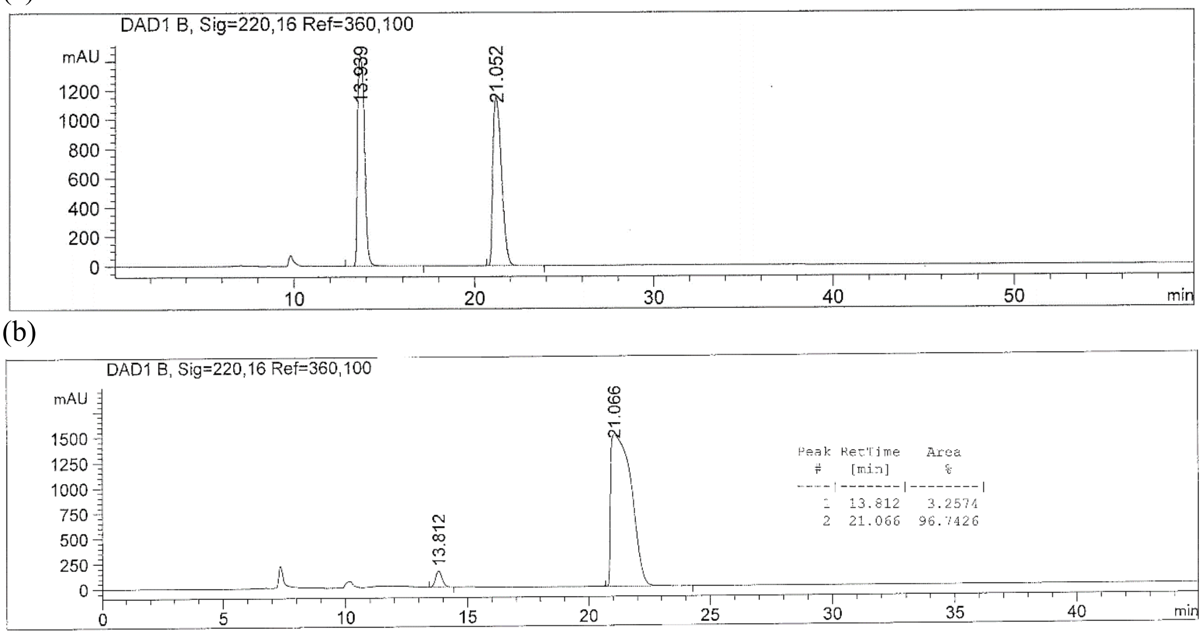

Figure S35. HPLC trace of: (a) racemic and (b) enantioenriched product (93% (*S*) ee using **4c** catalytic system; Table 3, entry 11).

6-Methoxy-1-naphthyl-1,2,3,4-tetrahydronaphthalene (from S16).[40] Enantiomeric excess determined

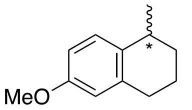

by GC using Chiradex B-DM column (100 kPa H_2, 60 °C for 30 min, 3 °C/min until 175 °C). t_R 59.7 min (*R*); t_R 59.9 min (*S*). ^{1}H NMR ($CDCl_3$), δ: 1.27 (d, 3H, *J*= 7.0 Hz), 1.49-1.56 (m, 1H), 1.70-1.74 (m, 1H), 1.84-1.93 (m, 2H), 2.72-2.77 (m, 2H), 2.85-2.86 (m, 1H), 3.78 (s, 3H), 6.61 (d, 1H, *J*= 2.8 Hz), 6.72 (dd, 1H, *J*= 8.5, *J*= 2.8 Hz), 7.12 (d, 1H, *J*= 8.5 Hz).

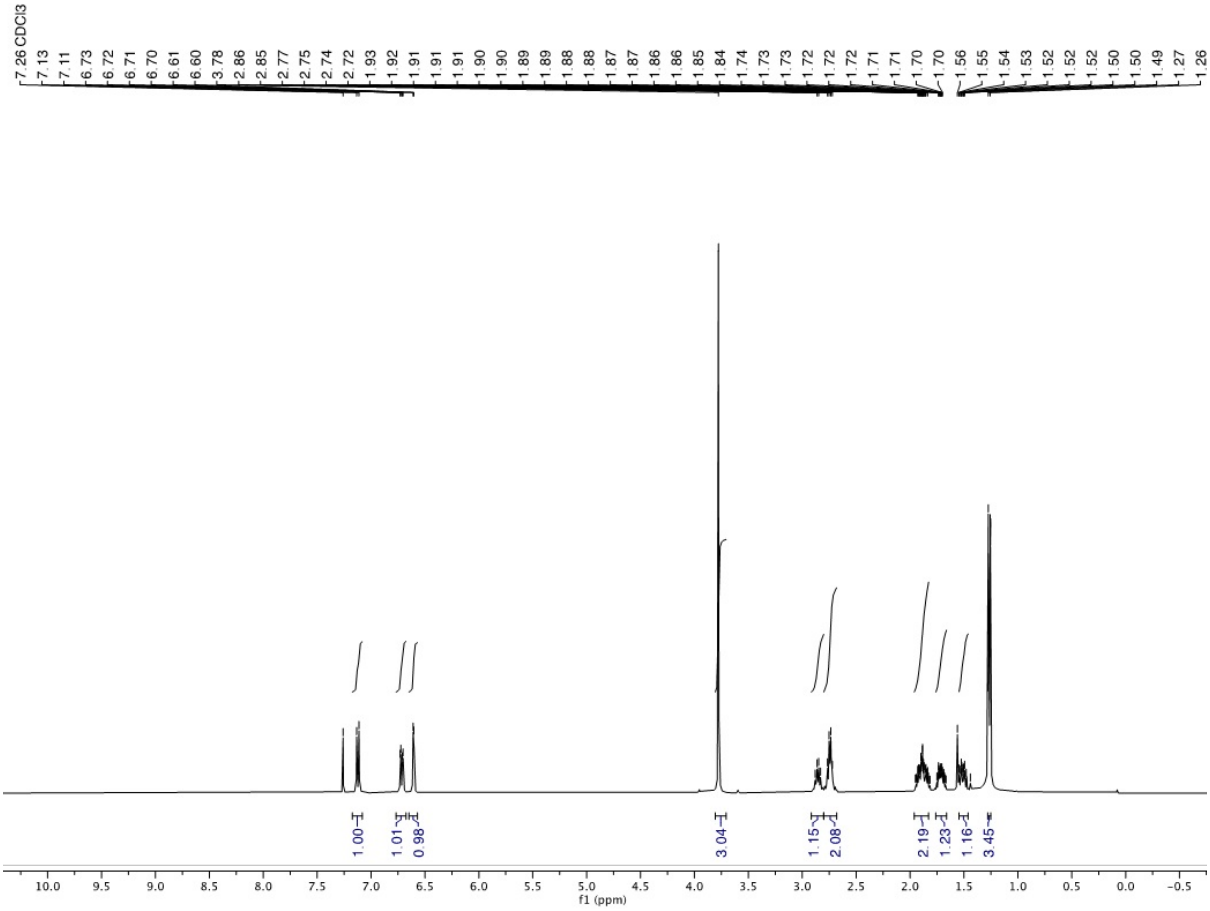

Figure S36. ^{1}H NMR spectrum of 6-methoxy-1-naphthyl-1,2,3,4-tetrahydronaphthalene (from **S16**) in $CDCl_3$.

(a)

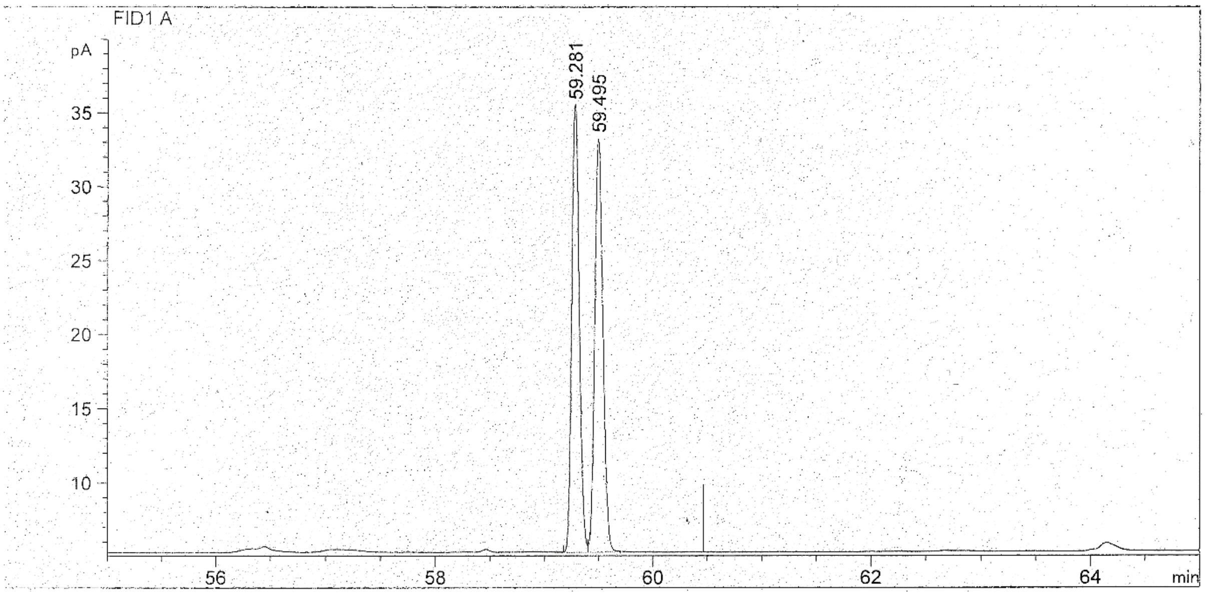

(b)

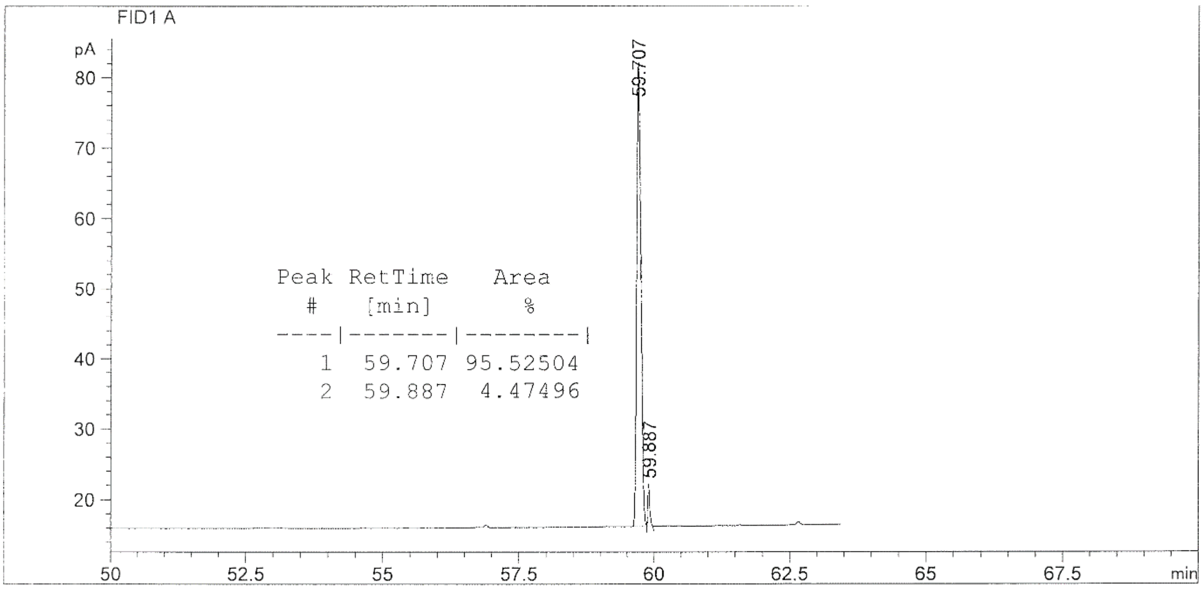

Figure S37. GC trace of: (a) racemic and (b) enantioenriched product (91% (*R*) ee using **4c** catalytic system; Table 3, entry 13).

1-Isopropyl-6-methoxy-1,2,3,4-tetrahydronaphthalene (from S17).[41] Enantiomeric excess determined

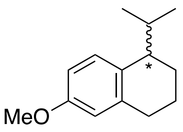

by GC using Chiradex B-DM column (100 kPa H_2, 60 °C for 30 min, 3 °C/min until 175 °C). t_R 65.0 min (*R*); t_R 65.2 min (*S*). ^{1}H NMR ($CDCl_3$), δ: 0.74 (d, 3H, *J*= 6.9 Hz), 1.00 (d, 3H, *J*= 6.9 Hz), 1.57-1.62 (m, 2H), 1.77-1.79 (m, 1H), 1.90-191 (m, 1H), 2.17-2.22 (m, 1H), 2.64-2.75 (m, 3H), 3.78 (s, 3H), 6.60 (d, 1H, *J*= 2.7 Hz), 6.70 (dd, 1H, *J*= 8.6 Hz, *J*= 2.8 Hz), 7.12 (dd, 1H, *J*= 8.5 Hz, *J*= 0.8 Hz).

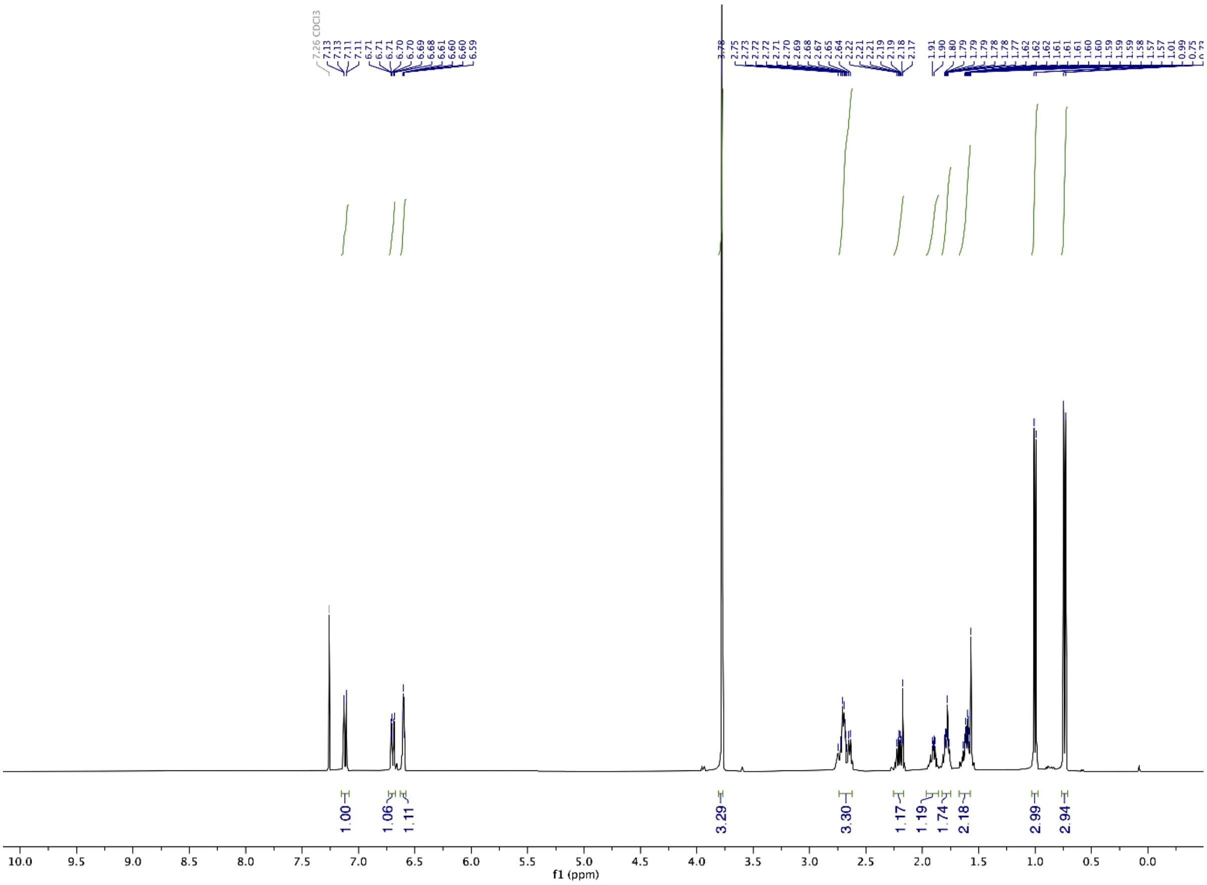

Figure S38. ^{1}H NMR spectrum of 1-isopropyl-6-methoxy-1,2,3,4-tetrahydronaphthalene (from **S17**) in $CDCl_3$.

(a)

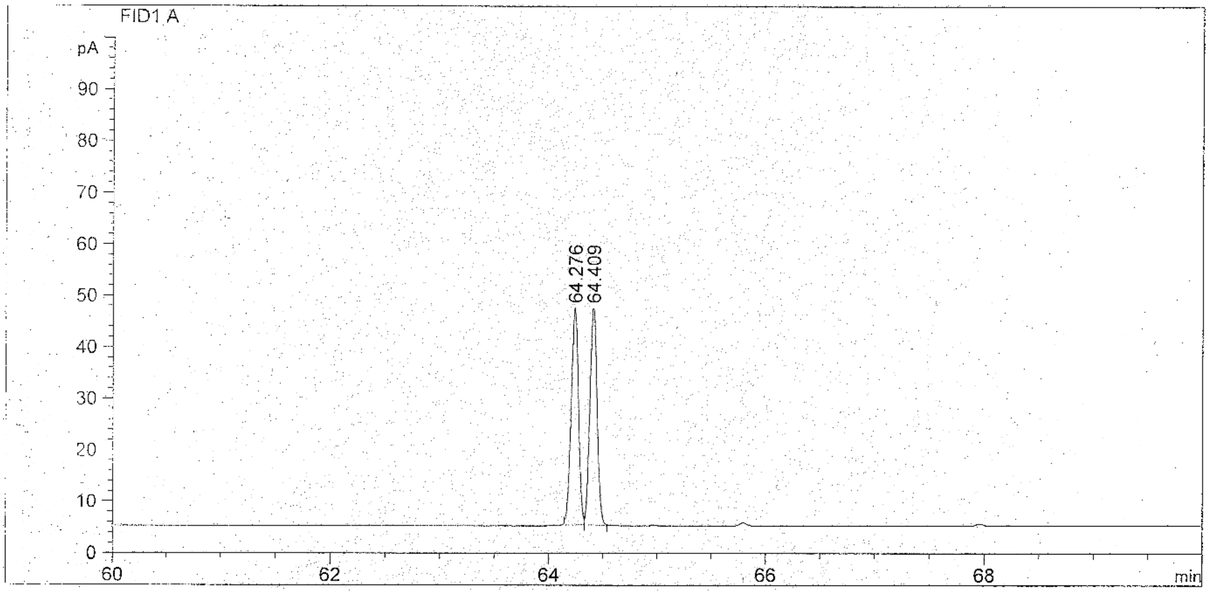

(b)

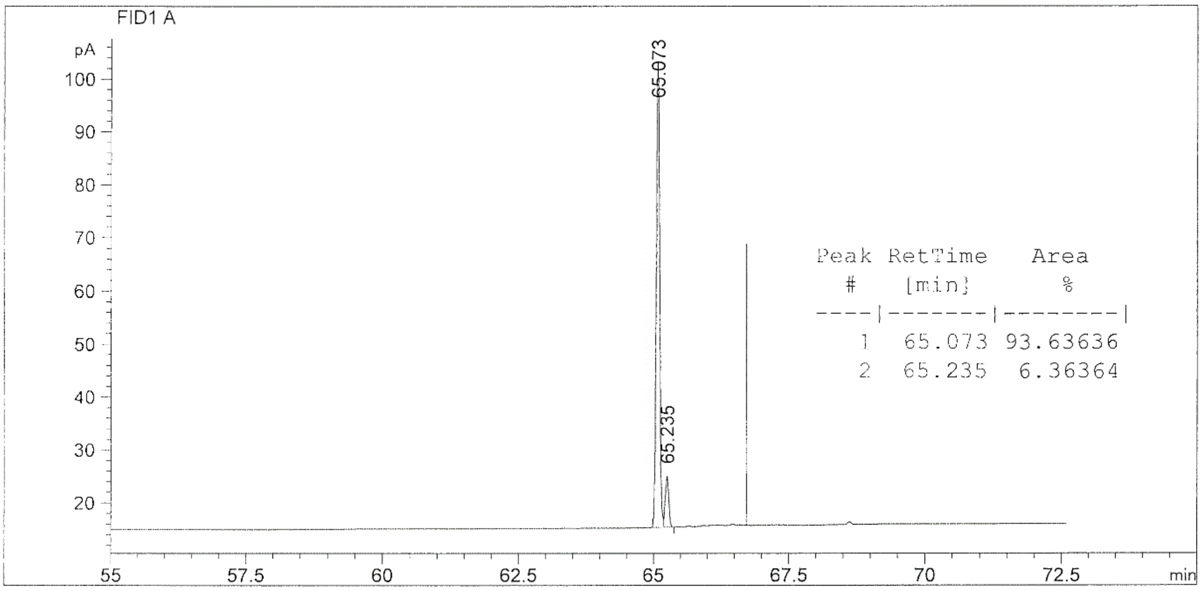

Figure S39. GC trace of: (a) racemic and (b) enantioenriched product (87% (*R*) ee using **4c** catalytic system; Table 3, entry 14).

(1-(3,5-Dimethylphenyl)ethane-1,2-diyl)dibenzene (from S18).[20] Enantiomeric excess determined by HPLC using Chiracel OJ-H column (hexane/2-propanol=98/2, 0.4 mL/min, 220 nm). t_R 18.7 min (-); t_R 21.9 min (+). ^{1}H NMR ($CDCl_3$), δ: 2.26 (s, 6H), 3.32-3.35 (m, 2H), 4.13-4.17 (m, 1H), 6.81 (s, 1H), 6.86 (s, 2H), 6.99-7.02 (m, 2H), 7.12-7.26 (m, 8H).

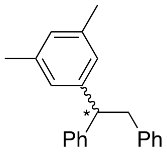

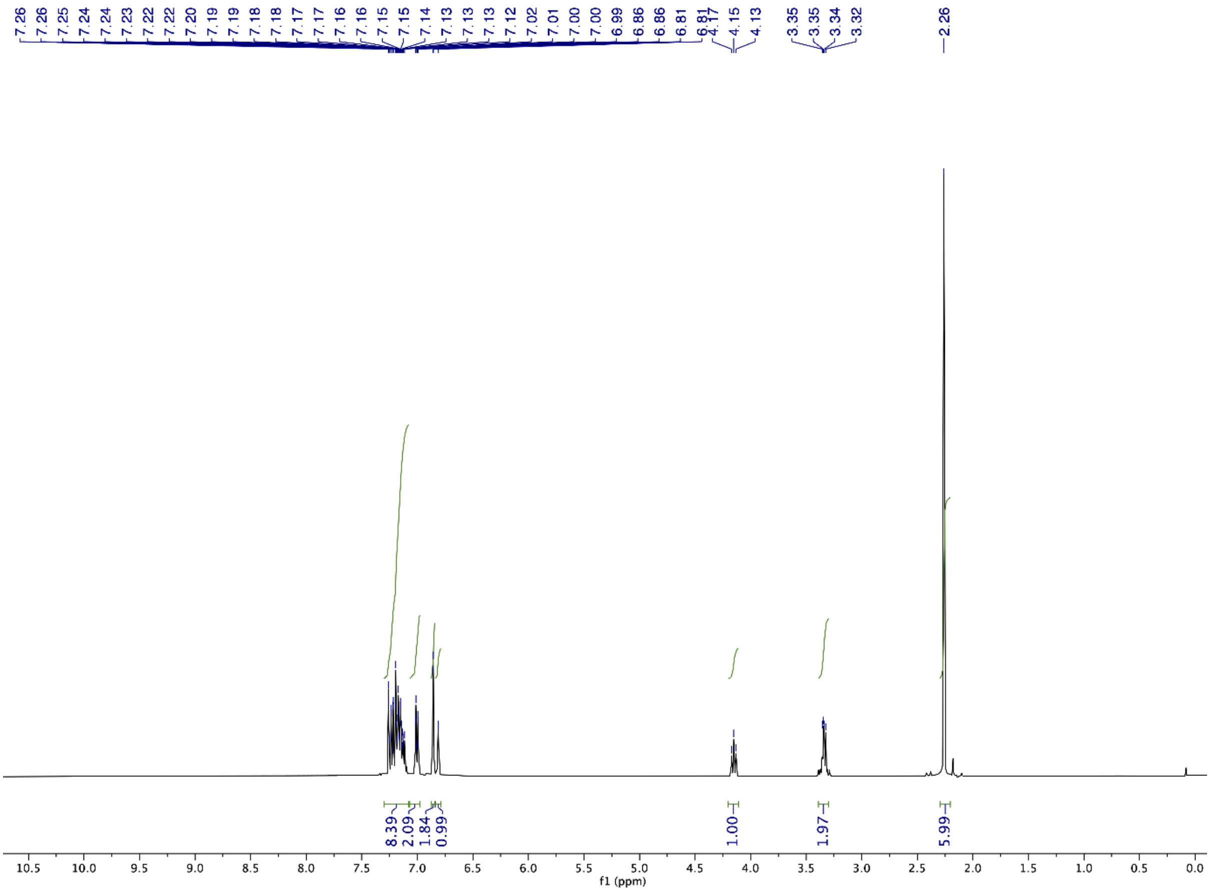

Figure S40. ^{1}H NMR spectrum of (1-(3,5-dimethylphenyl)ethane-1,2-diyl)dibenzene (from **S18**) in $CDCl_3$.

(a)

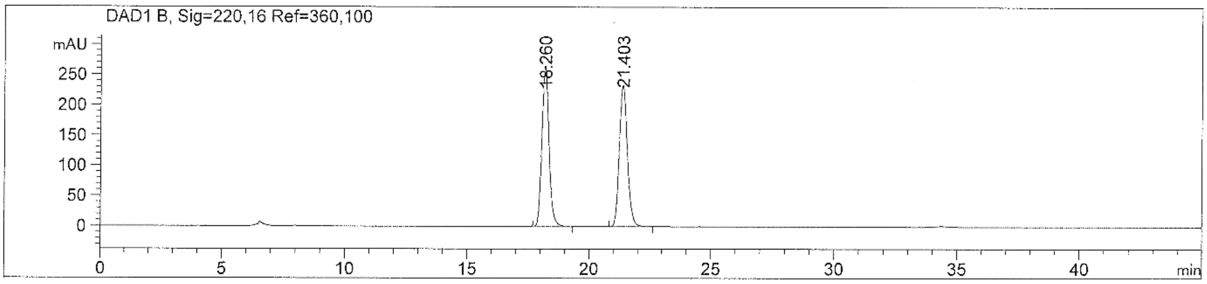

(b)

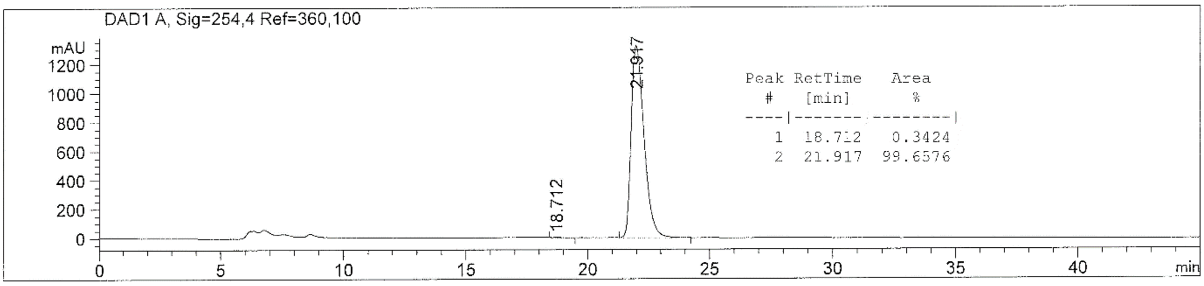

Figure S41. HPLC trace of enantioenriched product (99% (*R*) ee using **4c** catalytic system; Table 3, entry 15).

(1-(4-Methoxyphenyl)ethane-1,2-diyl)dibenzene (from S19).[20] Enantiomeric excess determined by HPLC using Chiracel OJ-H column (hexane/2-propanol=80/20, 0.5 mL/min, 254 nm). t_R 17.2 min (-); t_R 19.3 min (+).^{1}H NMR ($CDCl_3$), δ: 3.32 (d, 2H, *J*= 7.8 Hz), 3.76 (s, 3H), 4.18 (t, 1H, *J*= 7.8 Hz), 6.78 (d, 2H, *J*= (8.7 Hz), 6.99-7.01 (m, 2H), 7.10-7.26 (m, 10H).

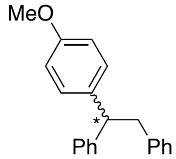

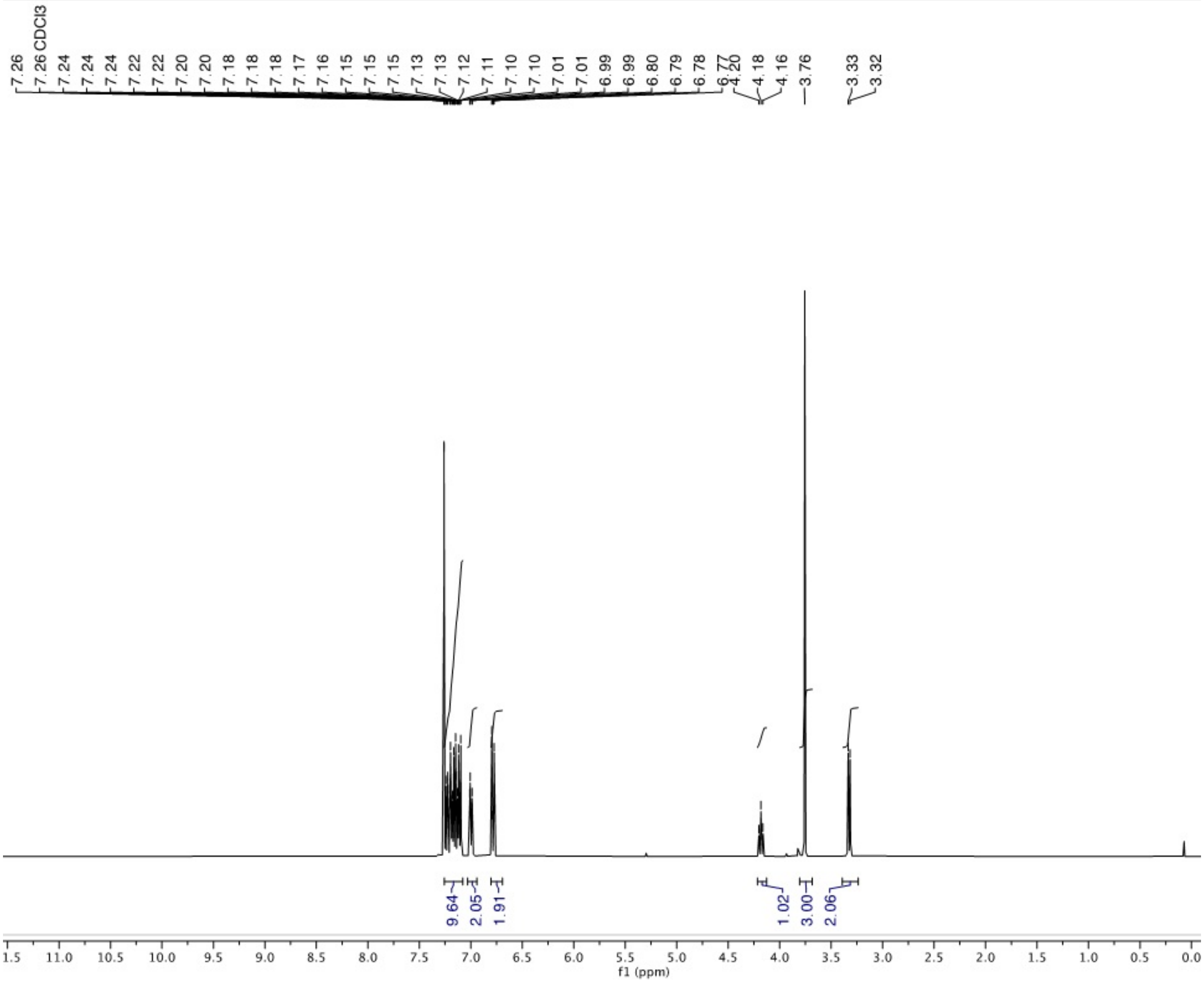

Figure S42. ^{1}H NMR spectrum of (1-(4-methoxyphenyl)ethane-1,2-diyl)dibenzene (from **S19**) in $CDCl_3$.

(a)

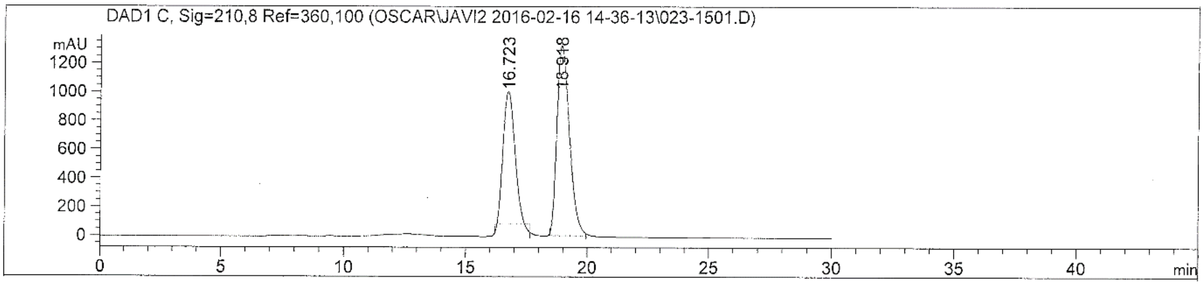

(b)

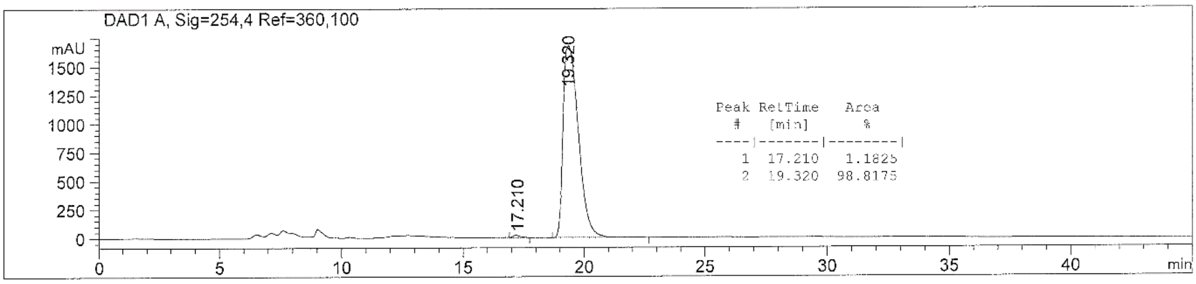

Figure S43. HPLC trace of: (a) racemic and (b) enantioenriched product (98% (*R*) ee using **4c** catalytic system; Table 3, entry 16).

2,3-Dimethylpentane (from S20).[42] Enantiomeric excess determined by GC using Chiradex B-DM

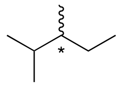

column (50 kPa H_2, 30 °C – 0.1 °C/min until 40 °C). t_R 7.7 min (*S*; t_R 7.9 min (*R*). ^1H NMR (CD_2Cl_2), δ: 0.78 (d, 3H, *J*= 6.6 Hz), 0.79 (d, 3H, *J*= 6.6 Hz), 0.83-0.86 (m, 6H), 1.05-1.20 (m, 2H), 1.32-1.38 (m, 1H), 1.51-1.58 (m, 1H).

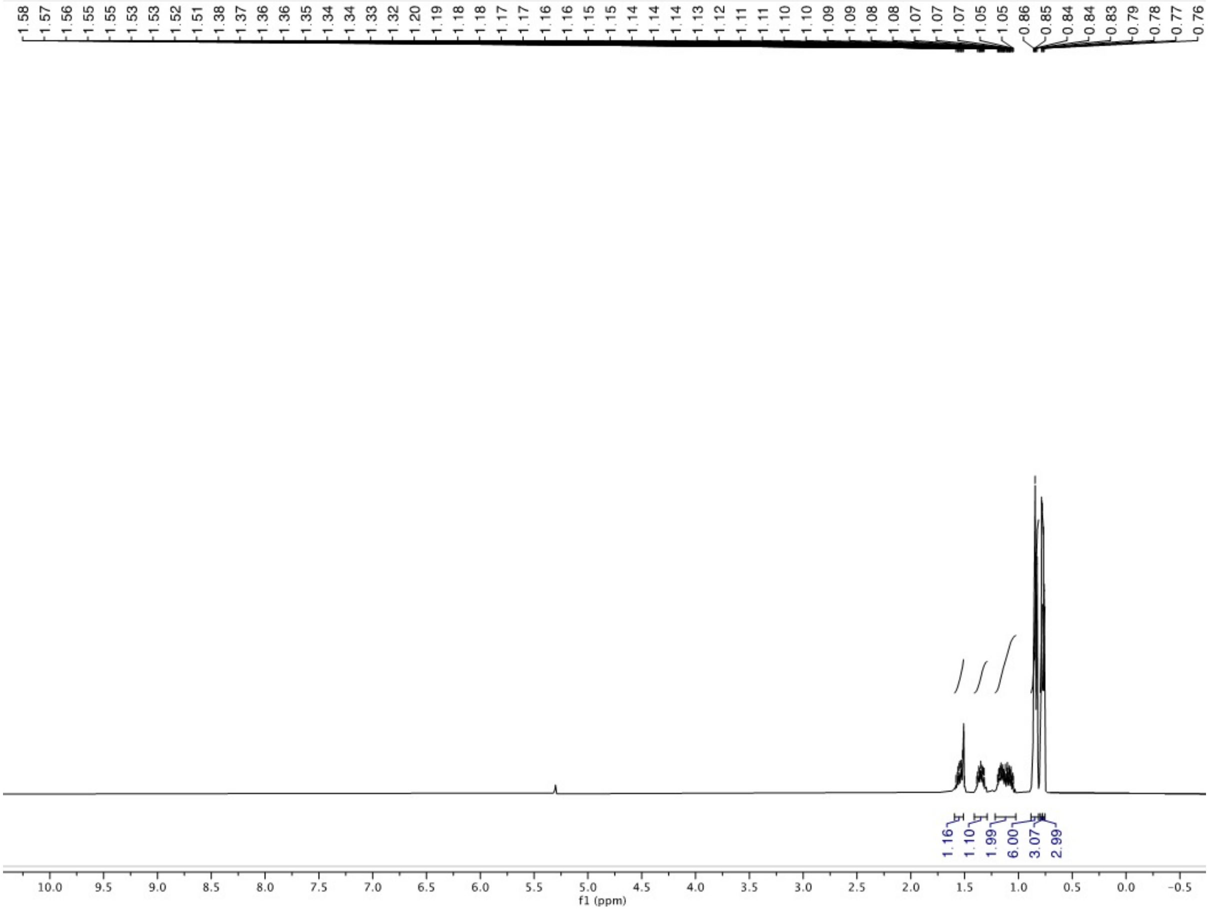

Figure S44. ^1H NMR spectrum of 2,3-dimethylpentane (from **S20**) in CD_2Cl_2.

(a)

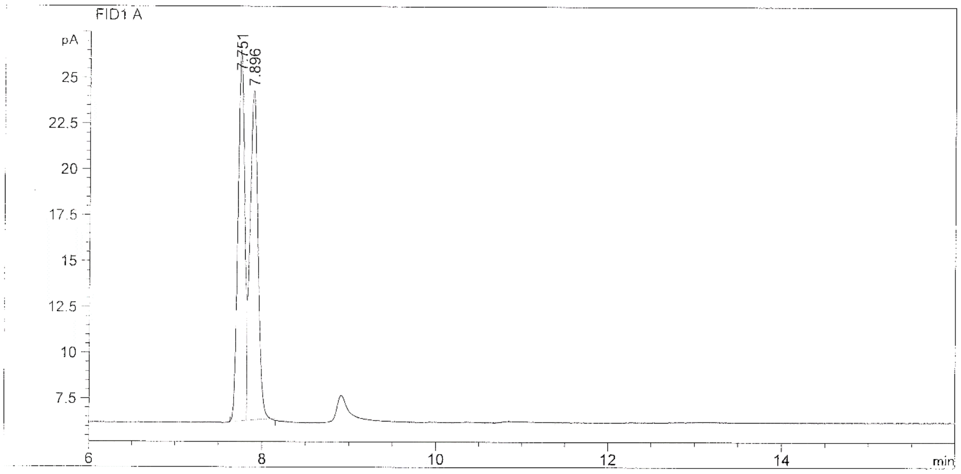

(b)

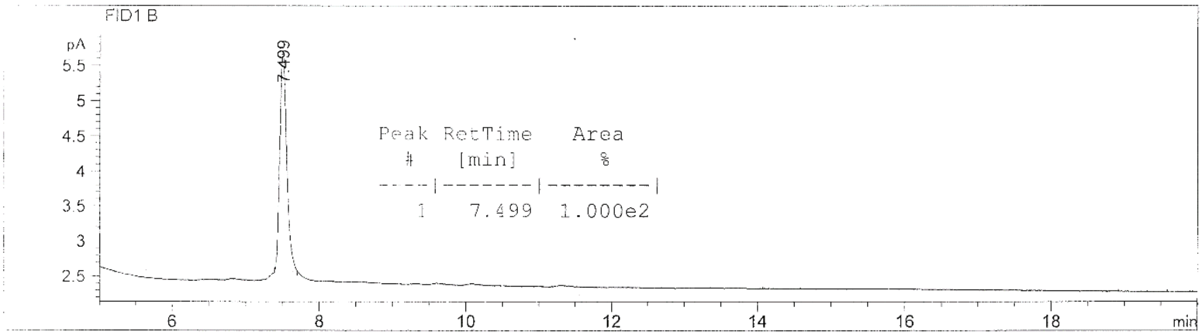

Figure S45. GC traces of (a) racemic and (b) enantioenriched product (>98% (*S*) ee using **4c** catalytic system; Table 3, entry 17).

***sec*-Butylcyclohexane (from S21).**[43] Enantiomeric excess determined by GC using Chiradex B-DM column (50 kPa H_2, 50 °C for 60 min, 3 °C/min until 175 °C). t_R 67.7 min (*S*); t_R 68.1 min (*R*). ^{1}H NMR (CD_2Cl_2), δ: 0.78-1.72 (complex multiplets).

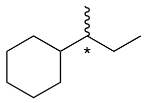

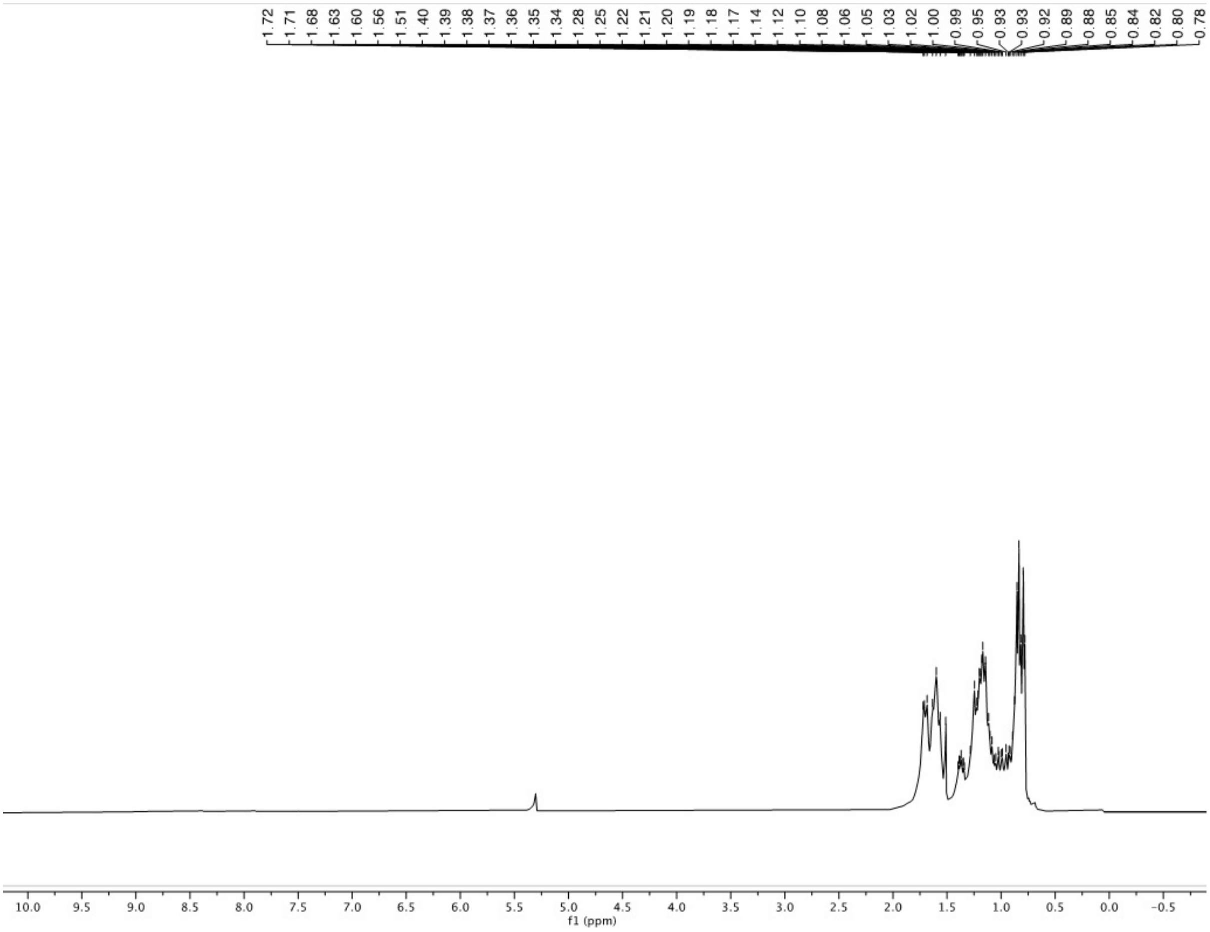

Figure S46. ^{1}H NMR spectrum of *sec*-butylcyclohexane (from **S21**) in CD_2Cl_2.

(a)

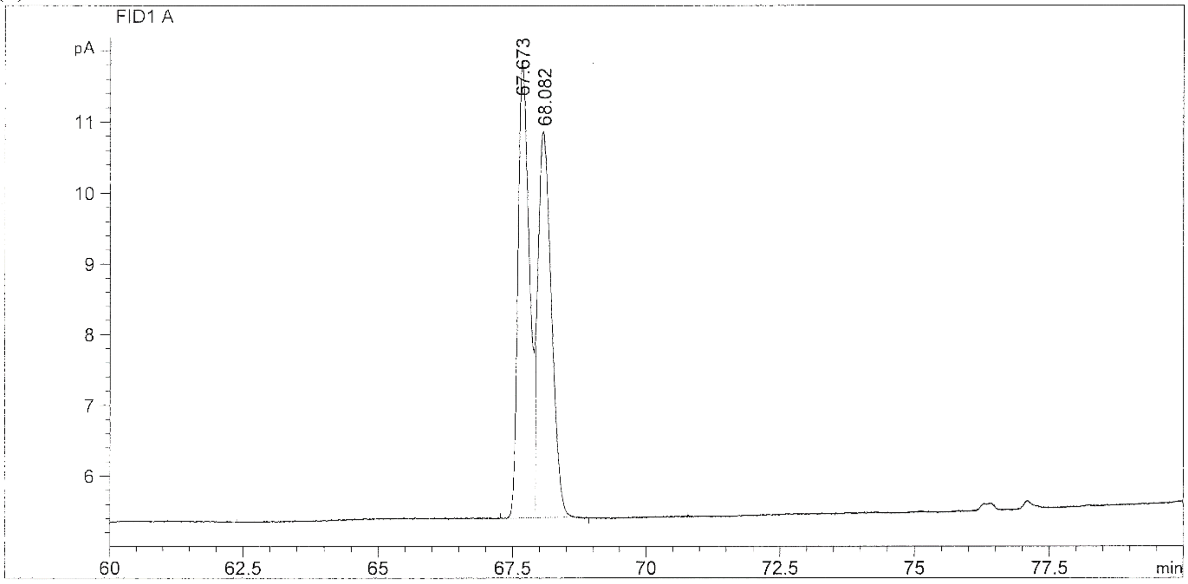

(b)

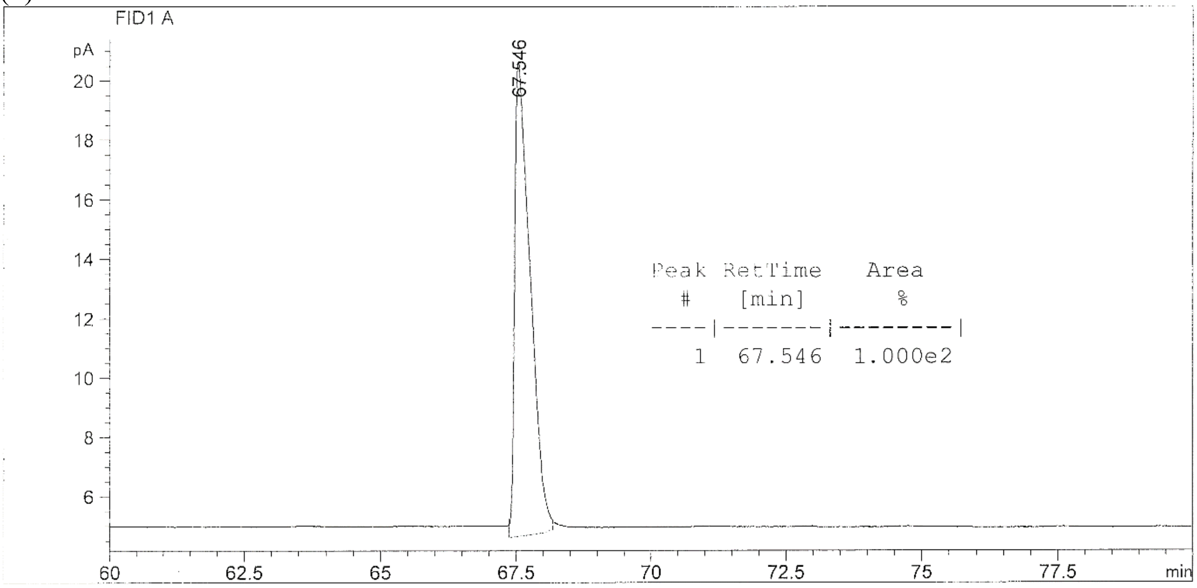

Figure S47. GC traces of (a) racemic and (b) enantioenriched product (>98% (*S*) ee using **4c** catalytic system; Table 3, entry 18).

1-Benzyl-2,3-dihydro-1H-indene (from S22).[22] Enantiomeric excess determined by HPLC using Chiracel OJ-H column (hexane/2-propanol=95/5, 0.5 mL/min, 210 nm). t_R 11.1 min (*S*); t_R 12.9 min (*R*). ^{1}H NMR ($CDCl_3$), δ: 1.75–1.81 (m, 1H), 2.14–2.18 (m, 1H), 2.68 (dd, 1H, *J*= 13.6 Hz, *J*= 9.2 Hz), 2.75–2.92 (m, 2H), 3.15 (dd, 1H, *J*= 13.6 Hz, *J*= 5.6 Hz), 3.42–3.49 (m, 1H), 7.11–7.17 (m, 3H), 7.20–7.32 (m, 6H).

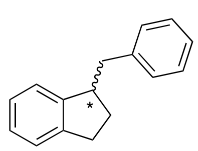

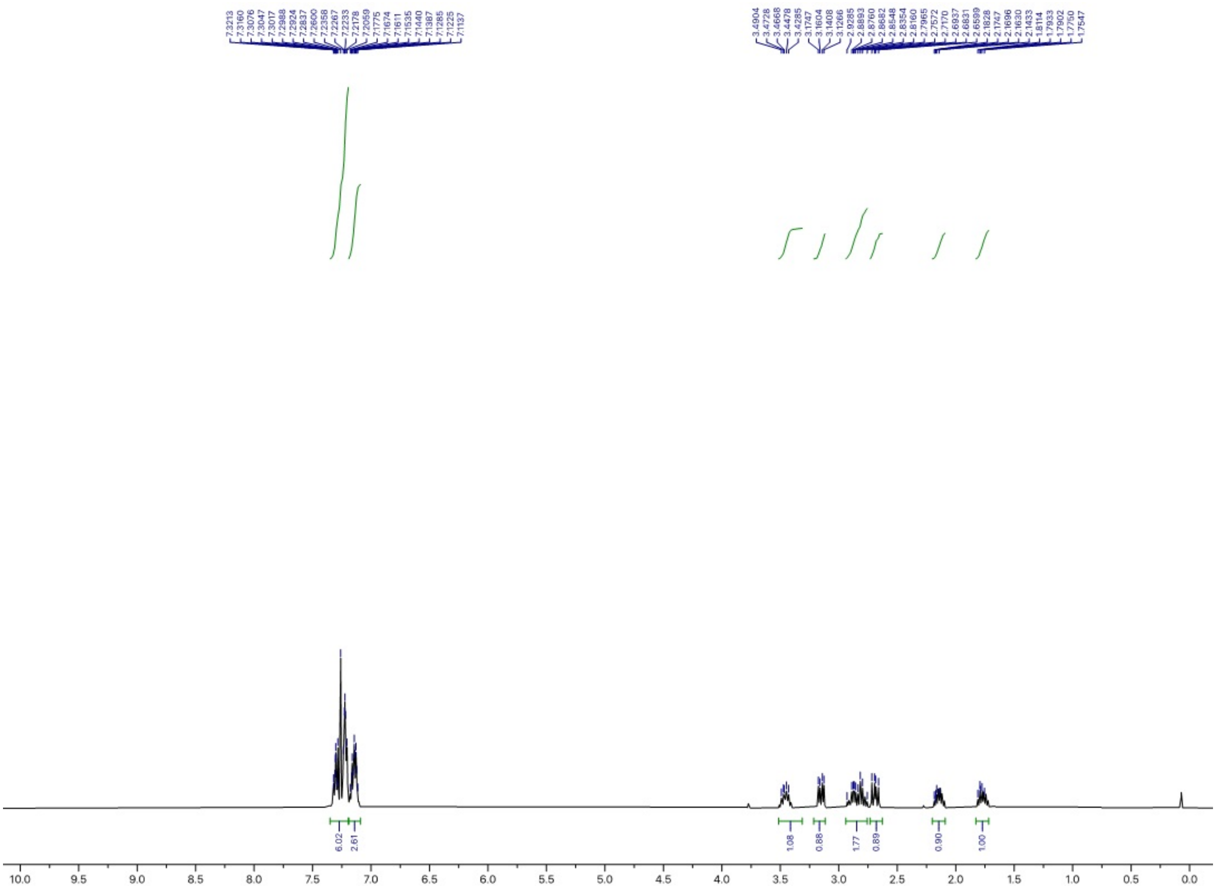

Figure S48. ^{1}H NMR spectrum of 1-benzyl-2,3-dihydro-1*H*-indene (from **S22**) in $CDCl_3$.

(a)

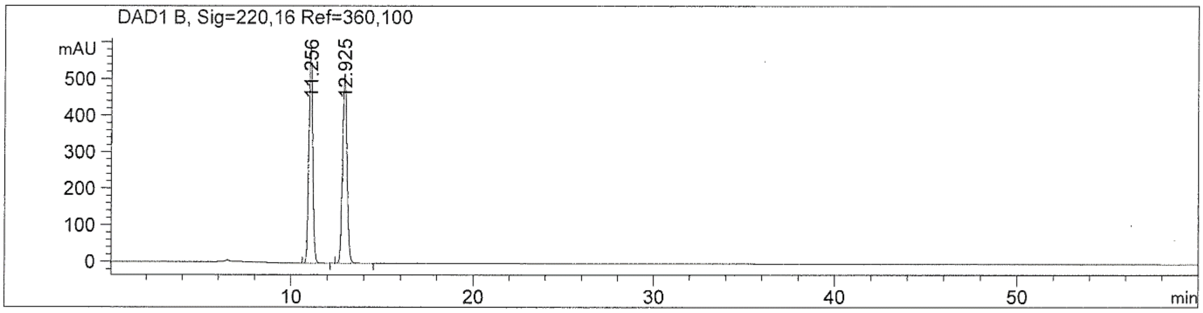

(b)

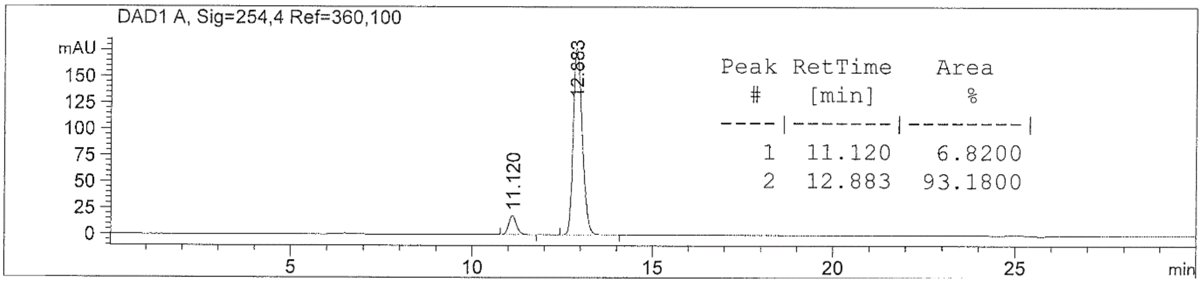

Figure S49. HPLC trace of (a) racemic and (b) enantioenriched product (86% (*R*) ee using **4c** catalytic system; Table 3, entry 19).

1-(4-Methylbenzyl)-2,3-dihydro-1H-indene (from S23).[22] Enantiomeric excess determined by HPLC

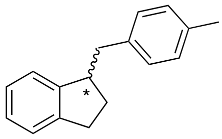

using Chiralcel OJ-H column (hexane/2-propanol=98/2, 0.5 ml/min, 254 nm). t_R 11.2 min (*S*); t_R 12.0 min (*R*). ^{1}H NMR ($CDCl_3$), δ: 1.72–1.80 (m, 1H), 2.09–2.18 (m, 1H), 2.35 (s, 3H), 2.64 (dd, 1H, *J*= 6.1 Hz, *J*= 13.5 Hz), 2.75–2.93 (m, 2H), 3.12 (dd, 1H, *J*= 5.6 Hz, *J*= 13.6 Hz), 3.38–3.46 (m, 1H), 7.10–7.18 (m, 7H), 7.21–7.24 (m, 1H).

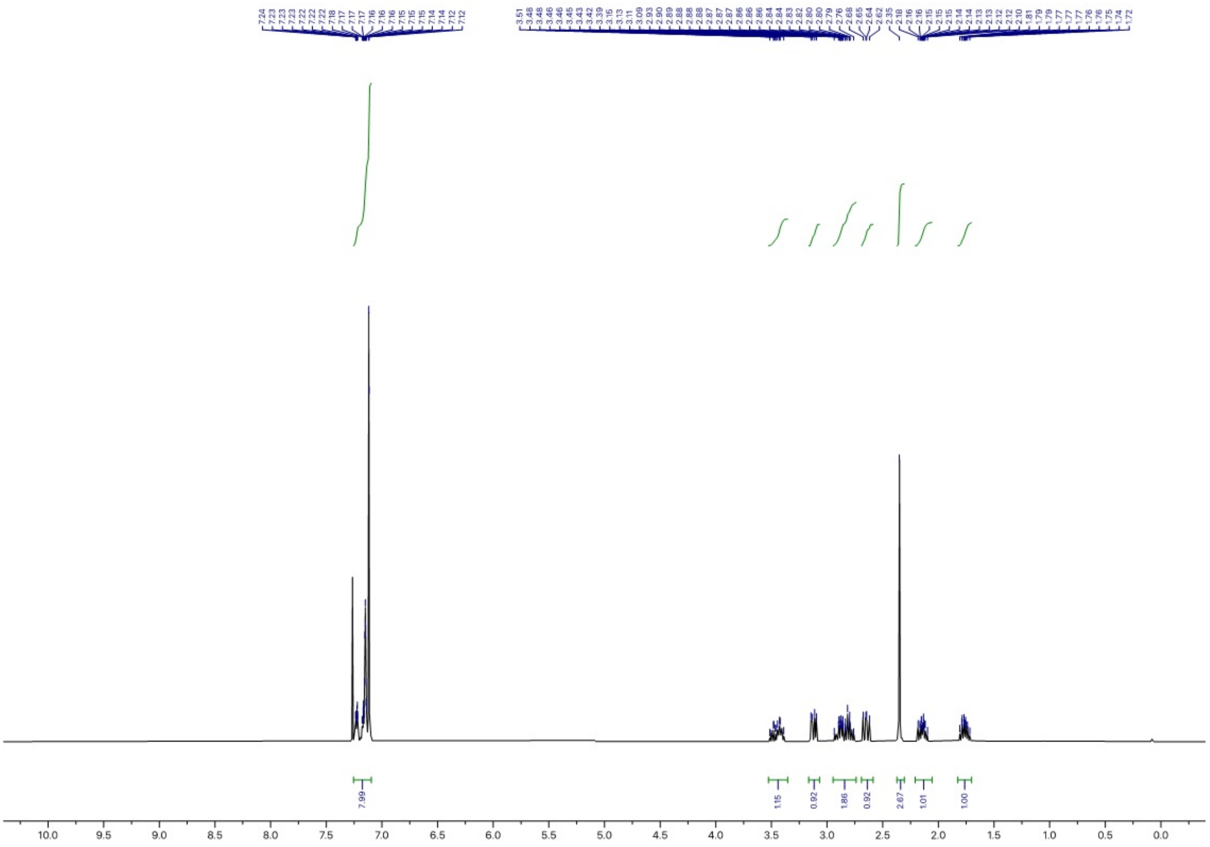

Figure S50. ^{1}H NMR spectrum of 1-(4-methylbenzyl)-2,3-dihydro-1*H*-indene (from **S23**) in $CDCl_3$.

(a)

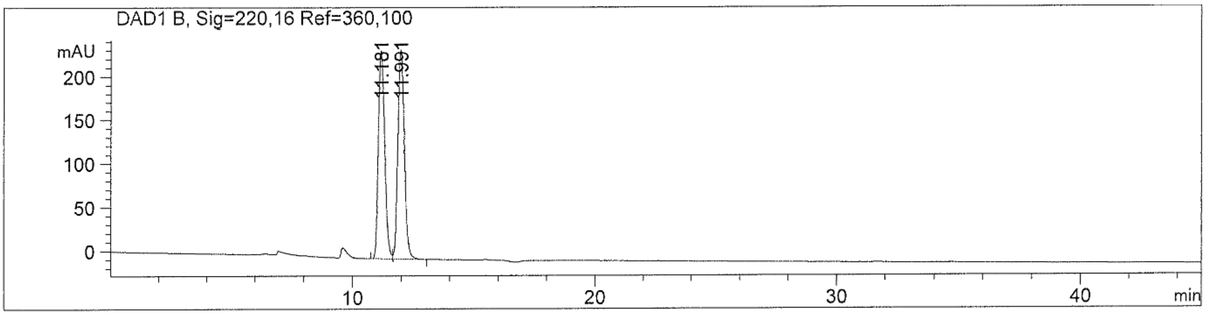

(b)

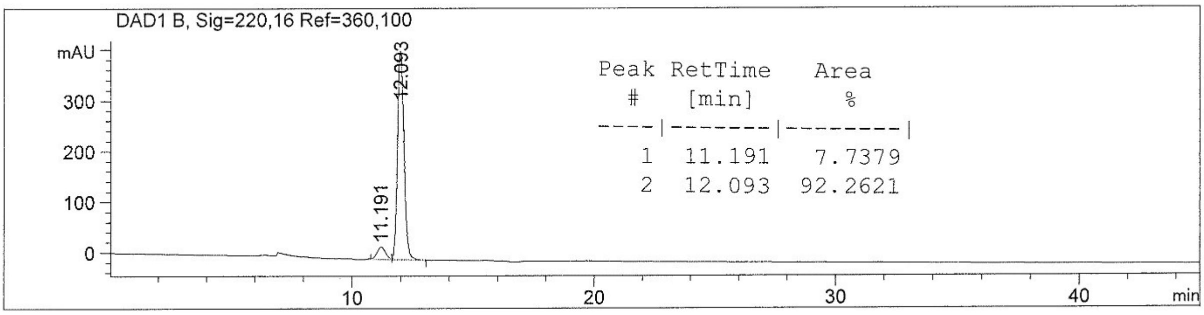

Figure S51. HPLC trace of: (a) racemic and (b) enantioenriched product (84% (*R*) ee using **4c** catalytic system; Table 3, entry 20).

1-(4-Methoxybenzyl)-2,3-dihydro-1H-indene (from S24).[23] Enantiomeric excess determined by HPLC using Chiralcel OJ-H column (95% 2-propanol/hexane, flow 0.5 ml/min, λ = 226 nm). t_R 15.1 min (*S*); t_R 17.3 min (*R*). ^{1}H NMR ($CDCl_3$), δ: 1.65–1.70 (m, 1H), 2.04–2.09 (m, 1H), 2.57 (dd, 1H, *J*= 13.9 Hz, *J*= 8.6 Hz), 2.71–2.80 (m, 2H), 3.00 (dd, 1H, *J*= 13.5 Hz, *J*= 5.5 Hz), 3.31–3.35 (m, 1H), 3.74 (s, 3H), 6.76–6.78 (m, 2H), 7.03–7.15 (m, 6H).

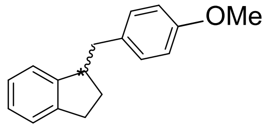

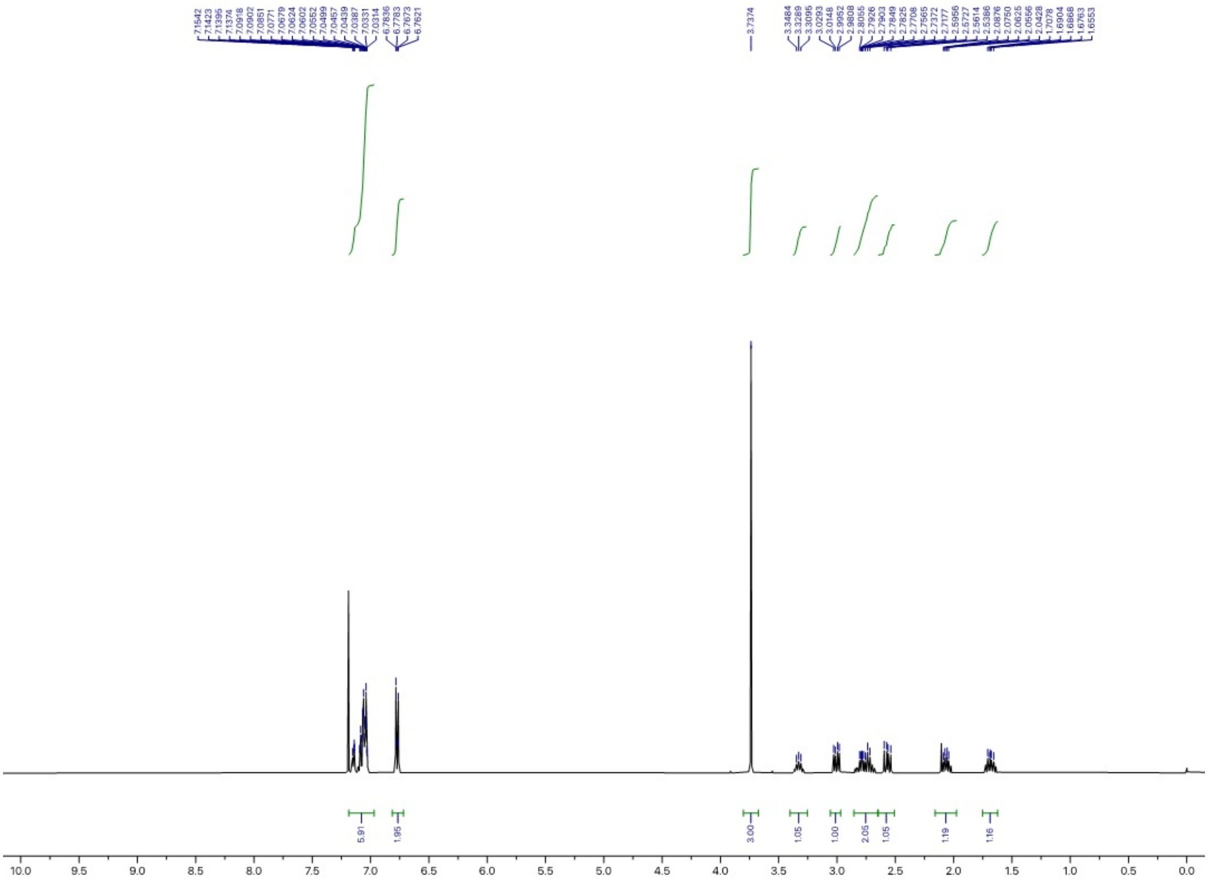

Figure S52. ^{1}H NMR spectrum of 1-(4-methoxybenzyl)-2,3-dihydro-1*H*-indene (from **S24**) in $CDCl_3$.

(a)

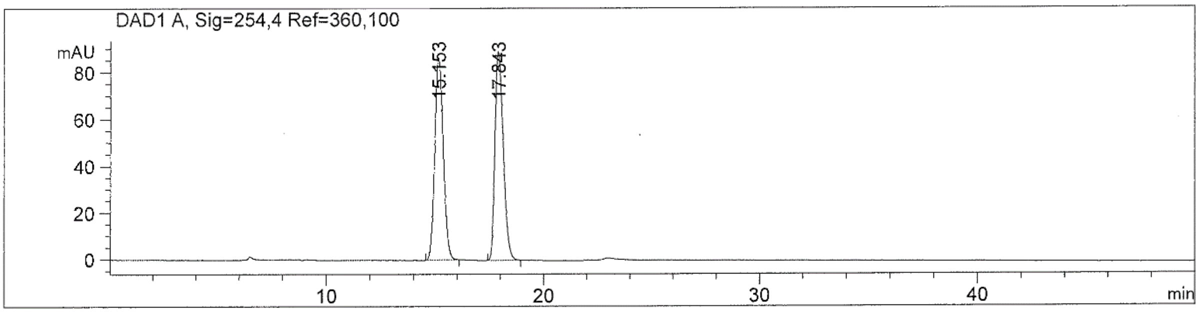

(b)

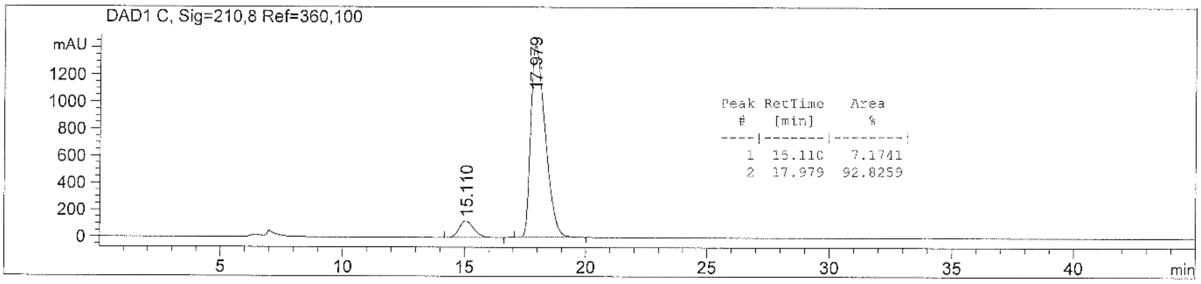

Figure S53. HPLC trace of: (a) racemic and (b) enantioenriched product (85% (*R*) ee using **4c** catalytic system; Table 3, entry 21).

1-(2-Methylbenzyl)-2,3-dihydro-1H-indene (from S25).[23] Enantiomeric excess determined by HPLC using Chiralcel OD-H column (98% 2-propanol/hexane, flow 0.5 ml/min, λ = 226 nm). t_R 8.7 min (*R*); t_R 9.4 min (*S*). ^{1}H NMR ($CDCl_3$), δ: 1.71–1.76 (m, 1H), 2.06–2.11 (m, 1H), 2.23 (s, 3H), 2.60 (dd, 1H, *J*= 13.7 Hz, *J*= 9.0 Hz), 2.73–2.77 (m, 1H), 2.86–2.90 (m, 1H), 3.07 (dd, 1H, *J*= 13.7 Hz, *J*= 5.5 Hz), 3.33–3.40 (m, 1H), 7.03–7.11 (m, 7H), 7.16–7.18 (m, 1H).

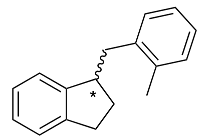

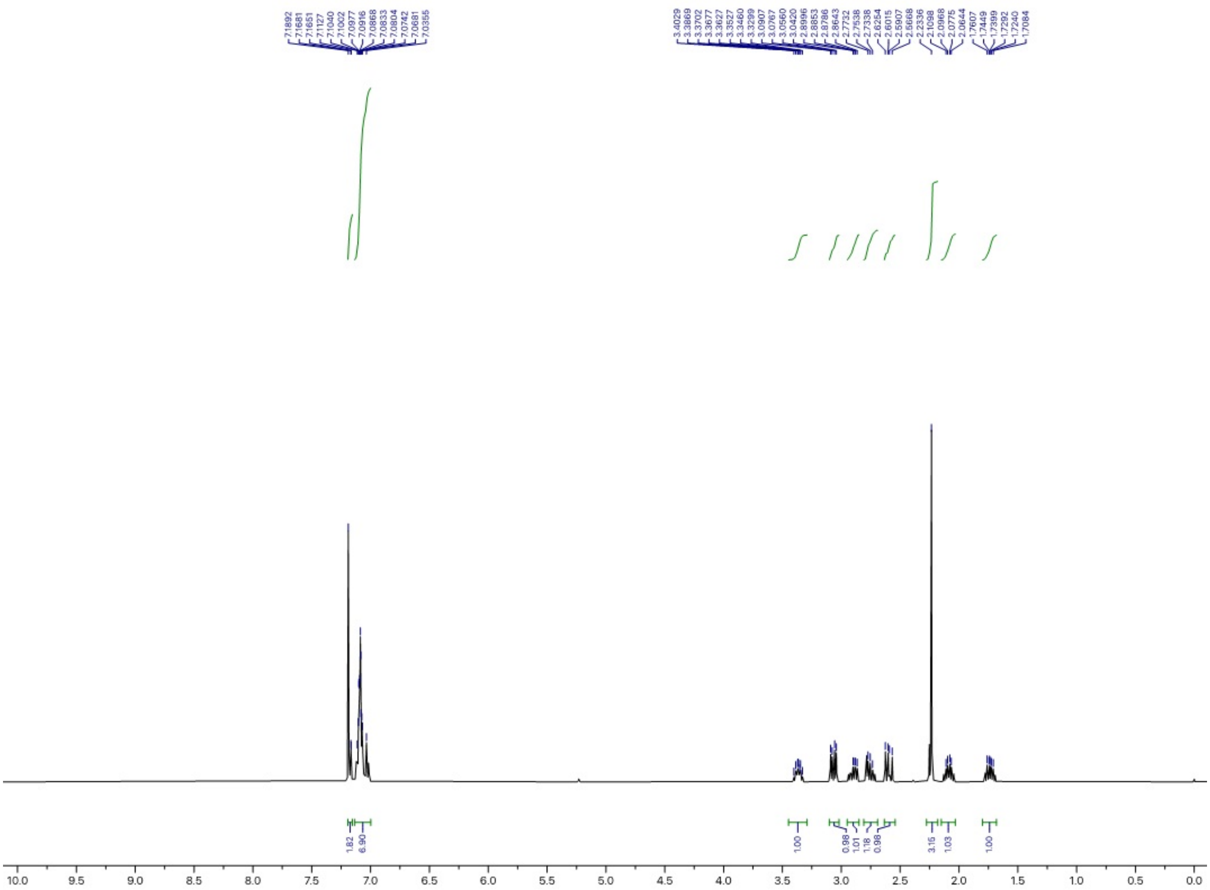

Figure S54. ^{1}H NMR spectrum of 1-(2-methylbenzyl)-2,3-dihydro-1*H*-indene (from **S25**) in $CDCl_3$.

(a)

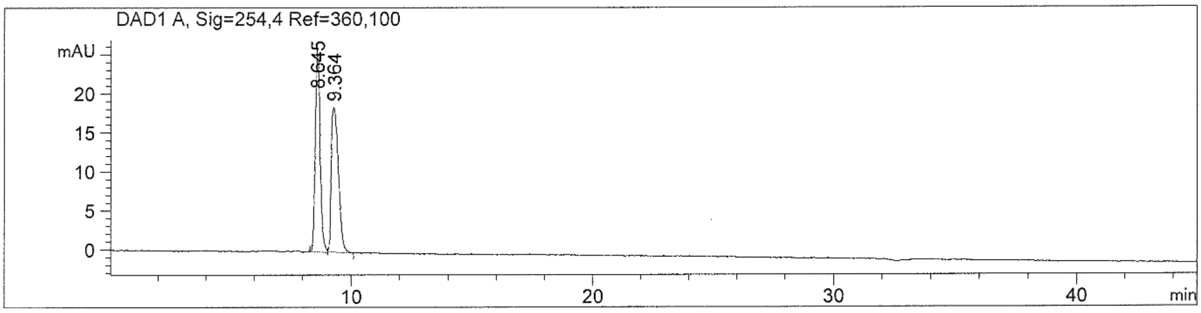

(b)

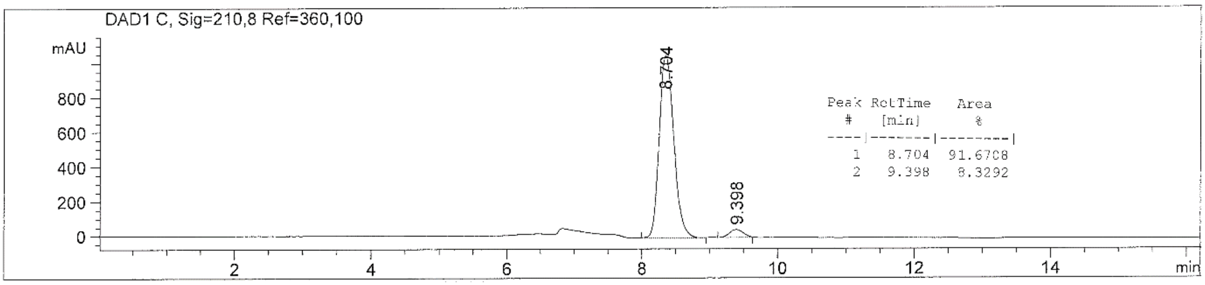

Figure S55. HPLC trace of enantioenriched product (83% (*R*) ee using **4c** catalytic system; Table 3, entry 22).

1-Benzyl-1,2,3,4-tetrahydronaphthalene (from S26).[22] Enantiomeric excess determined by HPLC using Chiracel OJ-H column (hexane/2-propanol=95/5, 0.5 mL/min, 226 nm). t_R 9.6 min (*S*); t_R 11.5 min (*R*). ^{1}H NMR ($CDCl_3$), δ: 1.55–1.63 (m, 3H), 1.78–1.83 (m, 1H), 2.64–2.73 (m, 3H), 3.03–3.06 (m, 2H), 7.03–7.07 (m, 3H), 7.14–7.19 (m, 4H), 7.22–7.24 (m, 2H).

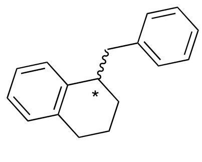

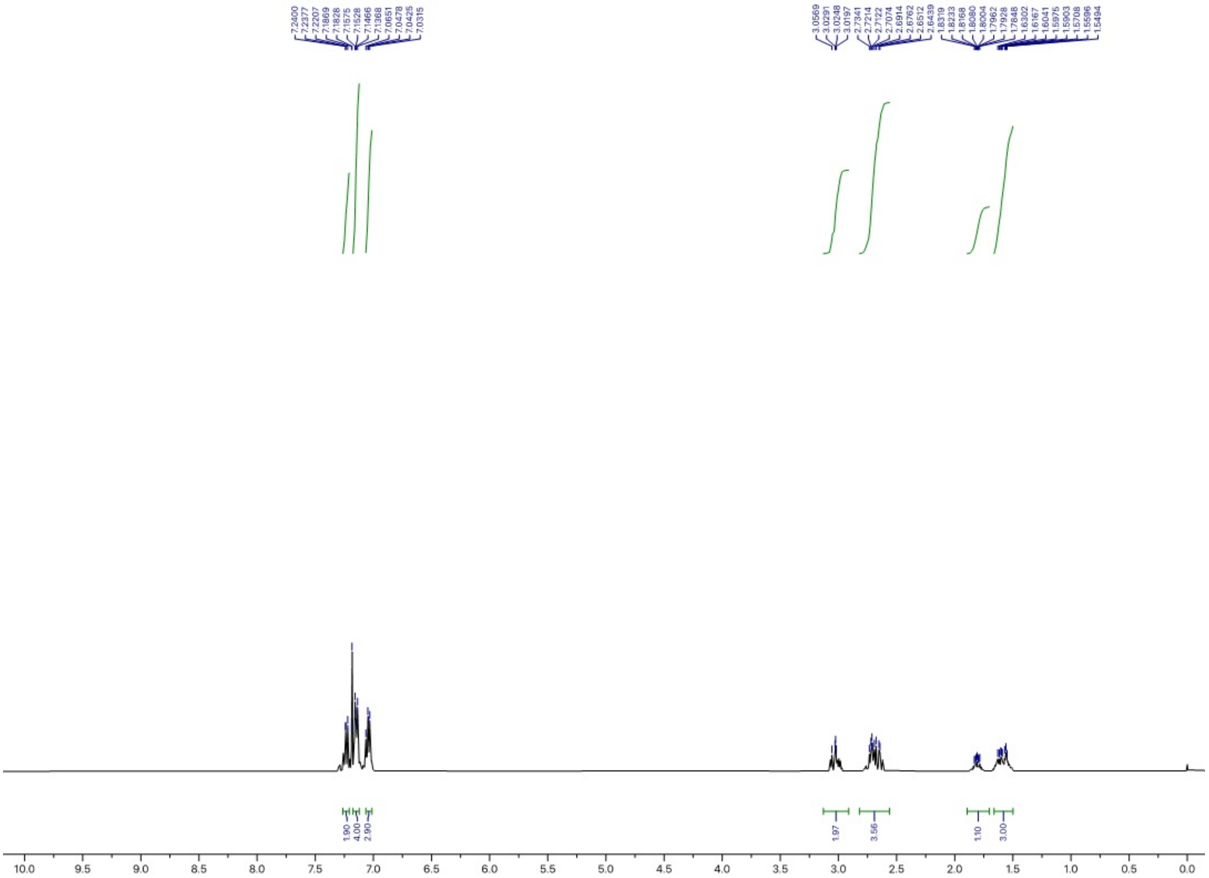

Figure S56. ^{1}H NMR spectrum of 1-benzyl-1,2,3,4-tetrahydronaphthalene (from **S26**) in $CDCl_3$.

(a)

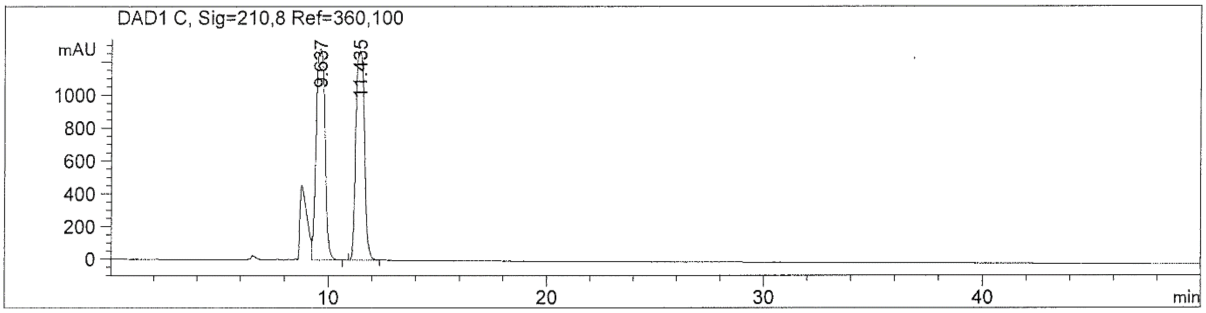

(b)

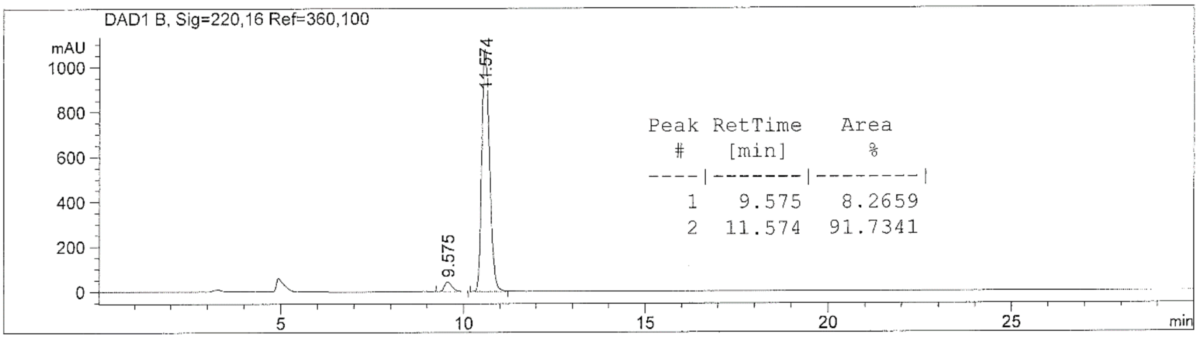

Figure S57. HPLC trace of: (a) racemic and (b) enantioenriched product (83% (*R*) ee using **4c** catalytic system; Table 3, entry 23).

1-(4-Methylbenzyl)-1,2,3,4-tetrahydronaphthalene (from S27).[23] Enantiomeric excess determined by HPLC using Chiralcel OJ-H column (98% 2-propanol/hexane, flow 0.5 ml/min, λ = 254 nm). t_R 14.1min (*S*); t_R 15.2 min (*R*). ^{1}H NMR ($CDCl_3$), δ: 1.49–1.64 (m, 3H), 1.78–1.81 (m, 1H), 2.27 (s, 3H), 2.60–2.73 (m, 3H), 2.95–3.03 (m, 2H), 7.02–7.18 (m, 8H).

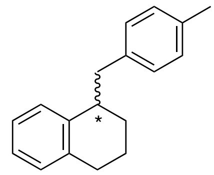

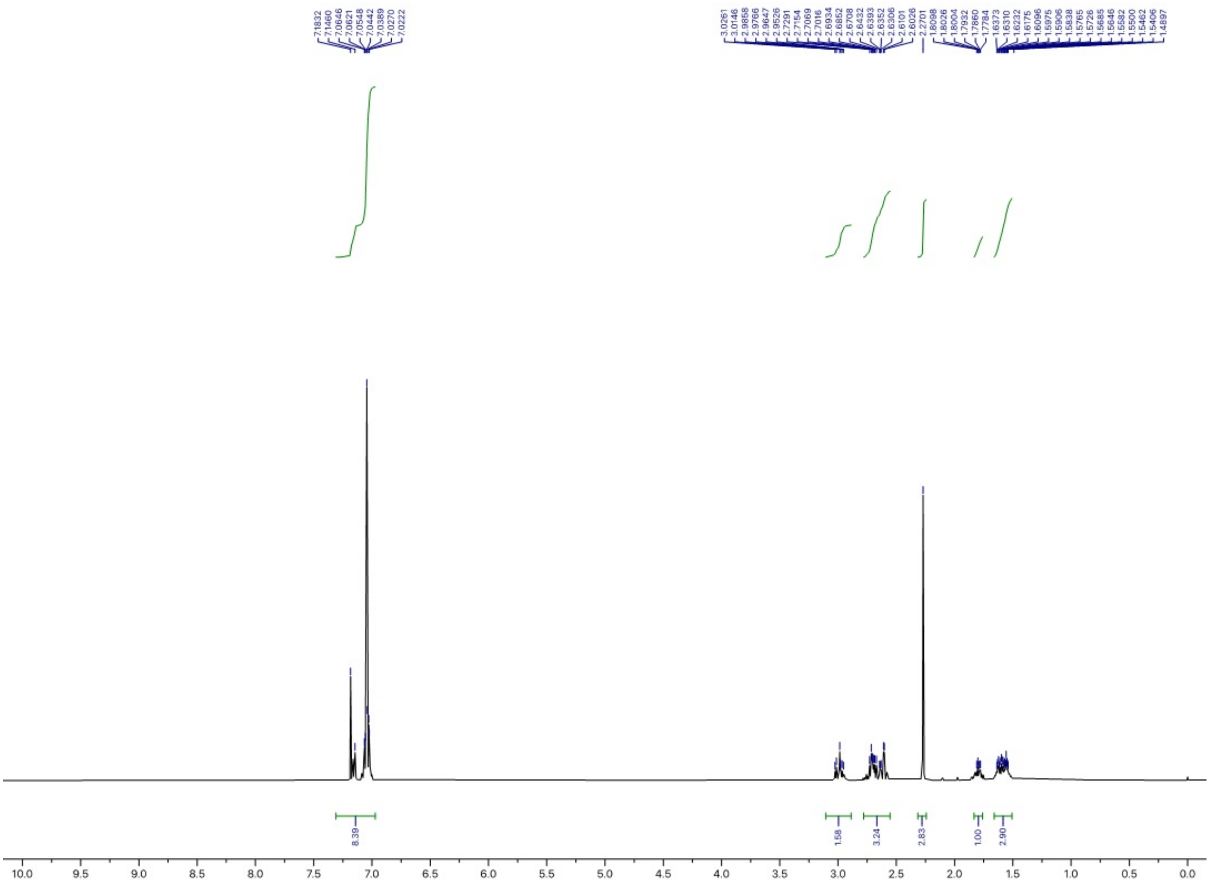

Figure S58. ^{1}H NMR spectrum of 1-(4-methylbenzyl)-1,2,3,4-tetrahydronaphthalene (from **S27**) in $CDCl_3$.

(a)

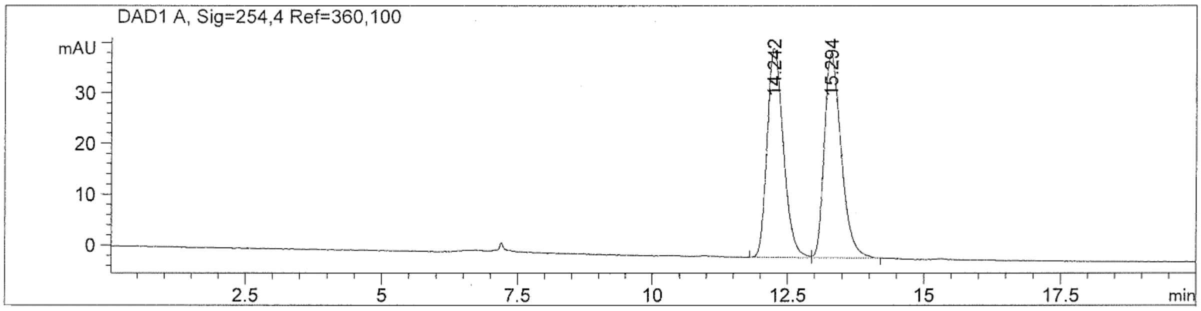

(b)

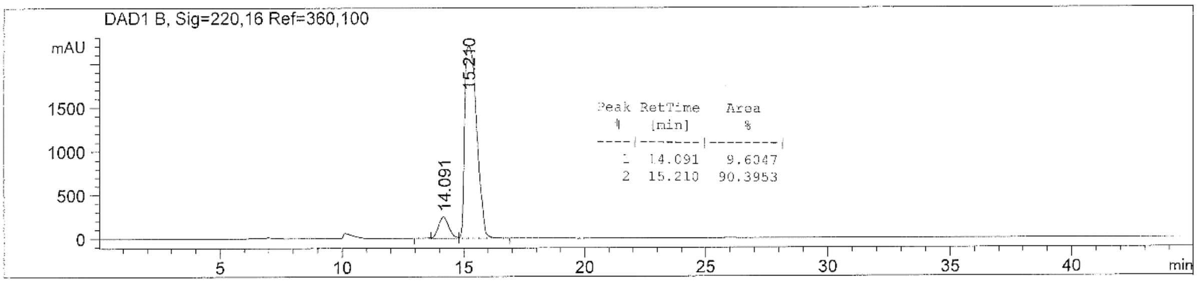

Figure S59. HPLC trace of: (a) racemic and (b) enantioenriched product (81% (*R*) ee using **4c** catalytic system; Table 3, entry 24).

1-(4-Chlorobenzyl)-1,2,3,4-tetrahydronaphthalene (from S28).[23] Enantiomeric excess determined by

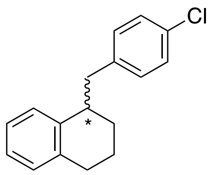

HPLC using Chiralcel OJ-H column (98% 2-propanol/hexane, flow 0.5 ml/min, λ = 220 nm). t_R 12.4 min (*S*); t_R13.9 min (*R*). ^{1}H NMR ($CDCl_3$), δ: 1.62–1.66 (m, 3H), 1.75–1.82 (m, 1H), 2.63–2.73 (m, 3H), 2.95–3.02 (m, 2H), 7.03–7.07 (m, 5H), 7.19–7.21 (m, 3H).

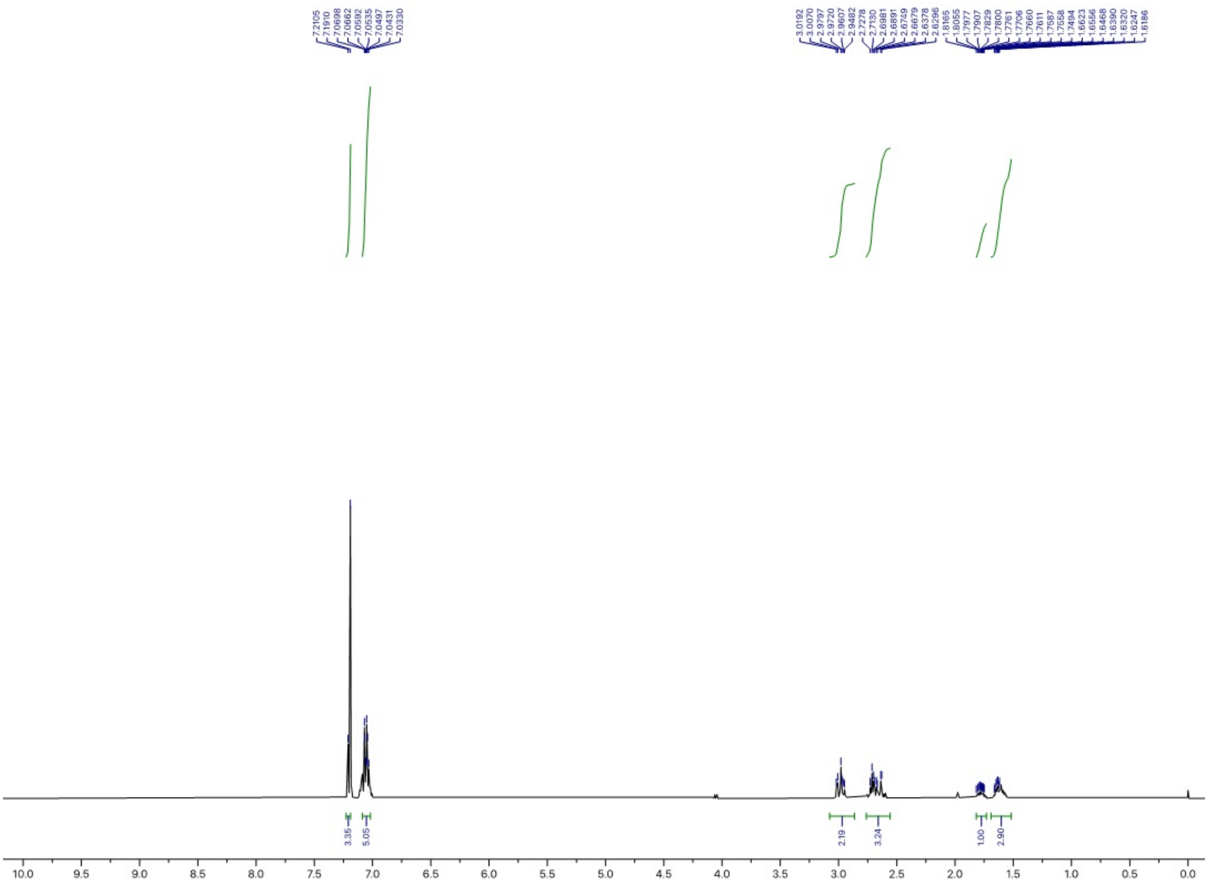

Figure S60. ^{1}H NMR spectrum of 1-(4-chlorobenzyl)-1,2,3,4-tetrahydronaphthalene (from **S28**) in $CDCl_3$.

(a)

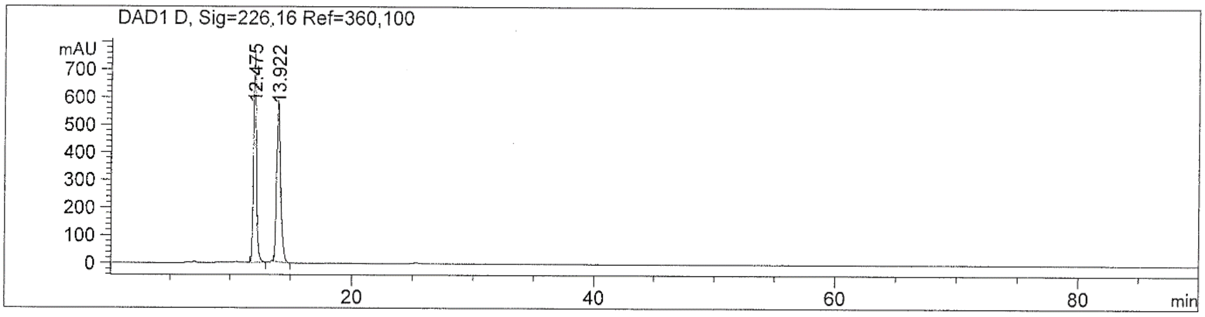

(b)

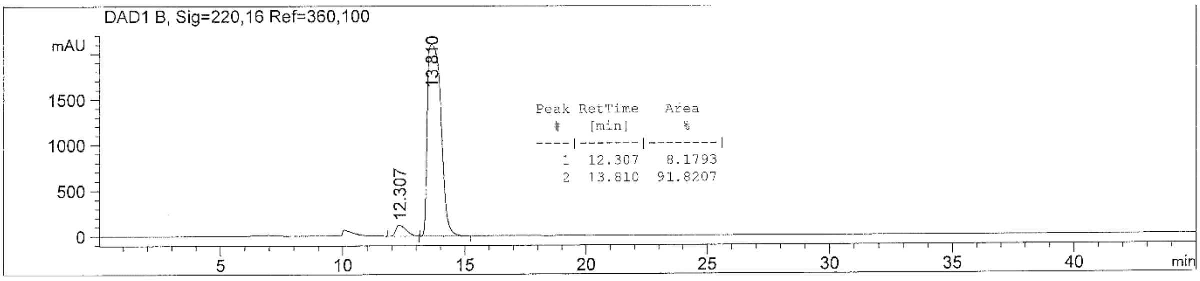

Figure S61. HPLC trace of: (a) racemic and (b) enantioenriched product (83% (*R*) ee using **4c** catalytic system; Table 3, entry 25).

1-(2-Methylbenzyl)-1,2,3,4-tetrahydronaphthalene (from S29).[23] Enantiomeric excess determined by

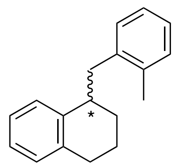

HPLC using Chiralcel OJ-H column (98% 2-propanol/hexane, flow 0.5 ml/min, λ = 226 nm). t_R 9.9 min (*S*); t_R 10.7 min (*R*). ^{1}H NMR ($CDCl_3$), δ: 1.66–1.76 (m, 3H), 1.89–1.99 (m, 1H), 2.36 (s, 3H), 2.75–2.83 (m, 3H), 3.08–3.11 (m, 2H), 7.11–7.17 (m, 8H).

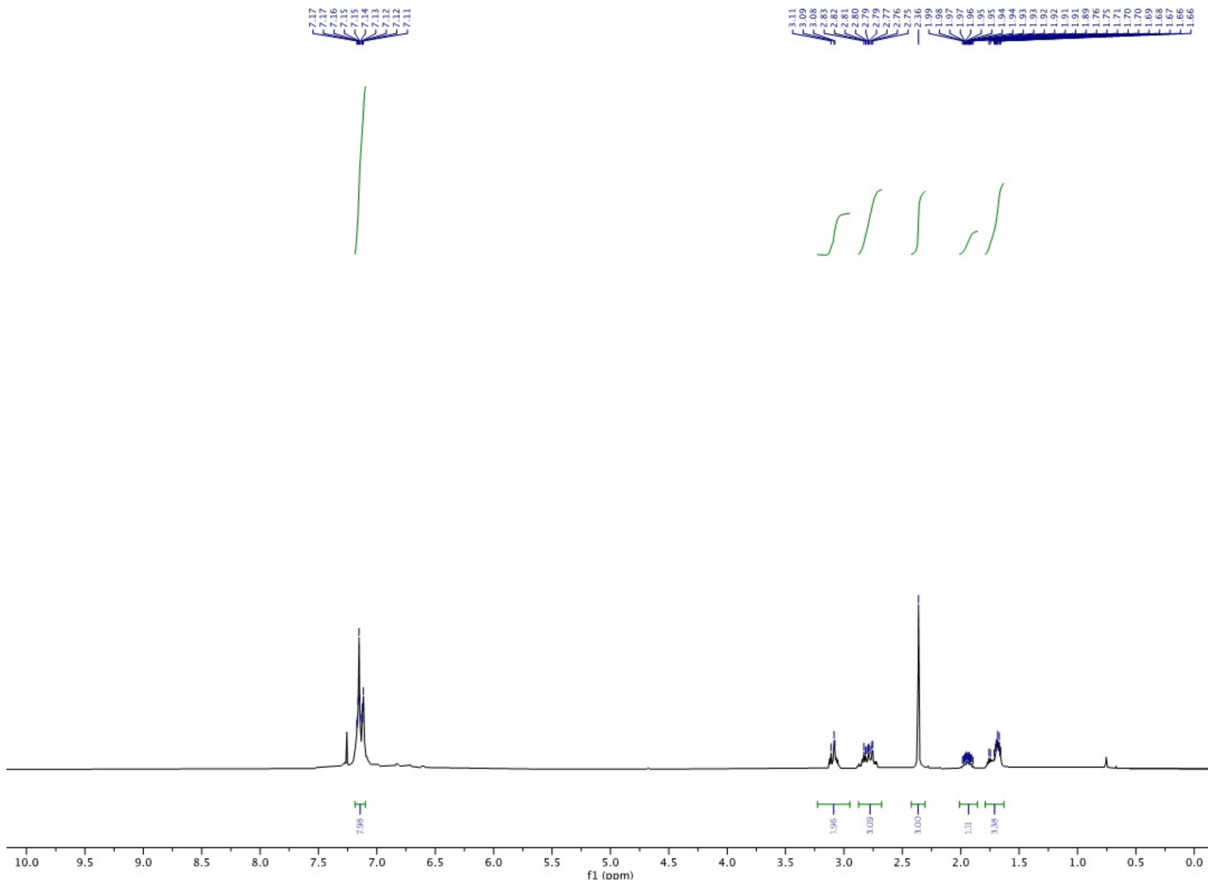

Figure S62. ^{1}H NMR spectrum of 1-(2-methylbenzyl)-1,2,3,4-tetrahydronaphthalene (from **S29**) in $CDCl_3$.

(a)

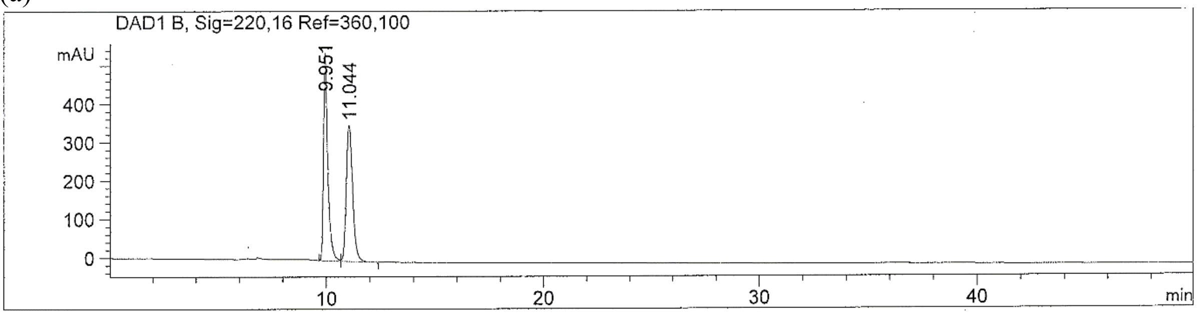

(b)

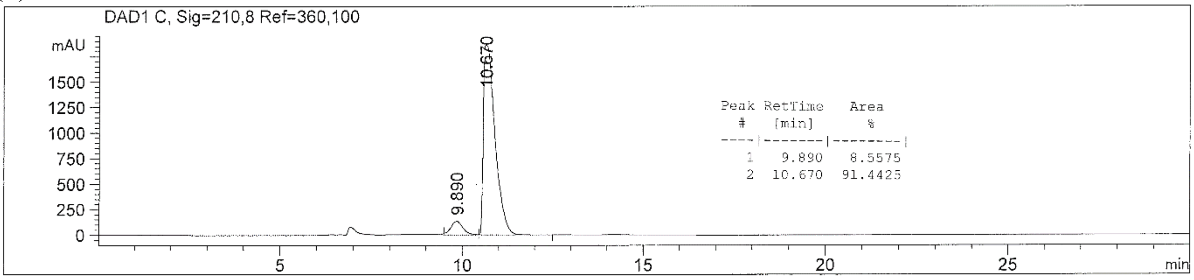

Figure S63. HPLC trace of: (a) racemic and (b) enantioenriched product (83% (*R*) ee using **4c** catalytic system; Table 3, entry 26).

1-Benzylbenzocyclobutene (from S30).[23] Enantiomeric excess determined by GC using Hydrodex β-

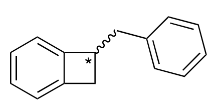

3P column (90 kPa H_2, 90 ºC, 15 min - 1 °C/min - 180 °C). t_R 63.6 min (*S*); t_R 64.0 min (*R*). ^{1}H NMR ($CDCl_3$), δ: 2.90 (dd, *J*= 14.2 Hz, *J*= 2.8 Hz, 1H), 2.99–3.02 (m, 2H), 3.34 (dd, *J*= 14.2 Hz, *J*= 5.1 Hz, 1H), 3.74-3.77 (m, 1H), 6.85–6.87 (m, 1H), 7.07–7.35 (m, 8H).

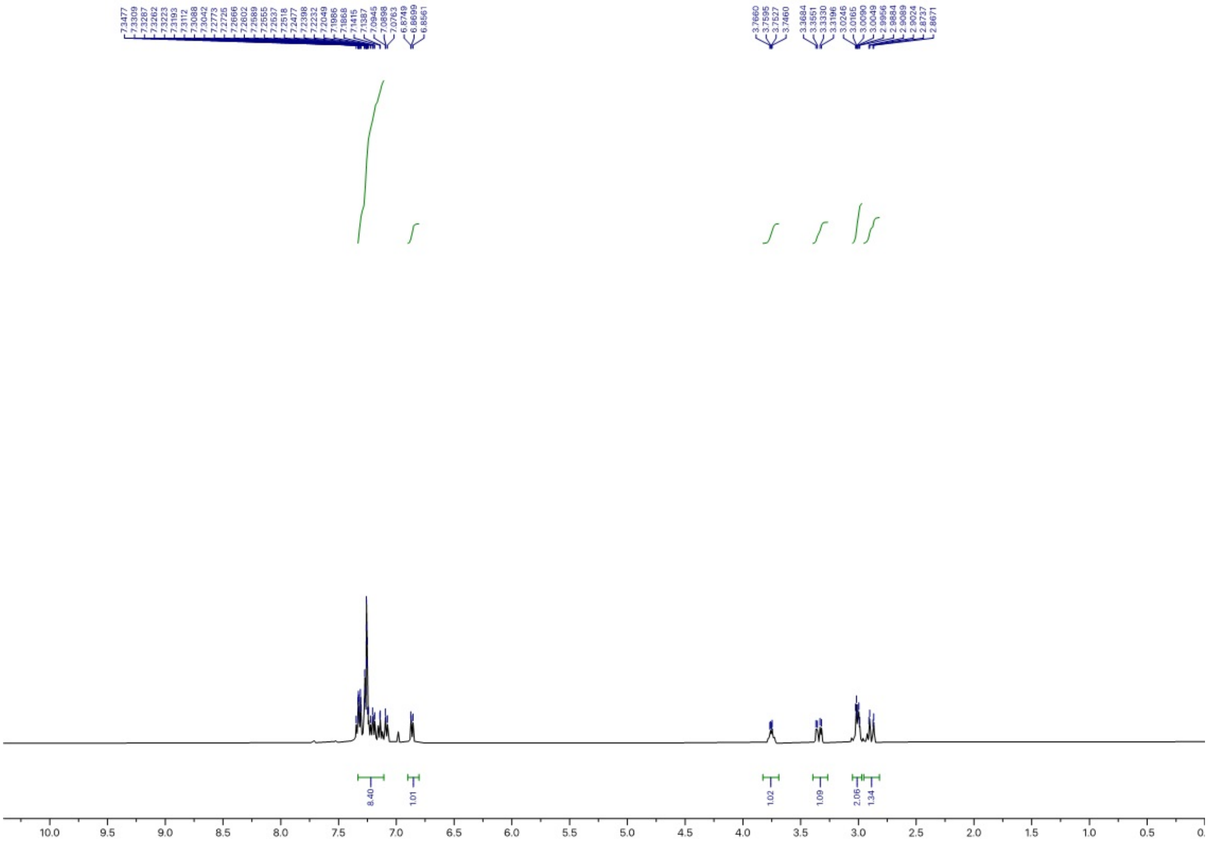

Figure S64. ^{1}H NMR spectrum of 1-benzylbenzocyclobutene (from **S30**) in $CDCl_3$.

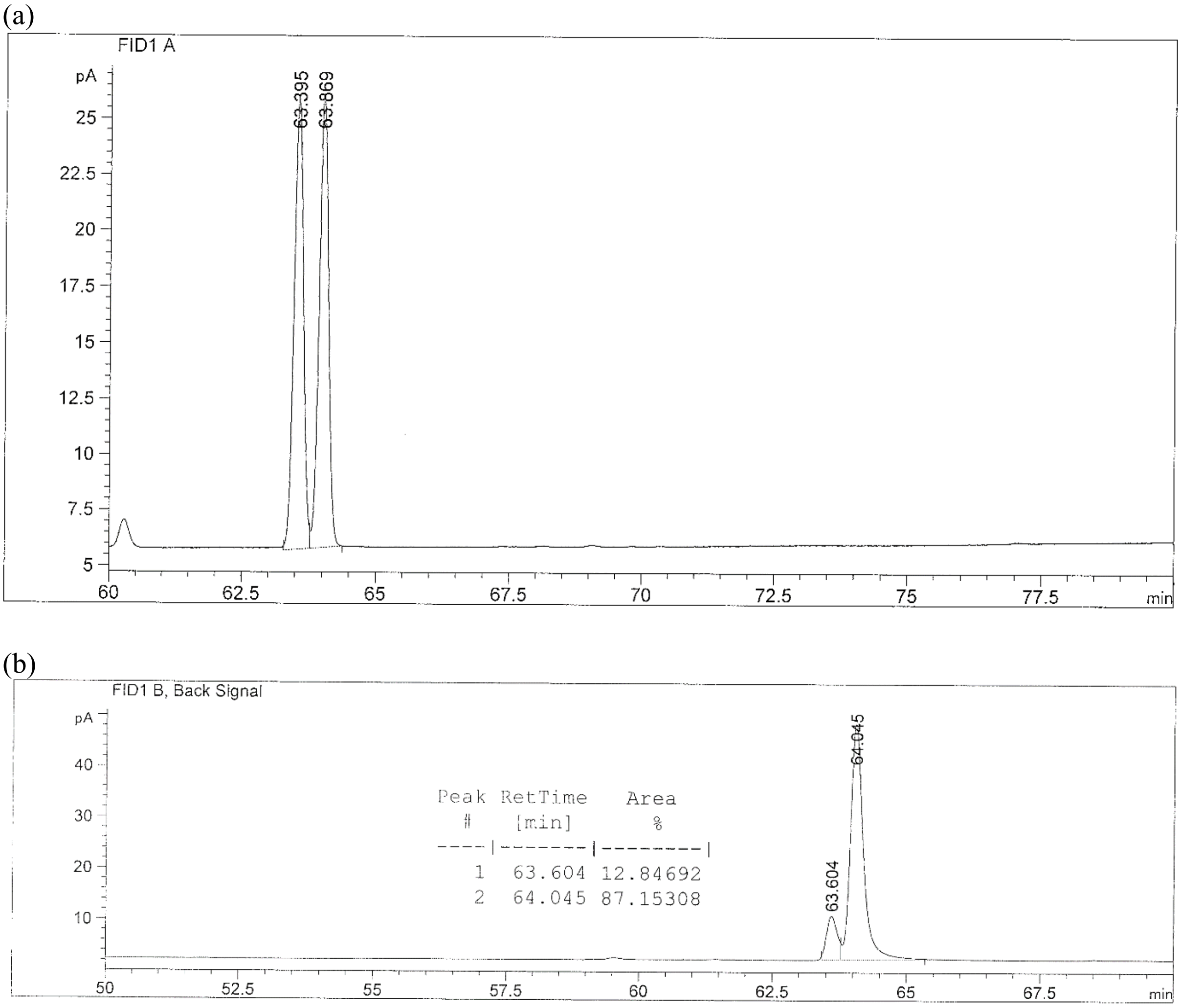

Figure S65. HPLC trace of enantioenriched product (74% (*R*) ee using **4c** catalytic system; Table 3, entry 27).

3-Methyl-4-phenylbutan-2-one (from S31).[25] Enantiomeric excess determined by HPLC using

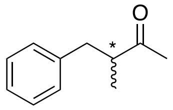

Chiracel OJ-H column (hexane/2-propanol=97/3, 1 mL/min, 220 nm). t_R 8.9 min (*S*); t_R 9.3 min (*R*). ^{1}H NMR ($CDCl_3$), δ: 1.10 (d, 3H, *J*= 6.9 Hz), 2.10 (s, 3H), 2.57 (dd, 1H, *J*= 13.5 Hz, *J*= 7.7 Hz), 2.82-2.87 (m, 1H), 3.01 (dd, 1H, *J*= 13.5 Hz, *J*= 6.8 Hz), 7.15-7.17 (m, 3H), 7.21-7.31 (m, 2H).

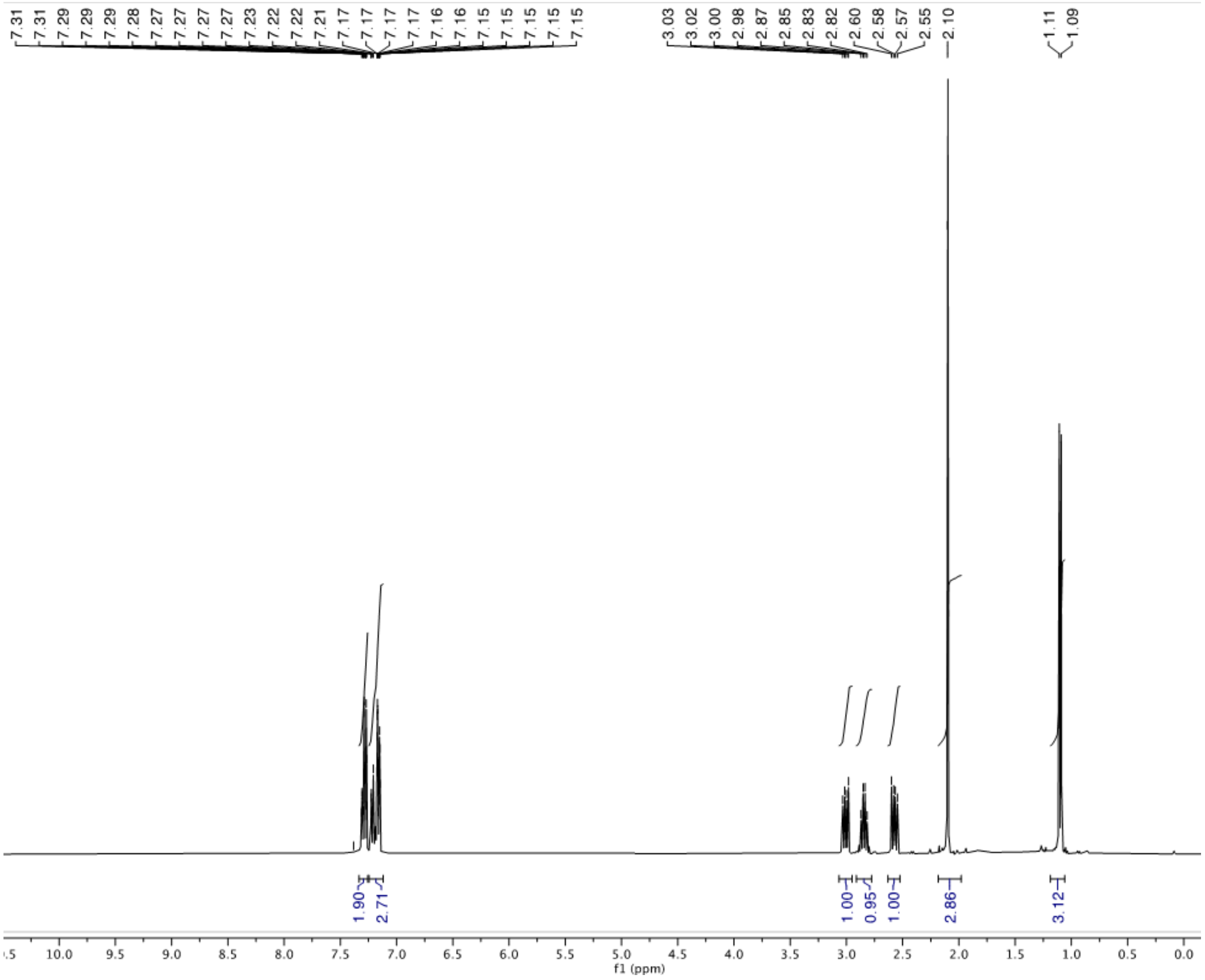

Figure S66. ^{1}H NMR spectrum of 3-methyl-4-phenylbutan-2-one (from **S31**) in $CDCl_3$.

(a)

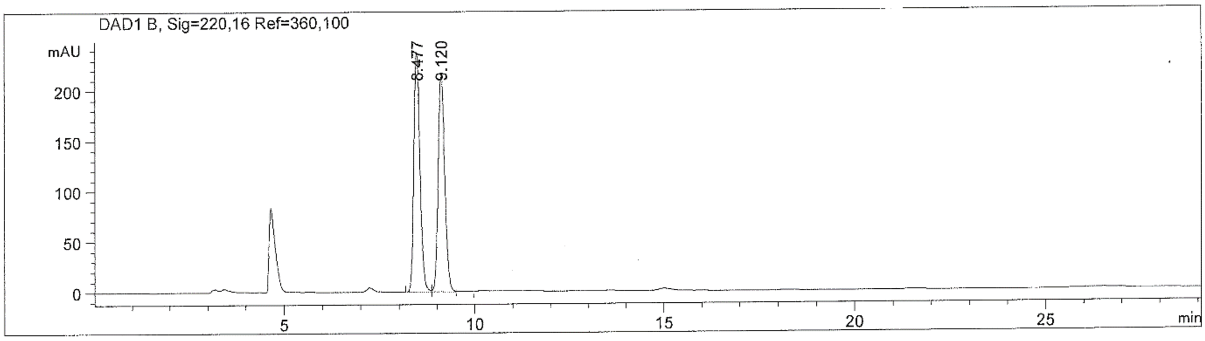

(b)

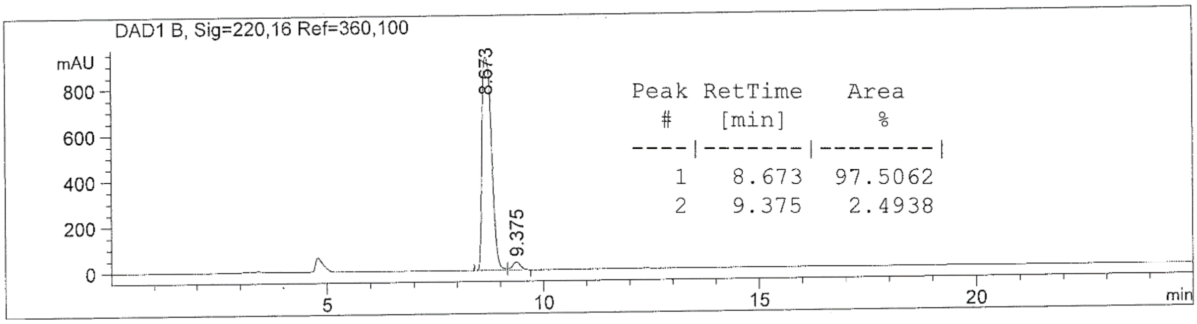

Peak #	RetTime [min]	Area %
1	8.673	97.5062
2	9.375	2.4938

Figure S67. HPLC trace of (a) racemic and (b) enantioenriched product (95% (*S*) ee using **4a** catalytic system; Scheme 2).

4-(4-Methoxyphenyl)-3-methylbutan-2-one (from S32).[44] Enantiomeric excess determined by HPLC using Chiracel OJ-H column (hexane/2-propanol=95/5, 0.5 mL/min, 220 nm). t_R 27.4 min (*S*); t_R 29.8 min (*R*). ^{1}H NMR ($CDCl_3$), δ: 1.07 (d, 3H, *J*= 6.9 Hz), 2.08 (s, 3H), 2.52 (dd, 1H, *J*= 7.5 Hz, *J*= 13.6 Hz), 2.78-2.81 (m, 1H), 2.92 (dd, 1H, *J*= 6.9 Hz, *J*= 13.6 Hz), 3.78 (s, 3H), 6.81 (d, 2H, *J*= 8.6 Hz), 7.05-7.07 (m, 2H).

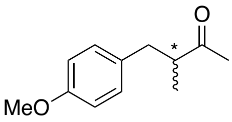

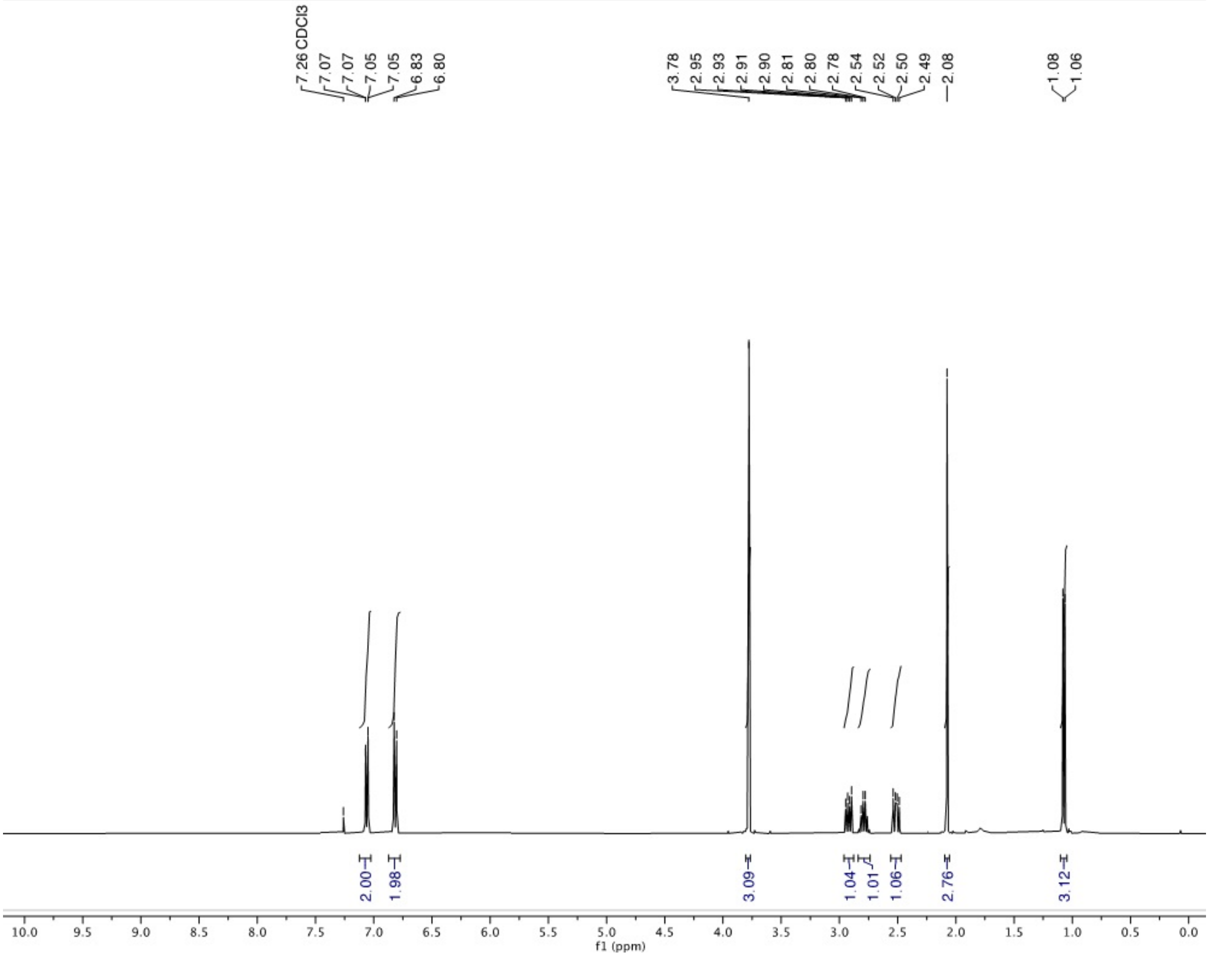

Figure S68. ^{1}H NMR spectrum of 4-(4-methoxyphenyl)-3-methylbutan-2-one (from **S32**) in $CDCl_3$.

(a)

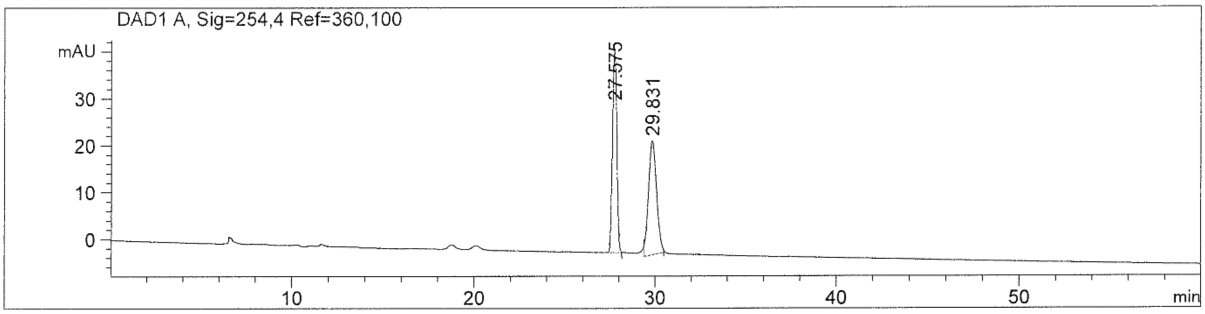

(b)

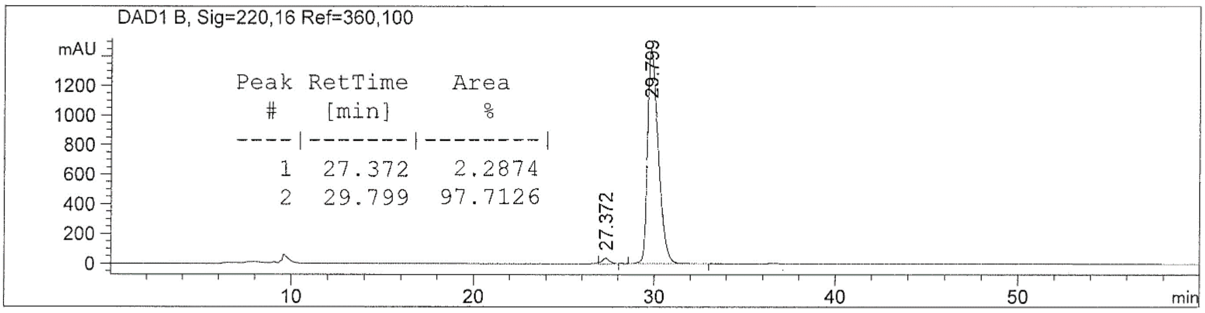

Figure S69. HPLC trace of enantioenriched product (95% (*S*) ee using **4a** catalytic system; Scheme 2).

2-Methyl-1-phenylpentan-3-one (from S33).[44] Enantiomeric excess determined by HPLC using

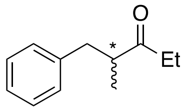

Chiracel OJ-H column (hexane/2-propanol=99/1, 0.5 mL/min, 220 nm). t_R 16.5 min (*S*); t_R 18.7 min (*R*). ^{1}H NMR ($CDCl_3$), δ: 0.97 (t, 3H, *J*= 7.2 Hz), 1.09 (d, 3H, *J*= 6.8 Hz), 2.23-2.31 (m, 1H), 2.39-2.47 (m, 1H), 2.57 (dd, 1H, *J*= 7.3 Hz, *J*= 13.4 Hz), 2.82-2.88 (m, 1H), 2.98 (dd, 1H, *J*= 7.2 Hz, *J*= 13.3 Hz), 7.13-7.22 (m, 3H), 7.25-7.30 (m, 2H).

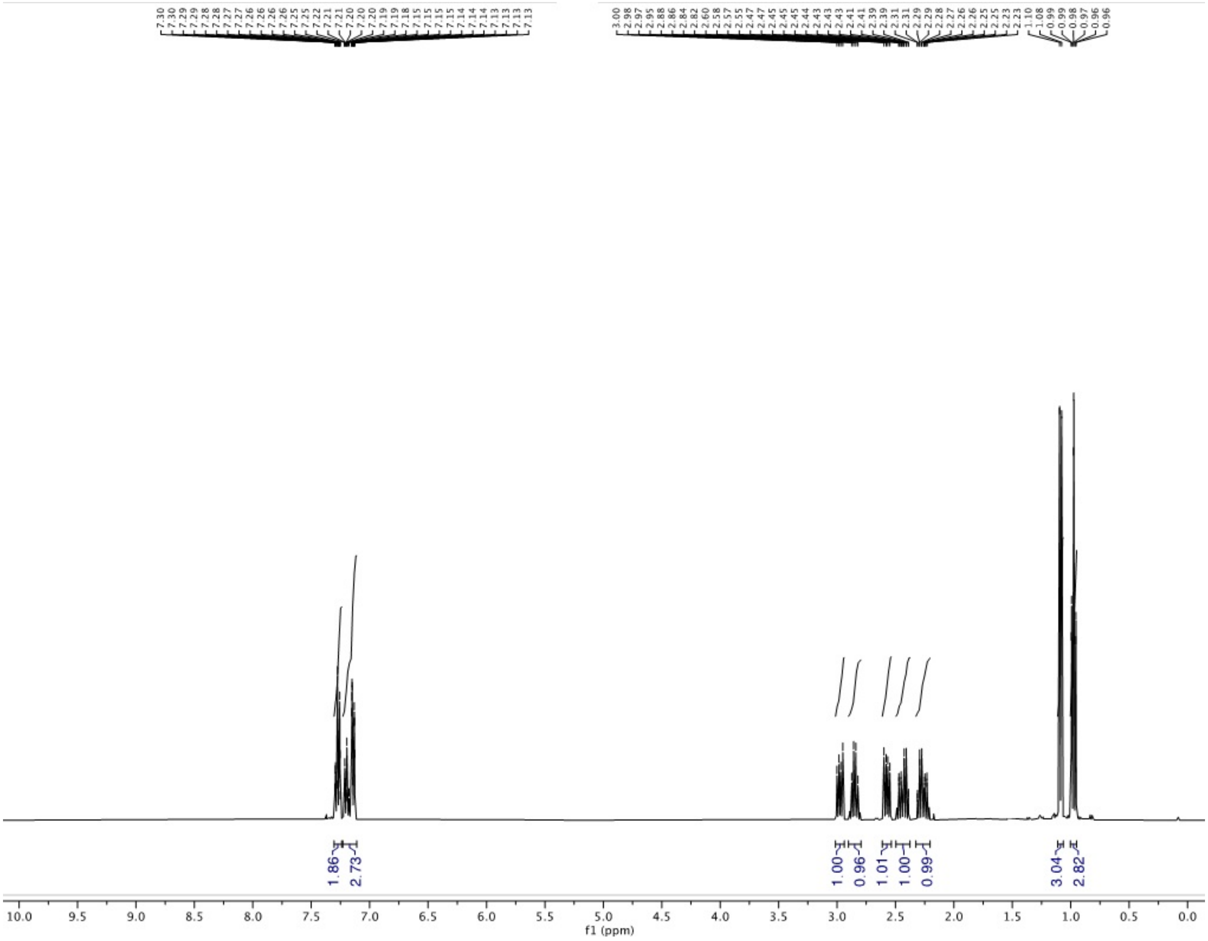

Figure S70. ^{1}H NMR spectrum of 2-methyl-1-phenylpentan-3-one (from **S33**) in $CDCl_3$.

(a)

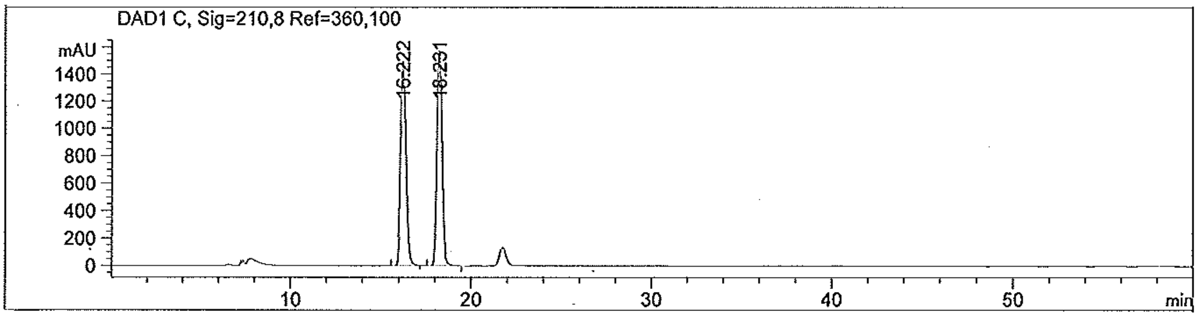

(b)

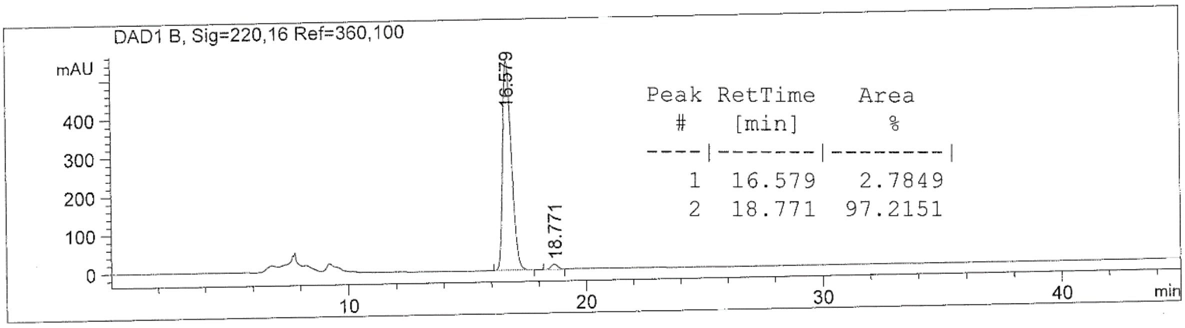

Figure S71. HPLC trace of enantioenriched product (94% (*S*) ee using **4a** catalytic system; Scheme 2).

2,4-Dimethyl-1-phenylpentan-3-one (from S34).[25] Enantiomeric excess determined by HPLC using

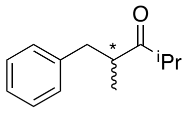

Chiracel OJ-H column (hexane/2-propanol=99/1, 0.5 mL/min, 220 nm). t_R 25.8 (*S*); t_R 27.2 min (*R*). ^{1}H NMR ($CDCl_3$), δ: 0.87 (d, 3H, *J*= 6.9 Hz), 1.01 (d, 3H, *J*= 6.9 Hz), 1.08 (d, 1H, *J*= 6.9 Hz), 2.48-2.57 (m, 2H), 2.92-3.02 (m, 2H), 7.12-7.20 (m, 3H), 7.23-7.28 (m, 2H).

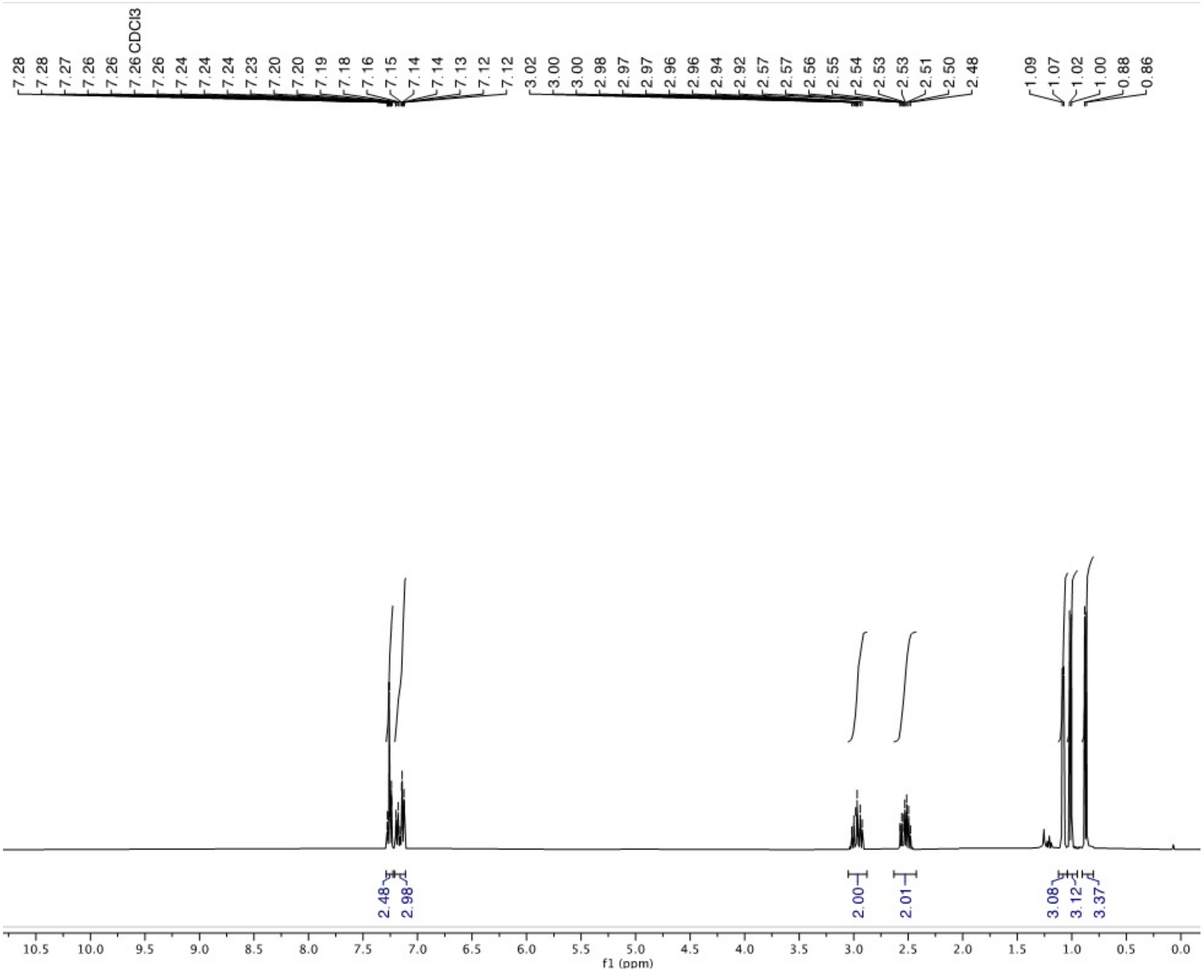

Figure S72. ^{1}H NMR spectrum of 2,4-dimethyl-1-phenylpentan-3-one (from **S34**) in $CDCl_3$.

(a)

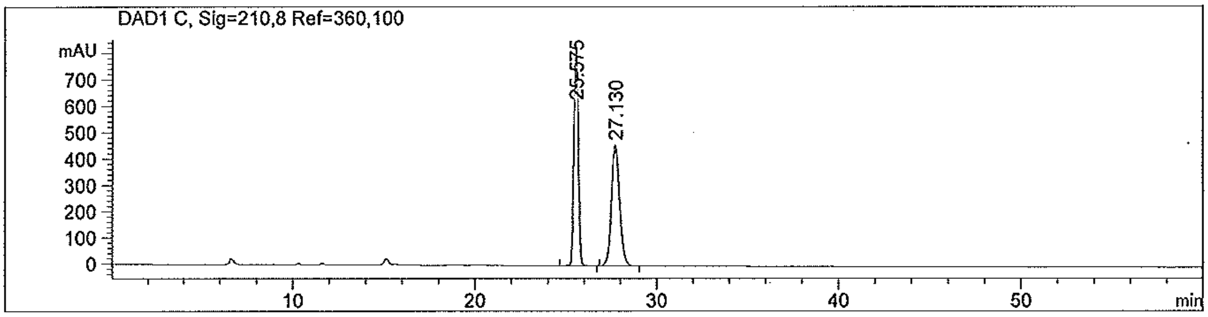

b)

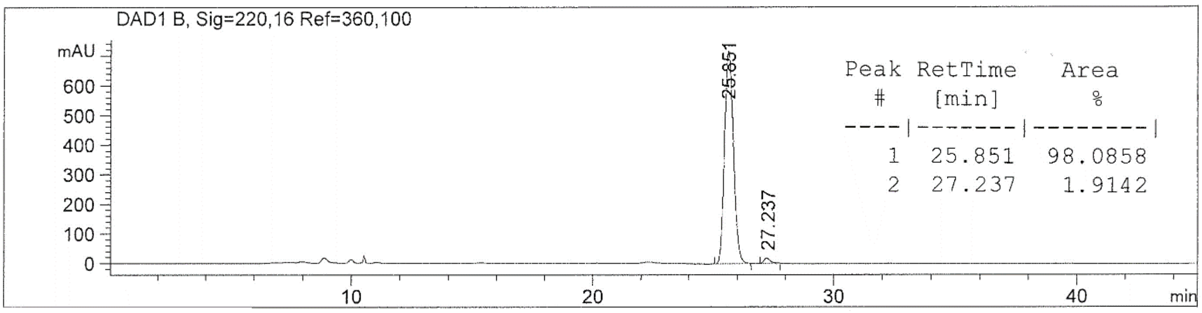

Figure S73. HPLC trace of enantioenriched product (96% (*S*) ee using **4a** catalytic system; Scheme 2).

2-Methyl-1,3-diphenylpropan-1-one (from S35).[25] Enantiomeric excess determined by HPLC using

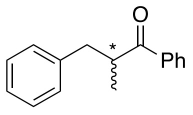

Chiracel OB column (hexane/2-propanol=98.2, 0.5 mL/min, 220 nm). t_R 16.9 min (*S*); t_R 18.1 min (*R*). ^{1}H NMR ($CDCl_3$), δ: 1.21 (d, 3H, *J*= 6.8 Hz), 2.70 (dd, 1H, *J*= 13.7 Hz, *J*= 7.8 Hz), 3.17 (dd, 1H, *J*= 13.7 Hz, *J*= 6.3 Hz), 3.74-3.79 (m, 1H), 7.18-7.29 (m, 5H), 7.43-7.47 (m, 2H), 7.53-7.57 (m, 1H), 7.92-7.94 (m, 2H).

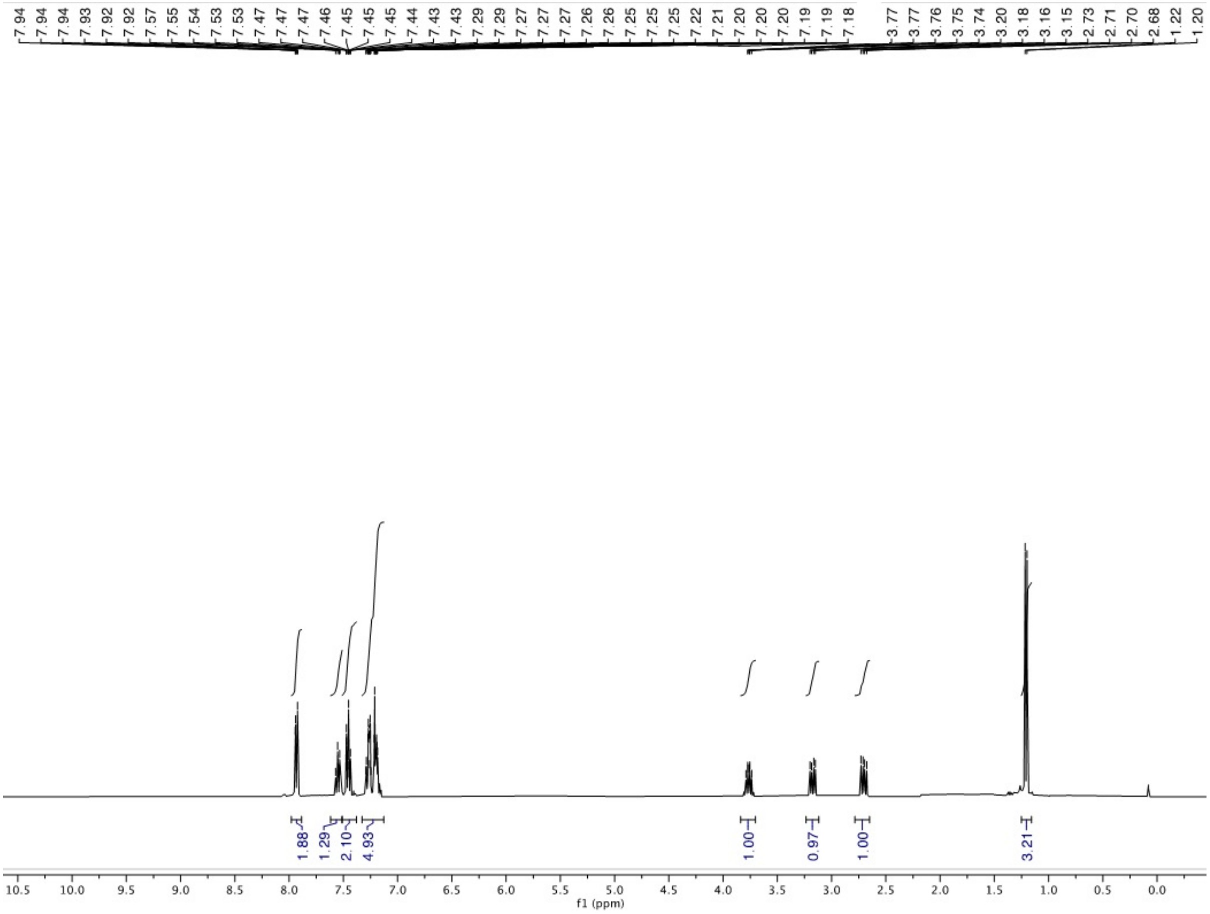

Figure S74. ^{1}H NMR spectrum of 2-methyl-1,3-diphenylpropan-1-one (from **S35**) in $CDCl_3$.

(a)

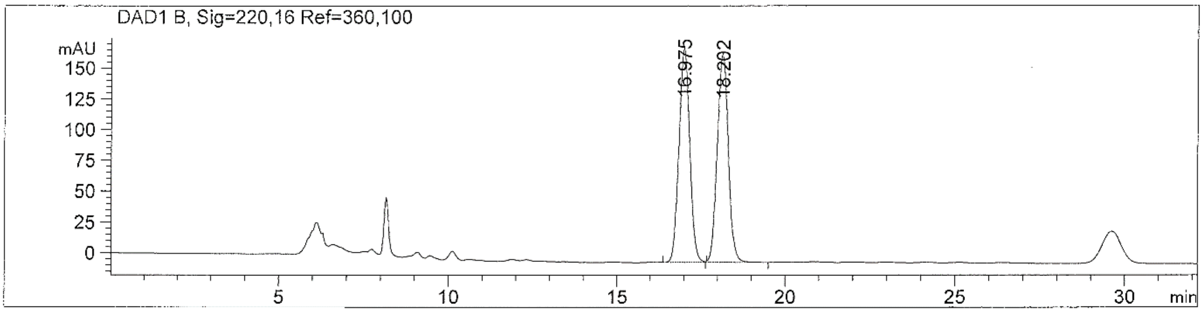

(b)

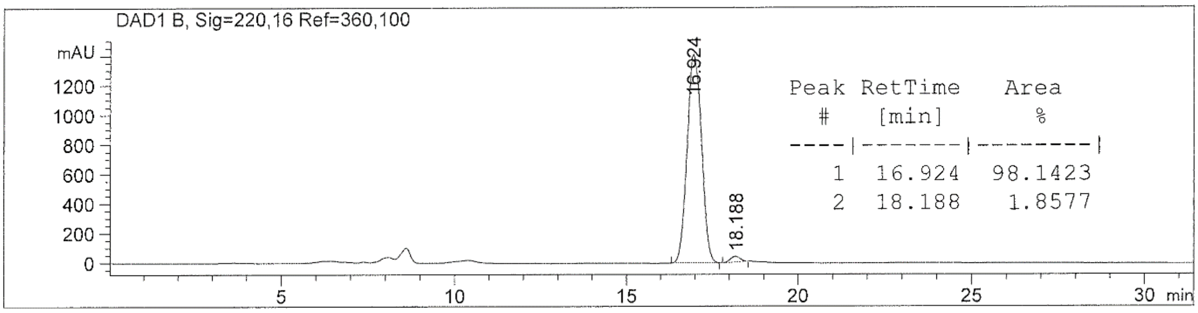

Figure S75. HPLC trace of (a) racemic and (b) enantioenriched product (96% (*S*) ee using **4a** catalytic system; Scheme 2).

2-Fluoro-1,3-diphenylpropan-1-one (from S36).[26] Enantiomeric excess determined by HPLC using

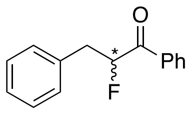

Chiracel OJ-H column (hexane/2-propanol=90.10, 0.7 mL/min, 220 nm). t_R 22.2 min (*R*); t_R 31.0 min (*S*). ^{1}H NMR ($CDCl_3$), δ: 3.22-3.39 (m, 2H), 5.74 (ddd, 1H, *J*= 49.0 Hz, *J*= 8.2 Hz, *J*= 4.0 Hz), 7.25-7.34 (m, 5H), 7.46-7.49 (m, 2H), 7.58-7.62 (m, 1H), 7.92-7.95 (m, 2H).

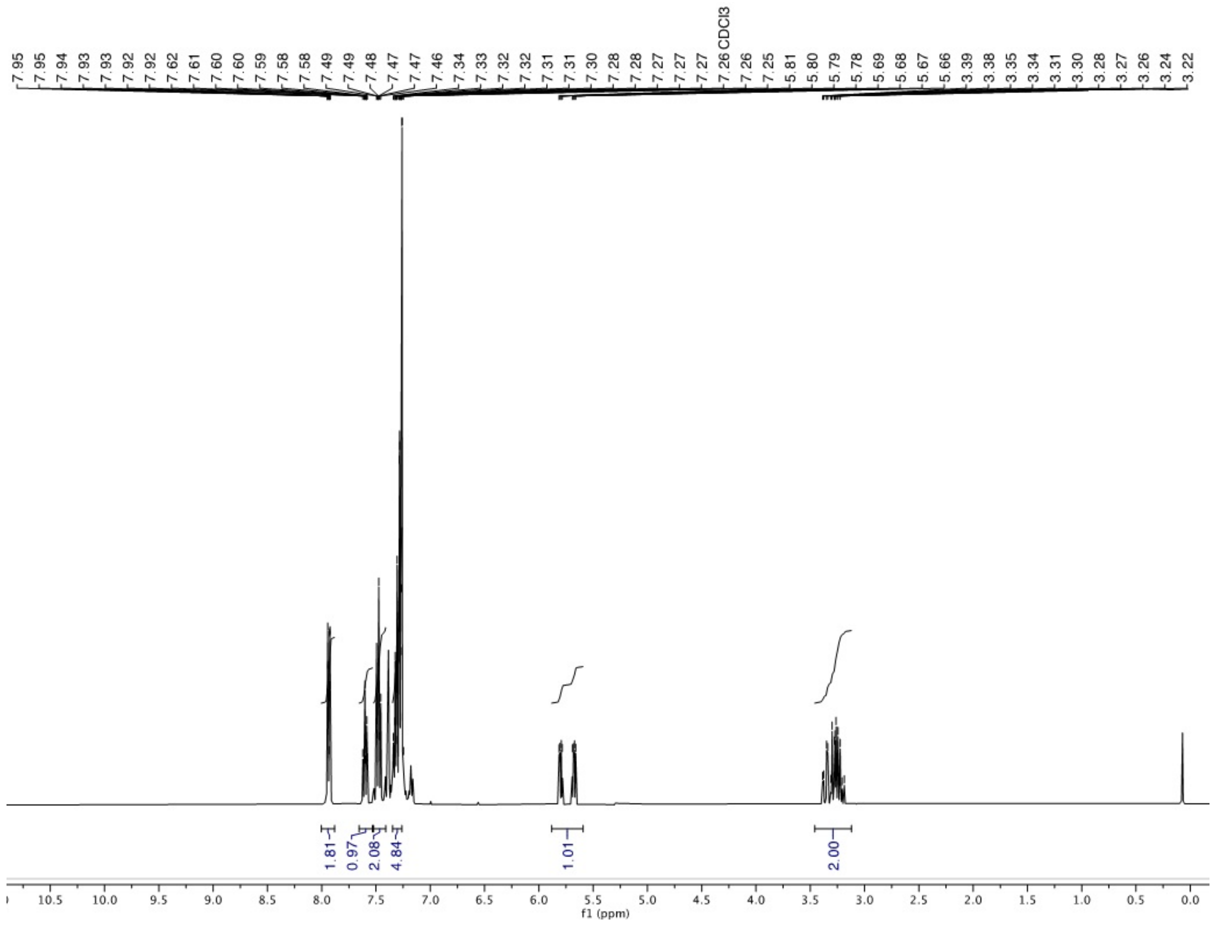

Figure S76. ^{1}H NMR spectrum of 2-fluoro-1,3-diphenylpropan-1-one (from **S36**) in $CDCl_3$.

(a)

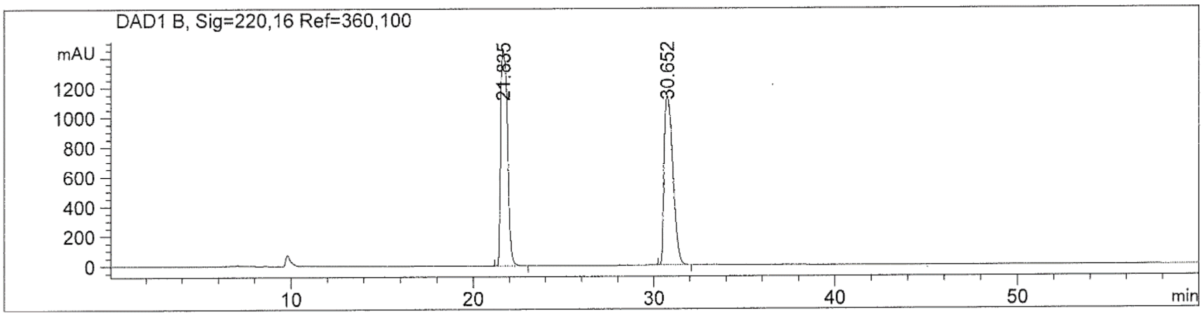

(b)

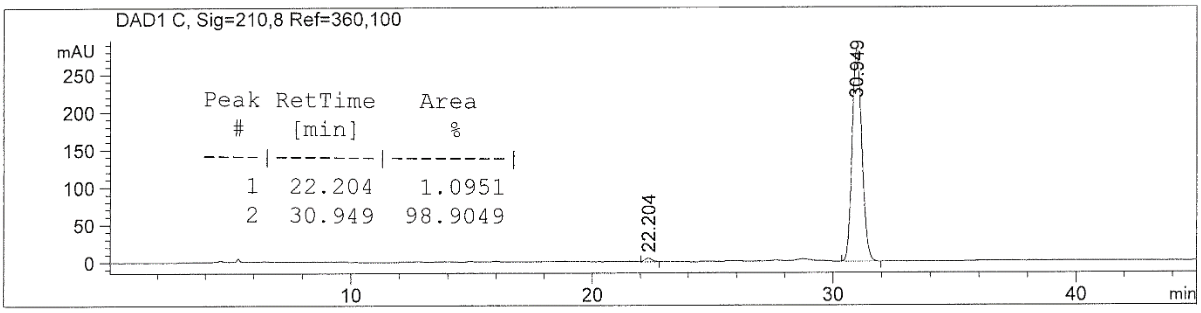

Figure S77. HPLC trace of: (a) racemic and (b) enantioenriched product (98% (*S*) ee using **4a** catalytic system; Scheme 2).

1,3-Diphenylbutan-1-one (from S37).[27] Enantiomeric excess determined by HPLC using a Chiracel OJ-H column (hexane/2-propanol = 97/3, 0.5 mL/min, 220 nm). t_R 26.7 min (*R*); t_R 30.9 min (*S*). ^{1}H NMR ($CDCl_3$), δ: 1.27 (d, 3H, *J*= 6.7 Hz), 3.08-3.14 (m, 1H), 3.20-3.26 (m, 1H), 3.39-3.46 (m, 1H), 7.12–7.24 (m, 5H), 7.35-7.39 (m, 2H), 7.46-7.40 (m, 1H), 7.84-7.86 (m, 2H).

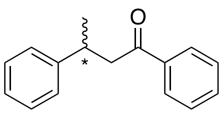

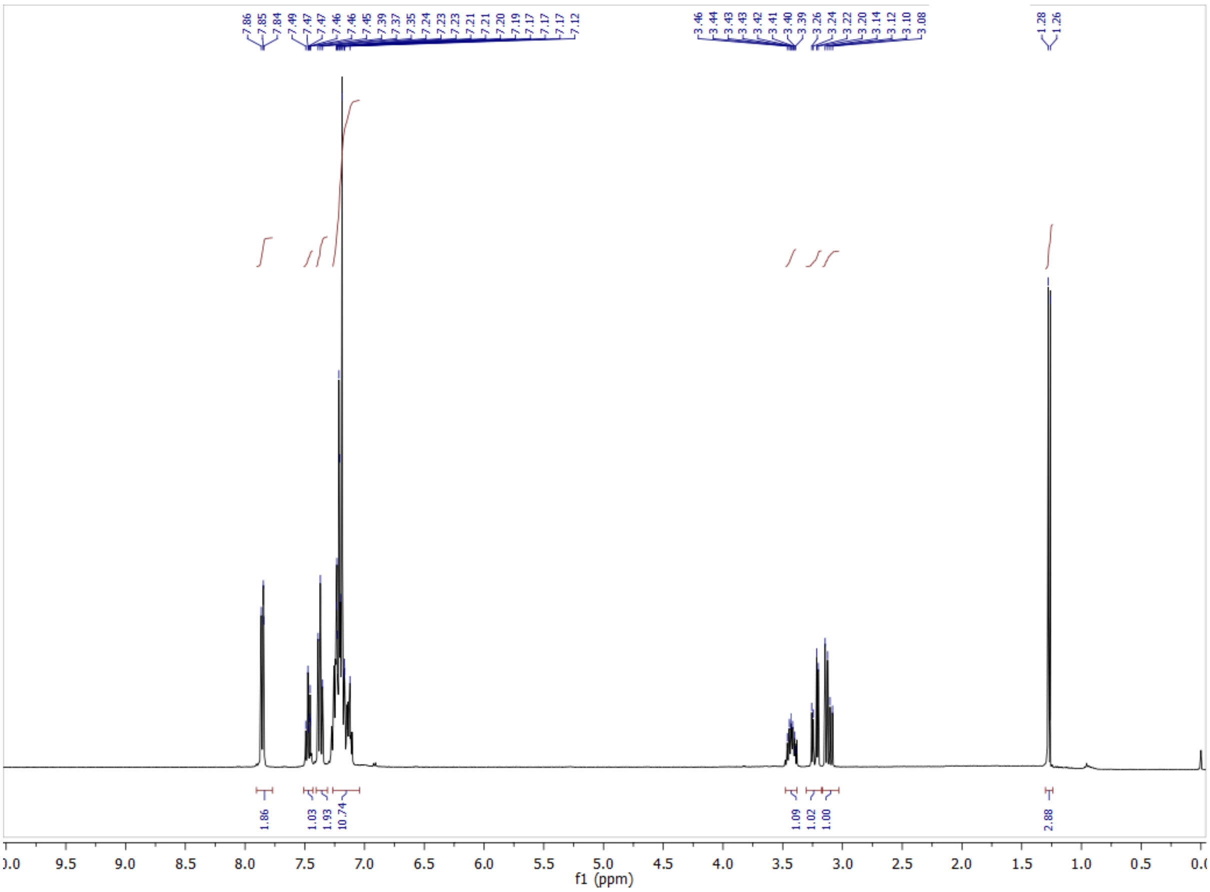

Figure S78. ^{1}H NMR spectrum of 1,3-diphenylbutan-1-one (from **S37**) in $CDCl_3$.

(a)

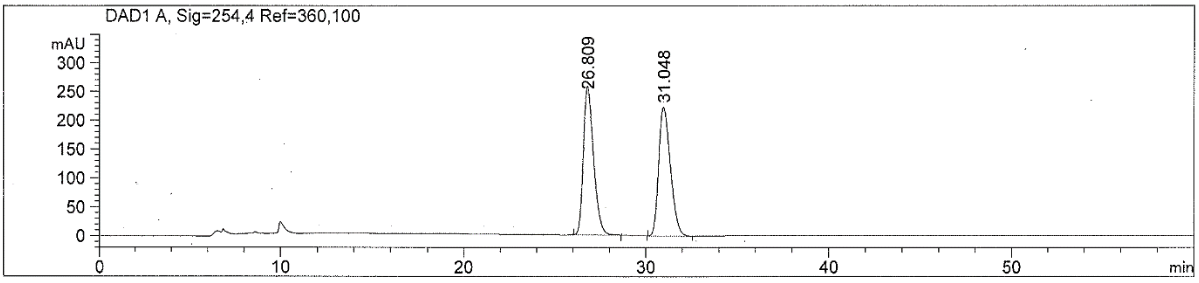

(b)

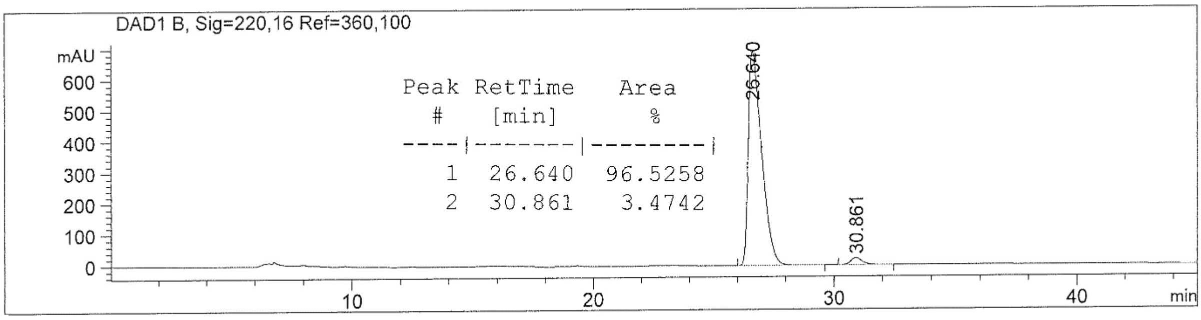

Figure S79. HPLC trace of: (a) racemic and (b) enantioenriched product (92% (*R*) ee using **4a** catalytic system; Scheme 2).

4-Methyl-1,3-diphenylpentan-1-one (from S38).[27] Enantiomeric excess determined by HPLC using a

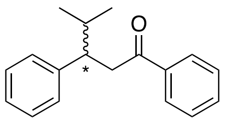

Chiracel AD column (hexane/2-propanol = 97/3, 0.5 mL/min, 220 nm). t_R 16.7 min (+); t_R 19.7 min (−). ^{1}H NMR ($CDCl_3$), δ: 0.78 (d, 3H, *J*= 6.9 Hz), 0.97 (d, 3H, *J*= 6.6 Hz), 1.91-1.97 (m, 1H), 3.15-3.19 (m, 1H), 3.35-3.37 (m, 2H), 7.15-7.26 (m, 5H), 7.40−7.44 (m, 2H), 7.50-7.52 (m, 1H), 7.86-7.88 (m, 2H).

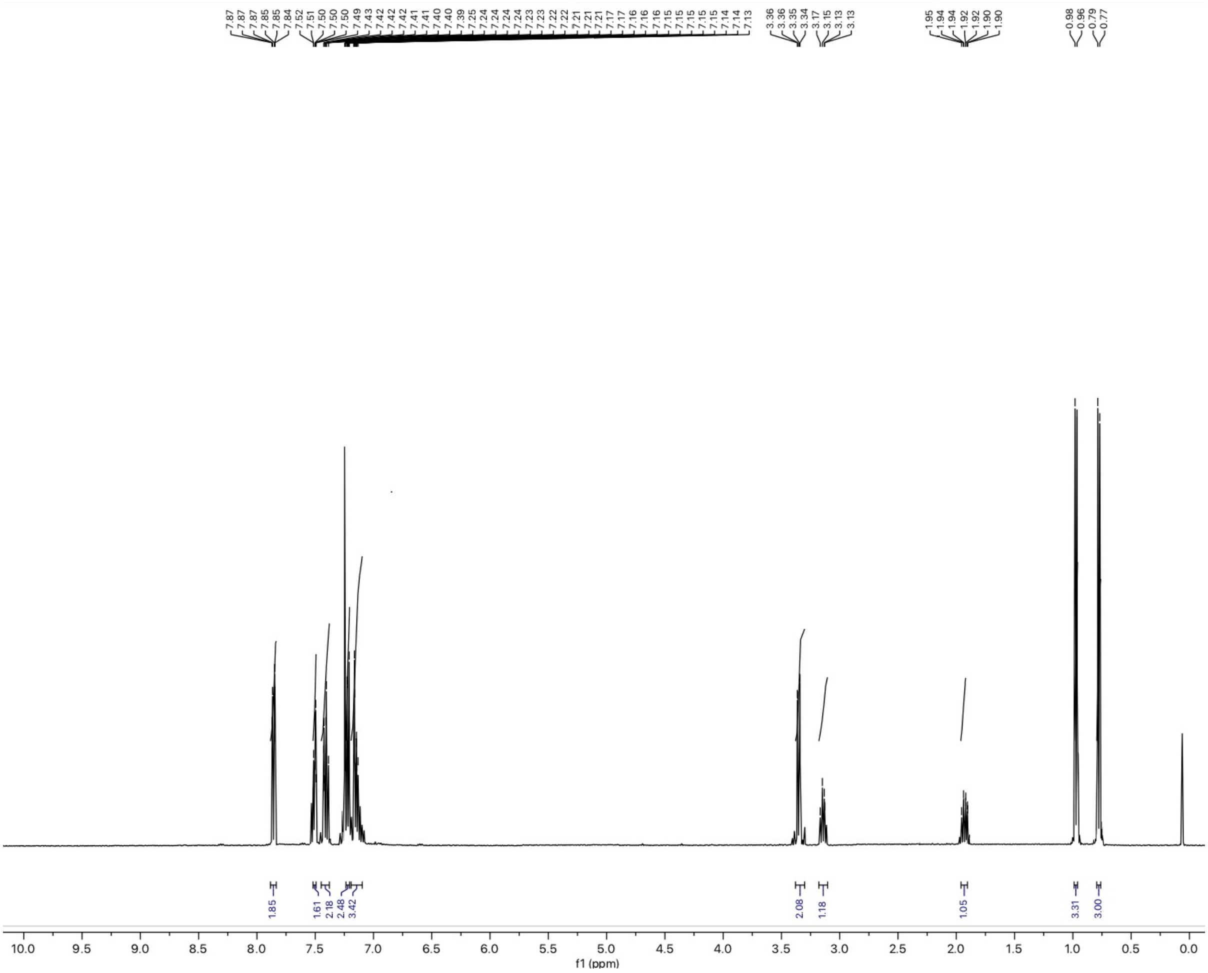

Figure S80. ^{1}H NMR spectrum of 4-methyl-1,3-diphenylpentan-1-one (from **S38**) in $CDCl_3$.

(a)

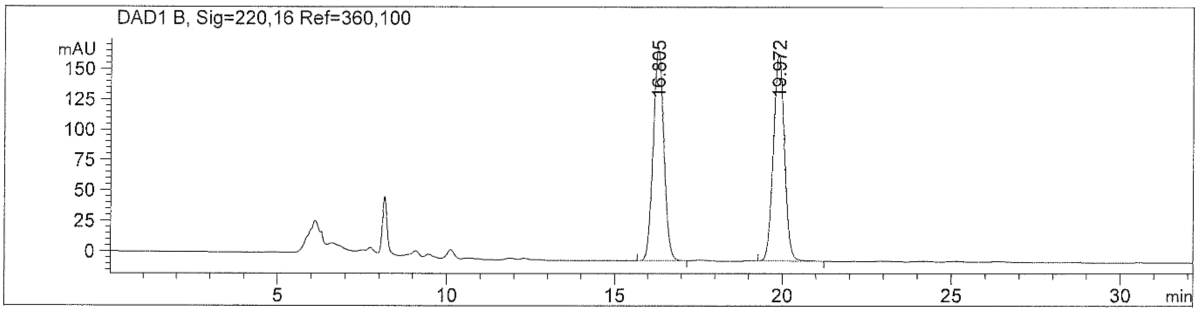

(b)

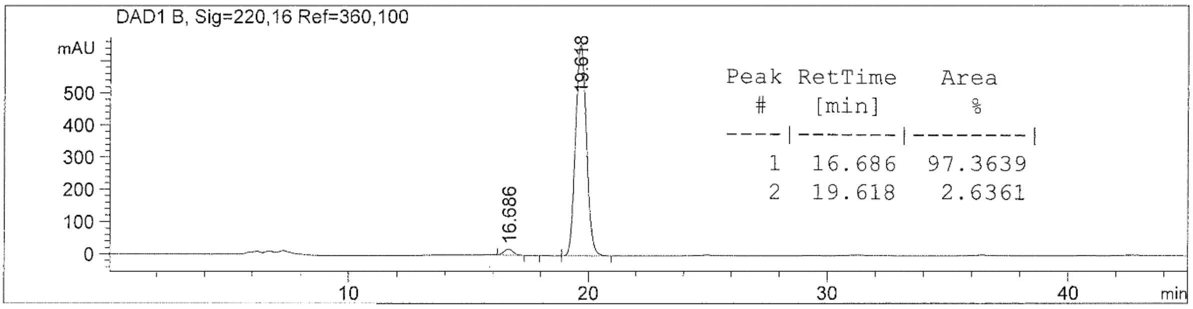

Figure S81. HPLC trace of: (a) racemic and (b) enantioenriched product (95% (*S*) ee using **4a** catalytic system; Scheme 2).

2-(1,2-Diphenylethyl)-4,4,5,5-tetramethyl-1,3,2-dioxaborolane (from S39).[28] Enantiomeric excess

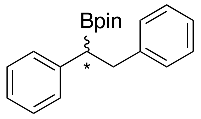

determined by HPLC using Chiracel OJ-H column (hexane/2- propanol=99/1, 0.5 mL/min, 254 nm). t_R 9.5 min (-); t_R 13.1 min (+). ^{1}H NMR ($CDCl_3$), δ: 1.12 (s, 6H), 1.13 (s, 6H), 2.71 (dd, 1H, *J*= 6.8 Hz, *J*= 10.0 Hz), 2.98 (dd, 1H, *J*= 7.0 Hz, *J*= 13.6 Hz), 3.17 (dd, 1H, *J*= 9.6 Hz, *J*= 13.6 Hz), 7.14-7.29 (m, 10H).

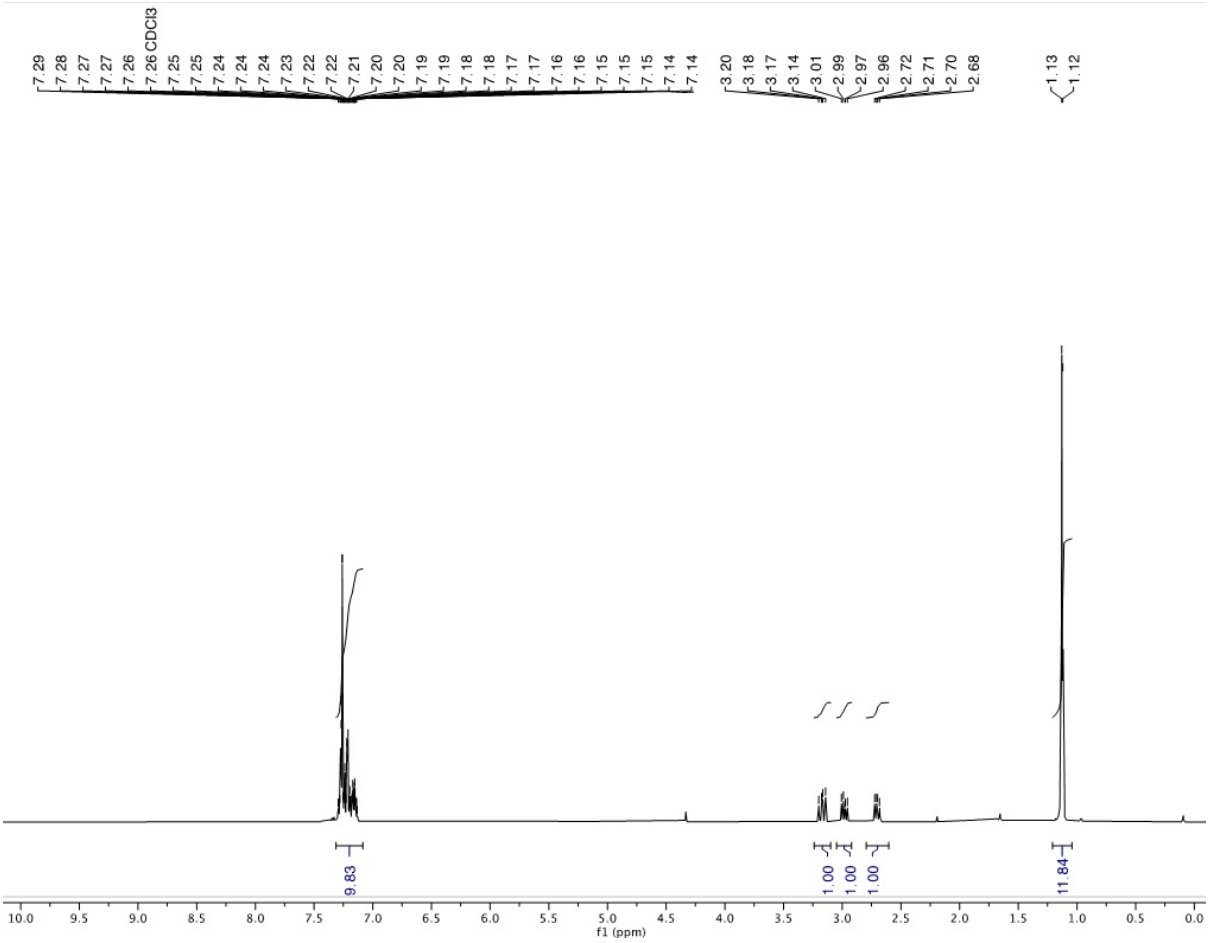

Figure S82. ^{1}H NMR spectrum of 2-(1,2-diphenylethyl)-4,4,5,5-tetramethyl-1,3,2-dioxaborolane (from **S39**) in $CDCl_3$.

(a)

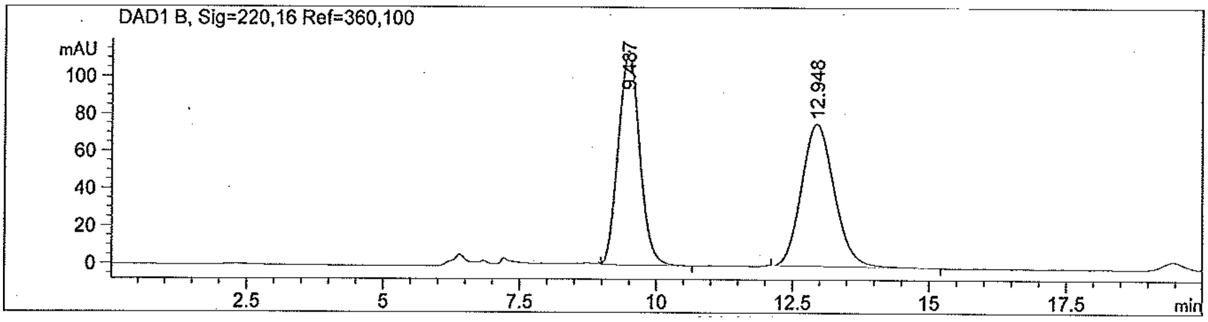

(b)

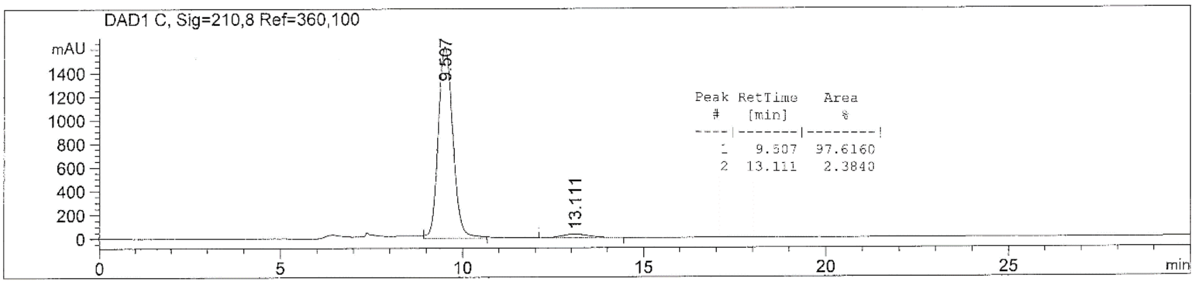

Figure S83. HPLC trace of: (a) racemic and (b) enantioenriched product (95% (-) ee using **4b** catalytic system; Scheme 3).

2-(1-Phenylethyl)-4,4,5,5-tetramethyl-1,3,2-dioxaborolane (from S40).[28] Enantiomeric excess were determined after oxidation of the pinacolborane derivative to the corresponding alcohol using sodium perborate monohydrate (75 mg, 0.75 mmol) in a THF/H_2O solution (1:1, 4 mL). Enantiomeric excess determined by GC using Chiralsil-Dex CB column (80 kPa H_2, 110 °C for 15 min, 10 °C/min, to 180 °C). t_R 3.6 min (*R*); t_R 3.8 min (*S*). ^{1}H NMR ($CDCl_3$), δ: 1.20 (s, 6H), 1.21 (s, 6H), 1.33 (d, 3H, *J*= 7.5 Hz), 2.43 (q, 1H, *J*= 7.5 Hz), 7.11-7.15 (m, 1H), 7.20-7.28 (m, 4H).

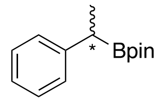

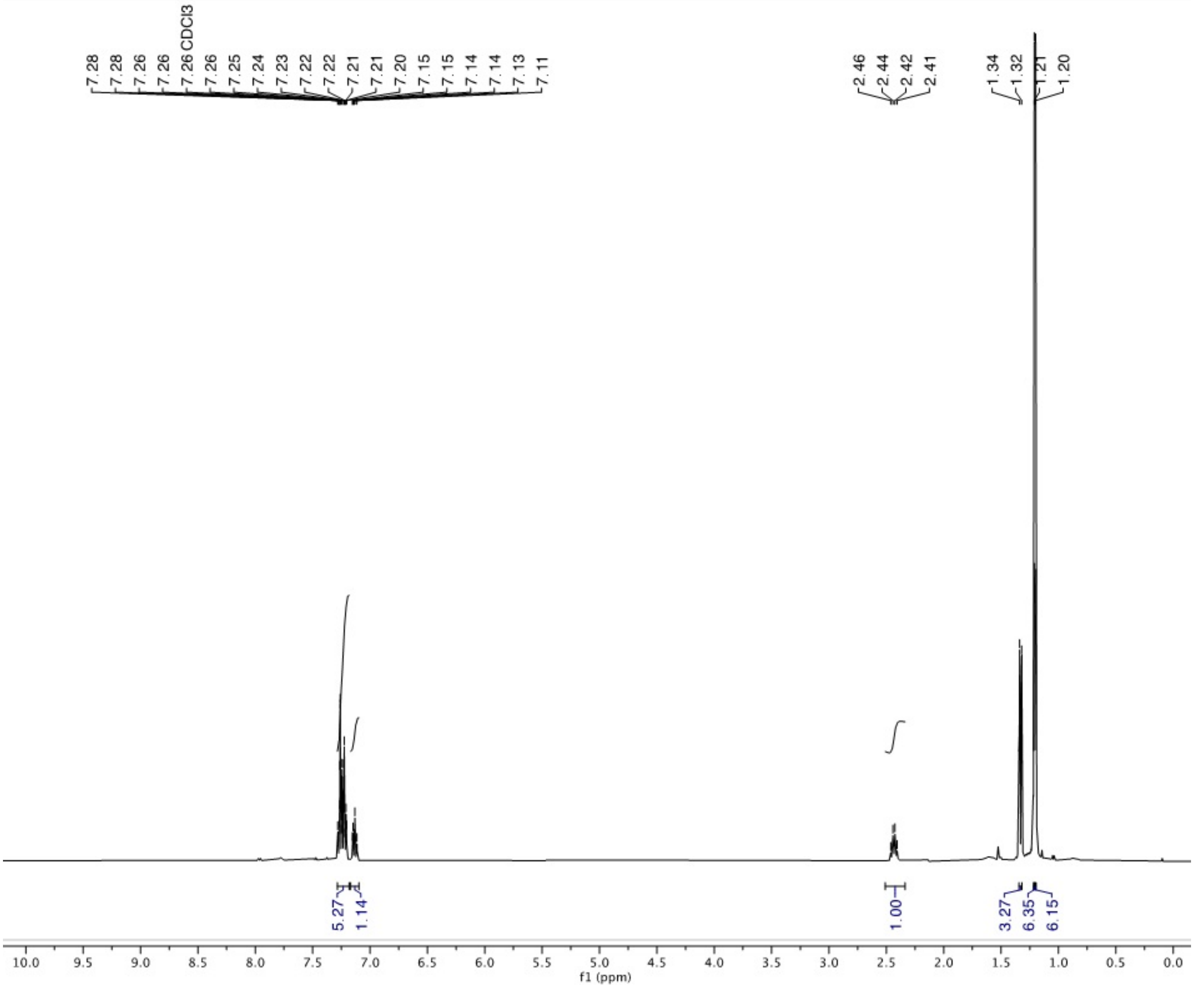

Figure S84. ^{1}H NMR spectrum of 2-(1-phenylethyl)-4,4,5,5-tetramethyl-1,3,2-dioxaborolane (from **S40**) in $CDCl_3$.

(a)

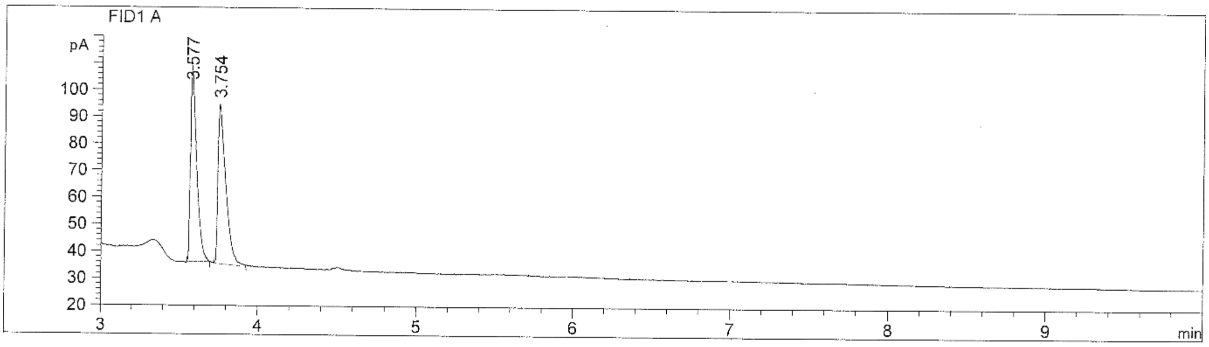

(b)

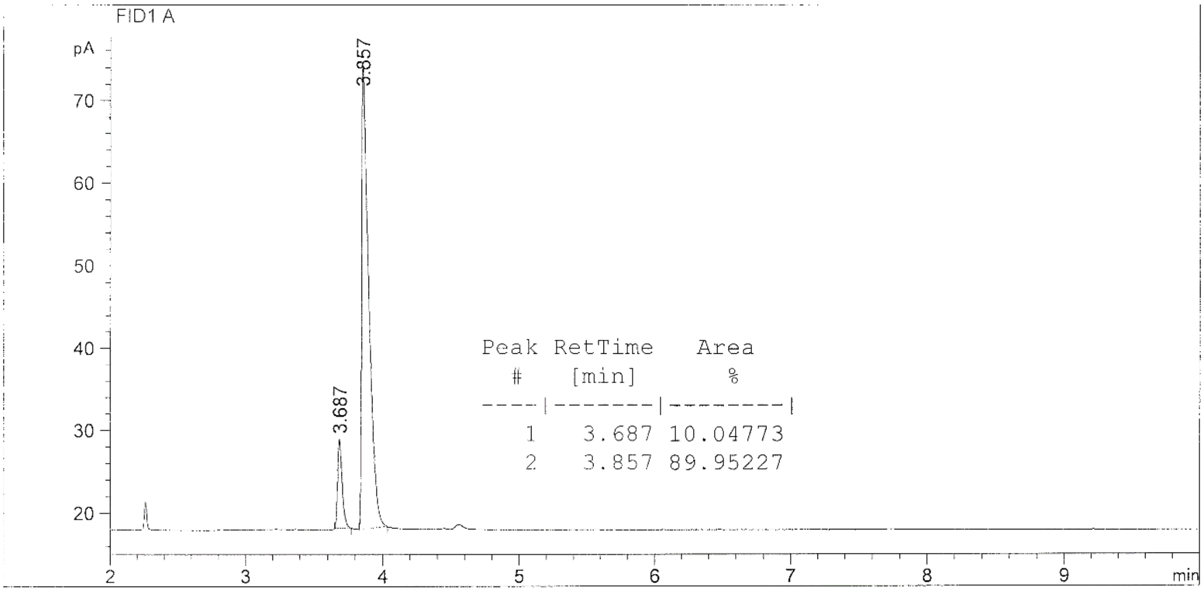

Figure S85. GC trace of (a) racemic and (b) enantioenriched product (80% (*S*) ee using **4b** catalytic system; Scheme 3).

2-(1-Cyclohexylethyl)-4,4,5,5-tetramethyl-1,3,2-dioxaborolane (from S41).[28] Enantiomeric excess

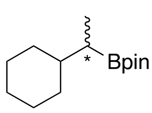

were determined after oxidation/acetylation of the pinacolborane derivative to the corresponding trifluoroacetylated compound using sodium perborate monohydrate (75 mg, 0.75 mmol) in a THF/H_2O solution (1:1, 4 mL). After 1 h, the reaction mixture was extracted with DCM (3×1 mL), dried with $MgSO_4$ and filtrated. To the filtrate acetic anhydride (240 μL, 2.5 mmol), NEt_3 (700 μL, 5 mmol) and a pinch of DMAP (c.a. 1 mg) was added. After 12 h, the volatiles were removed under vacuum. Enantiomeric excess determined by GC using HP CHIRAL 20B column (1.5 mL/min H_2, 50 °C, 1 oC/min to 230 ºC). t_R 49.8 min (*S*); t_R 52.5 min (*R*). ^{1}H NMR ($CDCl_3$), δ: 1.24 (bs, 12H), 0.87-1.26 (b, 10H), 1.52-1.72 (m, 5H).

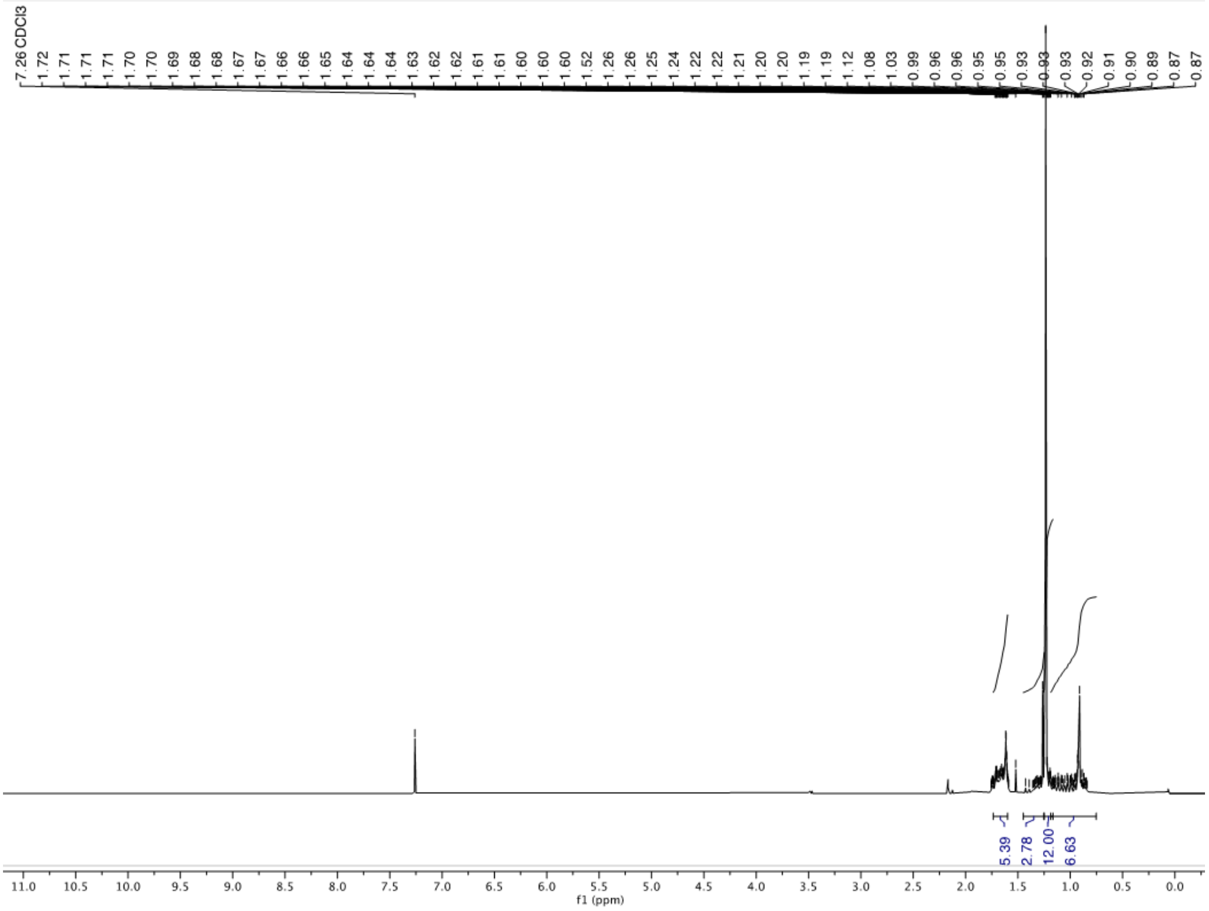

Figure S86. ^{1}H NMR spectrum of 2-(1-cyclohexylethyl)-4,4,5,5-tetramethyl-1,3,2-dioxaborolane (from **S41**) in $CDCl_3$.

(a)

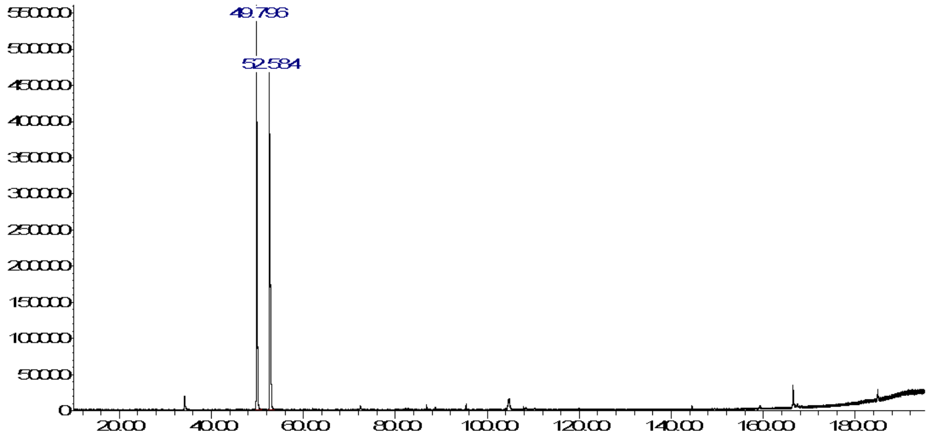

b)

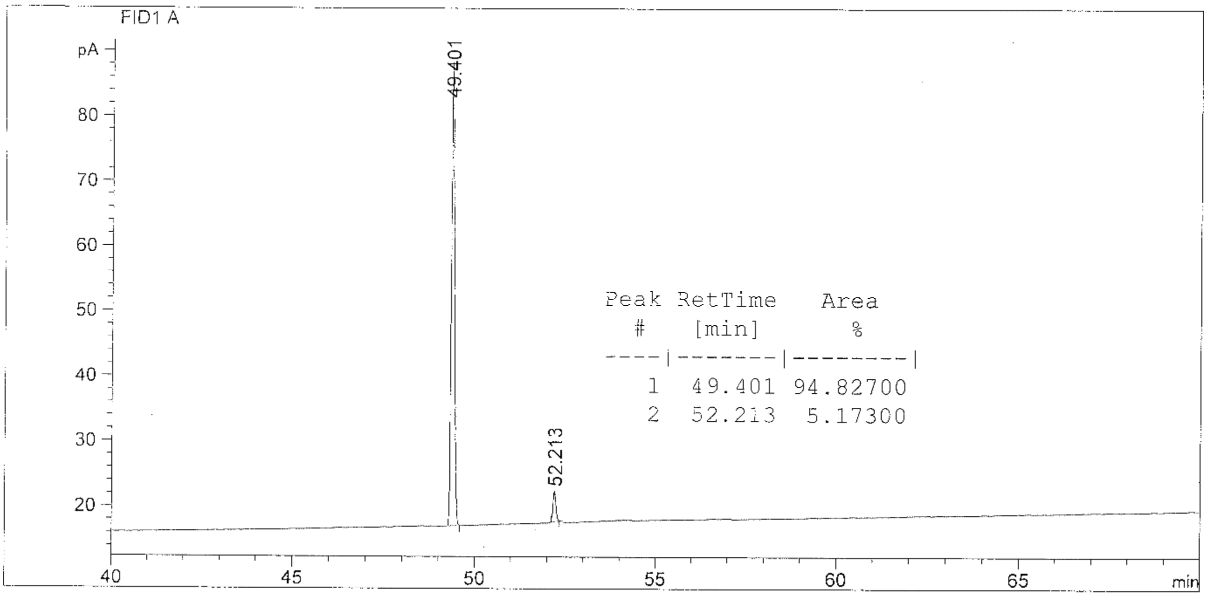

Figure S87. GC trace of: (a) racemic and (b) enantioenriched product (89% (*S*) ee using **4b** catalytic system; Scheme 3).

Ethyl 3-((diphenylphosphoryl)oxy)-3-phenylpropanoate (from S42).[29] Enantiomeric excess

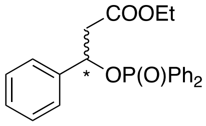

determined by HPLC using Chiralcel OD-H (hexane/2- propanol=90/10, 0.5 mL/min, 220 nm). t_R 29.7 min (*R*); t_R 33.6 min (*S*). ^{1}H NMR ($CDCl_3$), δ: 1.05 (t, 3H, *J*= 7.2 Hz), 2.84 (dd, 1H, *J*= 7.1 Hz, *J*= 15.2 Hz), 3.16 (dd, 1H, *J*= 7.1 Hz, *J*= 15.2 Hz), 3.86-3.98 (m, 2H), 5.67-5.73 (m, 1H), 7.17-7.23 (m, 7H), 7.35-7.39 (m, 4H), 7.45-7.54 (m, 2H), 7.69-7.74 (m, 2H).

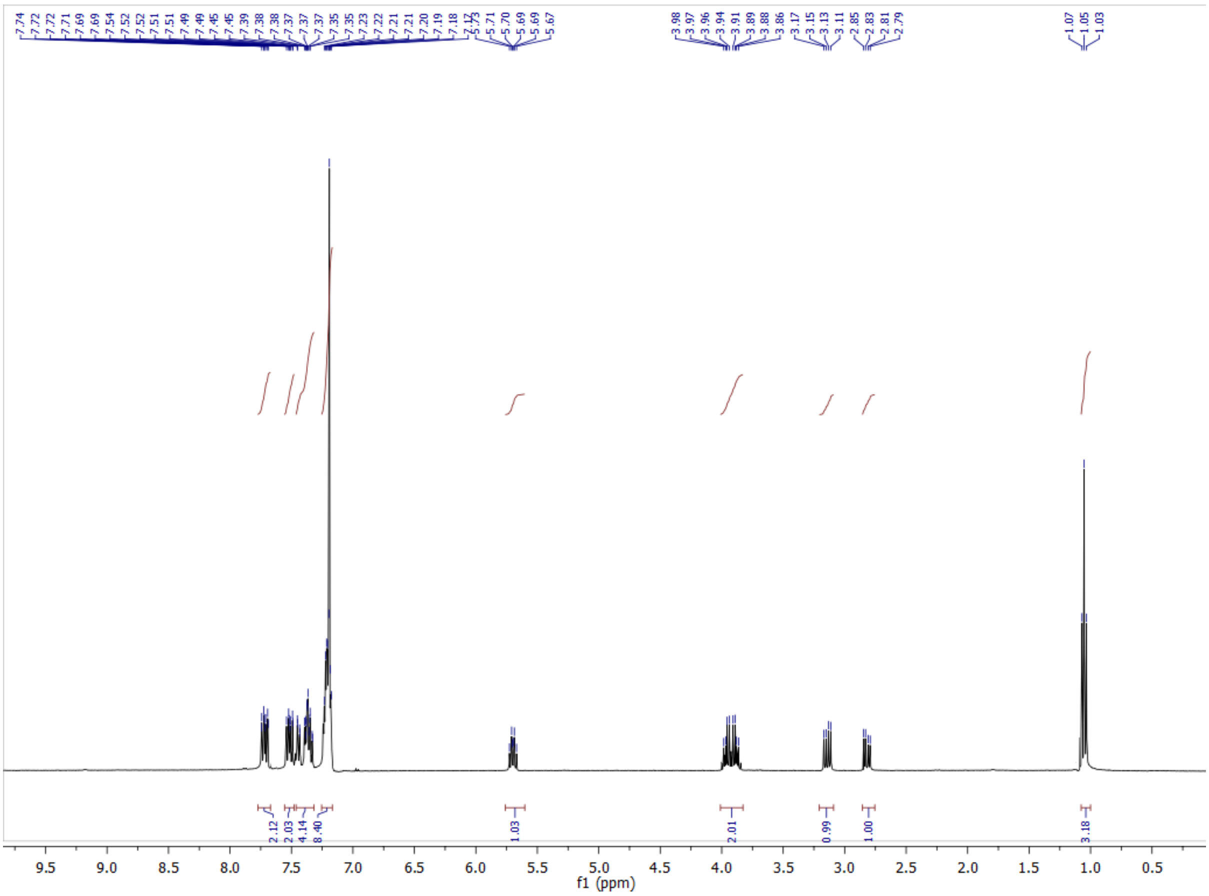

Figure S88. ^{1}H NMR spectrum of ethyl 3-((diphenylphosphoryl)oxy)-3-phenylpropanoate (from **S42**) in $CDCl_3$.

(a)

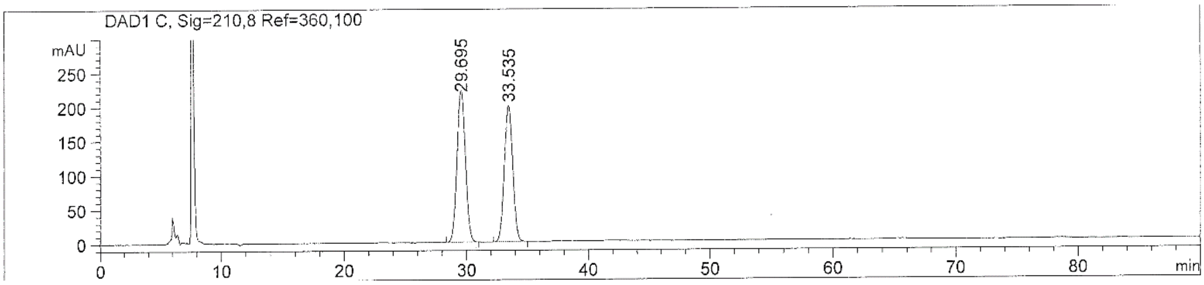

(b)

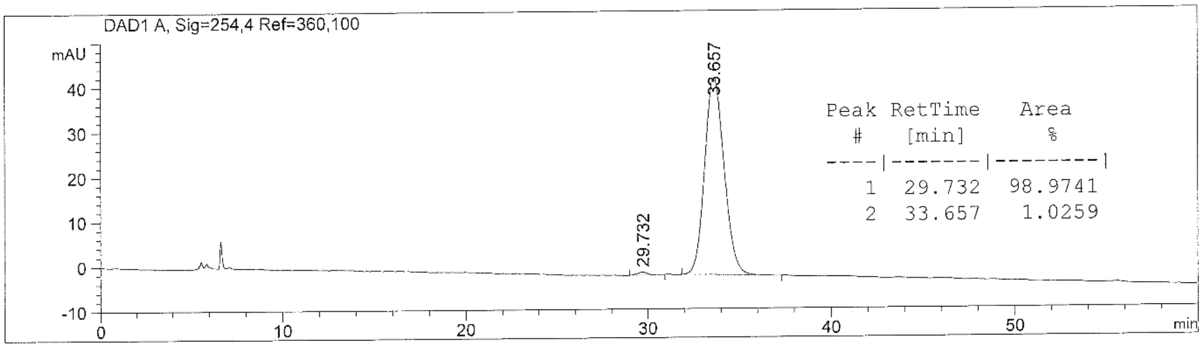

Figure S89. HPLC trace of: (a) racemic and (b) enantioenriched product (98% (*S*) ee using **4a** catalytic system; Scheme 3).

Ethyl 3-((diphenylphosphoryl)oxy)-3-(4-(trifluoromethyl)phenyl)propanoate (from S43).[29]

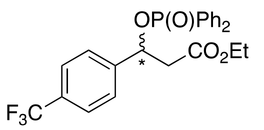

Enantiomeric excess determined by HPLC using Chiralcel OD-H (hexane/2-propanol=90/10, 0.5 mL/min, 220 nm). t_R 17.5 min (*R*); t_R 26.6 min (*S*). ^{1}H NMR ($CDCl_3$), δ: 1.13 (t, 3H, *J*= 7.2 Hz), 2.89 (dd, 1H, *J*= 6.4 Hz, *J*= 15.6 Hz), 3.20 (dd, 1H, *J*= 6.4 Hz, *J*= 15.6 Hz), 3.94-4.06 (m, 2H), 5.78-5.84 (m, 1H), 7.29-7.80 (m, 14H).

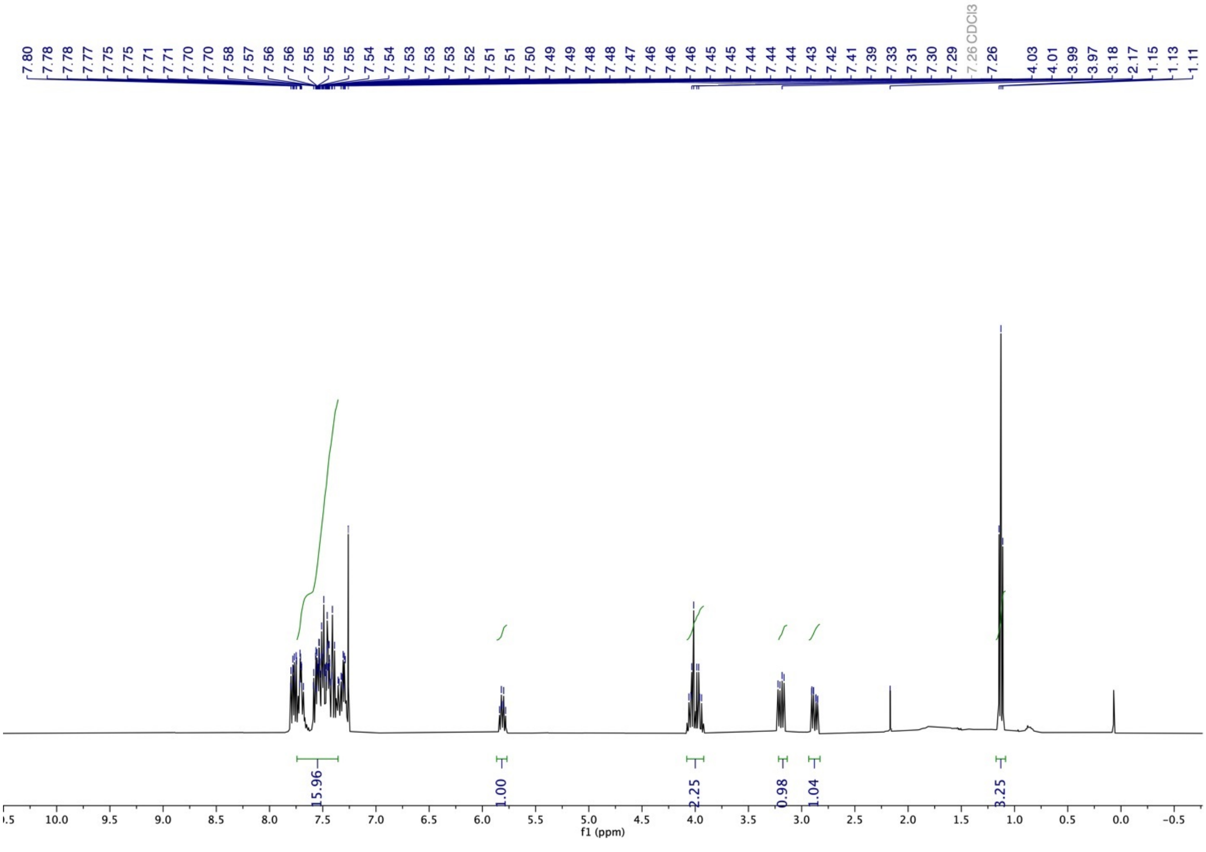

Figure S90. ^{1}H NMR spectrum of ethyl 3-((diphenylphosphoryl)oxy)-3-(4-(trifluoromethyl)phenyl)propanoate (from **S43**) in $CDCl_3$.

(a)

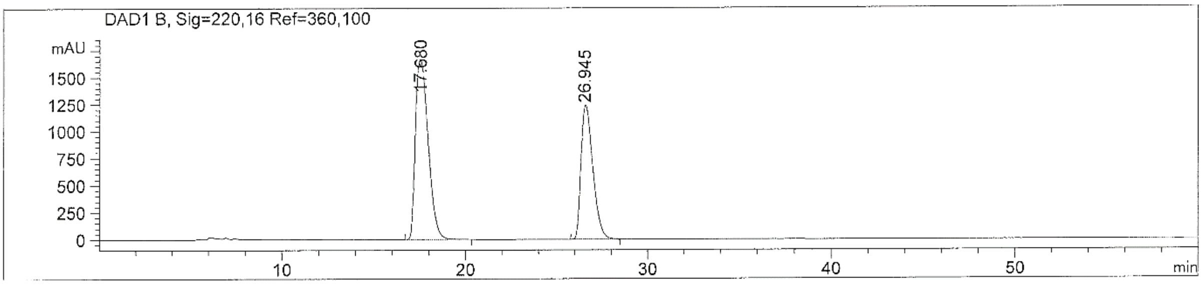

(b)

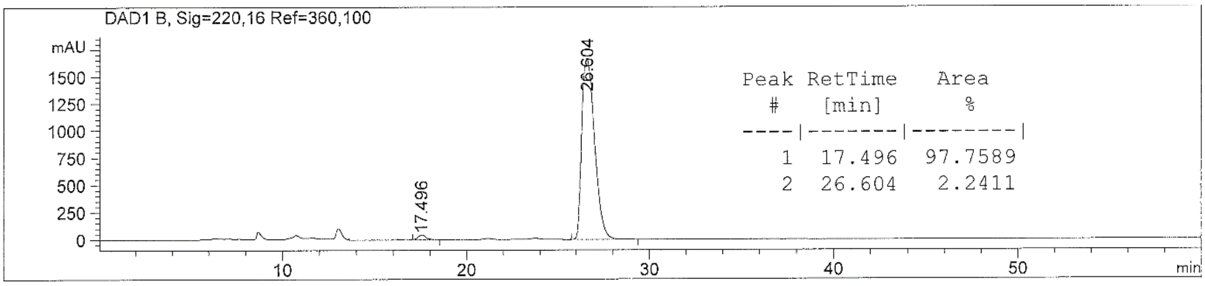

Figure S91. HPLC trace of: (a) racemic and (b) enantioenriched product (95% (*S*) ee using **4a** catalytic system; Scheme 3).

Ethyl 3-((diphenylphosphoryl)oxy)-3-(4-(trifluoromethyl)phenyl)propanoate (from S44).[29]

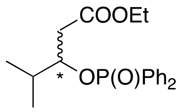

Enantiomeric excess determined by HPLC using Chiralcel OD-H (hexane/2-propanol=90/10, 0.5 mL/min, 220 nm). t_R 17.5 min (*R*); t_R 26.5 min (*S*). ^{1}H NMR ($CDCl_3$), δ: 0.91 (t, 6H, *J*= 7.1 Hz), 1.14 (t, 3H, *J*= 7.2 Hz), 2.02-2.11 (m, 1H), 2.55 (dd, 1H, *J*= 5.0 Hz, *J*= 15.6 Hz), 2.75 (dd, 1H, *J*= 7.4 Hz, *J*= 15.5 Hz), 3.88-4.00 (m, 2H), 4.74 (br, 1H), 7.40-7.81 (m, 10H).

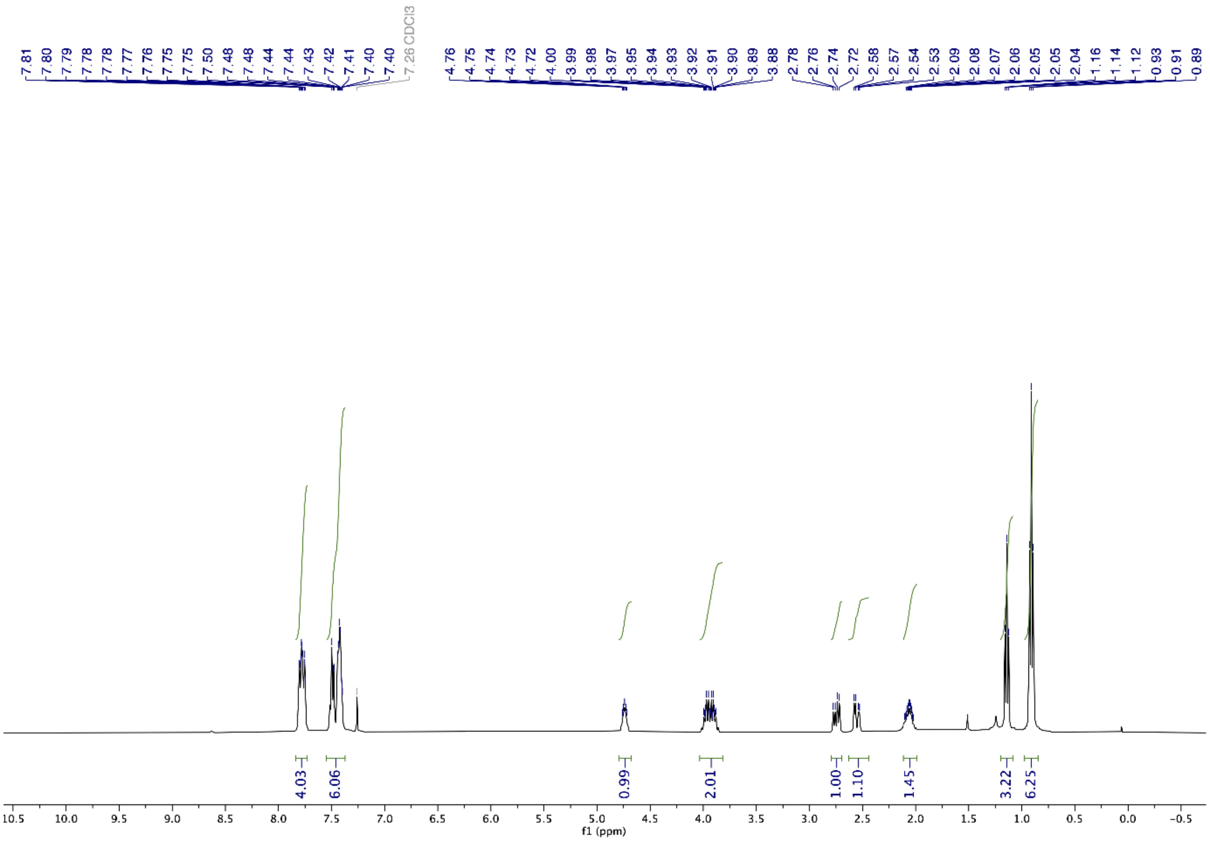

Figure S92. ^{1}H NMR spectrum of ethyl 3-((diphenylphosphoryl)oxy)-3-(4-(trifluoromethyl)phenyl)propanoate (from **S44**) in $CDCl_3$.

(a)

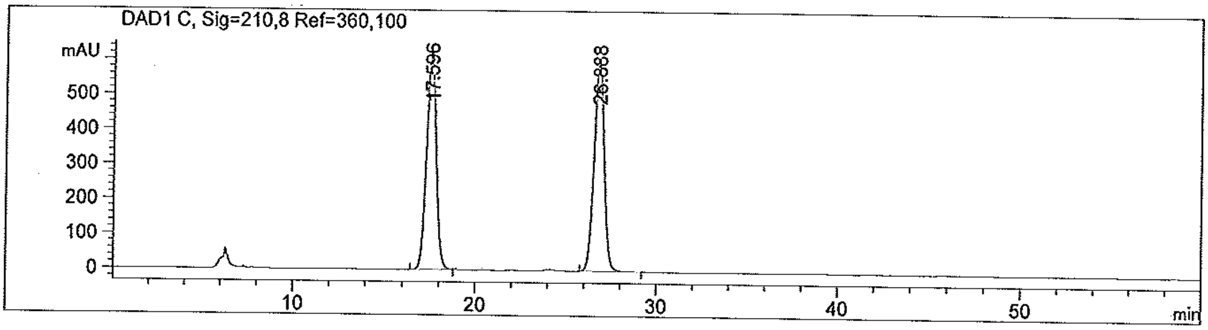

(b)

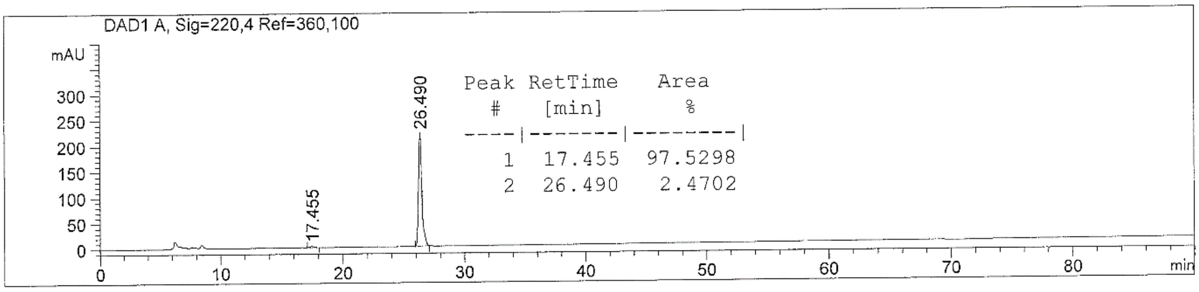

Figure S93. HPLC trace of: (a) racemic and (b) enantioenriched product (95% (*S*) ee using **4a** catalytic system; Scheme 3).

1-Phenylpropyl diphenylphosphinate (from S45).[29] Enantiomeric excess determined by HPLC using Chiralcel AD (hexane/2-propanol=90/10, 0.5 mL/min, 220 nm). t_R = 24.5 min (*R*); t_R 42.2 min (*S*). ^{1}H NMR ($CDCl_3$), δ: 0.84 (t, 3H, *J* = 7.4 Hz), 1.91-1.98 (m, 1H), 2.06-2.12 (m, 1H), 5.23-5.29 (m, 1H), 7.21-7.85 (m, 15H).

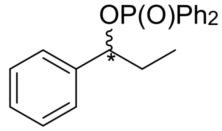

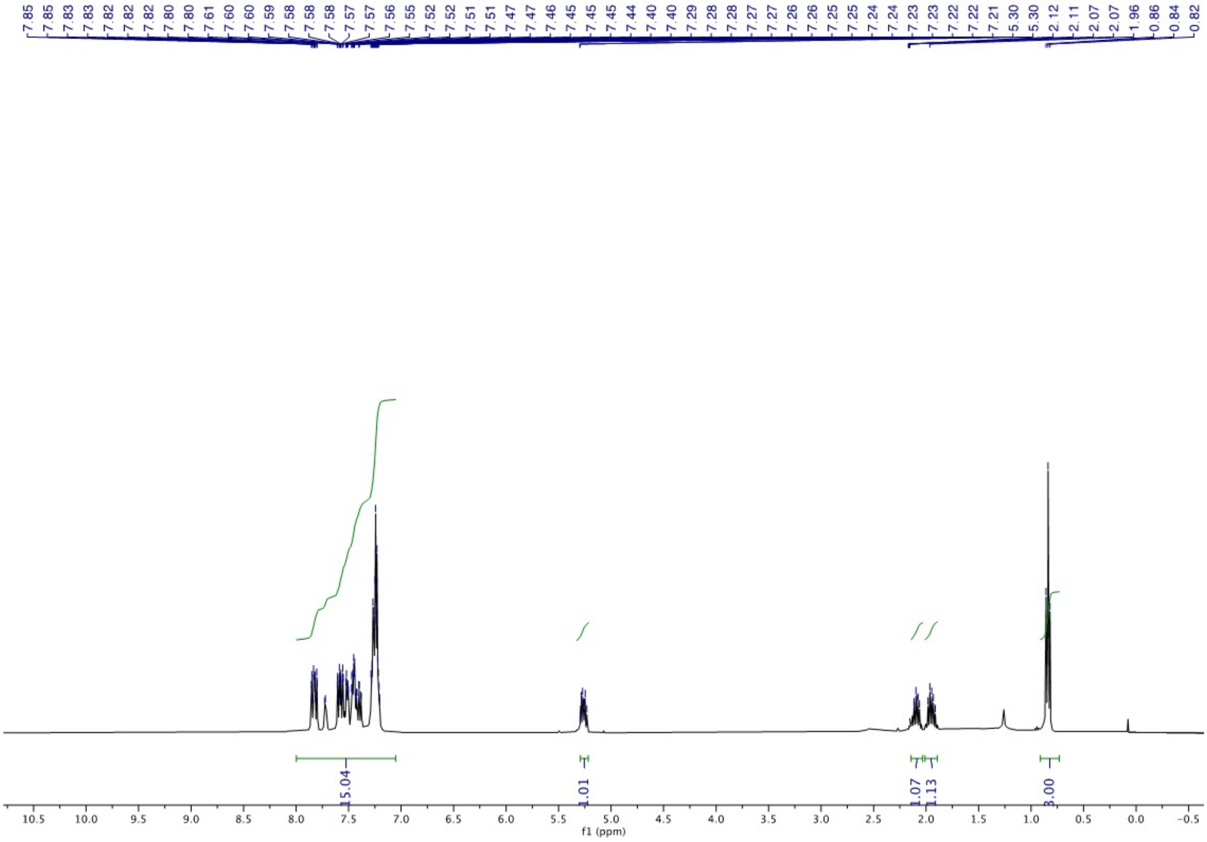

Figure S94. ^{1}H NMR spectrum of 1-phenylpropyl diphenylphosphinate (from **S45**) in $CDCl_3$.

(a)

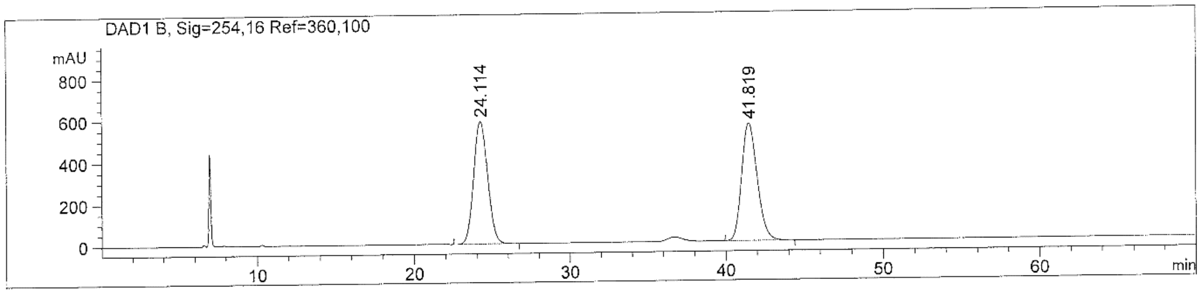

(b)

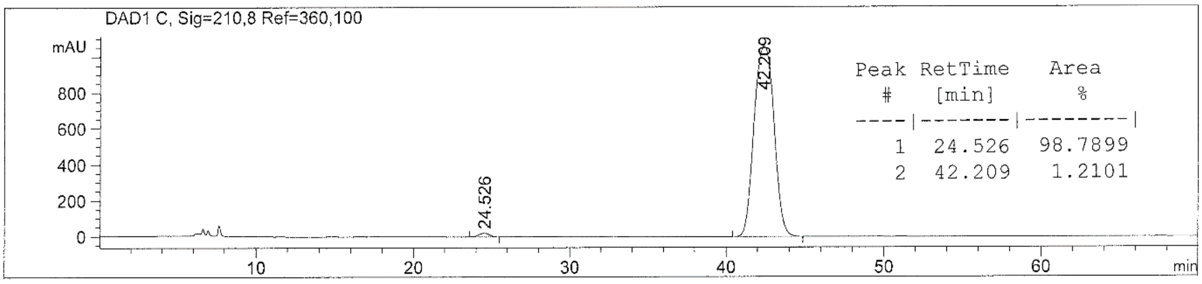

Figure S95. HPLC trace of: (a) racemic and (b) enantioenriched product (98% (*S*) ee using **4a** catalytic system; Scheme 3).

2,2-Dimethylpentan-3-yl diphenylphosphinate (from S46).[29] Enantiomeric excess determined by

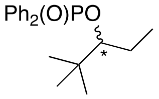

HPLC using Chiralcel IA (hexane/2-propanol=95/5, 0.5 mL/min, 220 nm). t_R 26.6 min (*R*); t_R 28.9 min (*S*). ^{1}H NMR ($CDCl_3$), δ: 0.70 (t, 3H, *J*= 7.2 Hz), 0.89 (s, 9H), 1.04-1.25 (m, 2H), 1.51-1.50 (m, 2H), 4.21-4.26 (m, 1H), 7.40-7.81 (m, 10H).

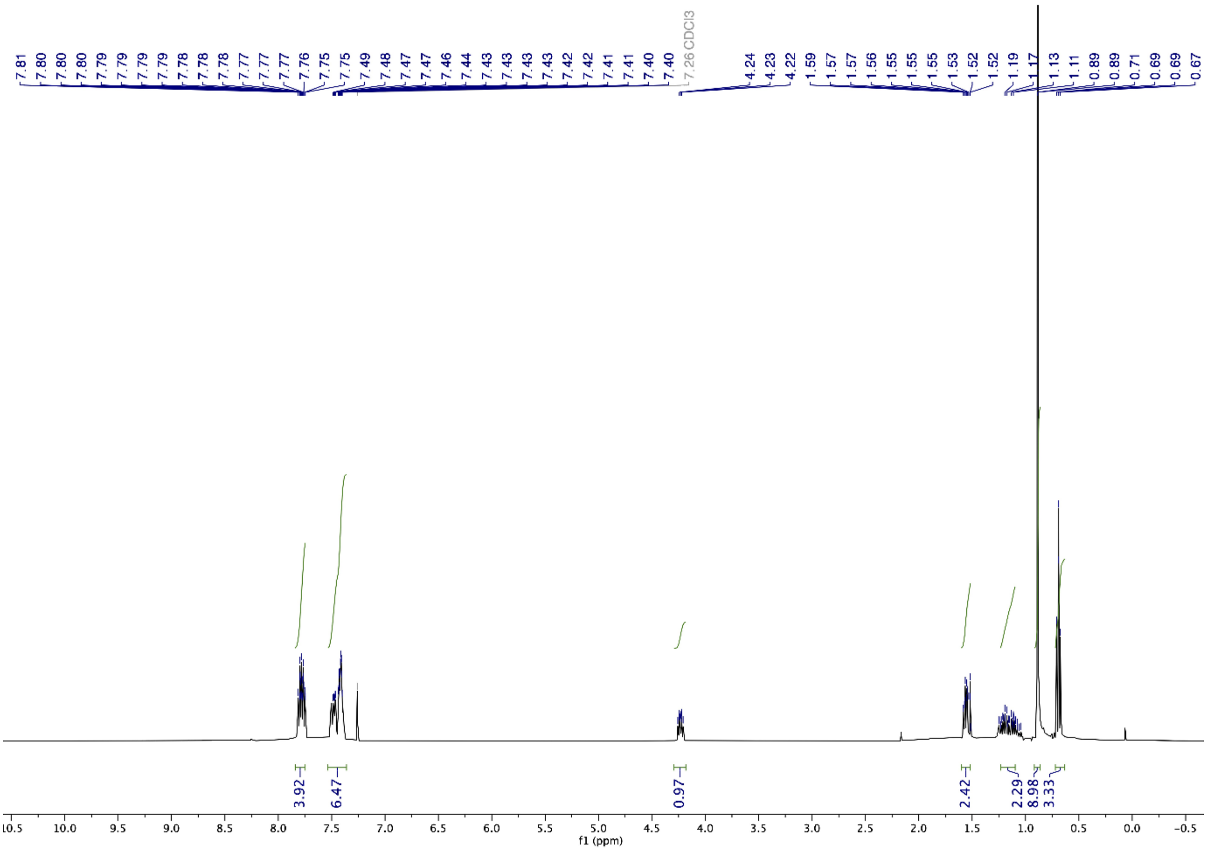

Figure S96. ^{1}H NMR spectrum of 2,2-dimethylpentan-3-yl diphenylphosphinate (from **S46**) in $CDCl_3$.

(a)

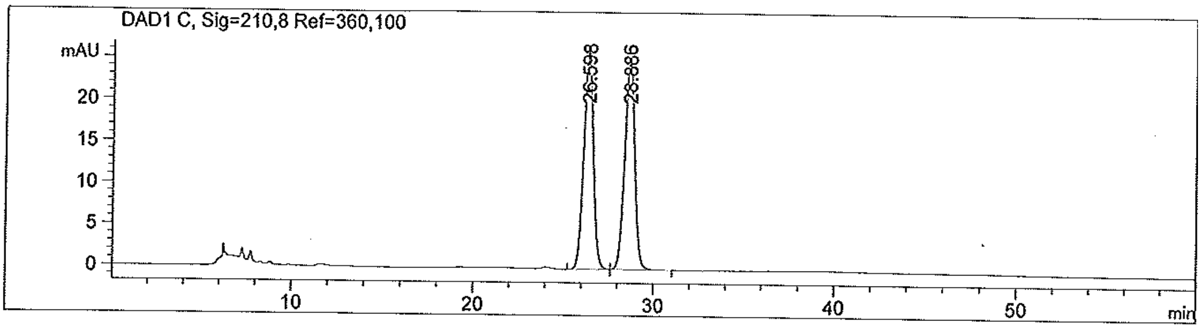

(b)

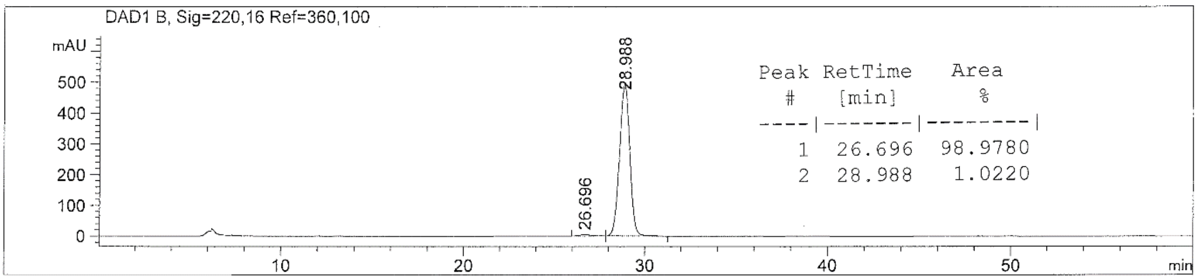

Figure S97. HPLC trace of: (a) racemic and (b) enantioenriched product (98% (*S*) ee using **4a** catalytic system; Scheme 3).

3-Methylbutan-2-yl diphenylphosphinate (from S47).[29] Enantiomeric excess determined by HPLC using Chiralcel S,S Whelk-01 (hexane/2-propanol=98/2, 0.5 mL/min, 220 nm). t_R 93.1 min (*R*); t_R 102.1 min (*S*). ^{1}H NMR ($CDCl_3$), δ: 0.90 (dd, 3H, *J*= 0.8 Hz, *J*= 6.8 Hz), 0.92 (dd, 3H, *J*= 0.8 Hz, *J*= 6.8 Hz), 1.22 (dd, 3H, *J*= 0.8 Hz, *J*= 6.4 Hz), 1.87-1.92 (m, 1H), 4.35-4.40 (m, 1H), 7.41-7.51 (m, 6H), 7.75-7.83 (m, 4H).

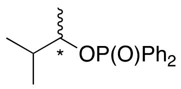

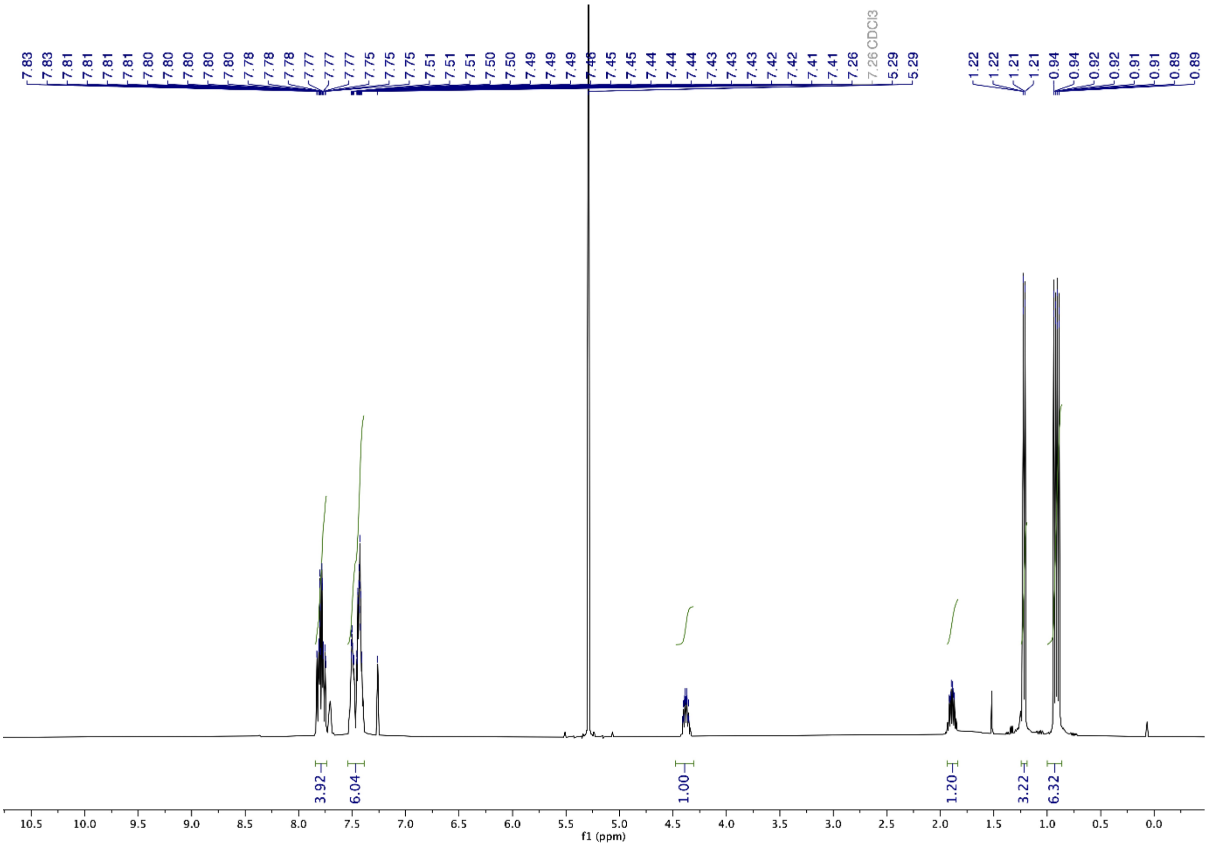

Figure S98. ^{1}H NMR spectrum of 3-methylbutan-2-yl diphenylphosphinate (from **S47**) in $CDCl_3$.The peak at 5.29 corresponds to CH_2Cl_2.

(a)

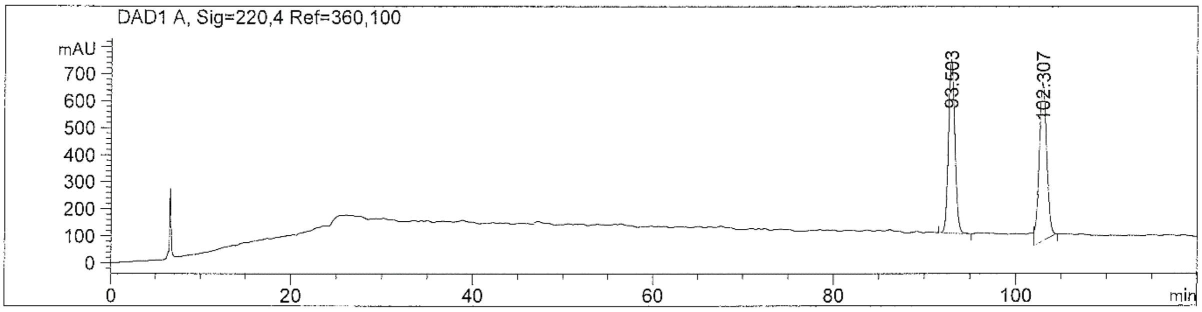

(b)

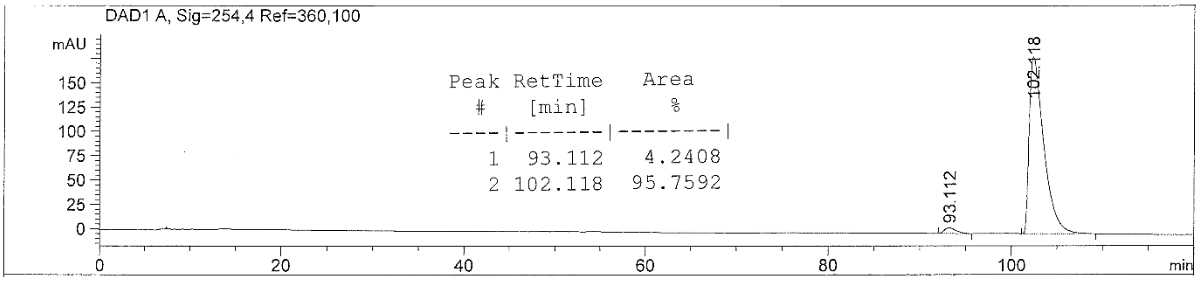

Figure S99. HPLC trace of: (a) racemic and (b) enantioenriched product (92% (*R*) ee using **4a** catalytic system; Scheme 3).

1-Phenylethyl diphenylphosphinate (from S48).[45] Enantiomeric excess determined by HPLC using Chiralcel AD (hexane/2-propanol=90/10, 0.5 mL/min, 220 nm). t_R 32.6 min (*R*); t_R 37.0 min (*S*). ^{1}H NMR ($CDCl_3$), δ: 1.66 (d, 3H, *J*= 6.5 Hz), 5.48-5.62 (m, 1H), 7.27-7.34 (m, 7H), 7.42-7.54 (m, 4H), 7.62-7.67 (m, 2H), 7.81-7.86 (m, 2H).

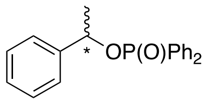

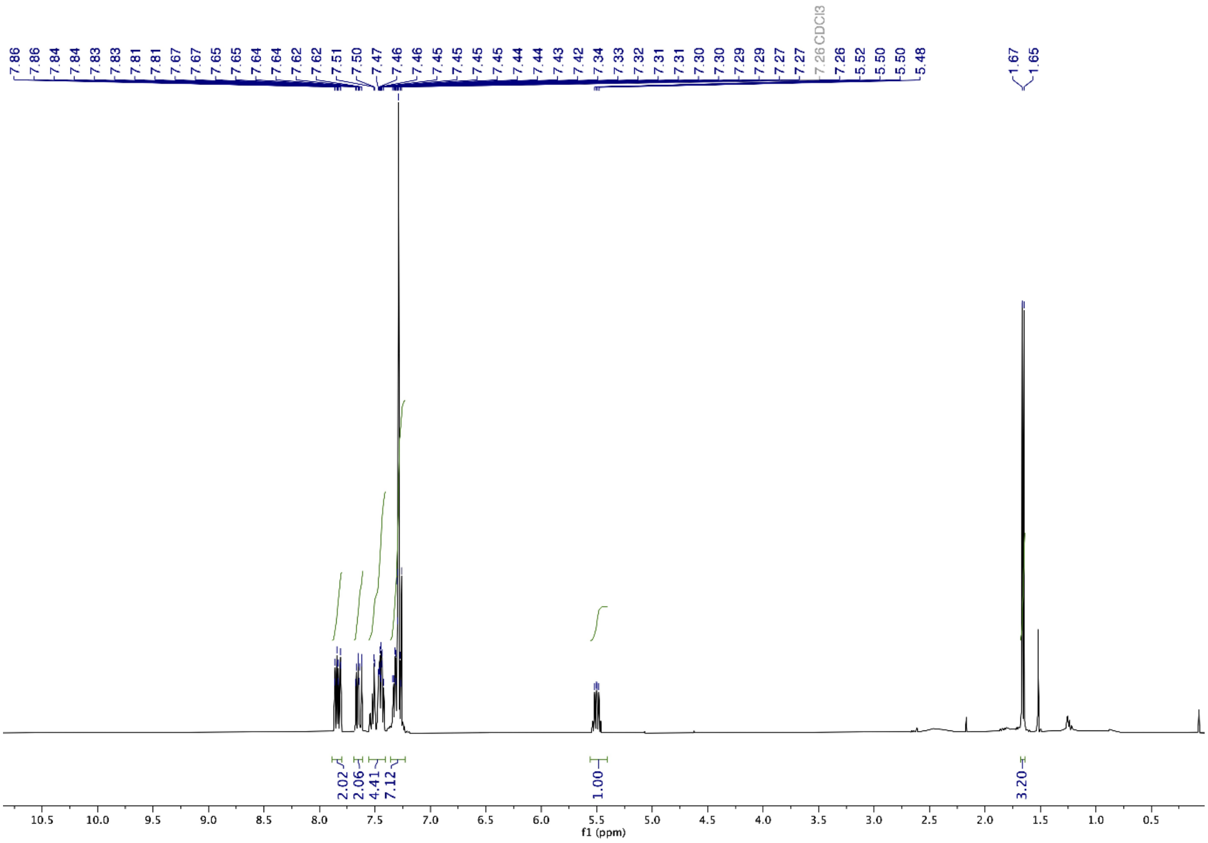

Figure S100. ^{1}H NMR spectrum of 1-phenylethyl diphenylphosphinate (from **S48**) in $CDCl_3$.

(a)

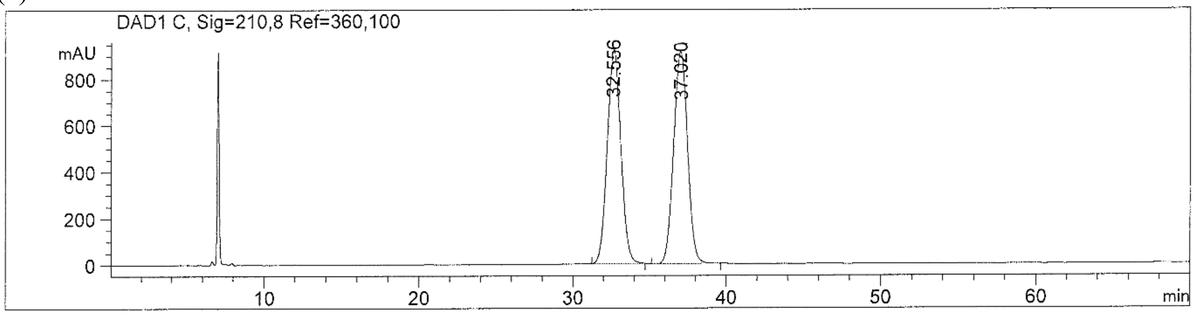

(b)

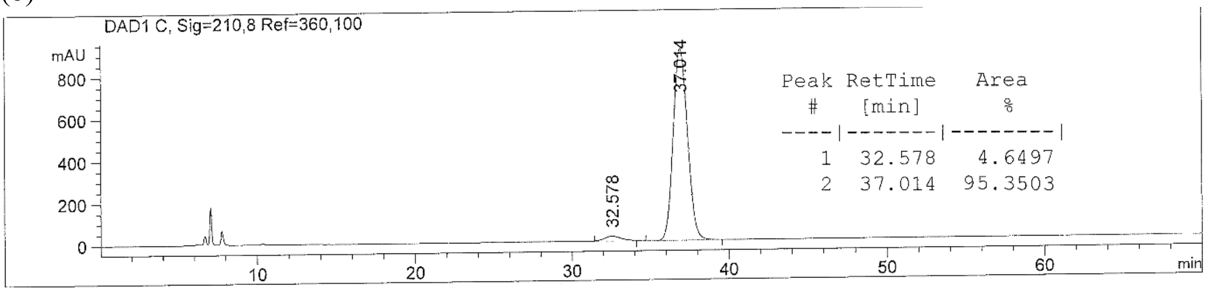

Figure S101. HPLC trace of: (a) racemic and (b) enantioenriched product (91% (*R*) ee using **4a** catalytic system; Scheme 3).

2-Benzylcyclohexan-1-one (from S49).[44] Enantiomeric excess determined by HPLC using Chiracel OJ-H column (hexane/2-propanol=97/3, 1 mL/min, 210 nm). t_R 8.4 min (*S*); t_R 9.2 min (*R*). ^{1}H NMR ($CDCl_3$), δ: 1.35-1.38 (m, 1H), 1.63-1.71 (m, 2H), 1.82-1.85 (m, 1H), 2.01-2.07 (m, 2H), 2.33-2.46 (m, 3H), 2.52-2.58 (m, 1H), 3.24 (dd, 1H, *J*= 14.0 Hz, *J*= 4.6 Hz), 7.15-7.22 (m, 3H), 7.26-7.30 (m, 2H).

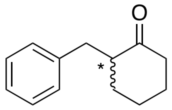

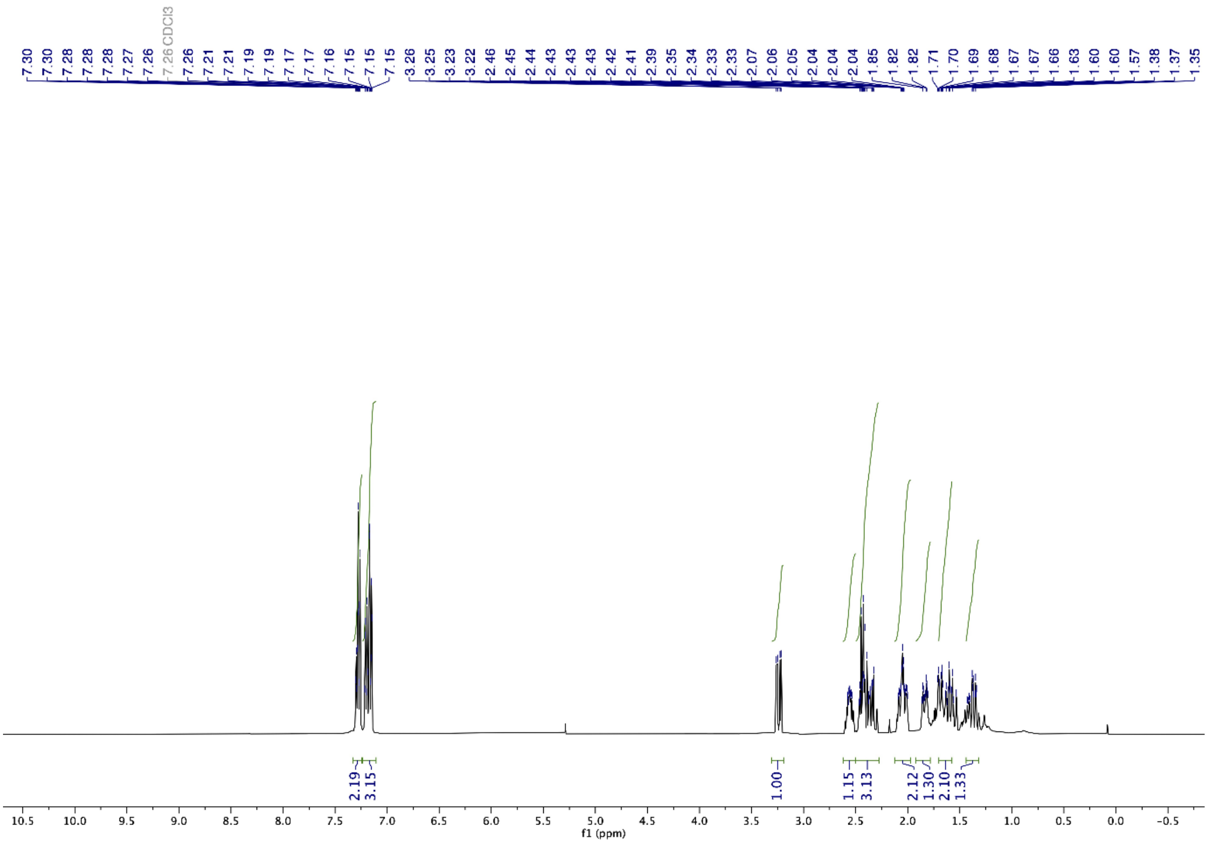

Figure S102. ^{1}H NMR spectrum of 2-benzylcyclohexan-1-one (from **S49**) in $CDCl_3$.

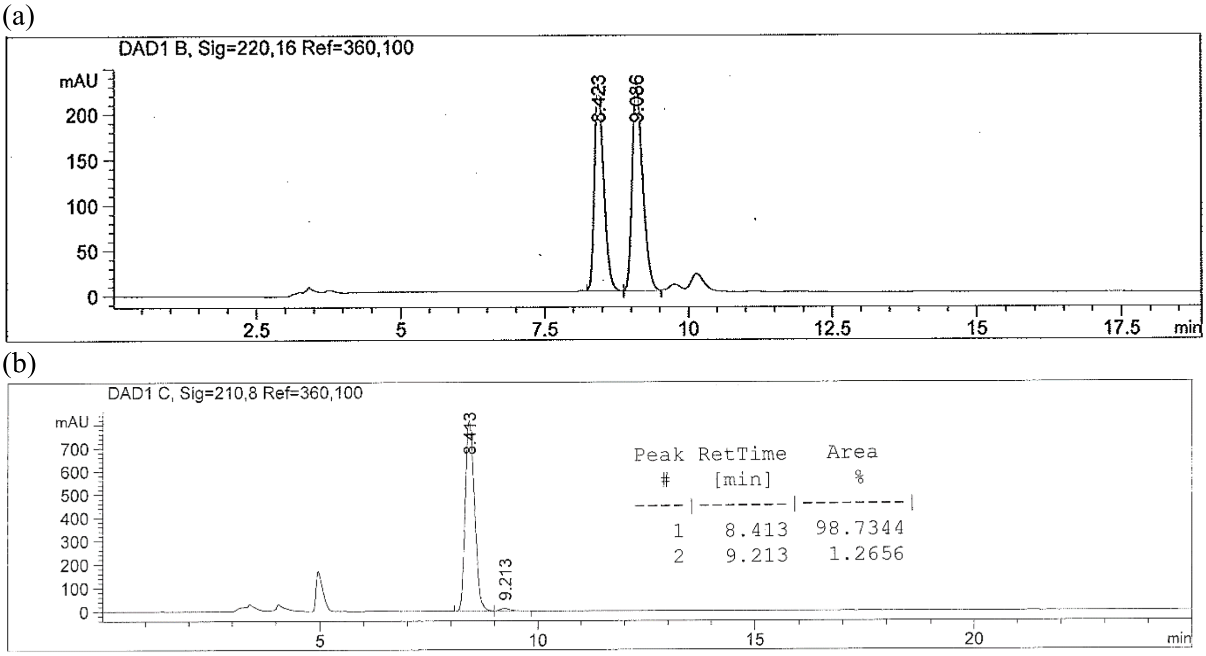

Figure S103. HPLC trace of: (a) racemic and (b) enantioenriched product (97% (*S*) ee using **4a** catalytic system; Scheme 4).

2-Benzylcyclopentan-1-one (from S50).[31] Enantiomeric excess determined by HPLC using Chiracel

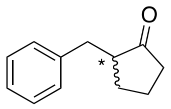

OJ-H column (hexane/2-propanol=99/1, 1 mL/min, 210 nm). t_R 13.0 min (*S*); t_R 14.4 min (*R*). ^{1}H NMR ($CDCl_3$), δ: 1.53-1.60 (m, 1H), 1.72-1.76 (m, 1H), 1.94-1.97 (m, 1H), 2.06-2.15 (m, 2H), 2.31-2.38 (m, 2H), 2.54 (dd, 1H, *J*= 13.8 Hz, *J*= 9.5 Hz), 3.15 (dd, 1H, *J*= 13.8 Hz, *J*= 4.1 Hz), 7.15-7.22 (m, 3H), 7.26-7.30 (m, 2H).

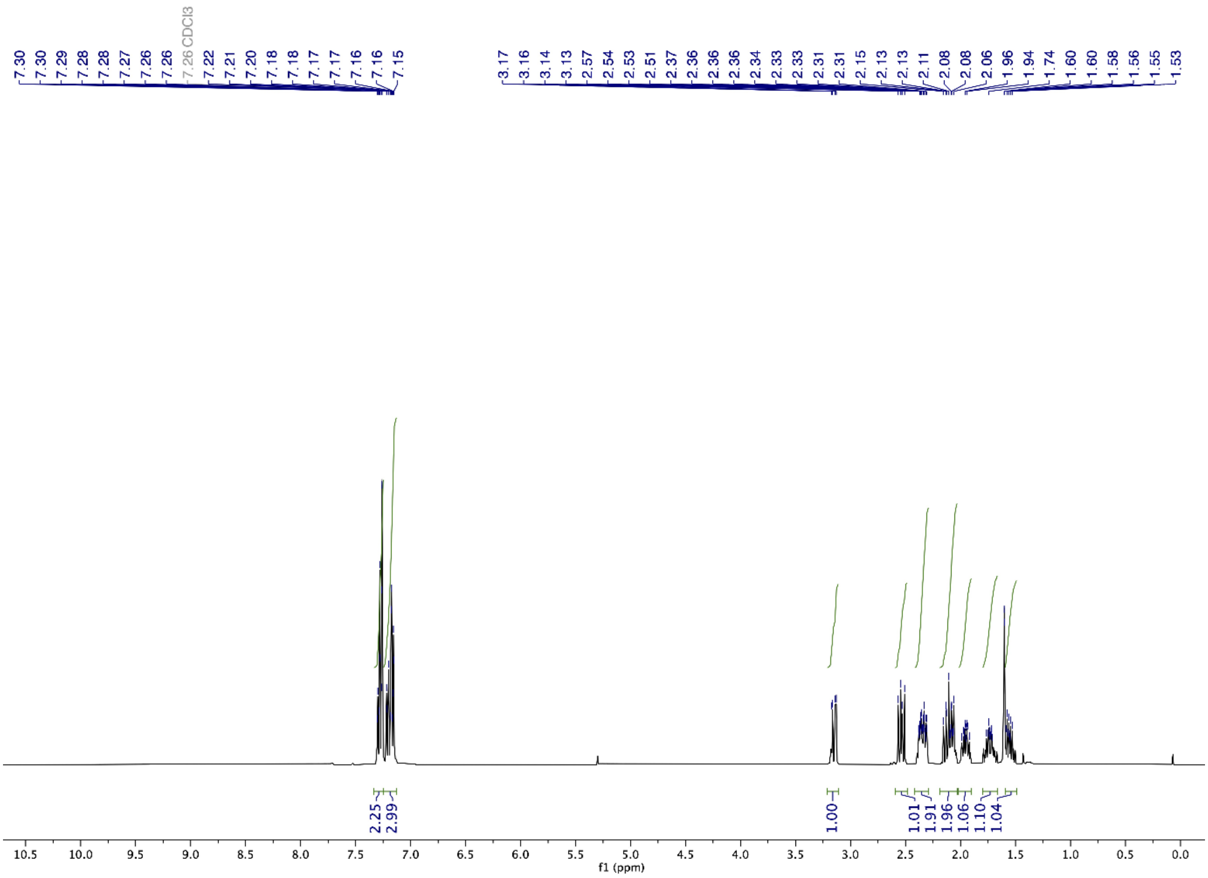

Figure S104. ^{1}H NMR spectrum of 2-benzylcyclopentan-1-one (from **S50**) in $CDCl_3$.

(a)

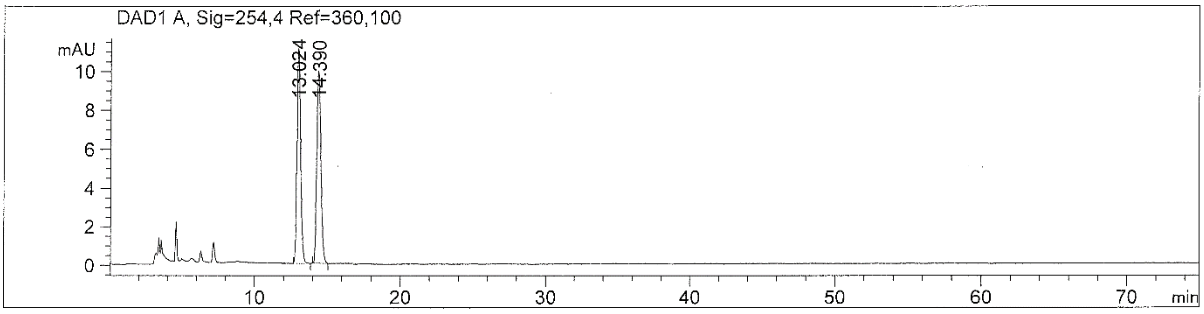

(b)

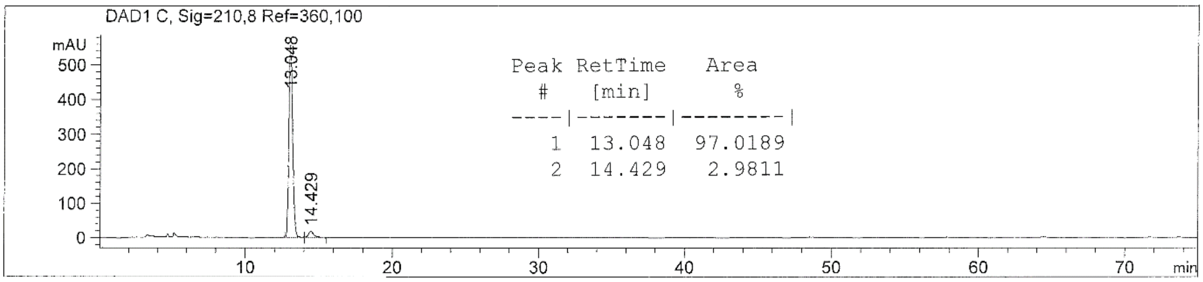

Figure S105. HPLC trace of: (a) racemic and (b) enantioenriched product (94% (*S*) ee using **4a** catalytic system; Scheme 4).

3-Benzyltetrahydro-2H-pyran-2-one (from S51).[34] Enantiomeric excess determined by HPLC using

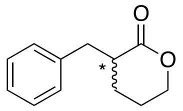

Chiracel OJ-H column (hexane/2-propanol=90/10, 1 mL/min, 210 nm). t_R 37.3 min (*R*); t_R 43.4 min (*S*). ^{1}H NMR ($CDCl_3$), δ: 1.49-1.56 (m, 1H), 1.79-1.94 (m, 3H), 2.68-2.77 (m, 2H), 3.34-3.41 (m, 1H), 4.23-4.34 (m, 2H), 7.18-7.32 (m, 5H).

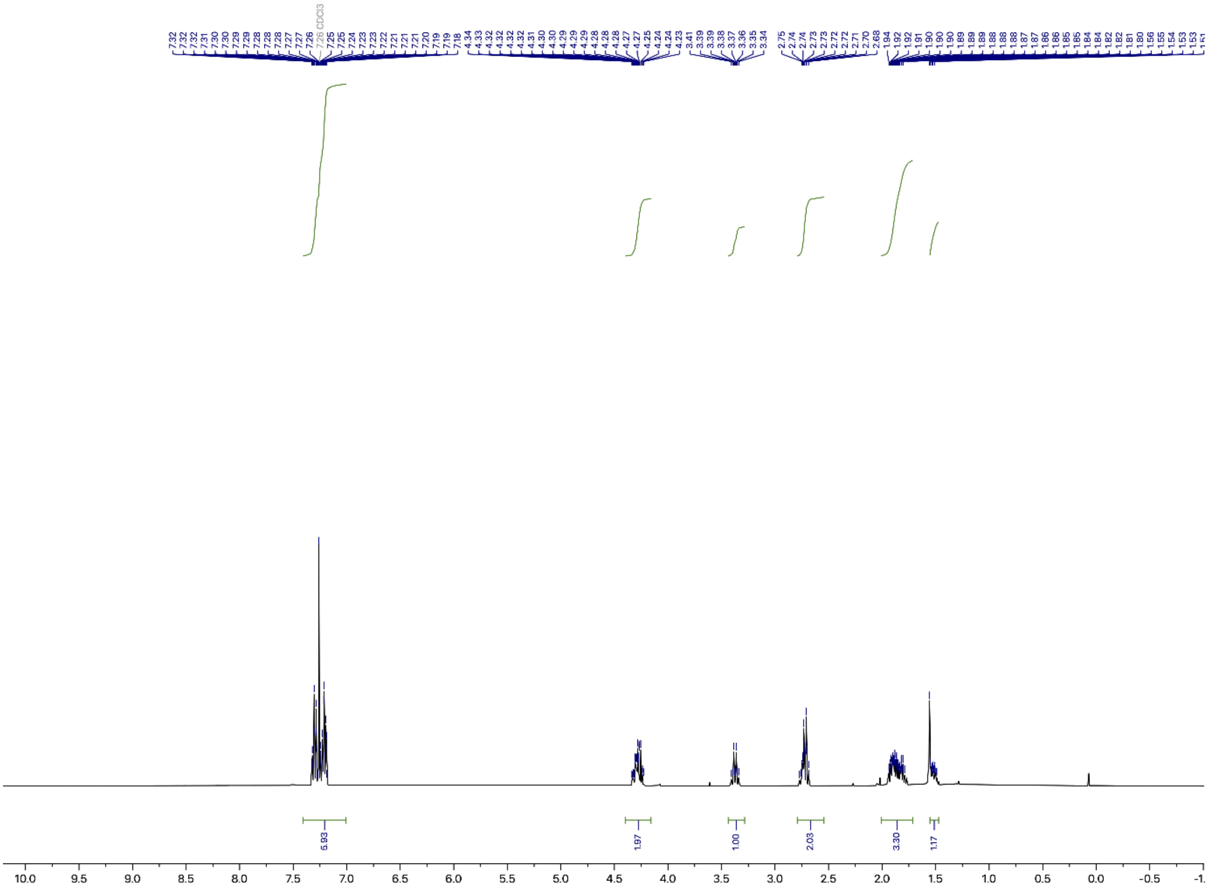

Figure S106. ^{1}H NMR spectrum of 3-benzyltetrahydro-2*H*-pyran-2-one (from **S51**) in $CDCl_3$.

(a)

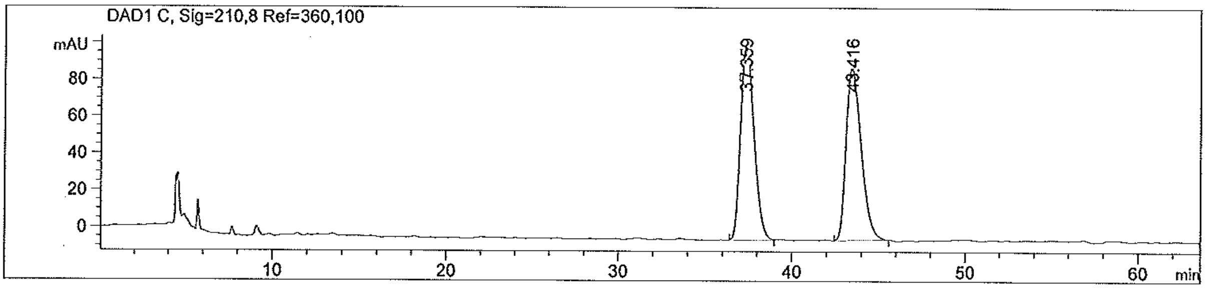

(b)

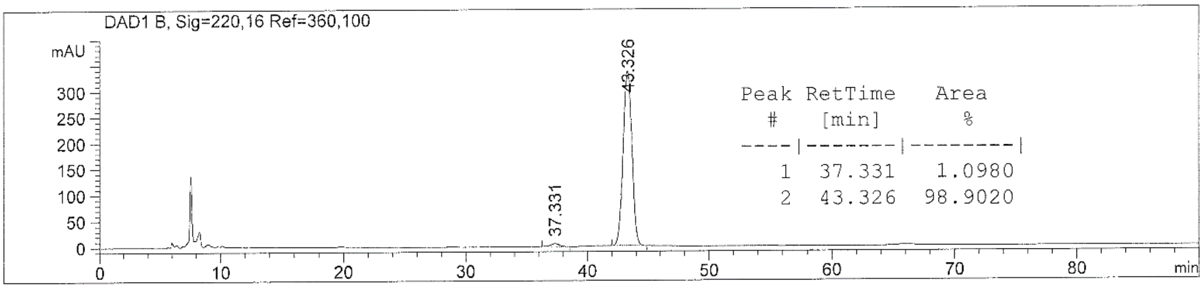

Figure S107. HPLC trace of: (a) racemic and (b) enantioenriched product (98% (*S*) ee using **4a** catalytic system; Scheme 4).

3-(4-Methoxybenzyl)tetrahydro-2H-pyran-2-one (from S52).[33] Enantiomeric excess determined by HPLC using Chiracel AD column (hexane/2- propanol=95/5, 0.5 mL/min, 220 nm). t_R 41.4 min (*S*); t_R 43.5 min (*R*). ^{1}H NMR ($CDCl_3$), δ: 1.49-1.54 (m, 1H), 1.77 – 1.94 (m, 3H), 2.64 –2.73 (m, 2H), 3.28 (q, 1H, *J*= 8.6 Hz), 3.79 (s, 3H), 4.22-4.30 (m, 2H), 6.84 (d, 2H, *J*= 8.6 Hz), 7.12 (d, 2H, *J*= 8.6 Hz).

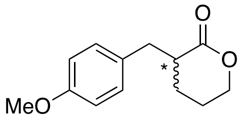

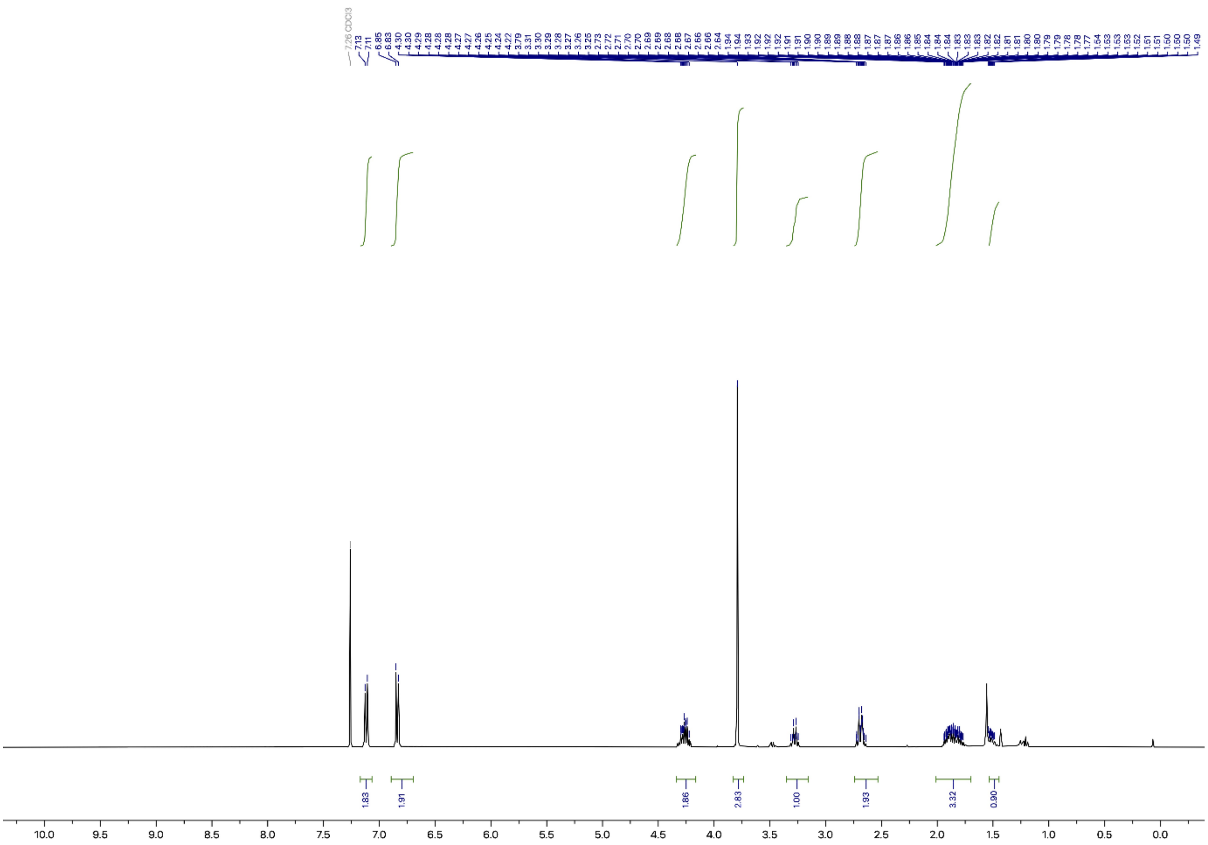

Figure S108. ^{1}H NMR spectrum of 3-(4-methoxybenzyl)tetrahydro-2*H*-pyran-2-one (from **S52**) in $CDCl_3$.

(a)

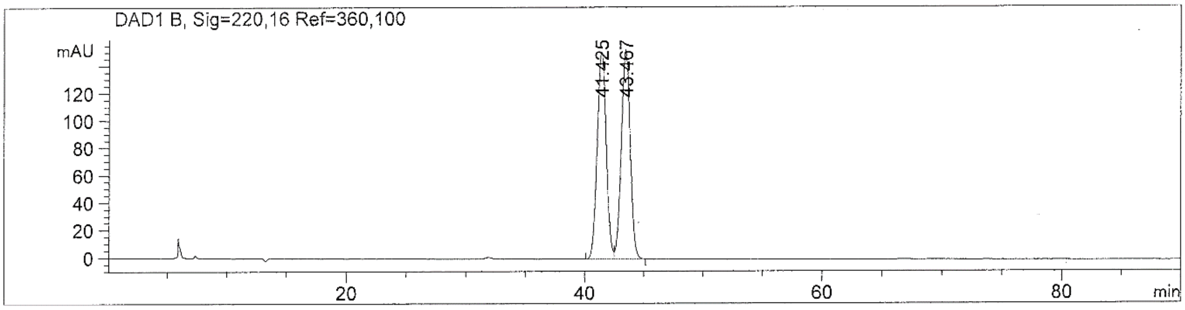

(b)

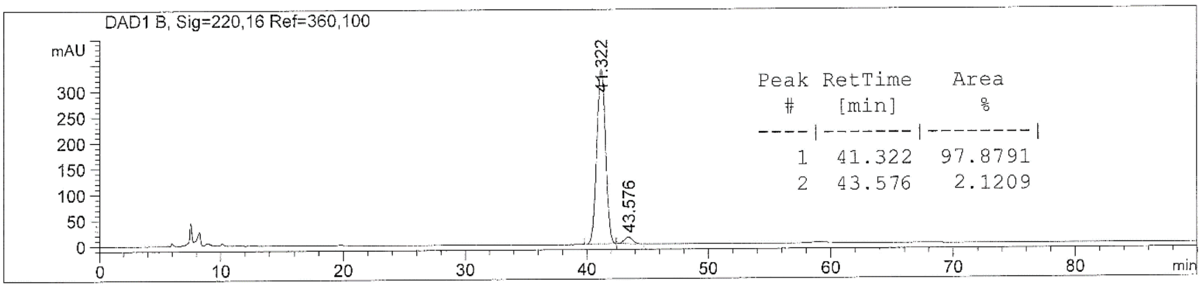

Peak #	RetTime [min]	Area %
1	41.322	97.8791
2	43.576	2.1209

Figure S109. HPLC trace of: (a) racemic and (b) enantioenriched product (96% (*S*) ee using **4a** catalytic system; Scheme 4).

3-(4-Bromobenzyl)tetrahydro-2H-pyran-2-one (from S53).[33] Enantiomeric excess determined by HPLC using Chiracel IA column (hexane/2- propanol=95/5, 0.5 mL/min, 210 nm).

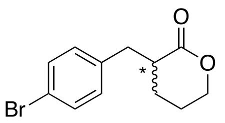

t_R 40.9 min (*S*); t_R 46.5 min (*R*). ^{1}H NMR ($CDCl_3$), δ: 1.48-1.53 (m, 1H), 1.80-1.93 (m, 3H), 2.68-2.74 (m, 2H), 3.26-3.30 (m, 1H), 4.22-4.32 (m, 2H), 7.08 (d, *J*= 8.3 Hz, 2H), 7.41 (d, 2H, *J*= 8.3 Hz).

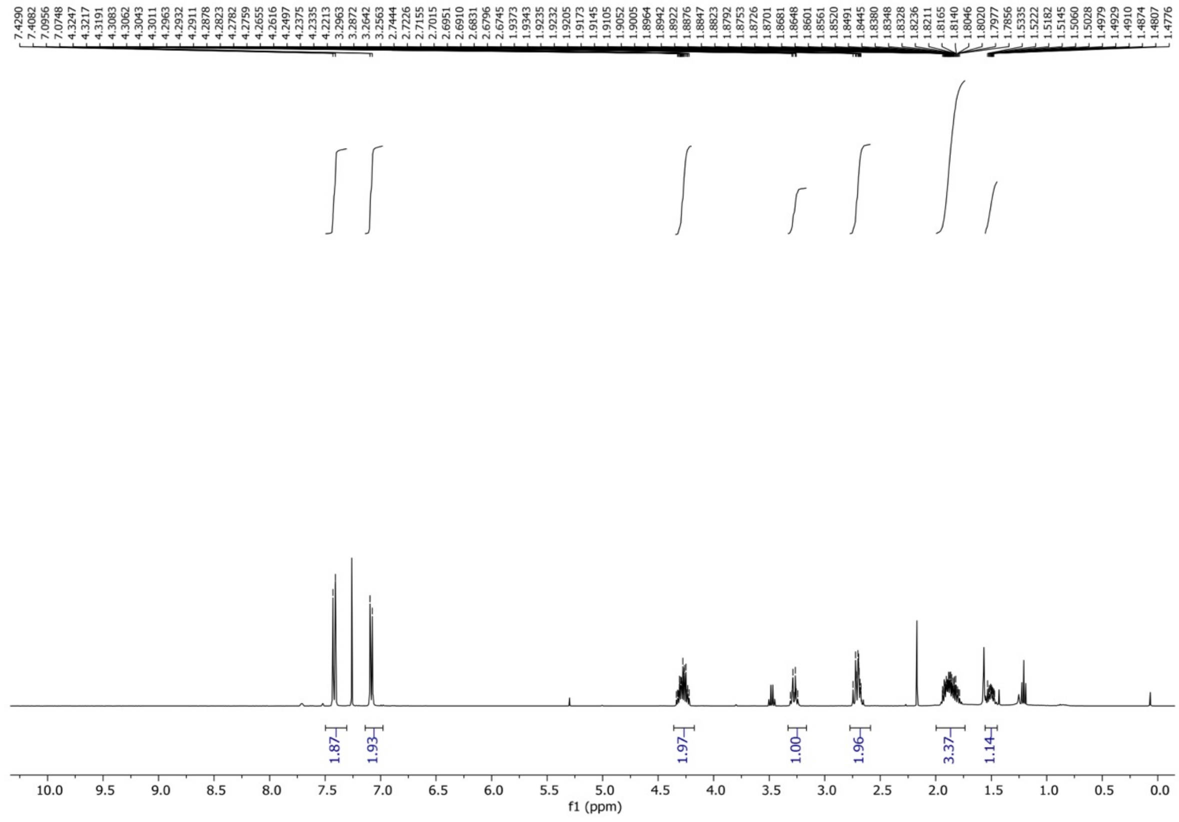

Figure S110. ^{1}H NMR spectrum of 3-(4-bromobenzyl)tetrahydro-2*H*-pyran-2-one (from **S53**) in $CDCl_3$.

(a)

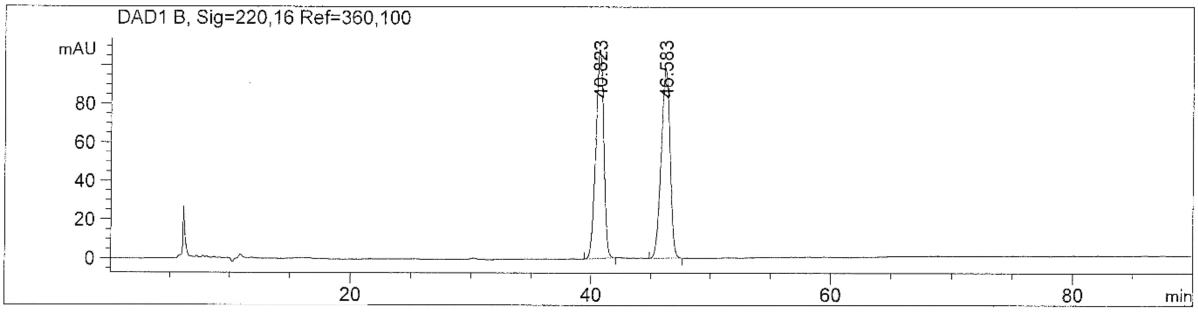

(b)

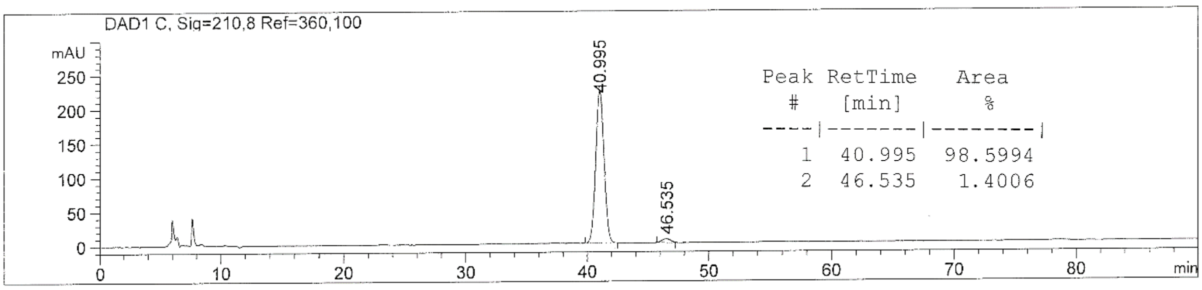

Peak #	RetTime [min]	Area %
1	40.995	98.5994
2	46.535	1.4006

Figure S111. HPLC trace of: (a) racemic and (b) enantioenriched product (97% (*S*) ee using **4a** catalytic system; Scheme 4).

3-(4-(Trifluoromethyl)benzyl)tetrahydro-2H-pyran-2-one (from S54).[33] Enantiomeric excess

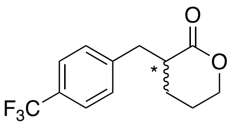

determined by HPLC using Chiracel IA column (hexane/2-propanol=95/5, 0.5 mL/min, 210 nm). t_R 30.8 min (*S*); t_R 33.6 min (*R*). ^{1}H NMR ($CDCl_3$), δ: 1.53-1.57 (m, 1H), 1.83-1.95 (m, 3H), 2.73-2.78 (m, 2H), 2.82-2.84 (m, 1H), 3.39 (dd, 1H, *J*= 4.2 Hz, *J*= 13.3 Hz), 4.26-4.32 (m, 2H), 7.33 (d, 2H, *J*= 8.0 Hz), 7.56 (d, 2H, *J*= 8.0 Hz).

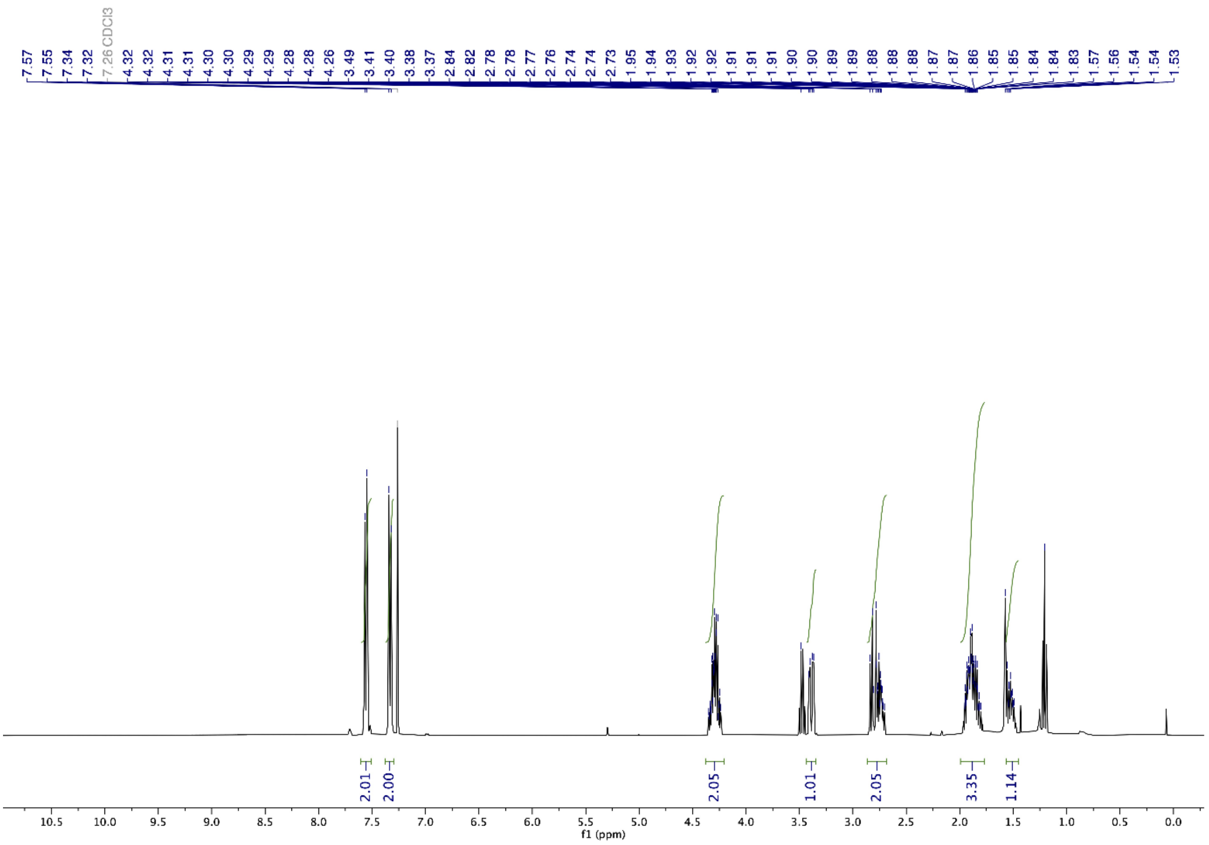

Figure S112. ^{1}H NMR spectrum of 3-(4-(trifluoromethyl)benzyl)tetrahydro-2*H*-pyran-2-one (from **S54**) in $CDCl_3$.

(a)

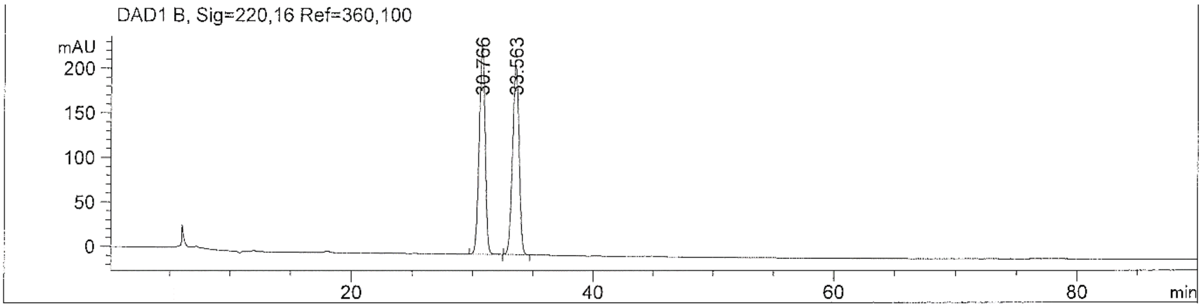

(b)

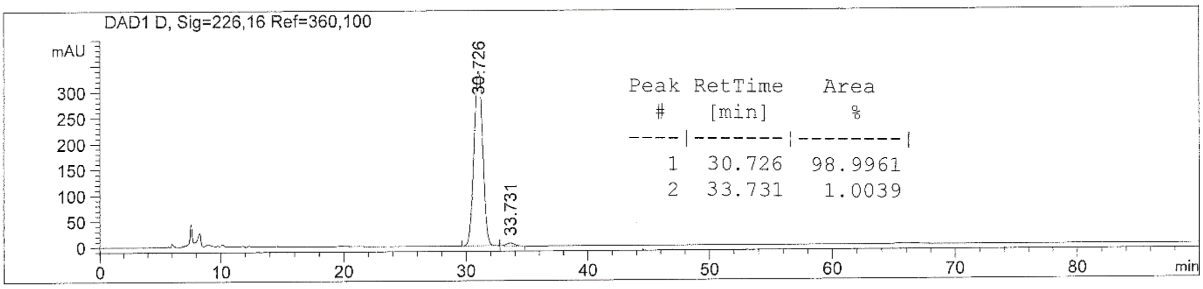

Figure S113. HPLC trace of: (a) racemic and (b) enantioenriched product (98% (*S*) ee using **4a** catalytic system; Scheme 4).

3-(3-Methoxybenzyl)tetrahydro-2H-pyran-2-one (from S55).[33] Enantiomeric excess determined by

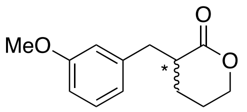

HPLC using Chiracel IA column (hexane/2- propanol=95/5, 0.5 mL/min, 210 nm). t_R 37.6 min (*S*); t_R 46.2 min (*R*). ^{1}H NMR ($CDCl_3$), δ: 1.52-1.56 (m, 1H), 1.81-1.91 (m, 3H), 2.65-2.72 (m, 2H), 3.34-3.49 (m, 1H), 3.80 (s, 3H), 4.26-4.31(m, 2H), 6.75-6.80 (m, 3H), 7.20-7.24 (m, 1H).

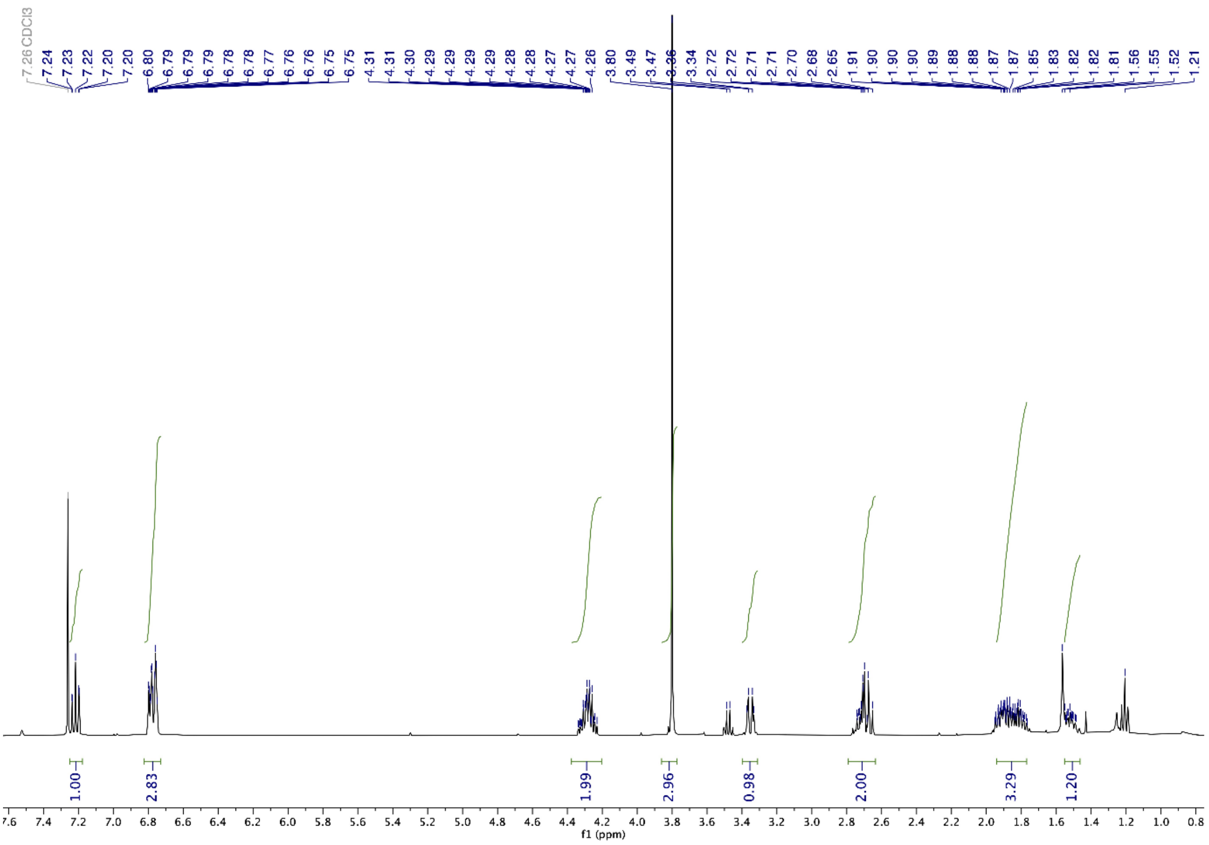

Figure S114. ^{1}H NMR spectrum of 3-(3-methoxybenzyl)tetrahydro-2*H*-pyran-2-one (from **S55**) in $CDCl_3$.

(a)

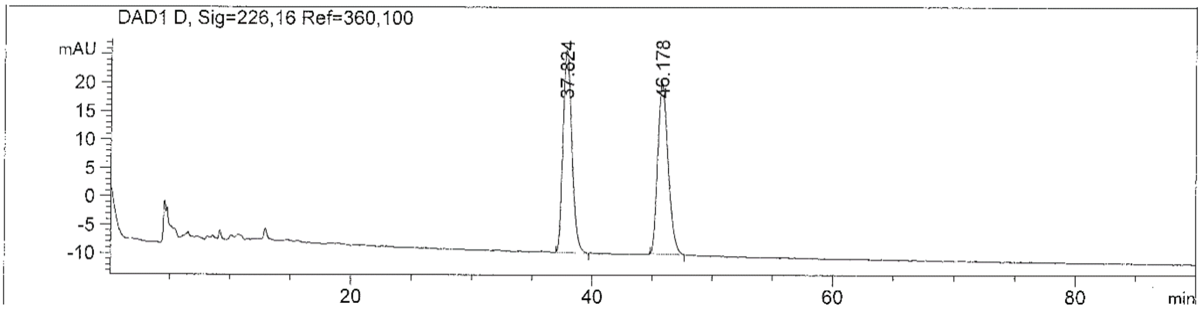

(b)

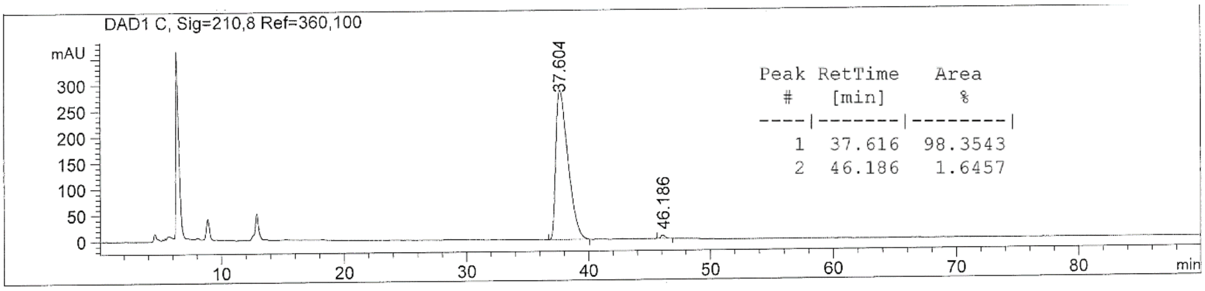

Figure S115. HPLC trace of: (a) racemic and (b) enantioenriched product (97% (*S*) ee using **4a** catalytic system; Scheme 4).

3-(2-Methoxybenzyl)tetrahydro-2H-pyran-2-one (from S56).[33] Enantiomeric excess determined by HPLC using Chiracel IA column (hexane/2-propanol=95/5, 0.5 mL/min, 210 nm). t_R 40.2 min (*S*); t_R 41.1 min (*R*). ^{1}H NMR ($CDCl_3$), δ: 1.48-1.53 (m, 1H), 1.77-1.90 (m, 3H), 2.66 (dd, 1H, *J*= 9.6 Hz, *J*= 13.5 Hz), 2.81-2.88 (m, 1H), 3.39-3.44 (dd, 1H, *J*= 4.6 Hz, *J*= 13.5 Hz), 3.77 (s, 3H), 4.26-4.29 (m, 2H), 6.84-6.90 (m, 2H), 7.15 (dd, 1H, *J*= 1.7 Hz, *J*= 7.4 Hz, 1H), 7.21 (dd, 1H, *J*= 1.8 Hz, *J*= 7.8 Hz).

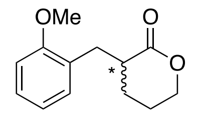

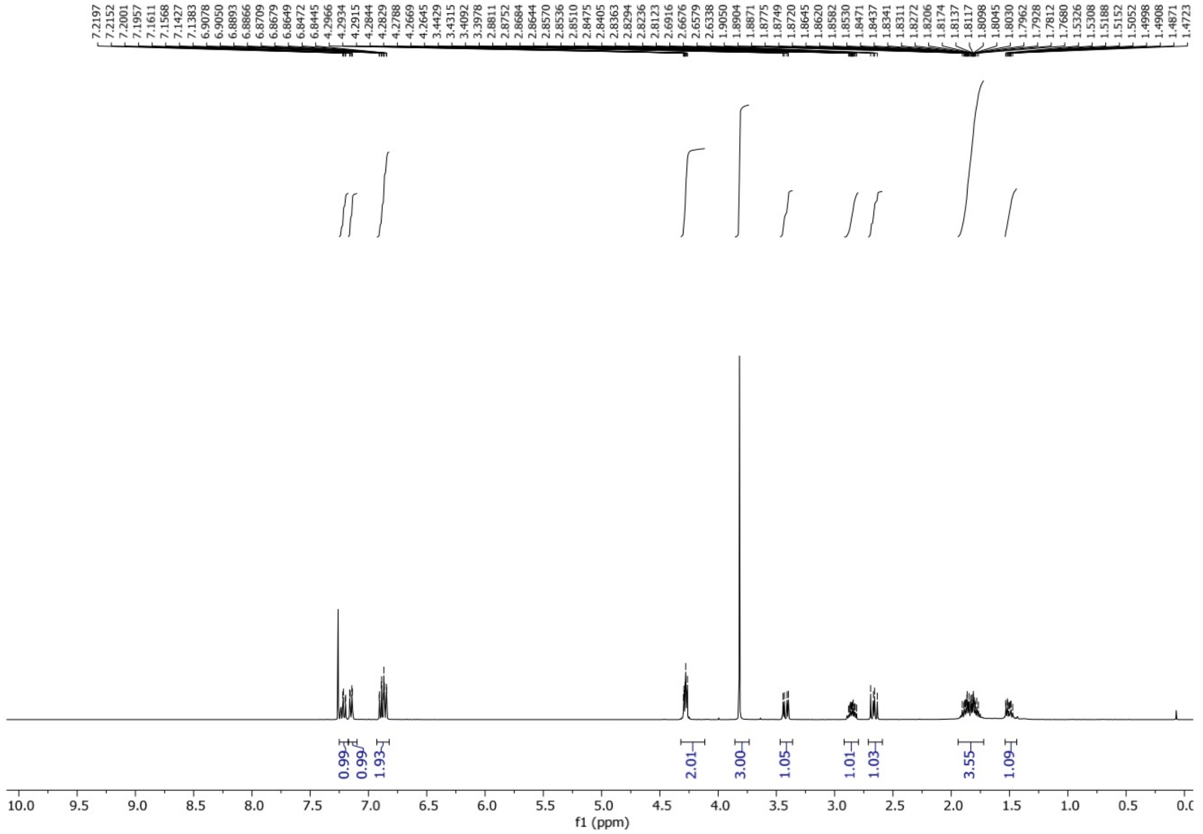

Figure S116. ^{1}H NMR spectrum of 3-(2-methoxybenzyl)tetrahydro-2*H*-pyran-2-one (from **S56**) in $CDCl_3$.

(a)

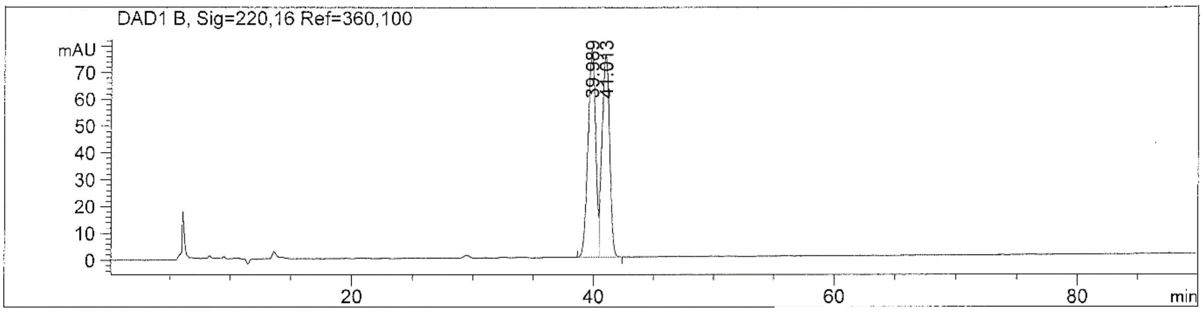

(b)

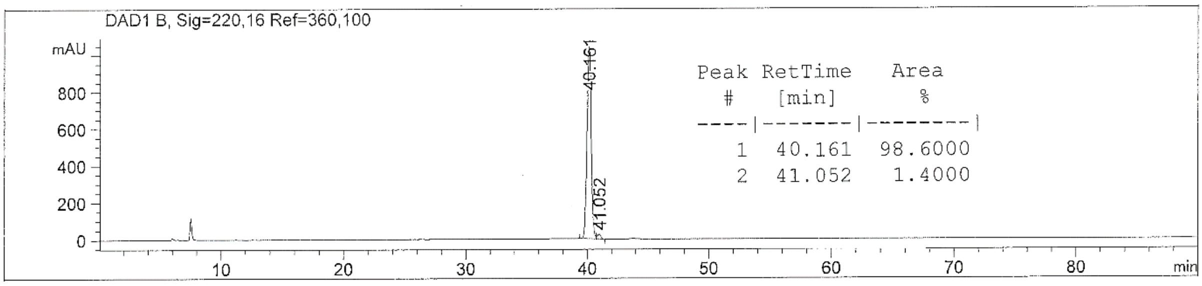

Figure S117. HPLC trace of: (a) racemic and (b) enantioenriched product (97% (*S*) ee using **4a** catalytic system; Scheme 4).

3-Benzyldihydrofuran-2(3H)-one (from S57).[32] Enantiomeric excess determined by HPLC using

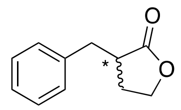

Chiracel OD-H column (hexane/2-propanol=90/10, 1 mL/min, 210 nm). t_R 18.3 min (*S*); t_R 19.4 min (*R*). ^{1}H NMR ($CDCl_3$), δ: 1.97-2.05 (m, 1H), 2.23-2.27 (m, 1H), 2.75 (dd, 1H, *J*= 9.5 Hz, *J*= 13.4 Hz), 2.81-2.89 (m, 1H), 3.26 (dd, 1H, *J*= 3.5 Hz, *J*= 13.4 Hz), 4.12-4.26 (m, 2H), 7.19-7.33 (m, 5H).

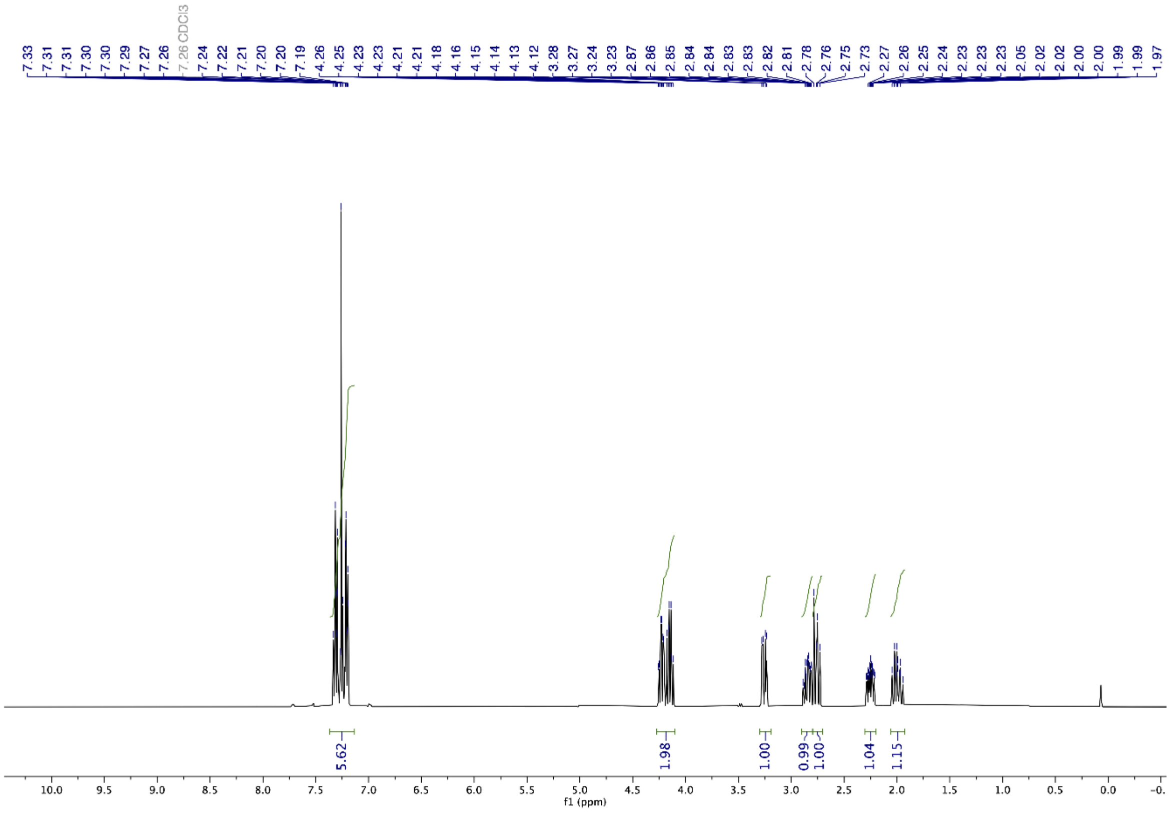

Figure S118. ^{1}H NMR spectrum of 3-benzyldihydrofuran-2(3*H*)-one (from **S57**) in $CDCl_3$.

(a)

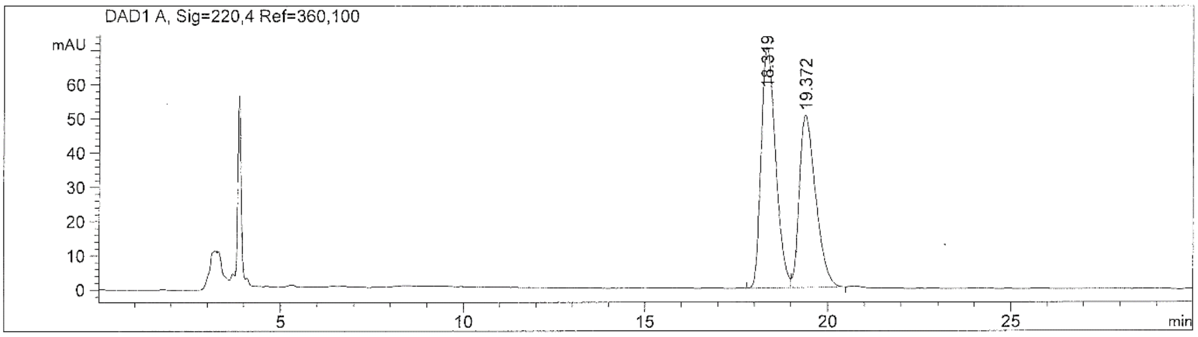

(b)

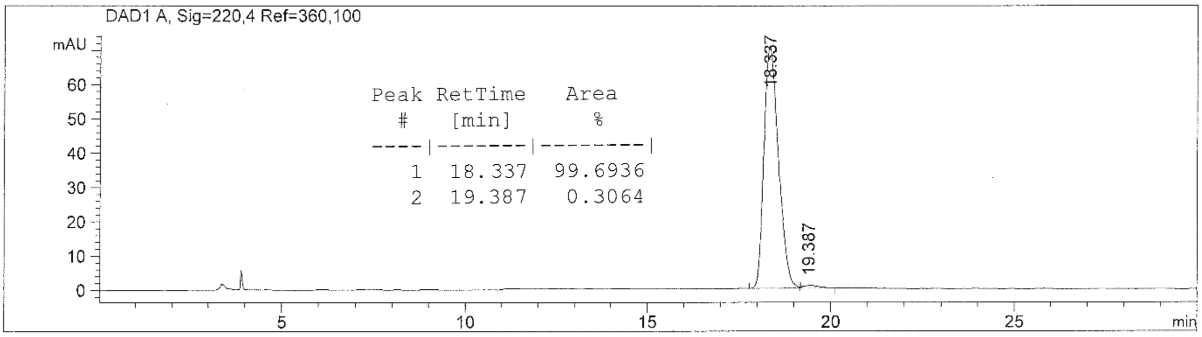

Peak #	RetTime [min]	Area %
1	18.337	99.6936
2	19.387	0.3064

Figure S119. HPLC trace of: (a) racemic and (b) enantioenriched product (99% (*S*) ee using **4a** catalytic system; Scheme 4).

3-(4-Methoxybenzyl)dihydrofuran-2(3H)-one (from S58).[32] Enantiomeric excess determined by

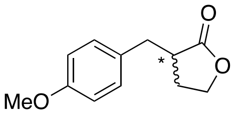

HPLC using Chiracel OD-H column (hexane/2-propanol=90/10, 1 mL/min, 210 nm). t_R 11.8 min (*S*); t_R 12.7 min (*R*). ^{1}H NMR ($CDCl_3$), δ: 1.98-2.05 (m, 1H), 2.23-2.27 (m, 1H), 2.73-2.83 (m, 2H), 3.15-3.19 (m, 1H), 3.80 (s, 3H), 4.13-4.24 (m, 2H), 6.85 (d, 2H, *J*= 8.4 Hz), 7.13 (d, 2H, *J*= 8.4 Hz).

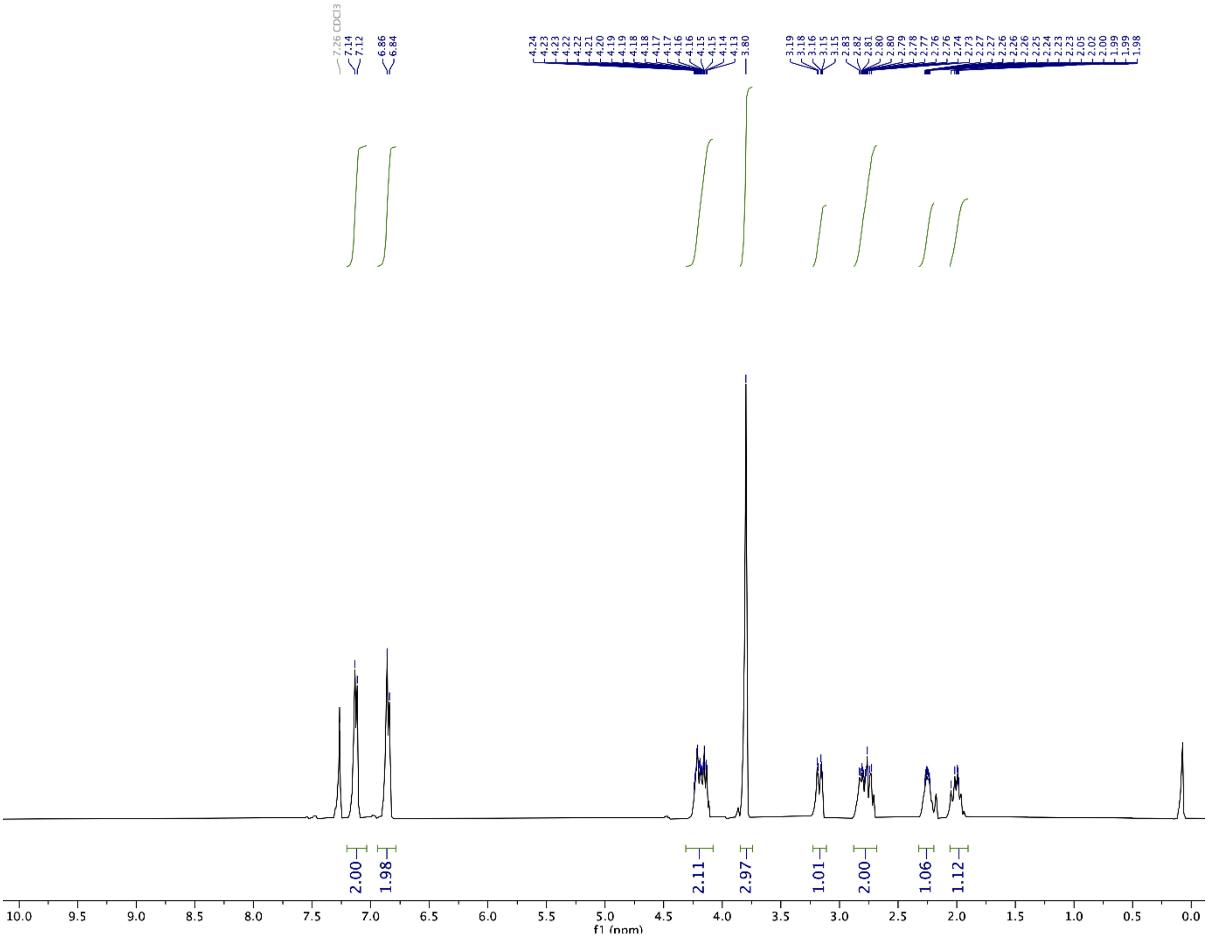

Figure S120. ^{1}H NMR spectrum of 3-(4-methoxybenzyl)dihydrofuran-2(3*H*)-one (from **S58**) in $CDCl_3$.

(a)

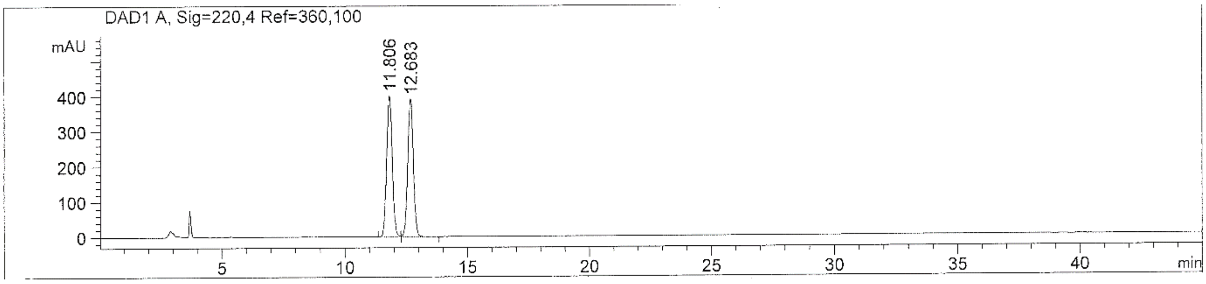

(b)

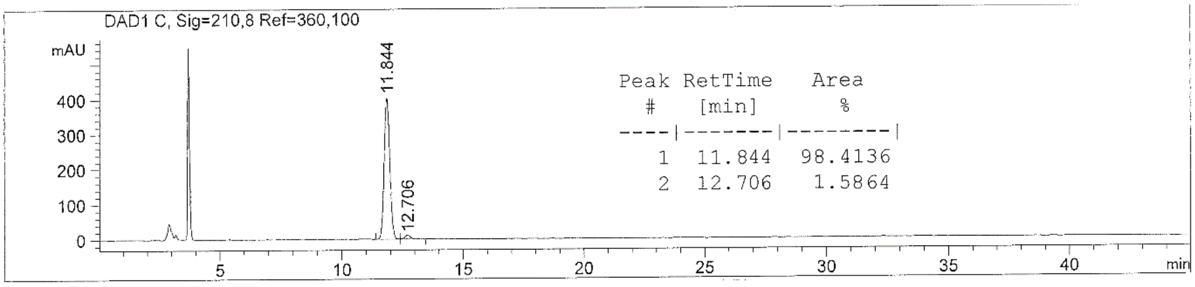

Figure S121. HPLC trace of enantioenriched product (97% (*S*) ee using **4a** catalytic system; Scheme 4).

3-(4-(Trifluoromethyl)benzyl)dihydrofuran-2(3H)-one (from S59). Enantiomeric excess determined

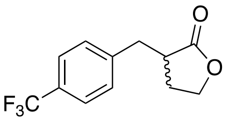

by HPLC using Chiracel OD-H column (hexane/2-propanol=90/10, 1 mL/min, 210 nm). t_R 9.6 min (*S*); t_R 10.4 min (*R*). ^1H NMR ($CDCl_3$), δ: 1.92-2.02 (m, 1H), 2.21-2.29 (m, 1H), 2.74-2.86 (m, 2H), 3.17-3.22 (m, 1H), 4.12-4.26 (m, 2H), 6.97-7.02 (m, 2H), 7.15-7.18 (m, 2H). ^{13}C NMR ($CDCl_3$), δ: 28.2, 35.3, 41.3, 66.7, 115.7 (d, *J*= 21.3 Hz), 130.5 (d, *J*= 7.9 Hz), 134.1 (d, *J*= 3.3 Hz), 160.7, 163.1, 178.7. HRMS-EI calculated for $C_{12}H_{11}F_3O_2$ [M+Na]+ : 267.0609, found:. 267.0605.

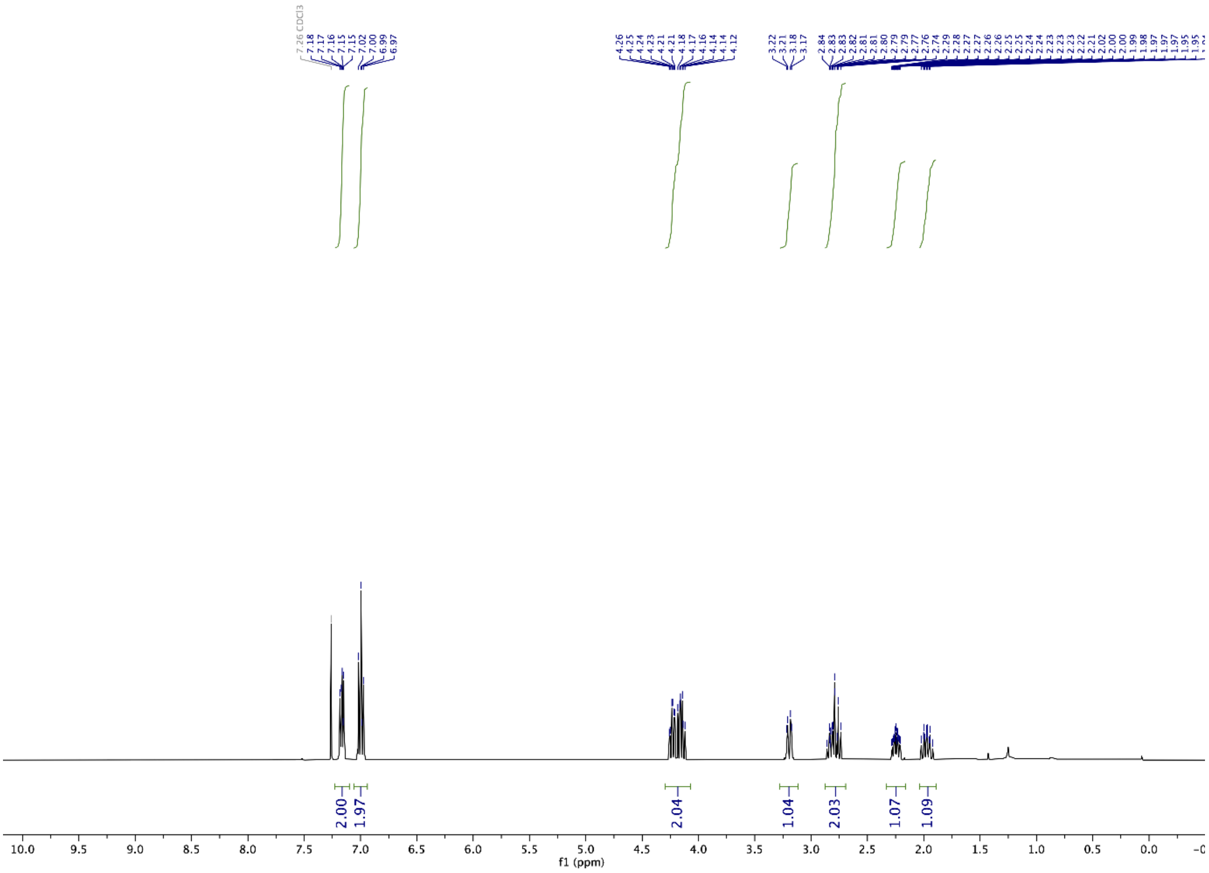

Figure S122. ^1H NMR spectrum of 3-(4-(trifluoromethyl)benzyl)dihydrofuran-2(3*H*)-one (from **S59**) in $CDCl_3$.

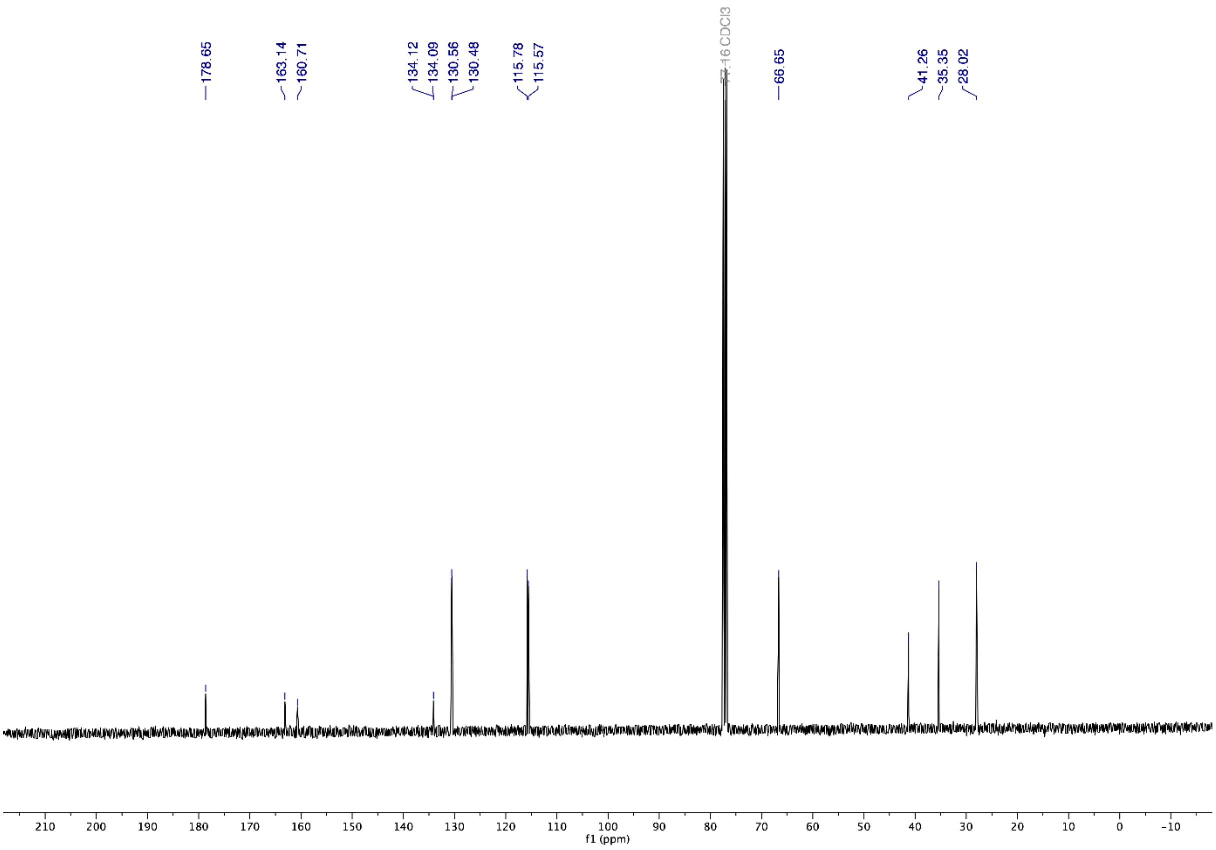

Figure S123. ^{13}C NMR spectrum of 3-(4-(trifluoromethyl)benzyl)dihydrofuran-2(3*H*)-one (from **S59**) in $CDCl_3$.

(a)

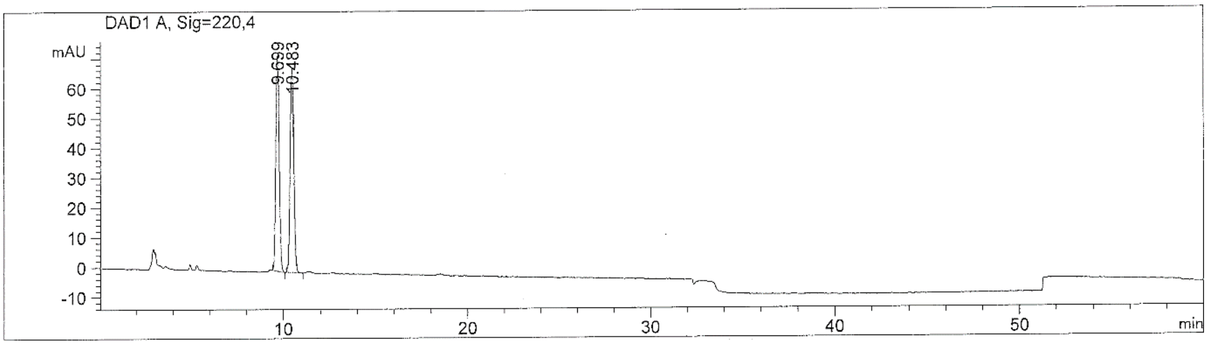

(b)

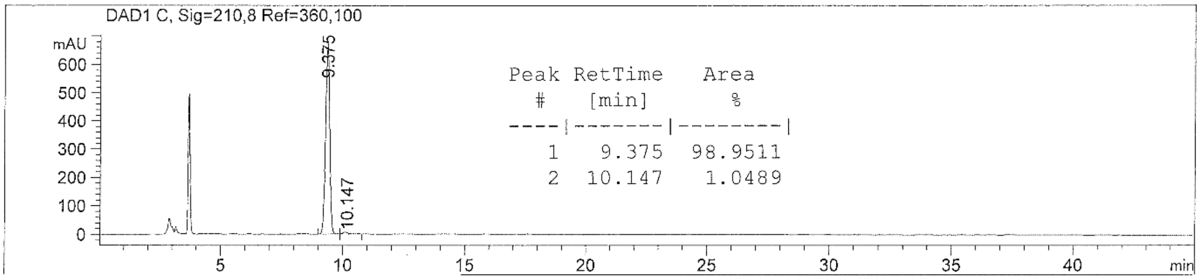

Figure S124. HPLC traces of (a) racemic and (b) enantioenriched product (98% (*S*) ee using **4a** catalytic system; Scheme 4).

1-Acetyl-3-benzylpiperidin-2-one (from S60).[34] Enantiomeric excess determined by HPLC using Chiracel OJ-H column (hexane/2-propanol=80/20, 0.5 mL/min, 210 nm). t_R 21.5 min (*S*); t_R 23.6 min (*R*). ^{1}H NMR ($CDCl_3$), δ: 1.45-1.53 (m, 1H), 1.68-1.76 (m, 1H), 1.81-1.90 (m, 2H), 2.54 (s, 3H), 2.65-2.75 (m, 2H), 3.35-3.39 (m, 1H), 3.59-3.64 (m, 1H), 3.81-3.88 (m, 1H), 7.19-7.33 (m, 5H).

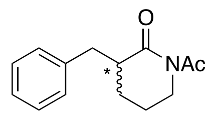

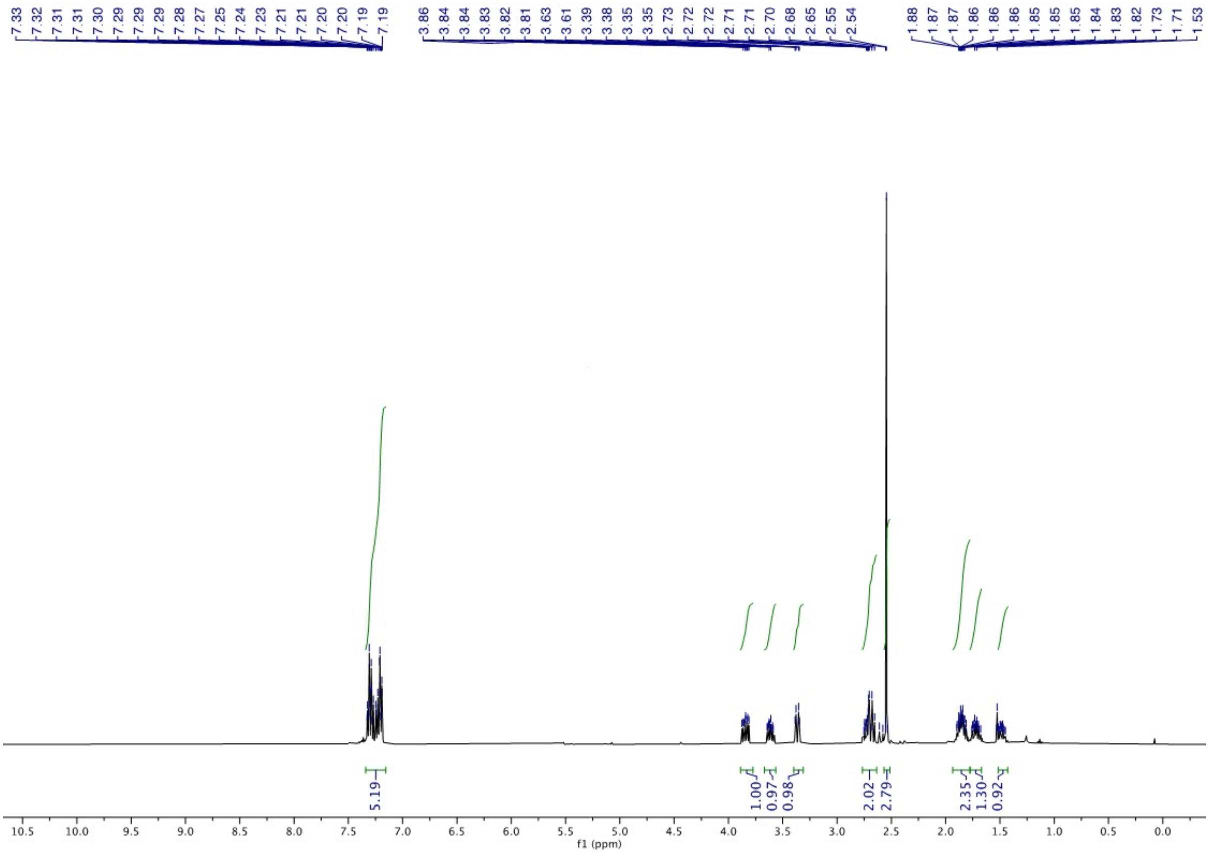

Figure S125. ^{1}H NMR spectrum of 1-acetyl-3-benzylpiperidin-2-one (from **S60**) in $CDCl_3$.

(a)

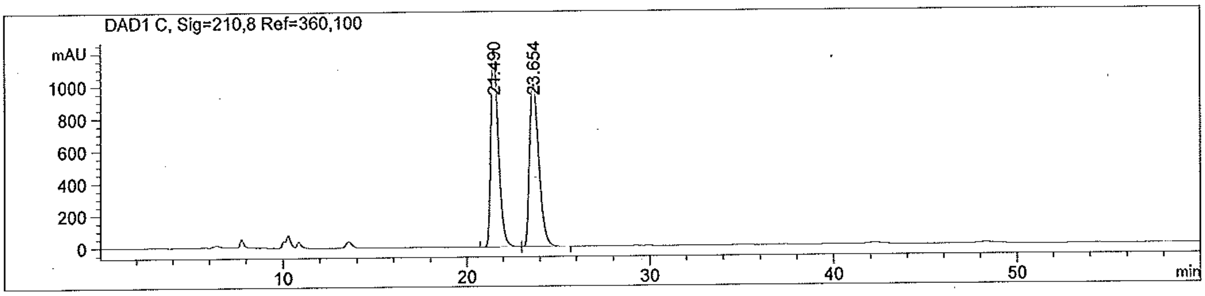

(b)

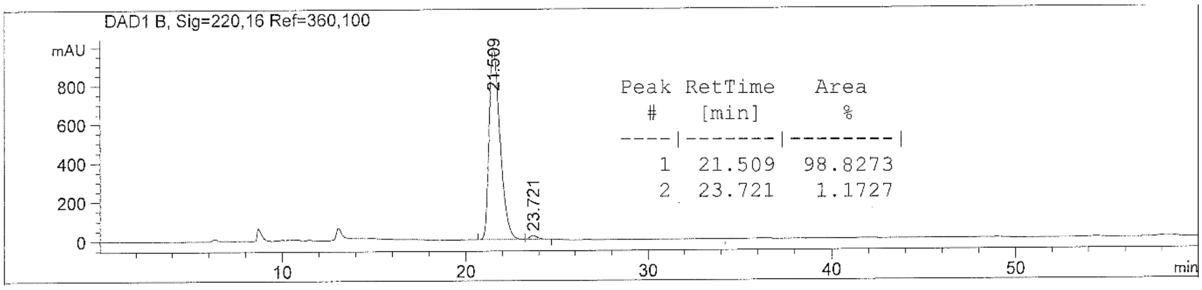

Figure S126. HPLC trace of: (a) racemic and (b) enantioenriched product (98% (*S*) ee using **4a** catalytic system; Scheme 4).

1,3-Dibenzylpiperidin-2-one (from S61).[34] Enantiomeric excess determined by HPLC using Chiracel

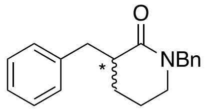

OJ-H column (hexane/2-propanol=80/20, 0.5 mL/min, 220 nm). t_R 14.1 min (-); t_R 21.0 min (+). ^{1}H NMR ($CDCl_3$), δ: 1.47-1.53 (m, 1H), 1.64-1.69 (m, 1H), 1.72-1.82 (m, 2H), 2.65-2.70 (m, 1H), 2.76 (dd, 1H, *J*= 13.2 Hz, *J*= 10 Hz), 3.17-3.20 (m, 2H), 3.45 (dd, 1H, *J*= 13.2 Hz, *J*= 3.6 Hz), 4.63 (m, 2H), 7.22-7.35 (m, 10H).

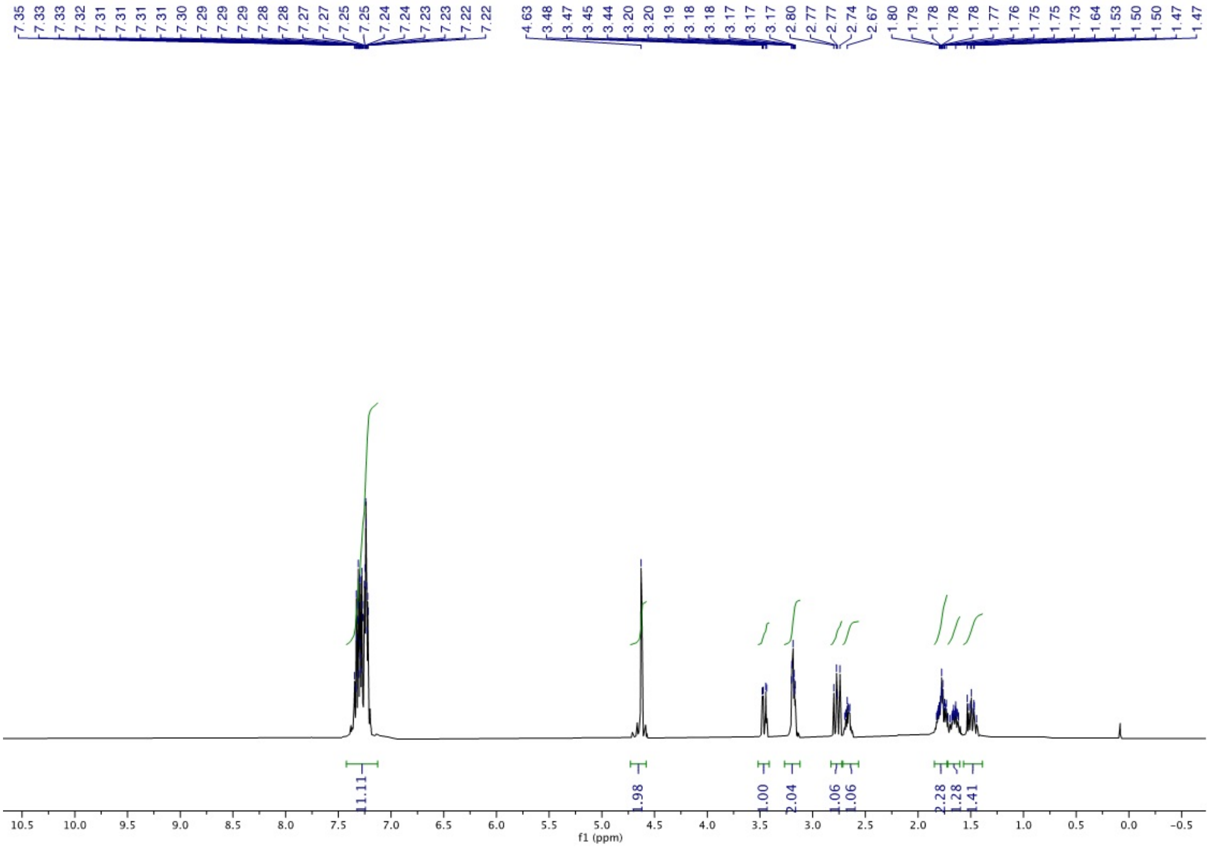

Figure S127. ^{1}H NMR spectrum of 1,3-dibenzylpiperidin-2-one (from **S61**) in $CDCl_3$.

(a)

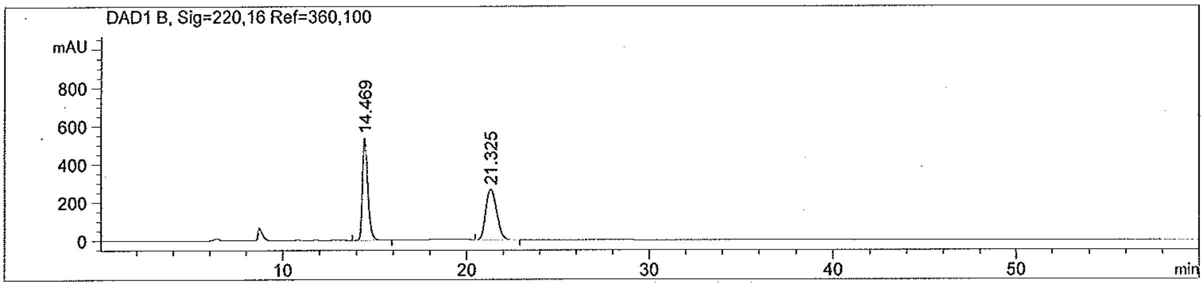

(b)

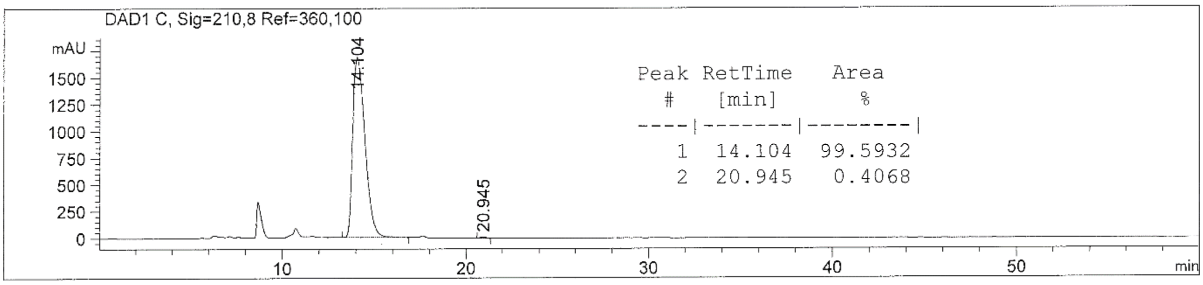

Figure S128. HPLC trace of: (a) racemic and (b) enantioenriched product (99% (*S*) ee using **4a** catalytic system; Scheme 4).

tert-Butyl 3-benzyl-2-oxopiperidine-1-carboxylate (from S62).[34] Enantiomeric excess determined by

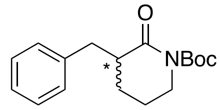

HPLC using Chiracel IA column (hexane/2-propanol=90/10, 0.5 mL/min, 220 nm). t_R 11.8 min (*S*); t_R 12.6 min (*R*). ^{1}H NMR ($CDCl_3$), δ: 1.54 (s, 9H), 1.80-1.82 (m, 3H), 2.61-2.65 (m, 2H), 3.40-3.48 (m, 2H), 3.57-3.60 (m, 1H), 3.68-3.71 (m, 1H), 7.13-7.31 (m, 5H).

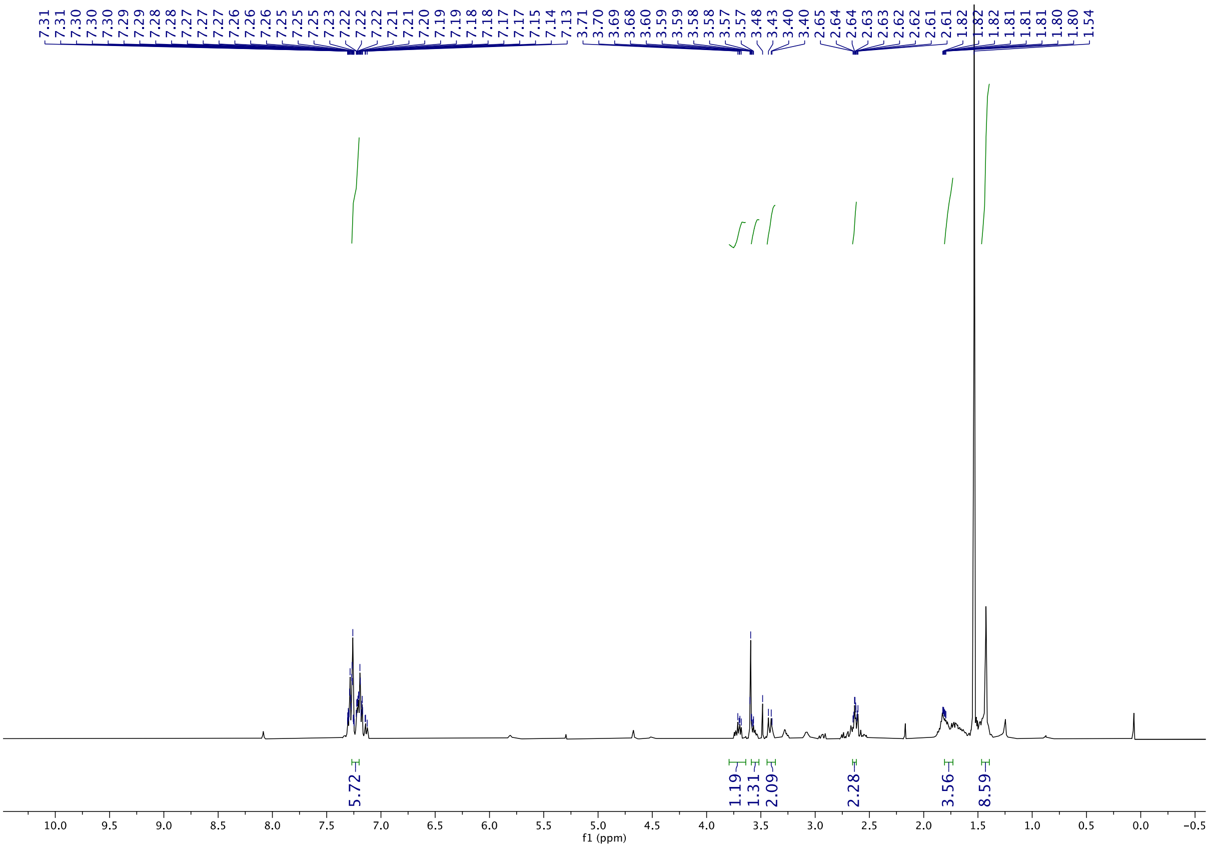

Figure S129. ^{1}H NMR spectrum of tert-butyl 3-benzyl-2-oxopiperidine-1-carboxylate (from **S62**) in $CDCl_3$.The signal at 1.5 ppm corresponds to water in the $CDCl_3$ and the minor signals correspond to the hydrogenated deprotected lactam.

(a)

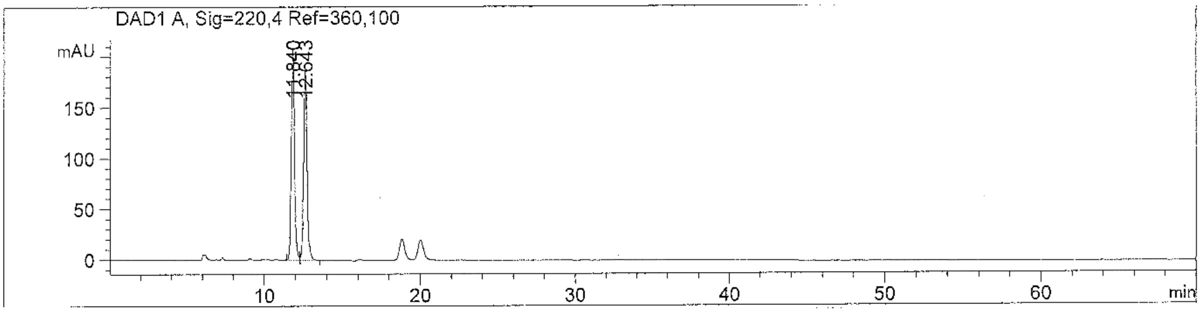

(b)

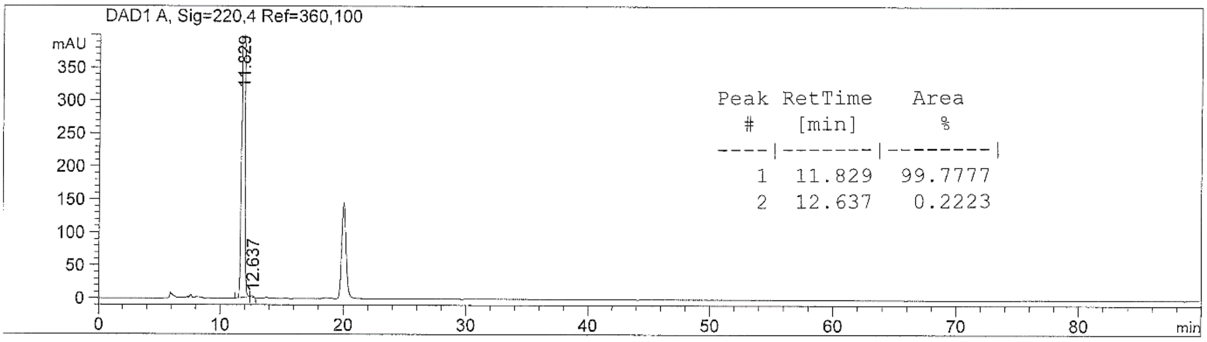

Peak #	RetTime [min]	Area %
1	11.829	99.7777
2	12.637	0.2223

Figure S130. HPLC trace of: (a) racemic and (b) enantioenriched product (>99% (*S*) ee using **4a** catalytic system; Scheme 4). The signals at ca 20 min correspond to the hydrogenated deprotected lactam.

1-Benzyl-3-(4-methoxybenzyl)piperidin-2-one (from S63).[33] Enantiomeric excess determined by

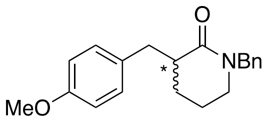

HPLC using Chiracel IA column (hexane/2- propanol=95/5, 0.5 mL/min, 210 nm). t_R 51.9 min (*S*); t_R 60.8 min (*R*). ^{1}H NMR ($CDCl_3$), δ: 1.43-1.51 (m, 1H), 1.61-1.66 (m, 1H), 1.72-1.78 (m, 2H), 2.59-2.63 (m, 1H), 2.75 (dd, 1H, *J*= 13.6 Hz, *J*= 9.7 Hz), 3.14-3.18 (m, 2H), 3.34 (dd, 1H, *J*= 13.6 Hz, *J*= 3.9 Hz), 3.79 (s, 3H), 4.58 (d, 1H, *J*= 14.6 Hz), 4.64 (d, 1H, *J*= 14.6 Hz), 6.80-6.84 (m, 2H), 7.12-7.14 (m, 2H), 7.21-7.33 (m, 5H).

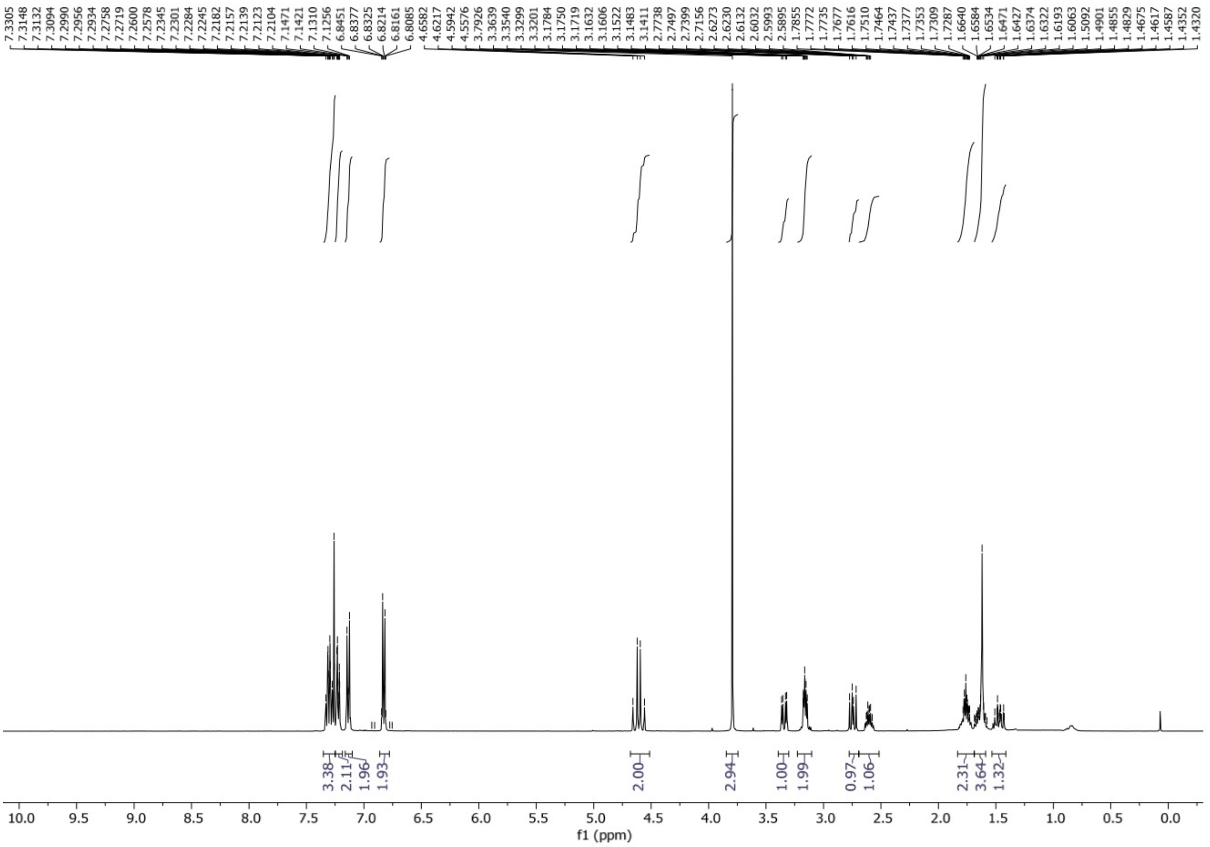

Figure S131. ^{1}H NMR spectrum of 1-benzyl-3-(4-methoxybenzyl)piperidin-2-one (from **S63**) in $CDCl_3$ (signal at 1.51 ppm corresponds to water).

(a)

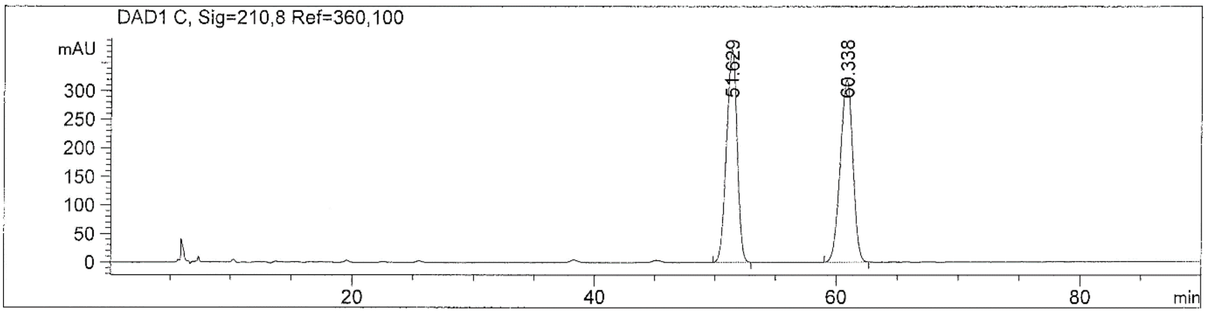

(b)

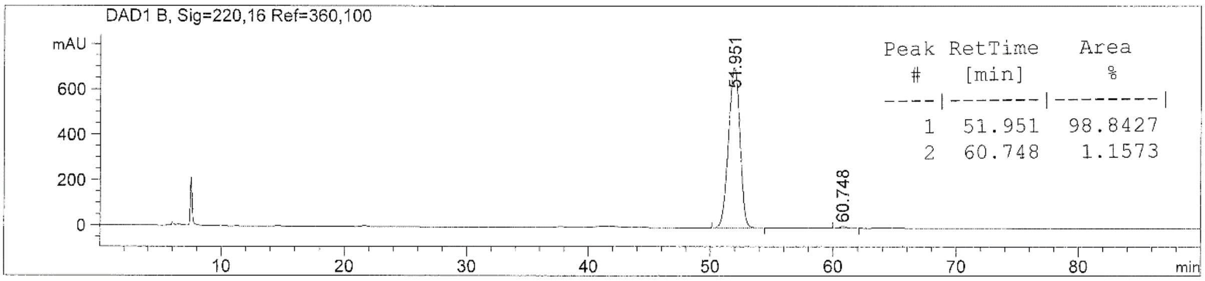

Figure S132. HPLC trace of: (a) racemic and (b) enantioenriched product (98% (*S*) ee using**4a** catalytic system; Scheme 4).

1-Benzyl-3-(4-bromobenzyl)piperidin-2-one (from S64).[33] Enantiomeric excess determined by HPLC

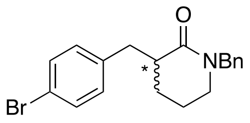

using Chiracel IA column (hexane/2- propanol=95/5, 0.5 mL/min, 210 nm). t_R 39.4 min (*S*); t_R 48.5 min (*R*). ^{1}H NMR ($CDCl_3$), δ: 1.41-1.48 (m, 1H), 1.62-1.68 (m, 1H), 1.73-1.80 (m, 2H), 2.59-2.64 (m, 1H), 2.81 (dd, 1H, *J*= 13.6 Hz, *J*= 9.3 Hz), 3.14-3.18 (m, 2H), 3.32 (dd, 1H, *J*= 13.6 Hz, *J*= 4.1 Hz), 4.57 (d, 1H, *J*= 14.6 Hz), 4.63 (d, 1H, *J*= 14.6 Hz), 7.09-7.11 (m, 2H), 7.19-7.21 (m, 3H), 7.26-7.32 (m, 2H), 7.40 (d, 2H, *J*= 8.4 Hz).

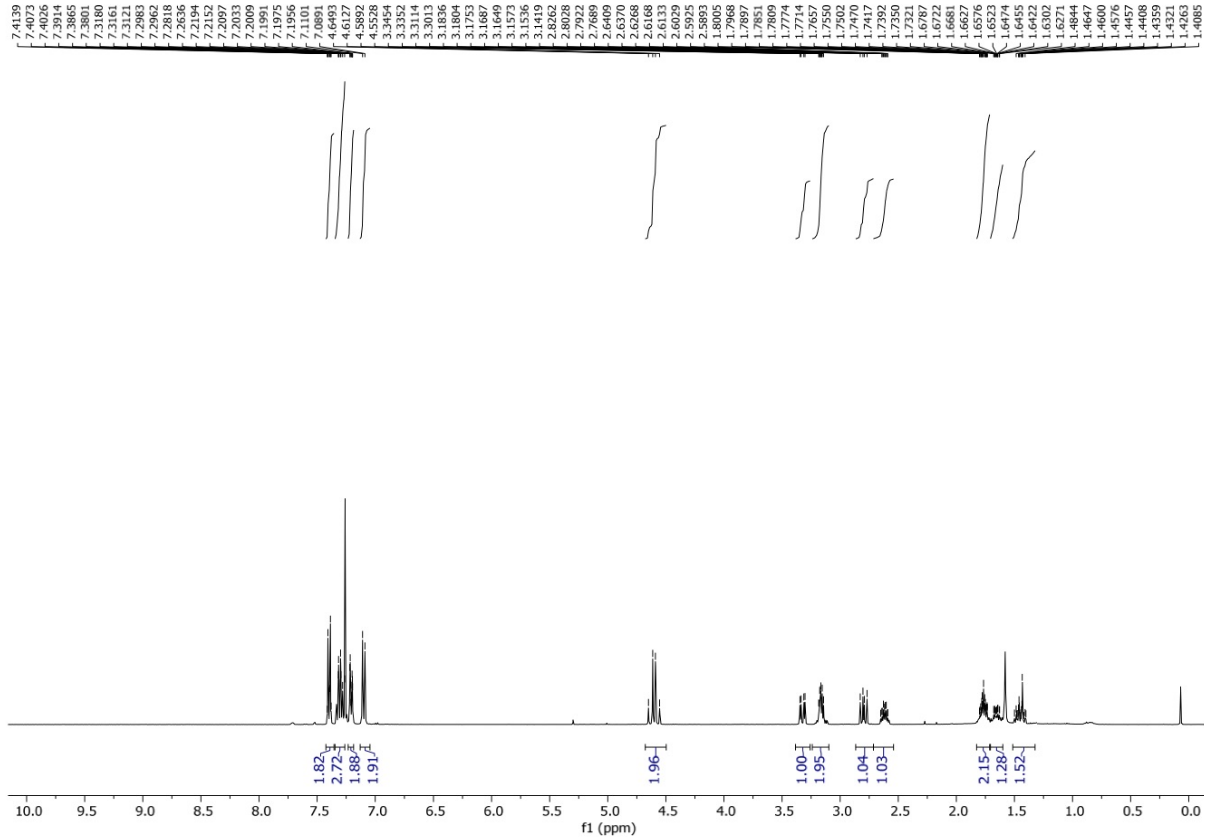

Figure S133. ^{1}H NMR spectrum of 1-benzyl-3-(4-bromobenzyl)piperidin-2-one (from **S64**) in $CDCl_3$.

(a)

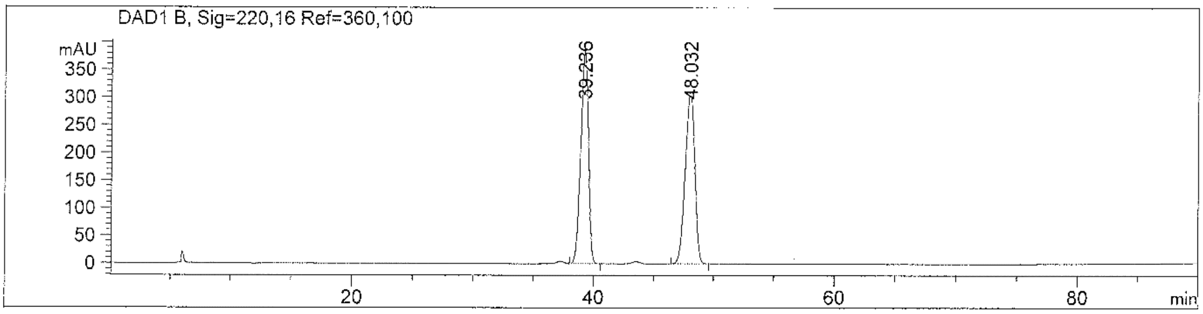

(b)

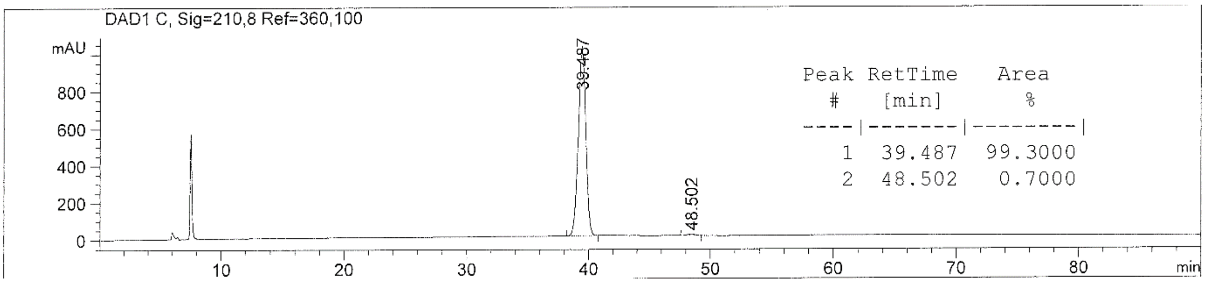

Figure S134. HPLC trace of: (a) racemic and (b) enantioenriched product (99% (*S*) ee using **4a** catalytic system; Scheme 4).

1-Benzyl-3-(3-methoxybenzyl)piperidin-2-one (from S65).[33] Enantiomeric excess determined by HPLC using Chiracel IA column (hexane/2- propanol=95/5, 0.5 mL/min, 210 nm). t_R 49.2 min (*S*); t_R 61.3 min (*R*). ^{1}H NMR ($CDCl_3$), δ: 1.43-1.51 (m, 1H), 1.63-1.65 (m, 2H), 1.73-1.79 (m, 1H), 2.63-2.65 (m, 1H), 2.73 (dd, 1H, *J*= 13.2 Hz, *J*= 10.1 Hz), 3.15-3.19 (m, 2H), 3.42-3.46 (m, 1H), 3.79 (s, 3H), 4.62 (s, 2H), 6.74-6.82 (m, 3H), 7.18-7.25 (m, 3H), 7.27-7.34 (m, 2H).

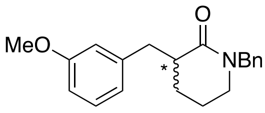

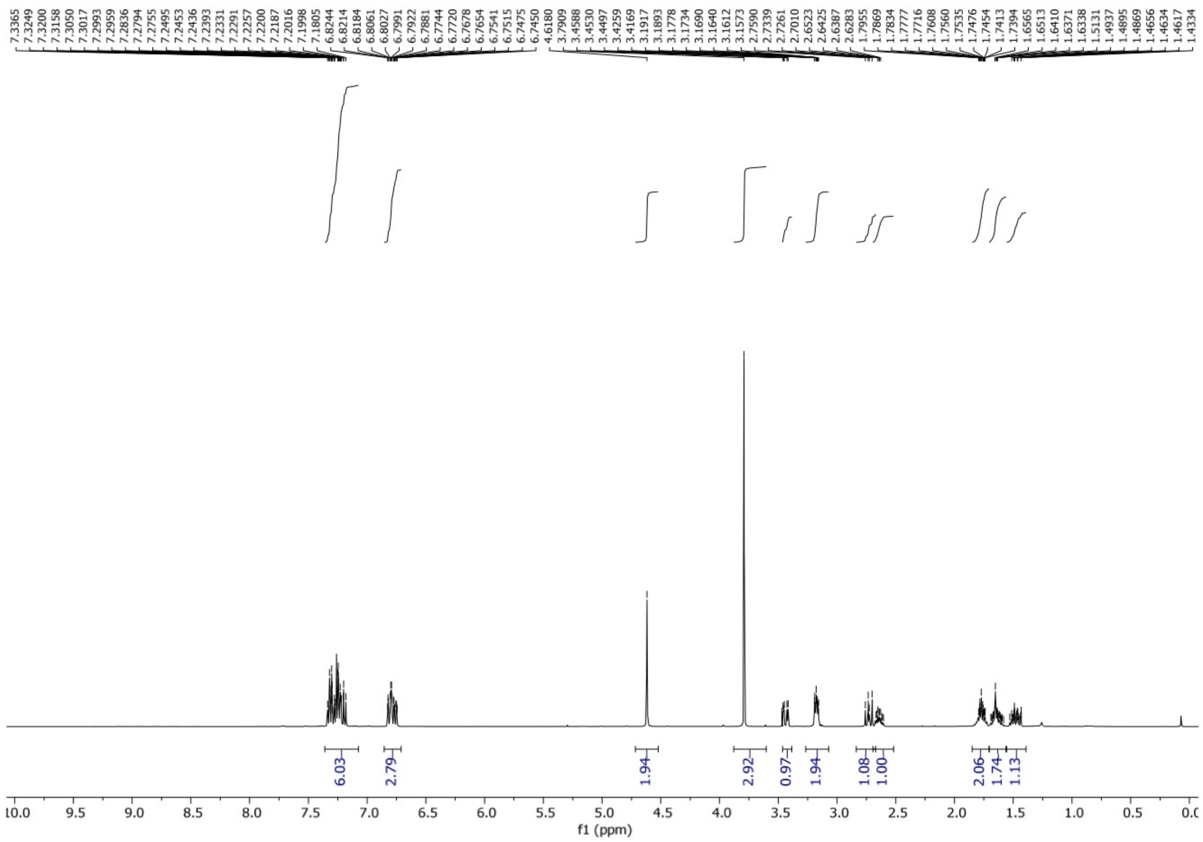

Figure S135. ^{1}H NMR spectrum of 1-benzyl-3-(3-methoxybenzyl)piperidin-2-one (from **S65**) in $CDCl_3$.

(a)

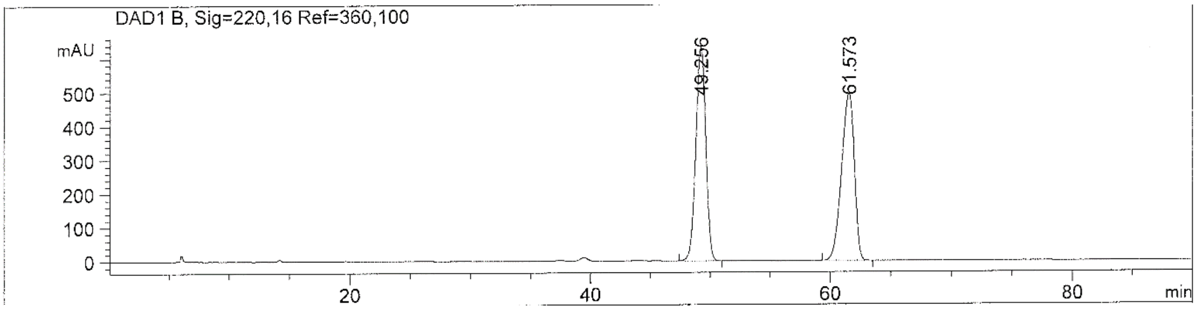

(b)

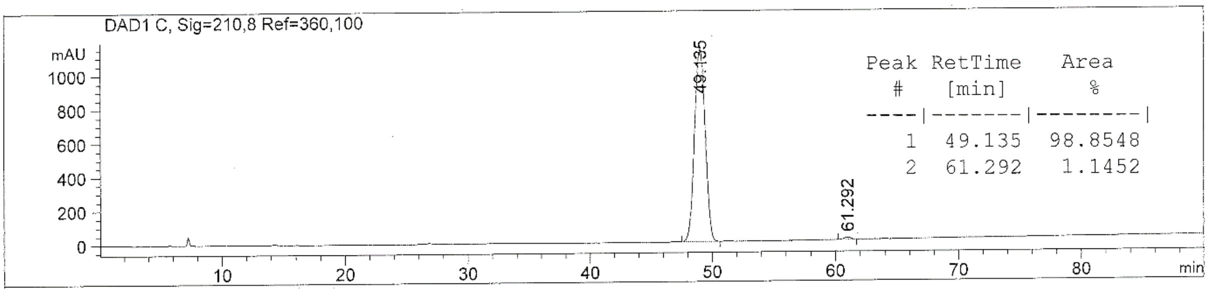

Peak #	RetTime [min]	Area %
1	49.135	98.8548
2	61.292	1.1452

Figure S136. HPLC trace of enantioenriched product (98% (*S*) ee using **4a** catalytic system; Scheme 4).

1-Benzyl-3-(2-methoxybenzyl)piperidin-2-one (from S66).[33] Enantiomeric excess determined by HPLC using Chiracel IA column (hexane/2- propanol=95/5, 0.5 mL/min, 210 nm). t_R 53.6 min (*S*); t_R 76.9 min (*R*). ^{1}H NMR ($CDCl_3$), δ: 1.43-1.47 (m, 1H), 1.59-1.67 (m, 2H), 1.79-1.83 (m, 1H), 2.70-2.74 (m, 2H), 3.18 (dd, 2H, *J*= 7.2 Hz, *J*= 5.1 Hz), 3.53 (d, 1H, *J*= 9.2 Hz), 3.82 (s, 3H), 4.59 (d, 1H, *J*= 14.6 Hz), 4.65 (d, 1H, *J*= 14.6 Hz), 6.84-6.90 (m, 2H), 7.16-7.22 (m, 2H), 7.24-7.28 (m, 3H), 7.30-7.35 (m, 2H).

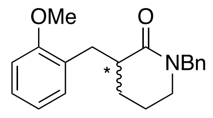

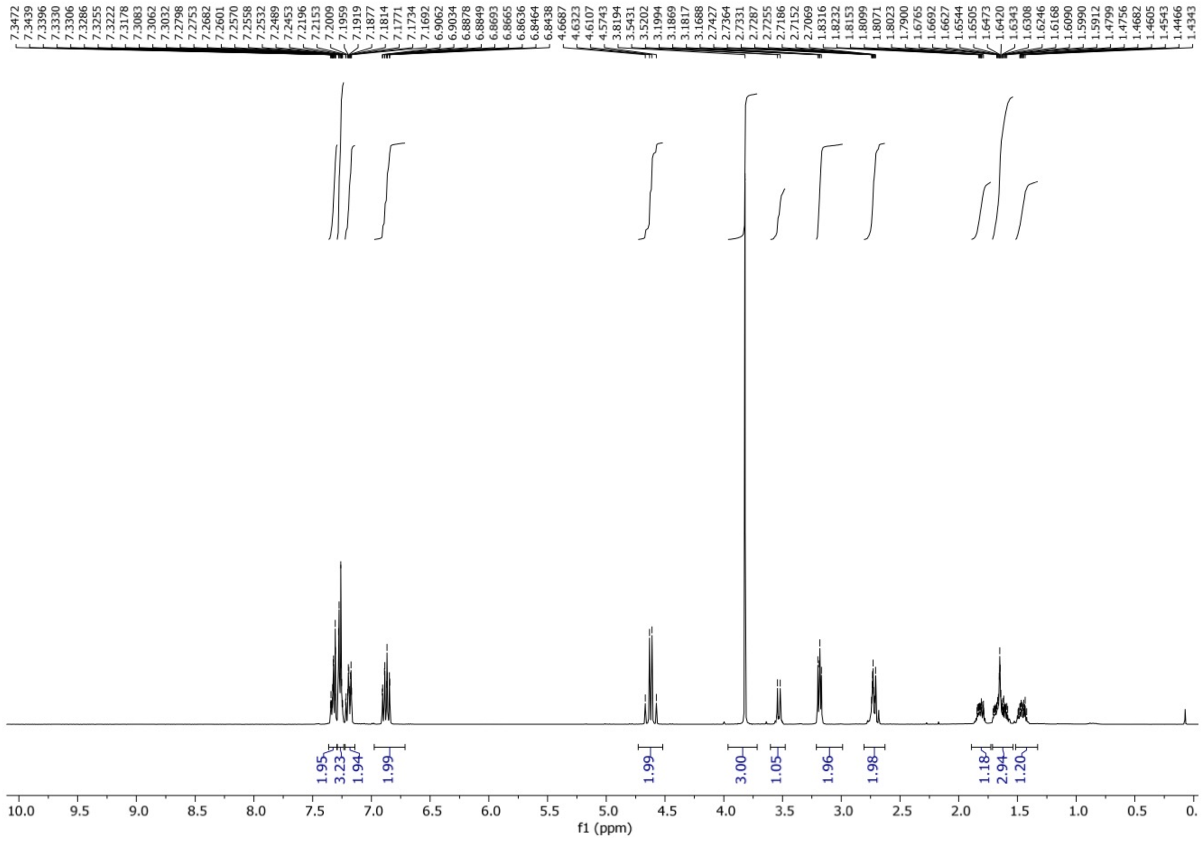

Figure S137. ^{1}H NMR spectrum of 1-benzyl-3-(2-methoxybenzyl)piperidin-2-one (from **S66**) in $CDCl_3$.

(a)

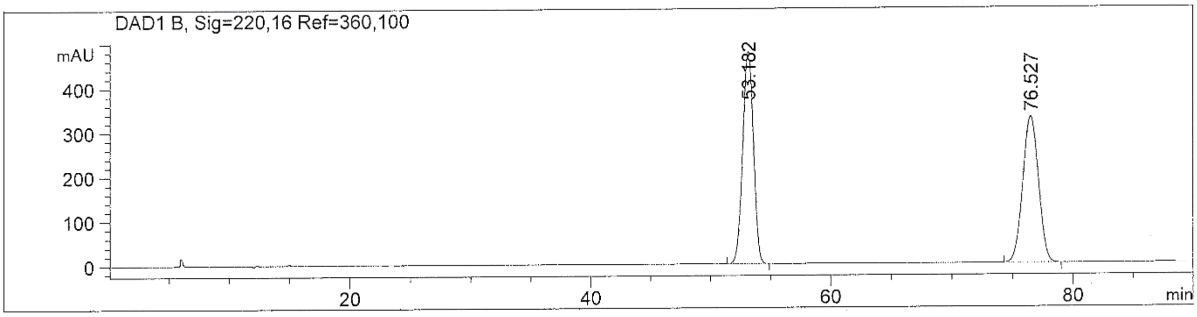

(b)

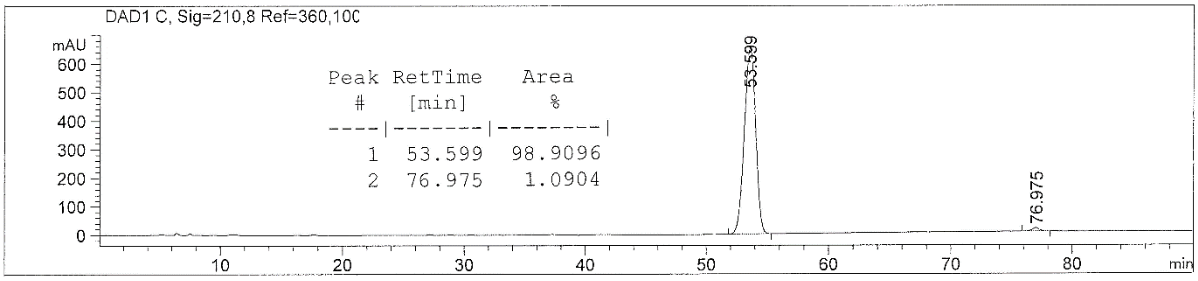

Figure S138. HPLC trace of: (a) racemic and (b) enantioenriched product (98% (*S*) ee using **4a** catalytic system; Scheme 4).

1-Benzyl-3-(cyclohexylmethyl)piperidin-2-one (from S67).[46] Enantiomeric excess determined by HPLC using Chiracel IA column (hexane/2-propanol=90/10, 0.5 mL/min, 210 nm). t_R 16.3 min (*S*); t_R 19.1 min (*R*). ^{1}H NMR ($CDCl_3$), δ: 0.88-1.04 (m, 2H), 1.13-1.41 (m, 5H), 1.44-1.53 (m, 1H), 1.63-1.77 (m, 6H), 1.79-1.87 (m, 1H), 1.88-1.96 (m, 2H), 2.41-2.48 (m, 1H), 3.17-3.20 (m, 2H), 4.51-4.63 (m, 2H), 7.22- 7.33 (m, 5H).

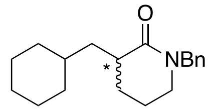

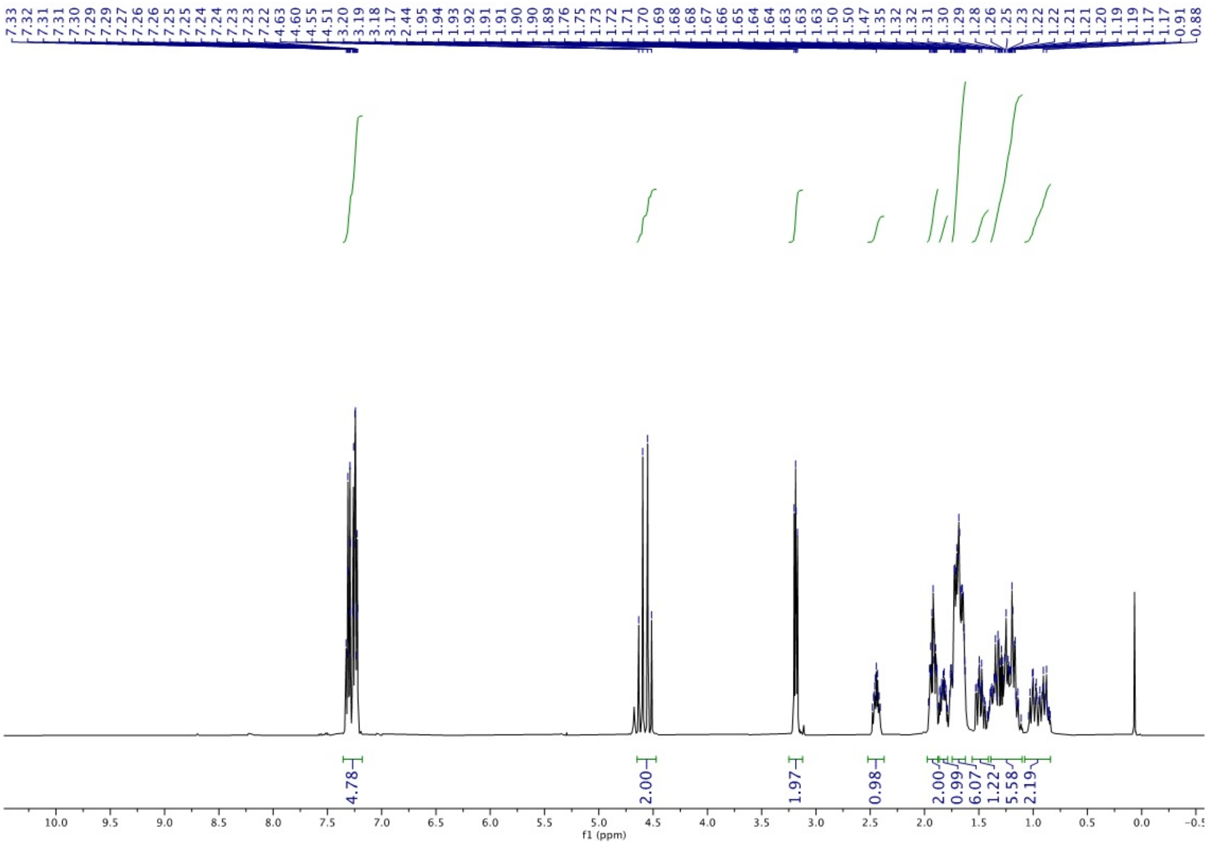

Figure S139. ^{1}H NMR spectrum of 1-benzyl-3-(cyclohexylmethyl)piperidin-2-one (from **S67**) in $CDCl_3$.

(a)

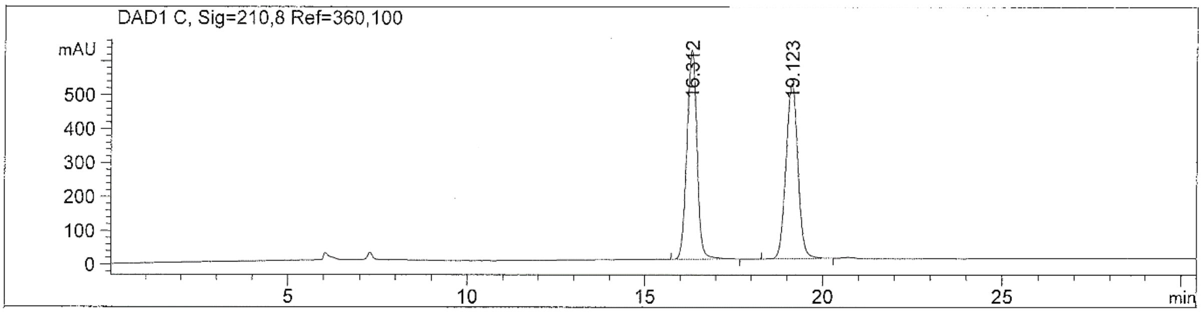

(b)

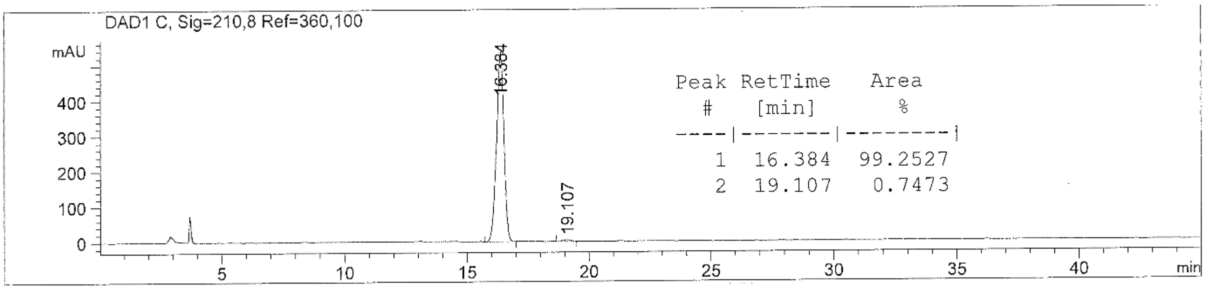

Figure S140. HPLC trace of: (a) racemic and (b) enantioenriched product (98% (*S*) ee using **4a** catalytic system; Scheme 4).

1,3-Dibenzyl-1λ⁴-pyrrolidin-2-one (from S68).[32] Enantiomeric excess determined by HPLC using

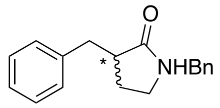

Chiracel AD column (hexane/2-propanol=90/10, 1 mL/min, 210 nm). t_R 11.1 min (*R*); t_R 12.4 min (*S*). ^{1}H NMR ($CDCl_3$), δ: 1.68-1.75 (m, 1H), 1.99-2.17 (m, 1H), 2.69-2.79 (m, 2H), 3.02-3.12 (m, 2H), 3.26 (dd, 1H, *J*= 2.8 Hz, *J*= 12.8 Hz), 4.45 (m, 2H), 7.18-7.32 (m, 10H).

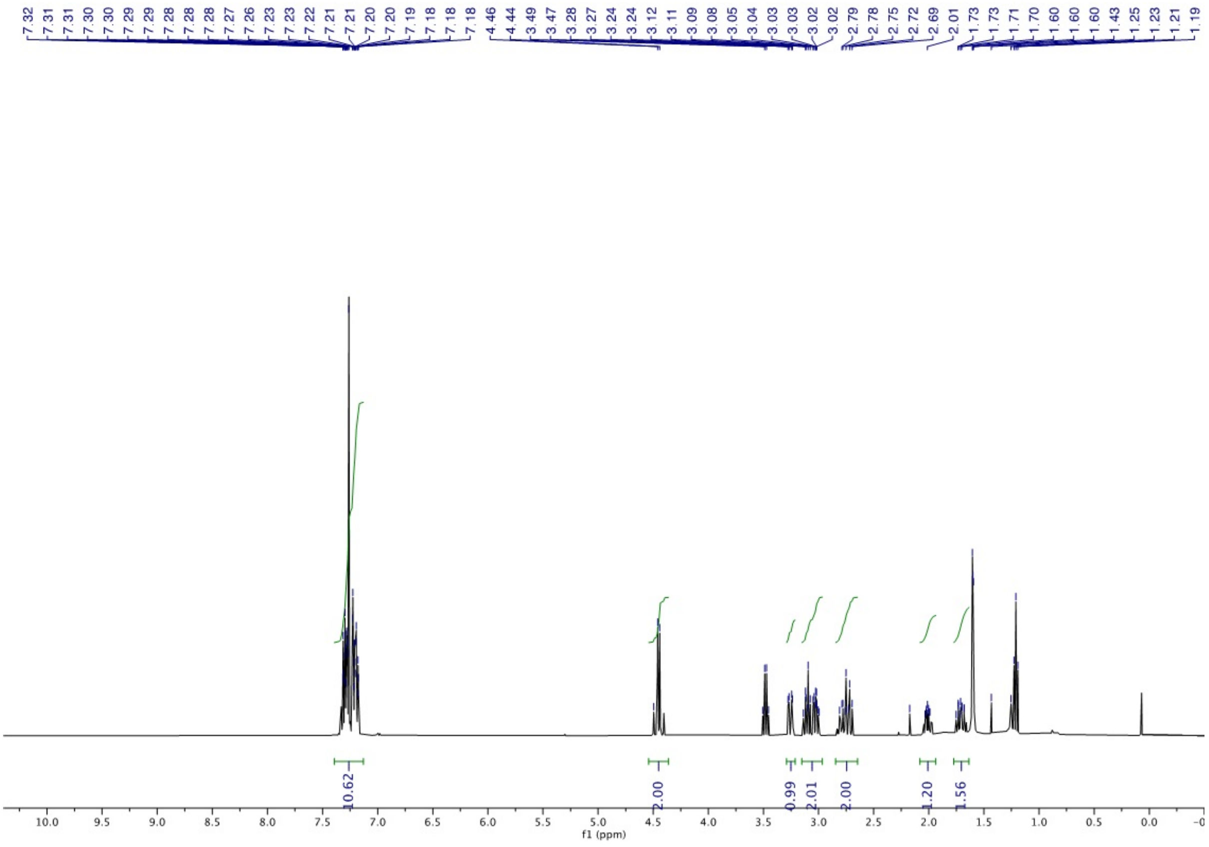

Figure S141. ^{1}H NMR spectrum of 1,3-dibenzyl-1λ^4-pyrrolidin-2-one (from **S68**) in $CDCl_3$ (signal at 1.60 ppm corresponds to water and signals at 1.21 and 3.47 ppm correspond to diethyl ether).

(a)

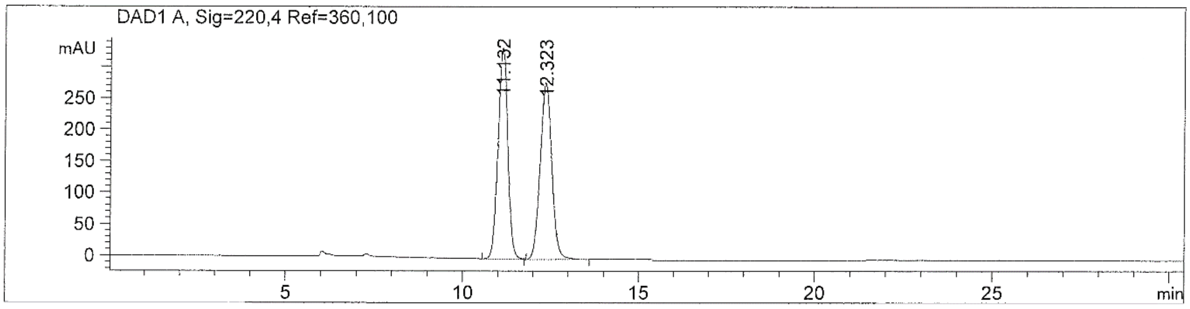

(b)

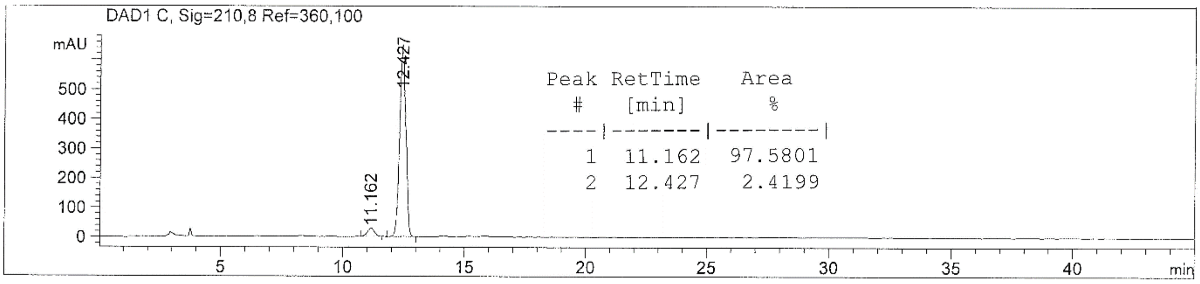

Figure S142. HPLC trace of: (a) racemic and (b) enantioenriched product (95% (*S*) ee using **4a** catalytic system; Scheme 4).

Ethyl 2,4,4,4-tetrafluoro-3-phenylbutanoate (from S70).[35] Enantiomeric excess determined by by GC

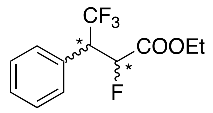

using Hydrodex B-3P column (100 kPa H_2, 80 °C isotherm). t_R 58.7 min (*S*,*R*); t_R 62.5 min (*R*,*S*). ^{1}H NMR ($CDCl_3$), δ: 1.15 (t, *J*= 7.2 Hz, 3H), 4.00 (ddd, 1H, *J*= 22.7 Hz, *J*= 8.8 Hz, *J*= 5.7 Hz), 4.14 (ddq, 2H, *J*= 10.8 Hz, *J*= 7.3 Hz, *J*= 3.7 Hz), 5.24 (dd, 1H, *J*= 47.7 Hz, *J*= 5.7 Hz), 7.39 (s, 5H).

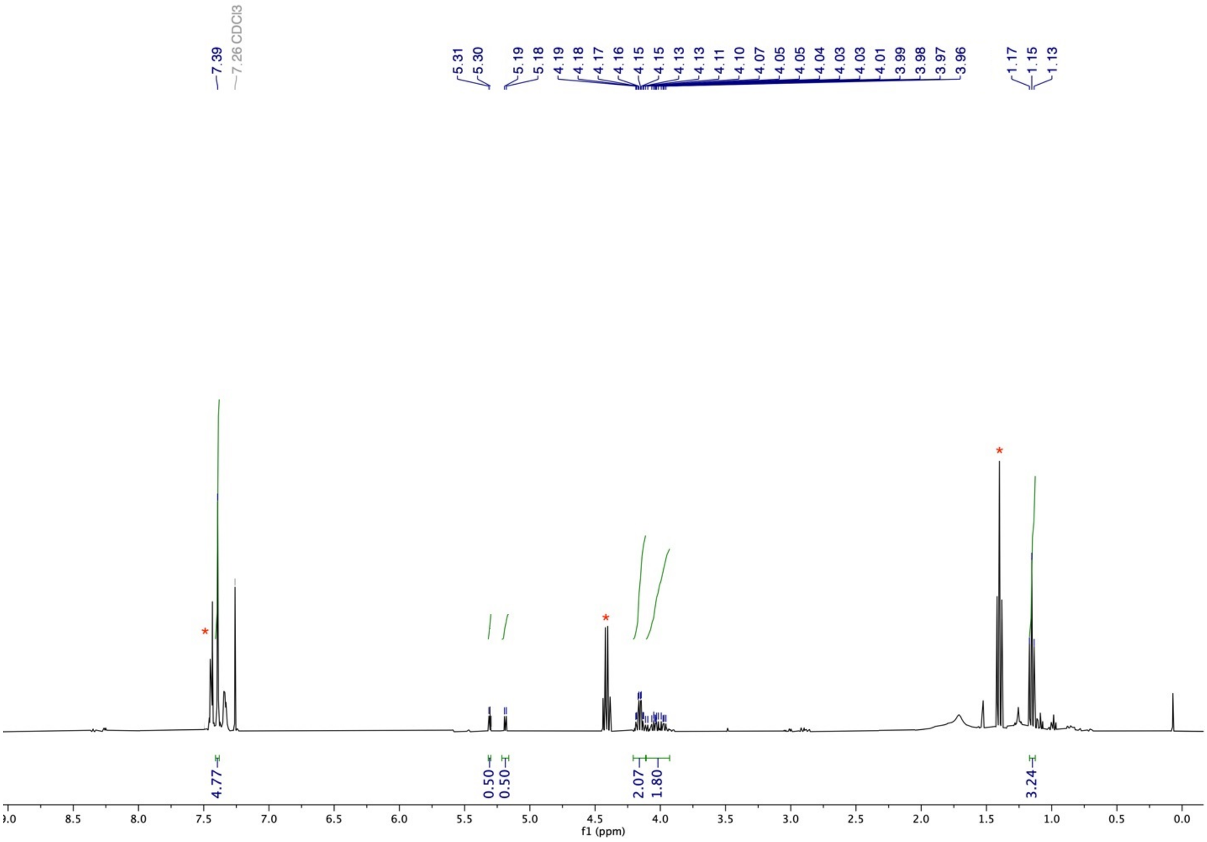

Figure S143. ^{1}H NMR spectrum of ethyl 2,4,4,4-tetrafluoro-3-phenylbutanoate (from **S70**) in $CDCl_3$ (signals marked with * correspond to starting material).

(a)

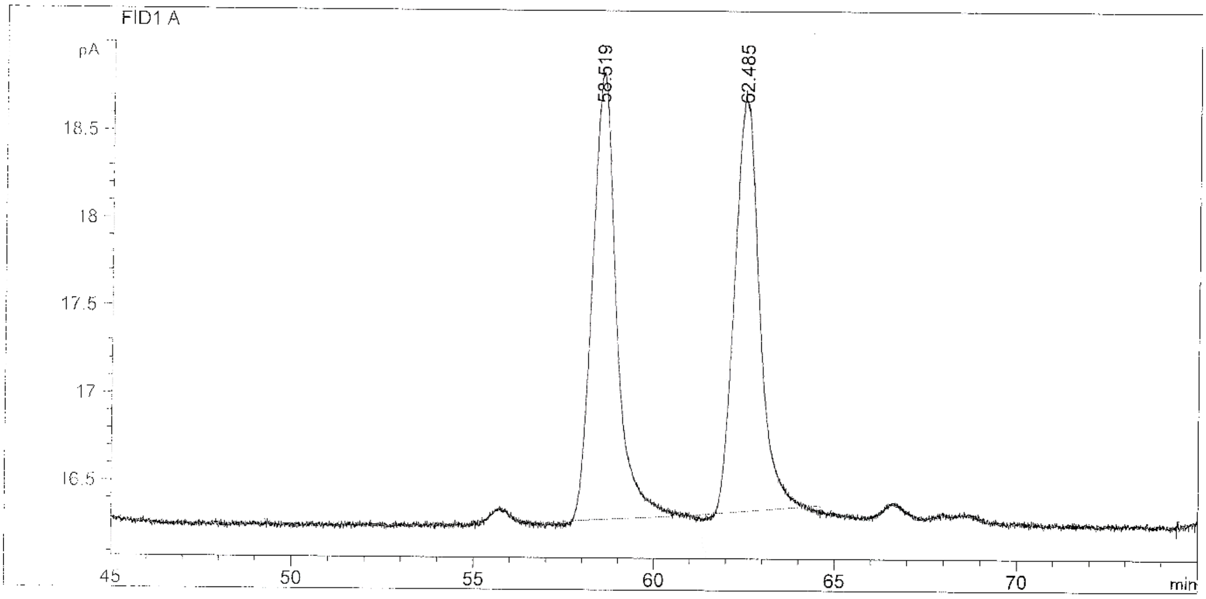

(b)

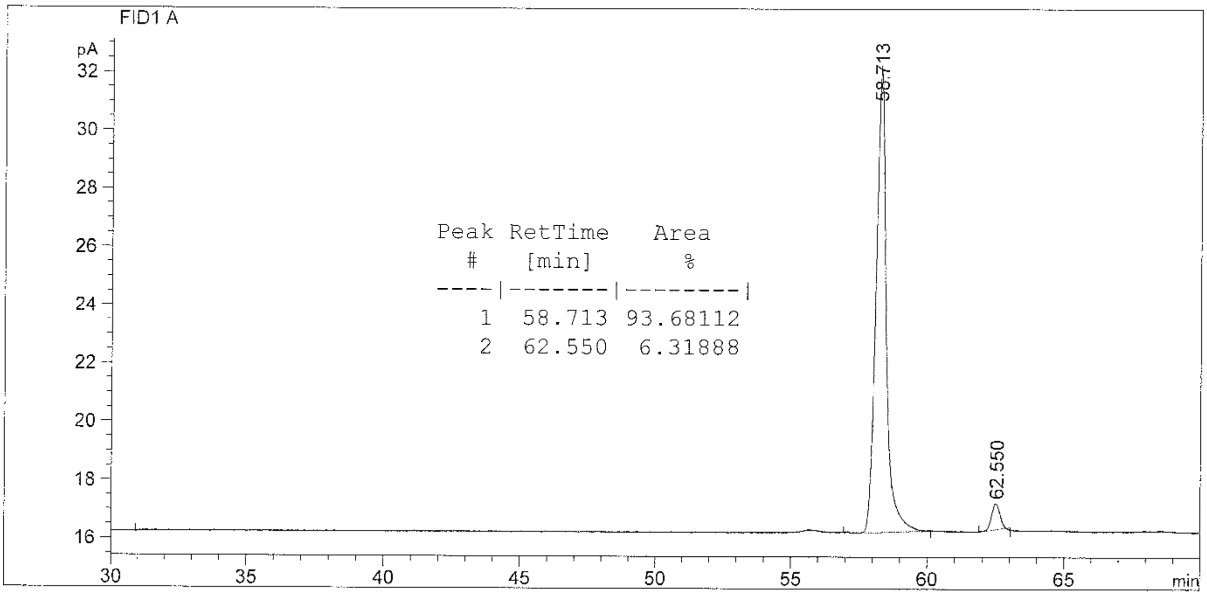

Figure S144. GC traces of: (a) racemic and (b) enantioenriched product (87% (*S*,*R*) ee using **4c** catalytic system; Scheme 5).

Ethyl 2,4,4,4-tetrafluoro-3-(p-tolyl)butanoate (from S71). Enantiomeric excess determined by HPLC using Chiracel OD-H column (hexane/2-propanol=90/10, 0.5 mL/min, 220 nm). t_R 8.3 min (*S,R*); t_R 9.1 min (*R,S*). ^{1}H NMR ($CDCl_3$), δ: 1.09 (t, *J*= 7.2 Hz, 3H), 2.28 (s, 3H), 3.94 - 4.00 (m, 1H), 4.08 (qd, 2H, *J*= 7.1 Hz, *J*= 2.5 Hz), 5.14 (dd, 1H, *J*= 47.8 Hz, *J*= 5.7 Hz), 7.10 – 7.21 (m, 4H). ^{19}F NMR ($CDCl_3$), δ: -194.1 - -194.0 (m), -65.3 (t, *J*= 9.7 Hz). ^{13}C NMR ($CDCl_3$), δ: 13.8, 21.1, 51.4 (dd, *J*= 19.1 Hz, *J*= 27.6 Hz), 62.2, 88.1 (d, *J*= 195.7 Hz), 123.7 – 139.2 (aromatic carbons), 166.9 (d, *J*= 23.3 Hz). HRMS-EI calculated for $C_{13}H_{14}F_4O_2$ $[M+Na]^+$: 301.0828, found:.301.0823.

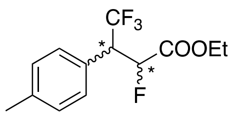

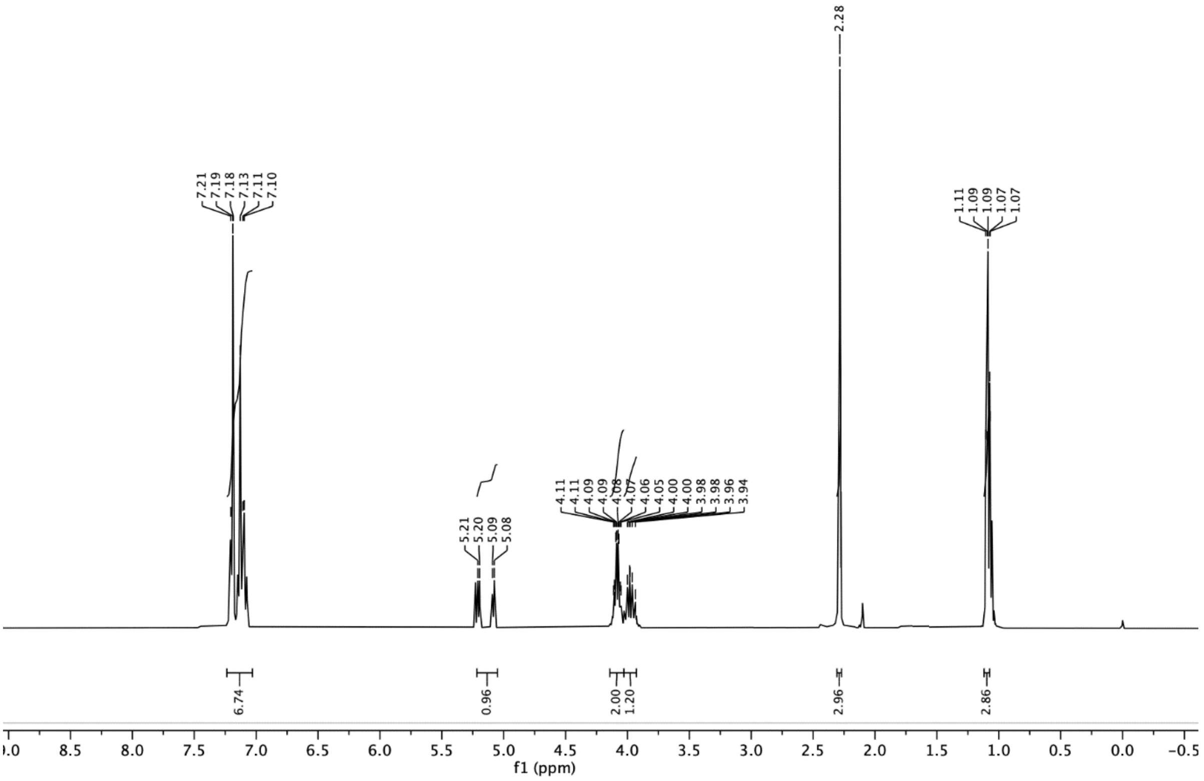

Figure S145. ^{1}H NMR spectrum of ethyl 2,4,4,4-tetrafluoro-3-(p-tolyl)butanoate (from **S71**) in $CDCl_3$.

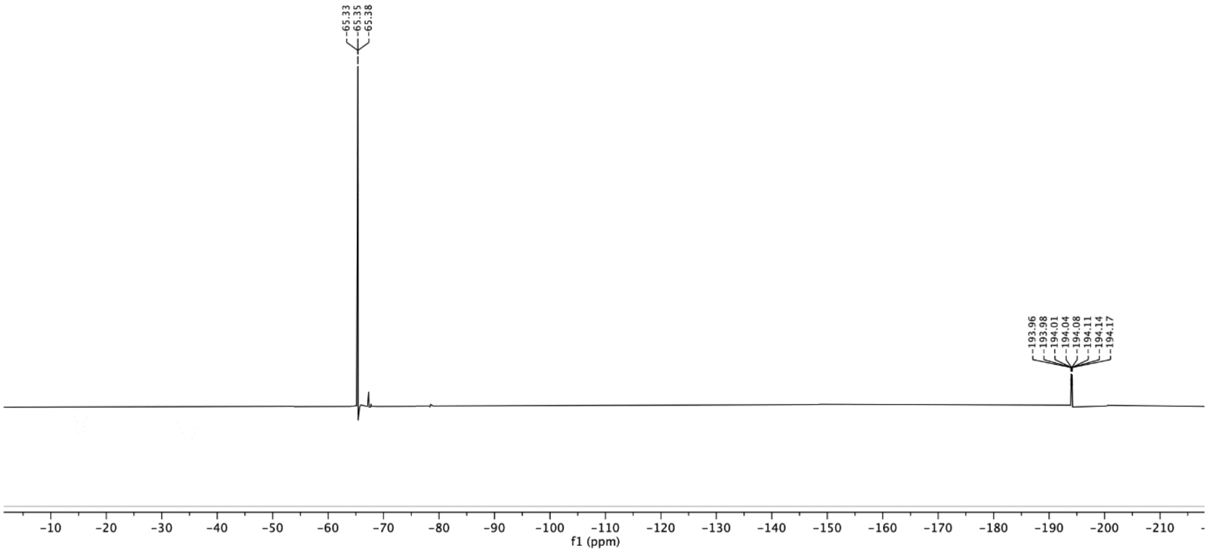

Figure S146. ^{19}F NMR spectrum of ethyl 2,4,4,4-tetrafluoro-3-(p-tolyl)butanoate (from **S71**) in $CDCl_3$.

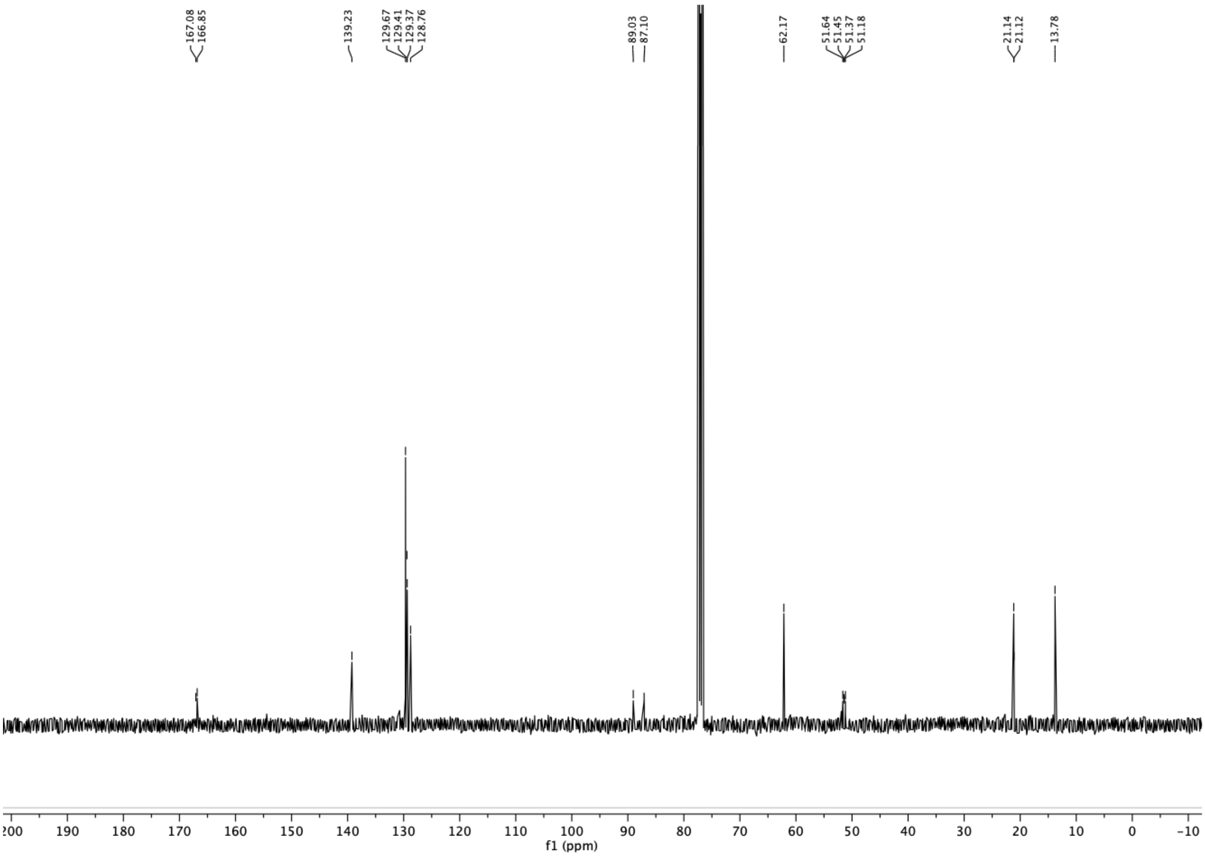

Figure S147. ^{13}C {^{1}H} NMR spectrum of ethyl 2,4,4,4-tetrafluoro-3-(p-tolyl)butanoate (from **S71**) in $CDCl_3$.

(a)

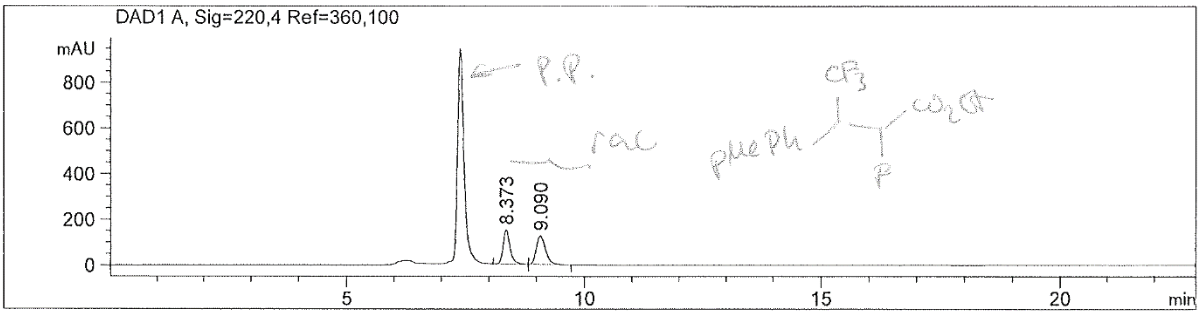

(b)

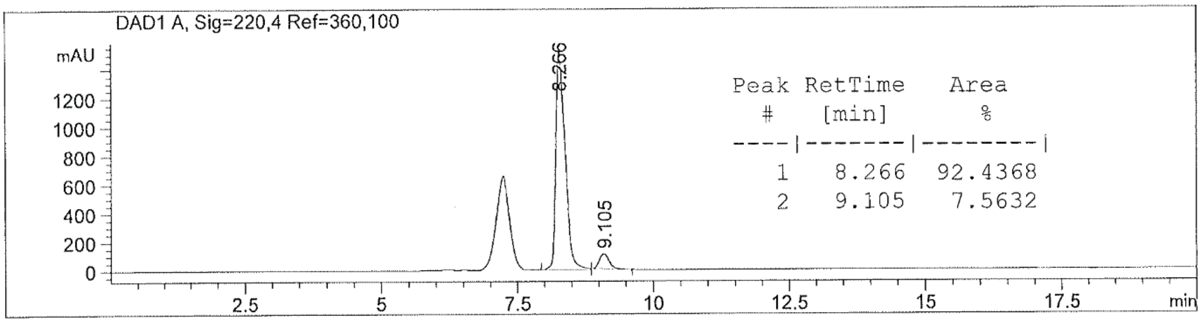

Figure S148. HPLC traces of (a) racemic and (b) enantioenriched product (85% (*S*,*R*) ee using **4c** catalytic system; Scheme 5). The peak at ca 7.5 min correspond to the unreacted starting material (**S71**).

Ethyl 2,4,4,4-tetrafluoro-3-(4-methoxyphenyl)butanoate (from S72). Enantiomeric excess determined

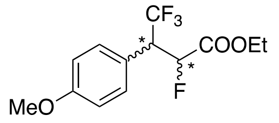

by HPLC using Chiracel OD-H column (hexane/2-propanol=90/10, 0.5 mL/min, 220 nm). t_R 10.1 min (*S*,*R*); t_R 10.7 min (*R*,*S*). ^1H NMR ($CDCl_3$), δ: 1.10 (t, *J*= 7.1 Hz, 3H), 3.74 (s, 3H), 3.83 – 3.92 (m, 1H), 4.09 (tq, 2H, *J*= 7.1 Hz, *J*= 3.3 Hz), 5.13 (dd, 1H, *J*= 47.8 Hz, *J*= 5.7 Hz), 6.83 (d, 2H, *J*= 8.9 Hz), 7.23 (d, 2H, *J*= 8.7 Hz). ^{19}F NMR ($CDCl_3$), δ: -194.1 - -193.8 (m), -65.6 (m). ^{13}C NMR ($CDCl_3$), δ: 13.8, 50.9 (m), 62.2, 88.1 (d, *J*= 196.2 Hz), 114.2 – 130.7 (aromatic carbons), 167.0 (d, *J*= 23.4 Hz). HRMS-EI calculated for $C_{13}H_{14}F_4O_3$ [M+Na]+ : 317.0777, found:.317.0772.

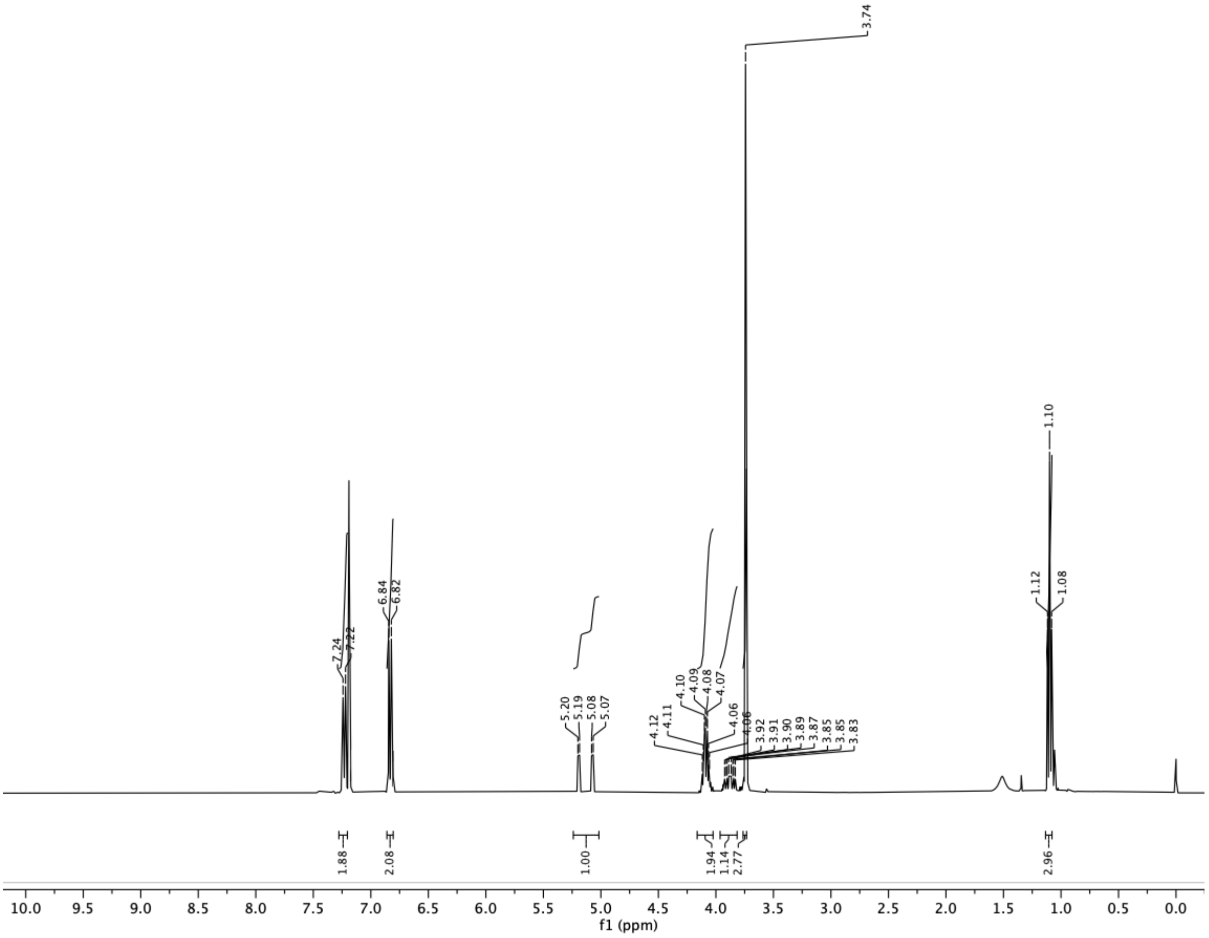

Figure S149. ^1H NMR spectrum of ethyl 2,4,4,4-tetrafluoro-3-(4-methoxyphenyl)butanoate (from **S72**) in $CDCl_3$.

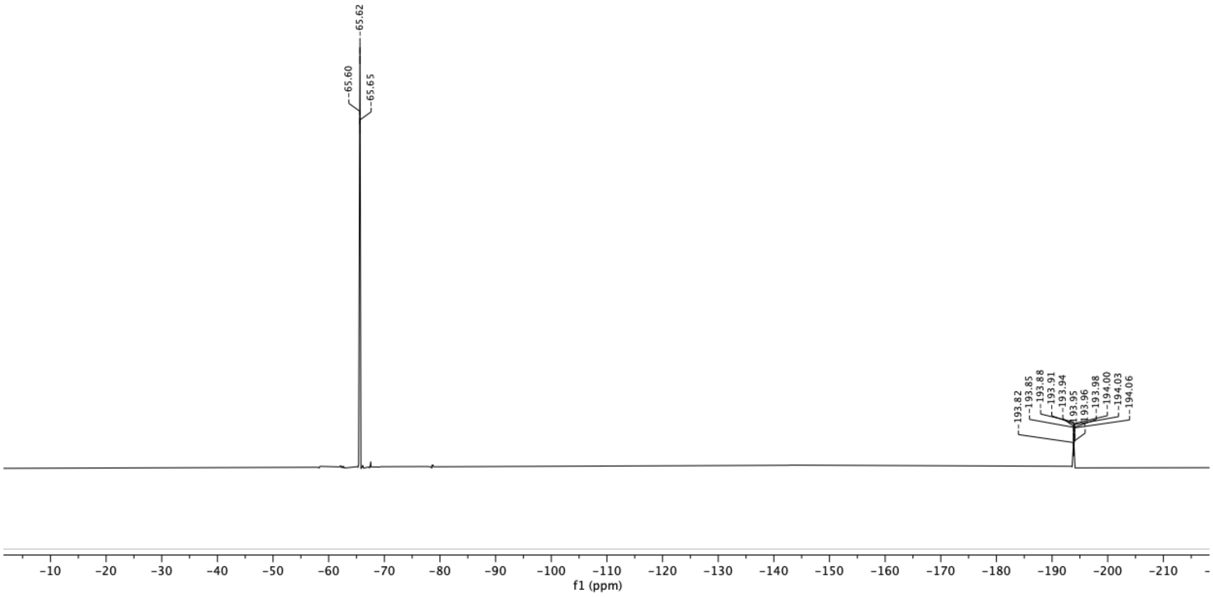

Figure S150. ^{19}F NMR spectrum of ethyl 2,4,4,4-tetrafluoro-3-(4-methoxyphenyl)butanoate (from **S72**) in $CDCl_3$.

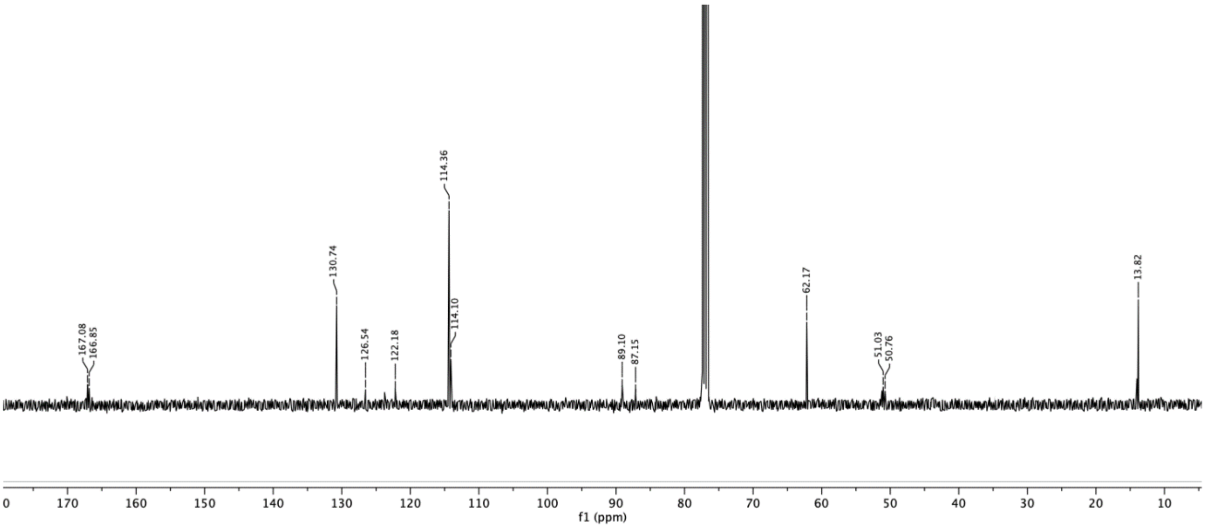

Figure S151. $^{13}C\{^1H\}$ NMR spectrum of ethyl 2,4,4,4-tetrafluoro-3-(4-methoxyphenyl)butanoate (from **S72**) in $CDCl_3$.

(a)

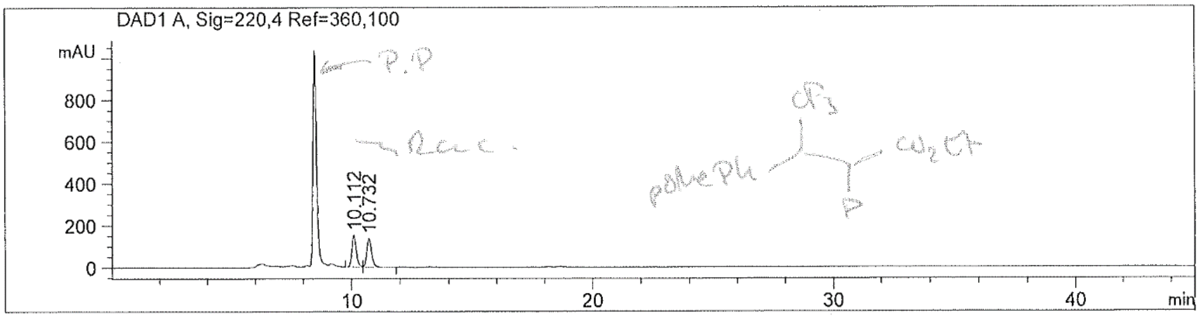

(b)

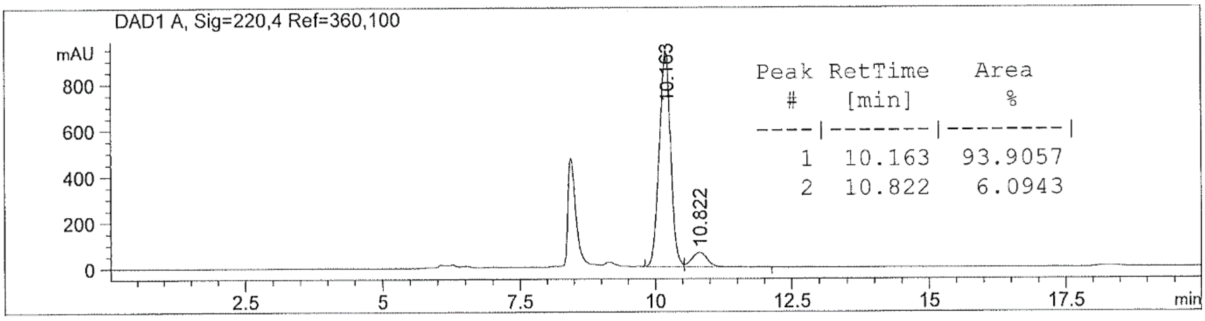

Figure S152. HPLC traces of (a) racemic and (b) enantioenriched product (88% (*S*,*R*) ee using **4c** catalytic system; Scheme 5). The peak at ca 8.5 min correspond to the unreacted starting material (**S72**).

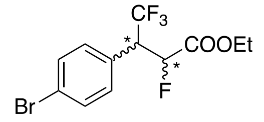

Ethyl 3-(4-bromophenyl)-2,4,4,4-tetrafluorobutanoate (from S73). Enantiomeric excess determined by HPLC using Chiracel OD-H column (hexane/2-propanol=90/10, 0.5 mL/min, 220 nm). t_R 9.2 min (*S*,*R*); t_R 9.9 min (*R*,*S*). ^{1}H NMR ($CDCl_3$), δ: 1.08 (t, *J*= 7.1 Hz, 3H), 3.88 - 4.05 (m, 1H), 4.06 – 4.11 (m, 2H), 5.17 (dd, 1H, *J*= 47.7 Hz, *J*= 5.6 Hz), 7.26 – 7.32 (m, 4H). ^{19}F NMR ($CDCl_3$), δ: -194.3 - -194.0 (m), -65.2 (m). ^{13}C NMR ($CDCl_3$), δ: 13.8, 51.8 (dd, *J*= 19.1 Hz, *J*= 27.6 Hz), 62.2, 88.0 (d, *J*= 196.0 Hz), 123.7 – 130.3 (aromatic carbons), 166.9 (d, *J*= 23.3 Hz). HRMS-EI calculated for $C_{12}H_{11}BrF_4O_2$ [M+Na]+ : 364.9776, found:.364.9772.

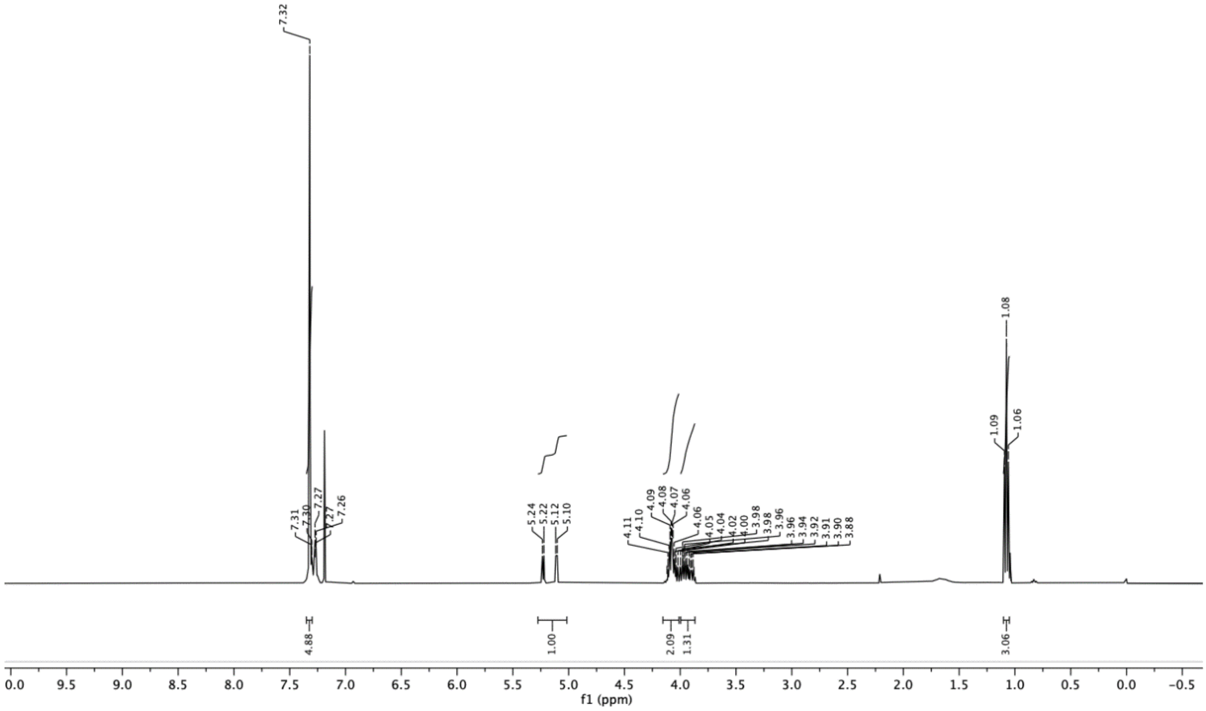

Figure S153. ^{1}H NMR spectrum of ethyl 3-(4-bromophenyl)-2,4,4,4-tetrafluorobutanoate (from **S73**) in $CDCl_3$.

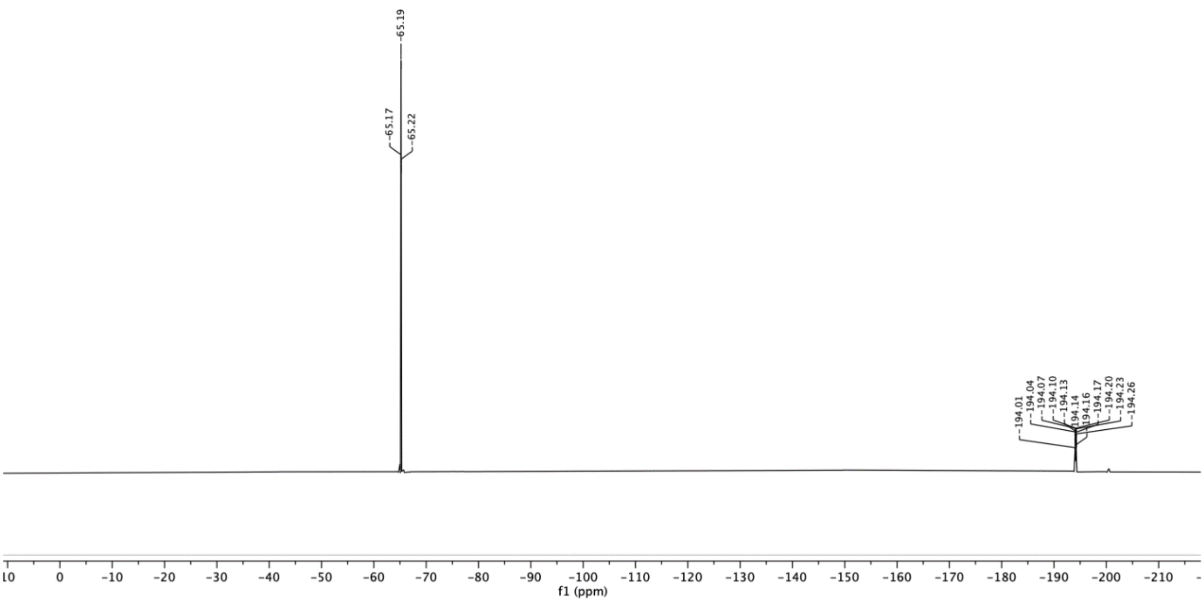

Figure S154. ^{19}F NMR spectrum of ethyl 3-(4-bromophenyl)-2,4,4,4-tetrafluorobutanoate (from **S73**) in $CDCl_3$.

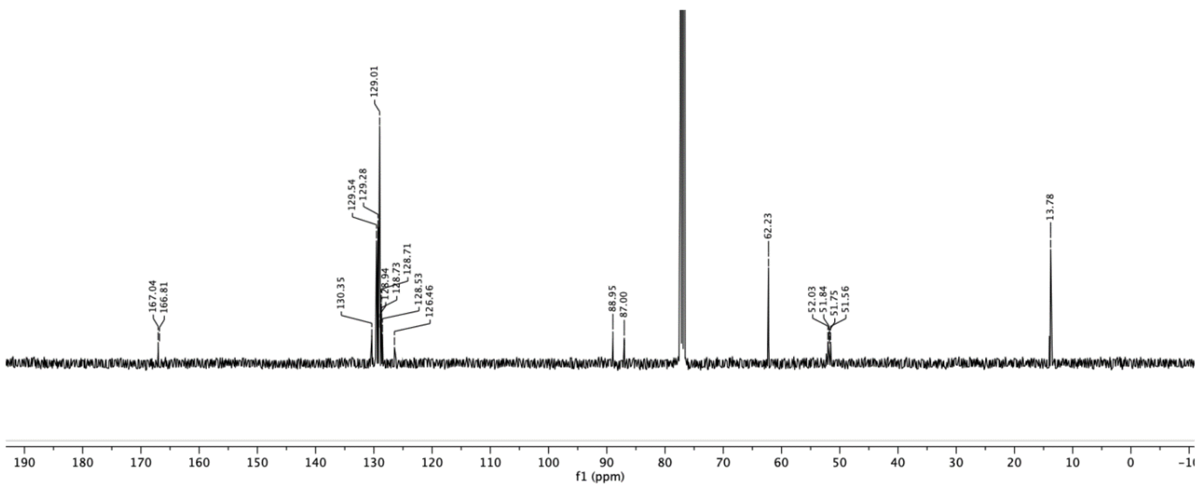

Figure S155. $^{13}C\{^{1}H\}$ NMR spectrum of ethyl 3-(4-bromophenyl)-2,4,4,4-tetrafluorobutanoate (from **S73**) in $CDCl_3$.

(a)

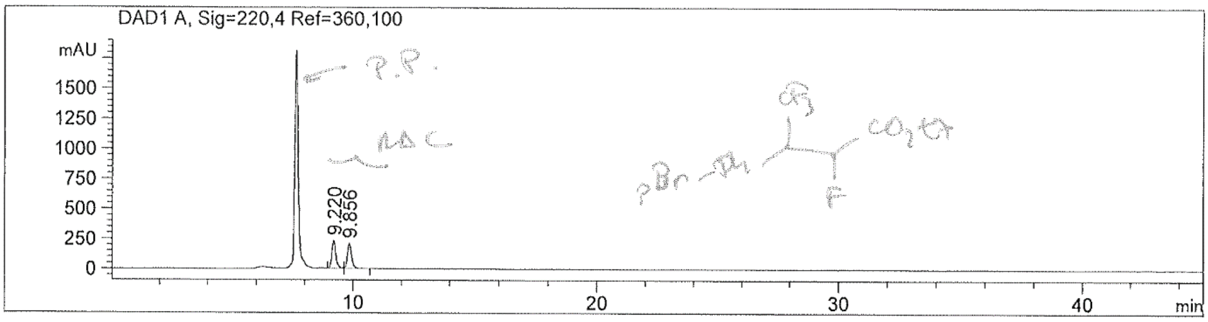

(b)

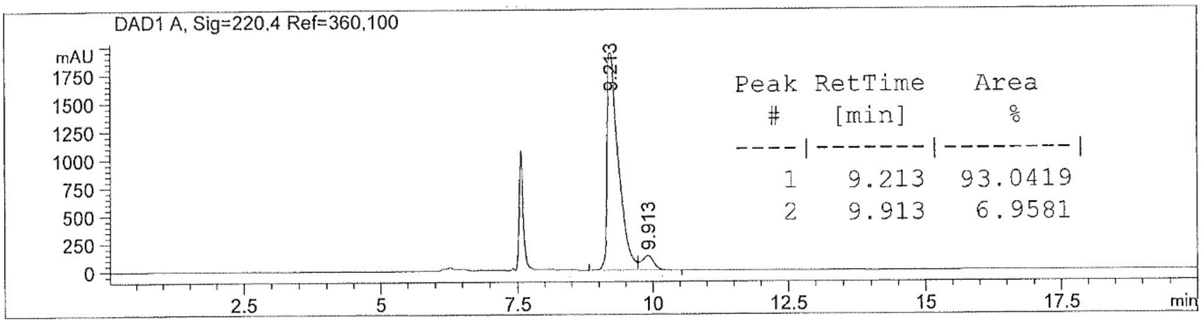

Figure S156. HPLC traces of (a) racemic and (b) enantioenriched product (86% (*S*,*R*) ee using **4c** catalytic system; Scheme 5). The peak at ca 7.5 min correspond to the unreacted starting material (**S73**).

Diethyl 2-methyl-3-phenylsuccinate (from S74).[36] Enantiomeric excess determined by HPLC using

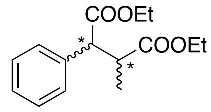

Chiracel OJ-H column (hexane/2-propanol=87/13, 0.7 mL/min, 210 nm). t_R 8.1 min (*R,R*); t_R 8.6 min (*S,S*). ^{1}H NMR ($CDCl_3$), δ: 0.94 (t, 3H, *J*= 7.1 Hz), 1.21 (t, 3H, *J*= 7.1 Hz), 1.29 (d, 3H, *J*= 6.9 Hz), 3.21-3.25 (m, 1H), 3.77 (d, 1H, *J*= 11.0 Hz), 3.86 (q, 2H, *J*= 7.1 Hz), 4.07-4.20 (m, 2H), 7.23-7.35 (m, 5H).

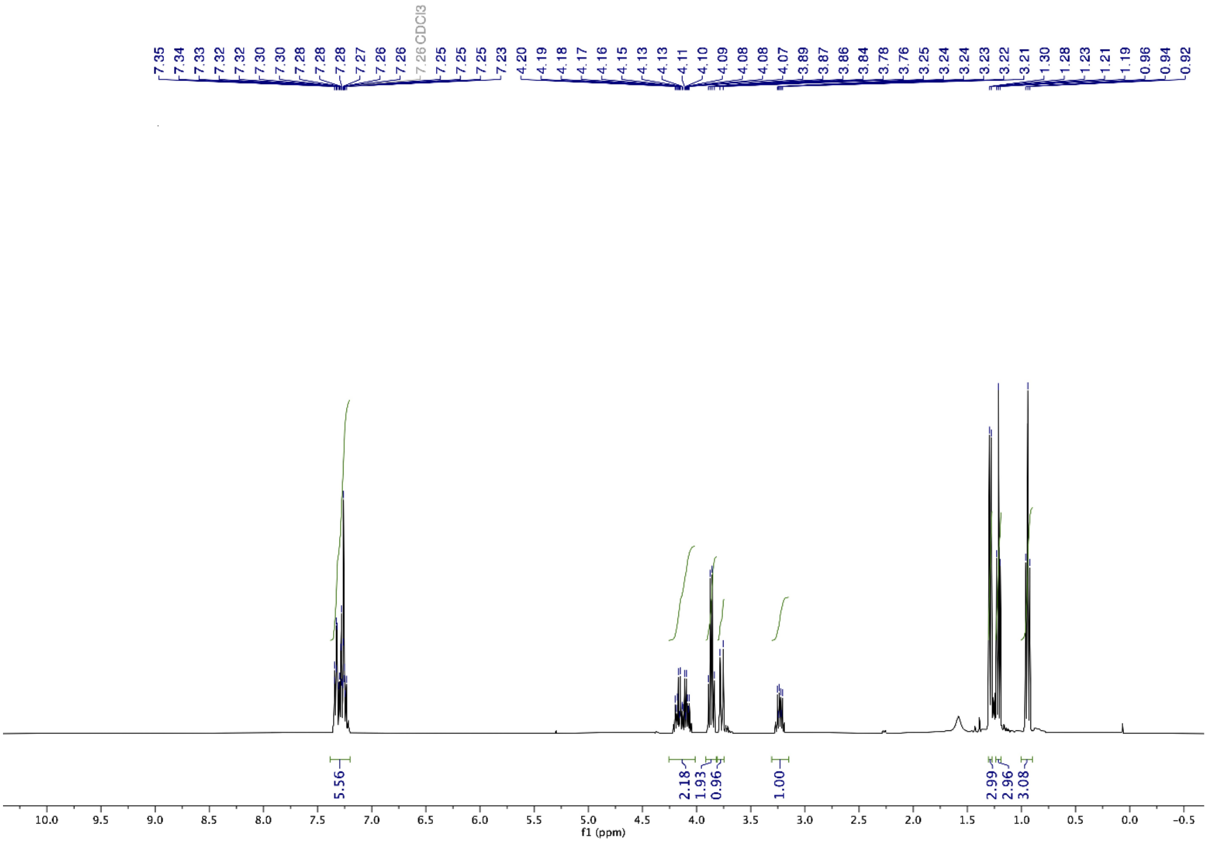

Figure S157. ^{1}H NMR spectrum of diethyl 2-methyl-3-phenylsuccinate (from **S74**) in $CDCl_3$.

(a)

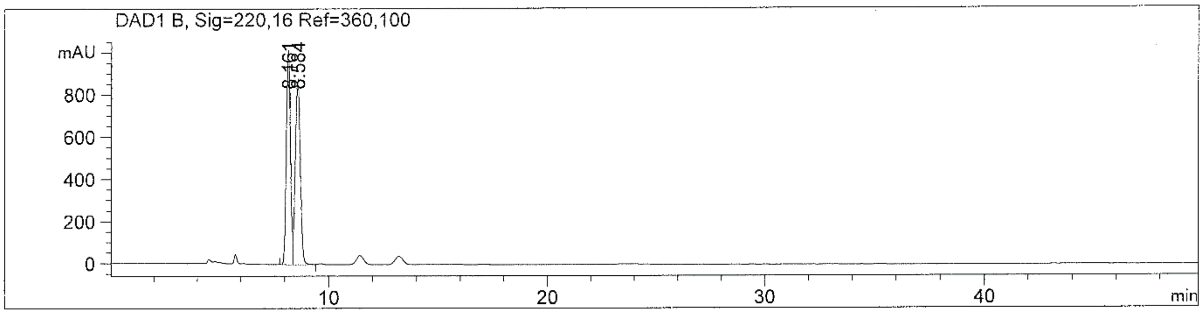

(b)

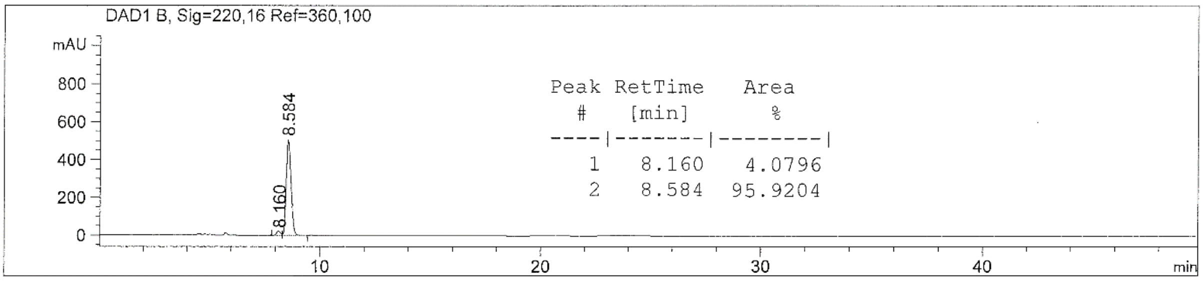

Figure S158. HPLC trace of: (a) racemic and (b) enantioenriched product (92% (*S*,*S*) ee using **4c** catalytic system; Scheme 5).

1-Benzyl 4-ethyl 3-methyl-2-phenylsuccinate (from S75).[36] Enantiomeric excess determined by HPLC

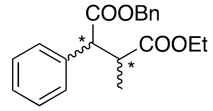

using Chiracel OJ-H column (hexane/2-propanol=95/5, 1 mL/min, 210 nm). t_R 11.8 min (*R,R*); t_R 13.0 min (*S,S*). ^{1}H NMR ($CDCl_3$), δ: 0.94 (t, 3H, *J*= 7.1 Hz), 1.27 (d, 3H, *J*= 6.8 Hz), 3.21-3.29 (m, 1H), 3.84-3.89 (m, 3H), 5.07 (d, 1H, *J*= 12.5 Hz), 5.16 (d, 1H, *J*= 12.4 Hz), 7.23-7.34 (m, 10H).

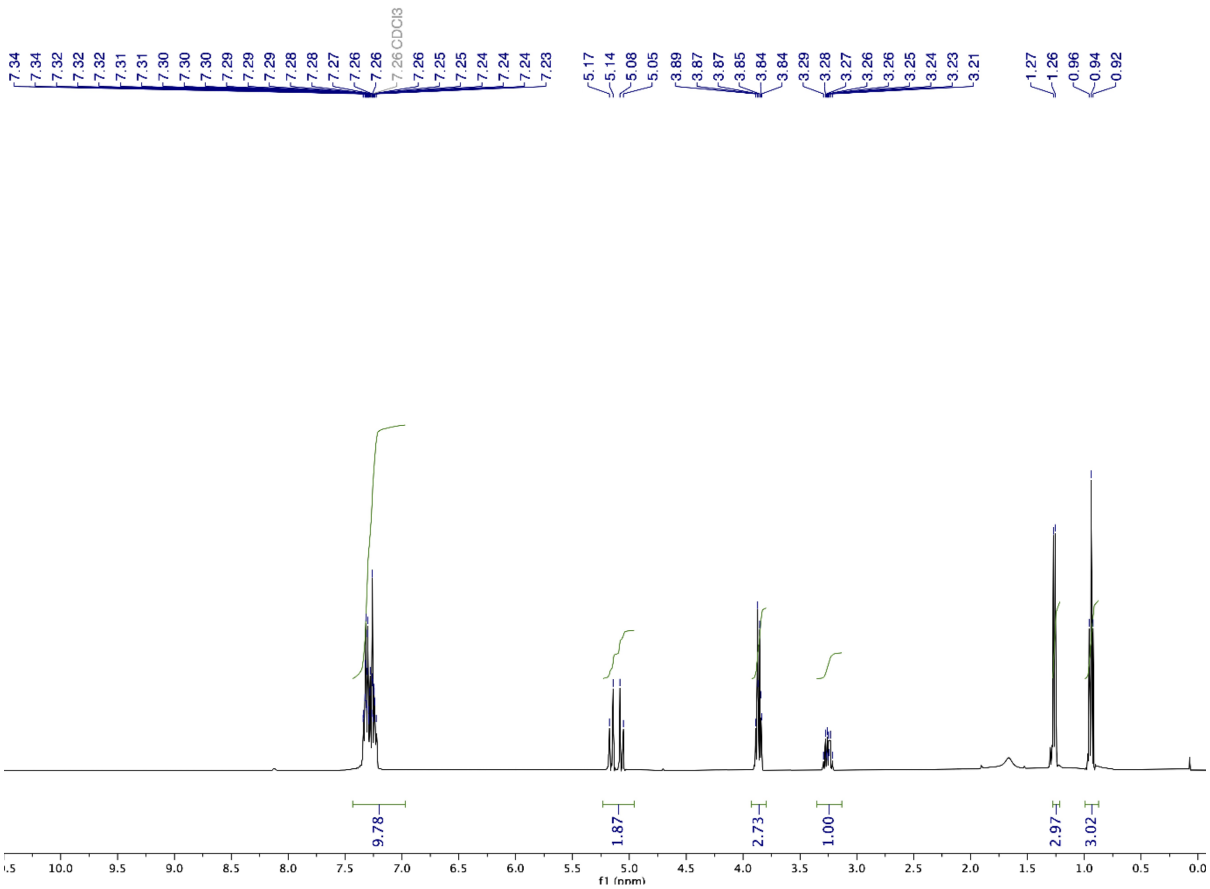

Figure S159. ^{1}H NMR spectrum of 1-benzyl 4-ethyl 3-methyl-2-phenylsuccinate (from **S75**) in $CDCl_3$.

(a)

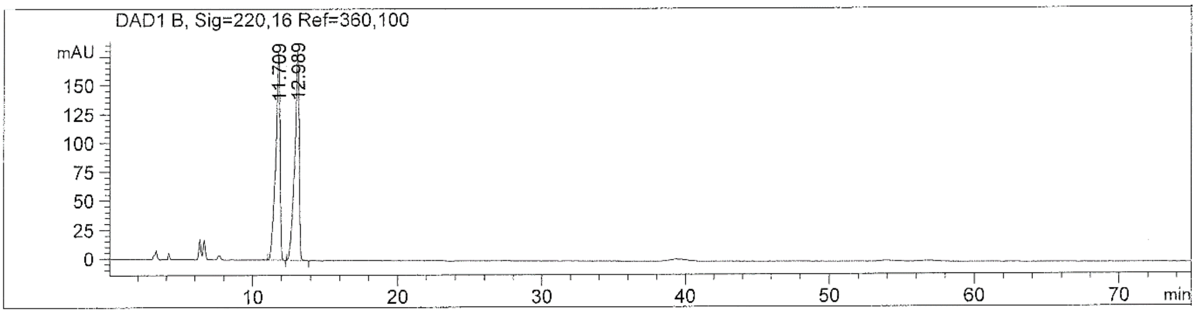

(b)

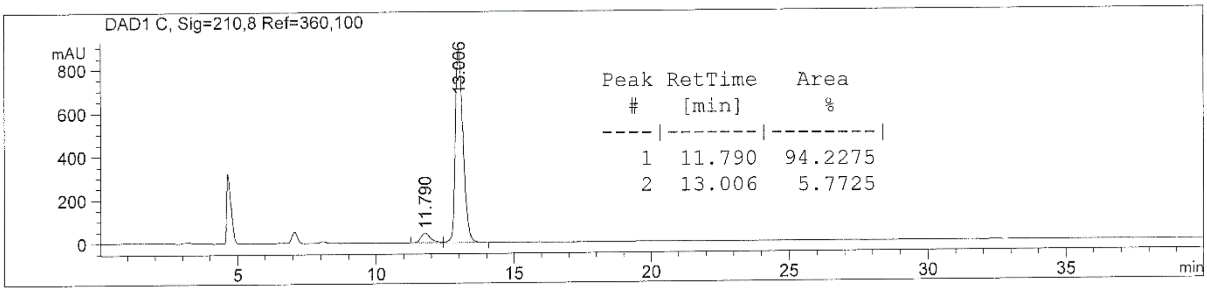

Figure S160. HPLC trace of: (a) and (b) enantioenriched product (88% (*S*,*S*) ee using **4c** catalytic system; Scheme 5).

1-Benzyl 4-ethyl 3-methyl-2-(p-tolyl)succinate (from S76).[36] Enantiomeric excess determined by HPLC using Chiracel OJ-H column (hexane/2-propanol=95/5, 1 mL/min, 210 nm). t_R 8.5 min (*R,R*); t_R 9.7 min (*S,S*). ^{1}H NMR ($CDCl_3$), δ: 0.97 (t, 3H, *J*= 7.1 Hz), 1.25 (d, 3H, *J*= 6.8 Hz), 2.31 (s, 3H), 3.23 (dq, 1H, *J*= 6.8 Hz, *J*= 10.9 Hz), 3.82 (d, 1H, *J*= 10.9 Hz), 3.88 (q, 2H, *J*= 7.1 Hz), 5.05 (d, 1H, *J*= 12.4 Hz), 5.16 (d, 1H, *J*= 12.4 Hz), 7.08-7.10 (m, 2H), 7.19-7.32 (m, 8H).

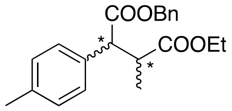

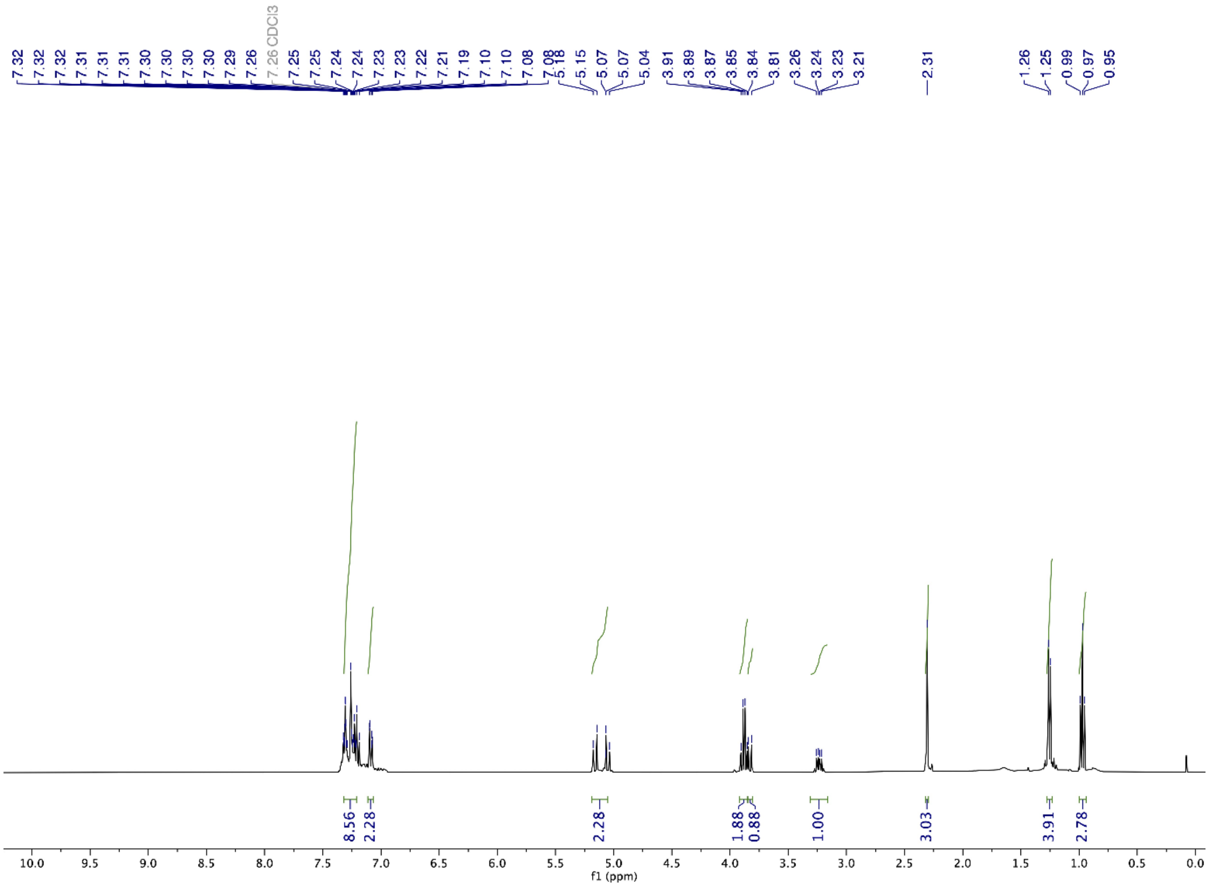

Figure S161. ^{1}H NMR spectrum of 1-benzyl 4-ethyl 3-methyl-2-(p-tolyl)succinate (from **S76**) in $CDCl_3$.

(a)

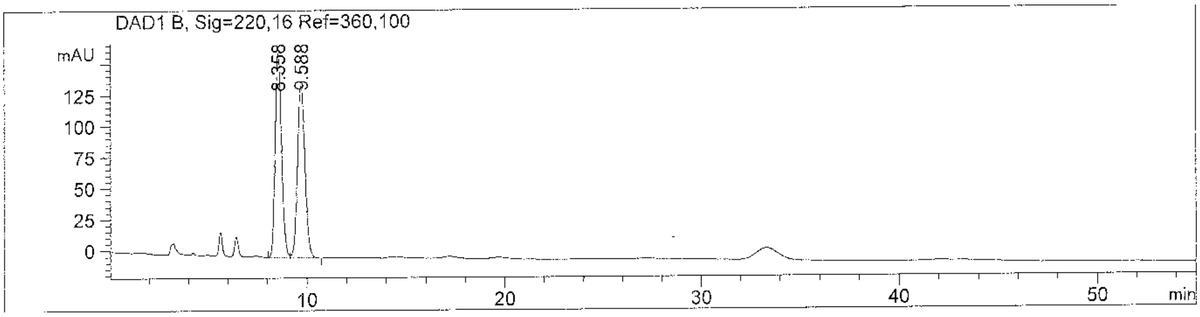

(b)

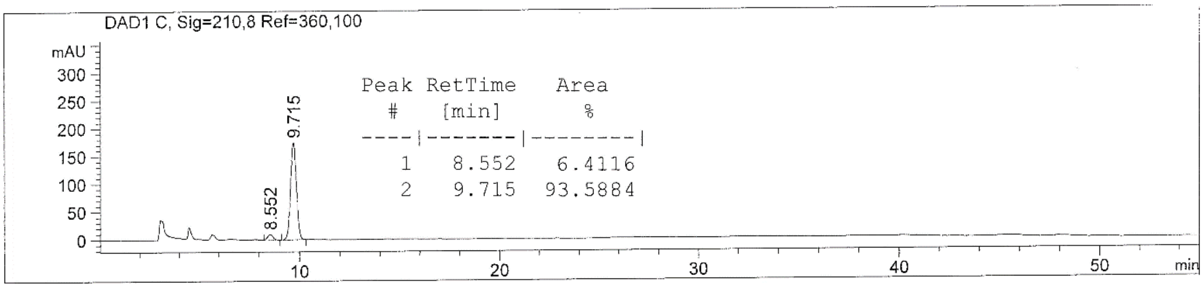

Peak #	RetTime [min]	Area %
1	8.552	6.4116
2	9.715	93.5884

Figure S162. HPLC trace of: (a) racemic and (b) enantioenriched product (87% (*S*,*S*) ee using **4c** catalytic system; Scheme 5).

1-Benzyl 4-ethyl 2-(4-methoxyphenyl)-3-methylsuccinate (from S77).[36] Enantiomeric excess

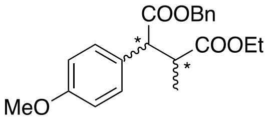

determined by HPLC using Chiracel OJ-H column (hexane/2-propanol=95/5, 1 mL/min, 210 nm). t_R 14.6 min (*R,R*); t_R 18.8 min (*S,S*). ^{1}H NMR ($CDCl_3$), δ: 0.97 (t, 3H, *J*= 7.1 Hz), 1.25 (d, 3H, *J*= 6.8 Hz), 3.22 (dq, 1H, *J*= 6.8 Hz, *J*= 11.0 Hz), 3.78 (s, 3H), 3.80 (d, 1H, *J*= 11.1 Hz), 3.88 (q, 2H, *J*= 7.1 Hz), 5.06 (d, 1H, *J*= 12.4 Hz), 5.15 (d, 1H, *J*= 12.4 Hz), 6.80-6.82 (m, 2H), 7.23-7.32 (m, 7H).

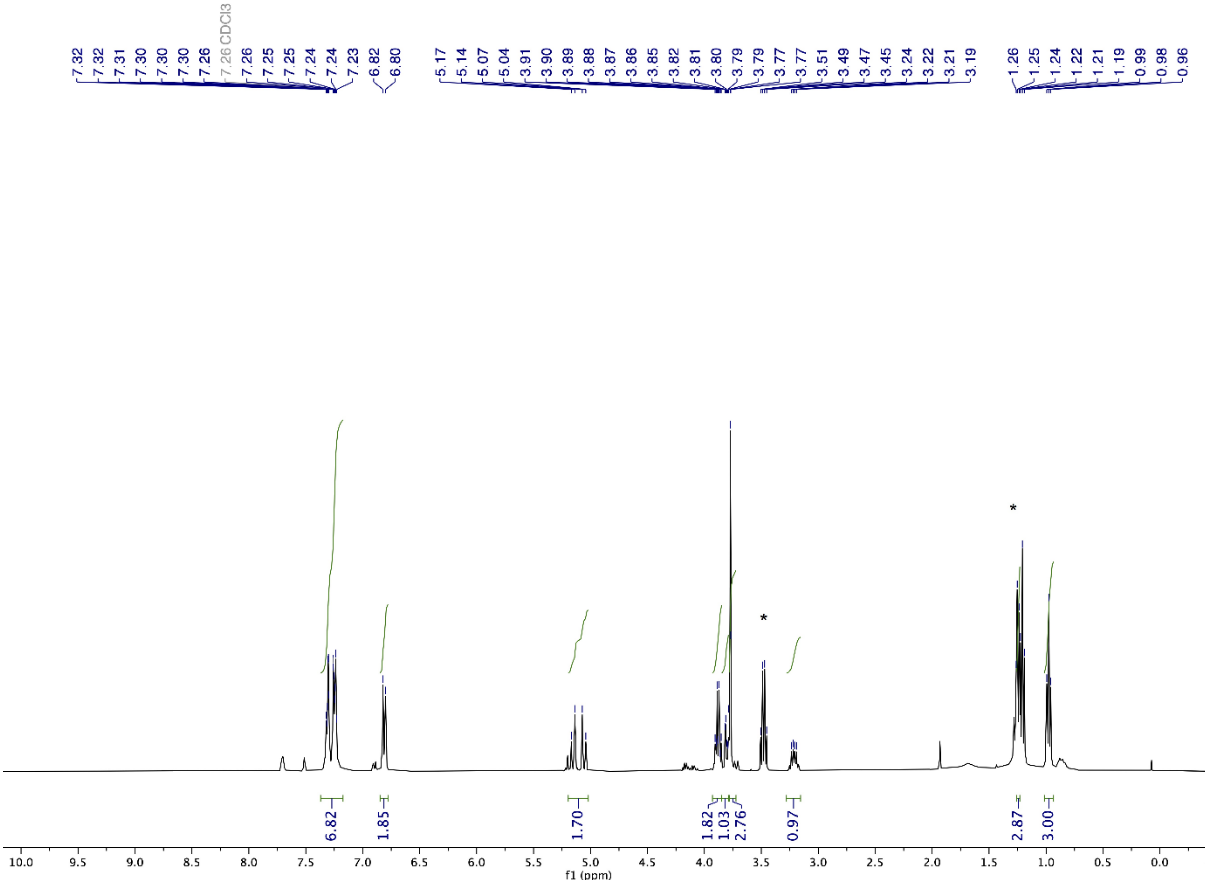

Figure S163. ^{1}H NMR spectrum of 1-benzyl 4-ethyl 2-(4-methoxyphenyl)-3-methylsuccinate (from **S77**) in $CDCl_3$. The signals marked with an * correspond to Et_2O.

(a)

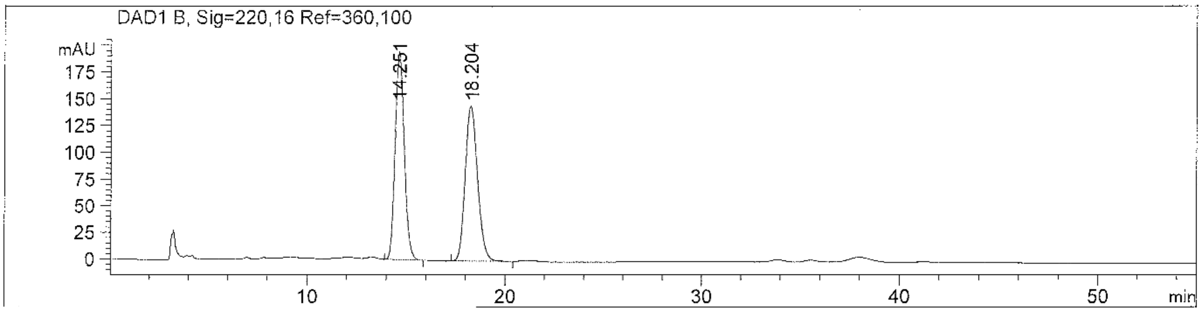

(b)

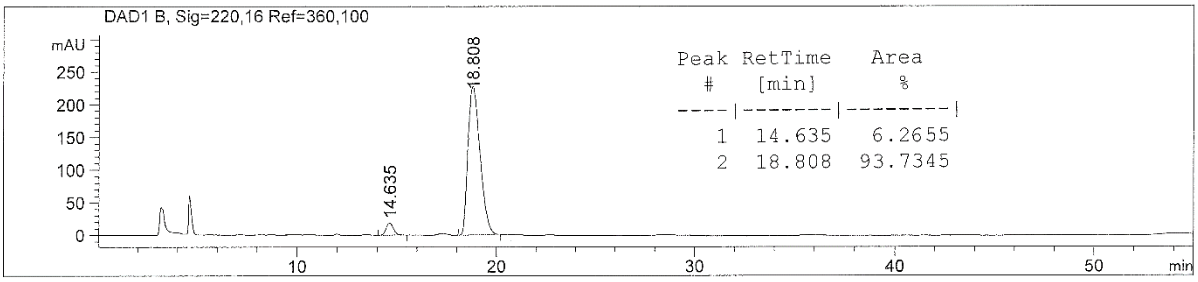

Figure S164. HPLC trace of: (a) racemic and (b) enantioenriched product (88% (*S*,*S*) ee using **4c** catalytic system; Scheme 5).

1-Benzyl 4-ethyl 2-(2,5-dimethylfuran-3-yl)-3-methylsuccinate (from S78).[36] Enantiomeric excess

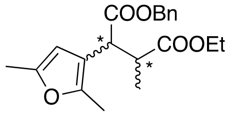

determined by HPLC using Chiracel IA column (hexane/2-propanol=95/5, 1 mL/min, 220 nm). t_R 6.4 min (*S,S*); t_R 7.1 min (*R,R*). ^{1}H NMR ($CDCl_3$), δ: 1.08 (t, 3H, *J*= 7.1 Hz), 1.19 (d, 3H, *J*= 6.9 Hz), 2.18 (s, 6H), 3.07 (dq, 1H, *J*= 6.9 Hz, *J*= 10.6 Hz), 3.67 (d, 1H, *J*= 10.6 Hz), 3.95-4.01 (m, 2H), 5.09 (d, 1H, *J*= 12.4 Hz), 5.15 (d, 1H, *J*= 12.4 Hz), 5.92 (s, 1H), 7.26-7.35 (m, 5H).

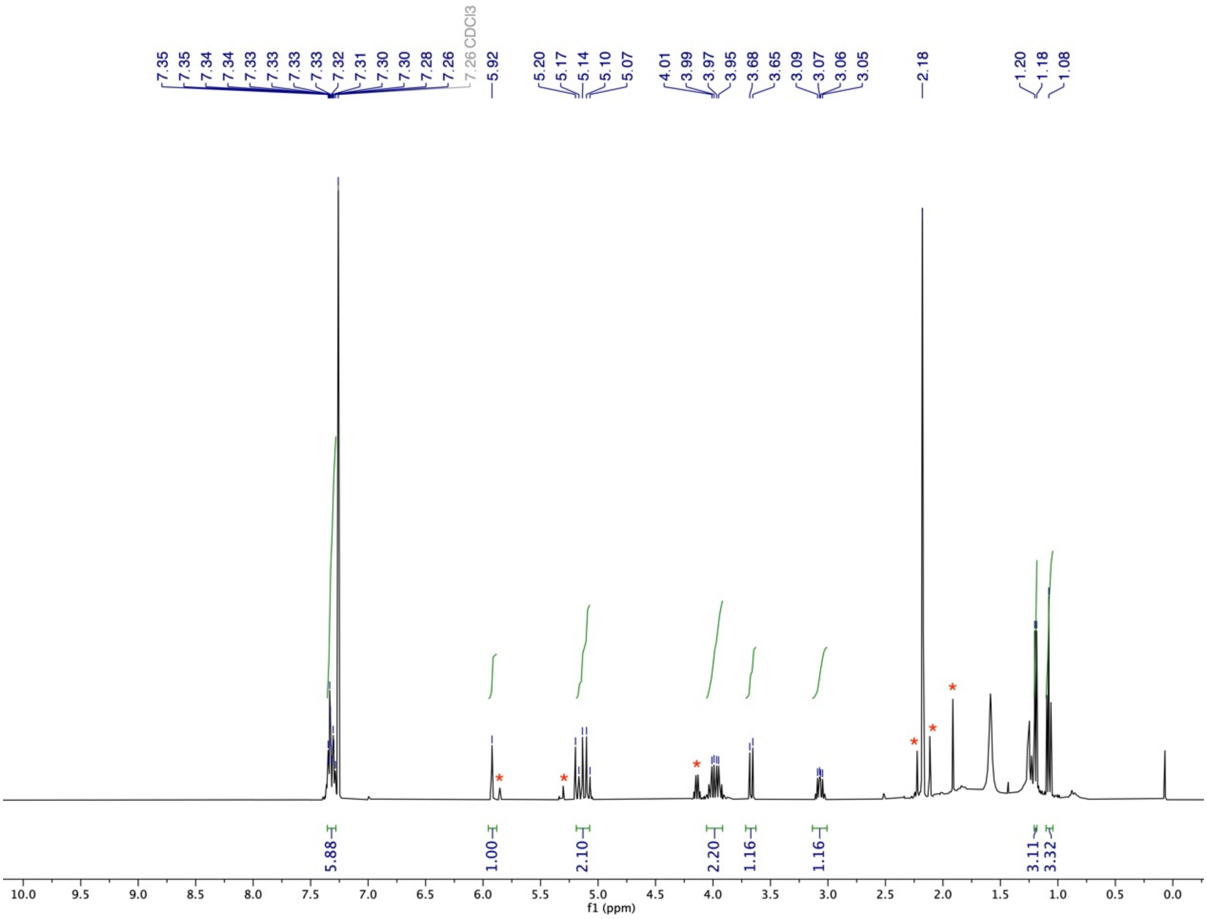

Figure S165. ^{1}H NMR spectrum of 1-benzyl 4-ethyl 2-(2,5-dimethylfuran-3-yl)-3-methylsuccinate (from **S78**) in $CDCl_3$ (peaks marked with * correspond to starting material).

(a)

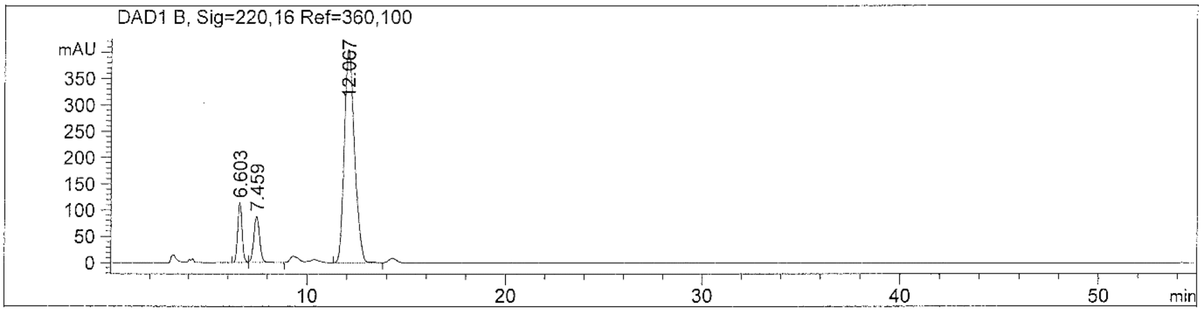

(b)

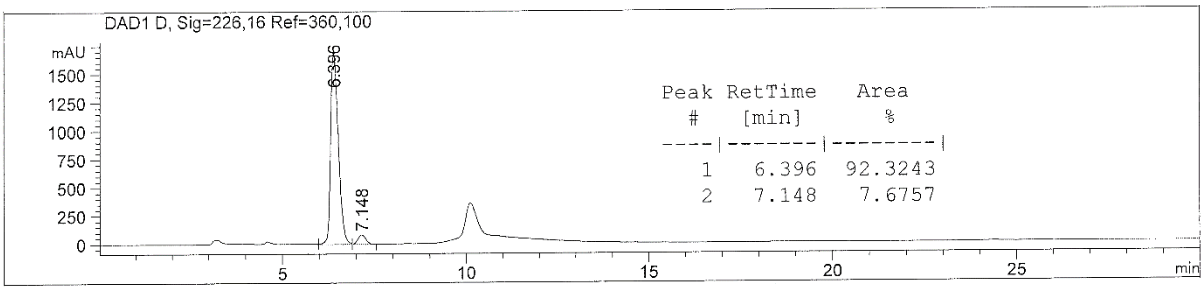

Peak #	RetTime [min]	Area %
1	6.396	92.3243
2	7.148	7.6757

Figure S166. HPLC trace of: (a) racemic and (b) enantioenriched product (85% (*S*,*S*) ee using **4c** catalytic system; Scheme 5). The peak at 12 min corresponds to the unreacted starting material (**S78**).

1-Benzyl 4-ethyl 2-(2,5-dimethylthiophen-3-yl)-3-methylsuccinate (from S79).[36] Enantiomeric excess determined by HPLC using Chiracel IA column (hexane/2-propanol=99/1, 0.5 mL/min, 210 nm). t_R 20.0 min (*S,S*); t_R 21.4 min (*R,R*). ^{1}H NMR ($CDCl_3$), δ: 1.01 (t, 3H, *J*= 7.1 Hz), 1.22 (d, 3H, *J*= 6.8 Hz), 2.31-2.34 (m, 3H), 2.33-2.36 (m, 3H), 3.17 (dq, 1H, *J*= 6.9 Hz, *J*= 10.9 Hz), 3.86-3.96 (m, 3H), 5.08 (d, 1H, *J*= 12.5 Hz), 5.15 (d, 1H, *J*= 12.5 Hz), 6.61-6.64 (m, 1H), 7.25-7. 33 (m, 5H).

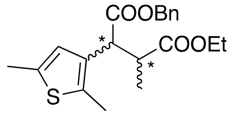

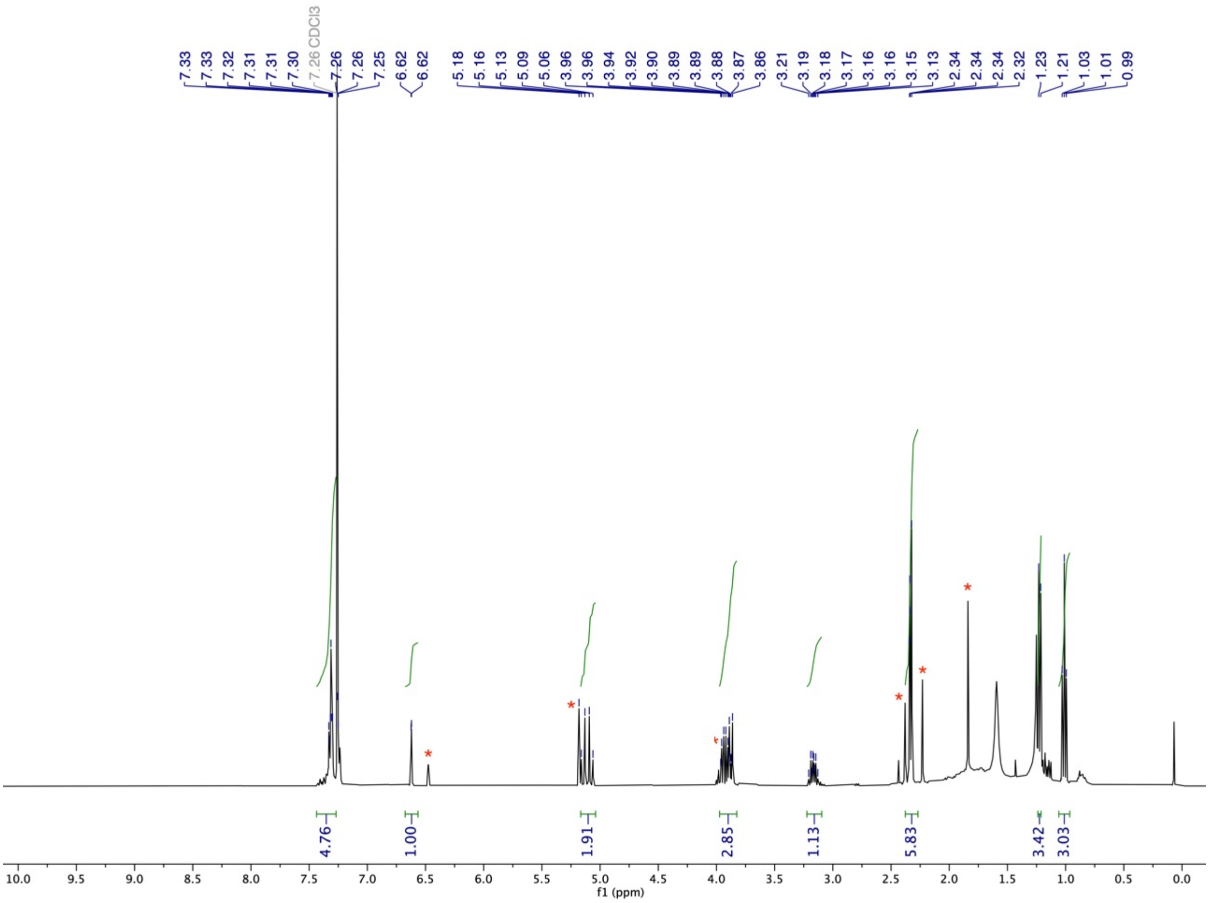

Figure S167. ^{1}H NMR spectrum of 1-benzyl 4-ethyl 2-(2,5-dimethylthiophen-3-yl)-3-methylsuccinate (from **S79**) in $CDCl_3$ (peaks marked with * correspond to starting material).

(a)

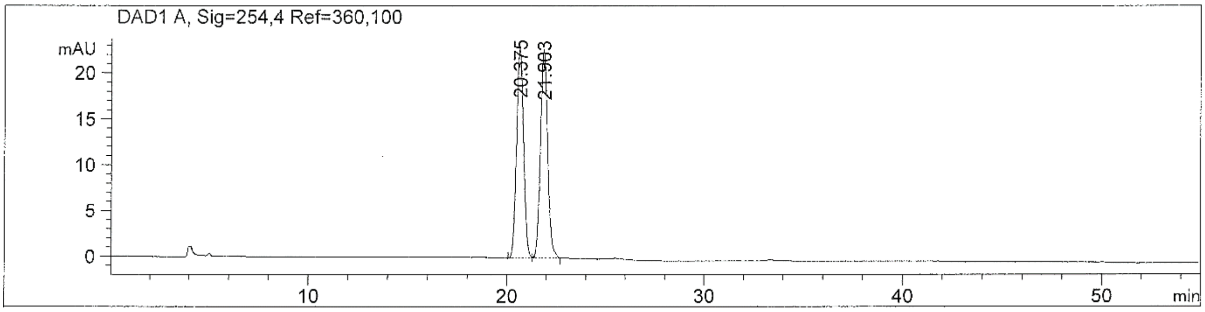

(b)

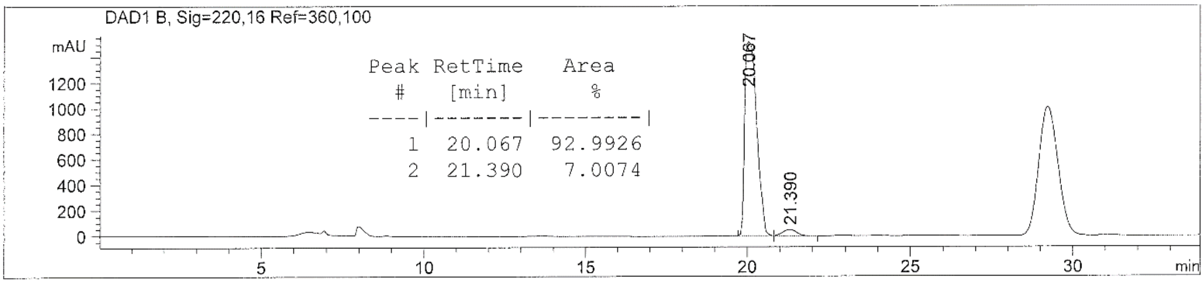

Figure S168. HPLC trace of: (a) racemic and (b) enantioenriched product (86% (*S*,*S*) ee using **4c** catalytic system; Scheme 5). The peak at ca 29 min corresponds to the unreacted starting material (**S79**).

Ethyl 2-methyl-3-phenylbutanoate (from S80).[47] Enantiomeric excess determined by HPLC using

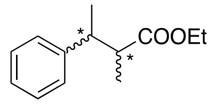

Chiracel OJ-H column (hexane/2-propanol=95/5, 0.5 mL/min, 220 nm). t_R 12.2 min (*R,R*); t_R 14.0 min (*S,S*). ^1H NMR ($CDCl_3$), δ: 0.93 (d, 3H, *J*= 6.8 Hz), 1.25 (d, 3H, *J*= 6.9 Hz), 1.29 (t, 3H, *J*= 7.1 Hz), 2.54-2.62 (m, 1H), 2.88-2.92 (m, 1H), 4.18 (q, 2H, *J*= 7.1 Hz), 7.16-7.23 (m, 3H), 7.26-7.32 (m, 2H).

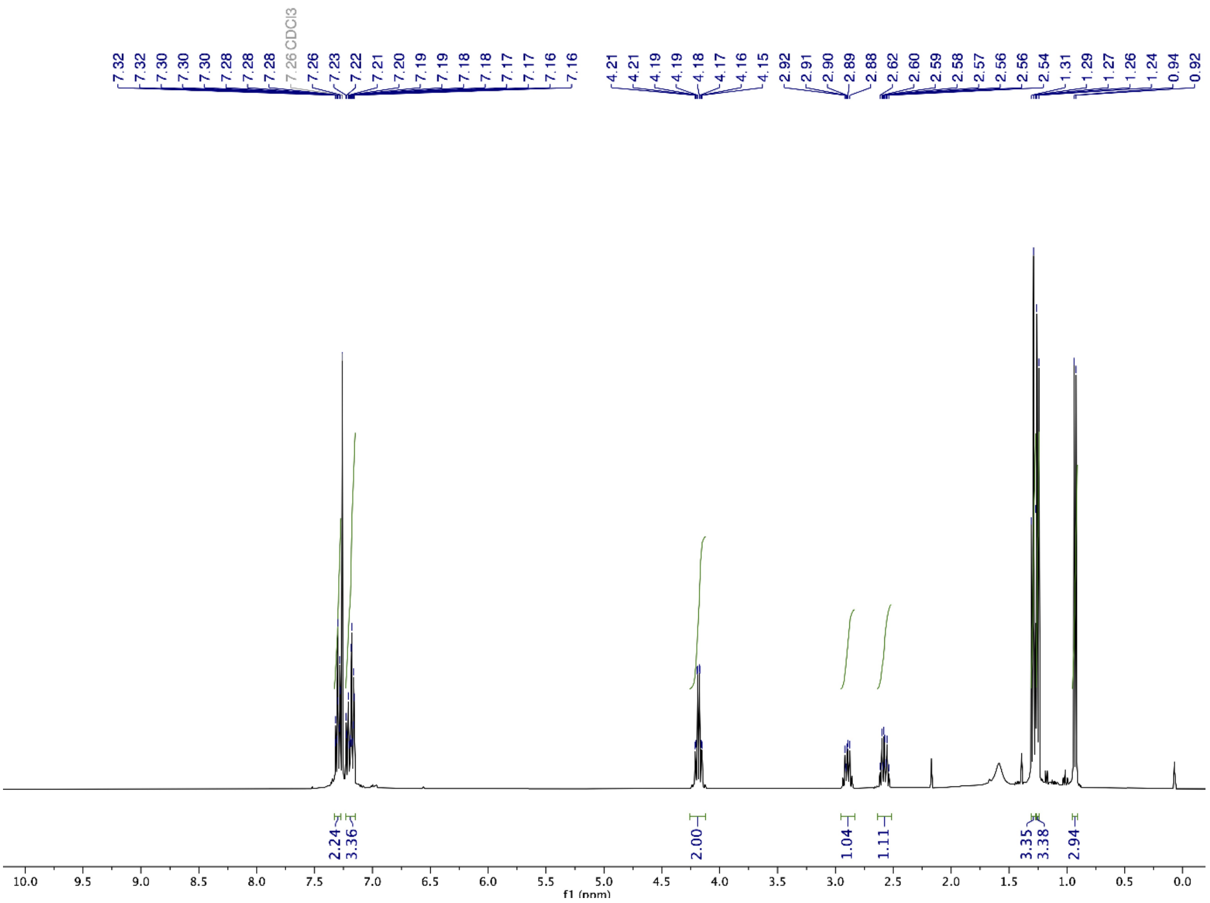

Figure S169. ^1H NMR spectrum of ethyl 2-methyl-3-phenylbutanoate (from **S80**) in $CDCl_3$.

(a)

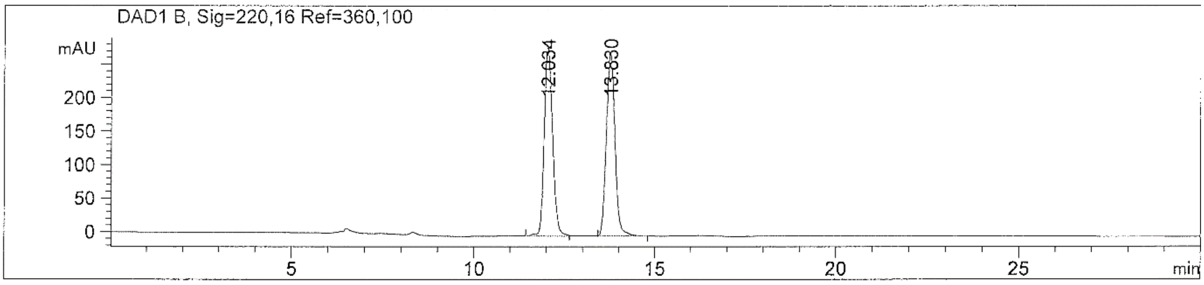

(b)

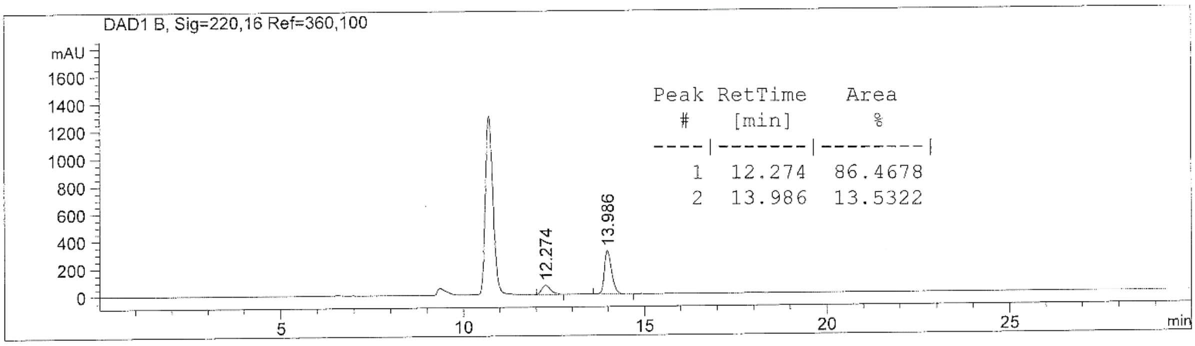

Peak #	RetTime [min]	Area %
1	12.274	86.4678
2	13.986	13.5322

Figure S170. HPLC trace of: (a) racemic and (b) enantioenriched product (73% (*S*,*S*) ee using **4c** catalytic system; Scheme 5). The peak at ca 11 min corresponds to the unreacted starting material (**S80**).

Ethyl 3-methyl-2-phenylbutanoate (from S81).[48] Enantiomeric excess determined by HPLC using

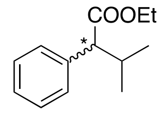

Chiracel OJ-H column (hexane/2-propanol=99/1, 1 mL/min, 210 nm). t_R 5.2 min (*R*); t_R 6.0 min (*S*). ^{1}H NMR ($CDCl_3$), δ: 0.69 (d, 3H, *J*= 6.7 Hz), 1.03 (d, 3H, *J*= 6.5 Hz), 1.20 (t, 3H, *J*= 7.1 Hz), 2.33 (dp, 1H, *J*= 10.6 Hz, *J*= 6.6 Hz), 3.12 (d, 1H, *J*= 10.6 Hz), 4.03-4.17 (m, 2H), 7.23-7.34 (m, 5H).

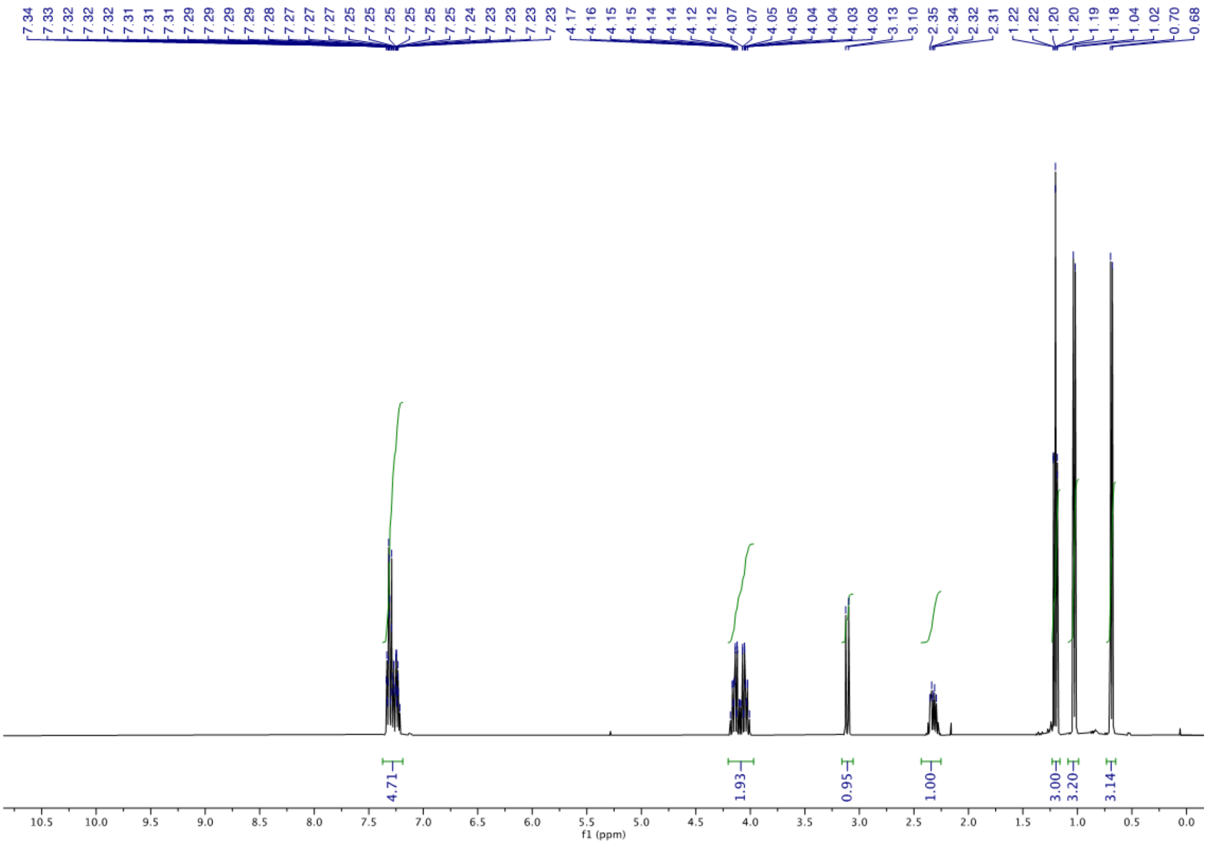

Figure S171. ^{1}H NMR spectrum of ethyl 3-methyl-2-phenylbutanoate (from **S81**) in $CDCl_3$.

(a)

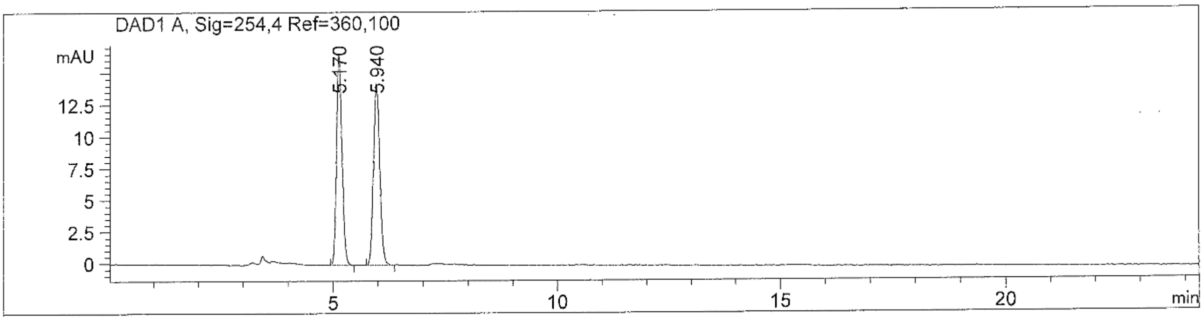

(b)

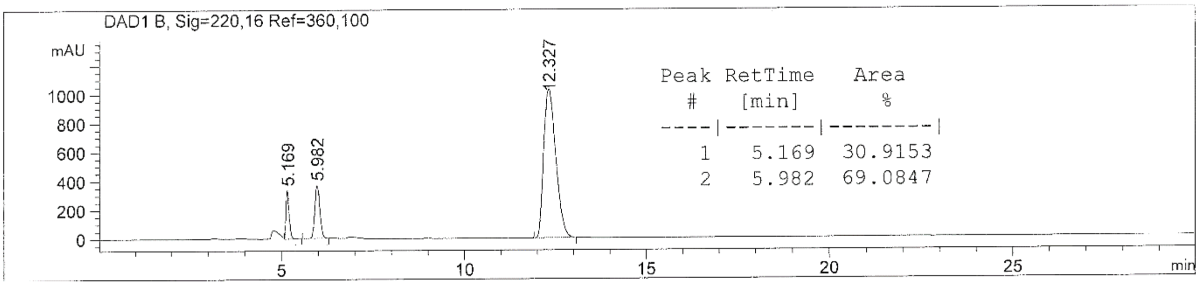

Figure S172. HPLC trace of: (a) racemic and (b) enantioenriched product (38% (*S*) ee using **4c** catalytic system; Scheme 5). The peak at 12.3 min corresponds to the unreacted starting material (**S81**).

Ethyl 3-((diphenylphosphoryl)oxy)-2-methylbutanoate (from S82). Enantiomeric excess determined

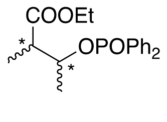

by HPLC using Chiracel OJ-H column (hexane/2-propanol=95/5, 0.5 mL/min, 210 nm). t_R 24.0 min (*minor*); t_R 28.0 min (*major*). ^{1}H NMR ($CDCl_3$), δ: 1.04 – 1.18 (m, 6H), 1.33 (d, 3H, *J*= 6.3 Hz), 2.58 (tt, 1H, *J*= 1.1 Hz, *J*= 7.1 Hz), 3.88-4.11 (m, 2H), 4.76-5.07 (m, 1H), 7.05 – 7.18 (m, 6H), 7.22 – 7.33 (m, 4H). ^{31}P NMR ($CDCl_3$), δ: -12.8 (s). ^{13}C NMR ($CDCl_3$), δ: 11.7, 15.1, 20.1, 46.6, 61.0, 78.3, 121.0, 125.4, 131.0, 151.9, 173.2. HRMS-EI calculated for $C_{19}H_{23}O_4P$ [M+Na]+ : 369.1232, found:. 369.1229.

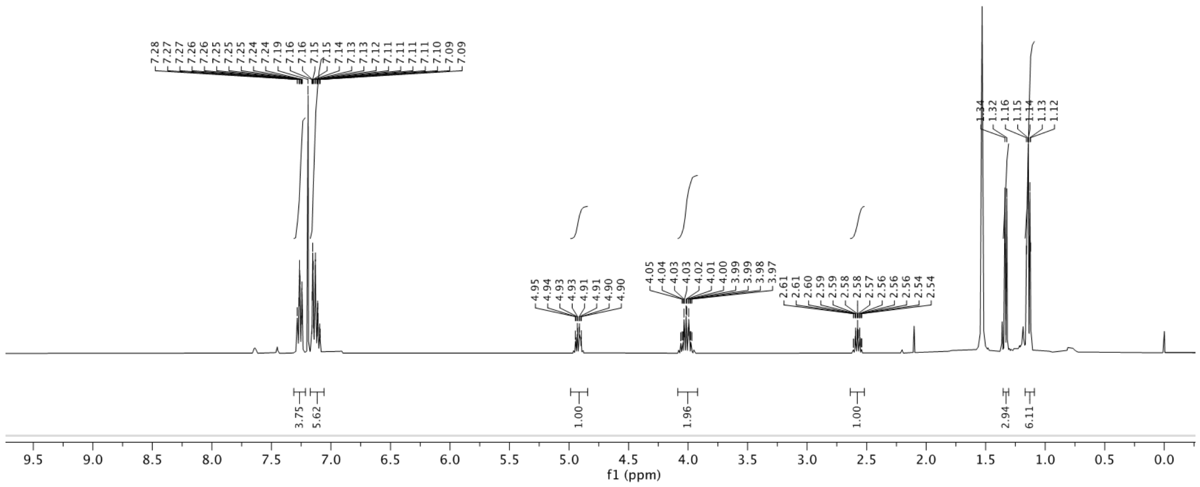

Figure S173. ^{1}H NMR spectrum of ethyl 3-((diphenylphosphoryl)oxy)-2-methylbutanoate (from **S82**) in $CDCl_3$.

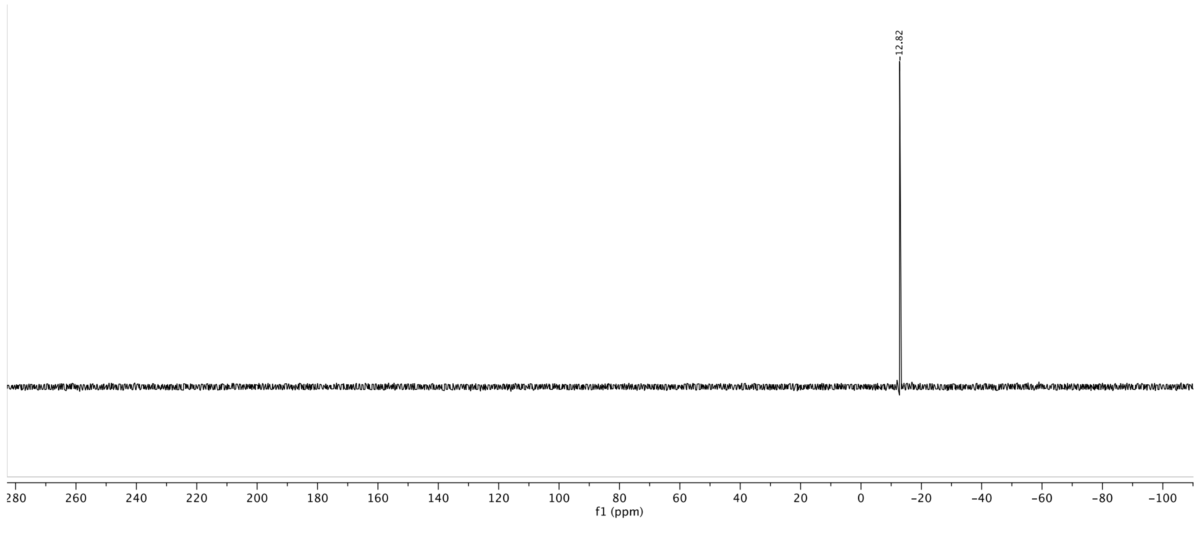

Figure S174. $^{31}P\{^1H\}$ NMR spectrum of ethyl 3-((diphenylphosphoryl)oxy)-2-methylbutanoate (from **S82**) in $CDCl_3$.

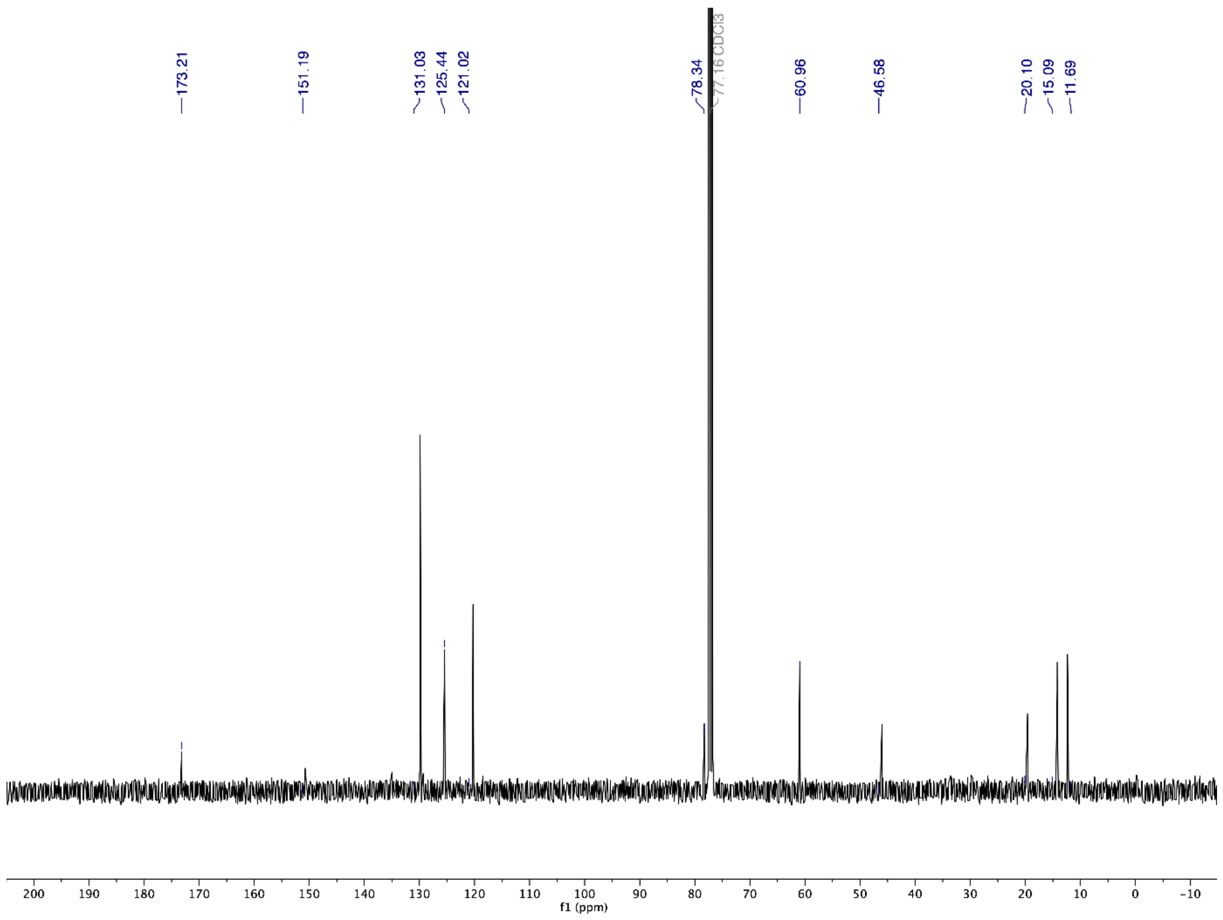

Figure S175. $^{13}C\{^{1}H\}$ NMR spectrum of ethyl 3-((diphenylphosphoryl)oxy)-2-methylbutanoate (from **S82**) in $CDCl_3$.

(a)

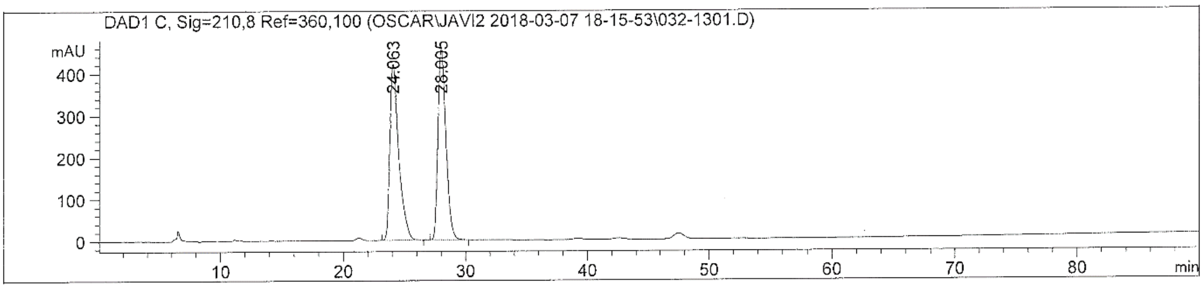

(b)

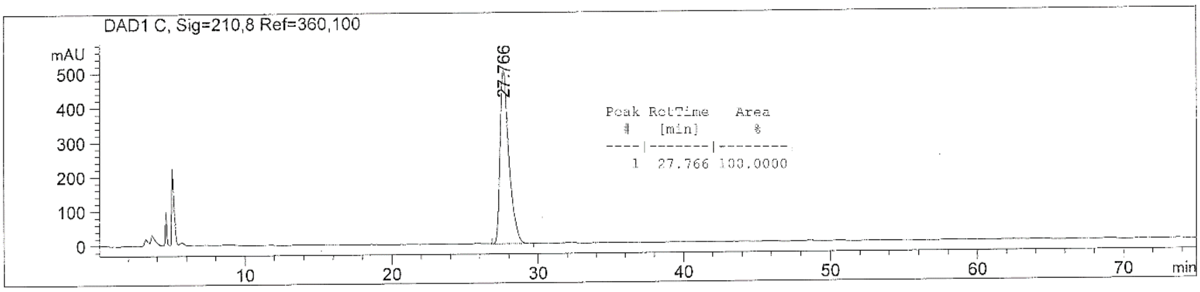

Figure S176. HPLC traces of (a) racemic and (b) enantioenriched product (>95% ee using **4c** catalytic system; Scheme 5).

Butane-2,3-diyldibenzene (from S83).[38] Enantiomeric excess determined by HPLC using Chiracel OJ-H column (hexane/2-propanol=98/2, 0.25 mL/min, 254 nm). t_R 18.0 min (*R,R*); t_R 19.1 min (*S,S*). ^{1}H NMR ($CDCl_3$), δ: 1.26-1.31 (m, 6H), 2.91-2.98 (m, 2H), 7.00-7.02 (m, 4H), 7.07-7.11 (m, 2H), 7.14-7.19 (m, 4H).

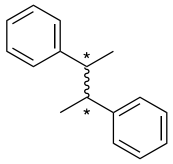

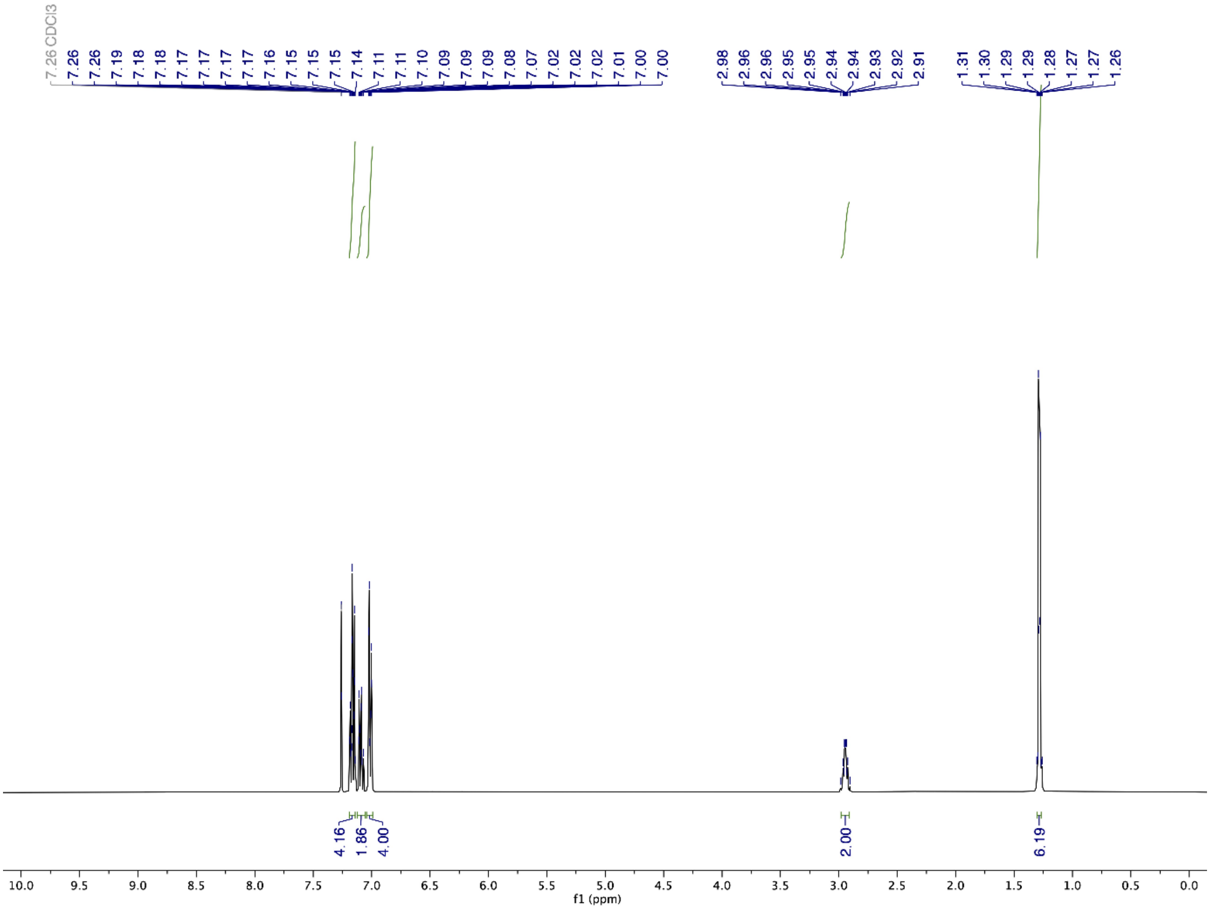

Figure S177. ^{1}H NMR spectrum of Butane-2,3-diyldibenzene (from **S83**) in $CDCl_3$.

(a)

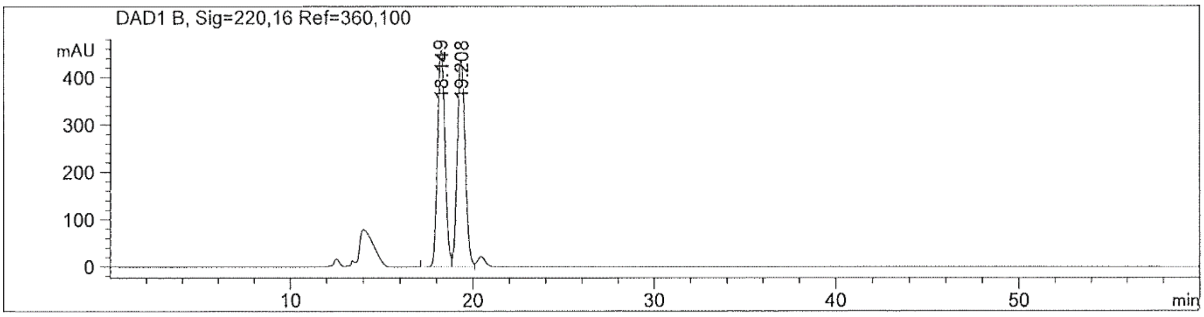

(b)

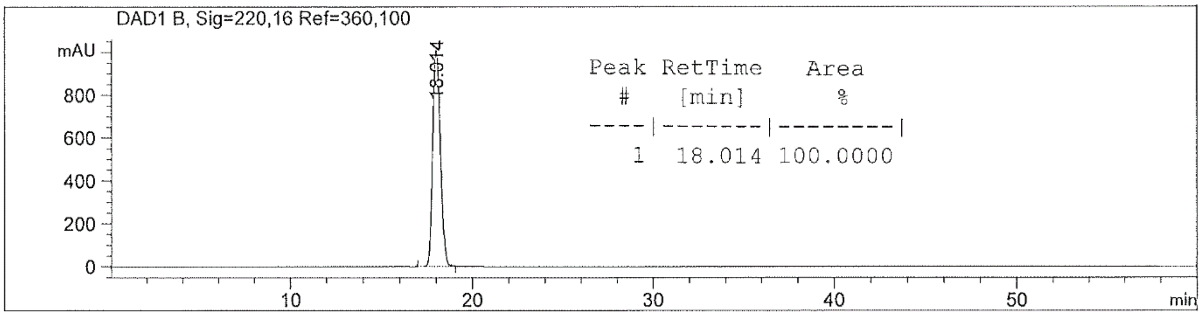

Figure S178. HPLC traces of (a) racemic and (b) enantioenriched product (99% (*R*,*R*) ee using **4c** catalytic system; Scheme 6).

(2,3-Dimethylbutane-1,1-diyl)dibenzene (from S84).[38] Enantiomeric excess determined by HPLC

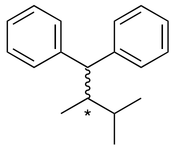

using Chiracel OJ-H column (hexane/2-propanol=99/1, 0.3 mL/min, 220 nm). t_R 44.0 min (*S*); t_R 47.8 min (*R*). ^{1}H NMR ($CDCl_3$), δ: 0.70 (d, 3H, *J*= 6.9 Hz), 0.76 (d, 3H, *J*= 6.9 Hz), 0.91 (d, 3H, *J*= 7.0 Hz), 1.68-1.71 (m, 1H), 2.35-2.36 (m, 1H), 3.63 (d, 1H, *J*= 11.6 Hz), 7.12-7.16 (m, 2H), 7.22-7.33 (m, 8H).

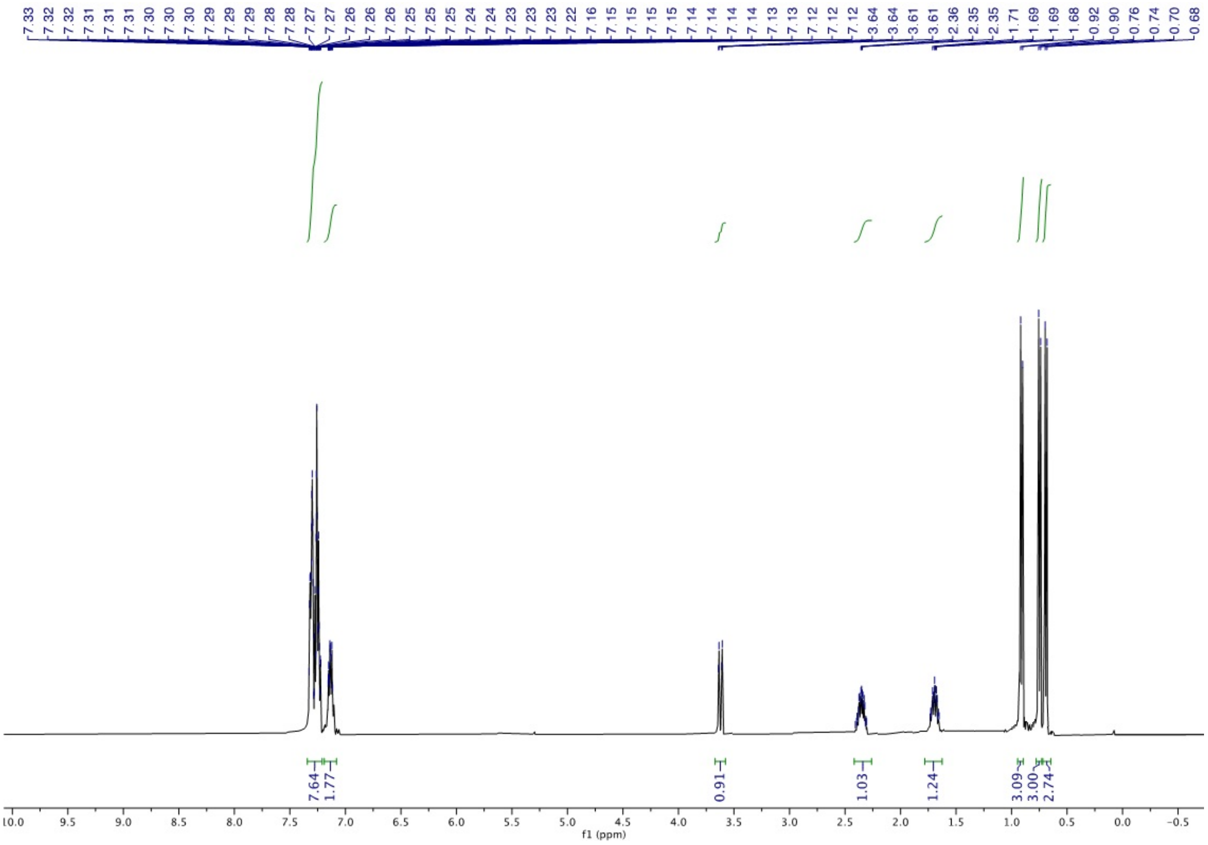

Figure S179. ^{1}H NMR spectrum of (2,3-dimethylbutane-1,1-diyl)dibenzene (from **S84**) in $CDCl_3$.

(a)

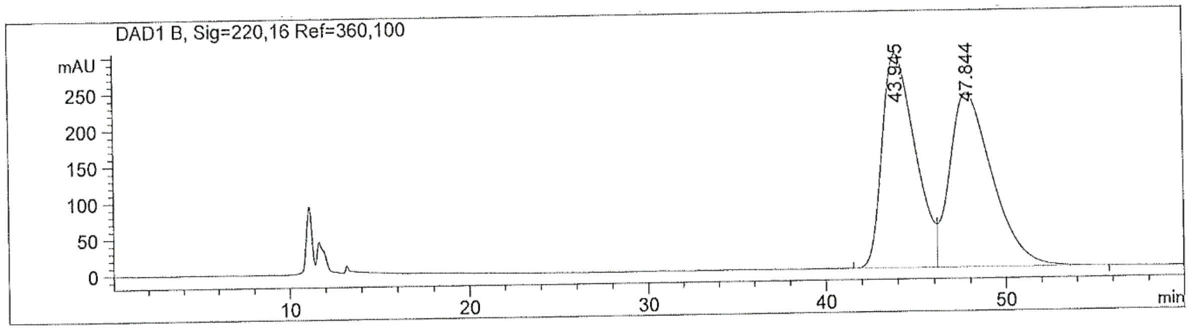

(b)

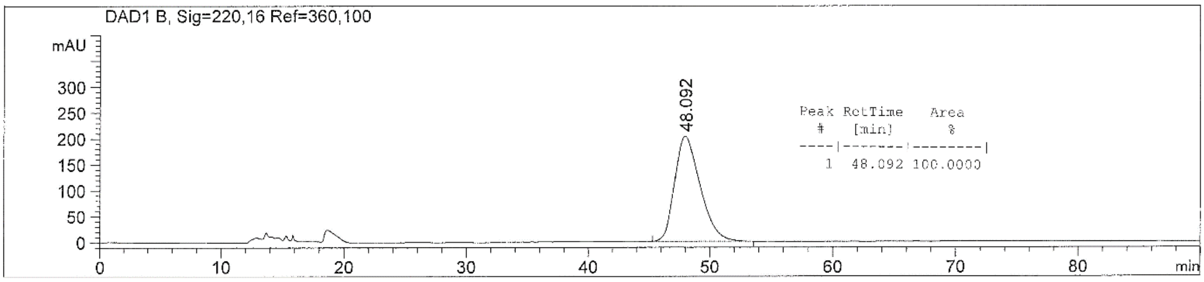

Figure S180. HPLC traces of (a) racemic and (b) enantioenriched product (>95% (*R*) ee using **4c** catalytic system; Scheme 6).

(2-Methylbutane-1,1-diyl)dibenzene (from S85).[38] Enantiomeric excess determined by HPLC using Chiracel OJ-H column (hexane/2-propanol=98/2, 0.25 mL/min, 220 nm). t_R 36.6 min (*S*); t_R 39.9 min (*R*). ^{1}H NMR ($CDCl_3$), δ: 0.84 (d, 3H, *J*= 6.6 Hz), 0.87 (dd, 3H, *J*= 7.4 Hz, *J*= 7.4 Hz), 1.02-1.05 (m, 1H), 1.44-1.46 (m, 1H), 2.28-2.30 (m, 1H), 3.53 (d, 1H, *J*= 10.9 Hz), 7.12-7.16 (m, 2H), 7.23-7.31 (m, 8H).

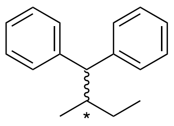

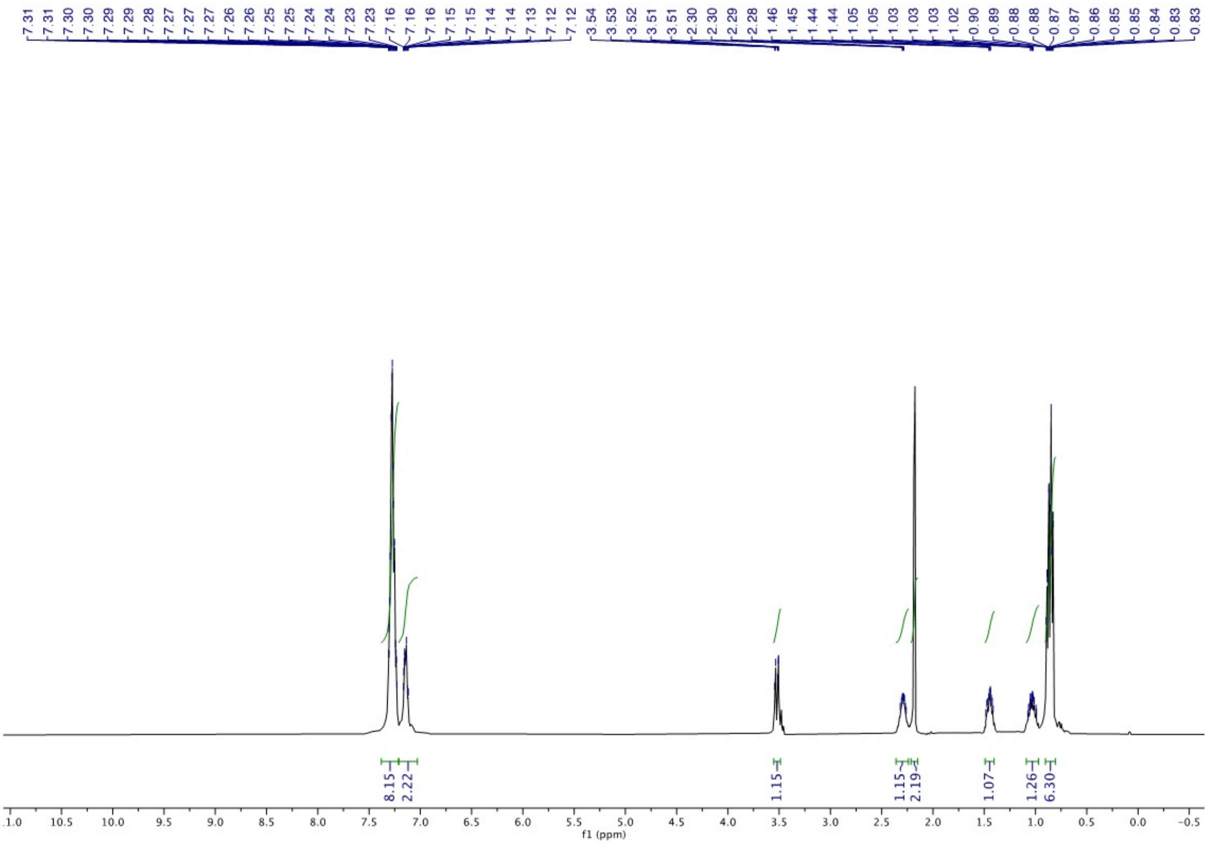

Figure S181. ^{1}H NMR spectrum of (2-methylbutane-1,1-diyl)dibenzene (from **S85**) in $CDCl_3$.

(a)

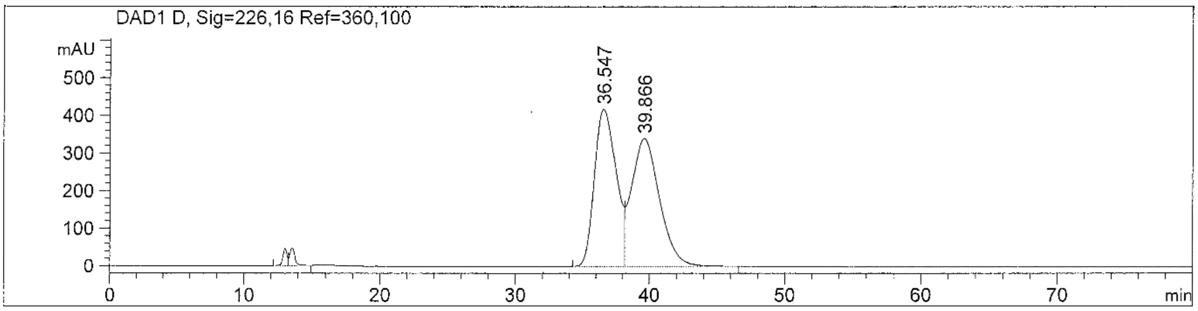

(b)

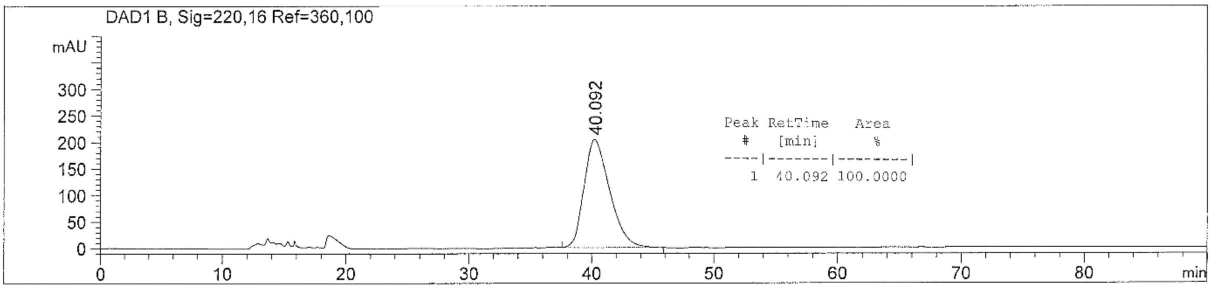

Figure S182. HPLC traces of (a) racemic and (b) enantioenriched product (>95% (*R*) ee using **4c** catalytic system; Scheme 6).

1-Methoxy-4-(2-methyl-1-phenylpropyl)benzene (from S86).[38] Enantiomeric excess determined by HPLC using Chiracel IB column (hexane/2-propanol=98/2, 0.5 mL/min, 210 nm). t_R 8.5 min (*S*); t_R 8.8 min (*R*). ^{1}H NMR ($CDCl_3$), δ: 0.87 (d, 3H, *J*= 8.2 Hz), 0.88 (d, 3H, *J*= 8.3 Hz), 2.39-2.48 (m, 1H), 3.36 (d, 1H, *J*= 10.8 Hz), 3.76 (s, 3H), 6.80 (d, 2H, *J*= 8.7 Hz), 7.13 (ddt, 1H, *J*= 6.5 Hz, *J*= 5.4 Hz, *J*= 2.6 Hz), 7.19 (d, 2H, *J*= 8.6 Hz), 7.24-7.26 (m, 4H).

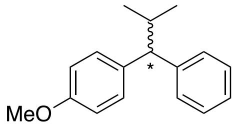

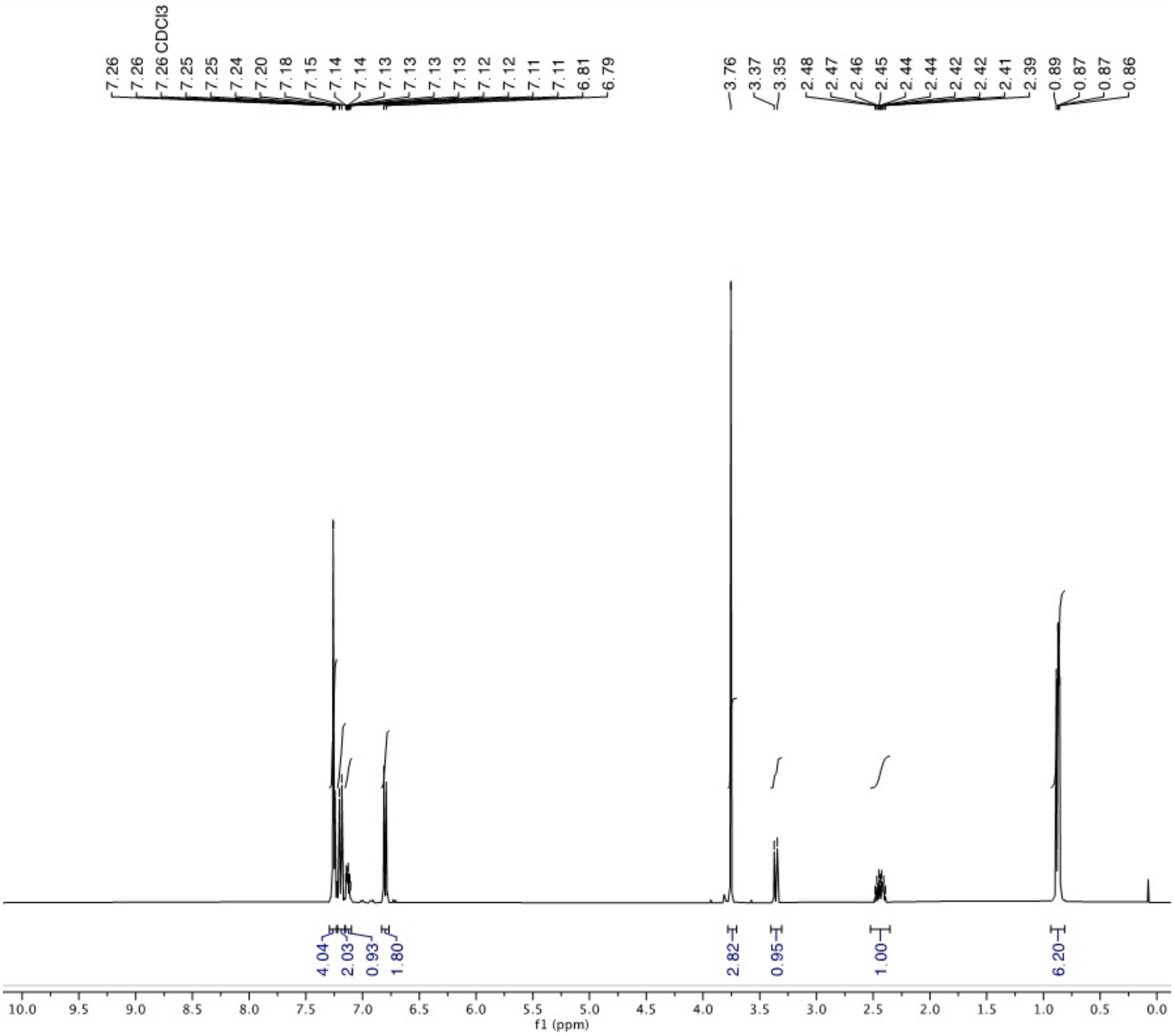

Figure S183. ^{1}H NMR spectrum of 1-methoxy-4-(2-methyl-1-phenylpropyl)benzene (from **S86**) in $CDCl_3$.

(a)

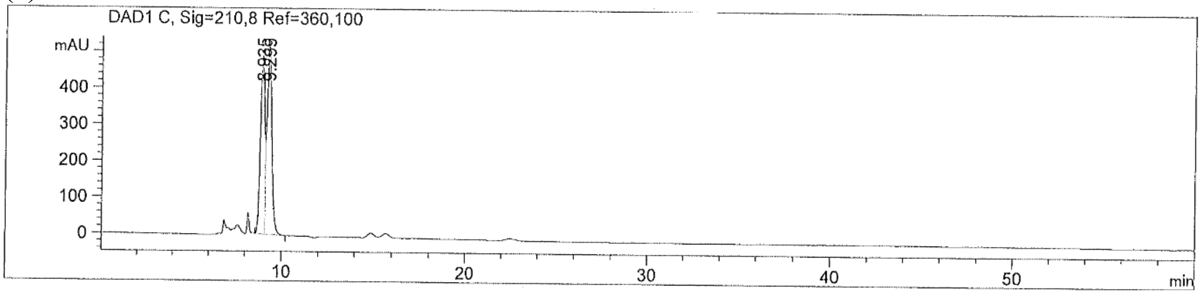

(b)

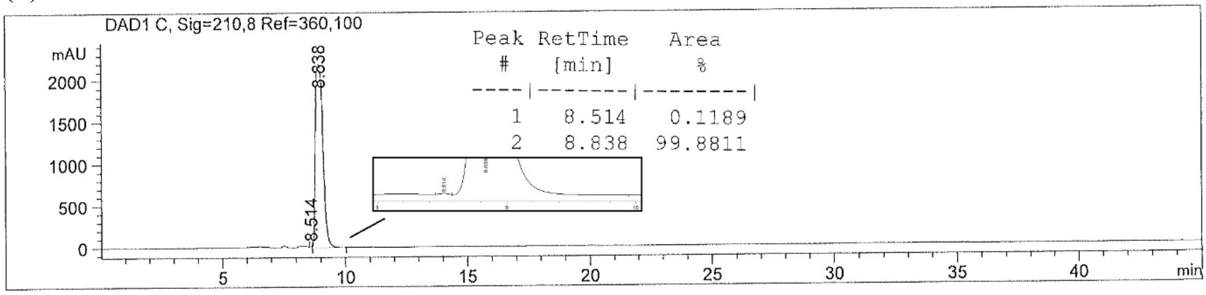

Peak #	RetTime [min]	Area %
1	8.514	0.1189
2	8.838	99.8811

Figure S184. HPLC traces of (a) racemic and (b) enantioenriched product (>95% (*R*) ee using **4c** catalytic system; Scheme 6).

10. Cartesian coordinates

Ir/4c with S2

TSs for the σ-methatesis pathway

A

N	1.47649200	1.02250200	0.56677000
P	1.00820000	-1.95207900	-0.58922300
Ir	-0.32803100	-0.06199100	-0.45587700
H	-1.34925600	0.48519100	0.75632600
C	-1.52925800	1.62878400	-1.33314000
C	-2.11064100	1.53148500	-0.01274000
C	2.40237800	2.56831300	2.06651200
H	1.60129900	2.92062700	2.72282900
H	3.30327700	3.15432300	2.23381100
C	2.05295700	0.42044300	1.55371800
O	2.70136200	1.18877100	2.43839800
C	1.96077600	2.44064800	0.60005300
C	2.07374800	-1.07477800	1.82085900
N	2.25548300	-1.79748400	0.55095200
H	2.80497400	-2.64098700	0.66862300
C	0.01426300	-3.43891000	-0.16976900
H	-0.83805400	-3.50777400	-0.84896200
H	0.60630600	-4.35497400	-0.22467100
H	-0.36398100	-3.30859700	0.84607700
C	1.88919000	-2.36097900	-2.19184600
C	0.82261600	-2.71201400	-3.24762100
H	0.10005000	-1.89969800	-3.37204800
H	1.31876000	-2.87198700	-4.21047500
H	0.27994800	-3.62875800	-2.99983800
C	2.71465500	-1.15163400	-2.65934100
H	3.30936800	-1.44476600	-3.53093500
H	2.07299100	-0.32066700	-2.95645000
H	3.39801800	-0.80305800	-1.88111600
C	2.83489400	-3.56206600	-1.96955900
H	3.29266600	-3.82393800	-2.92899600
H	3.64986000	-3.31851600	-1.28074700
H	2.31562100	-4.45269100	-1.60561200
H	0.24980800	0.13407100	-1.96134800
H	-1.48930200	-0.92119800	-1.06363600
C	3.15599900	-1.49541400	2.84909700
H	3.01119800	-0.83678800	3.71110700
C	4.59065100	-1.30206800	2.33492400
H	5.30306000	-1.54625800	3.12836100
H	4.81105600	-1.95553300	1.48446200
H	4.76986600	-0.27053200	2.02425900
C	2.91552400	-2.93765000	3.32236400
H	3.61982300	-3.18969000	4.12012500
H	1.90027100	-3.06779000	3.71106200
H	3.06428400	-3.67052700	2.52090900
H	1.09958600	-1.30303700	2.27921400
C	3.09940200	2.71450800	-0.43575500
C	4.31008300	1.79355100	-0.19074800
H	4.74711600	1.94004300	0.80181500
H	4.03753700	0.73911000	-0.29129700
H	5.09088300	2.00657300	-0.92699100
C	3.53433700	4.18862000	-0.30294900
H	4.25860200	4.42900600	-1.08765400
H	2.67998600	4.86518000	-0.41873300
H	4.01284600	4.40553400	0.65702500
H	1.13104800	3.11459900	0.38854600
C	2.57273000	2.49522400	-1.86497500
H	2.06418600	1.53656800	-1.95843800
H	1.87224600	3.28338800	-2.15044200
H	3.40250900	2.51912200	-2.57859500
H	-0.54160400	-0.82017100	0.98577100
C	-3.47155100	0.89299900	0.12055100
C	-3.82876700	0.15957000	1.25783300
C	-4.45153600	1.10682700	-0.86256300
C	-5.10860700	-0.36729700	1.41765200
H	-3.09018800	-0.02711400	2.03323600
C	-5.73443900	0.59342000	-0.71686500
H	-4.22112000	1.69899900	-1.74231300
C	-6.07449700	-0.15155800	0.42224000
H	-5.33830900	-0.94044300	2.30722400
H	-6.49355700	0.76354300	-1.47303000
C	-1.84309100	2.64692200	0.99616400
H	-2.09513900	1.12359400	-2.10916000
H	-0.79080100	2.92329900	1.03916600
H	-2.16865700	2.35602800	1.99627400
O	-7.35106600	-0.61396300	0.46916100
C	-7.76085300	-1.36514000	1.61176900
H	-7.68123800	-0.77184600	2.53048200
H	-8.80550000	-1.62543500	1.43955700
H	-7.17104600	-2.28316100	1.71986300
C	-0.86163600	2.88868600	-1.82434400
H	-0.16654000	3.32287800	-1.10909100
H	-0.32207300	2.69753700	-2.75208700
H	-1.63644500	3.64029600	-2.02701200
H	-2.42299300	3.52436300	0.69074700

B

N	0.14687400	0.83024900	0.17974700
P	-2.79255700	-0.36892100	-0.40384900
Ir	-0.65674000	-1.25657700	-0.33820400
H	0.52250300	-1.50839800	-1.48806600
C	0.90284000	-2.49536700	0.69292300
C	1.51435400	-2.32941400	-0.60086400
C	1.77172300	2.52437700	0.14402400
H	2.49923600	2.31618600	-0.64326100
H	2.10807300	3.36184500	0.75127700
C	-0.26996400	1.85447000	-0.48461300
O	0.52698000	2.92958500	-0.50297600
C	1.37166300	1.26052800	0.92683400
C	-1.55839400	1.96751300	-1.28184100
N	-2.66071500	1.31397000	-0.55984700
H	-3.54792600	1.77392800	-0.72933500
C	-3.72160000	-1.01395700	-1.84891700
H	-3.81737700	-2.09912000	-1.77013700
H	-4.71114700	-0.55956300	-1.92652200
H	-3.14239100	-0.78072600	-2.74427300
C	-3.90896800	-0.59744500	1.08484100
C	-4.11640300	-2.10794900	1.30970100
H	-3.16566100	-2.62451100	1.46819400
H	-4.73427600	-2.25066200	2.20241100
H	-4.63515000	-2.58285800	0.47141500
C	-3.25088400	0.04128300	2.32014600
H	-3.92196900	-0.07426200	3.17778700
H	-2.30401200	-0.44102600	2.56989500
H	-3.06744400	1.10803700	2.16963800
C	-5.26992000	0.08767500	0.83007300
H	-5.90149200	-0.06000400	1.71210800
H	-5.17083700	1.16842700	0.68662100
H	-5.80298400	-0.33560000	-0.02471600
H	-1.14761500	-1.51315500	1.18486700
H	-1.28319800	-2.63653600	-0.75634500
C	-1.93146900	3.43772400	-1.60899200
H	-1.03329700	3.87584100	-2.05445700
C	-2.29503600	4.25455300	-0.35924600
H	-2.47220700	5.29772200	-0.63687400
H	-3.20635600	3.88165000	0.11921900
H	-1.49110800	4.23218900	0.38024300
C	-3.04575300	3.49377500	-2.66543200
H	-3.21821600	4.53059400	-2.96775600
H	-2.78142500	2.91858700	-3.55870000
H	-4.00111000	3.11006000	-2.28829100
H	-1.34903100	1.46624700	-2.24049500
C	1.11569000	1.50221400	2.44641500
C	0.01268500	2.55289000	2.66712700
H	0.27315800	3.52065600	2.22619100
H	-0.93648700	2.22433000	2.23412600
H	-0.14517300	2.71239000	3.73819200
C	2.43866900	1.98001800	3.08156600
H	2.32635600	2.04189400	4.16850400
H	3.25345800	1.27917700	2.86605900
H	2.74264100	2.97103900	2.73101100
H	2.13448800	0.49233500	0.82252700
C	0.71134000	0.18449300	3.12907800
H	-0.16561300	-0.25842100	2.65717000
H	1.52961400	-0.54312500	3.09238000
H	0.48108900	0.36245400	4.18446400
H	-0.80061500	-0.73292900	-1.88882100
C	0.44238900	-3.84104400	1.20856200
H	-0.16894500	-4.38324600	0.48629400
H	1.31844500	-4.45817000	1.44729900
H	-0.14561800	-3.71197200	2.11894900
C	1.55226700	-3.50783600	-1.56962100
H	2.33354100	-4.19541800	-1.22715300
H	0.60196200	-4.04090000	-1.60388200
H	1.81079300	-3.17805900	-2.57789900
H	1.30509600	-1.84442500	1.46111800
C	2.69143100	-1.39720000	-0.69992000
C	2.85994200	-0.51247600	-1.77804000
C	3.68673100	-1.43879000	0.28393100
C	3.96175300	0.32644100	-1.84834500
H	2.10026300	-0.45615900	-2.55251300
C	4.80098600	-0.59831500	0.23058700
H	3.59634800	-2.13199300	1.11427800
C	4.93770300	0.30101900	-0.83548800
H	4.08382600	1.02539000	-2.66904400
H	5.54422800	-0.65468900	1.01573800
O	5.96031300	1.18042400	-0.98189700
C	6.96007700	1.23562900	0.03690900
H	7.65795100	2.01423300	-0.27137600
H	6.52387500	1.49997400	1.00767100
H	7.49452500	0.28254600	0.12504200

C

N	-0.01678500	0.88770400	-0.34822900
P	-2.75977000	-0.62810400	0.35074800
Ir	-0.58290400	-1.30669700	-0.05319600
H	-1.36879800	-1.18790100	-1.47768300
C	1.60942700	-2.27667600	-0.62779500
C	0.71871900	-1.99587300	-1.73252700
C	1.27998100	2.57106100	-1.32583100
H	1.77759300	2.15305400	-2.20586800
H	1.66552300	3.56745300	-1.12066500
C	-0.74279300	1.64271200	-1.10050700
O	-0.14016900	2.70803900	-1.64598300
C	1.28059000	1.60241300	-0.13352000
C	-2.20546200	1.43610800	-1.45026100
N	-2.93882600	0.93807000	-0.27535900
H	-3.89607600	1.26962700	-0.26100000
C	-3.94515300	-1.74799300	-0.49252600
H	-3.77047500	-2.77226000	-0.15646600
H	-4.98253500	-1.46820600	-0.29874900
H	-3.74609100	-1.69672900	-1.56470200
C	-3.37369300	-0.47683800	2.11597900
C	-3.43581500	-1.89098200	2.72476900
H	-2.46766600	-2.39731900	2.65860000
H	-3.70228700	-1.80948900	3.78373000
H	-4.19172700	-2.51691400	2.24180200
C	-2.41333900	0.40581400	2.92910200
H	-2.84592700	0.58312300	3.91936900
H	-1.44640200	-0.07983900	3.07077700
H	-2.25012300	1.37378000	2.44839800
C	-4.77658900	0.17146800	2.12087100
H	-5.15139600	0.17826900	3.14957600
H	-4.74476200	1.21226000	1.78394200
H	-5.50426000	-0.37578200	1.51559800
H	0.86219500	-1.77438500	0.65496100
H	-1.05622100	-2.79934700	0.09253500
C	-2.87616700	2.72465400	-1.99535500
H	-2.22485700	3.07244900	-2.80303500
C	-2.97582100	3.84060100	-0.94419900
H	-3.39033300	4.74381100	-1.40152300
H	-3.63380900	3.55945600	-0.11558500
H	-1.99626200	4.08954800	-0.52967000
C	-4.24815400	2.40572400	-2.60875700
H	-4.65307000	3.29705600	-3.09615800
H	-4.17998700	1.60977300	-3.35739700

H	-4.98044400	2.09679600	-1.85342300
H	-2.20759900	0.69953000	-2.26951400
C	1.37250100	2.29212600	1.26260300
C	0.24978600	3.32712200	1.45799400
H	0.29389500	4.12893800	0.71409800
H	-0.73704100	2.85902900	1.39709000
H	0.33731900	3.79021300	2.44565800
C	2.74708400	2.98675900	1.36042700
H	2.88241700	3.39813700	2.36575200
H	3.56046700	2.27793200	1.17327800
H	2.85069900	3.81656800	0.65414400
H	2.09507100	0.88813600	-0.21618900
C	1.28465600	1.22894100	2.36979000
H	0.34788300	0.67799200	2.31097700
H	2.10412900	0.51021000	2.29007700
H	1.34567200	1.70533500	3.35388500
H	-0.49950800	-1.23466500	1.57011400
C	2.83998900	-1.43138600	-0.45658600
C	3.43330200	-1.26887900	0.80199000
C	3.46172200	-0.82177400	-1.56154100
C	4.56823600	-0.48241500	0.98188300
H	2.98013800	-1.73172700	1.67399600
C	4.59538500	-0.03582500	-1.40059900
H	3.06322700	-0.96314500	-2.56006000
C	5.14865600	0.15777000	-0.12480100
H	4.97747200	-0.36301900	1.97694600
H	5.07034800	0.44114300	-2.25113700
C	1.77153200	-3.72385900	-0.17546400
H	2.42838100	-4.22340600	-0.89639200
H	2.24359200	-3.78446300	0.80614800
H	0.81881600	-4.25044900	-0.13927200
C	0.16854600	-3.10824800	-2.60287800
H	0.98852000	-3.57005100	-3.16819400
H	-0.32695700	-3.88834600	-2.02431200
H	-0.55585900	-2.70610200	-3.31339200
H	0.94986200	-1.08612000	-2.28174200
O	6.23037200	0.97269800	-0.06507300
C	6.82180200	1.22880300	1.21074800
H	7.20392700	0.30792000	1.66597000
H	7.65094500	1.91087800	1.02270400
H	6.10660000	1.70416400	1.89241600

D

N	-1.58808900	1.15313300	-0.19392000
P	-1.07364000	-2.03615200	0.03149100
Ir	0.27953800	-0.18476400	-0.28049500
H	-0.21780700	-0.65451000	-1.76482200
C	2.06524800	1.45127900	-0.60005000
C	1.36835600	1.05409600	-1.79935500
C	-2.93804300	3.02392700	-0.64865400
H	-2.60606800	3.53005600	-1.55931700
H	-3.69599600	3.62500800	-0.15095200
C	-2.66891200	0.80595500	-0.80678900
O	-3.56540900	1.77121400	-1.04741600
C	-1.76863700	2.57082600	0.24650200
C	-3.02540400	-0.58020500	-1.31663300
N	-2.67119100	-1.59183300	-0.31001300
H	-3.31869700	-2.37115000	-0.30947600
C	-0.59125000	-3.39831900	-1.09948500
H	0.43062900	-3.71530900	-0.87970600
H	-1.27025500	-4.24855200	-1.01040200
H	-0.62285500	-3.01175000	-2.11963800
C	-1.17164200	-2.79257900	1.74635600
C	0.24608600	-3.21015700	2.18151900
H	0.92879900	-2.35646100	2.20336100
H	0.19630900	-3.63048400	3.19138600
H	0.67075000	-3.97688700	1.52655200
C	-1.74723900	-1.75491500	2.72510700
H	-1.79780400	-2.19987400	3.72439300
H	-1.12016000	-0.86342400	2.78954800
H	-2.75463600	-1.44788800	2.43421400
C	-2.09658200	-4.03026600	1.72279400
H	-2.16666100	-4.42893100	2.74003300
H	-3.11548700	-3.78506100	1.40617600
H	-1.71219800	-4.83090800	1.08636600
H	1.24928200	0.86613100	0.59318300
H	1.48553100	-1.18575200	-0.39016800
C	-4.52096600	-0.71294100	-1.70582100
H	-4.72561200	0.12804300	-2.37537900
C	-5.46795800	-0.60787600	-0.49981500
H	-6.50655600	-0.62786400	-0.84276100
H	-5.33617400	-1.44316800	0.19581400
H	-5.31090200	0.32101300	0.05331600
C	-4.76168700	-2.00670900	-2.49927800
H	-5.79278300	-2.03271600	-2.86290500
H	-4.09421800	-2.07908700	-3.36410400
H	-4.61740500	-2.90459700	-1.88688200
H	-2.43631600	-0.70549600	-2.23872900
C	-2.02429200	2.72058900	1.77706100
C	-3.26358600	1.91798200	2.21146200
H	-4.17268300	2.26043000	1.70590900
H	-3.13580500	0.85251400	2.00226500
H	-3.42407700	2.03082100	3.28797400
C	-2.22983000	4.21857400	2.08501600
H	-2.33557600	4.36063900	3.16487500
H	-1.37206400	4.81345000	1.75140700
H	-3.12976800	4.62700800	1.61584800
H	-0.86787600	3.13307700	0.00291200
C	-0.79377600	2.23670000	2.56284500
H	-0.61315200	1.17512700	2.40509200
H	0.10888400	2.77929500	2.26118300
H	-0.94387200	2.40491600	3.63412300
H	0.36942000	-0.32243500	1.33549600
C	3.40253600	0.82154500	-0.30388900
C	3.79782700	0.52900200	1.01279100
C	4.32263000	0.60558400	-1.33325000
C	5.05644200	0.01808600	1.28524600
H	3.09873200	0.67924400	1.83091200
C	5.59878200	0.09598900	-1.07535300
H	4.06103900	0.85345200	-2.35731300
C	5.97067900	-0.20565300	0.23998400
H	5.35735500	-0.22317400	2.29929100
H	6.28375000	-0.05290200	-1.90071500
C	1.96534400	2.89297100	-0.11242800
H	2.64848500	3.48652400	-0.73033300
H	0.96739400	3.31223100	-0.21626500
H	2.28518100	2.98678200	0.92662800
C	0.54290800	2.02144900	-2.61872800
H	-0.15947100	2.60478500	-2.02360000
H	1.20928000	2.72624900	-3.13251400
H	-0.02929400	1.47966200	-3.37382000
H	1.88917700	0.31208600	-2.39541000
O	7.17675100	-0.71086000	0.61070900
C	8.14215600	-0.97527200	-0.40657200
H	9.01437800	-1.38134900	0.10637700
H	7.76993500	-1.71113100	-1.12945000
H	8.42714100	-0.05843200	-0.93653000

E

N	-0.47346500	1.10579100	-0.63388300
P	-2.31521000	-1.12071500	0.75700900
Ir	-0.46173600	-1.21157900	-0.61590500
H	-0.32608400	-1.09574700	-2.28269000
C	1.73338300	-1.80090500	-1.16440200
C	1.11500600	-1.35875800	-2.39100200
C	-0.08234200	3.00537800	-1.94114400
H	0.04765200	2.67178800	-2.97509800
H	0.15669000	4.06367700	-1.85980000
C	-1.58519200	1.68308700	-0.94519900
O	-1.49839900	2.84597600	-1.60574600
C	0.61339100	2.09141000	-0.92162500
C	-2.98469500	1.16390800	-0.67473500
N	-3.00767500	0.42372300	0.59681400
H	-3.90530600	0.50699900	1.06065600
C	-3.56138000	-2.36327000	0.23575200
H	-3.12300300	-3.36224700	0.28125000
H	-4.45950300	-2.32288500	0.85558700
H	-3.82623200	-2.14845100	-0.80155400
C	-2.09439400	-1.33890600	2.60393900
C	-1.60174700	-2.77663800	2.86066200
H	-0.67753300	-2.98739400	2.31430500
H	-1.39689600	-2.89398000	3.92992900
H	-2.34982400	-3.52569500	2.58486600
C	-1.05848700	-0.32531300	3.11810400
H	-0.99554800	-0.40428400	4.20863000
H	-0.06725400	-0.52753100	2.70785800
H	-1.33759500	0.70066000	2.86361900
C	-3.43836500	-1.10139400	3.32638800
H	-3.29120200	-1.27583400	4.39719000
H	-3.78689600	-0.06984300	3.21542500
H	-4.22730900	-1.78109900	2.99359000
H	0.29503300	-1.37432800	0.81361200
H	-0.63936700	-2.76720000	-0.65498900
C	-4.05631100	2.28649600	-0.67577400
H	-3.91696700	2.81920200	-1.62160200
C	-3.87962600	3.29134100	0.47129300
H	-4.64958100	4.06577000	0.40748100
H	-3.97355200	2.81069100	1.45050900
H	-2.90438900	3.78052700	0.42545900
C	-5.46998200	1.68373500	-0.68707000
H	-6.21020000	2.47854100	-0.81490700
H	-5.59571400	0.96645200	-1.50472000
H	-5.71386100	1.17116400	0.25095400
H	-3.21892800	0.50850600	-1.52869000
C	1.10249800	2.82889700	0.36606100
C	-0.02223800	3.65441200	1.01731300
H	-0.43058700	4.41682900	0.34690800
H	-0.84270000	3.01102000	1.34957400
H	0.36732300	4.16965100	1.90075600
C	2.27316100	3.75120900	-0.02940100
H	2.67815000	4.23808300	0.86347000
H	3.08255700	3.17676500	-0.49295200
H	1.97105200	4.54218300	-0.72340400
H	1.46348400	1.56736100	-1.36063300
C	1.61069800	1.79687700	1.38121200
H	0.83963300	1.06237400	1.61317400
H	2.47828800	1.26750600	0.99380900
H	1.90702000	2.29602900	2.31007800
H	-1.69551800	-1.09722600	-1.71414900
C	1.93594700	-3.29721600	-0.95794400
H	1.12619800	-3.89497700	-1.37347500
H	2.87409500	-3.58857200	-1.44754800
H	2.02375700	-3.54088500	0.10049100
H	1.36625700	-0.34141500	-2.68637200
C	2.81988400	-0.97651800	-0.54354800
C	3.21282000	-1.20480500	0.78403600
C	3.58617700	-0.06695100	-1.29495800
C	4.30951500	-0.56404400	1.35269200
H	2.62706000	-1.87533400	1.40425100
C	4.67257900	0.59958900	-0.73797500
H	3.36106500	0.10981800	-2.34170300
C	5.04853900	0.35539300	0.59055400
H	4.56301200	-0.76614900	2.38583700
H	5.25856200	1.29879900	-1.32502900
C	0.92150900	-2.30173300	-3.57349900
H	0.40860500	-3.22098400	-3.29036500
H	0.34725100	-1.81689700	-4.36646700
H	1.90803300	-2.56341500	-3.97069500
O	6.12651400	1.04747400	1.04090000
C	6.54896200	0.84559600	2.38975600
H	6.84014300	-0.19655000	2.56662600
H	7.41570800	1.49165200	2.53116500
H	5.76395100	1.12975600	3.10064800

F

N	1.77717100	-1.07359400	-0.13933100
P	0.71086200	1.97800200	-0.13664100
Ir	-0.25020600	-0.04191300	-0.70227500
H	-0.34402000	-0.83171000	-2.18523100
C	-1.87333400	-1.67451700	-0.47223000
C	-1.33759200	-1.85826200	-1.79989700
C	3.45118300	-2.70021500	-0.34141300
H	3.26877300	-3.24898400	-1.27004900
H	4.24064100	-3.18636200	0.22749600
C	2.86301900	-0.56096200	-0.61568200
O	3.92804300	-1.36847000	-0.70245800
C	2.16126600	-2.40176300	0.43936800
C	3.06496800	0.87307100	-1.07572900
N	2.39832200	1.78328600	-0.12596200
H	2.90375700	2.65595500	-0.02075900
C	0.29042300	3.24444000	-1.39784600
H	-0.79455700	3.34299700	-1.46935200
H	0.73596100	4.21342600	-1.16387100
H	0.66959700	2.89164900	-2.35884600
C	0.30318200	2.74809900	1.52006600
C	-1.20067200	3.09090900	1.52195700
H	-1.81500900	2.21247700	1.30162900
H	-1.47721700	3.45747500	2.51605500

```
H   -1.44651500    3.87678700    0.80173800
C    0.61885100    1.75712200    2.65248300
H    0.46255200    2.25813100    3.61367400
H   -0.03833700    0.88701000    2.61782700
H    1.65489400    1.41170000    2.61162500
C    1.14437500    4.02727500    1.72489100
H    0.84979600    4.48683100    2.67396100
H    2.21396700    3.80534700    1.79458900
H    0.98685700    4.77300000    0.94165100
H   -0.69711600    0.01980000    0.85346100
H   -1.50808500    0.79262000   -1.11832900
C    4.55702200    1.25780100   -1.24907100
H    4.99142100    0.46995200   -1.87262300
C    5.33308400    1.30133300    0.07613400
H    6.38948500    1.50515500   -0.12178700
H    4.96576000    2.09191000    0.73868800
H    5.26412300    0.35232700    0.61153800
C    4.68217000    2.58212200   -2.01881500
H    5.73497900    2.79625000   -2.22317400
H    4.15146700    2.54299500   -2.97577200
H    4.28725700    3.43220700   -1.45049000
H    2.60769600    0.92874800   -2.07540400
C    2.35204000   -2.34855600    1.99155900
C    3.41700800   -1.30836700    2.38870800
H    4.39800900   -1.53884500    1.96201800
H    3.13248900   -0.30183900    2.06900200
H    3.52917200   -1.29281600    3.47699800
C    2.78801500   -3.74617500    2.47758800
H    2.81740600   -3.76040100    3.57162400
H    2.08079200   -4.51755200    2.15246600
H    3.78477400   -4.02557000    2.12311400
H    1.39316800   -3.14060300    0.21017200
C    1.01856400   -1.99766500    2.67546700
H    0.55780800   -1.11902600    2.22519300
H    0.31129100   -2.82787000    2.60789000
H    1.18567900   -1.79550000    3.73831100
H    0.49128600    0.38304800   -2.12033600
C   -0.55700000   -3.09766400   -2.20002900
H    0.25204500   -3.32426200   -1.50771800
H   -1.24130300   -3.95293500   -2.21607100
H   -0.12932300   -2.98073700   -3.19812800
C   -1.60207500   -2.73207700    0.57977400
H   -2.29002100   -3.57008200    0.40809600
H   -0.58651400   -3.11752200    0.54624800
H   -1.79087200   -2.34368700    1.57981900
H   -1.97105500   -1.46351400   -2.59324500
C   -3.22622500   -1.03231100   -0.32754500
C   -3.55978700   -0.29306200    0.81542900
C   -4.23170500   -1.23114900   -1.28866500
C   -4.82666600    0.25644300    0.99497800
H   -2.79756400   -0.11254100    1.56659100
C   -5.50219500   -0.68790700   -1.12837300
H   -4.03795800   -1.83952500   -2.16671800
C   -5.81100000    0.06576200    0.01219400
H   -5.03042000    0.83514200    1.88756400
H   -6.27533700   -0.84680800   -1.87290600
O   -7.07664900    0.56106700    0.07477200
C   -7.44817800    1.32894400    1.21831100
H   -8.48740700    1.61871500    1.06001500
H   -7.37256700    0.73922100    2.13984400
H   -6.83085400    2.23044800    1.31357900
```

G

```
N   -1.59467500   -0.87730200    0.57859900
P   -0.55832500    1.89487400   -0.71903900
Ir   0.22675100   -0.27692300   -0.68077200
H    0.76246100    0.44593500    0.67716000
C    1.34864200   -2.38407000   -0.85907300
C    1.76614800   -1.66781000    0.31754900
C   -2.79743000   -2.12635200    2.16544900
H   -2.11476400   -2.63797900    2.84978600
H   -3.81502700   -2.47015800    2.33856900
C   -1.98251300   -0.13040900    1.55648600
O   -2.75653500   -0.70527900    2.48606800
C   -2.32733900   -2.17592900    0.69913900
C   -1.62622200    1.32773100    1.79031000
N   -1.69549300    2.07389900    0.52477800
H   -1.99927300    3.03022400    0.66411500
C    0.81945400    3.07367500   -0.43314400
H    1.56397800    2.96013100   -1.22405800
H    0.47080000    4.10767200   -0.39791000
H    1.28628200    2.81141500    0.51784500
C   -1.45781400    2.50254000   -2.24810100
C   -0.49659000    2.39813700   -3.44795400
H   -0.14834100    1.37185300   -3.59488100
H   -1.02566500    2.71023600   -4.35433400
H    0.37608700    3.04850000   -3.33553900
C   -2.70281200    1.63127600   -2.48463700
H   -3.22507800    1.99297100   -3.37657500
H   -2.43898700    0.58570900   -2.65371900
H   -3.39351400    1.68101500   -1.63924900
C   -1.89912400    3.97001200   -2.05240600
H   -2.43195900    4.29442100   -2.95229400
H   -2.59138200    4.08807600   -1.21269800
H   -1.05524000    4.65019400   -1.91283300
H    0.21445000   -1.62885600   -1.65472500
H    1.46841400    0.24751400   -1.48880800
C   -2.52354300    1.99935000    2.86218300
H   -2.49922100    1.32771100    3.72570400
C   -3.98587000    2.15055400    2.41385400
H   -4.58807600    2.53464000    3.24240900
H   -4.08710700    2.85508600    1.58187900
H   -4.41123700    1.19392800    2.10075500
C   -1.92701600    3.34395500    3.30571600
H   -2.51290700    3.75484000    4.13274700
H   -0.89227800    3.23156200    3.64537900
H   -1.93940200    4.09189200    2.50440300
H   -0.60017700    1.30925600    2.19101600
C   -3.47278800   -2.34564000   -0.34451000
C   -4.50745400   -1.21299000   -0.22154600
H   -4.99548100   -1.20443000    0.75860300
H   -4.04205100   -0.23637700   -0.38045600
H   -5.28980200   -1.33766100   -0.97642500
C   -4.14749200   -3.71094800   -0.09637900
H   -4.90948400   -3.88922100   -0.86130500
H   -3.41930200   -4.52804900   -0.15245200
H   -4.64614500   -3.76691100    0.87595500
H   -1.62078900   -2.99137200    0.55242700
C   -2.88577900   -2.35388800   -1.76584600
H   -2.41635500   -1.40174000   -2.00601600
H   -2.13171600   -3.14026600   -1.88023100
H   -3.67857300   -2.54060400   -2.49723300
H   -0.58280300   -0.39429500   -2.09569500
H    2.01217500   -2.26758600   -1.71284700
C    0.68442200   -3.74474100   -0.80846900
H   -0.02796900   -3.84866000    0.00701600
H    0.18375700   -3.98714800   -1.74797800
H    1.47731600   -4.48467300   -0.64758900
C    1.31422700   -2.15089900    1.68901600
H    0.26413900   -2.43901200    1.71727200
H    1.91863400   -3.02051800    1.97601700
H    1.47089500   -1.37301300    2.43700500
C    3.14536700   -1.06278000    0.31480700
C    3.45524000    0.08544800    1.05521300
C    4.20292000   -1.70909700   -0.34918600
C    4.74926400    0.59570800    1.12232500
H    2.65767700    0.61467300    1.56732700
C    5.50108100   -1.21273000   -0.29866600
H    4.02427100   -2.62964400   -0.89645700
C    5.78702200   -0.05338500    0.43495900
H    4.93437400    1.49563400    1.69594700
H    6.31243500   -1.71748600   -0.81266600
O    7.08404400    0.35642800    0.42060400
C    7.43560300    1.52226500    1.16395500
H    7.22800600    1.39431700    2.23319000
H    8.50767600    1.65843700    1.01867600
H    6.90481100    2.40851800    0.79551300
```

H

```
N    0.23977100    0.37160300    0.80988600
P   -2.48576100    0.30832900   -0.90299700
Ir  -0.86408900   -1.23969300   -0.33519800
H   -0.37602300   -0.57027000   -1.74098700
C    0.63887400   -2.97293600    0.26435900
C    1.08409400   -2.28660500   -0.92280800
C    2.16528400    1.40214800    1.68074300
H    3.06891400    1.08116100    1.16087500
H    2.42616500    1.99697600    2.55334700
C    0.40747500    1.54184000    0.29787700
O    1.42302700    2.27031100    0.77226500
C    1.19264800    0.23593200    1.95111600
C   -0.39948300    2.15590200   -0.83154500
N   -1.82697700    1.85047100   -0.65941200
H   -2.42142200    2.60676300   -0.97628800
C   -2.97870200    0.14351200   -2.66221000
H   -3.38317700   -0.85652900   -2.83366100
H   -3.71637600    0.89578000   -2.94783700
H   -2.08077300    0.26270700   -3.27130500
C   -4.07787300    0.34037500    0.09073300
C   -4.76771400   -1.02995500   -0.04942100
H   -4.12839400   -1.84119600    0.30966700
H   -5.68344900   -1.02920500    0.55088800
H   -5.05271800   -1.24491800   -1.08371300
C   -3.74507400    0.62418300    1.56550000
H   -4.67494200    0.63616200    2.14353400
H   -3.09814900   -0.14576700    1.99065200
H   -3.25320900    1.59251800    1.68592000
C   -5.00999900    1.45227900   -0.44034300
H   -5.91807900    1.46480400    0.17133600
H   -4.55934500    2.44686200   -0.36141400
H   -5.31807700    1.28479800   -1.47544100
H   -0.62681500   -2.36117400    0.88037100
H   -1.63357800   -2.31299000   -1.19330400
C   -0.18097300    3.68512500   -0.95935600
H    0.90448500    3.82120200   -0.99211200
C   -0.72631100    4.47080400    0.24345000
H   -0.47544500    5.53037800    0.13840500
H   -1.81613400    4.39753400    0.31774800
H   -0.29792900    4.11310600    1.18301900
C   -0.75375700    4.20828600   -2.28532000
H   -0.49576700    5.26365900   -2.41073300
H   -0.35180700    3.65615300   -3.14115500
H   -1.84722500    4.14105800   -2.32449500
H    0.00073700    1.69377800   -1.74749500
C    0.50423400    0.27508500    3.34821900
C   -0.29345000    1.57824100    3.53363200
H    0.34739200    2.46438100    3.47667600
H   -1.07440800    1.67131800    2.77351000
H   -0.77744100    1.58673200    4.51513200
C    1.60342100    0.16468700    4.42593400
H    1.14434100    0.12797100    5.41868900
H    2.19313400   -0.74985200    4.29563600
H    2.29143700    1.01527700    4.41895300
H    1.71918200   -0.71469300    1.85381400
C   -0.43854800   -0.92961600    3.50676300
H   -1.26911000   -0.88034600    2.80460700
H    0.09231800   -1.87474700    3.34233000
H   -0.85078700   -0.95243300    4.52058600
H   -1.89779200   -1.55622700    0.88948500
H    1.18245200   -2.71450900    1.17167700
C    0.14294200   -4.40686100    0.23470000
H   -0.30609500   -4.68462900    1.19081900
H   -0.59227900   -4.57100400   -0.55279600
H    1.00017700   -5.06452600    0.05277100
C    0.96566100   -2.99024600   -2.26992400
H    1.77942900   -3.72190400   -2.35443800
H    0.01517200   -3.50863400   -2.39285200
H    1.07314000   -2.27401400   -3.08502600
C    2.29457000   -1.39819000   -0.82398400
C    2.44507600   -0.26892100   -1.63963200
C    3.37642300   -1.74463800    0.00543300
C    3.59991300    0.51006400   -1.62249400
H    1.62768200    0.02034200   -2.29219000
C    4.53864400   -0.98070700    0.03777000
H    3.32585900   -2.63631500    0.62277300
C    4.66165100    0.15670400   -0.77454000
H    3.65785000    1.38419200   -2.25882800
H    5.36911500   -1.25444100    0.67983100
O    5.82850900    0.84373600   -0.66486900
C    5.99923100    2.02161300   -1.45354100
H    6.98789100    2.40755200   -1.20427800
H    5.95745400    1.79580000   -2.52557900
H    5.24229800    2.77727600   -1.21105500
```

TSs for the migratory insertion pathway

I

N	-0.66387900	1.19960500	-0.65025400
P	-2.19389800	-1.26829900	0.73026900
Ir	-0.58877000	-1.12484100	-0.98951800
H	-1.21673500	-0.75311600	-2.48833500
C	1.74886900	-1.78417100	-0.89070700
C	1.24523400	-1.23129600	-2.15534300
C	-0.61882200	3.15157600	-1.94516000
H	-0.66719600	2.84921500	-2.99547000
H	-0.42143100	4.21913800	-1.87527200
C	-1.84317200	1.72344300	-0.73423800
O	-1.93618500	2.90715400	-1.35783500
C	0.29819100	2.25327600	-1.10223000
C	-3.16018800	1.12370700	-0.27299900
N	-2.95509500	0.25124800	0.88787600
H	-3.77166500	0.23391100	1.48791900
C	-3.51189500	-2.50010800	0.37352800
H	-3.06643100	-3.49317600	0.27883300
H	-4.28429400	-2.51575100	1.14594400
H	-3.96624400	-2.23557600	-0.58459300
C	-1.60077900	-1.63764700	2.46874300
C	-1.00248300	-3.05773900	2.47229800
H	-0.23212800	-3.17202800	1.70263500
H	-0.53883300	-3.25240400	3.44510200
H	-1.76435400	-3.82591400	2.30968300
C	-0.52051000	-0.60567300	2.83559800
H	-0.17156800	-0.79186500	3.85715200
H	0.34243300	-0.67485800	2.17013600
H	-0.90935200	0.41469000	2.78376900
C	-2.75873500	-1.54481300	3.48248200
H	-2.37539000	-1.78446300	4.48025100
H	-3.17682400	-0.53458100	3.53343200
H	-3.56576200	-2.25037600	3.26621200
H	0.72628000	-1.59720900	0.02744600
H	-0.77573600	-2.64765100	-1.30392800
C	-4.23713600	2.20251400	0.02868800
H	-4.28012400	2.83045500	-0.86637900
C	-3.87615200	3.09402600	1.22452600
H	-4.66553800	3.83335800	1.38948800
H	-3.76689800	2.51266300	2.14571200
H	-2.94270100	3.63377100	1.05080500
C	-5.61862400	1.55169500	0.20257200
H	-6.38640700	2.32721200	0.27430100
H	-5.86804900	0.90405200	-0.64447200
H	-5.68793600	0.95179300	1.11752100
H	-3.53571300	0.56234800	-1.14609000
C	0.98401900	2.99467000	0.08993300
C	-0.03729400	3.74464800	0.96532600
H	-0.60595700	4.49573800	0.40852400
H	-0.74377000	3.04888500	1.42775600
H	0.48604500	4.26373900	1.77417600
C	2.00767200	3.98935000	-0.49313500
H	2.54415700	4.48715600	0.32079200
H	2.74785200	3.47305200	-1.11477600
H	1.53933100	4.77089300	-1.10032000
H	1.07295100	1.78821900	-1.71421400
C	1.72973900	1.98290100	0.96814800
H	1.05416500	1.21272500	1.34126200
H	2.52965700	1.49716100	0.41152200
H	2.17985100	2.48899100	1.82837100
H	-1.97013800	-0.86715700	-1.90260900
C	2.84813000	-1.04343900	-0.17156100
C	3.81006000	-0.33080400	-0.89466300
C	3.01429500	-1.15555800	1.21994200
C	4.89109500	0.28623700	-0.25898900
H	3.73564700	-0.25941000	-1.97446500
C	4.07154800	-0.53849300	1.86791900
H	2.29677300	-1.72003800	1.80796600
C	5.01895900	0.19651000	1.13277500
H	5.61559000	0.82654400	-0.85516500
H	4.18733300	-0.60515800	2.94440100
C	1.93131300	-3.30141400	-0.79215300
H	1.58036500	-0.21144600	-2.34169100
H	2.06584100	-3.61342500	0.24561600
H	1.09132400	-3.84925500	-1.21712300
O	6.01180800	0.77000400	1.85956700
C	6.99217200	1.54920400	1.17382900
H	7.66854700	1.92294600	1.94281600
H	7.55734100	0.94113700	0.45754000
H	6.53311500	2.39585600	0.64949100
C	1.23263700	-2.07305600	-3.41900100
H	0.72298900	-3.02867100	-3.28335700
H	0.72106300	-1.53380600	-4.22047500
H	2.25850200	-2.28001400	-3.75261500
H	2.84275300	-3.56153700	-1.34037800

J

N	1.90147300	-0.99802800	-0.25282500
P	0.59590300	1.93045200	-0.02822300
Ir	-0.17808800	-0.11505600	-0.93935100
H	0.31017400	-0.38790100	-2.48915000
C	-1.89445000	-1.60094600	-0.09287700
C	-1.39351100	-1.85629600	-1.45445800
C	3.65980600	-2.48565000	-0.66341400
H	3.47570400	-2.92079200	-1.65018000
H	4.48943800	-2.99644800	-0.18005900
C	2.94911000	-0.36628400	-0.67248800
O	4.05836800	-1.09320300	-0.86284400
C	2.38248800	-2.35158500	0.17616900
C	3.06151100	1.12097500	-0.96342600
N	2.30261000	1.87052600	0.05151300
H	2.72638200	2.77365700	0.23358600
C	0.17855600	3.32874300	-1.15033400
H	-0.90099400	3.35791000	-1.31285700
H	0.51779300	4.29063400	-0.75822000
H	0.66495800	3.14294100	-2.11117300
C	0.08159400	2.51088900	1.67850800
C	-1.39002700	2.96543300	1.59599200
H	-2.03256000	2.20043300	1.15007300
H	-1.76021500	3.16194700	2.60791700
H	-1.50247300	3.88766500	1.01903800
C	0.21937100	1.34030700	2.66424300
H	-0.04631400	1.67973900	3.67118000
H	-0.44879500	0.51672000	2.40117000
H	1.24255400	0.95649800	2.69319000
C	0.97181400	3.67909000	2.14821900
H	0.59677900	4.04430900	3.11039100
H	2.00779300	3.36213700	2.30206800
H	0.95985400	4.52454000	1.45388700
H	-1.10076800	-0.66045300	0.44644800
H	-1.43839100	0.65187100	-1.46780800
C	4.52854000	1.62166600	-1.04403800
H	5.03105900	0.94465900	-1.74205900
C	5.26930900	1.56258400	0.29939400
H	6.30374500	1.89284200	0.16572300
H	4.80822300	2.21711200	1.04591200
H	5.28696400	0.54873700	0.70262100
C	4.57888100	3.03367500	-1.64931500
H	5.61853300	3.32899300	-1.81652300
H	4.05426600	3.07933000	-2.60949500
H	4.13653200	3.78682500	-0.98678500
H	2.64264500	1.26097000	-1.97337600
C	2.62703100	-2.43410200	1.72099300
C	3.64560400	-1.37931300	2.19362100
H	4.61877200	-1.49924300	1.70894700
H	3.28775700	-0.36299000	2.00273100
H	3.80230200	-1.47844500	3.27204900
C	3.15185300	-3.84519100	2.05818400
H	3.20715100	-3.96584600	3.14476800
H	2.48240300	-4.62109600	1.67006800
H	4.15466300	-4.02987900	1.66127700
H	1.64603600	-3.10492100	-0.10220000
C	1.30272400	-2.22120200	2.47247800
H	0.77408500	-1.34183000	2.10437500
H	0.64867500	-3.08940100	2.36879900
H	1.49325200	-2.08351500	3.54151300
C	-1.69284900	-2.71059500	0.93928800
H	-0.71547300	-3.17815900	0.85319300
H	-1.82405400	-2.34408500	1.95894100
H	-2.45778100	-3.46974200	0.74620900
H	0.63828100	0.51852700	-2.23793400
C	-0.73789900	-3.16979500	-1.82968700
H	0.08775300	-3.44681800	-1.17456000
H	-1.47112600	-3.98700700	-1.79964900
H	-0.34436900	-3.10870700	-2.84733800
C	-3.24281900	-0.92361100	0.05453700
C	-3.51975700	-0.12505100	1.17660900
C	-4.27099300	-1.14267500	-0.86421000
C	-4.76244600	0.46323700	1.35405000
H	-2.74620700	0.04853200	1.91909600
C	-5.53315000	-0.56312700	-0.69901000
H	-4.10819700	-1.78585400	-1.72275700
C	-5.78312400	0.25248100	0.41058800
H	-4.96870400	1.09193600	2.21385700
H	-6.30295000	-0.75968600	-1.43480600
H	-2.06185500	-1.50632500	-2.23658900
O	-6.96426800	0.87519900	0.66877400
C	-8.03858700	0.69737500	-0.25360900
H	-8.87200800	1.27904100	0.14130500
H	-7.77621300	1.06983200	-1.25122400
H	-8.33373400	-0.35629700	-0.32649100

K

N	-1.70100200	-0.94544100	0.35546300
P	-0.43972600	1.88200700	-0.55219100
Ir	0.11461600	-0.37946600	-0.89806000
H	1.02786400	-0.23242800	0.52624800
C	1.23991400	-2.24595600	-0.59070400
C	1.71643900	-1.47006300	0.55443200
C	-2.93221400	-2.32298900	1.79759200
H	-2.25246700	-2.99745300	2.32660600
H	-3.96220900	-2.60633100	2.00355700
C	-1.95625200	-0.33413700	1.46318000
O	-2.73040500	-0.98267100	2.34216500
C	-2.57942600	-2.15621400	0.30935200
C	-1.43696800	1.03038300	1.88499000
N	-1.53275800	1.97297900	0.75806900
H	-1.68548000	2.91993000	1.08741800
C	1.04221800	2.87179500	-0.09250100
H	1.80825700	2.75590800	-0.86199200
H	0.81187100	3.93070700	0.04437000
H	1.43819800	2.46935300	0.84172400
C	-1.28976800	2.86801300	-1.90421300
C	-0.28085100	3.05807500	-3.05369700
H	0.11762600	2.09936100	-3.40182300
H	-0.78740100	3.53791000	-3.89772500
H	0.55807000	3.69740200	-2.76421700
C	-2.51561000	2.08704500	-2.40709100
H	-3.07489600	2.71129100	-3.11201000
H	-2.22510100	1.17776200	-2.93916300
H	-3.18624400	1.81381100	-1.58798200
C	-1.75273900	4.23827000	-1.36712700
H	-2.17753400	4.81592800	-2.19495500
H	-2.53711800	4.13377100	-0.61102100
H	-0.93367300	4.82894100	-0.94750600
H	-0.45402300	-1.16374800	-2.25181700
H	1.36983100	0.07175600	-1.73802600
C	-2.17010200	1.58982700	3.13186700
H	-2.15123000	0.78332200	3.87149800
C	-3.63754900	1.94966200	2.85147000
H	-4.12567800	2.26104000	3.77960500
H	-3.72444500	2.77677300	2.13975900
H	-4.18895000	1.09699400	2.44795900
C	-1.39789300	2.77815900	3.72478900
H	-1.87535800	3.10683100	4.65215900
H	-0.36159000	2.50852500	3.95410600
H	-1.37958200	3.64246600	3.05096200
H	-0.39027100	0.85936400	2.18435700
C	-3.81456000	-1.98833000	-0.62854200
C	-4.72118200	-0.83162000	-0.16969900
H	-5.15327800	-1.01011300	0.82003600
H	-4.17127400	0.11409500	-0.13771700
H	-5.55152200	-0.70898500	-0.87183200
C	-4.59992100	-3.31591800	-0.61440400
H	-5.45464500	-3.24701900	-1.29424800
H	-3.97218000	-4.14997000	-0.94784300
H	-4.99248800	-3.56240400	0.37696200
H	-1.99618800	-3.00004400	-0.05507100
C	-3.34100800	-1.71855700	-2.06571400
H	-2.87554900	-0.73716600	-2.14033300
H	-2.61990100	-2.47352100	-2.39895800
H	-4.19303600	-1.73840600	-2.75248500
H	-0.77804200	-0.24549300	-2.29086800
H	1.93993700	-2.27327500	-1.42128300
C	3.12181600	-0.91530700	0.52083200
C	3.45794700	0.23841400	1.25125500
C	4.14258400	-1.57167700	-0.17156800
C	4.75152100	0.73308600	1.26388100

H	2.69258700	0.76476500	1.81453200
C	5.45583800	-1.09213900	-0.16430900
H	3.93083600	-2.48503400	-0.71713400
C	5.76627900	0.07138800	0.54937500
H	5.00513000	1.63101000	1.81740400
H	6.21741200	-1.63443200	-0.71061100
C	0.54469600	-3.57847300	-0.40038400
H	1.29247200	-4.35985500	-0.20967300
H	-0.14878300	-3.58956700	0.43985400
H	-0.00631900	-3.85736900	-1.30179300
C	1.25880700	-1.89255900	1.95100100
H	0.18962300	-2.09957000	1.98626000
H	1.79867500	-2.80579100	2.22070000
H	1.49236500	-1.12965600	2.69590000
O	7.00042000	0.63463400	0.62112800
C	8.07170200	0.01442500	-0.08993200
H	7.87259500	-0.01391500	-1.16786800
H	8.95192900	0.62932100	0.09932100
H	8.25550600	-1.00409700	0.27227300

L

N	0.21179800	0.37323000	0.92488700
P	-2.38850300	0.26877900	-0.99649000
Ir	-1.00834800	-1.32126700	0.05296600
H	0.02683500	-1.30954200	-1.31684300
C	0.58521500	-2.77134300	0.35670200
C	0.97344400	-2.24061500	-0.95631100
C	2.23456300	1.31188900	1.66547500
H	3.08685000	0.81857500	1.19533400
H	2.57770100	1.99111100	2.44306800
C	0.49567700	1.43931800	0.25450600
O	1.58039900	2.11968500	0.64029400
C	1.13260200	0.32726900	2.09862500
C	-0.23688500	1.99144000	-0.95672800
N	-1.68785800	1.81362100	-0.81223700
H	-2.19365900	2.54652500	-1.29719900
C	-2.57563000	-0.03570500	-2.80201400
H	-3.04383500	-1.00948700	-2.96338500
H	-3.16209400	0.74308400	-3.29460200
H	-1.57585300	-0.06226700	-3.24254900
C	-4.12505800	0.51262800	-0.32545500
C	-4.87831700	-0.82488100	-0.46429800
H	-4.35964200	-1.63490300	0.05750000
H	-5.87588700	-0.72245600	-0.02398300
H	-5.00835900	-1.11798900	-1.51047200
C	-4.03264800	0.91471000	1.15888600
H	-5.04208500	1.09605900	1.54296600
H	-3.58968200	0.12391500	1.77087800
H	-3.44261300	1.82457800	1.29587700
C	-4.86902000	1.61845500	-1.10258700
H	-5.87739000	1.72326800	-0.68825600
H	-4.38154900	2.59384200	-1.00480500
H	-4.97728500	1.38507500	-2.16495500
H	-1.51122100	-1.90317700	1.52342900
H	-1.83757100	-2.48263100	-0.62123300
C	0.11061700	3.47941600	-1.22812100
H	1.20375600	3.52612800	-1.24381400
C	-0.39758000	4.41976100	-0.12506000
H	-0.07555300	5.44449000	-0.33254100
H	-1.49068100	4.42139100	-0.06595500
H	-0.00598500	4.13379400	0.85433300
C	-0.38906200	3.90960600	-2.61574200
H	-0.02892200	4.91683700	-2.84336900
H	-0.02892300	3.23477700	-3.39925700
H	-1.48310300	3.94271300	-2.67639400
H	0.15849900	1.42211400	-1.81169000
C	0.43062300	0.70047500	3.43981400
C	-0.11249300	2.14150300	3.40710700
H	0.68263500	2.88370500	3.28511200
H	-0.83346600	2.27264900	2.59398900
H	-0.62716100	2.36568300	4.34649500
C	1.46818200	0.54826500	4.57085500
H	1.00789800	0.79024700	5.53372100
H	1.84175600	-0.48041100	4.62560200
H	2.32755900	1.21408400	4.44459400
H	1.53481000	-0.68347500	2.19075200
C	-0.73693300	-0.26249500	3.71652300
H	-1.56470700	-0.08147100	3.02949200
H	-0.42646300	-1.30909400	3.62133500
H	-1.11120700	-0.11257700	4.73418900
C	0.20018700	-4.22775700	0.53294300
H	-0.56780400	-4.54974500	-0.17239300
H	1.07705600	-4.87586200	0.39997700
H	-0.18742400	-4.38890100	1.54214800
C	0.80566500	-3.14882400	-2.17755200
H	1.58268800	-3.91896100	-2.13549500
H	-0.17255500	-3.62974100	-2.18842500
H	0.93389200	-2.58592000	-3.10449000
H	1.20503300	-2.40981400	1.17369000
C	2.20413000	-1.36571800	-1.02407300
C	2.30673100	-0.33149000	-1.97084400
C	3.31186800	-1.62162100	-0.21143500
C	3.44654600	0.45033000	-2.06558500
H	1.47535800	-0.13259200	-2.64168200
C	4.48072400	-0.85934900	-0.30830600
H	3.28508900	-2.43737100	0.50320500
C	4.54793800	0.19495100	-1.22849700
H	3.51399000	1.26232100	-2.78165500
H	5.31908400	-1.09688100	0.33447700
O	5.61672400	1.01413700	-1.39477000
C	6.76106100	0.81439900	-0.56387200
H	7.48065100	1.57876000	-0.85748600
H	6.51024200	0.93821200	0.49647600
H	7.20035600	-0.17773000	-0.72040500
H	-2.17614100	-1.26132200	1.23120900

M

N	-1.47979300	1.12130200	-0.47516700
P	-1.13919600	-2.02110000	0.24721300
Ir	0.40166900	-0.26365600	0.07456200
H	0.83585900	0.14139200	-1.51735400
C	1.66927500	0.54008300	1.90839200
C	2.06490300	1.14798300	0.64352000
C	-2.30405800	2.87048100	-1.81634300
H	-1.55975000	3.11227700	-2.58068700
H	-3.10274400	3.60918300	-1.83326700
C	-2.27712100	0.67299900	-1.38857200
O	-2.88935800	1.58174600	-2.16310500
C	-1.68134300	2.60525200	-0.43712600
C	-2.58789300	-0.77358800	-1.73445500
N	-2.60049900	-1.59659700	-0.51908600
H	-3.25038800	-2.37012600	-0.60346700
C	-0.54611800	-3.56922900	-0.54680700
H	0.38931300	-3.88628500	-0.08053700
H	-1.28423400	-4.37182800	-0.47800400
H	-0.34769300	-3.35209200	-1.59939600
C	-1.67988000	-2.50790600	1.97683500
C	-0.45767700	-3.08369700	2.71882100
H	0.38520600	-2.38502600	2.71475700
H	-0.72860700	-3.27517600	3.76254200
H	-0.12119400	-4.03058600	2.28651700
C	-2.19109900	-1.24977700	2.70179100
H	-2.58281000	-1.53268700	3.68470300
H	-1.38912100	-0.52437000	2.86169600
H	-2.99083600	-0.75907600	2.14040100
C	-2.80816800	-3.55848900	1.92281200
H	-3.07399500	-3.84518000	2.94586000
H	-3.71417500	-3.16122300	1.45466800
H	-2.50937900	-4.47015100	1.39745800
H	0.44760300	-0.14406600	1.75426400
H	1.54337300	-1.32916500	0.15754000
C	-3.92363400	-0.93183600	-2.51201100
H	-3.85814400	-0.23827500	-3.35549600
C	-5.15261200	-0.55576600	-1.67186500
H	-6.05944500	-0.64989700	-2.27651200
H	-5.26337900	-1.21254500	-0.80324000
H	-5.09095800	0.47386400	-1.31268800
C	-4.04900900	-2.35071400	-3.08976100
H	-4.92186600	-2.40666500	-3.74633000
H	-3.16565500	-2.62745400	-3.67469600
H	-4.18951800	-3.10987900	-2.31133000
H	-1.79183000	-1.08200000	-2.43317800
C	-2.57546200	3.04857000	0.76389100
C	-3.96522700	2.38680500	0.71024200
H	-4.51080500	2.64089000	-0.20394200
H	-3.88886000	1.29731800	0.77403400
H	-4.56922800	2.72708300	1.55690900
C	-2.72530700	4.58276100	0.72324700
H	-3.26697100	4.92509900	1.61068400
H	-1.74715100	5.07698700	0.71673200
H	-3.28531200	4.92743500	-0.15144100
H	-0.71247800	3.09452000	-0.35566500
C	-1.88813600	2.66269900	2.08222400
H	-1.64273200	1.59943600	2.10629200
H	-0.96947000	3.24074800	2.21967500
H	-2.54135100	2.88380200	2.93222900
H	0.31788000	-0.60916200	-1.59335700
C	1.79330900	2.63274000	0.44747300
H	0.83896200	2.93725300	0.87389800
H	2.58166500	3.22168500	0.93431500
H	1.79368300	2.89482700	-0.61272600
H	2.25981800	-0.32980800	2.18716900
C	3.37306800	0.70797600	0.03774900
C	3.60152900	0.82391000	-1.34675900
C	4.43722000	0.25301500	0.82455300
C	4.81561100	0.47745900	-1.91675400
H	2.80091700	1.17580600	-1.99172700
C	5.66959600	-0.11032300	0.26836600
H	4.33087900	0.19283800	1.90344300
C	5.86438000	-0.00193600	-1.11167400
H	4.97767400	0.55907800	-2.98672800
H	6.46118500	-0.45749300	0.92109400
O	7.01409400	-0.32302200	-1.76752500
C	8.10908500	-0.82199600	-1.00250200
H	8.91022300	-1.01780500	-1.71599300
H	8.45225900	-0.08586500	-0.26526400
H	7.84679400	-1.75375400	-0.48653100
C	1.26531900	1.35172300	3.12739100
H	0.59365700	2.17178500	2.89295200
H	0.79876200	0.72161800	3.88855400
H	2.18465200	1.77278200	3.55044900

N

N	0.15572100	0.94908500	0.04236800
P	-2.74840000	-0.40873500	-0.31952100
Ir	-0.54751700	-1.17939900	-0.62593100
H	0.07296300	-0.87859500	-2.15133500
C	0.62661900	-2.57153100	0.86948200
C	1.32886900	-2.34846700	-0.40100400
C	1.68085400	2.71646500	-0.16854600
H	2.29358200	2.52689800	-1.05486800
H	2.07944600	3.56374800	0.38537400
C	-0.39826600	1.96919000	-0.52537200
O	0.34191700	3.08218100	-0.62447000
C	1.43222000	1.44653400	0.65395200
C	-1.79671300	2.05457800	-1.11625800
N	-2.74227900	1.29582200	-0.28352000
H	-3.66997400	1.70374500	-0.31157800
C	-3.86048000	-0.91115100	-1.69577500
H	-3.90061900	-2.00084700	-1.75913200
H	-4.87015200	-0.51153600	-1.57581400
H	-3.43438000	-0.52965100	-2.62703500
C	-3.64055900	-0.89305300	1.25654500
C	-3.82458000	-2.42355800	1.24055500
H	-2.86929100	-2.94137000	1.10432900
H	-4.24719000	-2.74362800	2.19876200
H	-4.50958200	-2.74947800	0.45256000
C	-2.77545700	-0.48621900	2.46169500
H	-3.31895700	-0.70626800	3.38676200
H	-1.83920800	-1.04896900	2.48900800
H	-2.53740800	0.58072200	2.44874800
C	-5.01039100	-0.19114800	1.35057400
H	-5.51734400	-0.52548400	2.26208200
H	-4.90798800	0.89655800	1.41909100
H	-5.66428900	-0.43194600	0.50792100
H	-0.50232600	-1.85224600	0.94412700
H	-1.14367200	-2.52131700	-1.17892600
C	-2.28257100	3.51825500	-1.29633300
H	-1.48771800	4.02579600	-1.85118300
C	-2.48382800	4.25420200	0.03690700
H	-2.78419300	5.28886100	-0.15320100
H	-3.26773800	3.78842200	0.64265700
H	-1.56530600	4.27225300	0.62749000
C	-3.55257300	3.56516900	-2.16085000
H	-3.81075200	4.60431900	-2.38362000
H	-3.41262900	3.03909500	-3.11077200
H	-4.41975100	3.12429900	-1.65522400
H	-1.71396300	1.63154600	-2.13139700

C	1.31094900	1.68735300	2.19089200
C	0.21624000	2.71580700	2.52717600
H	0.41919400	3.69450700	2.08092500
H	-0.76588300	2.37838200	2.18210300
H	0.15690900	2.85625100	3.61091300
C	2.67802500	2.18512100	2.70442400
H	2.65538600	2.27025000	3.79550400
H	3.47715800	1.48587900	2.43526100
H	2.94089400	3.17055200	2.30783400
H	2.21702500	0.71733300	0.48295400
C	0.98771500	0.35872900	2.89245600
H	0.05593900	-0.06449600	2.51562000
H	1.79540600	-0.36396400	2.74178600
H	0.87749400	0.51365600	3.97044800
H	-0.80167400	-0.50680000	-2.14530200
C	0.08627500	-3.94255400	1.24612500
H	-0.51033200	-3.88914600	2.16000200
H	-0.52805500	-4.37149300	0.45430900
H	0.93523100	-4.61013000	1.42692800
C	1.44187800	-3.51614000	-1.38000400
H	2.21263700	-4.21544400	-1.02929500
H	0.50641100	-4.06408100	-1.49862000
H	1.74794200	-3.15432600	-2.36390800
H	1.07032400	-2.04892200	1.71408800
C	2.58789200	-1.52562400	-0.35888200
C	2.96661900	-0.72882700	-1.44818600
C	3.45885100	-1.56133300	0.74521700
C	4.12084000	0.05339200	-1.43040400
H	2.31952000	-0.68066800	-2.31906900
C	4.61090800	-0.78224900	0.78733000
H	3.23960400	-2.20584400	1.59100100
C	4.94510800	0.04574600	-0.29422900
H	4.35590600	0.67032600	-2.28892000
H	5.26858800	-0.80261700	1.65003700
O	6.07397200	0.79036200	-0.14873500
C	6.44482700	1.67038000	-1.20859000
H	5.66644200	2.42037500	-1.39600400
H	7.35566600	2.17047400	-0.87838200
H	6.64869600	1.12069400	-2.13525000

O

N	-0.14430700	0.86072700	0.22980000
P	2.71838200	-0.50671900	-0.32726600
Ir	0.51226900	-1.28655600	-0.19177500
H	0.80714700	-1.40290500	1.45438500
C	-1.28842800	-2.27445100	0.73714600
C	-0.41805400	-1.96218600	1.86249100
C	-1.53946400	2.48341800	1.17981000
H	-2.10340700	2.02014100	1.99427700
H	-1.91747600	3.48544000	0.98789600
C	0.50794000	1.58558200	1.07588100
O	-0.15232600	2.61419200	1.62276000
C	-1.43329200	1.56801400	-0.04788800
C	1.94007900	1.37476600	1.53838100
N	2.79358200	1.04300300	0.38533400
H	3.74301900	1.37036600	0.52876000
C	3.84792900	-1.59449700	0.63611400
H	3.80582500	-2.60942100	0.23498400
H	4.88011800	-1.23648400	0.62968700
H	3.48173000	-1.61962200	1.66554100
C	3.54249700	-0.26919500	-1.99549500
C	3.70576500	-1.65777000	-2.64403300
H	2.74959100	-2.18774800	-2.70648500
H	4.08878900	-1.53414400	-3.66249200
H	4.41519600	-2.28702100	-2.09878000
C	2.64626100	0.61946700	-2.87524700
H	3.17123900	0.84646500	-3.80923400
H	1.71343000	0.11629800	-3.14165700
H	2.40377700	1.56388200	-2.38060000
C	4.91845800	0.40813600	-1.83235300
H	5.39557900	0.47976300	-2.81548900
H	4.82519200	1.42739100	-1.44492100
H	5.59422300	-0.15665100	-1.18425700
H	-0.33724500	-1.48204500	-1.64616900
H	1.05505800	-2.75013000	-0.40309600
C	2.51176100	2.59500900	2.30620800
H	1.76688000	2.83765900	3.07048200
C	2.70355000	3.82997100	1.41234600
H	3.03851400	4.67699900	2.01829900
H	3.45964600	3.65832700	0.63926100
H	1.77212000	4.11449700	0.91732600
C	3.81315900	2.21620300	3.03015600
H	4.14940300	3.05069200	3.65189400
H	3.67444100	1.34415400	3.67763200
H	4.62762100	1.99041700	2.33205600
H	1.89306600	0.54274800	2.25978400
C	-1.44087400	2.30758700	-1.41869500
C	-0.35671600	3.39844700	-1.48505000
H	-0.51206900	4.18307400	-0.73767000
H	0.64072700	2.97430800	-1.33198600
H	-0.36627900	3.87689400	-2.46945500
C	-2.83892200	2.93291200	-1.60345800
H	-2.90271400	3.42262700	-2.58025000
H	-3.61853400	2.16484800	-1.55703800
H	-3.06415100	3.68864200	-0.84445500
H	-2.23731500	0.84113000	-0.05206900
C	-1.20758700	1.29302600	-2.55087600
H	-0.19866200	0.88508600	-2.50235700
H	-1.92156900	0.46505800	-2.49421800
H	-1.32691800	1.78044000	-3.52382400
H	0.47873200	-1.12992700	-1.87627900
H	-0.70517900	-1.07100500	2.42098100
C	0.19118600	-3.05012700	2.73248200
H	0.68813200	-3.81586600	2.13679600
H	0.91562800	-2.63173600	3.43553000
H	-0.61376700	-3.52422700	3.30443300
C	-1.44144500	-3.73887900	0.33126500
H	-0.49521900	-4.27994700	0.34636300
H	-2.13907000	-4.23761000	1.01715600
H	-1.85455400	-3.82058600	-0.67558500
C	-2.56412900	-1.49340400	0.58915800
C	-3.19496400	-1.39787700	-0.66040100
C	-3.19903300	-0.86582800	1.67604500
C	-4.36981700	-0.67222100	-0.84846400
H	-2.73361300	-1.86578000	-1.52477800
C	-4.36471100	-0.12422200	1.50734900
H	-2.78802100	-0.95443900	2.67646000
C	-4.95504800	-0.00786700	0.24089600
H	-4.80186600	-0.61218100	-1.83959100
H	-4.83980500	0.36804500	2.34954100
O	-6.07695000	0.75801900	0.17228700
C	-6.71629200	0.91251500	-1.09439100
H	-7.57954500	1.55453900	-0.91731400
H	-6.05170800	1.39279100	-1.82302200
H	-7.05614200	-0.05066500	-1.49332200

P

N	-1.34466400	1.23241600	-0.03542900
P	-1.22409200	-1.98470900	0.10510000
Ir	0.37222000	-0.27631400	-0.06391200
H	0.19971700	-0.52837100	-1.70816300
C	1.90400500	1.02911600	-1.08100700
C	1.21589300	0.36602400	-2.17666100
C	-2.39857500	3.26773600	-0.51937000
H	-1.89377500	3.73726300	-1.36867600
H	-3.12128300	3.96035100	-0.09374700
C	-2.39877000	1.04094200	-0.75552600
O	-3.14240400	2.11921400	-1.03241400
C	-1.42237700	2.63383700	0.48606300
C	-2.86752800	-0.28511100	-1.33038800
N	-2.74943700	-1.33365100	-0.30213000
H	-3.46936800	-2.03878500	-0.41348000
C	-0.87097900	-3.32512700	-1.10654500
H	0.13354400	-3.71715000	-0.93311500
H	-1.59854000	-4.13845800	-1.05370000
H	-0.89837500	-2.88460700	-2.10642800
C	-1.53644800	-2.85839100	1.73537200
C	-0.28934200	-3.70312500	2.06334000
H	0.62166100	-3.09522900	2.06195800
H	-0.40351100	-4.13482900	3.06330200
H	-0.15502000	-4.52985500	1.36001100
C	-1.76198900	-1.80833900	2.83602000
H	-2.05398000	-2.31365700	3.76267100
H	-0.85001200	-1.24430800	3.04726100
H	-2.55439600	-1.10452700	2.56753000
C	-2.78320700	-3.76217400	1.63740300
H	-2.89419900	-4.31273000	2.57758600
H	-3.69736700	-3.17699900	1.49679500
H	-2.71021000	-4.50248200	0.83586000
H	0.99510800	0.38180100	1.36596800
H	1.50940300	-1.36433900	-0.08131200
C	-4.31294300	-0.22353900	-1.88862500
H	-4.33623800	0.64938100	-2.54884400
C	-5.37167600	-0.02577100	-0.79253000
H	-6.35932000	0.08839500	-1.24872800
H	-5.41895000	-0.88452100	-0.11551900
H	-5.16805400	0.86578800	-0.19512900
C	-4.61439000	-1.46249600	-2.74636000
H	-5.59251200	-1.35512700	-3.22374000
H	-3.86563400	-1.59874600	-3.53375500
H	-4.64919600	-2.38262000	-2.15153700
H	-2.20570100	-0.48022200	-2.18988800
C	-1.88871900	2.71719000	1.97205800
C	-3.29403900	2.11685400	2.16091400
H	-4.05834600	2.66784500	1.60370800
H	-3.32449600	1.07101600	1.84000700
H	-3.57339000	2.14814000	3.21847900
C	-1.88921500	4.20474800	2.38118000
H	-2.18231400	4.29957900	3.43126400
H	-0.89232000	4.64581200	2.26969300
H	-2.59191100	4.80291000	1.79307500
H	-0.43726800	3.09235000	0.42255400
C	-0.89344700	1.96853500	2.87353800
H	-0.95291000	0.89381800	2.71029700
H	0.13675800	2.29397500	2.68880500
H	-1.12361000	2.15852500	3.92662300
H	0.46802400	-0.32706100	1.62214000
C	0.35886900	1.12843000	-3.17164000
H	-0.37430200	1.76525000	-2.67679300
H	1.01134100	1.76692700	-3.77667700
H	-0.16974500	0.44485400	-3.84020300
H	1.75988600	-0.46619100	-2.62044500
C	1.73845300	2.53668100	-0.93460500
H	2.44571800	3.04395000	-1.60351300
H	0.73829300	2.87963100	-1.19383000
H	1.96328400	2.85740300	0.08491100
C	3.30712100	0.58007400	-0.75781200
C	3.81804900	0.68670300	0.54221000
C	4.18509700	0.13177500	-1.75944300
C	5.13168500	0.34096900	0.85484100
H	3.16633400	1.02941700	1.34103100
C	5.49730400	-0.22727300	-1.46743200
H	3.85317200	0.07915500	-2.79216900
C	5.98271900	-0.12951600	-0.15653700
H	5.47415200	0.43044900	1.87852500
H	6.16833400	-0.57427100	-2.24655700
O	7.27883800	-0.50639100	0.03000200
C	7.82714700	-0.41416100	1.34292700
H	7.28693000	-1.05528400	2.05039800
H	8.85922400	-0.75715500	1.26234700
H	7.81685600	0.61889500	1.71179900

Ir/4c with S3

TSs for the σ-methatesis pathway

A

N	-1.06726100	1.18431300	-0.45375100
P	-0.97973200	-1.94058700	0.38378100
Ir	0.63591200	-0.28828400	0.25663800
H	1.61228200	0.11825300	-1.04494400
C	2.26100300	1.07724500	1.11152500
C	2.57403600	1.01080900	-0.30674400
C	-1.93697800	2.99290700	-1.66936900
H	-1.18031600	3.30673700	-2.39436100
H	-2.75729200	3.70698800	-1.65897400
C	-1.84683800	0.77515500	-1.40007500
O	-2.47521400	1.71395000	-2.11934200
C	-1.34285700	2.65075900	-0.29426200
C	-2.14104000	-0.66636900	-1.78326400
N	-2.31040600	-1.46966500	-0.56012900
H	-2.99444500	-2.20702900	-0.68529700
C	-0.31497200	-3.50430500	-0.31603000
H	0.57350300	-3.80885400	0.24121700
H	-1.05707100	-4.30542600	-0.29633200
H	-0.02364600	-3.30522500	-1.34941900
C	-1.71153200	-2.39196900	2.04725100
C	-0.57567500	-2.94828300	2.92896300
H	0.24325400	-2.22947500	3.02789400
H	-0.97220900	-3.15027100	3.92938100
H	-0.17110800	-3.88736300	2.54018900
C	-2.32465800	-1.14413900	2.70263700
H	-2.78946700	-1.43524100	3.65061800
H	-1.56573100	-0.39139100	2.91970900
H	-3.09387100	-0.69271400	2.07111000
C	-2.81515400	-3.45668200	1.86339700
H	-3.19821300	-3.73161500	2.85152700
H	-3.66274500	-3.07262500	1.28722100
H	-2.45138000	-4.37259900	1.39100600
H	0.26018800	-0.11728000	1.82629500
H	1.65403300	-1.40404000	0.65405900
C	-3.37745200	-0.81283000	-2.70812400
H	-3.21702100	-0.10283500	-3.52540800
C	-4.70218500	-0.45619700	-2.01661300
H	-5.52041700	-0.50481000	-2.74109400
H	-4.94039900	-1.15394400	-1.20725300
H	-4.67853300	0.55210400	-1.59844200
C	-3.42566000	-2.22220800	-3.31879800
H	-4.24085700	-2.28507700	-4.04511400
H	-2.49163800	-2.47003500	-3.83360300
H	-3.60927300	-2.99619100	-2.56440700
H	-1.27007400	-0.99929100	-2.36833300
C	-2.29280300	2.95899000	0.90912800
C	-3.63794300	2.22311000	0.76302000
H	-4.17549700	2.52207800	-0.14233900
H	-3.49977900	1.13875500	0.73712300
H	-4.28059500	2.45644600	1.61728000
C	-2.53800800	4.48146200	0.95834500
H	-3.10714200	4.73183800	1.85907800
H	-1.59208500	5.03342100	0.99648600
H	-3.11142700	4.84688100	0.10099900
H	-0.40737700	3.18799100	-0.13890700
C	-1.62039600	2.53948900	2.22815700
H	-1.24526700	1.51798700	2.17640500
H	-0.78356900	3.19941400	2.46972700
H	-2.33857000	2.60424800	3.05177400
C	3.13541000	0.31072700	2.09489400
H	3.48135000	-0.64437000	1.70226200
H	4.01819300	0.91973300	2.33011900
H	2.58799000	0.13143600	3.02173600
C	1.78553400	2.39029700	1.69633400
H	1.05316000	2.90592600	1.08318200
H	1.35517900	2.23371500	2.68558600
H	2.65510500	3.05280700	1.80486300
H	0.59704500	-0.86649700	-1.29322400
C	3.83872900	0.26540200	-0.70578800
C	3.87459300	-1.01694500	-1.26125800
C	5.04651700	0.95557200	-0.51161100
C	5.09434900	-1.60176600	-1.61302800
H	2.95323300	-1.56845600	-1.40886100
C	6.26249100	0.37093100	-0.86228100
H	5.03532600	1.95133500	-0.07763300
C	6.29067800	-0.91225800	-1.41508900
H	5.10390700	-2.59981000	-2.04091600
H	7.18682700	0.91736800	-0.70076000
H	7.23718300	-1.36917200	-1.68738900
C	2.31064100	2.23235800	-1.19395900
H	3.00874400	3.03070600	-0.92414900
H	2.47047600	1.97797300	-2.24362000
H	1.29718900	2.61245700	-1.08616100

B

N	0.06989700	1.10540700	0.19041200
P	-2.25456900	-1.05004700	-0.38426800
Ir	0.05040100	-1.20193600	-0.25116800
H	1.22395800	-1.04403900	-1.42471700
C	1.98814800	-2.02772900	0.67439300
C	2.39912500	-1.58952300	-0.64831600
C	1.03815900	3.24140600	0.04800400
H	1.86658600	3.20875500	-0.66189500
H	1.02731500	4.20008400	0.56158500
C	-0.60510200	1.86774200	-0.60423100
O	-0.20346500	3.14050200	-0.71420600
C	0.98777100	2.00565100	0.96112500
C	-1.81228200	1.47831900	-1.43999100
N	-2.67447100	0.56999000	-0.66611600
H	-3.65329900	0.69708000	-0.89631900
C	-2.86062400	-2.05527400	-1.79663200
H	-2.56253900	-3.09650300	-1.65809900
H	-3.94537100	-1.99249400	-1.90453200
H	-2.38201800	-1.67616500	-2.70168400
C	-3.29304200	-1.55290800	1.09189000
C	-3.08259200	-3.06043100	1.33545700
H	-2.02410000	-3.29954200	1.47566200
H	-3.61804400	-3.34867300	2.24604000
H	-3.47409500	-3.67044400	0.51621000
C	-2.85462100	-0.75281800	2.32930700
H	-3.52747400	-0.98955100	3.16027400
H	-1.84033900	-1.01420300	2.63473100
H	-2.89773700	0.32450500	2.15030700
C	-4.78283000	-1.26387800	0.80471200
H	-5.37290900	-1.60492600	1.66152500
H	-4.97819700	-0.19340900	0.68665100
H	-5.15635000	-1.79212100	-0.07629100
H	-0.30943900	-1.59436400	1.28310000
H	-0.12851500	-2.70110300	-0.65675200
C	-2.62518500	2.70320500	-1.93679900
H	-1.89485500	3.36398900	-2.41390900
C	-3.30499000	3.48017200	-0.79927700
H	-3.81780000	4.35706800	-1.20521500
H	-4.05314400	2.87124300	-0.28145000
H	-2.57836400	3.82373400	-0.06028600
C	-3.63823900	2.27980400	-3.01247400
H	-4.11035200	3.16582500	-3.44620500
H	-3.15661000	1.72110300	-3.82162800
H	-4.44474000	1.65733500	-2.60665200
H	-1.40454900	0.98486200	-2.33583400
C	0.45781100	2.31758000	2.39996600
C	-0.92906000	2.98747800	2.34706800
H	-0.91499700	3.93314200	1.79650600
H	-1.67032100	2.33157300	1.88155800
H	-1.27150300	3.20691200	3.36271600
C	1.46387000	3.25987500	3.09310200
H	1.16873300	3.40948600	4.13651500
H	2.47305000	2.83254700	3.08886000
H	1.51121200	4.24809100	2.62531000
H	1.97202600	1.54093100	1.02477800
C	0.35755900	1.02682300	3.23062400
H	-0.16845500	0.24344600	2.68560300
H	1.34801200	0.65456200	3.50019400
H	-0.18342300	1.22352000	4.16183400
H	-0.18692200	-0.77369700	-1.82970000
C	1.86973000	-3.51713500	0.96490100
H	1.41034400	-4.09080000	0.16252100
H	2.87789600	-3.91739400	1.13874100
H	1.28296800	-3.67468300	1.87168300
C	2.69750900	-2.64890800	-1.71841700
H	3.65189200	-3.12399300	-1.46780000
H	1.92523500	-3.41493100	-1.77207800
H	2.79654700	-2.18222000	-2.70069800
C	3.30761400	-0.38335000	-0.79589900
C	2.96917000	0.73420500	-1.56794800
C	4.59901900	-0.46332000	-0.25229300
C	3.89234800	1.76192500	-1.77399800
H	1.97805600	0.80765100	-2.00432100
C	5.51801100	0.56709600	-0.44987600
H	4.88941400	-1.33845800	0.32029800
C	5.16626300	1.68589200	-1.20867800
H	3.61423900	2.61962800	-2.37942900
H	6.50990100	0.49160900	-0.01492400
C	2.46315600	-1.27063500	1.89863100
H	3.45601400	-1.64150100	2.18259200
H	2.54792100	-0.20050500	1.73467500
H	1.79051600	-1.44977600	2.73625300
H	5.88180100	2.48717500	-1.36489200

C

N	0.03960200	1.06504800	-0.24047900
P	-2.30796000	-1.05523400	0.29206600
Ir	-0.01412000	-1.21971600	0.00507600
H	-0.72908400	-1.31046000	-1.45667100
C	2.33822900	-1.72401000	-0.43935700
C	1.52709400	-1.60163400	-1.64000000
C	0.95810900	3.02850500	-1.14118100
H	1.60152800	2.78325500	-1.99001400
H	1.06375300	4.08313100	-0.89688500
C	-0.79138500	1.63288100	-1.04612800
O	-0.42459500	2.81332300	-1.56227700
C	1.11837200	2.06224000	0.04733000
C	-2.13584400	1.08672500	-1.49119600
N	-2.80628600	0.42794200	-0.36048000
H	-3.81222100	0.54232800	-0.39375700
C	-3.16081200	-2.41308100	-0.60466400
H	-2.76804600	-3.37333100	-0.26366500
H	-4.24189200	-2.38179600	-0.45586800
H	-2.93645900	-2.30556900	-1.66754500
C	-3.03856600	-1.06275200	2.02018700
C	-2.77117300	-2.44472000	2.64764400
H	-1.70197900	-2.67716000	2.66308200
H	-3.12982200	-2.43856700	3.68210400
H	-3.29600200	-3.24710700	2.12108000
C	-2.38303300	0.03727600	2.86977400
H	-2.88263900	0.07837100	3.84341600
H	-1.32685800	-0.17081500	3.04738900
H	-2.47121800	1.02010500	2.39928100
C	-4.55875400	-0.79815800	1.94374800
H	-4.97013200	-0.84927200	2.95710200
H	-4.78194100	0.20171300	1.55859300
H	-5.09191700	-1.53729800	1.34064900
H	1.43434300	-1.37857000	0.80207500
H	-0.16115700	-2.77673500	0.13451400
C	-3.05198600	2.17832100	-2.10403800
H	-2.44361200	2.67333100	-2.86735300
C	-3.49154600	3.23687900	-1.08090000
H	-4.07468200	4.01574700	-1.58090700
H	-4.12232500	2.80729000	-0.29547700
H	-2.63189200	3.71168300	-0.60222900
C	-4.25646900	1.54102000	-2.81331000
H	-4.83026600	2.31138900	-3.33604000
H	-3.93955100	0.79495300	-3.54934100
H	-4.94373300	1.05177300	-2.11337900
H	-1.90532400	0.37123800	-2.29666200
C	0.95604900	2.75295100	1.43968000
C	-0.37890300	3.51602400	1.53461400
H	-0.46207800	4.30952400	0.78526600
H	-1.23098200	2.84022500	1.41310000
H	-0.46691500	3.98605700	2.51880800
C	2.13792100	3.72677000	1.62661700
H	2.09767700	4.16821000	2.62743200
H	3.09558700	3.20361700	1.52715100
H	2.12372600	4.55170500	0.90743000
H	2.08437800	1.55739300	0.02036100
C	1.01211300	1.70613000	2.56376300
H	0.26219300	0.93124700	2.41815100
H	1.99239000	1.22867700	2.60777600

H	0.83091100	2.18663400	3.53080000
H	-0.01533700	-1.12528200	1.63293700
C	3.41672300	-0.71596400	-0.09491100
C	3.62299600	-0.31495500	1.23275200
C	4.33294900	-0.29627900	-1.06995500
C	4.67152100	0.53847200	1.57078600
H	2.94392400	-0.66335200	2.00501600
C	5.38807000	0.55483200	-0.73330300
H	4.23798400	-0.64119300	-2.09246200
C	5.55253400	0.98742000	0.58315400
H	4.79772100	0.85234900	2.60230000
H	6.08167000	0.87564900	-1.50439800
C	2.73045100	-3.12314300	0.04065300
H	3.54566200	-3.47887600	-0.59952500
H	3.09987900	-3.08679300	1.06708100
H	1.90671700	-3.83264500	-0.00783000
C	1.22433800	-2.86323700	-2.44086700
H	2.14513700	-3.19165100	-2.94125900
H	0.84997700	-3.68727700	-1.83646900
H	0.47956700	-2.64598300	-3.20891400
H	6.36708200	1.65695400	0.84122300
C	1.65319800	-0.38511900	-2.54855800
H	1.96177600	0.51193800	-2.02000600
H	2.39684300	-0.58971400	-3.32823500
H	0.70467100	-0.18826500	-3.05016500

D

N	-1.11494300	1.26915400	-0.14274700
P	-1.04758100	-1.97052700	0.05147200
Ir	0.56519600	-0.32968500	-0.20112100
H	0.03577100	-0.66916600	-1.70631200
C	2.56051600	1.02640000	-0.44783900
C	1.91828500	0.71803700	-1.70899100
C	-2.22836000	3.29947200	-0.56884000
H	-1.81791000	3.79232700	-1.45435000
H	-2.92461900	3.96822900	-0.06721700
C	-2.21390200	1.07053600	-0.78950800
O	-2.98473200	2.14061800	-1.02177500
C	-1.14097800	2.68726100	0.33526100
C	-2.72157400	-0.25087600	-1.34224800
N	-2.55793800	-1.30659900	-0.33028000
H	-3.31373500	-1.98102000	-0.35282600
C	-0.71729000	-3.37006800	-1.08845200
H	0.24836000	-3.81920300	-0.84557700
H	-1.50171000	-4.12790900	-1.03670200
H	-0.66496900	-2.96723500	-2.10145100
C	-1.29399400	-2.72516600	1.75145700
C	0.04712700	-3.31871200	2.22474400
H	0.82547500	-2.55378000	2.29128000
H	-0.09031200	-3.75099100	3.22135200
H	0.40164800	-4.11716200	1.56583600
C	-1.76731800	-1.63159000	2.72359400
H	-1.88044200	-2.06760300	3.72164500
H	-1.04873500	-0.81293300	2.79575800
H	-2.73120400	-1.21703500	2.41880300
C	-2.36150200	-3.83959000	1.68461400
H	-2.52110100	-4.22816300	2.69577200
H	-3.32920100	-3.47205400	1.32771600
H	-2.05128900	-4.67994500	1.05884700
H	1.63982000	0.49640500	0.76252800
H	1.59574000	-1.50569200	-0.29028500
C	-4.19348300	-0.18483400	-1.82453300
H	-4.24771600	0.68932000	-2.48096400
C	-5.19803400	0.01352500	-0.67825500
H	-6.20423200	0.14306000	-1.08745900
H	-5.22760600	-0.85179000	-0.00785700
H	-4.95750600	0.89632300	-0.08164900
C	-4.54021300	-1.42111600	-2.66875700
H	-5.54547400	-1.31611500	-3.08651400
H	-3.83855600	-1.54915500	-3.49945000
H	-4.53437900	-2.34438600	-2.07789600
H	-2.10032300	-0.44703500	-2.22962500
C	-1.41234100	2.82971500	1.86385800
C	-2.75397500	2.18182100	2.24963200
H	-3.60134100	2.65779700	1.74537700
H	-2.76228700	1.11719800	2.00150500
H	-2.91875200	2.27565800	3.32739300
C	-1.43021600	4.33403000	2.20799300
H	-1.55681200	4.46272200	3.28734100
H	-0.48863100	4.81613700	1.92132400
H	-2.24911900	4.87121200	1.72008400
H	-0.17841700	3.15185100	0.12533600
C	-0.27405900	2.17395200	2.66382100
H	-0.23015700	1.10106400	2.48693200
H	0.69877500	2.59943000	2.39489400
H	-0.42720200	2.33870700	3.73513500
H	0.62180800	-0.45717900	1.42497000
C	3.84984200	0.30844900	-0.08756400
C	3.98227200	-0.62210800	0.94833600
C	4.99522900	0.68649100	-0.80733100
C	5.22917300	-1.17732500	1.24713700
H	3.11027800	-0.92568700	1.51548200
C	6.23875300	0.13014200	-0.51067100
H	4.91250400	1.41536800	-1.60858600
C	6.35988800	-0.80720600	0.51828000
H	5.31131200	-1.90179700	2.05192600
H	7.11084000	0.42876700	-1.08459300
C	2.57561700	2.46463000	0.06742800
H	3.32955800	3.01513600	-0.50553200
H	1.62622400	2.97751300	-0.05521000
H	2.86112800	2.49881300	1.12030500
C	1.20137900	1.82238800	-2.46847200
H	0.56388800	2.44125700	-1.84187000
H	1.94839700	2.47426800	-2.94057900
H	0.57928600	1.39321100	-3.25597300
H	7.32716000	-1.24273200	0.74971200
C	2.57777400	-0.28959200	-2.64352500
H	3.37176200	0.21714300	-3.20700400
H	3.02449700	-1.12813600	-2.11137100
H	1.84946300	-0.67618300	-3.35779800

E

N	-0.78939000	1.30680900	-0.56803300
P	-1.51309700	-1.69564600	0.39276000
Ir	0.13386900	-0.86095400	-1.09162500
H	0.06804900	-0.36741000	-2.61574500
C	2.62864500	-1.01689900	-0.62333300
C	2.27962600	-0.46908900	-1.91289100
C	-1.36803200	3.45452400	-1.34634800
H	-1.28116800	3.48610600	-2.43609200
H	-1.54359600	4.45760800	-0.96186400
C	-2.07015200	1.43587500	-0.67898100
O	-2.53735600	2.64631100	-1.02126300
C	-0.21717900	2.68570200	-0.67643300
C	-3.12942100	0.36251800	-0.45241700
N	-2.75598300	-0.54983800	0.63087800
H	-2.66820100	-0.06100500	1.51459100
C	-2.36278100	-3.12874300	-0.36074500
H	-1.63847600	-3.93014300	-0.52413700
H	-3.18432300	-3.47658900	0.26727900
H	-2.75446300	-2.82020200	-1.33227900
C	-1.06852000	-2.18961500	2.14785400
C	0.02294900	-3.27338400	2.07273500
H	0.91534900	-2.92064600	1.55115100
H	0.31420300	-3.55770800	3.08889300
H	-0.33140200	-4.17526800	1.56485400
C	-0.54184700	-0.95045400	2.89779100
H	-0.12422400	-1.26482600	3.85969100
H	0.24171400	-0.43395500	2.34168000
H	-1.33183800	-0.22499900	3.11741700
C	-2.30704700	-2.74881000	2.88091100
H	-2.03633700	-2.93988000	3.92468900
H	-3.14552400	-2.04705800	2.87382300
H	-2.64528200	-3.69553600	2.45244200
H	1.13682100	-1.11290200	0.22978800
H	0.41442100	-2.31666300	-1.57131100
C	-4.54952900	0.94138700	-0.21618300
H	-4.72925300	1.64649700	-1.03410200
C	-4.66554400	1.70667300	1.11057900
H	-5.65103600	2.17545100	1.18472200
H	-4.56633700	1.03391800	1.97006400
H	-3.91334300	2.49583500	1.19506600
C	-5.59305600	-0.17926700	-0.30391300
H	-6.59979500	0.23212000	-0.18450600
H	-5.54956400	-0.69071900	-1.27161800
H	-5.43244200	-0.92398000	0.48122300
H	-3.17660200	-0.21808700	-1.38155600
C	0.21574100	3.25287300	0.71101000
C	-0.97776900	3.34436000	1.68019000
H	-1.77993500	3.98270800	1.29738200
H	-1.39531500	2.35427400	1.88690600
H	-0.64938500	3.76663800	2.63478000
C	0.82430900	4.65311700	0.49833300
H	1.21648800	5.03429000	1.44659900
H	1.65302600	4.61991800	-0.21779000
H	0.09031600	5.37902300	0.13448300
H	0.65248200	2.66120000	-1.32894800
C	1.28335400	2.33565100	1.32174900
H	0.93030200	1.30583800	1.37139700
H	2.20796100	2.34199400	0.73884300
H	1.53535400	2.66085000	2.33585500
C	3.00638000	-2.49095700	-0.52128900
H	2.37737900	-3.14133500	-1.12527700
H	4.04455500	-2.58482000	-0.86218100
H	2.97290300	-2.83152500	0.51442300
H	-1.21623400	-0.96566600	-1.93590200
C	2.47785300	1.01033100	-2.21241200
H	3.47127900	1.16852500	-2.64977200
H	1.74749100	1.34478900	-2.95123400
H	2.40076600	1.63662400	-1.32692000
C	3.33817600	-0.21629500	0.44303000
C	4.43284600	0.58999000	0.09779900
C	3.05805500	-0.40743700	1.80433700
C	5.18421500	1.23307100	1.08446400
H	4.71997300	0.70642300	-0.93962700
C	3.79947200	0.23824400	2.78969400
H	2.24452900	-1.06056000	2.09795000
C	4.86236000	1.07227900	2.43184900
H	6.02360000	1.85697800	0.79318900
H	3.54736100	0.08957400	3.83509900
H	5.44050800	1.58046600	3.19737400
C	2.53962900	-1.31918500	-3.15423800
H	2.18458400	-2.34450200	-3.07198100
H	2.05907300	-0.86713800	-4.02433400
H	3.62221700	-1.34442400	-3.33940400

F

N	-1.24529300	1.23305500	-0.14536800
P	-0.84926000	-1.97465800	-0.05480100
Ir	0.57628000	-0.21343400	-0.55163600
H	0.88042400	0.55254900	-2.00626800
C	2.44730500	1.06433800	-0.24145700
C	2.11356300	1.28524800	-1.64354000
C	-2.52893600	3.16922900	-0.47812100
H	-2.16498900	3.66567800	-1.38237000
H	-3.24733500	3.80829600	0.03058800
C	-2.36914500	0.95213400	-0.71781700
O	-3.23307900	1.96044500	-0.89157700
C	-1.39468100	2.62085500	0.40236700
C	-2.82930200	-0.41249500	-1.20037100
N	-2.45017500	-1.42742000	-0.20245800
H	-3.13362800	-2.17409400	-0.14787800
C	-0.59902300	-3.32960300	-1.26958800
H	0.44515500	-3.64836100	-1.25233800
H	-1.25056800	-4.18244900	-1.06585300
H	-0.82178400	-2.93020900	-2.26127100
C	-0.79119800	-2.78805600	1.63180100
C	0.54391600	-3.54972500	1.74895700
H	1.40033300	-2.89555500	1.56349100
H	0.63782400	-3.94358100	2.76613000
H	0.59985600	-4.39735100	1.06029900
C	-0.88843800	-1.72162400	2.73450300
H	-0.96234000	-2.22224900	3.70568300
H	-0.00357000	-1.08302200	2.75340700
H	-1.77068100	-1.08911100	2.60912100
C	-1.97407300	-3.77155900	1.77382700
H	-1.87745800	-4.29312300	2.73154200
H	-2.93683700	-3.25204200	1.78569400
H	-1.99002000	-4.53450400	0.99064000
H	0.91237400	-0.36789600	1.02693200
H	1.64914600	-1.30074800	-0.88474500
C	-4.35070000	-0.47803700	-1.49600100
H	-4.55751300	0.37353600	-2.15172600
C	-5.22408200	-0.33752000	-0.23979400
H	-6.28005700	-0.32340400	-0.52529300
H	-5.08398400	-1.17405800	0.45277500
H	-5.00680200	0.58669800	0.29931000
C	-4.68949600	-1.75776300	-2.27696600

H	-5.74291100	-1.74648500	-2.57041800
H	-4.08432000	-1.84537500	-3.18517200
H	-4.53344200	-2.66448400	-1.68057100
H	-2.31605500	-0.57812200	-2.15964200
C	-1.71202900	2.62993100	1.93397000
C	-3.00485000	1.84968600	2.24027300
H	-3.87563500	2.28088800	1.73694500
H	-2.92127800	0.80139500	1.93959900
H	-3.20385200	1.87283000	3.31591900
C	-1.87233800	4.09544100	2.38796100
H	-1.98666200	4.13213800	3.47590400
H	-0.99029800	4.69007900	2.12448400
H	-2.75232700	4.57947300	1.95340200
H	-0.47773500	3.18581900	0.23660800
C	-0.54381400	2.00825100	2.71954800
H	-0.25891900	1.03904300	2.31166800
H	0.33343100	2.65925700	2.70073900
H	-0.82952400	1.87258900	3.76758200
H	-0.16120800	-0.52604800	-1.99212400
C	2.94582900	0.59858000	-2.72774600
H	3.82924300	1.21150700	-2.93063800
H	3.27611900	-0.39302100	-2.42159500
H	2.37610900	0.51342600	-3.65645400
C	2.27067200	2.21058800	0.74314900
H	3.07975300	2.93697000	0.59317600
H	1.32600400	2.73341500	0.63249000
H	2.33762600	1.83613100	1.76427200
C	3.70990000	0.29306400	0.09515800
C	3.74869400	-0.85170900	0.89933800
C	4.93087200	0.85742100	-0.31452600
C	4.96529000	-1.43902300	1.25324500
H	2.81770800	-1.28201600	1.24337200
C	6.14775100	0.27106900	0.03318200
H	4.92955200	1.76895000	-0.90537800
C	6.16960000	-0.88602600	0.81530800
H	4.96705500	-2.33124100	1.87274300
H	7.07666900	0.72148400	-0.30407500
H	7.11509500	-1.34629300	1.08613200
C	1.59312800	2.65408200	-2.07478400
H	1.22491100	2.62111300	-3.10269900
H	0.79336300	3.02683300	-1.43877700
H	2.42571000	3.36537800	-2.03124800

G

N	1.12126600	1.11016500	0.50832500
P	0.73385000	-1.93114700	-0.55649400
Ir	-0.58797000	-0.02637000	-0.57452900
H	-0.84156600	-0.76650400	0.85701700
C	-2.27086900	1.72159800	-0.78612800
C	-2.33706700	0.99531000	0.46789300
C	2.13558400	2.71293500	1.89973800
H	1.41328300	3.11308700	2.61667900
H	3.05952000	3.28499900	1.95271800
C	1.75111200	0.54654500	1.48363800
O	2.44409700	1.34691000	2.30288500
C	1.54895200	2.54431900	0.48513400
C	1.76914300	-0.93574000	1.81393500
N	1.97168100	-1.70995400	0.57871100
H	2.54261600	-2.53292600	0.73089500
C	-0.25532700	-3.39503200	-0.05688500
H	-1.10997800	-3.50937600	-0.72722200
H	0.34288000	-4.30824400	-0.06100800
H	-0.62630400	-3.21350500	0.95406300
C	1.63190900	-2.42450100	-2.12769000
C	0.57757300	-2.70273200	-3.21667300
H	-0.04875200	-1.82562200	-3.40309800
H	1.09122700	-2.95634100	-4.14980600
H	-0.07175900	-3.54479900	-2.95849300
C	2.56555700	-1.28511100	-2.56742500
H	3.10514800	-1.59693300	-3.46780600
H	2.01056900	-0.37736600	-2.81056600
H	3.29991000	-1.04709100	-1.79401100
C	2.47589100	-3.69380300	-1.87414900
H	2.98075600	-3.96474200	-2.80709400
H	3.25764700	-3.52888000	-1.12574300
H	1.87231900	-4.55284400	-1.57148400
H	-0.97286500	1.22549600	-1.59338300
H	-1.68915700	-0.90323400	-1.26163800
C	2.83227700	-1.31503600	2.87579200
H	2.67625500	-0.62107100	3.70767200
C	4.27652300	-1.14618700	2.37742300
H	4.97411600	-1.34965900	3.19515100
H	4.51382800	-1.84232200	1.56610400
H	4.46158600	-0.13124700	2.01842500
C	2.58055100	-2.73690800	3.40235500
H	3.27337700	-2.96011600	4.21850600
H	1.55980900	-2.84885100	3.78212100
H	2.73645000	-3.50045800	2.63135600
H	0.78272500	-1.14052500	2.25804600
C	2.53912000	2.88053600	-0.67114400
C	3.81428700	2.02368800	-0.57553200
H	4.37014800	2.21158700	0.34898800
H	3.57576300	0.95737500	-0.62037700
H	4.48209000	2.25303700	-1.41154100
C	2.89737500	4.37824900	-0.57435400
H	3.54719900	4.65537600	-1.41012500
H	1.99912600	5.00385200	-0.62739700
H	3.43159200	4.62808200	0.34734400
H	0.67051900	3.17630400	0.36263200
C	1.85758800	2.63849400	-2.02799700
H	1.61633500	1.58643900	-2.16537700
H	0.92860300	3.21283600	-2.11462100
H	2.52150000	2.94849600	-2.84130700
H	0.09695900	0.14550200	-2.04084500
C	-3.26303700	1.38829100	-1.89478700
H	-4.17661100	1.96709700	-1.72194500
H	-2.86341000	1.67316500	-2.87093300
H	-3.52437900	0.33166500	-1.91090100
C	-1.88645700	1.68247200	1.75638000
H	-0.91957900	2.17272300	1.66343300
H	-2.62828100	2.43435100	2.05186400
H	-1.81250700	0.94590800	2.55774000
C	-3.56769700	0.13921500	0.71143400
C	-3.57814000	-1.25078800	0.87009200
C	-4.78021700	0.83465800	0.87304800
C	-4.76326900	-1.92927300	1.16681900
H	-2.65710000	-1.80491100	0.74805600
C	-5.96457100	0.16012000	1.16688500
H	-4.79721400	1.91642800	0.76867600
C	-5.96104200	-1.22930200	1.31340300

H	-4.74504800	-3.00935800	1.28034900
H	-6.88786600	0.72037000	1.28131800
H	-6.88172400	-1.75850400	1.54011400
C	-1.87057300	3.18826600	-0.79570200
H	-2.76596400	3.76546900	-0.53645600
H	-1.10243800	3.43592600	-0.06955500
H	-1.53982500	3.50911400	-1.78574100

H

N	0.26479500	0.92454100	0.49505600
P	-2.26762200	-0.75195800	-0.58496100
Ir	-0.07146000	-1.28172200	-0.07654600
H	-0.08110200	-0.79557200	-1.63320500
C	2.08083900	-2.27894000	0.47507900
C	2.06438400	-1.63351400	-0.83293100
C	1.55758600	2.88204800	0.71875200
H	2.45713100	2.83932500	0.10297500
H	1.59074900	3.75156100	1.37203000
C	-0.18513100	1.86603300	-0.26246900
O	0.41921200	3.05689000	-0.17722800
C	1.25459700	1.54634700	1.42468400
C	-1.31064100	1.76421900	-1.27708500
N	-2.39329300	0.92834700	-0.74201400
H	-3.30815200	1.24606500	-1.03910000
C	-2.76265500	-1.53685300	-2.16812600
H	-2.65095800	-2.62029700	-2.08804700
H	-3.79052100	-1.28971100	-2.43951600
H	-2.08330400	-1.17604700	-2.94269800
C	-3.60709600	-1.18075300	0.65606900
C	-3.63037900	-2.71094000	0.83562800
H	-2.65980300	-3.09212200	1.16589700
H	-4.37208100	-2.96701400	1.59927800
H	-3.91338100	-3.22937200	-0.08535500
C	-3.30521000	-0.49034500	1.99712500
H	-4.12166800	-0.70144000	2.69558800
H	-2.38150100	-0.86141300	2.44553600
H	-3.22330800	0.59350000	1.88289800
C	-4.97834600	-0.69290200	0.13594400
H	-5.74245000	-0.96310900	0.87193000
H	-5.01750800	0.39490500	0.02156800
H	-5.26126300	-1.15933300	-0.81089900
H	0.77404800	-1.98101200	1.18156800
H	-0.40308900	-2.73285100	-0.57259000
C	-1.85914800	3.15085700	-1.70339100
H	-0.98185200	3.73301200	-2.00139900
C	-2.56199400	3.89457300	-0.55698100
H	-2.86405700	4.89087400	-0.89259600
H	-3.46510300	3.37164800	-0.22616500
H	-1.90340500	4.01302100	0.30690300
C	-2.76843100	3.02024400	-2.93505200
H	-3.05596200	4.01389600	-3.29019400
H	-2.26210100	2.49966500	-3.75447400
H	-3.69694500	2.48001600	-2.71591300
H	-0.85829500	1.31013100	-2.17283400
C	0.71621900	1.69808600	2.88034400
C	-0.55403600	2.56834400	2.90994400
H	-0.37300900	3.57954800	2.53128200
H	-1.35328400	2.11801800	2.31392200
H	-0.91714000	2.66456000	3.93767100
C	1.82463900	2.34623000	3.73538700
H	1.49918900	2.40717300	4.77853000
H	2.74407400	1.75043800	3.70519500
H	2.06864400	3.36284500	3.41183600
H	2.14975500	0.92778600	1.44083500
C	0.39484700	0.31763800	3.47679700
H	-0.37080200	-0.19574900	2.89721700
H	1.28352700	-0.31944800	3.50821600
H	0.03116100	0.43011500	4.50334100
H	-0.76699500	-1.62946100	1.35419000
C	2.90590700	-1.74571600	1.64586400
H	3.90812600	-2.18084900	1.57760900
H	3.00722800	-0.66514000	1.65528700
H	2.47763900	-2.07109400	2.59614700
C	2.06417100	-2.52330600	-2.07964800
H	3.07041200	-2.94104400	-2.21426000
H	1.35003600	-3.34276300	-2.02575800
H	1.82783100	-1.92602500	-2.96065600
C	2.84991300	-0.36629100	-1.10238200
C	2.33026500	0.64773800	-1.92433300
C	4.19150800	-0.25419800	-0.70256900
C	3.09371700	1.75564000	-2.28523500
H	1.31105800	0.55899700	-2.28019400
C	4.95878400	0.86065500	-1.05123300
H	4.65966200	-1.04999100	-0.13629300
C	4.41143100	1.87661500	-1.83473800
H	2.65470700	2.52781000	-2.90982300
H	5.99016300	0.92324600	-0.71744500
H	5.00631700	2.74405300	-2.10364000
C	2.00138700	-3.80378700	0.53813500
H	1.26484000	-4.22797400	-0.13968400
H	2.98895300	-4.20099000	0.27614600
H	1.76354700	-4.13207600	1.55269300

TSs for the migratory insertion pathway

I

N	-0.76164900	1.30361300	-0.56675600
P	-1.54609300	-1.70148700	0.41017800
Ir	0.15771400	-0.89426700	-1.00921300
H	-0.33593400	-0.54524500	-2.56952400
C	2.54636200	-1.04279000	-0.58872300
C	2.15247900	-0.51682500	-1.91405700
C	-1.32214600	3.44995200	-1.36371300
H	-1.20915100	3.47924800	-2.45142700
H	-1.50695300	4.45391700	-0.98612200
C	-2.03993000	1.43379000	-0.70077800
O	-2.49666400	2.64324600	-1.06387000
C	-0.18794300	2.68506100	-0.66462400
C	-3.11668500	0.37198200	-0.53774700
N	-2.77979900	-0.53897500	0.56087900
H	-3.61122500	-0.88488300	1.02569900
C	-2.32131000	-3.22101600	-0.27983400
H	-1.57645200	-4.01789100	-0.34436600
H	-3.17089100	-3.55977300	0.31754800
H	-2.66400400	-2.99119900	-1.29172600

C	-1.13004600	-2.10595300	2.19425800
C	-0.02566300	-3.18005100	2.20039000
H	0.85457000	-2.86172200	1.63475400
H	0.28855600	-3.36977000	3.23211200
H	-0.37187600	-4.12938200	1.78091000
C	-0.62931800	-0.81700000	2.86973000
H	-0.33058800	-1.03853500	3.89994500
H	0.23489300	-0.39990700	2.35046500
H	-1.40685500	-0.04930500	2.89214600
C	-2.36779600	-2.62858100	2.95180700
H	-2.08595100	-2.83085900	3.99095900
H	-3.17758000	-1.89239700	2.97831300
H	-2.75367000	-3.56126000	2.53185000
H	1.38266700	-1.12433800	0.16545900
H	0.41396300	-2.37511200	-1.42155100
C	-4.52831600	0.98224400	-0.31652500
H	-4.67206000	1.69787900	-1.13112100
C	-4.65237100	1.73758100	1.01518100
H	-5.64561400	2.18944600	1.09419300
H	-4.52368300	1.07023700	1.87331300
H	-3.91033300	2.53545500	1.09356800
C	-5.61308400	-0.09747500	-0.45550100
H	-6.60294400	0.36691200	-0.43004900
H	-5.51800600	-0.64320100	-1.39993800
H	-5.58515400	-0.82886900	0.36081000
H	-3.15939100	-0.15117500	-1.50868700
C	0.21421400	3.25603300	0.72924300
C	-0.99636300	3.33252600	1.67841400
H	-1.78727200	3.98230300	1.29034000
H	-1.42332400	2.34112400	1.85788400
H	-0.68443800	3.73870700	2.64559800
C	0.81316800	4.66221000	0.52833100
H	1.18370200	5.04627900	1.48411700
H	1.65579700	4.63746500	-0.17185000
H	0.07951800	5.38189100	0.15149000
H	0.69626800	2.66020300	-1.29643200
C	1.28362300	2.34803000	1.34884200
H	0.92987300	1.31953000	1.40980900
H	2.20671700	2.34879600	0.76262900
H	1.53661300	2.68267600	2.35973200
C	2.42860700	-1.38557400	-3.13946100
H	3.50727500	-1.39466900	-3.35040800
H	2.09526800	-2.41747700	-3.03633300
H	1.92439400	-0.96015400	-4.01127100
H	-1.10484500	-0.88193600	-2.11867300
C	2.39006800	0.95216600	-2.24222600
H	2.35885000	1.59172200	-1.36267600
H	3.37182800	1.08678600	-2.71434400
H	1.64318800	1.30048000	-2.95999900
C	3.35036600	-0.21230900	0.39935500
C	4.47054500	0.50785400	-0.03548700
C	3.10416200	-0.30119800	1.77597400
C	5.28786500	1.17095700	0.88307500
H	4.71998900	0.54438300	-1.08900200
C	3.91475000	0.36221600	2.69421100
H	2.26578200	-0.88902200	2.13533000
C	5.00638600	1.11169000	2.24845200
H	6.14677900	1.73110200	0.52612800
H	3.69292500	0.29397600	3.75475600
H	5.63770500	1.63461800	2.96023900
C	3.02075700	-2.50113400	-0.50609900
H	2.39654100	-3.18561900	-1.07717000
H	4.04304700	-2.53996300	-0.89651000
H	3.04809300	-2.83535100	0.53305000

J

N	-1.38616900	1.15672900	-0.19648700
P	-0.64811900	-1.97310800	0.04861200
Ir	0.55447100	-0.11948200	-0.82553400
H	0.10695200	0.21052800	-2.40296000
C	2.42515000	1.10568400	0.03243300
C	2.05927000	1.39903900	-1.37234000
C	-2.82799400	2.94267400	-0.67246000
H	-2.49853700	3.37223700	-1.62312400
H	-3.59348400	3.57272000	-0.22523000
C	-2.49253100	0.73321600	-0.71616900
O	-3.44038400	1.64825100	-0.96013400
C	-1.65933500	2.56545100	0.24810100
C	-2.84352400	-0.70144900	-1.07419000
N	-2.31535800	-1.60368300	-0.04083500
H	-2.91341400	-2.41243200	0.08291000
C	-0.39689500	-3.46179600	-1.00403600
H	0.66560300	-3.71254000	-1.04084900
H	-0.96403700	-4.32183100	-0.63906100
H	-0.72660900	-3.21813600	-2.01710800
C	-0.44118500	-2.59539200	1.81366500
C	0.87155900	-3.39485900	1.91869000
H	1.73880600	-2.79891900	1.62454000
H	1.02116000	-3.69150100	2.96235200
H	0.85577300	-4.30724500	1.31662600
C	-0.38673800	-1.38502700	2.75694400
H	-0.30150100	-1.73190000	3.79246600
H	0.47934900	-0.75250200	2.54079200
H	-1.29034200	-0.77622500	2.67721900
C	-1.62856100	-3.50007100	2.20503700
H	-1.45261100	-3.89591900	3.21105100
H	-2.56898100	-2.94276200	2.23385000
H	-1.74499800	-4.35867800	1.53570200
H	1.53267600	0.20657900	0.55280000
H	1.64372100	-1.11715800	-1.32495800
C	-4.36553700	-0.92674800	-1.28244600
H	-4.68244400	-0.15017000	-1.98560700
C	-5.19133500	-0.77460700	0.00318600
H	-6.25395100	-0.90111300	-0.22454100
H	-4.92360100	-1.52820500	0.75091800
H	-5.05754400	0.21098700	0.45266000
C	-4.61679400	-2.28773800	-1.95134900
H	-5.67718200	-2.38982300	-2.19863900
H	-4.04035200	-2.39322300	-2.87634900
H	-4.35947200	-3.12798000	-1.29601600
H	-2.38232800	-0.88587100	-2.05794900
C	-1.99798100	2.66107600	1.77455500
C	-3.21242500	1.78635600	2.13944200
H	-4.11247300	2.08511500	1.59391800
H	-3.02167200	0.72876800	1.93444900
H	-3.42907600	1.88425300	3.20739400
C	-2.30370700	4.13471600	2.11518800
H	-2.42306200	4.24274600	3.19785300
H	-1.48445800	4.79228000	1.80316600
H	-3.22628000	4.49517600	1.65056100

H	-0.79160900	3.19327400	0.04719600
C	-0.78591300	2.22055500	2.61388000
H	-0.36826700	1.28082400	2.25381600
H	0.00124500	2.97823600	2.59585200
H	-1.08118100	2.08485100	3.65918200
C	2.22060800	2.21620500	1.06228200
H	1.25576900	2.69832500	0.94697500
H	2.30166400	1.82882100	2.07943600
H	2.99528200	2.97414800	0.91610800
C	3.00759700	0.91526800	-2.46673200
H	3.85345500	1.61075700	-2.55351600
H	3.41244800	-0.07742300	-2.27157700
H	2.49103000	0.89533100	-3.42985800
H	-0.33315400	-0.58988800	-2.18021700
C	1.51124200	2.77775700	-1.71534000
H	0.72061500	3.11693200	-1.05058000
H	2.31868200	3.52239800	-1.68121000
H	1.10802000	2.77231200	-2.73122100
C	3.72557600	0.34982900	0.26600800
C	3.79434900	-1.01522400	0.55051400
C	4.92023700	1.08392600	0.18953800
C	5.02483300	-1.64166700	0.76185300
H	2.88003600	-1.59293600	0.59031000
C	6.14997300	0.45993900	0.39589700
H	4.89307000	2.14356300	-0.04574500
C	6.20768800	-0.90618900	0.68576000
H	5.05304500	-2.70426300	0.98402100
H	7.06396600	1.04216800	0.32665600
H	7.16572900	-1.39079600	0.84731200

K

N	-1.24519200	-1.08770100	0.33732100
P	-0.46996000	1.94989700	-0.42919100
Ir	0.51711000	-0.15610000	-0.80888300
H	1.38157000	0.11602800	0.60055400
C	1.94433800	-1.86544800	-0.59903700
C	2.22482300	-1.06660900	0.59799200
C	-2.33667400	-2.68517700	1.66180000
H	-1.58751400	-3.27486800	2.19846100
H	-3.32094700	-3.12488400	1.80674200
C	-1.64724100	-0.56610600	1.44843100
O	-2.35990000	-1.35398000	2.26238900
C	-1.94983900	-2.40362400	0.20020600
C	-1.37896300	0.85092700	1.92403000
N	-1.63416500	1.78448500	0.81162600
H	-1.97908800	2.67439000	1.15489700
C	0.74288300	3.19188900	0.18683900
H	1.54540600	3.32931900	-0.54112300
H	0.26936500	4.15529500	0.38823400
H	1.17641200	2.80956400	1.11458900
C	-1.39762600	2.82714900	-1.80472100
C	-0.36642000	3.23747900	-2.87530200
H	0.22066600	2.37908800	-3.21924200
H	-0.89495300	3.65096200	-3.74065000
H	0.32315300	4.00497700	-2.51215700
C	-2.42879400	1.86491600	-2.41687400
H	-3.03137300	2.40507300	-3.15473400
H	-1.94564100	1.03527100	-2.93815700
H	-3.10328900	1.45625200	-1.65988600
C	-2.13284000	4.07085500	-1.26364000
H	-2.60459500	4.59414700	-2.10224000
H	-2.92958000	3.79803700	-0.56455800
H	-1.46272800	4.78298400	-0.77449500
H	0.08765600	-0.98936200	-2.21640600
H	1.67238000	0.56267200	-1.59337500
C	-2.20093100	1.23980200	3.17835100
H	-2.05182000	0.42347700	3.89241000
C	-3.70723800	1.36328800	2.90004000
H	-4.24144500	1.54793200	3.83665500
H	-3.92943800	2.19736600	2.22659500
H	-4.10898500	0.45090000	2.45364100
C	-1.63849400	2.52140800	3.81320000
H	-2.16548100	2.73786200	4.74672400
H	-0.57196200	2.42226800	4.04063700
H	-1.76363100	3.39629200	3.16463000
H	-0.31952000	0.86175300	2.22656300
C	-3.15960300	-2.35902400	-0.78340800
C	-4.22680700	-1.34742300	-0.32543500
H	-4.66140000	-1.61503500	0.64280400
H	-3.80987100	-0.33885000	-0.24493800
H	-5.04350500	-1.31256400	-1.05303700
C	-3.76816100	-3.77547900	-0.84805000
H	-4.59456900	-3.78951600	-1.56531900
H	-3.02517400	-4.51050900	-1.17760000
H	-4.16921900	-4.10684000	0.11465100
H	-1.24773500	-3.14708400	-0.17088500
C	-2.67352400	-1.97789700	-2.19096300
H	-2.34087300	-0.94211900	-2.21787600
H	-1.84803900	-2.61996700	-2.51901900
H	-3.49040800	-2.08427300	-2.91159700
H	-0.33705600	-0.17715700	-2.27153000
C	3.57476100	-0.37560000	0.67098100
C	3.79163400	0.97700500	0.40164300
C	4.66732100	-1.17840400	1.03816900
C	5.07346600	1.52482500	0.49708100
H	2.95973000	1.59871700	0.09928300
C	5.94696000	-0.63274800	1.13085000
H	4.51953400	-2.23533200	1.24016400
C	6.15516400	0.72290800	0.86150500
H	5.22173500	2.57892900	0.28216000
H	6.78162600	-1.26871000	1.41024900
C	1.34764100	-3.25250900	-0.41688800
H	2.15647600	-3.96383300	-0.19917900
H	0.63410400	-3.31960700	0.40073800
H	0.85577800	-3.58391900	-1.33493500
C	1.76414200	-1.57599800	1.96674300
H	0.72519900	-1.90055000	1.95875000
H	2.38389600	-2.42967300	2.25631400
H	1.88271500	-0.79693100	2.72297500
C	2.97634100	-1.86538200	-1.72350400
H	3.41390600	-0.88285300	-1.89695100
H	3.79169100	-2.55975200	-1.47923500
H	2.51350800	-2.20694500	-2.65315500
H	7.15206200	1.14702000	0.93275500

L

N	0.09341100	1.05091200	0.60257400
P	-1.97057000	-0.99875400	-0.76870700

Ir	0.02512800	-1.24766900	0.44449000
H	0.89500200	-1.21671900	-1.00603000
C	2.14181300	-1.79962400	0.90542900
C	2.17851300	-1.62557600	-0.55494000
C	1.24406800	3.08644700	0.72657600
H	2.23480000	2.93126400	0.28932500
H	1.18795900	4.06965500	1.18925200
C	-0.24215100	1.82525300	-0.37539400
O	0.27924500	3.05958000	-0.36979000
C	0.79268800	1.91292300	1.60724500
C	-1.14133200	1.50054600	-1.55749700
N	-2.25435400	0.64488000	-1.12911900
H	-3.06725200	0.78543000	-1.71936100
C	-1.88327400	-1.89129200	-2.37553400
H	-1.72961700	-2.95747800	-2.19335200
H	-2.77868800	-1.74315800	-2.98327700
H	-1.01334500	-1.51076300	-2.91658800
C	-3.57782900	-1.53482300	0.04064400
C	-3.45565800	-3.02947700	0.39725700
H	-2.60662800	-3.21660500	1.06145800
H	-4.36686900	-3.34994300	0.91339100
H	-3.34210600	-3.65756700	-0.49162200
C	-3.79351100	-0.70412900	1.31960900
H	-4.74369200	-0.99648200	1.77894600
H	-3.00741200	-0.88056800	2.05891600
H	-3.83039500	0.36688500	1.10420100
C	-4.77377300	-1.31929600	-0.90990300
H	-5.68872400	-1.64609900	-0.40451800
H	-4.91385000	-0.26507200	-1.16996000
H	-4.68674700	-1.90044700	-1.83151300
H	-0.13738000	-1.43603200	2.10731700
H	-0.15258100	-2.79915900	0.27987400
C	-1.67162600	2.77384300	-2.27188800
H	-0.78721600	3.38336800	-2.48077900
C	-2.62894500	3.59610900	-1.39721200
H	-2.93983800	4.49711600	-1.93409500
H	-3.53222700	3.03168800	-1.14497600
H	-2.15070400	3.90561800	-0.46521800
C	-2.30964600	2.40942000	-3.62186700
H	-2.55987900	3.32157400	-4.17080300
H	-1.62932500	1.81575200	-4.24147600
H	-3.24153900	1.84334800	-3.50708800
H	-0.49892800	0.98914900	-2.29285100
C	-0.11767900	2.30882900	2.80986900
C	-1.32945500	3.14775000	2.36438300
H	-1.03272400	4.09035400	1.89388800
H	-1.95635000	2.59265300	1.65962500
H	-1.94828600	3.39513300	3.23254400
C	0.74632200	3.11351500	3.80243300
H	0.15069600	3.38456700	4.67959700
H	1.60331800	2.52427200	4.14757600
H	1.12742100	4.04290100	3.36767200
H	1.64728500	1.37268700	2.00321900
C	-0.62931400	1.04295500	3.51861200
H	-1.34661300	0.51136400	2.89198700
H	0.19072100	0.36031000	3.76906600
H	-1.13629300	1.31427000	4.44997600
C	2.26698300	-3.21018300	1.47488700
H	1.64822100	-3.94872900	0.96703800
H	3.31418100	-3.53831100	1.41155200
H	1.98407000	-3.20869800	2.53086600
C	2.27366500	-2.86680000	-1.44977600
H	3.29167100	-3.26322100	-1.37741600
H	1.57075800	-3.64367100	-1.15288500
H	2.08428900	-2.59991200	-2.49197900
C	2.99715500	-0.47735300	-1.11767100
C	2.44855100	0.56400600	-1.86752800
C	4.38865000	-0.51732500	-0.93684800
C	3.25557600	1.57621000	-2.39103000
H	1.38269400	0.57784600	-2.04868200
C	5.19980500	0.48658400	-1.46464700
H	4.83648600	-1.33210900	-0.37600100
C	4.63523200	1.54393900	-2.18413000
H	2.80305700	2.38304900	-2.95943500
H	6.27364900	0.44444200	-1.30987400
H	-1.00219400	-1.34380600	1.77425700
H	5.26708600	2.32996500	-2.58591700
C	2.90347400	-0.81081400	1.77809400
H	3.93872300	-1.15166200	1.91103400
H	2.95020900	0.18605400	1.34572600
H	2.45160700	-0.74966200	2.77138900

M

N	-1.18773000	1.24030200	-0.34855600
P	-1.02013400	-1.98062100	0.13499400
Ir	0.68380500	-0.38626100	-0.17150600
H	0.87198200	0.13358000	-1.76613900
C	2.33085800	0.16170700	1.49540400
C	2.52550500	0.80989900	0.18520700
C	-2.00351900	3.13044700	-1.49489000
H	-1.32993400	3.36062700	-2.32553600
H	-2.73292800	3.93010400	-1.38009200
C	-2.11670800	0.91609800	-1.18512200
O	-2.73071600	1.91574800	-1.83959900
C	-1.25536500	2.73093000	-0.21324300
C	-2.59504300	-0.47686600	-1.55779100
N	-2.51818400	-1.36693300	-0.39441900
H	-3.25384600	-2.06398400	-0.41327300
C	-0.70202800	-3.52075600	-0.81908400
H	0.26409200	-3.94017900	-0.52925400
H	-1.48634000	-4.26754300	-0.67387200
H	-0.65539000	-3.25164100	-1.87747200
C	-1.38105800	-2.52765800	1.89246400
C	-0.16749100	-3.32391900	2.40824900
H	0.76228800	-2.75659000	2.30680700
H	-0.30829200	-3.54754000	3.47089500
H	-0.04672800	-4.27594200	1.88329400
C	-1.60024000	-1.27367600	2.75780700
H	-1.85856600	-1.57867300	3.77761300
H	-0.69817200	-0.65818900	2.81255700
H	-2.41362700	-0.65583200	2.36790500
C	-2.64537700	-3.40926200	1.94329700
H	-2.80423400	-3.73937900	2.97539700
H	-3.54088900	-2.85823100	1.64023300
H	-2.55434900	-4.30732500	1.32561200
H	1.05777500	-0.48048200	1.46878200
H	1.67879000	-1.56031600	-0.38911100
C	-4.03033300	-0.48060200	-2.15317900
H	-4.01369100	0.25231100	-2.96517900
C	-5.10314100	-0.05181900	-1.14176100
H	-6.08301200	-0.03058200	-1.62795500
H	-5.16899400	-0.74629100	-0.29772900
H	-4.90218200	0.94541100	-0.74468100
C	-4.35450100	-1.84920000	-2.77290600
H	-5.31345200	-1.80087300	-3.29653500
H	-3.58913600	-2.15622800	-3.49329800
H	-4.44391300	-2.64024200	-2.01900600
H	-1.92950400	-0.80800400	-2.37292300
C	-1.96366100	3.17172700	1.10710200
C	-3.40651800	2.63919700	1.18220900
H	-4.02538000	3.00291700	0.35620500
H	-3.42654300	1.54537700	1.17220400
H	-3.87569200	2.97112600	2.11343400
C	-1.97054600	4.71244400	1.16600100
H	-2.38545100	5.04498500	2.12276800
H	-0.95524700	5.11616900	1.08158900
H	-2.58007200	5.15934800	0.37466000
H	-0.24606700	3.13901500	-0.22075200
C	-1.17253500	2.64115000	2.31172000
H	-1.04707700	1.55820000	2.25526600
H	-0.18541900	3.11132100	2.36219500
H	-1.69288200	2.87838900	3.24509900
H	0.24707300	-0.55645000	-1.79934500
C	2.30188700	2.31840700	0.09952100
H	1.41004400	2.63997100	0.63411900
H	3.15628400	2.85968000	0.52605000
H	2.20366700	2.62380300	-0.94484600
C	3.20190900	-1.04095800	1.85399800
H	3.41706000	-1.67143200	0.99276700
H	4.15067100	-0.66771100	2.25181100
H	2.72832000	-1.64443000	2.63179700
C	3.76973800	0.41819600	-0.59465800
C	3.76872500	-0.20439900	-1.84852500
C	5.01317500	0.78429000	-0.04571600
C	4.96365400	-0.46682500	-2.52536800
H	2.83050900	-0.49976300	-2.30168500
C	6.20799100	0.51948200	-0.71318300
H	5.04475300	1.28496600	0.91840500
C	6.18801300	-0.11114100	-1.96008000
H	4.93163900	-0.95338600	-3.49602600
H	7.15249700	0.80769500	-0.26083800
H	7.11589200	-0.31922000	-2.48443500
C	2.03159300	1.01552600	2.72231100
H	1.27366700	1.76918100	2.54307300
H	1.71544000	0.39581900	3.56505700
H	2.96083300	1.52378900	3.00434600

N

N	-0.05377400	1.19414300	0.05633200
P	-2.21508200	-1.18208200	-0.17835100
Ir	0.10625200	-1.13415600	-0.62011500
H	0.53782200	-0.65547000	-2.13052400
C	1.78614000	-2.05029100	0.82896600
C	2.26034900	-1.58757400	-0.50726900
C	0.82330700	3.28417900	-0.53826700
H	1.48337500	3.11957200	-1.39386900
H	0.92155800	4.30846900	-0.18605900
C	-0.89514800	1.85890300	-0.66791300
O	-0.55483100	3.11146400	-1.00117900
C	0.95927500	2.19483200	0.53449200
C	-2.22773800	1.37303300	-1.21449500
N	-2.82786700	0.40506300	-0.28593100
H	-3.84025800	0.45339000	-0.29678400
C	-3.11528200	-2.19339700	-1.42283100
H	-2.72688300	-3.21442100	-1.41560200
H	-4.19288700	-2.20901000	-1.24457200
H	-2.92095100	-1.75901300	-2.40675400
C	-2.79314400	-1.79334900	1.49406000
C	-2.44146700	-3.29069500	1.59596400
H	-1.37718400	-3.46903200	1.40943400
H	-2.66807800	-3.64333500	2.60755800
H	-3.02081200	-3.89956100	0.89602700
C	-2.04852000	-1.00499000	2.58384600
H	-2.39656000	-1.33023400	3.57003600
H	-0.97059500	-1.18159000	2.53532200
H	-2.22494500	0.06981900	2.49288700
C	-4.31239400	-1.58723300	1.65898700
H	-4.62076800	-1.98694900	2.63094800
H	-4.58226300	-0.52655800	1.64583300
H	-4.89429100	-2.10889900	0.89383800
H	0.49845600	-1.76064700	0.93937500
H	0.00217800	-2.58793600	-1.17138200
C	-3.21638600	2.52787900	-1.53295600
H	-2.66202800	3.21542000	-2.17925800
C	-3.67497500	3.30249300	-0.29024200
H	-4.36939800	4.09513500	-0.58396300
H	-4.19440000	2.65539500	0.42399800
H	-2.83192600	3.76763900	0.22325000
C	-4.41416700	1.99876200	-2.33855600
H	-5.03242000	2.83582600	-2.67496600
H	-4.09058400	1.43851600	-3.22209400
H	-5.06206600	1.34455600	-1.74307000
H	-1.99466500	0.91456900	-2.18929700
C	0.67636100	2.71892800	1.98324000
C	-0.70237100	3.39597100	2.08900600
H	-0.80687900	4.24050800	1.40175100
H	-1.50851000	2.68421900	1.88758600
H	-0.84733000	3.77994800	3.10341100
C	1.78327500	3.73175800	2.34477900
H	1.67853800	4.03413300	3.39159100
H	2.77827800	3.28949500	2.22072000
H	1.73811300	4.64186400	1.73900100
H	1.94836100	1.73915400	0.50910100
C	0.73239300	1.56769700	2.99938300
H	0.10283500	0.73324400	2.68979000
H	1.75401700	1.20508000	3.13099700
H	0.38287400	1.91488700	3.97704400
C	2.24651300	-1.30456100	2.07926000
H	2.39971500	-0.24815700	1.88511300
H	1.52655400	-1.41895900	2.89285600
H	3.19546200	-1.73416100	2.41304300
C	2.69863600	-2.66018900	-1.51365100
H	3.68589200	-3.04900300	-1.23078500
H	2.00684600	-3.49952900	-1.58580000
H	2.79044700	-2.21415000	-2.50651500
H	-0.48741800	-0.63214800	-2.07222700
C	1.70174500	-3.55797300	1.08333900
H	1.17288000	-4.09086600	0.29488400
H	2.72052600	-3.95348600	1.15190000
H	1.19596600	-3.75510600	2.03234100

C	3.22772300	-0.42250200	-0.60416000
C	3.03201600	0.63702400	-1.50229800
C	4.44876000	-0.46739300	0.09172200
C	3.99024700	1.63946300	-1.66498600
H	2.10508200	0.69101200	-2.06342100
C	5.40460500	0.53872500	-0.05544000
H	4.66126000	-1.30408300	0.74888800
C	5.17648000	1.60283300	-0.93048300
H	3.80432400	2.45104900	-2.36282300
H	6.33166000	0.48375100	0.50767800
H	5.91862900	2.38676500	-1.04662700

O

N	-0.22689100	1.05059700	0.12730300
P	2.32651000	-0.88477900	-0.21500100
Ir	-0.00616900	-1.19429400	-0.28953900
H	0.09591800	-1.45371200	1.39479400
C	-1.98438300	-1.84438600	0.51358000
C	-1.15463200	-1.74917000	1.74558000
C	-1.33830600	2.92064700	1.01081400
H	-2.02954000	2.61189700	1.79968500
H	-1.49083300	3.97430400	0.78574800
C	0.50664500	1.64511400	1.00737300
O	0.01901800	2.77690200	1.53173200
C	-1.35113000	1.97941500	-0.20507500
C	1.85405200	1.18671100	1.54102600
N	2.67188500	0.65204000	0.44123600
H	3.65949500	0.79349900	0.62361800
C	3.11993800	-2.12872900	0.88532400
H	2.89947900	-3.13423800	0.52038000
H	4.20093600	-1.98937200	0.95761200
H	2.67775000	-2.02051500	1.87924800
C	3.31006600	-0.90014200	-1.81037700
C	3.21302200	-2.31491800	-2.41423000
H	2.17161300	-2.62059100	-2.55894500
H	3.70514700	-2.32006400	-3.39245300
H	3.70971600	-3.06396600	-1.79045600
C	2.70495000	0.13003300	-2.78109900
H	3.33486200	0.19395300	-3.67463100
H	1.70418200	-0.16193400	-3.10960300
H	2.64605500	1.12431900	-2.33012800
C	4.78534200	-0.53562500	-1.54195000
H	5.34214400	-0.60674500	-2.48211400
H	4.89265300	0.49252300	-1.18201000
H	5.26509100	-1.21197500	-0.82931300
H	-0.77110600	-1.16323600	-1.75548700
H	0.24284200	-2.72692800	-0.53432900
C	2.61309300	2.31484200	2.28867500
H	1.89866400	2.72145900	3.01105300
C	3.05686500	3.45385900	1.35796000
H	3.51337100	4.25531300	1.94620700
H	3.79996000	3.11588100	0.62850600
H	2.21106500	3.87542800	0.80956600
C	3.79590000	1.74029400	3.08363800
H	4.25047400	2.52686300	3.69251800
H	3.47671900	0.93511700	3.75363800
H	4.58571400	1.34498300	2.43405500
H	1.62708800	0.41188500	2.29199400
C	-1.15534500	2.70302100	-1.57325000
C	0.09706200	3.59980700	-1.56772400
H	0.03566900	4.40039900	-0.82424500
H	1.00065200	3.01455000	-1.36815100
H	0.21906800	4.07275700	-2.54708100
C	-2.41913400	3.54818300	-1.83411700
H	-2.34068300	4.04638400	-2.80549900
H	-3.31463400	2.91715900	-1.84703700
H	-2.56560200	4.32781700	-1.07969200
H	-2.27499700	1.40769100	-0.24397500
C	-1.01559500	1.67221000	-2.70605000
H	-0.08511700	1.11210900	-2.61079100
H	-1.85126300	0.96742200	-2.70947000
H	-1.00021900	2.18301600	-3.67419100
H	0.18655600	-0.96527800	-1.91215900
C	-1.34544600	-0.60364200	2.74417500
H	-1.66222100	0.31999200	2.26796700
H	-2.10124100	-0.89500400	3.47843900
H	-0.41860700	-0.42099700	3.29464100
C	-2.37379200	-3.25449700	0.04865100
H	-1.54639400	-3.96282900	0.07080500
H	-3.17997400	-3.64175400	0.68643100
H	-2.75189000	-3.21962200	-0.97461100
C	-3.15003700	-0.91074400	0.25643400
C	-3.49401500	-0.58653800	-1.06904300
C	-4.01182100	-0.46174800	1.26942300
C	-4.59798700	0.20766900	-1.36825700
H	-2.86730700	-0.94952000	-1.87690300
C	-5.11288400	0.35057600	0.97824100
H	-3.84533300	-0.75366300	2.29791300
C	-5.40480700	0.70199200	-0.33861100
H	-4.81946500	0.45055300	-2.40325800
H	-5.74713200	0.69686400	1.78904500
H	-6.25588200	1.33722300	-0.56382000
C	-0.80381900	-3.05299700	2.47161700
H	-0.38083100	-3.80239500	1.80508300
H	-0.08598100	-2.86046300	3.27314500
H	-1.71917800	-3.45530400	2.91775400

P

N	0.92378600	1.29671800	0.07485700
P	1.10385600	-1.91328200	-0.17750700
Ir	-0.64728800	-0.34672800	-0.18590900
H	-0.65508500	-0.66634000	1.44852600
C	-2.38894100	0.75353200	0.67937100
C	-1.83113300	0.06125000	1.85099100
C	1.74712700	3.38520100	0.73949000
H	1.09425600	3.78948900	1.51884500
H	2.46734400	4.14190800	0.43597000
C	1.88375900	1.15844300	0.92699800
O	2.49910400	2.27794500	1.32673800
C	0.97419300	2.71396400	-0.40774300
C	2.37364900	-0.15102000	1.52113900
N	2.50225500	-1.15215600	0.44546400
H	3.25986500	-1.79820800	0.63842700
C	0.70254500	-3.30288400	0.96286000
H	-0.24934400	-3.75059400	0.67009400
H	1.48145000	-4.06872500	0.98153700
H	0.58933700	-2.88318200	1.96571700
C	1.69575200	-2.72191200	-1.76060100
C	0.57571200	-3.66135700	-2.25125400
H	-0.37745300	-3.13164000	-2.35477000
H	0.84834500	-4.05852300	-3.23466100
H	0.42822700	-4.51232500	-1.58014500
C	1.96779500	-1.63699200	-2.81585900
H	2.41092400	-2.10162200	-3.70305600
H	1.04528400	-1.14411500	-3.13373900
H	2.66243500	-0.87765500	-2.44672700
C	2.99265800	-3.51864100	-1.50958400
H	3.26804400	-4.04334000	-2.43050300
H	3.82777500	-2.86022700	-1.25126800
H	2.88274400	-4.27480700	-0.72734500
H	-1.12870700	0.33441100	-1.64922000
H	-1.66337400	-1.52975800	-0.38171100
C	3.70476900	-0.00244300	2.30143400
H	3.55381200	0.84155900	2.98226400
C	4.90266400	0.32113700	1.39481000
H	5.79144300	0.49860600	2.00745700
H	5.13317200	-0.50564400	0.71494000
H	4.72100800	1.21447000	0.79306700
C	3.96917200	-1.24988900	3.15877300
H	4.85363600	-1.09223800	3.78237000
H	3.12350300	-1.47137700	3.81812600
H	4.16277300	-2.13965900	2.54851700
H	1.60805100	-0.44435400	2.25748700
C	1.64637500	2.87105100	-1.80647000
C	3.10906200	2.38841300	-1.79301700
H	3.73474500	2.97915600	-1.11641900
H	3.17787900	1.33798600	-1.49252600
H	3.53854300	2.47507700	-2.79585900
C	1.58459700	4.36392900	-2.19149400
H	2.01954500	4.50710300	-3.18543800
H	0.54899800	4.72050400	-2.22415800
H	2.14080200	5.00268900	-1.49854600
H	-0.04017900	3.10132000	-0.48231700
C	0.86307100	2.06824300	-2.85751300
H	0.98495300	0.99865600	-2.69578800
H	-0.20622300	2.30763000	-2.83248900
H	1.23675500	2.29851500	-3.86019300
C	-2.56189400	-1.15500300	2.41570000
H	-3.35287700	-0.80308800	3.08511700
H	-3.01567900	-1.75506700	1.62845200
H	-1.88172700	-1.78131400	2.99822100
H	-0.48795400	-0.31380900	-1.85964900
C	-2.28414700	2.27799000	0.63437400
H	-3.06976900	2.72567300	1.25636300
H	-1.33033000	2.65382500	0.99844000
H	-2.43114900	2.63398500	-0.38795700
C	-3.76764300	0.32890700	0.20028500
C	-4.04430200	-0.16519100	-1.08023300
C	-4.85320700	0.54801600	1.06825800
C	-5.35405000	-0.45336700	-1.47449700
H	-3.23006100	-0.33774900	-1.77309100
C	-6.16068200	0.25432000	0.68332900
H	-4.67092400	0.95599800	2.05894200
C	-6.41699200	-0.25166500	-0.59364400
H	-5.53866300	-0.83905900	-2.47307900
H	-6.97766900	0.42448200	1.37860200
H	-7.43367300	-0.48123800	-0.89856800
C	-1.13307300	0.87970800	2.93319000
H	-0.63540500	0.22685000	3.65467700
H	-0.39855800	1.57386300	2.52862400
H	-1.89364500	1.46121000	3.46531300

Ir/1b with S3

TSs for the σ-methatesis pathway

A

N	-0.95699300	1.51922300	0.16144900
P	-1.33853200	-1.42485300	-1.03037700
Ir	0.46425900	-0.21990600	-0.18383600
H	1.39255000	-1.45108000	-0.46927700
H	0.62019400	0.15841700	-1.76368500
C	1.66200500	0.01658800	1.77122900
C	2.42968500	0.78957800	0.82389700
H	1.66323200	0.81213600	-0.65019800
H	-0.20435800	-1.02857400	1.05790900
C	-2.08991700	3.57566700	0.29922200
H	-2.62596500	4.25111000	-0.36643500
H	-1.89376200	4.07315100	1.25249400
C	-2.17364100	1.35028300	0.55456800
O	-2.96006500	2.43393100	0.57448900
C	-0.82949500	2.92893800	-0.30708200
C	-2.79347000	-0.00293400	0.86890400
N	-2.78000200	-0.81823500	-0.35562000
H	-3.51192000	-0.57688500	-1.01451400
C	-1.60876400	-1.27746500	-2.83349300
H	-0.81661300	-1.81852300	-3.35452100
H	-2.57850300	-1.69027700	-3.12105000
H	-1.55438000	-0.22605300	-3.11313200
C	-1.32531400	-3.27177700	-0.66745500
C	-0.08408900	-3.92038800	-1.30649600
H	0.84182700	-3.49773300	-0.90766300
H	-0.09153400	-4.99221400	-1.08124500
H	-0.07323800	-3.81583500	-2.39571600
C	-1.30164000	-3.48621200	0.85618600
H	-1.37196100	-4.55899900	1.06453700
H	-0.37398900	-3.11751000	1.30075100
H	-2.14761100	-2.99585300	1.34676200
C	-2.61320200	-3.89233200	-1.24922200
H	-2.65227700	-4.94969900	-0.96802100
H	-3.50820200	-3.40485200	-0.85172000
H	-2.64045600	-3.84506900	-2.34143100
C	0.85781400	0.73167200	2.84730700
H	0.19570500	0.02331400	3.34950300
H	1.54621500	1.14339300	3.59702900
H	0.24421700	1.54392800	2.46096900
C	2.45175900	2.31045300	0.94262900
H	1.49589800	2.72000100	1.25695600
H	3.19289100	2.57699000	1.70354000
H	2.75242700	2.77819800	0.00295600
C	3.76039800	0.24109100	0.34097700
C	4.00550700	-0.25582800	-0.94298600
C	4.82559700	0.30566100	1.25535800
C	5.28417400	-0.68985800	-1.30339100
H	3.19536000	-0.31689500	-1.65968800
C	6.10020300	-0.12985900	0.89617800
H	4.65533500	0.69258500	2.25621500
C	6.33432300	-0.63100100	-0.38703300
H	5.45394100	-1.07505600	-2.30444100
H	6.90849100	-0.07822400	1.61945100
C	-4.19924100	0.07694200	1.50058400
H	-4.83463100	0.67709600	0.83635900
C	-4.81320700	-1.32233700	1.64597500
H	-4.18623100	-1.95463400	2.28549500
H	-5.79921400	-1.24894900	2.11438500
H	-4.92756700	-1.82685700	0.68427000
C	-4.12673400	0.76792600	2.87253800
H	-3.71831200	1.77853100	2.80689200
H	-5.12685300	0.83829200	3.30978000
H	-3.50239900	0.18599900	3.56103400
H	-2.13083900	-0.48590000	1.59315400
C	-0.72224500	3.01088000	-1.84490500
H	0.06910200	3.37034600	0.12259600
C	2.21233900	-1.32324700	2.23988100
H	2.71001600	-1.87407500	1.44293900
H	2.94490800	-1.14238600	3.03698800
H	1.41367200	-1.94335400	2.65043300
H	7.32634800	-0.97112100	-0.66831500
C	-1.99291000	2.53142800	-2.55912900
H	-1.81195300	2.44077200	-3.63436100
H	-2.32251300	1.55617400	-2.19197600
H	-2.82285400	3.23395000	-2.42709200
C	-0.34538100	4.43561000	-2.27465600
H	-0.22480000	4.48620000	-3.36088800
H	-1.12192900	5.15813200	-1.99726500
H	0.59469200	4.75698800	-1.81384200
H	0.10005200	2.34119400	-2.12007400

B

N	0.24258100	1.20270200	0.19692500
P	-2.19607500	-0.47920300	-0.91631900
Ir	-0.02008900	-0.97875500	-0.28157900
H	-0.29222900	-2.49466200	-0.60038000
H	0.22487300	-0.69679800	-1.87407100
C	1.26002200	-1.64458500	1.50063000
C	2.18842200	-1.76942800	0.39409800
H	1.48280900	-1.22970800	-0.92288600
H	-0.94374900	-1.12686700	1.04909800
C	1.10614600	3.31125700	0.77533400
H	1.23785200	4.27597400	0.28659500
H	1.66329000	3.29416700	1.71520500
C	-0.65249000	1.91581200	0.78780000
O	-0.31222500	3.17132400	1.10478800
C	1.39519000	2.08730700	-0.11655600
H	2.31737800	1.59185700	0.18936600
C	1.47058700	2.37614000	-1.63183100
C	-2.06122800	1.43909000	1.11360100
N	-2.72195200	0.90952300	-0.08560200
H	-3.10427800	1.63263500	-0.68302700
C	-2.39892600	-0.00981600	-2.67276200
H	-2.23130000	-0.88839300	-3.29828200
H	-3.39808800	0.38867000	-2.86410200
H	-1.64593600	0.74085500	-2.91527300
C	-3.47458000	-1.81523400	-0.58247800
C	-3.13727700	-3.07361400	-1.40221800
H	-2.14957300	-3.46729500	-1.14836200
H	-3.87846700	-3.84847700	-1.17904800
H	-3.17230700	-2.88771500	-2.48000900
C	-3.46943200	-2.14820300	0.92055600
H	-4.26653400	-2.87061200	1.12604000
H	-2.52012700	-2.59218800	1.22980700
H	-3.65902800	-1.26065000	1.53252500
C	-4.86359200	-1.27453200	-0.98269000
H	-5.62062900	-2.01880500	-0.71498300
H	-5.10132900	-0.34658200	-0.45464500
H	-4.94640200	-1.09867000	-2.05883600
C	3.39803700	-0.86591800	0.25883300
C	3.78079600	-0.33333400	-0.98094600
C	4.26346100	-0.71094500	1.35151700
C	4.97941800	0.36550300	-1.11885900
H	3.13265200	-0.45882700	-1.84367100
C	5.46087800	-0.00545300	1.21626900
H	4.01385500	-1.15500500	2.30826700
C	5.82014600	0.54050900	-0.01668000
H	5.25409300	0.77308600	-2.08669600
H	6.11360700	0.10952600	2.07615200
C	0.72620300	-2.91478900	2.15234800
H	0.34801900	-3.64462200	1.43909900
H	1.53310200	-3.38239000	2.73258100
H	-0.08501500	-2.66716400	2.83972700
C	2.49536000	-3.16308500	-0.15863300
H	2.97682500	-3.09001800	-1.13568000
H	3.19860700	-3.64695800	0.52869500
H	1.60752700	-3.78549700	-0.25351000
C	-2.94260300	2.49886200	1.81565600
H	-1.92616800	0.60772500	1.81287700
H	6.75078000	1.08942900	-0.12169500
C	1.39756300	-0.51212300	2.51035100
H	2.03191200	-0.84549000	3.34086500
H	1.83743700	0.39083900	2.09291800
H	0.42100100	-0.26201100	2.92786300
C	0.21283500	3.06959600	-2.17154500
H	0.10583000	4.08387600	-1.77134000
H	0.26507700	3.15279900	-3.26107900
H	-0.69466500	2.51397200	-1.92152500
C	2.73422400	3.18343700	-1.96030300
H	2.83778100	3.30326000	-3.04301200
H	2.69285400	4.18791200	-1.52350600
H	3.63403300	2.68807600	-1.58495900
H	1.55101900	1.39417100	-2.11335400
C	-3.35748300	3.65621700	0.89178000
H	-3.84116100	4.44162200	1.47954800
H	-2.50033900	4.09854900	0.37839900
H	-4.08823600	3.33083300	0.14202200
C	-4.17199800	1.82709200	2.44223800
H	-4.78954100	1.35320000	1.67282800
H	-3.88365100	1.05900300	3.16785400
H	-4.78648300	2.56803300	2.96207600
H	-2.32368600	2.91279300	2.61959100

C

Ir	0.12521600	-1.08851200	-0.01311700
H	0.61544400	-2.53621100	-0.35791800
H	0.17816300	-1.47717100	1.57006800
H	0.68969500	-0.61871100	-1.48373400
H	-0.75913300	-1.04403500	-1.42271900
C	-2.03016400	-1.70256100	-0.90400700
C	-1.83453900	-2.19202200	0.45046000
C	-4.89490300	1.43483900	-1.72642300
C	-3.55128700	1.63104700	-2.05167700
C	-2.61001800	0.63705800	-1.77798500
C	-2.99429000	-0.55901900	-1.15886300
C	-4.35126900	-0.76002500	-0.86354100
C	-5.29332400	0.23128900	-1.14008700
C	-2.01928500	-2.70759200	-2.06346100
C	-1.58760200	-3.67772500	0.67551200
H	-3.23155300	2.55651800	-2.52138200
H	-1.56803700	0.80301400	-2.02753800
H	-4.67342200	-1.69573100	-0.41725200
H	-6.33763700	0.06139000	-0.89648400
H	-1.97764900	-2.18455000	-3.02112000
H	-1.18017300	-3.39991300	-2.00677300
H	-2.95346900	-3.27791100	-2.02606200
H	-0.91524100	-4.13015700	-0.05051200
H	-1.16807100	-3.83917800	1.67018700
H	-2.55171400	-4.20235100	0.62510000
P	2.31582000	-0.51068500	0.53106900
N	2.58316800	1.12945300	0.15180200
C	2.70743000	-0.60272900	2.31408200
C	3.66017300	-1.47382700	-0.35775400
C	1.68655300	1.89392400	-0.72679400
H	2.99380100	1.68503500	0.89383500
H	2.72564700	-1.64967000	2.62199100
H	3.67389700	-0.14210100	2.53098800
C	3.55677700	-2.96706700	0.00125200
C	3.48115900	-1.27973500	-1.87450400
C	5.03260200	-0.91536300	0.07350100
C	0.34485900	2.06727600	-0.02605900
H	1.49680500	1.28117400	-1.61300500
C	2.32542100	3.21567400	-1.20779700
H	2.59235700	-3.38790100	-0.29500900
H	4.34256600	-3.51293200	-0.53182600
H	3.70060800	-3.14730800	1.07090700
H	4.29531600	-1.79308800	-2.39691700
H	2.53546500	-1.69928400	-2.22662300
H	3.52268000	-0.22198600	-2.15239100
H	5.81419900	-1.41347400	-0.50952500
H	5.10620100	0.15971500	-0.11423600
H	5.24321100	-1.10503500	1.12983000
N	-0.38650900	1.09516000	0.40165400
O	-0.07547000	3.30825500	0.25235500
H	2.35201700	3.90946400	-0.35795400
C	1.47428300	3.83934100	-2.32686800
C	3.75928700	2.98414200	-1.70557700
C	-1.46194400	1.68431500	1.24159100
C	-1.41184800	3.16557600	0.82975000
H	0.44510500	4.02275500	-2.01181900
H	1.90557400	4.79550700	-2.63711800
H	1.45668100	3.18146800	-3.20384200
H	3.77319900	2.25288900	-2.52212600
H	4.17319600	3.92137000	-2.08951600
H	4.41990600	2.61746000	-0.91723300
H	-2.41623100	1.24909900	0.94422600
C	-1.19726600	1.40333300	2.73702700

H	-1.49952900	3.87622100	1.65069600
H	-2.13371100	3.40670800	0.04546800
H	1.91768800	-0.09059900	2.86409400
H	-5.62654600	2.20915800	-1.93554800
C	-2.63164500	-1.60078200	1.60165500
H	-2.83249800	-0.54094700	1.49024200
H	-3.60083600	-2.11188500	1.65848100
H	-2.11533700	-1.76861600	2.54721400
C	0.04760000	2.12434200	3.27393200
H	0.92703500	1.93914000	2.65089900
H	-0.10012800	3.20806100	3.32996000
H	0.27630800	1.77467700	4.28500100
H	-1.01257700	0.32579000	2.80679600
C	-2.44021500	1.74245800	3.57032700
H	-2.27158300	1.50520300	4.62496600
H	-2.68222500	2.80995500	3.50774500
H	-3.31655300	1.17934600	3.23226700

D

Ir	-0.45553900	0.03256500	-0.41710000
H	-1.43269500	1.07678500	-1.04692000
H	-0.19075600	-0.55455900	-1.91275900
H	-0.29389500	1.00468300	0.90362000
H	-1.34870600	0.02740700	0.99909000
C	-2.38797400	-0.99664600	0.56276700
C	-2.18093900	-1.41274400	-0.81405400
P	1.31346700	1.39310100	-1.08448600
N	2.60676400	1.22423000	0.01273100
C	2.05058700	0.97267100	-2.70371900
C	0.92817600	3.23097000	-1.15731400
C	2.43584600	0.61724500	1.34168300
H	3.51725700	1.05134700	-0.39852300
H	1.35424700	1.25772800	-3.49430400
H	3.00013800	1.49270500	-2.85016600
C	-0.16904800	3.49452000	-2.20469600
C	0.45200600	3.69145900	0.23211900
C	2.21868500	3.98890800	-1.53352700
C	2.15592700	-0.86777000	1.15641000
H	1.53879800	1.05126300	1.79144600
C	3.62276200	0.92976900	2.27837600
H	-1.09231800	2.95920300	-1.96751700
H	-0.39360800	4.56642300	-2.21740800
H	0.14414800	3.21423500	-3.21497700
H	0.28210900	4.77304700	0.20980500
H	-0.48618700	3.20891500	0.51663000
H	1.20132000	3.48798000	1.00311700
H	2.01933300	5.06485500	-1.49523000
H	3.02943100	3.77137900	-0.83237000
H	2.55624800	3.75606700	-2.54731200
N	1.14241100	-1.35125300	0.51944900
O	3.08483300	-1.73779900	1.57317400
H	4.50156100	0.38928000	1.90366300
C	3.31828700	0.44741600	3.70649600
C	3.92928800	2.43411500	2.28549500
C	1.41292000	-2.80116400	0.30692000
C	2.54395300	-3.06982600	1.31559400
H	3.11410100	-0.62442800	3.74740400
H	4.17178700	0.65163200	4.35944400
H	2.45219100	0.98205000	4.11438100
H	3.05658500	3.00447000	2.62489600
H	4.75198200	2.64187000	2.97617200
H	4.20961400	2.80608000	1.29793300
H	0.52966700	-3.37833300	0.58238500
C	1.76273600	-3.08459100	-1.17041800
H	3.35425600	-3.69482500	0.94226600
H	2.17917900	-3.46546400	2.26686300
H	2.20384800	-0.10480600	-2.74795500
C	-2.07771100	-1.97658700	1.69902100
H	-2.16461500	-1.47379300	2.66423600
H	-2.79833200	-2.79985600	1.67417000
H	-1.07510700	-2.39270500	1.62185500
C	-1.79840200	-2.85178500	-1.10319800
H	-1.07672300	-3.26814500	-0.40846800
H	-2.70790900	-3.46444100	-1.03666900
H	-1.40293700	-2.95011100	-2.11522300
C	-3.60113500	-0.12538300	0.84961600
C	-3.56858800	1.25898900	1.04113600
C	-4.83353400	-0.79369700	0.93692800
C	-4.74389900	1.96449200	1.31334200
H	-2.62859400	1.79287300	0.96527800
C	-6.00548500	-0.08888700	1.20796400
H	-4.87767600	-1.86825100	0.78413000
C	-5.96482400	1.29510100	1.39746400
H	-4.69958100	3.03994900	1.45712300
H	-6.94992300	-0.62140800	1.26766400
C	-3.10022400	-0.85801300	-1.89466500
H	-4.01918200	-1.45829200	-1.91715400
H	-3.37999300	0.18008300	-1.72242700
H	-2.61974900	-0.93408200	-2.87147800
H	-6.87730800	1.84489600	1.60695200
C	3.11575300	-2.49084000	-1.58909700
H	3.20845400	-1.43913500	-1.30534900
H	3.95461900	-3.03215200	-1.13912000
H	3.23433900	-2.55528600	-2.67480800
H	0.98100900	-2.59042000	-1.75763800
C	1.70691700	-4.59180300	-1.45220600
H	1.92655800	-4.79303700	-2.50485400
H	2.44435800	-5.13714900	-0.85169000
H	0.71778400	-5.00451100	-1.22750300

E

N	-0.46243500	1.06355600	0.48320500
P	2.28323900	-0.57148300	0.53660200
Ir	0.03223600	-1.13888300	0.41312800
H	0.47856100	-2.64154500	0.32171000
H	0.26026200	-1.17530200	2.03364500
C	-1.79069900	-1.67791900	-0.88255400
C	-2.19941200	-2.13105600	0.44097400
H	-1.15913800	-1.67595100	1.44770500
H	0.50577900	-0.93473500	-1.13052200
C	-1.60427300	3.13417300	0.56494000
H	-1.68288800	3.88750500	1.34929600
H	-2.32303100	3.34622700	-0.22836700
C	0.24502900	2.00182700	-0.04045800
O	-0.26944000	3.23755900	-0.01656600
C	-1.68014800	1.67356000	1.06518500
C	1.60376800	1.81711700	-0.70112200
N	2.52359600	1.07149700	0.16403400
H	2.84305600	1.61876200	0.95529300
C	3.00274300	-0.68818600	2.21446100
H	3.05196800	-1.73753600	2.51130200
H	4.00408800	-0.25328700	2.24222700
H	2.34561000	-0.15864800	2.90625500
C	3.43703900	-1.52786800	-0.59943000
C	3.27059900	-3.03876700	-0.35897400
H	2.26908400	-3.38005300	-0.63149300
H	3.99469500	-3.57538800	-0.98135900
H	3.46003600	-3.31968400	0.68204500
C	3.10999800	-1.19120200	-2.06580000
H	3.78461100	-1.76017500	-2.71421300
H	2.08464200	-1.46292800	-2.33020600
H	3.26372000	-0.13001200	-2.27974500
C	4.88729800	-1.09445400	-0.29526400
H	5.55098200	-1.54333300	-1.04124500
H	5.00310300	-0.00797700	-0.35231800
H	5.22427700	-1.43514200	0.68746000
C	-3.32462000	-1.46242300	1.23379100
H	-3.42785900	-0.40022300	1.03872500
H	-4.26612500	-1.94995000	0.96092300
H	-3.18198000	-1.61325900	2.30639100
C	2.24438300	3.13967300	-1.18644200
H	1.41149600	1.19939100	-1.58489800
C	-1.72435800	1.48281700	2.59426300
H	-2.54496400	1.18738100	0.61257400
C	-1.42546900	-2.72650600	-1.93512800
H	-0.75468400	-3.49836800	-1.56193500
H	-2.34631300	-3.20420000	-2.29456600
H	-0.94338200	-2.24202000	-2.78509500
C	-2.46319800	-0.48580800	-1.53073100
C	-1.72776000	0.48844600	-2.22410500
C	-3.86457300	-0.40482200	-1.58128500
C	-2.35875600	1.54892200	-2.87140800
H	-0.64797300	0.41175200	-2.24027600
C	-4.50283200	0.65548300	-2.22971300
H	-4.46959900	-1.17988900	-1.12509000
C	-3.75292100	1.64738200	-2.86221100
H	-1.75999900	2.29799200	-3.38110000
H	-5.58779800	0.69858000	-2.24187400
H	-4.24752300	2.47750000	-3.35727600
C	-2.15128000	-3.62682400	0.74611900
H	-3.01685000	-4.09271000	0.26084500
H	-1.24759900	-4.11225900	0.38584200
H	-2.23103900	-3.80204100	1.82154600
C	2.72473400	4.03131200	-0.02968700
H	3.03536000	5.00573000	-0.41729700
H	1.93904100	4.20032900	0.71155600
H	3.59675000	3.59785600	0.47361600
H	1.45388900	3.67490900	-1.72354400
C	3.38617200	2.84770200	-2.16844700
H	4.17861600	2.26881200	-1.68410300
H	3.03267200	2.28316200	-3.03790000
H	3.82307700	3.78311600	-2.53037400
C	-0.51032400	2.10044300	3.29904000
H	-0.47379000	3.18829900	3.17062900
H	-0.55363700	1.90001100	4.37363700
H	0.42449600	1.68166000	2.91549000
H	-1.68845000	0.39935200	2.76115300
C	-3.04876100	2.01229600	3.15992900
H	-3.11673000	1.80462000	4.23179300
H	-3.13638800	3.09709200	3.02987500
H	-3.90978200	1.54508400	2.66955600

F

N	1.39820100	-1.14856700	0.49437900
P	0.92880800	1.57275600	-1.16523300
Ir	-0.37943200	-0.29284800	-0.65298700
H	-1.54263800	0.34697100	-1.48614300
H	0.28387200	-0.77376300	-2.06417500
C	-2.00636300	-1.13340900	0.72367500
C	-1.94329600	-2.13972400	-0.31572700
H	-0.74510900	-1.75646900	-1.32844600
H	-0.64048000	0.76393800	0.55926000
C	3.16550400	-2.44271900	1.38369600
H	4.10320600	-2.83325200	0.98920300
H	2.95438900	-2.90254100	2.35179100
C	2.24231800	-0.41105400	1.13133300
O	3.33898400	-1.01210700	1.60838800
C	1.97253000	-2.52090500	0.40628300
H	1.23734800	-3.23405900	0.77920200
C	2.33258600	-2.90853200	-1.04279300
C	2.13680300	1.09948400	1.28286800
N	2.21099300	1.70951600	-0.05328200
H	3.14249000	1.70161500	-0.45425400
C	1.78565000	1.48850300	-2.77920200
H	1.04777700	1.59561300	-3.57707000
H	2.53375800	2.27893700	-2.86822800
H	2.26209700	0.51305700	-2.87339500
C	0.05509000	3.23959000	-1.13970900
C	-1.18484800	3.19603200	-2.05106900
H	-1.93235300	2.48570400	-1.69221800
H	-1.64311300	4.19056600	-2.06959800
H	-0.93129000	2.93164700	-3.08265400
C	-0.34769600	3.57941100	0.30677700
H	-0.88522500	4.53340600	0.30847500
H	-1.00454100	2.82802700	0.74991400
H	0.53199100	3.69124400	0.94489400
C	1.03979600	4.31825800	-1.64107000
H	0.58091400	5.30247300	-1.50154100
H	1.97605100	4.30405400	-1.07480900
H	1.26615800	4.21219900	-2.70524500
C	-3.01574900	-2.15685500	-1.39861300
H	-2.65612900	-2.67097200	-2.29322400
H	-3.88103800	-2.70830400	-1.01546400
H	-3.34053500	-1.15340300	-1.66879100
C	3.17292900	1.70039900	2.25878500
H	4.16764800	1.36481900	1.93779500
C	2.91900000	1.18998600	3.68715500
H	3.66810100	1.60134800	4.36999200
H	1.93290600	1.51471700	4.04025200
H	2.96831000	0.10113000	3.75389000
C	3.13863900	3.23422000	2.23391100
H	3.89000000	3.63156400	2.92285000
H	3.33882100	3.63665000	1.23860600
H	2.16073800	3.60627900	2.55990700
H	1.13780400	1.30153100	1.68070700
C	-1.42410400	-1.43435700	2.10561100
H	-2.07755800	-2.13727700	2.63638500

H	-0.42012900	-1.85460300	2.06621800
H	-1.37603100	-0.51248000	2.68738500
C	3.40124900	-1.99116000	-1.64859200
H	4.37908200	-2.13694800	-1.17665800
H	3.51994700	-2.19736500	-2.71628500
H	3.12889300	-0.93883400	-1.53622500
C	2.74334100	-4.38587500	-1.11156700
H	2.94593400	-4.67625000	-2.14672600
H	3.65368100	-4.58023900	-0.53263300
H	1.95222800	-5.03775700	-0.72540300
H	1.41284100	-2.78711000	-1.62482900
C	-1.40849600	-3.52733900	-0.00193300
H	-0.63378600	-3.52760100	0.75851700
H	-2.25121500	-4.11156700	0.38700400
H	-1.03553800	-4.03335700	-0.89515200
C	-3.29790500	-0.34407500	0.83473800
C	-3.44589200	1.02429000	0.59212600
C	-4.41885400	-1.06241200	1.29037000
C	-4.67526300	1.65964700	0.78635500
H	-2.59803700	1.59130600	0.23781000
C	-5.64731600	-0.43232000	1.48489600
H	-4.32734200	-2.12599800	1.49501000
C	-5.78119900	0.93540900	1.23132200
H	-4.76257100	2.72361900	0.58611700
H	-6.49823300	-1.01000500	1.83376400
H	-6.73689900	1.42892200	1.38017200

G

Ir	0.42006200	0.17309300	-0.32563900
H	1.38413900	1.39703600	-0.45379700
H	0.89072700	-0.06531000	1.21475300
H	-0.45929100	0.82299200	-1.54977600
H	0.62733000	-0.16267300	-1.94329300
C	1.94352100	-0.91340200	-1.83879600
C	2.38383400	-0.97552900	-0.45206000
C	2.64391000	0.04583000	-2.80050300
H	2.00203200	0.27849200	-3.65369400
H	2.93291200	0.97416100	-2.30938800
H	3.54691400	-0.44146600	-3.18152500
P	-1.05735300	1.53162600	0.85961100
N	-2.65909300	1.05221400	0.53312300
C	-0.93389700	1.41042700	2.67917300
C	-0.95168700	3.36285100	0.45565200
C	-3.00461900	0.17877700	-0.59822700
H	-3.25469800	0.92282100	1.34379400
H	0.00731300	1.85863200	3.00237600
H	-1.76758400	1.92146500	3.16641500
C	0.44041500	3.89676800	0.84116500
C	-1.19075300	3.55073900	-1.05290600
C	-2.04690400	4.10768800	1.24693300
C	-2.43820100	-1.20905600	-0.32536800
H	-2.48405400	0.55948800	-1.48070100
C	-4.51733200	0.20053600	-0.90569000
H	1.23914700	3.36822500	0.31407500
H	0.49955500	4.95581900	0.56818900
H	0.62892500	3.82588300	1.91686600
H	-1.18625600	4.62150400	-1.28268200
H	-0.40729100	3.07332600	-1.64613100
H	-2.16091000	3.14826800	-1.36031800
H	-2.03922700	5.16220400	0.95225500
H	-3.04097700	3.70511500	1.03211800
H	-1.87638500	4.07007800	2.32661500
N	-1.18538300	-1.47472400	-0.16064700
O	-3.30324800	-2.21173200	-0.12361500
H	-5.03686300	-0.30720400	-0.08299700
C	-4.80820400	-0.55509900	-2.21320100
C	-5.03225600	1.64364600	-1.00485900
C	-1.10088400	-2.86038500	0.37954300
C	-2.49772100	-3.41708000	0.05403000
H	-4.47205600	-1.59331100	-2.17533900
H	-5.88387600	-0.55779300	-2.41172300
H	-4.31495400	-0.06091400	-3.05863800
H	-4.49958000	2.19142300	-1.79120600
H	-6.09569800	1.64098600	-1.26187000
H	-4.90844600	2.19443300	-0.07011400
H	-0.34304800	-3.41821700	-0.17199900
C	-0.72950000	-2.84306100	1.87937300
H	-2.95213100	-4.01200700	0.84517200
H	-2.52446000	-3.97052300	-0.88807700
H	-0.93002600	0.35684200	2.95596900
C	2.38022400	-2.32244600	0.26198200
H	1.48321000	-2.90588500	0.08258500
H	3.23655000	-2.91191000	-0.08860900
H	2.49144900	-2.17800800	1.33712700
C	3.61525700	-0.18495200	-0.04265800
C	3.64173700	0.80261500	0.94991500
C	4.83541800	-0.57214100	-0.62535000
C	4.84416900	1.40660600	1.32593900
H	2.71551400	1.10531200	1.42159800
C	6.03631300	0.03224700	-0.25459700
H	4.84689600	-1.35793900	-1.37572200
C	6.04476500	1.03043500	0.72236300
H	4.83743700	2.17503100	2.09364300
H	6.96291400	-0.27933500	-0.72776300
H	6.97728500	1.50513400	1.01265900
C	1.44224000	-2.18549900	-2.51536800
H	0.70171100	-2.71843900	-1.92175100
H	0.99899600	-1.95716600	-3.48736000
H	2.29842100	-2.85041800	-2.67696200
C	-1.84885800	-2.27855100	2.76640300
H	-2.69423500	-2.96997500	2.84511000
H	-1.47486200	-2.10754400	3.78023800
H	-2.23246600	-1.32774800	2.38704400
H	0.13416200	-2.17390800	1.96213700
C	-0.30843900	-4.24373100	2.34259900
H	0.53958600	-4.62073400	1.76117000
H	-0.01445900	-4.22611700	3.39624000
H	-1.13173100	-4.96064700	2.24198800

H

Ir	-0.00803400	-1.06677900	-0.15180000
H	0.20249400	-2.60716200	0.05150900
H	-0.54976900	-1.01460700	1.38079900
H	1.15701400	-1.13956200	-1.30317400
H	-0.28196900	-1.34193300	-1.78362700
C	-1.70274100	-1.56298400	-1.78973600
C	-2.20560300	-1.62992600	-0.41713600
P	1.95090300	-0.59617900	1.00025100
N	2.70496800	0.73634600	0.25892800
C	1.69931200	-0.06533500	2.73182800
C	3.22852000	-1.96629000	1.04259600
C	2.28841800	1.32673900	-1.02078800
H	3.09722000	1.41220700	0.90275400
H	1.37431100	-0.92339600	3.32289600
H	2.61530300	0.34673600	3.16181900
C	2.66291300	-3.17844000	1.80549500
C	3.56707500	-2.36161300	-0.40650700
C	4.49973000	-1.43687300	1.73917700
C	0.86305400	1.85568700	-0.92356500
H	2.25398600	0.52713600	-1.76723300
C	3.31872500	2.36943000	-1.51503000
H	1.74167000	-3.54968300	1.34809800
H	3.40145000	-3.98684200	1.78226200
H	2.46147000	-2.95014900	2.85661000
H	4.35868200	-3.11821000	-0.39230500
H	2.70082300	-2.78621500	-0.91982100
H	3.93238200	-1.50483600	-0.98172800
H	5.27134300	-2.21291400	1.70218900
H	4.89235100	-0.54964000	1.23380600
H	4.32908300	-1.19751700	2.79277300
N	-0.13559300	1.19141000	-0.44636400
O	0.62336800	3.11893100	-1.29549000
C	4.62106000	1.66746600	-1.92408400
C	-1.22991600	2.17506900	-0.20837700
C	-0.82296100	3.31120300	-1.15834600
H	4.44432400	0.91483100	-2.69992700
H	5.33757900	2.39551600	-2.31555000
H	5.08018700	1.16779300	-1.06533600
H	-2.18878400	1.73641000	-0.48563400
C	-1.28591600	2.57797300	1.28446300
H	-0.98596800	4.31661300	-0.77305900
H	-1.26278200	3.21614300	-2.15485800
H	0.90978700	0.68649000	2.74600900
C	-2.02073800	-0.37695200	-2.70259300
H	-1.20857600	-0.22150100	-3.41708900
H	-2.92323000	-0.60063300	-3.27920400
H	-2.17930600	0.54639900	-2.15284000
C	-2.48038600	-3.00984200	0.18654700
H	-3.44406200	-3.37597700	-0.19158600
H	-1.71282500	-3.74617500	-0.04537700
H	-2.55578200	-2.92989500	1.27154700
C	-3.24224300	-0.65458800	0.09370200
C	-4.35636200	-0.32539000	-0.69596800
C	-3.24566300	-0.24050500	1.43568500
C	-5.40770200	0.43597700	-0.17872100
H	-4.42557200	-0.68547900	-1.71524900
C	-4.30031200	0.50312500	1.96021400
H	-2.40273200	-0.50449400	2.06505800
C	-5.38373900	0.85658800	1.15065500
H	-6.25147400	0.68368100	-0.81586700
H	-4.26934100	0.81800900	2.99884400
H	-6.20082700	1.44615900	1.55504200
C	-1.57178900	-2.87043800	-2.57729500
H	-1.03882400	-2.70045300	-3.51598600
H	-1.05036400	-3.64790700	-2.02160400
H	-2.58045400	-3.22643400	-2.81376100
H	2.87187800	2.82217100	-2.40720400
C	3.59255000	3.49321400	-0.50152700
H	4.20932000	4.26665200	-0.96796900
H	2.67077100	3.96320400	-0.15088400
H	4.15404500	3.12930400	0.36726400
C	-0.00875800	3.26743300	1.78713900
H	0.13909100	4.24474700	1.31546900
H	-0.07575100	3.43292600	2.86650400
H	0.88630100	2.66802300	1.60201400
H	-1.40830300	1.63934900	1.83621700
C	-2.51563800	3.45882500	1.54742600
H	-3.43586400	2.97302000	1.21356600
H	-2.61174900	3.66551900	2.61772400
H	-2.43143500	4.42419700	1.03536700

TSs for the migratory insertion pathway

I

N	0.26801400	1.49240400	-0.14983300
P	-2.24798600	-0.55533400	-0.44529900
Ir	0.01513700	-0.54372000	-1.15552200
H	-0.27810900	-1.90307600	-1.88548900
H	0.22080000	0.15398300	-2.67506900
C	1.84921200	-1.72870300	-0.17107700
C	2.15771900	-1.05982100	-1.44423500
H	-0.68068900	0.24059000	-2.48550600
H	0.52394900	-1.40183000	0.21332500
C	0.70228400	3.75487200	0.39530000
H	0.32769600	4.70949900	0.02649800
H	1.56976400	3.92502200	1.03691800
C	-0.43586200	1.87402100	0.86234600
O	-0.34194900	3.16008300	1.21995000
C	0.95862100	2.69705400	-0.70036800
H	2.02411200	2.48338700	-0.77563000
C	0.44276300	3.08116000	-2.10311600
C	-1.42361900	1.00889500	1.64073400
H	-0.89678800	0.08589300	1.89528800
C	-1.88670900	1.65326500	2.96753100
H	-2.26843900	2.65559900	2.73755400
C	-0.69792300	1.78842400	3.93483600
H	0.12313000	2.36873700	3.50767300
H	-1.01572700	2.28776800	4.85462800
H	-0.31584800	0.79823700	4.21157900
C	-3.00895200	0.83844700	3.62342500
H	-2.66381100	-0.17095800	3.87468100
H	-3.32002100	1.32219400	4.55423700
H	-3.88193600	0.74398400	2.97492600
N	-2.53607900	0.61315000	0.77475000
H	-3.17170800	1.36688100	0.53800700
C	-3.47195400	-0.07996200	-1.72411500
H	-3.50149500	-0.85284900	-2.49465900
H	-4.46945200	0.04005800	-1.29556200
H	-3.15820400	0.85811800	-2.18570700
C	-2.88401200	-2.17963100	0.26617600
C	-2.71842300	-3.29212000	-0.78586600
H	-1.66839000	-3.44678100	-1.04744900
H	-3.10536900	-4.23014800	-0.37306900

H	-3.27641800	-3.08378600	-1.70423900
C	-2.05289500	-2.52553400	1.51539300
H	-2.37543800	-3.49755200	1.90346400
H	-0.98501100	-2.59756400	1.28941100
H	-2.19631200	-1.78995600	2.31087200
C	-4.36521600	-2.03418400	0.66412800
H	-4.68578700	-2.94091800	1.18837900
H	-4.51529700	-1.18391400	1.33559300
H	-5.01604900	-1.91430100	-0.20661000
C	1.67796000	-3.25182800	-0.15267500
H	1.02540500	-3.60120000	-0.95097400
H	2.66189200	-3.71494000	-0.27519000
H	1.26776900	-3.57932500	0.80530200
C	2.40674600	-1.92027000	-2.67862800
H	3.40304000	-2.37954400	-2.61086000
H	1.67467200	-2.71541100	-2.81536700
H	2.39152600	-1.29584900	-3.57599700
C	2.57725200	-1.23207500	1.06607200
C	1.97975600	-0.48447200	2.08046000
C	3.93217700	-1.57980100	1.19162800
C	2.71111300	-0.07645200	3.19802100
H	0.94009300	-0.20954200	1.98935900
C	4.66454800	-1.17794800	2.30790600
H	4.41715800	-2.15787300	0.41043000
C	4.05740000	-0.42205700	3.31536100
H	2.22416700	0.51037800	3.97115000
H	5.71193700	-1.45280400	2.38845400
C	3.10977000	0.12768100	-1.40441100
H	4.14911000	-0.22727200	-1.41481200
H	2.96790100	0.75989300	-2.28470600
H	2.98391600	0.73179900	-0.50746800
H	4.63023700	-0.10657700	4.18195800
C	-1.06618600	3.35167200	-2.12489300
H	-1.32547500	4.24517500	-1.54609000
H	-1.41117300	3.51390100	-3.15010500
H	-1.62697300	2.51081800	-1.70812500
H	0.64981500	2.22096400	-2.74937800
C	1.24338300	4.26668900	-2.66140900
H	0.93732100	4.47850600	-3.69014100
H	1.07888900	5.17924900	-2.07776500
H	2.31818200	4.05632400	-2.66422200

J

N	1.45453200	-1.05471300	0.41276400
P	0.81357100	1.61566700	-1.03918800
Ir	-0.27343900	-0.50135800	-0.92366500
H	-1.45029800	-0.02039500	-1.85422800
H	0.21910800	-1.58020900	-2.10190400
C	-1.91773000	-1.20692500	0.69252300
C	-1.59776400	-2.18448800	-0.37333900
H	0.61832400	-0.74920200	-2.32386000
H	-1.23246000	-0.05617900	0.42453300
C	2.97101500	-2.22278500	1.75942600
H	4.03596800	-2.44486800	1.80903200
H	2.42363300	-2.86067100	2.45821500
C	1.84590700	-0.31605500	1.39657700
O	2.78186400	-0.83566700	2.19899600
C	2.36636000	-2.22903800	0.34492900
H	1.77865100	-3.12940300	0.16984300
C	3.38440000	-2.07108000	-0.80772100
C	1.32546900	1.09227000	1.66006200
H	0.24835500	0.97144200	1.81442000
C	1.89354400	1.74902300	2.93926600
N	1.51323800	1.95115100	0.48505600
H	2.42539900	2.39035400	0.45479300
C	2.24742300	1.62217900	-2.18758000
H	1.88782700	1.58752600	-3.21797500
H	2.86848700	2.51199100	-2.05635300
H	2.84702500	0.73276700	-1.99874700
C	-0.15557400	3.15452000	-1.50804100
C	-0.63609600	3.01315100	-2.96521400
H	-1.19779000	2.08490500	-3.11399900
H	-1.30211800	3.84948100	-3.20393100
H	0.19099500	3.03986700	-3.68045000
C	-1.37338700	3.24881900	-0.57722300
H	-1.91590000	4.17955600	-0.77598800
H	-2.05842700	2.41743100	-0.75434600
H	-1.07928200	3.25269300	0.47781100
C	0.72471200	4.40770100	-1.34247800
H	0.12904500	5.29678400	-1.57643200
H	1.08576900	4.50768000	-0.31433000
H	1.58582700	4.40595400	-2.01731700
C	-0.91340700	-3.48145800	0.03666500
H	-0.13662300	-3.35035200	0.78657700
H	-1.65891400	-4.17267200	0.45404200
H	-0.46686000	-3.96584200	-0.83575800
C	-1.39533100	-1.47641700	2.10956000
H	-1.98341700	-2.29351000	2.53863600
H	-0.34540800	-1.76076400	2.12543700
H	-1.53560900	-0.59528500	2.73984600
C	-3.31048100	-0.60174600	0.76966100
C	-3.51233800	0.77059300	0.94289600
C	-4.41583400	-1.46130700	0.84213800
C	-4.79475500	1.28701300	1.13142500
H	-2.66051800	1.43937000	0.93601300
C	-5.69924600	-0.94819900	1.03594400
H	-4.27648900	-2.53345100	0.75533200
C	-5.89495900	0.42819500	1.17065700
H	-4.93020200	2.35776100	1.25087200
H	-6.54560900	-1.62694000	1.08141000
H	-6.89482100	0.82647100	1.31378600
C	-2.62185200	-2.44623700	-1.47710800
H	-3.33396900	-3.22200900	-1.16722400
H	-3.18771900	-1.55521800	-1.74889200
H	-2.11144100	-2.81354100	-2.37089600
C	3.37781400	2.13533600	2.82429700
H	3.75217300	2.45167200	3.80210900
H	3.99388900	1.30113900	2.48059400
H	3.52312200	2.98307500	2.14439100
C	1.03731300	2.96287800	3.32565800
H	1.06329300	3.72009300	2.53602000
H	-0.00796400	2.68017200	3.49032500
H	1.41421900	3.41700100	4.24671100
H	1.80182800	0.99131900	3.72618300
C	4.08609800	-3.40912700	-1.08034200
H	4.78095100	-3.31350400	-1.91991100
H	4.66572900	-3.73764500	-0.20962600
H	3.36549600	-4.19693100	-1.32334800
C	4.41017800	-0.95367200	-0.56331300
H	5.11617600	-1.22477300	0.22821900
H	4.99346500	-0.77315600	-1.47096300
H	3.93911800	-0.00911800	-0.27779400
H	2.79105300	-1.80988400	-1.69346400

K

N	-1.26149000	-1.43414400	-0.34339300
P	-0.87125600	1.50057400	0.67992800
Ir	0.42343400	0.10686500	-0.72359000
H	1.37720200	-0.44018700	0.57364500
C	2.06616200	-1.11801900	-1.56067400
C	2.40217500	-1.09147400	-0.12388000
C	-2.62811100	-3.33952500	-0.25512000
H	-3.12298600	-3.98328300	0.47085700
H	-2.62957400	-3.81506900	-1.23955500
C	-2.50679100	-1.11174400	-0.46859900
O	-3.40162400	-2.10348500	-0.35952800
C	-1.24012600	-2.83493100	0.16301500
H	-0.45970100	-3.40218000	-0.34357300
C	-0.97736700	-2.84742300	1.68941700
C	-3.02751800	0.31082700	-0.62949400
H	-2.59975900	0.67959500	-1.56650700
C	-4.56514500	0.41710400	-0.77181600
N	-2.52972300	1.15821500	0.45973400
H	-3.09550900	1.12065000	1.29816000
C	-0.60907100	1.24636700	2.47856000
H	0.37112900	1.63638800	2.76092900
H	-1.37512400	1.74918800	3.07425000
H	-0.62711500	0.17596200	2.68555800
C	-0.71144100	3.35123500	0.40508000
C	0.73364300	3.79062200	0.70553600
H	1.45833400	3.25372000	0.08654600
H	0.83544800	4.85956400	0.48836100
H	1.00094800	3.64630200	1.75746200
C	-1.04812900	3.62903400	-1.07229200
H	-1.01661900	4.70872600	-1.25410900
H	-0.32600700	3.15516800	-1.74357800
H	-2.05268100	3.27664500	-1.32787100
C	-1.70153300	4.10573200	1.31276400
H	-1.65137100	5.17664800	1.08795600
H	-2.73069200	3.77519500	1.14321400
H	-1.46422800	3.98375100	2.37390200
H	-0.02551200	0.14801700	-2.34682900
H	-0.53074800	0.80972700	-1.92990200
H	1.42319000	1.28557600	-0.97073800
C	2.35827900	-2.41042200	0.65282300
H	1.48896200	-3.01422600	0.41079600
H	3.24736300	-2.98759700	0.38113800
H	2.38413800	-2.23480400	1.73010500
C	3.57691800	-0.23596500	0.32838100
C	3.44075700	0.94253200	1.06721900
C	4.86717700	-0.69805600	0.02507900
C	4.56628000	1.65529500	1.48680300
H	2.45122200	1.31462200	1.30523100
C	5.99176700	0.01411100	0.44079400
H	4.99422100	-1.61162000	-0.54860500
C	5.84566600	1.19524500	1.17339000
H	4.43771600	2.57100900	2.05623100
H	6.98206600	-0.35360600	0.18932500
C	1.64127100	-2.43740900	-2.19235400
H	0.83979900	-2.94368700	-1.65856500
H	1.28902700	-2.25784500	-3.21124900
H	2.49596400	-3.12533700	-2.25113700
H	6.72137100	1.75035600	1.49558300
C	2.94700500	-0.31871500	-2.51851100
H	3.27491100	0.63258800	-2.10036200
H	3.84251600	-0.90196700	-2.77328600
H	2.40564800	-0.11956200	-3.44682700
C	-2.12725500	-2.25048200	2.51556000
H	-2.40686400	-1.25038900	2.17387700
H	-3.02201200	-2.88031200	2.48242800
H	-1.82606800	-2.16776100	3.56403800
C	-0.64636100	-4.27037400	2.15975900
H	0.22024300	-4.68114100	1.63139900
H	-0.42392300	-4.27803800	3.23070600
H	-1.49177800	-4.94791100	1.99343300
H	-0.09618000	-2.21445300	1.84557800
C	-4.94335800	1.80074600	-1.31924400
H	-4.63786300	2.58974700	-0.62475400
H	-4.46655400	1.99520700	-2.28598900
H	-6.02634100	1.87169300	-1.45732200
H	-4.84702200	-0.33960100	-1.51290500
C	-5.32991400	0.11628900	0.52809100
H	-6.40252200	0.06903400	0.31919000
H	-5.02965500	-0.83627600	0.97013200
H	-5.19094400	0.91034700	1.27129500

L

N	0.23744900	1.43950700	-0.71930000
P	1.67026300	-0.89404500	0.91787300
Ir	0.00253900	-0.90644400	-0.75165600
H	-1.09247400	-1.18923700	0.49263700
C	-2.02175100	-1.22219100	-1.65761200
C	-2.27802600	-1.51149400	-0.23848900
C	0.10689000	3.75526800	-1.21937400
H	-0.11862500	4.64367000	-0.62881600
H	0.10507900	4.01147300	-2.28143700
C	1.40112200	1.99587900	-0.67779100
O	1.45464700	3.32083800	-0.87522300
C	-0.76615900	2.52778600	-0.88861200
H	-1.39813400	2.28435600	-1.74127000
C	-1.64675300	2.66006100	0.36710300
C	2.73028800	1.28469400	-0.43763700
H	2.85254400	0.61446900	-1.29570800
C	3.95775300	2.22886300	-0.42906200
N	2.69460500	0.45874300	0.76737300
H	2.84965500	0.98074000	1.62093800
C	1.00982100	-0.68935800	2.61768400
H	0.41973200	-1.56520900	2.89419000
H	1.81350400	-0.55325800	3.34577200
H	0.36433200	0.19133400	2.62645800
C	2.81049900	-2.38227700	0.98573600
C	1.98041800	-3.64267100	1.28886100
H	1.18637800	-3.79234000	0.55148100
H	2.63610200	-4.51977400	1.26024900
H	1.52565500	-3.60549600	2.28382300
C	3.48176600	-2.50661900	-0.39528000
H	4.17529600	-3.35443900	-0.38656000

H	2.74516100	-2.68096600	-1.18494600
H	4.05689800	-1.60823500	-0.64249200
C	3.88853300	-2.17694900	2.06710200
H	4.59637400	-3.01217300	2.03070800
H	4.44939200	-1.25231100	1.90115600
H	3.46437800	-2.15324200	3.07548300
H	0.47732300	-0.75697400	-2.36118500
H	1.27435600	-0.79316500	-1.85081700
H	0.13084600	-2.45861700	-0.86607700
C	-2.41786500	-2.97276400	0.20357900
C	-3.20212100	-0.63653100	0.59160800
C	-2.83878200	-0.19426400	1.87030400
C	-4.50299600	-0.38329100	0.13592900
C	-3.73312600	0.52784000	2.65949400
H	-1.83769200	-0.39280300	2.23951500
C	-5.40047500	0.33975100	0.92411800
H	-4.81937200	-0.75227400	-0.83355500
C	-5.01630700	0.80611200	2.18293900
H	-3.42456000	0.87673400	3.64008600
H	-6.40047200	0.53793300	0.55062300
C	-2.03880300	-2.38321700	-2.64941700
H	-1.46735300	-3.25165300	-2.32379300
H	-3.07541600	-2.70226300	-2.82800600
H	-1.62440400	-2.05351300	-3.60569600
H	-3.41926800	-3.31311100	-0.08096700
H	-1.67807100	-3.62494600	-0.25614200
H	-2.33040500	-3.05321700	1.28933600
C	-2.62494200	0.02565300	-2.28848500
H	-3.60613300	-0.20725800	-2.72276700
H	-2.77136400	0.83223600	-1.57427400
H	-1.98934800	0.38158100	-3.10296000
H	-5.71331100	1.37498600	2.79044700
C	4.01901300	3.13752000	0.81002300
H	4.82492200	3.86787900	0.69250800
H	3.08652200	3.68666900	0.96117900
H	4.24377900	2.56627800	1.71858600
H	3.85136800	2.86815300	-1.31202100
C	5.24900200	1.41192900	-0.57490100
H	5.37776100	0.73122800	0.27222900
H	5.24471400	0.81475700	-1.49322500
H	6.11649100	2.07770700	-0.61012400
C	-0.85343100	3.03974500	1.62331800
H	-0.47253900	4.06608900	1.57443400
H	-1.49654400	2.97060200	2.50574600
H	-0.00142300	2.37064900	1.77702100
H	-2.06574100	1.66641600	0.53384200
C	-2.81439300	3.62334600	0.11948200
H	-3.48592800	3.63095600	0.98316700
H	-2.46581100	4.65037200	-0.04092100
H	-3.40022200	3.32472300	-0.75686800

M

N	-0.88168500	1.48854100	0.24909300
P	-1.33263000	-1.37682400	-1.04813700
Ir	0.56449400	-0.11945200	-0.39722100
H	1.52945000	-1.25779700	-0.89470800
H	1.18255600	0.91013600	-1.57152100
C	1.61530400	-0.40304200	1.72937600
C	2.26172700	0.58945200	0.84572100
H	0.49043600	0.41525700	-1.98832800
H	0.50124100	-0.93106800	1.07595600
C	-1.92281000	3.49713100	0.86710400
H	-2.58610800	4.24381300	0.43256500
H	-1.49978300	3.87344000	1.80228000
C	-1.99750600	1.25493600	0.85361300
O	-2.73077800	2.32084800	1.19496000
C	-0.85513700	2.93884900	-0.09149000
H	0.12844500	3.34688700	0.13771900
C	-1.14197000	3.16641400	-1.59360500
C	-2.54676500	-0.13842700	1.13242500
H	-1.78136800	-0.63382700	1.73796000
C	-3.85973800	-0.14597600	1.94970800
H	-3.70263400	0.56098400	2.77245500
C	-4.10243900	-1.54036300	2.54259200
H	-3.27670400	-1.84966100	3.19240400
H	-5.02022000	-1.54667500	3.13795600
H	-4.20889100	-2.28528000	1.74776900
C	-5.08046700	0.32657100	1.14206600
H	-5.37278700	-0.41543300	0.39001200
H	-5.93780700	0.45270200	1.80934100
H	-4.89812600	1.28137600	0.64283900
N	-2.68374200	-0.89631300	-0.11587000
H	-3.51923000	-0.66914000	-0.64073000
C	-1.91287900	-1.12380300	-2.76903200
H	-1.20228900	-1.58860400	-3.45598700
H	-2.90105500	-1.56258100	-2.92635700
H	-1.94964100	-0.05327600	-2.97637400
C	-1.22760700	-3.24041500	-0.82887500
C	-0.05671000	-3.78586300	-1.66591400
H	0.89353300	-3.33618500	-1.36484900
H	0.01701400	-4.86849900	-1.51581600
H	-0.19773400	-3.61252900	-2.73763000
C	-0.98150900	-3.52696100	0.66322500
H	-0.92532200	-4.60963000	0.81914400
H	-0.03673300	-3.09502200	1.00213000
H	-1.79210500	-3.14108100	1.28843200
C	-2.55337500	-3.89475500	-1.26391200
H	-2.51737000	-4.96515600	-1.03420300
H	-3.40634600	-3.46518900	-0.72942400
H	-2.72745200	-3.79740100	-2.33935300
C	2.31906600	-1.73950700	1.95967600
H	2.76632700	-2.12307700	1.04345200
H	3.11418000	-1.58999900	2.69614500
H	1.62677400	-2.48272600	2.36228100
C	2.17991500	2.05997300	1.25785900
H	2.90076000	2.26916100	2.05846600
H	2.43716400	2.69928300	0.41008500
H	1.19512000	2.34637100	1.62186800
C	-2.54079600	2.70253700	-2.02141200
H	-3.32759900	3.32007800	-1.57513700
H	-2.64579700	2.77589300	-3.10784000
H	-2.72902000	1.66343800	-1.73837400
C	-0.90375300	4.63866300	-1.95811500
H	-1.04973300	4.79388600	-3.03110300
H	-1.60205900	5.30026100	-1.43286200
H	0.11416400	4.95322400	-1.70516800
H	-0.40325900	2.56380800	-2.13487600
C	3.66650700	0.27630500	0.35457300
C	4.01789700	0.08828400	-0.98760900
C	4.70010000	0.26337600	1.31000500
C	5.34766400	-0.12606500	-1.36285700
H	3.24665200	0.09624400	-1.74769600
C	6.02661500	0.04230400	0.94252800
H	4.46166000	0.42821000	2.35744300
C	6.35702000	-0.15623500	-0.40059800
H	5.58930900	-0.27172100	-2.41186800
H	6.80091900	0.02851700	1.70421100
H	7.38894300	-0.32801600	-0.69204700
C	0.86910000	0.08816500	2.96842900
H	0.17753500	0.89974900	2.74617400
H	0.31201900	-0.72871000	3.43460900
H	1.60589900	0.45410100	3.69153000

N

N	0.50642900	1.17935200	-0.02355600
P	-2.24810400	-0.18803700	-0.99228400
Ir	-0.03834400	-0.95752400	-0.54174400
H	-0.48486300	-2.41969500	-0.90938800
H	0.99670700	-1.06336600	-1.81075500
C	0.61935000	-1.67606700	1.65563100
C	1.62760200	-1.89981600	0.58192200
H	0.11917100	-0.67232000	-2.15588800
H	-0.49837300	-1.24095900	1.08680600
C	1.50556800	3.30035500	0.27101200
H	1.64001800	4.16740500	-0.37542200
H	2.10572000	3.42136000	1.17559300
C	-0.31168400	2.00442700	0.53530700
O	0.10365100	3.26733800	0.67975200
C	1.72476200	1.93801300	-0.42320000
H	2.59457500	1.42943000	-0.00690900
C	1.88240300	2.00890400	-1.95597200
C	-1.72799900	1.66224900	0.98301500
H	-1.64188800	0.76999500	1.60980800
C	-2.38805500	2.76549200	1.84105200
H	-2.32223100	3.70815700	1.28286900
C	-1.63450900	2.92751500	3.17208500
H	-0.57725400	3.15796900	3.02529700
H	-2.07634800	3.73933100	3.75701200
H	-1.70917800	2.01032200	3.76843800
C	-3.86604700	2.44956800	2.10604400
H	-3.97035100	1.49601400	2.63644400
H	-4.30414300	3.22980100	2.73577100
H	-4.44743900	2.38661700	1.18400100
N	-2.53884100	1.28748400	-0.17856500
H	-2.83734600	2.06982600	-0.74920500
C	-2.60638300	0.24178300	-2.73719600
H	-2.61921800	-0.67136000	-3.33563900
H	-3.57138300	0.74573600	-2.82909700
H	-1.81715800	0.89419800	-3.11349500
C	-3.64609900	-1.33167900	-0.46290900
C	-3.52968200	-2.66500300	-1.22411400
H	-2.57447900	-3.16028000	-1.02835400
H	-4.33163700	-3.33414600	-0.89354000
H	-3.63726600	-2.53533600	-2.30547700
C	-3.50734200	-1.58333900	1.04966200
H	-4.31820000	-2.23994900	1.38256300
H	-2.56236500	-2.07695900	1.29289000
H	-3.57878400	-0.65387000	1.62191700
C	-5.00596700	-0.66672700	-0.74894100
H	-5.80452200	-1.30057500	-0.34843600
H	-5.07939800	0.31309800	-0.26856400
H	-5.19083400	-0.54845100	-1.82054000
C	0.79890800	-0.55072100	2.67944900
H	1.38136700	-0.92735000	3.52447200
H	1.30201400	0.32007600	2.26436400
H	-0.17203200	-0.24463100	3.07791900
C	1.87464600	-3.35303600	0.15537300
H	2.49468300	-3.85868700	0.90794000
H	0.95757200	-3.92612100	0.02139400
H	2.42061100	-3.37459700	-0.78965500
C	2.95048300	-1.16041700	0.56443300
C	3.57107500	-0.86543400	-0.66303900
C	3.68594300	-0.90575300	1.73270300
C	4.83798300	-0.28971900	-0.72381800
H	3.04280300	-1.08047200	-1.58663400
C	4.95011700	-0.31018100	1.68024400
H	3.28788000	-1.18866600	2.69857400
C	5.52958300	0.01059200	0.45360400
H	5.28059900	-0.06930200	-1.69056600
H	5.48405300	-0.11193800	2.60506700
H	6.51065800	0.47368800	0.41188700
C	-0.01235100	-2.91464100	2.30278200
H	-0.80806100	-2.62209700	2.99265200
H	-0.42679500	-3.60471600	1.56945900
H	0.76199300	-3.43650700	2.87434700
C	0.68951600	2.68281900	-2.64437900
H	0.62609900	3.74819900	-2.39626100
H	0.78315000	2.60612300	-3.73159100
H	-0.25417100	2.21515500	-2.35256300
H	1.93502300	0.97069500	-2.30126100
C	3.20881200	2.68821000	-2.32408200
H	4.05540200	2.19052600	-1.84148500
H	3.36516300	2.65512300	-3.40650600
H	3.21833400	3.74246500	-2.02491100

O

N	-0.24270100	1.19892000	0.03217200
P	2.13086000	-0.72091500	0.54767900
Ir	-0.04534800	-1.05567200	-0.31739100
H	-0.64148800	-1.51214300	1.24166700
C	-2.17172500	-1.61220900	-0.46114900
C	-1.88411300	-1.91205800	0.97166200
C	-1.10354100	3.37354100	0.02658300
H	-1.21142300	4.24219700	0.67447800
H	-1.73747600	3.48049500	-0.85776800
C	0.62567800	2.02819000	-0.44298000
O	0.28889700	3.32486000	-0.42871800
C	-1.28287600	2.01598400	0.71601600
H	-2.26691200	1.60249600	0.50261900
C	-1.03477500	2.02565600	2.24420900
C	2.01259800	1.64289800	-0.94318400
H	1.84697300	1.03993100	-1.84221700
C	2.88582900	2.84565800	-1.37561300
N	2.70430400	0.80685500	0.04505600
H	3.20555400	1.33960200	0.74493600
C	2.20641600	-0.63496500	2.37846500
H	2.01683000	-1.62679800	2.79423800

H	3.17918200	-0.27857800	2.72730600
H	1.42396000	0.04395100	2.72092400
C	3.45319800	-1.94459300	0.02923600
C	3.08040600	-3.34106300	0.55988100
H	2.08780800	-3.65090000	0.21846800
H	3.80735700	-4.07204400	0.18940800
H	3.10004400	-3.38484000	1.65337900
C	3.49411000	-1.95685100	-1.51058300
H	4.28323600	-2.63847900	-1.84601100
H	2.54749500	-2.30361500	-1.93529300
H	3.71779900	-0.96338800	-1.91273000
C	4.82332000	-1.50513700	0.57925500
H	5.59513900	-2.19114100	0.21396100
H	5.08244900	-0.49710200	0.24128600
H	4.85605100	-1.52812100	1.67271200
H	-0.23536600	-0.85196100	-1.94410100
H	0.73628500	-0.73094100	-1.73840300
H	0.25328600	-2.56600900	-0.61975500
C	-2.57761000	-1.10755800	2.07499600
H	-2.77627300	-0.07984500	1.78695800
H	-3.53697400	-1.58517900	2.29489600
H	-1.98654000	-1.11983300	2.99397500
C	-2.41862500	-2.80184300	-1.39811100
H	-1.68257500	-3.59800900	-1.28897200
H	-3.41629000	-3.22183500	-1.21222000
H	-2.39133800	-2.45931100	-2.43499900
C	0.25482000	2.74851300	2.66064700
H	1.13418700	2.34659700	2.15012500
H	0.20708900	3.82283300	2.45544100
H	0.41662000	2.62838100	3.73596600
C	-3.14564800	-0.50292900	-0.81508800
C	-2.84088700	0.49020300	-1.75922900
C	-4.45324900	-0.52980700	-0.29941200
C	-3.78203400	1.45170000	-2.13175800
H	-1.84450000	0.53195100	-2.18366500
C	-5.39623400	0.43357400	-0.66162000
H	-4.74238100	-1.31637700	0.38963200
C	-5.06196100	1.43618900	-1.57399500
H	-3.51005600	2.21457200	-2.85567400
H	-6.39379300	0.39457900	-0.23383400
H	-5.79237100	2.18918700	-1.85407400
C	-1.75526600	-3.38184300	1.38334200
H	-1.08846000	-3.94532900	0.73378200
H	-1.38387200	-3.45814900	2.40874500
H	-2.75073200	-3.83683100	1.34593700
C	-2.25370800	2.61037300	2.97121100
H	-3.17073700	2.06355800	2.72793200
H	-2.11125800	2.56829100	4.05499600
H	-2.40996200	3.66084600	2.70009800
H	-0.92748100	0.97466000	2.53608200
C	3.34719000	3.72687300	-0.20281100
H	3.82199500	4.63308400	-0.58959500
H	2.51454500	4.02965300	0.43652600
H	4.09854600	3.21954300	0.41370300
C	4.08523900	2.35239300	-2.19685400
H	4.72318000	1.69618100	-1.59668700
H	3.76289800	1.79481300	-3.08281600
H	4.69010000	3.19986800	-2.53274400
H	2.24680000	3.45530700	-2.02420000

P

N	1.10171200	1.34269100	-0.60284300
P	1.22796600	-1.35743700	0.96825600
Ir	-0.55447700	-0.17856800	-0.04391600
H	-0.94570200	0.59164100	1.43338600
C	-2.43564400	0.93896900	-0.30642700
C	-2.16472600	1.13050900	1.14108000
C	2.32269800	3.18547900	-1.38681400
H	3.03616500	3.93056000	-1.03751900
H	1.92363800	3.47799300	-2.36171100
C	2.21088500	0.95990300	-1.14465400
O	3.04135600	1.92414700	-1.56522000
C	1.22443900	2.81093600	-0.38247800
H	0.28864500	3.30536500	-0.64191600
C	1.56302100	3.10482300	1.09988500
C	2.67482500	-0.48795100	-1.26019000
H	1.93857200	-0.98544200	-1.89961400
C	4.04963100	-0.66338700	-1.94993900
N	2.65180200	-1.13992400	0.05393600
H	3.50974600	-1.02968900	0.57957300
C	1.69300600	-0.78378000	2.64776500
H	0.89665600	-1.04048400	3.34942600
H	2.62906000	-1.23719300	2.98426400
H	1.80154000	0.30078100	2.62584800
C	1.00474900	-3.21660000	1.10962600
C	-0.17068700	-3.51813300	2.05761500
H	-1.09228800	-3.02679300	1.73082900
H	-0.35235100	-4.59841900	2.06979800
H	0.04015000	-3.21078600	3.08647100
C	0.69393500	-3.75565400	-0.30008400
H	0.61242300	-4.84722800	-0.25960800
H	-0.25356300	-3.36233900	-0.67992000
H	1.48905200	-3.50726500	-1.01090400
C	2.29911900	-3.86445400	1.63635700
H	2.17774400	-4.95301500	1.64856200
H	3.15256100	-3.62857000	0.99365500
H	2.53243900	-3.55074900	2.65833000
H	-0.76000000	-0.47280700	-1.67242500
H	-0.09153000	-1.09397200	-1.36301100
H	-1.52532900	-1.35149300	0.31356600
C	-2.98385200	0.32100300	2.15051600
H	-3.94446100	0.81985900	2.30707600
H	-3.17456900	-0.69189200	1.79869400
H	-2.46442600	0.27219400	3.11125000
C	-2.32050600	2.14369400	-1.24350900
H	-3.14321400	2.85051000	-1.07461300
H	-1.38421300	2.68654800	-1.13542800
H	-2.38429900	1.80120800	-2.27873900
C	-3.68058200	0.14511200	-0.66957300
C	-3.68161600	-1.05712600	-1.38740100
C	-4.92498900	0.71548500	-0.34303800
C	-4.87959900	-1.68173100	-1.74639700
H	-2.74065100	-1.51853400	-1.66136200
C	-6.12272200	0.09361900	-0.69339400
H	-4.95559600	1.66123300	0.19187500
C	-6.10474600	-1.11383700	-1.39645000
H	-4.84961100	-2.61589300	-2.30006100
H	-7.06804800	0.55347300	-0.41994900
H	-7.03483100	-1.60227800	-1.67150300
C	-1.90025200	2.54810400	1.64265800
H	-1.47559700	2.54221700	2.64928700
H	-1.24710100	3.11912800	0.98906400
H	-2.86534200	3.06485400	1.68383700
C	5.23547900	-0.17329900	-1.10168700
H	6.15122500	-0.20596600	-1.69892200
H	5.09582200	0.85214000	-0.75280300
H	5.40610200	-0.82077800	-0.23347000
H	4.00684200	-0.04951200	-2.85677000
C	4.24217500	-2.12983600	-2.36191300
H	4.24776400	-2.78304500	-1.48355500
H	3.44333300	-2.46719600	-3.03095000
H	5.19526600	-2.25554300	-2.88404000
C	2.96728700	2.64016700	1.51434600
H	3.13938200	1.58596100	1.28260200
H	3.75186200	3.22523000	1.02396400
H	3.09510300	2.76537500	2.59368100
H	0.83770500	2.53172100	1.68960300
C	1.36598000	4.59612100	1.40408400
H	0.34552200	4.92324200	1.17903300
H	1.56076100	4.80072400	2.46081400
H	2.05357500	5.21481000	0.81617500

Ir/4c with S83

TSs for the σ-methatesis pathway

A

N	-1.27864900	0.62980500	-1.22992600
P	-1.56791200	-1.45532000	1.25690000
Ir	0.31516500	-0.88390800	-0.05459500
H	0.29906300	-1.56925600	-1.57441600
C	2.32837300	0.22206200	0.72130300
C	2.33462300	-0.22135900	-0.69627200
C	-2.04309000	1.58510600	-3.24606500
H	-1.55746900	1.09650800	-4.09567900
H	-2.60274400	2.45261200	-3.59177300
C	-2.40817100	0.11832900	-1.59255600
O	-2.99765800	0.63988400	-2.68077000
C	-1.07841100	1.84678100	-2.07727000
C	-3.17748800	-1.01019400	-0.91092500
N	-3.04866600	-0.96007200	0.54657300
H	-3.37347300	-0.07321800	0.91579500
C	-1.75851100	-3.27224100	1.39028000
H	-0.88183300	-3.68946200	1.89180300
H	-2.67036700	-3.54145700	1.92540200
H	-1.79754000	-3.68615700	0.37970600
C	-1.70538200	-0.76824800	2.99746500
C	-0.43425700	-1.17864000	3.76551600
H	0.47166800	-0.80194200	3.28441000
H	-0.47699800	-0.76487800	4.77827300
H	-0.34183900	-2.26517100	3.85483700
C	-1.79581600	0.76886300	2.91236300
H	-1.67271800	1.19356500	3.91398100
H	-1.02234700	1.19200900	2.26808800
H	-2.76757000	1.11027700	2.54085700
C	-2.95315900	-1.32378300	3.71256900
H	-3.05236300	-0.82920000	4.68478500
H	-3.86828200	-1.13725700	3.14383100
H	-2.87467800	-2.39824100	3.89748200
H	1.04958700	0.01985600	1.20074500
H	1.06389400	-2.10766700	0.54633100
C	-4.67266300	-1.07104500	-1.32422800
H	-4.69053000	-1.05456900	-2.41878500
C	-5.47236200	0.13583200	-0.81126000
H	-6.49628200	0.09549800	-1.19406500
H	-5.53697900	0.13372400	0.28268300
H	-5.03119200	1.08276800	-1.13415000
C	-5.29060200	-2.39168500	-0.84717100
H	-6.33560600	-2.45692900	-1.16459400
H	-4.75815500	-3.25561500	-1.26033500
H	-5.25788000	-2.46334100	0.24387700
H	-2.72099800	-1.94534400	-1.25875100
C	-1.35809500	3.16004000	-1.28287500
C	-2.81217000	3.21391200	-0.77916100
H	-3.53966700	3.17572600	-1.59591700
H	-3.02221500	2.38708000	-0.09357200
H	-2.98170800	4.14570800	-0.23109700
C	-1.07861600	4.35888900	-2.20996500
H	-1.19367500	5.29422900	-1.65304400
H	-0.05514300	4.32475000	-2.60003600
H	-1.76516200	4.40056500	-3.06179200
H	-0.05186600	1.87189500	-2.43538400
C	-0.41004100	3.23264200	-0.07799000
H	-0.51348700	2.34970500	0.55293100
H	0.63668600	3.30171500	-0.38621100
H	-0.63035700	4.11485000	0.53098100
C	3.09321100	-0.64026900	1.73658400
H	3.02135300	-1.70261900	1.50666000
H	4.14565400	-0.34560900	1.71117700
H	2.72320000	-0.46184800	2.74846100
H	-0.44307100	-1.95189900	-1.09491500
C	2.24785100	0.81939500	-1.81263700
H	3.24371600	1.16763800	-2.10896200
H	1.79040700	0.37360000	-2.69890400
H	1.66744000	1.68959200	-1.51309000
C	2.43241800	1.68703700	1.11260600
C	3.36174200	2.52803300	0.48669500
C	1.72501800	2.17753300	2.21851900
C	3.53725100	3.84275100	0.92494300
H	3.95931400	2.16442800	-0.33965200
C	1.89517100	3.48888400	2.65616600
H	1.02860300	1.53024000	2.74132600
C	2.79720200	4.33172900	2.00201500
H	4.25611700	4.48156800	0.42105700
H	1.32332700	3.85078700	3.50505400
H	2.92847200	5.35647600	2.33548700
C	3.33817000	-1.30199100	-1.06595400
C	3.00737500	-2.57485700	-1.54523500
C	4.70222100	-0.96178600	-0.99716800
C	3.99991300	-3.48102200	-1.93142700
H	1.96796200	-2.87258100	-1.60876700
C	5.69610200	-1.86320800	-1.37385400
H	4.98937200	0.02413400	-0.64157400
C	5.34730300	-3.13234600	-1.84354700
H	3.71310800	-4.46251100	-2.29782400
H	6.74046300	-1.57350100	-1.30289200
H	6.11755100	-3.83856100	-2.13865100

B

N	-1.14100700	1.05336500	0.32431700
P	-1.44140500	-2.03370300	-0.55887100
Ir	0.35863300	-0.57438500	-0.47901600
H	0.94393900	0.48317300	-1.62622500
C	2.43323400	0.06643700	0.24702300
C	2.25791700	0.80251700	-1.00042300
C	-1.91611000	3.26177400	0.53514600
H	-1.37990900	3.89429900	-0.17474300
H	-2.51735600	3.87694200	1.20077400
C	-2.24286900	1.22261100	-0.32785000
O	-2.83355300	2.41962300	-0.22787100
C	-1.00184700	2.23919300	1.23039900
C	-2.94938200	0.20104900	-1.20118800
N	-2.87232800	-1.12111500	-0.55785300
H	-3.70213500	-1.67590300	-0.73523300
C	-1.37685600	-3.00037600	-2.11824800
H	-0.41267900	-3.50667500	-2.19442000
H	-2.18502700	-3.73269900	-2.17502600
H	-1.46482500	-2.29485800	-2.94702700
C	-1.69763600	-3.28927600	0.80914200
C	-0.57477600	-4.33971100	0.71112700
H	0.41538800	-3.87704500	0.74436400
H	-0.65341400	-5.02259000	1.56324800
H	-0.64721600	-4.93918900	-0.20063300
C	-1.65436900	-2.59063800	2.17797000
H	-1.92595900	-3.31401000	2.95397100
H	-0.65489100	-2.21508500	2.40528600
H	-2.35861400	-1.75614200	2.22816600
C	-3.07486200	-3.96665500	0.63339600
H	-3.16653100	-4.76188800	1.38026200
H	-3.89712200	-3.26597300	0.80646300
H	-3.20246600	-4.42975700	-0.34895700
H	0.50777700	-1.26830400	0.97799100
H	1.20091300	-1.72024900	-1.12752900
C	-4.42402200	0.57419000	-1.50531300
H	-4.39427500	1.60363400	-1.87551200
C	-5.32784300	0.53789100	-0.26364200
H	-6.33124500	0.88474200	-0.52740400
H	-5.42728200	-0.47521300	0.13964300
H	-4.94409600	1.18201300	0.53024200
C	-4.97972000	-0.30931600	-2.63320900
H	-5.98421500	0.02518800	-2.90726800
H	-4.34985400	-0.26252800	-3.52755100
H	-5.06318900	-1.36131200	-2.33623200
H	-2.41596000	0.21465900	-2.16429500
C	-1.42612600	1.92223700	2.70275600
C	-2.89071800	1.44643500	2.76759500
H	-3.58983900	2.19775300	2.38805600
H	-3.03583100	0.52526900	2.19565900
H	-3.16596600	1.23993700	3.80620600
C	-1.25829600	3.20576700	3.54202000
H	-1.44948700	2.98343700	4.59657900
H	-0.23853900	3.59913700	3.46296800
H	-1.95235000	3.99903900	3.24757700
H	0.03221600	2.58444600	1.21301700
C	-0.52049300	0.83288700	3.30337600
H	-0.44448900	-0.03108200	2.64346800
H	0.48558800	1.21481000	3.48868300
H	-0.92602500	0.49925800	4.26402300
H	-0.30340000	-0.26758300	-1.95767400
C	3.00958200	0.34039000	-2.25779200
H	4.01607500	0.76656600	-2.24040700
H	3.08679300	-0.74569300	-2.30203100
H	2.50868700	0.70397200	-3.15798900
C	2.08542600	2.30921800	-0.96468400
C	0.98017000	2.96214400	-1.52251100
C	3.14865400	3.08113400	-0.47188100
C	0.92711900	4.35703500	-1.56857200
H	0.15222200	2.38002700	-1.91487200
C	3.09281000	4.47403100	-0.51045800
H	4.02767800	2.59350900	-0.06236900
C	1.97910300	5.11731400	-1.05550900
H	0.06345600	4.84702600	-2.00825100
H	3.92185700	5.05482900	-0.11812300
C	2.39643900	0.81252800	1.57232500
H	3.38060200	1.25273300	1.76807100
H	1.66225500	1.61287200	1.58403700
H	2.17829800	0.11554300	2.38024300
H	1.93540900	6.20149700	-1.08672900
C	3.46411400	-1.04431100	0.30861600
C	3.18261200	-2.35563700	0.70971000
C	4.80759200	-0.69143100	0.09341800
C	4.20348300	-3.29696800	0.85441900
H	2.15565100	-2.63445300	0.90382100
C	5.82989100	-1.63040200	0.23192700
H	5.06149800	0.32918100	-0.17777400
C	5.53057500	-2.94170000	0.60782000
H	3.95665900	-4.30943200	1.16051000
H	6.85873300	-1.33408800	0.05017400
H	6.32384900	-3.67544200	0.71434700

C

N	0.75476800	1.11111400	0.59457300
P	1.89380400	-1.58741000	-0.72908700
Ir	-0.19927200	-0.59550600	-0.62205600
H	0.01270700	-1.52811600	0.69420200
C	-2.53105100	0.12526200	-0.56730300
C	-2.12816400	-0.67200200	0.58723300
C	0.66364600	2.79276000	2.22594000
H	-0.21300400	2.63682700	2.86041600
H	1.12122300	3.74980400	2.46663400
C	1.52514700	0.82637300	1.58856200
O	1.63405400	1.74909900	2.55339400
C	0.38521900	2.55564900	0.73076800
C	2.30512000	-0.45999100	1.78511400
N	2.85423900	-0.90928700	0.49455300
H	3.74925100	-1.37094800	0.60761500
C	1.76470800	-3.39624400	-0.43850500
H	1.05958700	-3.83025200	-1.15052700
H	2.73375400	-3.89079800	-0.52968000
H	1.37890700	-3.54720600	0.57173500
C	2.91905300	-1.39855700	-2.28576100
C	2.17850600	-2.11424300	-3.43279500
H	1.16444100	-1.72283400	-3.55904500
H	2.72476500	-1.94523500	-4.36653800
H	2.11911100	-3.19531300	-3.27688000
C	3.09193000	0.09187300	-2.62051000
H	3.78481200	0.18775800	-3.46296600
H	2.14584400	0.54725500	-2.91757200
H	3.49949200	0.65385900	-1.77620800
C	4.31316900	-2.03102600	-2.07557900
H	4.87925700	-1.93503000	-3.00761400
H	4.88590400	-1.51506400	-1.29853900
H	4.26787100	-3.09602800	-1.83425500
H	-1.27157500	0.33176400	-1.48231400
H	-0.76344500	-1.82170700	-1.42111100
C	3.43454800	-0.32503600	2.83912400
H	2.95409300	0.09788600	3.72690900
C	4.55732000	0.62859200	2.40131200
H	5.28663700	0.73655300	3.20938800
H	5.09239900	0.25135400	1.52386600
H	4.16924100	1.62091700	2.16024200
C	3.98576000	-1.70696700	3.22293800
H	4.69584700	-1.60730200	4.04877800
H	3.18700600	-2.38461000	3.54156700

H	4.52431000	-2.18591100	2.39673700
H	1.57805600	-1.18267900	2.18756200
C	1.21052700	3.48680100	-0.21534900
C	2.71976500	3.39321100	0.07836400
H	2.96603600	3.68577300	1.10359900
H	3.09234900	2.37784000	-0.08730300
H	3.26890700	4.06065300	-0.59270300
C	0.71790000	4.93355200	-0.00301200
H	1.21921300	5.60191900	-0.71018000
H	-0.36166000	5.00918900	-0.17507700
H	0.93035000	5.30847600	1.00313200
H	-0.67375300	2.67416000	0.49872600
C	0.96659900	3.10262800	-1.68319800
H	1.21260300	2.05752000	-1.86002900
H	-0.07594200	3.26179300	-1.96319500
H	1.58829600	3.72071200	-2.33920400
H	0.28616700	0.05229500	-2.03401700
C	-3.35644600	-0.55604000	-1.66201100
H	-4.40984400	-0.51360700	-1.36898800
H	-3.25033800	-0.02851300	-2.61178600
H	-3.07130500	-1.59872000	-1.79457600
C	-1.90135600	-0.02935900	1.95688000
H	-1.50677100	0.98038700	1.88613800
H	-2.84399500	0.01016000	2.51247400
H	-1.21007300	-0.63830100	2.54036600
C	-2.78245700	-2.03434600	0.74138000
C	-2.12709800	-3.26830700	0.68037600
C	-4.15444800	-2.02548800	1.05299800
C	-2.82102300	-4.45944800	0.91180900
H	-1.07342600	-3.29746700	0.43726200
C	-4.84991700	-3.21150300	1.28127600
H	-4.68327900	-1.07813100	1.11774700
C	-4.18353900	-4.43766300	1.20946800
H	-2.29004000	-5.40522100	0.85506400
H	-5.90979400	-3.17682100	1.51529500
H	-4.72166000	-5.36431000	1.38485400
C	-2.85357300	1.60157600	-0.46869800
C	-2.52060800	2.46815000	-1.52088500
C	-3.62495400	2.10358000	0.58897900
C	-2.89047000	3.81081200	-1.49133800
H	-1.95017400	2.08989500	-2.36391400
C	-3.99907800	3.44912300	0.62049800
H	-3.95173500	1.44937000	1.38689300
C	-3.62230100	4.31028800	-0.41036100
H	-2.60442400	4.46510400	-2.30882700
H	-4.58919000	3.81879400	1.45330300
H	-3.90622800	5.35755200	-0.38014500

D

N	-1.10194400	0.86657800	0.53847100
P	-1.24880800	-2.12461600	-0.66053400
Ir	0.48044900	-0.65380500	-0.20951300
H	-0.03162300	-0.30423200	-1.71591300
C	2.55711000	0.48128000	0.29481800
C	1.95297800	0.91945700	-0.96162400
C	-1.95383300	3.03373900	0.90258900
H	-1.43695000	3.77122000	0.28637200
H	-2.60878300	3.53297400	1.61390600
C	-2.17294100	1.06637000	-0.15148900
O	-2.79877300	2.23679900	0.02014600
C	-0.99770500	1.99264600	1.51474600
C	-2.79485500	0.12745000	-1.16937400
N	-2.70360900	-1.25980500	-0.69190900
H	-3.49932400	-1.81543700	-0.98294000
C	-1.00078700	-2.92379500	-2.29290200
H	-0.04105300	-3.44475700	-2.30241200
H	-1.80563900	-3.62405600	-2.52510500
H	-0.97284100	-2.13515100	-3.04714700
C	-1.57442500	-3.51183300	0.55940700
C	-0.33330900	-4.42421200	0.59760500
H	0.56759000	-3.86571100	0.86863900
H	-0.48814400	-5.20163900	1.35298900
H	-0.15766500	-4.92513200	-0.35893900
C	-1.84391300	-2.91988400	1.95302900
H	-2.11763800	-3.73101200	2.63562100
H	-0.95997500	-2.42764900	2.36338900
H	-2.66442300	-2.19826500	1.93117300
C	-2.81347900	-4.32148600	0.11351100
H	-2.95994100	-5.14429000	0.82072600
H	-3.72737100	-3.71924400	0.13135800
H	-2.69955200	-4.76383300	-0.87928300
H	1.60430400	-0.46035700	1.01180600
H	1.43890200	-1.72161300	-0.83749800
C	-4.26754000	0.48926700	-1.49429900
H	-4.25671000	1.55287800	-1.75096500
C	-5.21016400	0.29336800	-0.29674800
H	-6.21622400	0.63405900	-0.55813400
H	-5.28471700	-0.75944500	-0.00586200
H	-4.87492800	0.86174800	0.57403200
C	-4.75675200	-0.28059700	-2.73098000
H	-5.75279900	0.06988300	-3.01586700
H	-4.08789700	-0.13515700	-3.58566700
H	-4.83958400	-1.35839500	-2.54823500
H	-2.21783800	0.27428700	-2.09577800
C	-1.35637700	1.56681000	2.97154800
C	-2.79672900	1.02655300	3.04906100
H	-3.53877600	1.77637000	2.75610400
H	-2.92360000	0.15110400	2.40550100
H	-3.02668800	0.72287400	4.07489500
C	-1.20282300	2.80031100	3.88460800
H	-1.38420000	2.51509200	4.92557700
H	-0.19011800	3.21484900	3.82242800
H	-1.91077400	3.59777000	3.63859200
H	0.02016700	2.37713200	1.49780000
C	-0.38479500	0.47905800	3.45955100
H	-0.43017100	-0.40425900	2.82461400
H	0.64735200	0.83997500	3.46735800
H	-0.64012900	0.18138200	4.48167200
H	0.46573400	-1.49138300	1.18835600
C	2.64291000	1.37782400	1.53570200
H	3.54667200	1.98824800	1.46233400
H	1.78891200	2.03903700	1.64514900
H	2.74131400	0.76557900	2.43425500
C	2.59267800	0.45306600	-2.27264200
H	3.45156700	1.09635800	-2.49870100
H	2.93805500	-0.57892600	-2.22316900
H	1.87778700	0.54699600	-3.09024600
C	1.35471900	2.30050300	-1.12230500
C	0.20380100	2.49822800	-1.90417900
C	2.02750300	3.44134500	-0.65638200
C	-0.28656100	3.77464000	-2.16851200
H	-0.31160600	1.63197900	-2.30185500
C	1.53261800	4.72431700	-0.90712400
H	2.95978900	3.34390300	-0.11442600
C	0.36905600	4.89819600	-1.65602200
H	-1.18628800	3.88971400	-2.76537600
H	2.07016800	5.58655100	-0.52420500
H	-0.01629100	5.89458400	-1.84920200
C	3.78743700	-0.41128400	0.22099700
C	3.82392300	-1.74531300	0.63849600
C	4.97716600	0.19792000	-0.20793500
C	5.02157500	-2.46424600	0.60666700
H	2.91588800	-2.22949500	0.97864800
C	6.17138900	-0.52047700	-0.24247000
H	4.96780700	1.23803500	-0.52147400
C	6.19693100	-1.85751100	0.16264700
H	5.03045800	-3.50082200	0.93005700
H	7.07980400	-0.03514000	-0.58623500
H	7.12575000	-2.41899900	0.13471700

TSs for the migratory insertion pathway

E

N	-1.26315100	0.58782600	-1.22948400
P	-1.55626100	-1.40372900	1.34319200
Ir	0.30362900	-0.88021600	-0.01538200
H	0.26569700	-1.64732600	-1.50361000
C	2.32689700	0.23957600	0.70193700
C	2.32243000	-0.26468900	-0.69470600
C	-2.03029200	1.48301600	-3.27216000
H	-1.53719400	0.97811600	-4.10797800
H	-2.59854500	2.33497800	-3.64124100
C	-2.38577200	0.05629900	-1.58190000
O	-2.97570900	0.54343000	-2.68570900
C	-1.07259000	1.78635700	-2.10824200
C	-3.12550800	-1.09583500	-0.91904700
N	-3.00382800	-1.00960100	0.53995700
H	-3.83303700	-1.36199000	1.00427500
C	-1.61816800	-3.20393100	1.71432800
H	-0.73477200	-3.49215600	2.28930100
H	-2.52209900	-3.47790300	2.26307600
H	-1.59836500	-3.74281800	0.76385700
C	-1.74595600	-0.53398600	2.99467100
C	-0.50870000	-0.85404500	3.85408400
H	0.42114800	-0.58687900	3.34450500
H	-0.55703900	-0.28101700	4.78606200
H	-0.45523200	-1.91395800	4.12006800
C	-1.84126000	0.97769500	2.72571400
H	-1.91424900	1.51375200	3.67806800
H	-0.96307400	1.34913400	2.19618400
H	-2.72016000	1.21912700	2.12309700
C	-3.02066000	-1.00013500	3.72707500
H	-3.09354900	-0.46563600	4.68036700
H	-3.92899300	-0.76935000	3.16159000
H	-3.00744300	-2.06958100	3.95345700
H	1.04067900	0.07445700	1.19198600
H	1.05392400	-2.08130800	0.62965700
C	-4.62109600	-1.16502600	-1.33233900
H	-4.62668600	-1.14530400	-2.42603500
C	-5.43759900	0.03362800	-0.82613500
H	-6.46240400	-0.03001100	-1.20382400
H	-5.49026800	0.05635200	0.26712500
H	-5.00977500	0.98023800	-1.16452800
C	-5.24416100	-2.49940800	-0.89211400
H	-6.25907800	-2.58389300	-1.29077200
H	-4.66429800	-3.35380700	-1.25660100
H	-5.32270800	-2.58729300	0.19788900
H	-2.65686700	-2.01164200	-1.31761000
C	-1.36638900	3.12010700	-1.35675900
C	-2.81705900	3.16707600	-0.84192700
H	-3.54780600	3.09920800	-1.65432100
H	-3.01300800	2.35459700	-0.13573200
H	-2.99509000	4.11176800	-0.31892300
C	-1.11268000	4.29210200	-2.32496000
H	-1.23349000	5.24249700	-1.79548000
H	-0.09306900	4.25892800	-2.72519700
H	-1.80915300	4.29931300	-3.16951600
H	-0.04553600	1.81030500	-2.46449400
C	-0.40455200	3.24777000	-0.16778500
H	-0.48818800	2.38763100	0.49518400
H	0.63728300	3.31961300	-0.49293600
H	-0.62880300	4.14773500	0.41344500
H	-0.46408800	-1.99498500	-1.01375400
C	2.23579000	0.72609300	-1.85547300
H	1.67241200	1.61745200	-1.58705700
H	3.23257200	1.04492200	-2.18024000
H	1.75901800	0.24794100	-2.71436400
C	3.08275700	-0.58731600	1.75241000
H	3.00422600	-1.65738200	1.56466500
H	4.13708900	-0.30045700	1.71830700
H	2.71089200	-0.36665600	2.75519300
C	3.31680200	-1.36732100	-1.02098200
C	2.97431500	-2.65574700	-1.44724500
C	4.68363300	-1.03558600	-0.96822500
C	3.95845000	-3.58520600	-1.79785800
H	1.93224200	-2.94703800	-1.49564600
C	5.66913200	-1.96014300	-1.30917200
H	4.97953600	-0.03833100	-0.65354700
C	5.30882300	-3.24439300	-1.72622600
H	3.66294600	-4.57849300	-2.12322600
H	6.71605600	-1.67685500	-1.25137200
H	6.07266400	-3.96853600	-1.99318700
C	2.44842400	1.71837000	1.03068300
C	1.76070000	2.26068700	2.12482200
C	3.37774600	2.52332500	0.35910800
C	1.94880400	3.58721500	2.50551200
H	1.06435400	1.64264900	2.68202700
C	3.57206800	3.85283300	0.74109500
H	3.96208000	2.12031400	-0.45835100
C	2.85071500	4.39316000	1.80611100
H	1.39079500	3.98970400	3.34523300
H	4.29101600	4.46279300	0.20276400
H	2.99614000	5.42939100	2.09530500

F

N	-1.40549300	0.88100300	0.16830900
P	-1.03554100	-2.29197500	-0.26189600
Ir	0.40406100	-0.48514500	-0.77245400
H	0.05730300	0.24727300	-2.23169600
C	2.36196800	0.15534700	0.43135200
C	2.11843800	0.91475400	-0.82623800
C	-2.43593600	2.94369900	-0.27820400
H	-1.94685800	3.36739200	-1.15834200
H	-3.11139300	3.67447500	0.16006800
C	-2.50629700	0.71883700	-0.49264700
O	-3.24553000	1.81216300	-0.72707200
C	-1.44304500	2.28944200	0.69397000
C	-3.05492900	-0.56569000	-1.09234700
N	-2.64250000	-1.72490500	-0.29873100
H	-3.33640700	-2.46264300	-0.32289300
C	-0.90717600	-3.64616800	-1.49914200
H	0.12370700	-4.00623900	-1.53671300
H	-1.57835600	-4.47799600	-1.27226300
H	-1.16330700	-3.23423100	-2.47826800
C	-0.93433700	-3.13417000	1.41561000
C	0.37271600	-3.94143000	1.50494800
H	1.25156000	-3.30232600	1.39017400
H	0.43583600	-4.40611200	2.49468500
H	0.42224300	-4.74228800	0.76165700
C	-0.94466900	-2.04309600	2.49710700
H	-0.93732400	-2.50901000	3.48837100
H	-0.05968400	-1.40549300	2.41995700
H	-1.83364100	-1.41238600	2.41876300
C	-2.14237500	-4.07181400	1.61998600
H	-2.03997500	-4.56994600	2.59001200
H	-3.08761600	-3.52169900	1.63706500
H	-2.20099200	-4.85472700	0.85800300
H	1.38927800	-0.75915800	0.59535800
H	1.34726700	-1.45762700	-1.53609900
C	-4.60009900	-0.53946300	-1.26064600
H	-4.81841800	0.37938700	-1.81292200
C	-5.35360100	-0.48810000	0.07536500
H	-6.43213700	-0.46580700	-0.10669800
H	-5.13937800	-1.36426400	0.69570100
H	-5.09133700	0.40368600	0.64771400
C	-5.06709800	-1.72072200	-2.12650500
H	-6.12968100	-1.61002100	-2.36039900
H	-4.51463000	-1.77144100	-3.07052200
H	-4.95388900	-2.68486200	-1.61701200
H	-2.65060600	-0.60132700	-2.11817600
C	-1.89082200	2.38816000	2.19105900
C	-3.28788500	1.77898800	2.40870200
H	-4.05233100	2.26050900	1.79177400
H	-3.29036100	0.70757200	2.18691800
H	-3.58638300	1.90011900	3.45440300
C	-1.90486500	3.88091100	2.58486800
H	-2.09723000	3.97654300	3.65820600
H	-0.93918300	4.35396200	2.37316100
H	-2.68213200	4.44912200	2.06532800
H	-0.45204700	2.73039200	0.59341300
C	-0.89128800	1.66705300	3.10843900
H	-0.70296700	0.64886200	2.76955200
H	0.06030000	2.20305900	3.15534500
H	-1.28686600	1.61988800	4.12813100
C	2.13726000	0.83456900	1.78451700
H	1.32441700	1.55006200	1.72373100
H	1.90564600	0.09305000	2.55218700
H	3.03846400	1.36251800	2.09965300
C	3.05518900	0.63389500	-2.00745800
H	4.00201900	1.16975000	-1.86262900
H	3.27969100	-0.42644200	-2.12366600
H	2.60180600	0.99855100	-2.93184300
H	-0.55138200	-0.47746200	-2.15244000
C	1.80506600	2.39595300	-0.78468400
C	0.83709100	2.95981500	-1.63153700
C	2.59466800	3.27708200	-0.02661700
C	0.62907400	4.33818900	-1.68357000
H	0.22122300	2.30780800	-2.24174700
C	2.37807000	4.65615900	-0.05900500
H	3.39821800	2.89035100	0.58935800
C	1.38744000	5.19384400	-0.88185600
H	-0.13176400	4.74172600	-2.34547300
H	2.99447300	5.30866900	0.55222600
H	1.21765200	6.26576500	-0.90906400
C	3.57170100	-0.77182500	0.43835100
C	3.51249300	-2.14336500	0.17732500
C	4.82121400	-0.19017000	0.70478100
C	4.67037500	-2.92508400	0.19831700
H	2.56186900	-2.60312500	-0.06322700
C	5.97842500	-0.96779800	0.71903400
H	4.89764500	0.87713900	0.88932600
C	5.90728000	-2.34117300	0.47069200
H	4.59977500	-3.98979600	-0.00289800
H	6.93605800	-0.49831000	0.92277900
H	6.80815400	-2.94683700	0.48509600

G

N	0.86660900	1.07799800	0.50121800
P	1.55789100	-1.86625100	-0.61786500
Ir	-0.28057100	-0.40447900	-0.83940500
H	-0.96064100	-1.13306000	0.53127300
C	-2.32916700	0.42834300	-0.50808300
C	-2.13397700	-0.45411500	0.67012200
C	0.99470700	2.80131700	2.09263400
H	0.05512100	2.87216100	2.64744400
H	1.63419200	3.64260300	2.35150200
C	1.46708200	0.67257100	1.56968900
O	1.67504500	1.58381100	2.52832200
C	0.79056000	2.57110700	0.58608300
C	1.93569100	-0.74061500	1.87164200
N	2.54353900	-1.32743600	0.66659200
H	3.27419000	-1.98710400	0.91025400
C	1.04958900	-3.58432900	-0.19671900
H	0.38658900	-3.97964500	-0.96974000
H	1.91097200	-4.24576800	-0.08217900
H	0.50369300	-3.55125200	0.74924500
C	2.76495000	-2.04289400	-2.04451200
C	2.01985200	-2.71374500	-3.21492000
H	1.11661600	-2.15877600	-3.48961500
H	2.67752200	-2.73972600	-4.09012900
H	1.73552400	-3.74470200	-2.98504800
C	3.25666500	-0.64655300	-2.46511400
H	4.04171800	-0.75517800	-3.22080000
H	2.45848700	-0.05189800	-2.91630300
H	3.67026300	-0.09069600	-1.61930800
C	3.98462600	-2.89309500	-1.62967600
H	4.64342800	-3.00628600	-2.49700700
H	4.56980000	-2.40675100	-0.84293000
H	3.71233600	-3.89882800	-1.29829100
H	-0.26453400	0.57818100	-2.19944800
H	-0.99817100	-1.48550600	-1.72383300
C	2.92087600	-0.80592000	3.06769300
H	2.43385800	-0.25975200	3.88158500
C	4.26703200	-0.12713000	2.77041100
H	4.89479100	-0.14101600	3.66621100
H	4.81506000	-0.64313700	1.97554100
H	4.13277900	0.91398700	2.46725400
C	3.11054600	-2.25813500	3.53334600
H	3.72230100	-2.28068800	4.43960400
H	2.15164900	-2.73582400	3.75954800
H	3.62529100	-2.87385700	2.78643300
H	1.03088300	-1.28996000	2.17937800
C	1.84715700	3.28421100	-0.31399300
C	3.28534400	2.93597900	0.11419500
H	3.51090400	3.26455500	1.13346700
H	3.46465100	1.85772400	0.05408000
H	3.99989100	3.42799800	-0.55288500
C	1.60816200	4.80375000	-0.19813400
H	2.30084600	5.34084300	-0.85364200
H	0.58672700	5.06277800	-0.49730800
H	1.76876800	5.17570200	0.81880400
H	-0.20154500	2.88894700	0.27592400
C	1.65910400	2.87009100	-1.78281300
H	1.89373000	1.81513000	-1.92058200
H	0.63562300	3.05042900	-2.12119200
H	2.33323000	3.44862200	-2.42254500
H	0.53982200	0.10657800	-2.20956100
C	-3.04086900	-1.67494200	0.75747300
C	-2.64513100	-2.96602300	0.40315300
C	-4.34893100	-1.46721000	1.22135900
C	-3.53320400	-4.03848300	0.51797300
H	-1.64788000	-3.12982600	0.01506400
C	-5.23667500	-2.53627000	1.33358800
H	-4.67939600	-0.46564300	1.48175200
C	-4.83066000	-3.82770000	0.98502900
H	-3.20734900	-5.03573500	0.23782400
H	-6.24674700	-2.35912500	1.69042200
C	-3.31608400	-0.06279000	-1.57518000
H	-3.24750700	-1.13706700	-1.74382100
H	-4.34215900	0.16961200	-1.26256700
H	-3.13173000	0.44743600	-2.52227200
H	-5.52239500	-4.65980800	1.07319100
C	-2.42382200	1.93219500	-0.38468100
C	-2.01174600	2.73668500	-1.46311300
C	-3.03329800	2.57795900	0.70246400
C	-2.13736400	4.12305200	-1.43302000
H	-1.56335400	2.26419700	-2.33118800
C	-3.14721500	3.97129400	0.74682800
H	-3.44124100	2.00252600	1.52288400
C	-2.68930400	4.75261500	-0.31281900
H	-1.79181000	4.71158500	-2.27753500
H	-3.60752600	4.43971900	1.61177500
H	-2.77317300	5.83428400	-0.27609900
C	-1.82876700	0.11466600	2.06334400
H	-1.25353700	1.03480900	2.01268500
H	-2.76131500	0.31681000	2.59312800
H	-1.27940600	-0.62169900	2.65497300

H

N	-1.04387700	0.79632100	0.84179300
P	-1.32633800	-1.81301300	-1.03281800
Ir	0.47822400	-0.73024100	0.01157900
H	0.70740100	0.18466700	-1.39045000
C	2.41023400	0.35697400	0.22221900
C	1.96492600	0.75353800	-1.13319200
C	-1.66393700	2.94534900	1.53256300
H	-0.98126100	3.65345900	1.05385500
H	-2.28856900	3.45901400	2.26037900
C	-2.03416800	1.23384500	0.13682900
O	-2.54341000	2.42143300	0.49029800
C	-0.95150900	1.68502700	2.04313000
C	-2.68870400	0.57521800	-1.06836200
N	-2.74499800	-0.88034600	-0.88558900
H	-3.54168800	-1.27846700	-1.37019000
C	-0.99904400	-2.05151800	-2.82814800
H	-0.10301000	-2.66398500	-2.95383000
H	-1.84105900	-2.51434400	-3.34724400
H	-0.80262500	-1.06971800	-3.26572500
C	-1.85272000	-3.49016800	-0.37255300
C	-0.64078000	-4.43896000	-0.45700900
H	0.21336900	-4.05334500	0.10790500
H	-0.91720200	-5.40981900	-0.03242000
H	-0.32123100	-4.60940000	-1.48950800
C	-2.29761700	-3.33300700	1.09339700
H	-2.64489100	-4.30219500	1.46652600
H	-1.47498200	-3.01330700	1.73894600
H	-3.11329900	-2.61231100	1.19264400
C	-3.02549400	-4.06144600	-1.19750500
H	-3.29927000	-5.03970000	-0.78835900
H	-3.91975200	-3.43190600	-1.14117800
H	-2.76652800	-4.20991200	-2.24896200
H	0.83116300	-1.25400600	1.56553200
H	1.39971500	-1.84527100	-0.59705200
C	-4.10284600	1.14567700	-1.36559200
H	-3.97805900	2.23174800	-1.41272800
C	-5.12278400	0.82406100	-0.26399900
H	-6.08943600	1.27240200	-0.51171500
H	-5.27487700	-0.25456000	-0.15718000
H	-4.80434300	1.21977800	0.70278000
C	-4.59416900	0.67699000	-2.74428600
H	-5.52650800	1.18909300	-2.99798200
H	-3.86199300	0.89648300	-3.52825800
H	-4.80622000	-0.39842000	-2.77095300
H	-2.06738900	0.84855400	-1.93644800
C	-1.57888100	1.04881300	3.32194300
C	-3.08566100	0.78257500	3.15409300
H	-3.65264000	1.70113600	2.97348100
H	-3.27419600	0.09175500	2.32640700
H	-3.48505200	0.32370500	4.06383600
C	-1.34150600	2.02546000	4.49185700
H	-1.73732100	1.59902900	5.41876300
H	-0.27225000	2.21502700	4.63860400

H	-1.83806000	2.98881900	4.33864800
H	0.09406800	1.88747100	2.25589500
C	-0.87603600	-0.28222700	3.64095000
H	-1.13848800	-1.04377100	2.90565800
H	0.21411400	-0.17062800	3.65696900
H	-1.19128500	-0.64658700	4.62370000
C	2.65063600	0.13446600	-2.35805300
H	3.57920800	0.67608100	-2.55632300
H	2.88548500	-0.91667000	-2.19259200
H	2.01082500	0.22898200	-3.23885200
H	0.12189100	-1.78399300	1.26976800
C	2.41867800	1.42110200	1.31851500
H	3.36318500	1.97774700	1.30549200
H	1.61528500	2.14390000	1.19195700
H	2.32996100	0.95097300	2.30084100
C	3.65226500	-0.51105400	0.34196500
C	3.68547000	-1.76100700	0.97415600
C	4.87254800	0.02631600	-0.10606700
C	4.88549200	-2.45984900	1.13075900
H	2.76689400	-2.20240500	1.34076800
C	6.07178700	-0.67023400	0.03857800
H	4.88624100	1.00707800	-0.57277700
C	6.08271500	-1.92266900	0.65716600
H	4.87862400	-3.42842800	1.62239600
H	6.99572000	-0.23215700	-0.32760500
H	7.01369600	-2.46955900	0.77268600
C	1.59870000	2.20920800	-1.36506400
C	0.32475100	2.62494200	-1.75470200
C	2.61240400	3.17253300	-1.24706500
C	0.04412000	3.97372500	-1.98117000
H	-0.45195600	1.88462700	-1.88759400
C	2.34051900	4.52000200	-1.48062500
H	3.61547100	2.87199800	-0.96205200
C	1.05200400	4.92790400	-1.83734200
H	-0.95918500	4.27166900	-2.26985400
H	3.13577000	5.25200000	-1.37796000
H	0.83939100	5.97865200	-2.00805300

Free energy profile

Int1

N	1.28296600	-0.13965300	-0.44743700
P	-1.86120500	0.16209400	-0.03816400
Ir	-0.52013100	-1.34653100	-0.93477400
H	-0.65800100	-0.43656000	-2.21327600
C	3.42811300	0.58047600	-1.05969000
H	3.59142000	0.42219700	-2.12869500
H	4.35743000	0.88738300	-0.58455500
C	1.29285700	1.13103600	-0.66876700
O	2.48075400	1.68660500	-0.92222300
C	2.70250500	-0.59863300	-0.38104900
C	0.08498600	2.04469000	-0.72985600
N	-0.95697700	1.58054000	0.19639300
H	-1.50335700	2.35080700	0.56436300
C	-3.28234400	0.54545400	-1.12915400
H	-3.86260200	-0.36151800	-1.30851000
H	-3.92000500	1.31668500	-0.69098500
H	-2.88477600	0.89952500	-2.08206900
C	-2.55556000	-0.19808200	1.66479200
C	-3.53443200	-1.38364800	1.55499300
H	-3.05441000	-2.26981300	1.12971400
H	-3.89007300	-1.64291100	2.55753800
H	-4.41061100	-1.14076700	0.94669000
C	-1.40041700	-0.53986000	2.62476400
H	-1.79156600	-0.59301000	3.64588800
H	-0.95563000	-1.50969800	2.39584800
H	-0.61585800	0.22097700	2.59658800
C	-3.29783800	1.05016000	2.19360900
H	-3.75237000	0.79676000	3.15689200
H	-2.61763000	1.88836500	2.37298600
H	-4.10353200	1.37980700	1.53230600
H	-0.75304000	-2.73947900	0.26317500
H	-1.79329000	-2.11092100	-1.51390500
C	0.45515200	3.52293300	-0.43534600
H	1.26934200	3.76059400	-1.12692100
C	0.96497400	3.73156000	0.99818500
H	1.25291000	4.77688400	1.14229200
H	0.19638200	3.49332500	1.74012300
H	1.83963600	3.10963900	1.20585900
C	-0.71945300	4.45593500	-0.76570000
H	-0.39972400	5.49821400	-0.68083500
H	-1.08618700	4.29411000	-1.78444400
H	-1.56413900	4.32731000	-0.07861600
H	-0.25177100	1.99960300	-1.77819200
C	3.17586900	-0.89744500	1.07447400
C	3.09755500	0.36060300	1.95986900
H	3.74358900	1.16655700	1.59811200
H	2.07247000	0.74006600	2.01625800
H	3.41756800	0.11768000	2.97768000
C	4.62849200	-1.41024000	1.00687800
H	4.98162400	-1.65762400	2.01263000
H	4.70001100	-2.31531600	0.39348500
H	5.31705700	-0.66643200	0.59407600
H	2.80349200	-1.51097500	-0.97438800
C	2.29605600	-1.99531000	1.69638600
H	1.28171400	-1.62969000	1.86817400
H	2.24884700	-2.88337700	1.05552000
H	2.70439200	-2.30188100	2.66446300
H	-0.08817300	-2.40183600	0.57353300

Int2*Si*

N	0.01994300	1.07425400	-0.29743900
P	-2.25171300	-1.06357200	0.34366600
Ir	-0.03072500	-1.19591700	-0.14435600
H	-0.63777000	-1.20781300	-1.59594000
C	2.43406600	-1.73951200	-0.65829300
C	1.75431600	-1.55545300	-1.86266400
C	0.88034000	3.04146300	-1.22737200
H	1.44743200	2.78454700	-2.12562100
H	1.02471200	4.09317900	-0.99019200
C	-0.87343800	1.66492500	-1.01709700
O	-0.53798300	2.85208500	-1.53742700
C	1.11107200	2.06846600	-0.05877000
C	-2.26136100	1.13733400	-1.32999100
N	-2.81085800	0.47726600	-0.13007100
H	-3.82099600	0.55830600	-0.09881800
C	-3.18708300	-2.30370000	-0.63927600
H	-2.80324800	-3.30345700	-0.42718800
H	-4.25886500	-2.26603600	-0.43219400
H	-3.01254500	-2.08491300	-1.69493300
C	-2.86346700	-1.25581800	2.10398900
C	-2.60630900	-2.71434000	2.53410500
H	-1.55474800	-2.99384100	2.41039500
H	-2.86031900	-2.82256400	3.59371600
H	-3.22049600	-3.42456200	1.97325900
C	-2.09197200	-0.29444700	3.02222300
H	-2.51365900	-0.34571100	4.03160500
H	-1.03749600	-0.56804400	3.09257600
H	-2.16490800	0.73973100	2.67507500
C	-4.37094900	-0.93383200	2.18871400
H	-4.70891300	-1.11894300	3.21381000
H	-4.57557400	0.11797400	1.96566800
H	-4.97770000	-1.56015200	1.52924800
H	0.51579200	-1.69677000	1.51735400
H	-0.18029000	-2.76516900	-0.20998700
C	-3.22335500	2.23713000	-1.84837600
H	-2.68920000	2.72687000	-2.66912000
C	-3.55454700	3.29955900	-0.79001800
H	-4.19064600	4.07410300	-1.22863300
H	-4.09551100	2.87061500	0.05959500
H	-2.65074300	3.78016800	-0.40909600
C	-4.49714900	1.60778400	-2.43415400
H	-5.10813400	2.37987300	-2.91015200
H	-4.26104500	0.84828200	-3.18678700
H	-5.12150400	1.13812600	-1.66522400
H	-2.12316200	0.42339800	-2.15757900
C	1.01435300	2.74922300	1.34543600
C	-0.28301900	3.56655500	1.49776100
H	-0.35921100	4.38045200	0.77044400
H	-1.16673100	2.92871600	1.39359900
H	-0.31812300	4.01564700	2.49507300
C	2.24449300	3.66498500	1.50682400
H	2.23366700	4.12963200	2.49783800
H	3.17354800	3.09236500	1.41187100
H	2.26601200	4.47348100	0.76890000
H	2.07519100	1.56405200	-0.13434100
C	1.04502400	1.68658100	2.45406800
H	0.18079700	1.02811800	2.38139000
H	1.95337200	1.08489800	2.40145100
H	1.01688000	2.16903900	3.43658700
H	0.71478000	-0.90400500	1.51092700
C	1.79565000	-0.27347600	-2.67243900
H	2.08274200	0.59569800	-2.08952200
H	2.51725200	-0.38856900	-3.49096000
H	0.82108400	-0.08310800	-3.12776100
C	2.65847100	-3.13241400	-0.08379300
H	1.88536400	-3.84416000	-0.36522400
H	3.62573500	-3.50619900	-0.44327000
H	2.71039200	-3.10882400	1.00622800
C	3.40004500	-0.73450800	-0.09092100
C	3.54429600	-0.57216300	1.29811600
C	4.30032600	-0.04479700	-0.92294000
C	4.50065000	0.28977900	1.83230200
H	2.89887000	-1.11951000	1.97618500
C	5.25629300	0.82338100	-0.39190300
H	4.27689300	-0.20383200	-1.99388400
C	5.35333600	1.00542800	0.98811700
H	4.57463400	0.40543400	2.90931500
H	5.93318300	1.34582900	-1.06116600
H	6.09516600	1.68081800	1.40286700
C	1.30904300	-2.73366900	-2.71205700
H	1.00295300	-3.60569200	-2.13848100
H	0.47976200	-2.44830700	-3.36298100
H	2.15238500	-3.02405800	-3.35305500

Int2*Re*

N	1.17403800	1.19183200	0.08486400
P	0.90466100	-1.99125300	0.00147100
Ir	-0.58552000	-0.28098700	0.18677200
H	-0.23322900	-0.46757700	1.70792200
C	-2.65744600	1.21497800	0.30225300
C	-2.03488100	1.26224100	1.54606400
C	2.24763100	3.20211300	0.63773800
H	1.71903200	3.65788900	1.47944200
H	2.99184100	3.89347200	0.24837900
C	2.20322500	0.97332500	0.83484000
O	2.95826400	2.03320000	1.14789000
C	1.29585600	2.60041400	-0.40768300
C	2.64078400	-0.36162100	1.40967400
N	2.45887600	-1.41481600	0.39609600
H	3.14000500	-2.15723100	0.51053400
C	0.49987000	-3.31871900	1.20527500
H	-0.50116900	-3.70518400	1.00388000
H	1.22689400	-4.13318000	1.17169500
H	0.50127800	-2.87179900	2.20152400
C	1.14966900	-2.85429900	-1.64533100
C	-0.15215900	-3.60412100	-1.99111700
H	-1.01911000	-2.93521300	-1.98759900
H	-0.06210300	-4.03167600	-2.99514200
H	-0.35006100	-4.42631900	-1.29773400
C	1.45991600	-1.80614900	-2.72753400
H	1.72629700	-2.31833200	-3.65804900
H	0.59376500	-1.17681500	-2.93921400
H	2.29768400	-1.16490000	-2.44059200
C	2.32892700	-3.84618800	-1.55615900
H	2.41549000	-4.37206100	-2.51290300
H	3.27943000	-3.33160100	-1.38427100
H	2.19053300	-4.60514600	-0.78151200
H	-0.81636600	0.18489300	-1.58017300
H	-1.70756500	-1.35106200	0.45746800
C	4.10438700	-0.34560800	1.92240500
H	4.17641000	0.52833500	2.57741700
C	5.13283400	-0.18772200	0.79241800
H	6.13864100	-0.10624800	1.21466100
H	5.12675200	-1.04997100	0.11778000
H	4.94033700	0.70855900	0.19809900
C	4.39308300	-1.58931500	2.77757000
H	5.38736200	-1.50860300	3.22601700
H	3.66354200	-1.69986900	3.58664000
H	4.38507800	-2.51313900	2.18735500
H	1.99577200	-0.52457000	2.28749000
C	1.80386100	2.69739100	-1.87907100
C	3.20635100	2.08097900	-2.03437200
H	3.95839200	2.60760100	-1.43857300
H	3.21136200	1.02751200	-1.73825400
H	3.52181500	2.13332000	-3.08080300
C	1.83376900	4.18793800	-2.27355600
H	2.14605600	4.28857200	-3.31756500
H	0.84166200	4.64298900	-2.17528500
H	2.53521100	4.76899900	-1.66707100
H	0.32138400	3.08269100	-0.36071600
C	0.82685400	1.96622300	-2.81398000
H	0.82218800	0.89742800	-2.60505700
H	-0.19370200	2.34995900	-2.71024300
H	1.12993300	2.10034700	-3.85712600
C	-2.51325200	0.41597300	2.71433500
H	-3.24229300	0.99206600	3.29714200
H	-2.98283800	-0.51418000	2.39786800
H	-1.67957700	0.18017800	3.37966300
H	-1.23212300	-0.51012300	-1.49758300
C	-2.50971100	2.29881400	-0.75303800
H	-3.30897000	3.03710900	-0.61271600
H	-1.55694000	2.82194100	-0.71641700
H	-2.63319400	1.88085100	-1.75481800
C	-3.91836000	0.41172900	0.09166900
C	-4.08442000	-0.49395300	-0.96652800
C	-5.02957000	0.69252600	0.90338500
C	-5.30587900	-1.13336300	-1.17858900
H	-3.25392500	-0.71309100	-1.62639700
C	-6.25513600	0.05909100	0.69033600
H	-4.93769900	1.42079100	1.70298400
C	-6.39584800	-0.86353300	-0.34732900
H	-5.40536400	-1.84063600	-1.99674400
H	-7.09790500	0.29050800	1.33503300
H	-7.34648500	-1.36164000	-0.51245600
C	-1.18147600	2.42570000	2.00431400
H	-0.28638300	2.07569400	2.52442500
H	-0.88308400	3.09961400	1.20604600
H	-1.76868900	3.00985100	2.72425500

Int3$_{Si}$

N	-0.14495400	1.11730500	0.08946900
P	2.27291500	-1.01625600	-0.13176200
Ir	-0.07455100	-1.07461900	-0.51781200
H	-0.07190800	-1.44492600	1.28456700
C	-2.03799500	-1.73188700	0.32237200
C	-1.20594900	-1.69990900	1.59042700
C	-1.15506800	3.04503100	0.96965900
H	-1.93493900	2.77043900	1.68545600
H	-1.19135400	4.11649200	0.78385700
C	0.56139600	1.60396200	1.05475900
O	0.13349900	2.75308200	1.59113800
C	-1.15228700	2.15660100	-0.28629500
C	1.80725800	1.00291400	1.68926200
N	2.68495500	0.43427700	0.65461700
H	3.65903600	0.48464000	0.93205100
C	2.79547000	-2.41481300	0.94400800
H	2.51519300	-3.35893600	0.47177300
H	3.86988600	-2.40201300	1.13938200
H	2.26013200	-2.33351000	1.89342200
C	3.39575100	-1.04490400	-1.63214900
C	3.17100500	-2.37591300	-2.37559300
H	2.12409000	-2.50442800	-2.66594300
H	3.77819800	-2.37895800	-3.28682600
H	3.47372700	-3.23917800	-1.77539900
C	3.03891200	0.14309100	-2.54432700
H	3.74790800	0.17671700	-3.37830500
H	2.03654000	0.04200200	-2.96723600
H	3.09387500	1.09419700	-2.00805900
C	4.87150700	-0.92544500	-1.19539200
H	5.50424000	-0.97555300	-2.08761800
H	5.08203100	0.03174100	-0.70805800
H	5.18243800	-1.73657200	-0.53144800
H	-1.22926400	-0.97386800	-1.59940000
H	0.06959800	-2.58036700	-0.94199200
C	2.58596000	2.02590100	2.55894000
H	1.84565200	2.45597700	3.24029100
C	3.20341800	3.16608400	1.73505600
H	3.68082700	3.88793600	2.40425100
H	3.96992300	2.79900100	1.04505100
H	2.44682700	3.69521800	1.15108700
C	3.64424000	1.31315500	3.41521700
H	4.09381900	2.02331300	4.11474500
H	3.20804200	0.49379000	3.99609800
H	4.46236400	0.90186800	2.81219900
H	1.43963400	0.22718900	2.38105400
C	-0.78995000	2.90668400	-1.60490400
C	0.55729600	3.64236700	-1.48122800
H	0.54555800	4.40400100	-0.69514000
H	1.37078300	2.94176300	-1.26795400
H	0.79334600	4.14826000	-2.42242200
C	-1.92302500	3.91241600	-1.89703000
H	-1.72982700	4.42457000	-2.84472100
H	-2.88940000	3.40234200	-1.98021600
H	-2.01298700	4.68248400	-1.12447300
H	-2.11836000	1.67252900	-0.41487700
C	-0.70891700	1.91707700	-2.77986600
H	0.11123100	1.21223800	-2.64737300
H	-1.63539800	1.34527300	-2.89066400
H	-0.53982800	2.46357100	-3.71334600
H	0.41199300	-0.76994500	-1.96924700
C	-1.48265600	-0.58354200	2.61008400
H	-1.76483200	0.35166400	2.13028100
H	-2.29298900	-0.88038300	3.27869000
H	-0.59974700	-0.41299800	3.23178500
C	-2.43645100	-3.11358900	-0.20785000
H	-1.60853600	-3.82076200	-0.23943400
H	-3.22749800	-3.52781700	0.43048800
H	-2.83988800	-3.01454400	-1.21732100
C	-3.25538800	-0.82872300	0.22482800
C	-3.58210500	-0.11157400	-0.93421600
C	-4.21017600	-0.86245600	1.25690400
C	-4.77932900	0.59853800	-1.04050500
H	-2.88295300	-0.10161900	-1.76287100
C	-5.40676200	-0.15126200	1.15962600
H	-4.03349400	-1.46904700	2.13839000
C	-5.69280500	0.59412100	0.01428300
H	-4.99058600	1.15559300	-1.94844200
H	-6.11903000	-0.18995300	1.97847600
H	-6.62121400	1.15191900	-0.06044800
C	-0.96527000	-3.04992700	2.28708300
H	-0.52296000	-3.78687200	1.61719200
H	-0.29355400	-2.91428700	3.13864500
H	-1.91614900	-3.44040300	2.66132200

Int3$_{Re}$

N	0.91958400	1.29923900	0.12865300
P	1.10100500	-1.91438600	-0.25094400
Ir	-0.62822700	-0.27189300	-0.38854700
H	-0.72787300	-0.75072400	1.40119200
C	-2.32329100	0.61713700	0.72395200
C	-1.75170000	-0.28479700	1.80702600
C	1.72743500	3.34683100	0.93537300
H	1.05907300	3.73420600	1.70957400
H	2.47448500	4.09672200	0.68607200
C	1.83197600	1.11022400	1.02221100
O	2.43490600	2.20203400	1.50428000
C	0.97439900	2.74268300	-0.26518400
C	2.27073900	-0.22670700	1.60197200
N	2.44519500	-1.20878900	0.51762700
H	3.19590200	-1.85897000	0.72046600
C	0.56710900	-3.35445500	0.76459700
H	-0.32515900	-3.80373900	0.32326200
H	1.35476300	-4.10535700	0.85781000
H	0.30992400	-2.98682800	1.76099800
C	1.79323400	-2.61659600	-1.84326300
C	0.65211000	-3.35127400	-2.57356900
H	-0.18948900	-2.68332100	-2.77973600
H	1.02900500	-3.72817900	-3.53005600
H	0.28373000	-4.20900800	-2.00317200
C	2.32486800	-1.46279700	-2.71205000
H	2.77738300	-1.87950100	-3.61801900
H	1.52500700	-0.78677800	-3.02261400
H	3.08661600	-0.88122800	-2.18659300
C	2.94682500	-3.59578200	-1.53982100
H	3.32636100	-3.99023500	-2.48821600
H	3.78707600	-3.10443700	-1.03856800
H	2.62648300	-4.45049600	-0.93876000
H	-1.41972100	0.79204800	-1.26816500
H	-1.65733000	-1.38952800	-0.79305900
C	3.56182500	-0.11818000	2.45425400
H	3.38614400	0.70808900	3.15028300
C	4.80741700	0.21594700	1.61824000
H	5.66626500	0.36118700	2.27991400
H	5.06170100	-0.59136100	0.92395300
H	4.66767700	1.13080100	1.03771600
C	3.76483300	-1.39211500	3.28898200
H	4.62605600	-1.26887000	3.95148400
H	2.88829200	-1.60907400	3.90824200
H	3.96309100	-2.27261300	2.66681400
H	1.46553200	-0.52438200	2.29323600
C	1.65910400	2.98797800	-1.64446300
C	3.10131600	2.44932300	-1.65625800
H	3.73695700	2.95342800	-0.92094800
H	3.12212000	1.37576700	-1.44690900
H	3.55168700	2.60587300	-2.64117900
C	1.65806900	4.50864600	-1.91004400
H	2.07965500	4.70875000	-2.89993200
H	0.63942300	4.91220200	-1.89238100
H	2.25770700	5.06746200	-1.18517200
H	-0.04318400	3.12859100	-0.32512100
C	0.84984000	2.31066400	-2.76304000
H	0.88472800	1.22619600	-2.67517200
H	-0.20158000	2.61773700	-2.73803600
H	1.26115900	2.58507200	-3.73966800
C	-2.54435700	-1.56080500	2.12598800
H	-3.41392600	-1.31517900	2.74008100
H	-2.89109000	-2.05044900	1.21580700
H	-1.92061400	-2.26096900	2.68796100
H	-0.22858900	-0.28375400	-1.89665800
C	-2.18832100	2.12005500	0.96292500
H	-2.87444200	2.43314600	1.75866200
H	-1.18430100	2.40857900	1.26475300
H	-2.45626300	2.66871500	0.05820100
C	-3.73350800	0.30399900	0.24992900
C	-4.07897400	-0.06472900	-1.05533300
C	-4.77377900	0.48211800	1.18175700
C	-5.41430800	-0.26955300	-1.41497100
H	-3.29952800	-0.20179400	-1.79402700
C	-6.10548700	0.27074500	0.82814800
H	-4.54234800	0.78979600	2.19778300
C	-6.43262700	-0.10993500	-0.47565000
H	-5.65259800	-0.55673200	-2.43504100
H	-6.88600300	0.40786100	1.57079400
H	-7.46890500	-0.27464400	-0.75514800
C	-1.23477700	0.40633000	3.07966200
H	-0.78005000	-0.33159600	3.74575000
H	-0.49373500	1.17802800	2.86855100
H	-2.07141800	0.87190800	3.60883400

TS1$_{Si}$

N	-0.06569000	1.12509300	0.11668700
P	2.26170500	-1.07978900	-0.15696100
Ir	-0.06745800	-1.07735900	-0.45956000
H	-0.20390400	-1.48740300	1.37616400
C	-2.13879900	-1.65876100	0.28375100
C	-1.35388300	-1.60067100	1.60446400
C	-0.98647700	3.10369000	0.99146000
H	-1.76778800	2.88516200	1.72400600
H	-0.97200600	4.17138900	0.78282100
C	0.67060500	1.59311500	1.06795100
O	0.29496500	2.76152200	1.60111800
C	-1.04603000	2.19309100	-0.24950800
C	1.89975300	0.94358200	1.68478000
N	2.73844100	0.34957300	0.63339500
H	3.71849300	0.36414900	0.89192100
C	2.77679400	-2.49593500	0.89892500
H	2.50376100	-3.43408200	0.41101800
H	3.84963300	-2.48206200	1.10356700
H	2.23179600	-2.42905000	1.84388200
C	3.34956600	-1.12257600	-1.68093900
C	3.06179300	-2.43008900	-2.44444400
H	2.00611100	-2.51205900	-2.71926800
H	3.65399400	-2.43962900	-3.36553800
H	3.34036900	-3.31555200	-1.86510500
C	3.01289900	0.09332300	-2.56366600
H	3.69269200	0.10951900	-3.42227700
H	1.99204800	0.04201700	-2.94897900
H	3.12899800	1.03244500	-2.01639600
C	4.83783100	-1.06336700	-1.27634300
H	5.44893200	-1.11415800	-2.18357600
H	5.09260100	-0.12590500	-0.77165800
H	5.13379500	-1.89992500	-0.63773100
H	-1.46298400	-1.02821000	-1.25435800
H	0.03944000	-2.59304800	-0.86531600
C	2.72930500	1.93067600	2.54852700
H	2.01504100	2.38844400	3.23977600
C	3.38366300	3.04775000	1.72108800
H	3.89452500	3.74921000	2.38728300
H	4.12955200	2.65241800	1.02429100
H	2.64309100	3.60670700	1.14426600
C	3.76751300	1.17204300	3.38980900
H	4.25542100	1.86076500	4.08511000
H	3.30443400	0.37046200	3.97458000
H	4.55975800	0.72796900	2.77545200
H	1.51035300	0.17827200	2.37712000
C	-0.69053300	2.91486700	-1.58666700
C	0.69045500	3.59210800	-1.50369900
H	0.73335700	4.35659100	-0.72136900
H	1.47832000	2.85783100	-1.30906100
H	0.92152800	4.08371900	-2.45356400
C	-1.78604100	3.96674800	-1.85911600
H	-1.60074400	4.45496600	-2.82087400
H	-2.77641400	3.49956500	-1.90336600
H	-1.81559500	4.75180700	-1.09730100
H	-2.03209900	1.73993500	-0.34581100
C	-0.68322800	1.91507400	-2.75540900
H	0.08509400	1.15444800	-2.62273700
H	-1.64801600	1.40981100	-2.86029200
H	-0.48231800	2.44337200	-3.69306600
H	0.31154000	-0.80801100	-1.94625000
C	-1.63794800	-0.40082200	2.52243300
H	-1.84478100	0.50399100	1.95512000
H	-2.50006100	-0.60277700	3.16068700
H	-0.78169400	-0.21879500	3.17760300
C	-2.53019700	-3.05642300	-0.21016600
H	-1.69787200	-3.75856000	-0.20792300
H	-3.32626900	-3.44932300	0.43317900

H	-2.92350500	-2.99138400	-1.22664000
C	-3.35715000	-0.75623100	0.16325100
C	-3.62542900	0.02175900	-0.96869600
C	-4.35039900	-0.83861000	1.15488500
C	-4.81507600	0.74334500	-1.08930300
H	-2.89012800	0.06907600	-1.76541300
C	-5.53957600	-0.11906200	1.04114000
H	-4.20477200	-1.48271900	2.01607500
C	-5.77320600	0.68497600	-0.07712600
H	-4.98583400	1.34854100	-1.97452000
H	-6.28658700	-0.19381700	1.82562500
H	-6.69667600	1.24924500	-0.16279200
C	-1.31071300	-2.91551500	2.40591500
H	-0.90342800	-3.74230100	1.82404500
H	-0.68530600	-2.78117500	3.29271000
H	-2.31916100	-3.18176000	2.73609600

TS1$_{Re}$

N	0.91225900	1.30492600	0.10568600
P	1.14702300	-1.90094900	-0.18783400
Ir	-0.59435300	-0.32934200	-0.30477600
H	-0.77682200	-0.63395300	1.54821000
C	-2.36678900	0.67647000	0.67269400
C	-1.81125400	-0.16427600	1.84437700
C	1.71063700	3.39255100	0.80523500
H	1.05405900	3.78885700	1.58527300
H	2.43189100	4.15130100	0.51025300
C	1.86031200	1.16510900	0.97073600
O	2.46145900	2.28362500	1.38918400
C	0.93739600	2.73127100	-0.35022500
C	2.34708800	-0.14633200	1.56707900
N	2.51343900	-1.14163000	0.49214200
H	3.28205800	-1.77377000	0.68569400
C	0.68398400	-3.29116800	0.92789700
H	-0.22158000	-3.77549700	0.55685800
H	1.48637900	-4.02600900	1.02493000
H	0.46894300	-2.86933900	1.91327700
C	1.78474400	-2.67173100	-1.76887100
C	0.63243600	-3.48100600	-2.39717400
H	-0.24111700	-2.85098200	-2.58966200
H	0.97079300	-3.89479200	-3.35292400
H	0.32346800	-4.32006300	-1.76654300
C	2.23252600	-1.55783200	-2.73228800
H	2.64705600	-2.01700900	-3.63613100
H	1.39630400	-0.92411200	-3.03564100
H	3.00531900	-0.92656300	-2.28648000
C	2.98415400	-3.59485300	-1.47092600
H	3.31666800	-4.04844500	-2.41049200
H	3.83723300	-3.04349800	-1.06226500
H	2.73030500	-4.41027600	-0.78876000
H	-1.63892700	0.74738000	-0.90779300
H	-1.59352500	-1.50193100	-0.63331400
C	3.65711800	0.00798400	2.38166700
H	3.48101600	0.84353400	3.06674600
C	4.87280700	0.35285700	1.50673600
H	5.74567800	0.52820600	2.14234700
H	5.12573800	-0.46219100	0.82098700
H	4.69822400	1.25301200	0.91322000
C	3.91346200	-1.24552600	3.23257400
H	4.78164300	-1.08539500	3.87792900
H	3.05504200	-1.48136400	3.87005700
H	4.12997100	-2.12750500	2.61881000
H	1.56571000	-0.45052800	2.28351600
C	1.58530700	2.92546800	-1.75524900
C	3.02862600	2.39044600	-1.78423300
H	3.67945800	2.92192800	-1.08210700
H	3.05836900	1.32477100	-1.53818500
H	3.45392600	2.51444500	-2.78481900
C	1.57232100	4.43459400	-2.07986700
H	1.96142400	4.59556900	-3.08998600
H	0.55397600	4.83798000	-2.04563300
H	2.19382500	5.02207500	-1.39736000
H	-0.08487300	3.10453500	-0.40311600
C	0.74777500	2.20340900	-2.82366100
H	0.77734100	1.12427100	-2.68491900
H	-0.30096900	2.51890200	-2.78914800
H	1.13815800	2.43053400	-3.82090100
C	-2.62586900	-1.41471100	2.21174600
H	-3.52327600	-1.13355600	2.76755300
H	-2.92920300	-1.96758700	1.32246000
H	-2.02513200	-2.07259900	2.84579600
H	-0.29252500	-0.32854400	-1.83172700
C	-2.26458100	2.19257100	0.86160400
H	-2.95601200	2.51399500	1.64696600
H	-1.26588800	2.50651400	1.15584400
H	-2.54225000	2.71071600	-0.05856200
C	-3.76596000	0.30971900	0.19933100
C	-4.06609300	-0.19355500	-1.07110100
C	-4.83052900	0.55676300	1.08507200
C	-5.38761800	-0.45488800	-1.44429400
H	-3.26227600	-0.39064900	-1.77045000
C	-6.14807100	0.28872300	0.71793700
H	-4.63069600	0.95788100	2.07439500
C	-6.43307100	-0.22030300	-0.55171100
H	-5.59378200	-0.84456600	-2.43682800
H	-6.95088900	0.48148100	1.42333100
H	-7.45888200	-0.42773900	-0.84093200
C	-1.39696500	0.62842600	3.09728300
H	-0.98227100	-0.05663100	3.84158000
H	-0.64468700	1.38891600	2.88262500
H	-2.26950200	1.11872800	3.53881300

Int4$_{Si}$

N	0.32722600	1.25401000	-0.10096700
P	2.19762100	-1.28070300	0.23822700
Ir	0.60288700	-0.66471400	-1.13472800
H	-1.30959000	-2.27636200	0.66998100
C	-3.00085700	-1.29918500	-0.17354100
C	-2.25954100	-1.89197600	1.06490300
C	-0.97776600	2.98844400	0.81409600
H	-1.88118400	2.63595700	1.31787600
H	-0.99901400	4.07427300	0.74152400
C	0.76618900	1.59244100	1.06368400
O	0.15834100	2.62663200	1.65548000
C	-0.70211400	2.25253500	-0.51425700
C	1.83986900	0.92296200	1.89722500
N	2.72828800	0.09553900	1.08118800
H	3.64082600	-0.00802000	1.50803500
C	1.56424100	-2.50176900	1.45728700
H	1.27493200	-3.42055300	0.94335000
H	2.31699400	-2.71980200	2.21857000
H	0.67989600	-2.08152900	1.94110700
C	3.78894400	-1.97184600	-0.47631000
C	3.45322200	-3.23649100	-1.29079200
H	2.77666800	-3.01204000	-2.11801100
H	4.38217600	-3.64142000	-1.70551000
H	3.00002500	-4.01770500	-0.67296300
C	4.43462700	-0.91316100	-1.39162300
H	5.37476900	-1.31372000	-1.78498800
H	3.79052200	-0.67278300	-2.24151700
H	4.65665900	0.01317000	-0.85547800
C	4.76558000	-2.34294800	0.66298700
H	5.67996100	-2.74392500	0.21414800
H	5.06423700	-1.48352600	1.27195000
H	4.36143800	-3.11247000	1.32549000
H	-2.38612300	-0.44865600	-0.50388600
H	0.67977300	-2.07556800	-1.87484800
C	2.66036800	1.97548800	2.69821500
H	1.92067600	2.54718800	3.26645800
C	3.42094800	2.94295500	1.78152400
H	3.97457300	3.67002200	2.38259900
H	4.13896900	2.41466300	1.14667700
H	2.73760900	3.49748400	1.13249600
C	3.59224000	1.29331600	3.71067900
H	4.03248600	2.04677600	4.36971300
H	3.05351200	0.57073800	4.33225400
H	4.42876500	0.77216900	3.23018700
H	1.28485800	0.32377200	2.63981200
C	-0.23557900	3.14919900	-1.69723400
C	1.01518000	3.96361400	-1.32329400
H	0.83595100	4.64084700	-0.48205700
H	1.84829700	3.30273900	-1.06551900
H	1.32792600	4.57634000	-2.17417700
C	-1.40199400	4.09029400	-2.06049300
H	-1.12382100	4.71536200	-2.91434900
H	-2.29631300	3.52094500	-2.33787300
H	-1.66868200	4.76204800	-1.23848700
H	-1.59247300	1.70637700	-0.84081800
C	0.08823100	2.27152000	-2.91944600
H	0.99394100	1.68280100	-2.76155200
H	-0.73669100	1.58605500	-3.15362200
H	0.25146300	2.90063800	-3.80043400
H	1.72007700	-0.23057000	-2.09365900
C	-1.94087200	-0.80793300	2.10295700
H	-1.36273900	0.00894900	1.66253700
H	-2.85959700	-0.37893900	2.51594300
H	-1.36296300	-1.21899400	2.93805300
C	-3.03774800	-2.29428000	-1.34671400
H	-2.02159000	-2.61508900	-1.60296200
H	-3.61783500	-3.19128100	-1.11269900
H	-3.48672700	-1.83370900	-2.23213300
C	-4.37059600	-0.72690900	0.16187800
C	-4.52989700	0.65360500	0.35395600
C	-5.50383500	-1.54338600	0.30600000
C	-5.76894100	1.20451800	0.68771300
H	-3.67229500	1.31049700	0.23366500
C	-6.74519500	-0.99966500	0.64189300
H	-5.41997900	-2.61477000	0.15585400
C	-6.88356300	0.37692700	0.83625100
H	-5.86243200	2.27780300	0.82763900
H	-7.60615500	-1.65345500	0.74966000
H	-7.84993400	0.79941800	1.09532900
C	-2.97840900	-3.07155500	1.73388200
H	-3.20526300	-3.87345000	1.02529300
H	-2.34757700	-3.49495400	2.52254000
H	-3.91732000	-2.75038100	2.19552800

Int4$_{Re}$

N	0.63621600	1.35447800	0.02214100
P	1.88009700	-1.60031400	-0.06919600
Ir	0.36303700	-0.47471100	-1.18900100
H	-1.30111400	-1.13445600	1.34425900
C	-2.99457100	-0.01379300	0.67239400
C	-2.37473200	-1.34503800	1.20512800
C	-0.00098000	3.36465700	1.10212900
H	-1.00857700	3.48386700	1.50053300
H	0.52451000	4.31712200	1.14964300
C	1.00331100	1.35306300	1.25776700
O	0.71428800	2.44165400	1.97631000
C	0.03626800	2.68370400	-0.28983600
C	1.66481400	0.21523400	2.01890600
N	2.51516100	-0.58127200	1.13076500
H	3.33235000	-0.94611400	1.60487500
C	1.15262400	-3.07282000	0.75177700
H	0.79925000	-3.77946900	-0.00129400
H	1.88127000	-3.55450100	1.40825100
H	0.29691700	-2.74407300	1.34522800
C	3.40587500	-2.16040700	-1.00300000
C	2.96187100	-3.03282500	-2.19386300
H	2.33283300	-2.47231700	-2.88864900
H	3.85358200	-3.37158600	-2.73163000
H	2.41242400	-3.92270400	-1.87171100
C	4.16913000	-0.92091500	-1.51093000
H	5.05329200	-1.25505500	-2.06388700
H	3.55804000	-0.31758500	-2.18774500
H	4.50128600	-0.28093600	-0.68976100
C	4.32125300	-2.98766600	-0.07326100
H	5.21331500	-3.27918600	-0.63724500
H	4.66785800	-2.42289900	0.79831000
H	3.83884700	-3.90440600	0.27441900
H	-2.44438800	0.22496800	-0.25172900
H	0.04305400	-1.78575500	-2.03352300
C	2.48199900	0.73047300	3.23457300
H	1.78760600	1.34558200	3.81456800
C	3.66962600	1.60889100	2.81391100
H	4.17866400	1.99446500	3.70198000
H	4.40363800	1.04431900	2.23022800
H	3.34410900	2.46318500	2.21415900
C	2.92763900	-0.43556600	4.12963100
H	3.37468000	-0.04571400	5.04828800
H	2.08427900	-1.07625900	4.40690900
H	3.68743700	-1.06428600	3.65070100
H	0.82844200	-0.37923300	2.42526400
C	0.82004600	3.47433600	-1.37796300
C	2.29807000	3.63264000	-0.97997700
H	2.41828700	4.19099600	-0.04525100
H	2.77606600	2.65583000	-0.85818300

H	2.83861100	4.18095700	-1.75747900
C	0.15173300	4.85633000	-1.53815900
H	0.64476500	5.41478400	-2.33954200
H	-0.90703700	4.75486200	-1.80235300
H	0.21858400	5.46532100	-0.63151300
H	-0.98010100	2.51119500	-0.65938000
C	0.72952600	2.74083400	-2.72792300
H	1.26998700	1.79165500	-2.70742600
H	-0.31342400	2.54252600	-3.00127700
H	1.16922200	3.35574600	-3.51984300
C	-2.50408300	-2.48163700	0.18202800
H	-3.55255700	-2.76285400	0.04301400
H	-2.10212800	-2.18830700	-0.79349400
H	-1.95859500	-3.37012200	0.51535300
H	1.45710600	-0.24606100	-2.23810500
C	-2.76320100	1.15524500	1.64287300
H	-3.33822400	1.04940600	2.56587300
H	-1.70891800	1.21575900	1.92922100
H	-3.05539100	2.10414700	1.18267200
C	-4.45393400	-0.15871500	0.26275100
C	-4.78029200	-0.37598700	-1.08446800
C	-5.50398600	-0.11133100	1.19324100
C	-6.10398300	-0.54833500	-1.49318700
H	-3.98314100	-0.41539200	-1.82316400
C	-6.83029100	-0.28445600	0.79078400
H	-5.29150800	0.06271500	2.24305400
C	-7.13664400	-0.50477000	-0.55390600
H	-6.32733300	-0.71437200	-2.54333100
H	-7.62500600	-0.24519300	1.53052500
H	-8.16831600	-0.63746200	-0.86636200
C	-2.92451100	-1.79424300	2.56644000
H	-2.40572300	-2.69923300	2.89980900
H	-2.79226100	-1.03266500	3.34043400
H	-3.99127100	-2.03082800	2.49930400

Int5

N	1.32210100	-0.02000400	-0.47223300
P	-1.85012000	0.03260300	-0.21143900
Ir	-0.31957200	-1.46415600	-0.68687900
C	3.40942700	0.89373800	-1.05981400
H	3.64112300	0.78545600	-2.12209600
H	4.28211400	1.27006600	-0.53000300
C	1.22509300	1.25004200	-0.68057300
O	2.36116100	1.90300300	-0.94170800
C	2.77254900	-0.37420300	-0.44881400
C	-0.04116700	2.08738600	-0.71770100
N	-1.08105900	1.50709600	0.13455700
H	-1.69493100	2.21456400	0.51993500
C	-2.97242900	0.28373400	-1.64428800
H	-3.56934700	-0.61613500	-1.80538400
H	-3.62646600	1.14413000	-1.48597200
H	-2.36043800	0.45774600	-2.53309100
C	-2.91345000	-0.24201400	1.30571900
C	-3.62903900	-1.60009200	1.16578100
H	-2.91630600	-2.42360200	1.08565800
H	-4.24769800	-1.76414400	2.05435300
H	-4.29005700	-1.62968800	0.29402000
C	-2.00987500	-0.24294700	2.55483400
H	-2.63282100	-0.40590400	3.44059400
H	-1.26549300	-1.04287400	2.51566700
H	-1.48230700	0.70661100	2.67545500
C	-3.96799100	0.88182200	1.42876000
H	-4.56269800	0.69731200	2.32937500
H	-3.52402300	1.87632300	1.54177600
H	-4.65750200	0.90178600	0.58075200
H	-1.46398200	-2.52523500	-1.00954800
C	0.23541200	3.56041700	-0.30482000
H	1.04682000	3.89864900	-0.95629700
C	0.70277400	3.68507600	1.15270000
H	0.93647700	4.72923900	1.37986700
H	-0.06962300	3.35644700	1.85528100
H	1.60228800	3.09142300	1.33683300
C	-0.98544200	4.44805700	-0.58926600
H	-0.72425500	5.49770200	-0.42833300
H	-1.33105000	4.33862400	-1.62237100
H	-1.82924100	4.22811200	0.07556400
H	-0.34254700	2.10789300	-1.77865900
C	3.27915800	-0.74771800	0.97653500
C	3.03996300	0.40558800	1.96788800
H	3.56597200	1.31999800	1.67385300
H	1.97326500	0.63432300	2.05454000
H	3.40323900	0.12525000	2.96129700
C	4.78563900	-1.06591600	0.88051600
H	5.15997700	-1.37976100	1.85966400
H	4.97322700	-1.88096000	0.17252800
H	5.37992300	-0.20152000	0.56851500
H	2.93068600	-1.23594400	-1.10390000
C	2.54882200	-2.00683800	1.47642700
H	1.48990100	-1.80924600	1.65588100
H	2.62708400	-2.82688800	0.75274500
H	2.99079800	-2.34849200	2.41796200
H	-0.50655900	-1.97072200	0.74886300

S3

C	-1.03245100	0.54036300	0.16506400
C	-1.97676300	-0.36946700	-0.15787000
C	-1.65339200	-1.74066500	-0.71009300
H	-0.59412900	-1.87899500	-0.92829700
H	-1.96125900	-2.52652700	-0.00742300
H	-2.22022900	-1.91563400	-1.63406100
C	-1.31316000	1.94323400	0.66397100
H	-2.37349000	2.19687600	0.68383700
H	-0.91128600	2.07777600	1.67637900
H	-0.80750900	2.68374200	0.03261200
C	0.43015300	0.23159700	0.07652500
C	1.26578100	0.95289700	-0.79264100
C	1.01316700	-0.75127600	0.89303400
C	2.63494000	0.68909600	-0.85615500
H	0.83560700	1.71753500	-1.43402800
C	2.38295700	-1.01617400	0.83404300
H	0.37996200	-1.30895600	1.57724700
C	3.19929600	-0.29672000	-0.04189300
H	3.26118900	1.25163300	-1.54318000
H	2.81221200	-1.78186200	1.47443600
H	4.26517400	-0.50080400	-0.08894800
C	-3.46509300	-0.13557000	-0.03572100
H	-3.73327700	0.80866800	0.43872400
H	-3.94090100	-0.16355600	-1.02512700
H	-3.92466600	-0.94378700	0.54825300

S3-H_2-(*R*)

H	3.08647300	0.26607000	-0.29555400
C	1.09125100	-0.22582600	-0.87769100
C	2.08995300	0.39229500	0.15255400
H	1.17730900	0.39844500	-1.77761700
C	1.85158800	1.89786800	0.33632600
H	1.88544600	2.42804200	-0.62213900
H	0.87200100	2.08872200	0.78777800
H	2.61357000	2.33480700	0.99075000
C	1.50066200	-1.65039700	-1.29044800
H	2.52232800	-1.65250300	-1.68512900
H	1.47149100	-2.35402100	-0.45355900
H	0.83362000	-2.03553900	-2.06805700
C	-0.36106200	-0.13809000	-0.42757300
C	-1.16743300	0.92448600	-0.86376500
C	-0.93537500	-1.08320300	0.43750600
C	-2.49558900	1.04713000	-0.45011200
H	-0.74579300	1.66579000	-1.53806600
C	-2.26307100	-0.96651500	0.85493100
H	-0.34328800	-1.92135800	0.79078300
C	-3.04994500	0.09996900	0.41369700
H	-3.09674800	1.87947000	-0.80584300
H	-2.68325500	-1.71226700	1.52435800
H	-4.08340400	0.18918900	0.73612300
C	2.10069900	-0.31537000	1.51539600
H	2.29980800	-1.38761200	1.42445400
H	2.87869000	0.11196900	2.15706200
H	1.14169900	-0.19328500	2.02971900

S3-H_2-(*S*)

H	3.08649500	0.26625900	0.29590900
C	1.09114200	-0.22561300	0.87774600
C	2.09005100	0.39227400	-0.15244400
C	2.10112000	-0.31566900	-1.51511300
H	2.30037800	-1.38786400	-1.42397600
H	1.14213100	-0.19381000	-2.02950600
H	2.87910600	0.11169000	-2.15679000
C	1.50038700	-1.65014600	1.29078300
H	1.47141800	-2.35382300	0.45392300
H	2.52191700	-1.65227900	1.68579300
H	0.83307700	-2.03520600	2.06821000
C	-0.36104800	-0.13796400	0.42745300
C	-0.93524100	-1.08308400	-0.43767900
C	-1.16755600	0.92450100	0.86372400
C	-2.26295500	-0.96650800	-0.85509900
H	-0.34309300	-1.92118200	-0.79099300
C	-2.49572100	1.04700600	0.45010700
H	-0.74597300	1.66580500	1.53806500
C	-3.04996500	0.09983000	-0.41377400
H	-2.68302400	-1.71228600	-1.52457100
H	-3.09699900	1.87923300	0.80591000
H	-4.08343900	0.18896600	-0.73617800
C	1.85149800	1.89776300	-0.33664600
H	1.88489900	2.42819400	0.62172100
H	2.61363300	2.33475500	-0.99086700
H	0.87204400	2.08838700	-0.78853600
H	1.17715100	0.39882800	1.77753300

H_2

H	0.00000000	0.00000000	0.37155000
H	0.00000000	0.00000000	-0.37155000

11. References

(1) (a) Becke, A. D. Density-functional thermochemistry. III. The role of exact Exchange. *J. Chem. Phys.* **1993**, *98*, 5648−5652. (b) Stephens, P. J.; Devlin, F. J.; Chabalowski, C. F.; Frisch, M. J. Ab Initio Calculation of Vibrational Absorption and Circular Dichroism Spectra Using Density Functional Force Fields. *J. Phys. Chem.* **1994**, *98*, 11623−11627.

(2) Grimme, S.; Antony, J.; Ehrlich, S.; Krieg, H. A consistent and accurate ab initio parametrization of density functional dispersion correction (DFT-D) for the 94 elements H-Pu. *J. Chem. Phys.* **2010**, *132*, 154104.

(3) Petersson, G. A.; Bennett, A.; Tensfeldt, T. G.; Al-Laham, M. A.; Shirley, W. A.; Mantzaris, J. "A complete basis set model chemistry. I. The total energies of closed-shell atoms and hydrides of the first-row atoms," *J. Chem. Phys.* **1988**, 89, 2193–2218.

(4) (a) Hay, P. J.; Wadt, W. R. Ab initio effective core potentials for molecular calculations. Potentials for the transition metal atoms Sc to Hg. *J. Chem. Phys.* **1985**, *82*, 270−283. (b) Hay, P. J.; Wadt, W. R. Ab initio effective core potentials for molecular calculations. Potentials for K to Au including the outermost core orbitals. *J. Chem. Phys.* **1985**, *82*, 299−310.

(5) (a) Brandt, P.; Hedberg, C.; Andersson, P. G. New Mechanistic Insights into the Iridium−Phosphanooxazoline-Catalyzed Hydrogenation of Unfunctionalized Olefins: A DFT and Kinetic Study. *Chem. − Eur. J.* **2003**, *9*, 339−347. (b) Fan, Y.; Cui, X.; Burgess, K.; Hall, M. B. Electronic effects steer the mechanism of asymmetric hydrogenations of unfunctionalized aryl-substituted alkenes. *J. Am. Chem. Soc.* **2004**, *126*, 16688−16689. (c) Cui, X.; Fan, Y.; Hall, M. B.; Burgess, K. Mechanistic Insights into Iridium-Catalyzed Asymmetric Hydrogenation of Dienes. *Chem. − Eur. J.* **2005**, *11*, 6859−6868. (d) Church, T. L.; Rasmussen, T.; Andersson, P. G. Enantioselectivity in the Iridium-Catalyzed Hydrogenation of Unfunctionalized Olefins. *Organometallics* **2010**, *29*, 6769−6781. (e) Hopmann, K. H.; Bayer, A. On the mechanism of iridium-catalyzed asymmetric hydrogenation of imines and alkenes: A theoretical study. *Organometallics* **2011**, *30*, 2483−2497. (f) Mazuela, J.; Norrby, P.-O.; Andersson, P. G.; Pàmies, O.; Diéguez, M. Pyranoside Phosphite−Oxazoline Ligands for the Highly Versatile and Enantioselective Ir-Catalyzed Hydrogenation of Minimally Functionalized Olefins. A Combined Theoretical and Experimental Study. *J. Am. Chem. Soc.* **2011**, *133*, 13634−13645. (g) Gruber, S.; Pfaltz, A. Asymmetric hydrogenation with iridium C, N and N, P ligand complexes: characterization of dihydride intermediates with a coordinated alkene. *Angew. Chem., Int. Ed.* **2014**, *53*, 1896−1900.

(6) (a) Aguado-Ullate, S.; Saureu, S.; Guasch, L.; Carbó, J. J. Theoretical Studies of Asymmetric Hydroformylation Using the Rh−(R,S)-BINAPHOS Catalyst − Origin of Coordination Preferences and Stereoinduction. *Chem. − Eur. J.* **2012**, *18*, 995−1005. (b) Aguado- Ullate, S.; Urbano-Cuadrado, M.; Villalba, I.; Pires, E.; García, J. I.; Bo, C.; Carbó, J. J. Predicting the Enantioselectivity of the Copper-

Catalysed Cyclopropanation of Alkenes by Using Quantitative Quadrant-Diagram Representations of the Catalysts. *Chem. − Eur. J.* **2012**, *18*, 14026−14036.

(7) Dunning, T. H. Gaussian basis sets for use in correlated molecular calculations. I. The atoms boron through neon and hydrogen. *J. Chem. Phys.* **1989**, *90*, 1007–1023.

(8) Woon, D. E.; Dunning, T. H. Gaussian basis sets for use in correlated molecular calculations. III. The atoms aluminum through argon. *J. Chem. Phys.* **1993**, *98*, 1358–1371.

(9) Figgen, D.; Peterson, K. A.; Dolg, M.; Stoll, H. Energy-consistent pseudopotentials and correlation consistent basis sets for the 5d elements Hf-Pt. *J. Chem. Phys.* **2009**, *130*, 164108.

(10) Pritchard, B.P.; Altarawy, D.; Didier, B.; Gibson, T.D.; Windus, T.L.. *A New Basis Set Exchange: An Open, Up-to-date Resource for the Molecular Sciences Community. J. Chem. Inf. Model.* **2019**, *59*, 4814–4820.

(11) Hopmann, K. H. How Accurate is DFT for Iridium-Mediated Chemistry? *Organometallics*, **2016**, *35*, 3795–3807.

(12) Grimme, S. Semiempirical GGA-type density functional constructed with a long-range dispersion correction, *J. Comp. Chem.* **2006**, *27*, 1787–1799.

(13) Grimme, S.; Ehrlich S.; Goerigk, F. Effect of the damping function in dispersion corrected density functional theory. *J. Comp. Chem.* **2011**, *32*, 1456–1465.

(14) Biosca, M.; Paptchikhine, A.; Pam̀ies, O.; Andersson, P. G.; Dieǵuez, M. Extending the Substrate Scope of Bicyclic P-Oxazoline/Thiazole Ligands for Ir-Catalyzed Hydrogenation of Unfunctionalized Olefins by Introducing a Biaryl Phosphoroamidite Group. *Chem. - Eur. J.* **2015**, *21*, 3455−3464.

(15) Gupton, J. T.; Layman, W. J. Reaction of 2-aryl-3-(N,N-dimethylamino)-1-propenes and their corresponding quaternary ammonium salts with organometallic species and reducing agents. *J. Org. Chem.* **1987**, *52*, 3683–3686.

(16) Biosca, M.; Salomó, E.; de la Cruz-Sánchez, P.; Riera, A.; Verdaguer, X.; Pàmies, O.; Diéguez, M. Extending the Substrate Scope in the Hydrogenation of Unfunctionalized Tetrasubstituted Olefins with Ir-P Stereogenic Aminophosphine–Oxazoline Catalysts. *Org. Lett.* **2019**, *21*, 807–811.

(17) Berthiol, F.; Doucet, H.; Santelli, M. Synthesis of Polysubstituted Alkenes by Heck Vinylation or Suzuki Cross-Coupling Reactions in the Presence of a Tetraphosphane−Palladium Catalyst. *Eur. J. Org. Chem.* **2003**, 1091–1096.

(18) Yanagisawa, A.; Nezu, T.; Mohri, S.-I. Brønsted Acid-Promoted Hydrocyanation of Arylalkenes. *Org. Lett.* **2009**, *11*, 5286–5289.

(19) Schrems, M. G.; Pfaltz, A. NeoPHOX—an easily accessible P,N-ligand for iridium-catalyzed asymmetric hydrogenation: preparation, scope and application in the synthesis of demethyl methoxycalamenene. *Chem. Commun.* **2009**, 6210–6212.

(20) Tolstoy, P.; Engman, M.; Paptchikhine, A.; Bergquist, J.; Church, T. L.; Leung, A. W. M.; Andersson, P. G. Iridium-Catalyzed Asymmetric Hydrogenation Yielding Chiral Diarylmethines with Weakly Coordinating or Noncoordinating Substituents. *J. Am. Chem. Soc.* **2009**, *131*, 8855–8860.
(21) Terao, Y.; Nomoto, M.; Satoh, T.; Miura, M.; Nomura, M. Palladium-Catalyzed Dehydroarylation of Triarylmethanols and Their Coupling with Unsaturated Compounds Accompanied by C−C Bond Cleavage. *J. Org. Chem.* **2004**, *69*, 6942–6944.
(22) Xia, J.; Guoqiang, Y.; Zhuge, R.; Liu, Y.; Zhang, W. Iridium-Catalyzed Asymmetric Hydrogenation of Unfunctionalized Exocyclic C=C Bonds. *Chem. Eur. J.* **2016**, *22*, 18354–18359.
(23) Biosca, M.; de la Cruz-Sánchez, P.; Tarr, D.; Llanes, P.; Karlsson, E. A.; Margalef, J.; Pàmies, O.; Pericàs, M. A.; Diéguez, M. Filling the Gaps in the Challenging Asymmetric Hydrogenation of Exocyclic Benzofused Alkenes with Ir−P,N Catalysts. *Adv. Synth. Catal.* **2022**, https://doi.org/10.1002/adsc.202200870.
(24) Too, P. C.; Noji, T.; Lim, Y. J.; Li, X.; Chiba, S. Rhodium(III)-Catalyzed Synthesis of Pyridines from α,β-Unsaturated Ketoximes and Internal Alkynes. *Synlett* **2011**, 2789–2794.
(25) Lu, S. M.; Bolm, C. Highly Enantioselective Synthesis of Optically Active Ketones by Iridium-Catalyzed Asymmetric Hydrogenation. *Angew. Chem. Int. Ed.* **2008**, *47*, 8920–8923.
(26) Ponra, S.; Yang, J.; Kerdphon, S.; Andersson, P. G. Asymmetric Synthesis of Alkyl Fluorides: Hydrogenation of Fluorinated Olefins. *Angew. Chem. Int. Ed.* **2019**, *58*, 9282–9287.
(27) Lu, S.-M.; Bolm, C. Highly Chemo- and Enantioselective Hydrogenation of Linear α,β-Unsaturated Ketones. *Chem. - Eur. J.* **2008**, *14*, 7513–7516.
(28) Ganić, A.; Pfaltz, A. Iridium-Catalyzed Enantioselective Hydrogenation of Alkenylboronic Esters. *Chem. Eur. J.* **2012**, *18*, 6724–6728.
(29) Cheruku, P.; Diesen, J.; Andersson, P. G. Asymmetric Hydrogenation of Di and Trisubstituted Enol Phosphinates with N,P- Ligated Iridium Complexes. *J. Am. Chem. Soc.* **2008**, *130*, 5595–5599.
(30) Galambos, J.; Wágner, G.; Nógrádi, K.; Bielik, A.; Molnár, L.; Bobok, A.; Horváth, A.; Kiss, B.; Kolok, S.; Nagy, J.; Kurkó, D.; Bakk, M. L.; Vastag, M.; Sághy, K.; Gyertyán, I.; Gál, K.; Greiner, I.; Szombathelyi, Z.; Keserű, G. M.; Domány, G. Carbamoyloximes as novel non-competitive mGlu5 receptor antagonists. *Bioorg. Med. Chem. Lett.* **2010**, *20*, 4371–4375.
(31) Peters, B. B. C.; Jongcharoenkamol, J.; Krajangsri, S.; Andersson, P. G. Highly Enantioselective Iridium-Catalyzed Hydrogenation of Conjugated Trisubstituted Enones. *Org. Lett.* **2021**, *23*, 242–246.
(32) Fengtao, T.; Dongmei, Y.; Yuanyuan, L.; Fang, X.; Wanbin, Z. Iridium-Catalyzed Highly Enantioselective Hydrogenation of Exocyclic α,β-Unsaturated Carbonyl Compounds. *Adv. Synth. Catal.* **2010**, *352*, 1841–1845.
(33) Margalef, J.; Biosca, M.; de la Cruz-Sánchez, P.; Caldentey, X.; Rodríguez-Escrich, C.; Pàmies, O.; Pericàs, M. A.; Diéguez, M. Indene Derived Phosphorus-Thioether Ligands for the Ir-Catalyzed

Asymmetric Hydrogenation of Olefins with Diverse Substitution Patterns and Different Functional Groups. *Adv. Synth. Catal.* **2021**, *363*, 4561–4574.

(34) Liu, X.; Han, Z.; Wang, Z.; Ding, K. SpinPhox/Iridium(I)-Catalyzed Asymmetric Hydrogenation of Cyclic α-Alkylidene Carbonyl Compounds. *Angew. Chem. Int. Ed.* **2014**, *53*, 1978–1982.

(35) Ponra, S.; Rabten, W.; Yang, J.; Wu, H.; Kerdphon, S.; Andersson, P. G. Diastereo-and enantioselective synthesis of fluorine motifs with two contiguous stereogenic centers. *J. Am. Chem. Soc.* **2018**, *140*, 13878–13883.

(36) Kerdphon, S.; Ponra, S.; Yang, J.; Wu, H.; Eriksson, L.; Andersson, P. G. Diastereo- and Enantioselective Synthesis of Structurally Diverse Succinate, Butyrolactone, and Trifluoromethyl Derivatives by Iridium-Catalyzed Hydrogenation of Tetrasubstituted Olefins. *ACS Catal.* **2019**, *9*, 6169−6176.

(37) Nakatsuji, H.; Ashida, Y.; Hori, H.; Sato, Y.; Honda, A.; Taira, M.; Tanabe, Y. (E)- and (Z)-stereodefined enol phosphonates derived from β-ketoesters: stereocomplementary synthesis of fully-substituted α,β-unsaturated esters. *Org. Biomol. Chem.*, **2015**, *13*, 8205–8210.

(38) Bigler, R.; Mack, K. A.; Shen, J.; Tosatti, P.; Han, C.; Bachmann, S.; Zhang, H.; Scalone, M.; Pfaltz, A.; Denmark, S. E.; Hildbrand, S.; Gosselin, F. Asymmetric Hydrogenation of Unfunctionalized Tetrasubstituted Acyclic Olefins. *Angew. Chem. Int. Ed.* **2020**, *59*, 2844–2849.

(39) Lightfoot, A.; Schnider, P.; Pfaltz, A. Enantioselective Hydrogenation of Olefins with Iridium–Phosphanodihydrooxazole Catalysts. *Angew. Chem. Int. Ed.* **1998**, *37*, 2897–2899.

(40) Källström, K.; Hedberg, C.; Brandt, P.; Bayer, A.; Andersson, P. G. Rationally Designed Ligands for Asymmetric Iridium-Catalyzed Hydrogenation of Olefins. *J. Am. Chem. Soc.* **2004**, *126*, 14308–14309.

(41) Woodmansee, D. H.; Muller, M.-A.; Neuburger, M.; Pfaltz, A. Chiral pyridyl phosphinites with large aryl substituents as efficient ligands for the asymmetric iridium-catalyzed hydrogenation of difficult substrates. *Chem. Sci.* **2010**, *1*, 72–78.

(42) Wang, A.; Fraga, R. P. A.; Hörmann, E.; Pfaltz, A. Iridium-Catalyzed Asymmetric Hydrogenation of Unfunctionalized, Trialkyl-Substituted Olefins. *Chem. Asian J.* **2011**, *6*, 599–606.

(43) Bell, S.; Wüstenberg, B.; Kaiser, S.; Menges, F.; Netscher, F.; Netscher, T.; Pfaltz, A. Asymmetric Hydrogenation of Unfunctionalized, Purely Alkyl-Substituted Olefins. *Science*, **2006**, *311*, 642–644.

(44) Lu, W. J.; Hou, X. L. Highly Enantioselective Construction of the α-Chiral Center of Amides via Iridium-Catalyzed Hydrogenation of α,β-Unsaturated Amides. *Adv. Synth. Catal.* **2009**, *351*, 1224–1228.

(45) Cheruku, P.; Gohil, S.; Andersson, P. G. Asymmetric hydrogenation of enol phosphinates by iridium catalysts having N,P ligands. *Org. Lett.* **2007**, *9*, 1659–1661.

(46) Biosca, M.; Magre, M.; Coll, M.; Pàmies, O.; Diéguez, M. Alternatives to Phosphinooxazoline (t-BuPHOX) Ligands in the Metal-Catalyzed Hydrogenation of Minimally Functionalized Olefins and Cyclic β-Enamides. *Adv. Synth. Catal.* **2017**, *359*, 2801–2814.

(47) Durkin, K.; Liotta, D.; Rancourt, J.; Lavallee, J. F.; Boisvert, L.; Guindon, Y. Stereoselective hydrogen-transfer reactions involving acyclic radicals. A study of radical conformations using semiempirical calculations. *J. Am. Chem. Soc.* **1992**, 114, 4912–4914.

(48) Malmedy, F.; Wirth, T. Stereoselective Ketone Rearrangements with Hypervalent Iodine Reagents. *Chem. Eur. J.* **2016**, *22*, 16072–16077.
